# Carbohydrate‐Mediated Cellular Uptake of Boronic Acid Hybrids *In Cellulo* and Insulin‐Deficient Zebrafish: Live Imaging and Application of Multiphoton FLIM

**DOI:** 10.1002/advs.77481

**Published:** 2026-09-10

**Authors:** Haobo Ge, John S. Fossey, David Gonzalez Calatayud, Stephen E. Flower, Sabrina Wang, Hanna Aitessaid, Melita Tvardauskaite, Julia Dudzic, Max Jewell, Charareh Pourzand, Stanley W. Botchway, David Gurevich, Tony D. James, Sofia I. Pascu

**Affiliations:** ^1^ Department of Chemistry University of Bath Bath UK; ^2^ School of Chemistry University of Birmingham Birmingham UK; ^3^ Department of Inorganic Chemistry Universidad Autónoma de Madrid Madrid Spain; ^4^ Instituto De Cerámica y Vidrio CSIC Madrid Spain; ^5^ Merry Life Biomedical Company Ltd. Tainan Taiwan; ^6^ Department of Life Sciences University of Bath Bath UK; ^7^ Centre of Therapeutic Innovation University of Bath Bath UK; ^8^ Central Laser Facility Research Complex at Harwell STFC Rutherford Appleton Laboratory Oxfordshire Didcot UK; ^9^ Centre For Bioengineering & Biomedical Technologies (CBio) University of Bath Bath UK; ^10^ School of Chemistry and Chemical Engineering Henan Normal University Xinxiang China

**Keywords:** biochemistry, biophysics, boronic acid, chemistry, confocal, fluorescence microscope, live cell imaging, zebrafish

## Abstract

Understanding how synthetic molecular probes interact with biological carbohydrates is essential for theranostics design and discovery. We report new supramolecular polysaccharide complexes, comprising simultaneously functionalised boronic acids conjugated to xanthate‐based fluorescent tags bound to saccharides such as glucose or β‐D‐glucan (a natural product from *Hordeum Vulgare*). These hybrids were investigated in solution and in the cellular milieu using advanced biophysical methods, including multiphoton fluorescence lifetime imaging microscopy (MP‐FLIM). A new type of fluorescent hybrid nanoparticle was also generated by conjugating boronic acids to ─Si(OH)_2_ functionalities on the surface of core–shell Fe_3_O_4_@SiO_2_ nanoparticles. Cellular uptake and distribution were probed using correlated confocal and multiphoton fluorescence microscopy using 2‐photon excitation at 910 nm, whereby TCSPC and FLIM provided insight into probe environments, interactions with β‐D‐glucan, and cellular context. Colocalization studies with a range of standard dyes were carried out to assess the potential of the probes for cellular penetration and targeting cellular organelles, and the nature of the lipophilic linker involved greatly influences the cytoplasmic distribution and facilitates zebrafish uptake. Complementary in vivo investigations using fluorescence stereomicroscopy of zebrafish showed differential uptake and quenching of these complexes throughout the digestive system, dependent on exogenous and endogenous concentrations of carbohydrates, glucose, or β‐d‐glucan.

## Graphical abstract

Boronic acid‐based fluorescent hybrids have been used as part of a versatile platform for real‐time metabolic bioimaging across cellular and organismal scales. Including glucose‐responsive imaging in living cells and in an insulin‐deficient vertebrate model, this work opens new opportunities for the development of precision diagnostics, metabolic disease monitoring, and future theranostic technologies capable of both visualising and ultimately modulating dysregulated carbohydrate pathways.

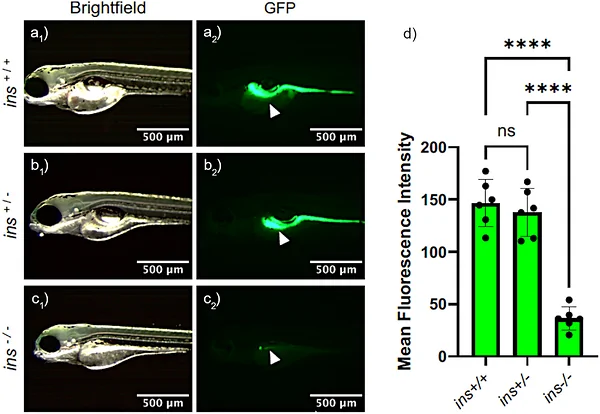

## 1 Introduction

**SCHEME 1:**
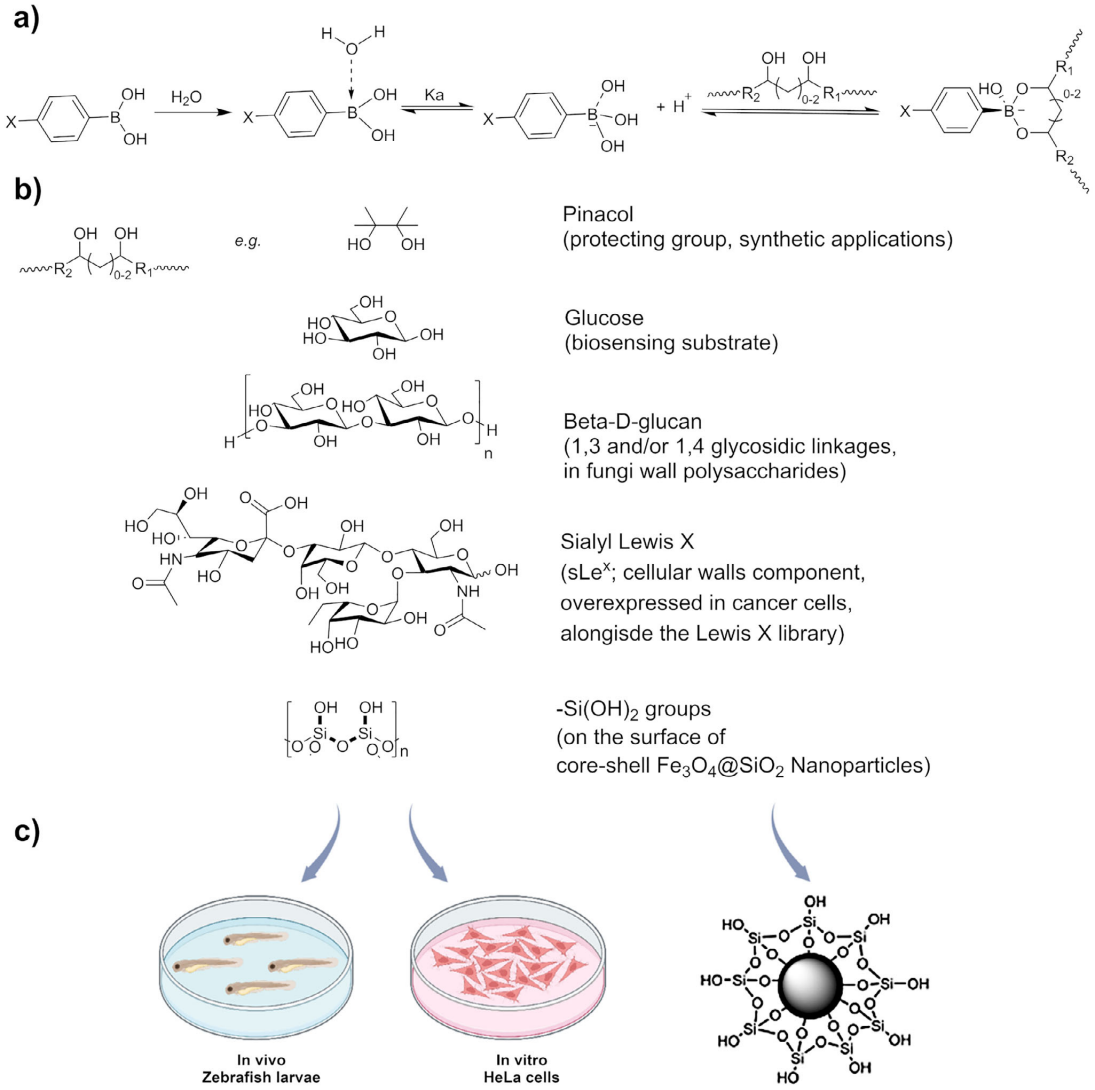
(a) General representation of phenyl‐boronic acids and reversible binding with the diols present in scaffold molecules. (b) Boronic acid fluorophores bind to diol‐containing substrates such as pinacol, mono/polysaccharides (e.g., glucose units in glycosylated biomolecules, biopolymers such as β‐d‐glucan, or to cell surfaces. Sialyl Lewis X is overexpressed in cancerous tissues and in living systems. The boronate group is likely to be negatively charged in a physiological environment. (c) Compounds 1–6 were utilised as boronic acid tags for in vitro (in living HeLa, MCF‐7, PC‐3, CHO or FEK‐4 cell lines) or in vivo (larval zebrafish) imaging assays and were shown to bind to −Si(OH)_2_ groups in Fe_3_O_4_@SiO_2_ magnetic nanoparticles in dispersed aqueous phases.

Boronic acid derivatives have attracted significant research attention due to their influence within a plethora of biomedical applications emergent from their inherent low toxicity [1] and biocompatibility [2]. One of their most prominent uses is as chemical sensors for biomolecules [3]. It is well established that the equilibrium between boronic acid and diols shifts in response to changes in pH and temperature [4]. While the addition of free diols to a boronic acid solution influences this reversible reaction, altering the pH and solubility [5] (Scheme 1). This fundamental principle underlies the molecular sensing of various diol‐containing biomolecules, including saccharides [6, 7, 8], ATPs [9], nucleotides [10], and heavy metal ions [11, 12]. Boronic acid derivatives bind selectively and reversibly to 1,2‐ and 1,3‐diol‐containing molecules, forming boronic esters that can, in turn, modulate the fluorescence properties of appended fluorophores [4]. As illustrated in Scheme 1, phenylboronic acid (pKa ≈ 9) [5] serves as a simple model for reversible boronic acid binding to saccharides. In a basic solution, boronic acid groups react with diols to form boronic esters with five‐ or six‐membered rings, while neutral or acidic conditions binding is negligible [14]. This process forms either an ester (via a single hydroxyl group) or a more stable diester (via two hydroxyl groups). However, fluorescence based systems can also compromise the selectivity of boronic acid‐based probes for target living cells, as certain fluorophores are also known to dominate the uptake and biolocalisation. To address this, we explored the incorporation of boronic esters as cross‐linking motifs in the design of fluorescence imaging probes, minimizing non‐specific binding to diol‐containing biomolecules in living cells.

The ability of boronic acids to recognise mono‐ or polysaccharides has applications in the targeting of cancer, with the cell‐surface, e.g. tetrasaccharide Sialyl Lewis X, being overexpressed in cancerous tissues and providing a good indication of malignancy: targeting this factor can be a useful tool for early disease diagnosis [19, 20, 21, 22]. It has also been reported that boronic acids can inhibit cell proliferation in prostate cancer cell lines [23, 24, 25]. Additionally, many cancer cells overexpress the sodium‐dependent multivitamin transporter (SMVT) receptors, which selectively bind to the well‐established targeting group D‐biotin [13]. To enhance cellular uptake and biocompatibility, 1,3‐β‐d‐glucans that are glucopyranose polysaccharides with (1,3) glycosidic linkages and varying degrees of (1,4) branching were employed as carrier molecules [32, 33, 34]. These glucans are commonly found in the cell walls of yeasts and plants such as oats and barley [15]. They have been widely investigated in cancer therapy [16, 17, 18, 19, 20, 21, 22], vaccine development [23, 24, 25, 26], immune activation [27], chemotherapy protection [28], antimicrobial applications [29], infection prevention [30], etc. β‐d‐glucans are well known to self‐assemble from random coils into tubular triple‐helix structures at organic solvent–water interfaces, forming nano‐aggregated supramolecular architectures. These natural polysaccharides contain vicinal 1,2‐ or proximal 1,3‐diol units, which interact readily with boronic acids, making them ideal platforms for molecular recognition.

Fluorescent boronic acid‐based probes have also demonstrated potential in glucose detection and monitoring. By contrast to traditional glucose enzyme‐based sensing, boronic acid probes are more stable under physiological conditions and less prone to degradation, exploiting their reversible diol binding to enable continuous real‐time monitoring without reagent depletion [32, 33, 34]. The tunability of boronic acid chemistry, e.g., use of dual boronic acid groups to improve selectivity for glucose as opposed to fructose or galactose, indicates how accuracy can be improved and addresses common challenges in monosaccharide and polysaccharide sensing [35, 36]. Moreover, their fluorescent optical signalling allows for highly sensitive, potentially non‐invasive visualisation of glucose in living systems, in real time and non‐destructively. Additionally, these probes can be integrated into wearable or implantable devices, making them ideal for next‐generation glucose monitoring technologies [37, 38]. This suite of abilities presents precision diagnostic and management applications that could greatly improve patient outcomes and quality of life for a broad range of medical contexts where glucose levels are dysregulated, such as cancer, ageing, tissue repair and metabolic disorders, most notably diabetes [39].

In this study, a combined laser scanning confocal microscopy and fluorescence lifetime imaging microscopy (FLIM) approach was used to assess the uptake of a range of fluorescent boronic tags in vitro, together with the investigation of fluorescence distribution and lifetime within the cellular microenvironment [11, 45, 46]. FLIM offers a distinct advantage by allowing sub‐cellular, diffraction‐limited mapping of probe interactions in living cells [48], and has been extensively applied in imaging cellular protein interactions [50, 51, 52], conformational changes [53], viscosity [54, 55], temperature [56, 57, 58, 59], pH [60], ion concentrations [61], and oxygen levels [62, 63, 64]. In addition, whole live zebrafish embryos were utilized to evaluate the potential of these probes for in vivo monitoring of saccharides (e.g., glucose) and polysaccharides (e.g., glucan) as models for cellular surface receptors at the organismal level. We expanded on previous work showing detection of exogenous glucose in zebrafish [65] by using an insulin mutant zebrafish strain that has up to 10‐fold higher glucose content than their wildtype siblings [66] to demonstrate the capacity of these probes to detect elevated endogenous levels of glucose.

**FIGURE 1:**
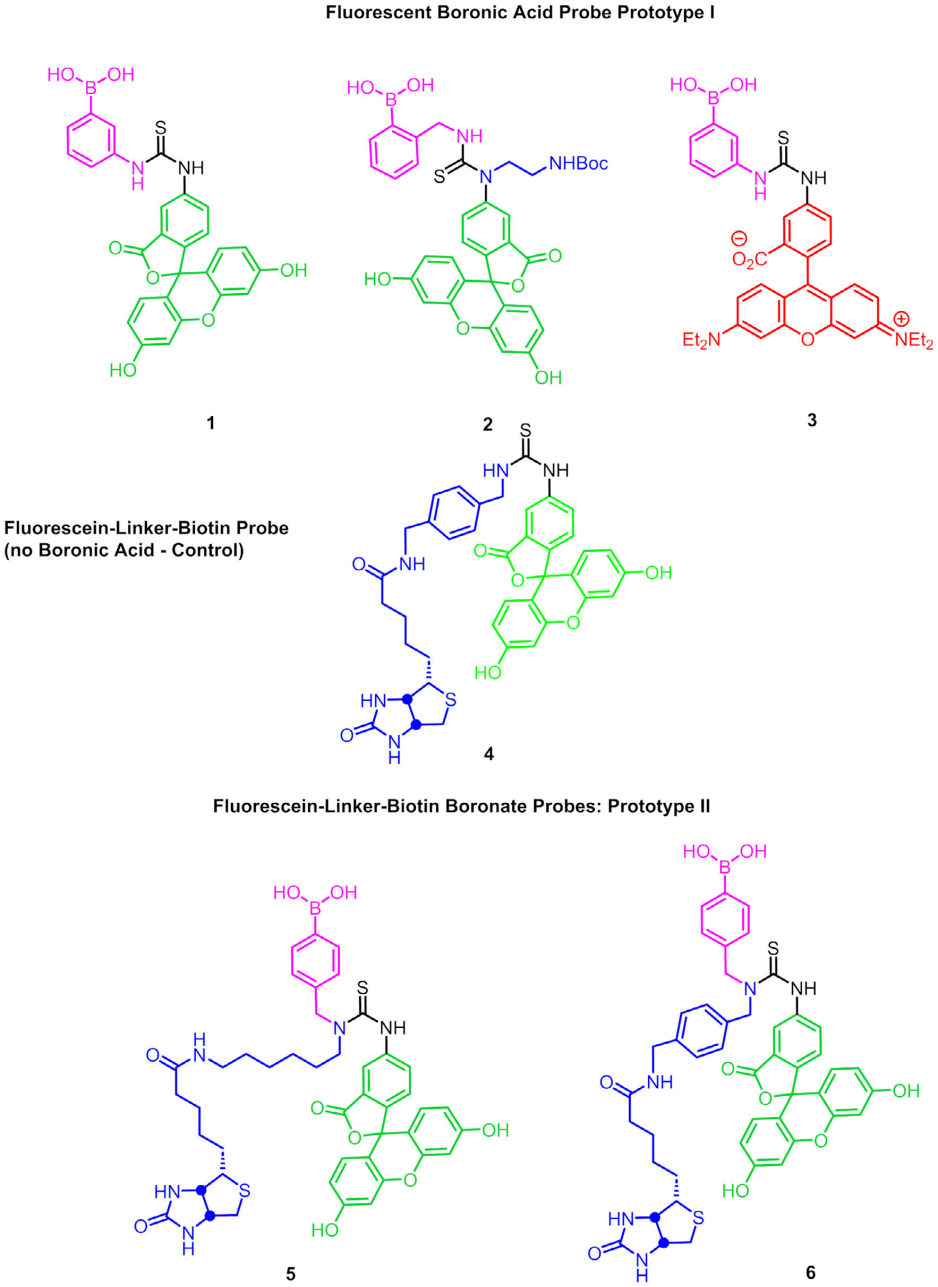
Structural representations of the molecular building blocks: fluorescent boronates (1–3), a biotin‐fluorescein probe without boronic acid (4), and bioorthogonal biotin‐boronic acids (isolated as pinacol‐protected BA), conjugates (5,6), investigated hereby.

Novel, or underexplored, probes denoted compounds 1–6 (Figure 1) incorporating boronic acid and/or boronic ester groups were investigated, whereby the following design elements were incorporated to explore the scope of the imaging tags:

- Compounds 1–3 feature a boronic acid group directly functionalized onto fluorescein isothiocyanate (FITC, compounds 1–2) and rhodamine (3) via the ubiquitous thiourea linkage. These derivatives of xanthene probes generally have excellent fluorescent properties such as high quantum yield of fluorescence (>85%), very good extinction coefficient and brightness, and are non‐toxic to biological systems.
- Compound 4 integrates fluorescein and a biotin functional group via a bio‐orthogonal linker and serves both as an intermediate toward formation of Compounds 5 and 6 and as a control probe to verify the boronic acid's role in cellular uptake.
- Compounds 5 and 6 incorporate a boronic ester group attached via a flexible hexamine spacer (compound 5) or a rigid p‐xylylene‐diamine spacer (compound 6), along with a biotin‐based cell recognition motif. Additionally, using the hydroxyl groups present on the surface of silica, a boronic acid fluorophore was conjugated onto silica surfaces, resulting in the formation of a boronic‐acid labelled nanoparticle derived from compound 6.

The overarching goal of this research was to assess this fluorophore library's ability to bridge the development and application of small molecules and bio‐nanomaterials for cell and tissue labelling. Building on the specific targeting properties of glucans toward cancer cells, a bio‐nanotechnological approach to fluorescent glucan‐based conjugates could enable highly precise, non‐invasive, and cost‐effective early cancer diagnostics.

## 2 Results and Discussion

### 2.1 Synthesis and Spectroscopic Investigations in Solution

**SCHEME 2:**
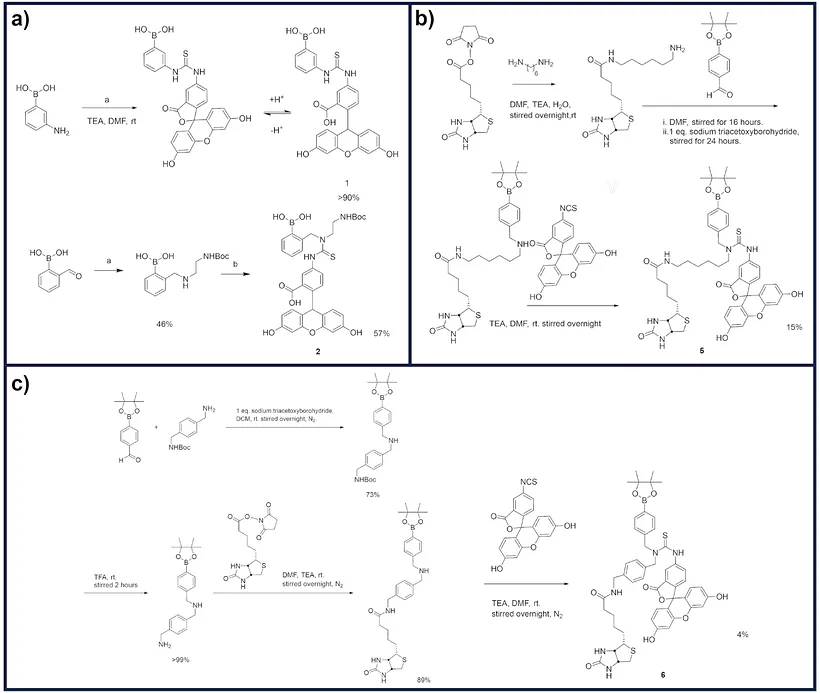
(a) The synthesis of the new fluorescein isothiocyanate (FITC, mixture of 5(6)‐fluorescein isomers) based boronic acid sensor 2: a) 2‐(formylphenyl) boronic acid, DCM, rt.) NaBH_4_, MeOH, rt. (46%), FITC, TEA, DMF, rt. (57%). An adapted synthetic protocol for compound 3 synthesised by an analogous method to compound 1 is given in the Supporting Information. (b) Organic linker synthetic approaches for Compound 5. (c) Organic linker approaches and synthesis of Compound 6, using 4‐formylphenylboronic acid precursor. Adapted synthetic protocols for related, biotin‐conjugated and boronate‐free compound 4 used for control experiments are given in Supporting Information. Additional synthetic and characterisation details are included in Schemes S1.1–S1.7 and Figures S1.1–S1.41. The formation of the new hybrid nanoparticle denoted 6@Fe_3_O_4_@SiO_2_ generated by conjugating boronic acids to ─Si(OH)_2_ functionalities of Fe_3_O_4_@SiO_2_ is detailed in Supporting Information, including the corresponding nanoscale characterisation of the core‐shell entities (Scheme S2.1 and Figures S2.1–S2.11).

Compounds 1 and 3 were synthesised and characterised spectroscopically following adapted methods from the literature, as detailed in Scheme 2 and in Supporting Information (Schemes S1.1–S1.7 and Figures S1.1.–S1.48), starting from commercially available FITC and Rhodamine B (mixtures of isomers), respectively. The boronic acid group was directly functionalised with FITC, a widely used green fluorophore in biomolecular labelling applications. To broaden the scope of fluorescein‐based boronic acid sensors, a new fluorescein isothiocyanate boronic acid derivative containing an additional terminal amino linker, denoted Compound 2, was synthesised. This involved the preparation of a novel boronic acid building block, designed to deliver a fluorescein‐boronic acid conjugate featuring a hydrophobic linker and the extended amino functionality. A new boronic acid building block was synthesised for the FITC‐boronic acid with an extra terminal amino linker following the general reactions shown in Scheme 2a. All related conjugates with structural representations shown in Figure 1 were synthesised following the general synthetic routes shown in Scheme 2.

Simple synthetic modifications to include organic linkers were expected to render the compounds of interest significantly less hydrophilic than the fluorescein starting material. The synthesis involved the reductive amination of mono Boc‐ethylenediamine and 2‐formylphenylboronic acid, yielding a novel boronic acid building block (Scheme 2a). The isothiocyanate group in the fluorescein fluorophore enabled the successful conjugation with the boronic acid building block to obtain compound 2 in 57% yield (Scheme 2). Since fluorescein‐functionalized compounds exhibit noticeable photobleaching under UV–vis light, [66] a rhodamine‐based fluorophore was also explored as an alternative tag and incorporated as a design element in compound 3, which was synthesised following an analogous method to that of compound 1 (Scheme 2a). Additionally, multifunctional compounds 4, 5, and 6 were synthesized following adapted protocols described in Scheme 2b,c and in the Supporting Information (Schemes S1.1–S1.7 and Figures S1.1–S1.41).

All compounds were fully characterized using spectroscopic techniques (^1^H and ^13^C{^1^H} NMR, 2P fluorescence spectroscopy, solid‐state IR spectroscopy and HRMS‐ESI, and reversed‐phase C18 HPLC (Figures S1.1–S1.49). Notably, these compounds share the fluorescein fluorophore, but feature extended organic linkers and a biotin group. Compound 6 was designed to be structurally related to compound 5, with the key difference being the substitution of a flexible hexamethylene diamine with the more rigid xylylene diamine in the linker. Meanwhile, compound 4 incorporated the functionalized xylylene diamine linker and biotin groups, but no boronic acid or pinacol‐protected boronic acid. The rigidity introduced by the linker modifications in compounds 4 and 6 was expected to reduce self‐assembly, which can introduce dipoles [68] and potentially lead to fluorescence quenching effects [69]. We also found that this linker facilitated the synthesis and purification. These fluorescein‐based boronic acids were evaluated for their binding ability to β‐d‐glucans (from barley, Aldrich, MW 20–80 kDa range) using fluorescence titrations. The results showed binding constants above ca. 10^4^ M^−^^1^ in H_2_O:DMSO 1:1 mixtures. Notably, compound 2 also exhibited, as expected, strong binding to d‐glucose, consistent with other well‐studied boronic acid compounds, with a binding constant in the range of 10^8^ M^−^^1^ (Figures S8.1–S8.3). Compounds 1 and 2 acted as the simpler design prototypes, and their fluorescence emission varied with the solvent system. In organic solvents such as methanol or DMSO, compound 1 was colourless with an excitation wavelength of 490 nm and an emission wavelength of 500 nm. Under pH‐controlled buffer conditions (PBS, pH 8.21), the short linker in 2 decreased the basicity of fluorescein isothiocyanate compared to 1, causing a blue shift in emission from 580 to 568 nm. At pH 8.21 phosphate buffer solutions, the emission wavelength red‐shifted from 500 to 580 nm. Compound 2 had an excitation maximum at 490 nm and an emission at 568 nm. Preliminary fluorescence analysis in dilute solutions showed complex behaviour with pH variations, which were further indicated by a fluorescence intensity change upon saccharide addition.

The fluorescence analysis of 2 (λex 490 nm, λem 568 nm) demonstrated an initial reduction in emission at lower concentration regimens, followed by a significant fluorescence intensity increase upon saccharide addition, with saturation occurring at 1.2 × 10^−^^3^ M glucose concentration (pH 8.21) and a range of dynamic equilibria being present. In the presence of β‐d‐glucan, fluorescence titration experiments in water:DMSO (where pH was adjusted to 8.21 with PBS) carried out for compound 5 revealed a binding constant exceeding 10^4^ M^−^^1^, aligning with earlier findings with 6 and the related coumarin‐boronic acid derivatives. For compounds 4 and 5, avidin binding assays were initially used prior to investigations in living systems, as this naturally occurring tetrameric biotin‐binding protein has a very high affinity for biotinylated structures (dissociation constants approaching 10^−^^15^ mol/L).

**FIGURE 2:**
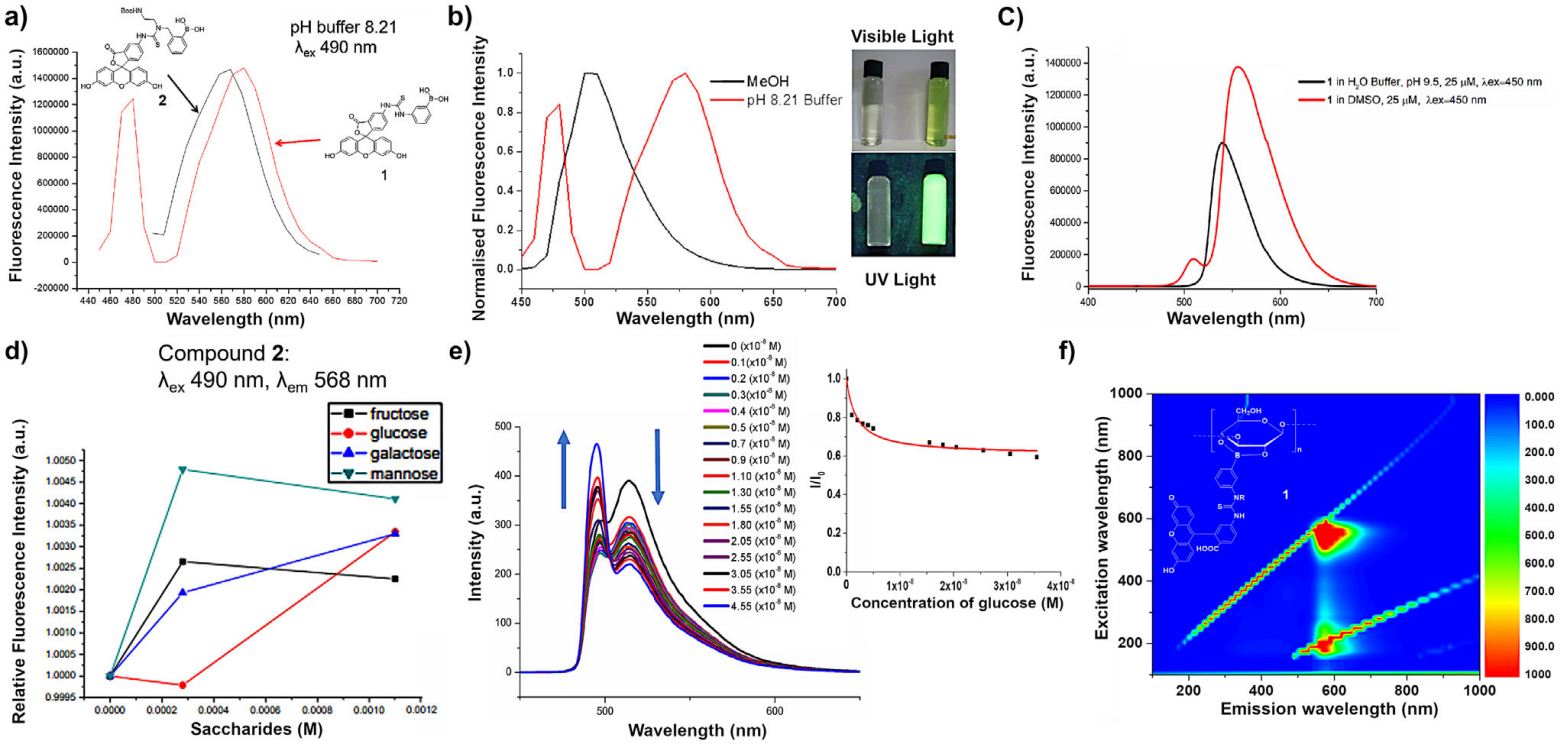
(a) The fluorescence analysis of fluorescein isothiocyanate‐based boronic acid compounds, Compounds 1 and 2 (λ_ex_ 490 nm) at 1 × 10^−6^ M concentration. (b) The fluorescence analysis of fluorescein isothiocyanate‐based boronic acid compound 1: (λ_ex_ 490 nm, λ_em_ = 500 in MeOH and λ_em_ = 580 in pH 8.21 buffer) at 1 × 10^−6^ M concentration; The inset pictures show the colour change of boronic acid sensor 1 from colourless in methanol to yellow in pH 8.21 buffer. Photographs show images under visible light vs. UV_365_ radiation. (c) H_2_O vs. DMSO Solvent effect on the emission of Compound 1. (d) High dilution titration experiment of compound 2 with glucose, showing complex behaviour: binding constant estimated for a first‐order isotherm (λ_ex_ = 450 nm, λ_em_ = 525 nm was 5.35 × 10^8^ M^−1^, R^2^ = 0.95. (e) Selected fluorescence spectra of compounds 1 and 2. (f) Excitation‐emission map for compound 1 in the presence of excess β‐d‐glucan. Further spectroscopic details are given in Figures S6.1–S6.14.

The avidin binding experiments for compounds 4, 5, and 6 were conducted using fluorescence techniques (Figure 2 and Figures S9.1–S9.6). Avidin‐coated beads were employed to assess whether probe 5 could selectively bind to cells via either the biotinylated site or the boronic acid site, and to evaluate whether they retained their fluorescence intensity maxima. As expected, for compound 4, a meaningful binding constant could not be determined in solution phase with respect to glucose or β‐d‐glucans as substrates due to the absence of boronate groups; however, compound 4 still displayed avidin‐binding properties due to the presence of D‐biotin functionality. (also see Figures S.8.1–S8.3, S9.1–S9.5, and S10.1–S10.2). Encouraged by the fluorescence titration results, fluorescein‐based compounds 1, 2, 5, and 6 were treated with β‐d‐glucan in DMSO:H_2_O (1:1 v/v), following a previously reported protocol, yielding new glucan hybrid conjugates denoted as compound@glucan. This protocol was similarly adapted for the formation of the rhodamine–boronic acid hybrid with β‐d‐glucan, referred to as compound 3@glucan.

**FIGURE 3:**
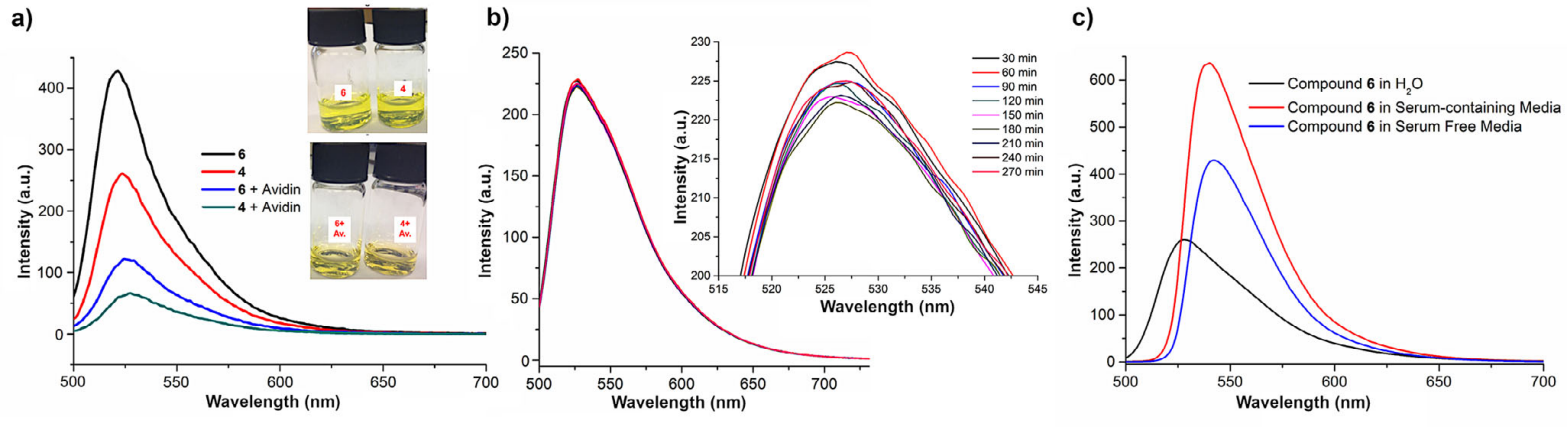
(a) The fluorescence analysis of fluorescein isothiocyanate Compounds 4 and 6 (λ_ex_ 488 nm) at 1 × 10^−6^ M concentration (inset: avidin binding in aqueous solutions for compounds 4 and 6); (b,c) kinetic stability of compound 6 in aqueous media as a pre‐requisite for biological assays.

**FIGURE 4:**
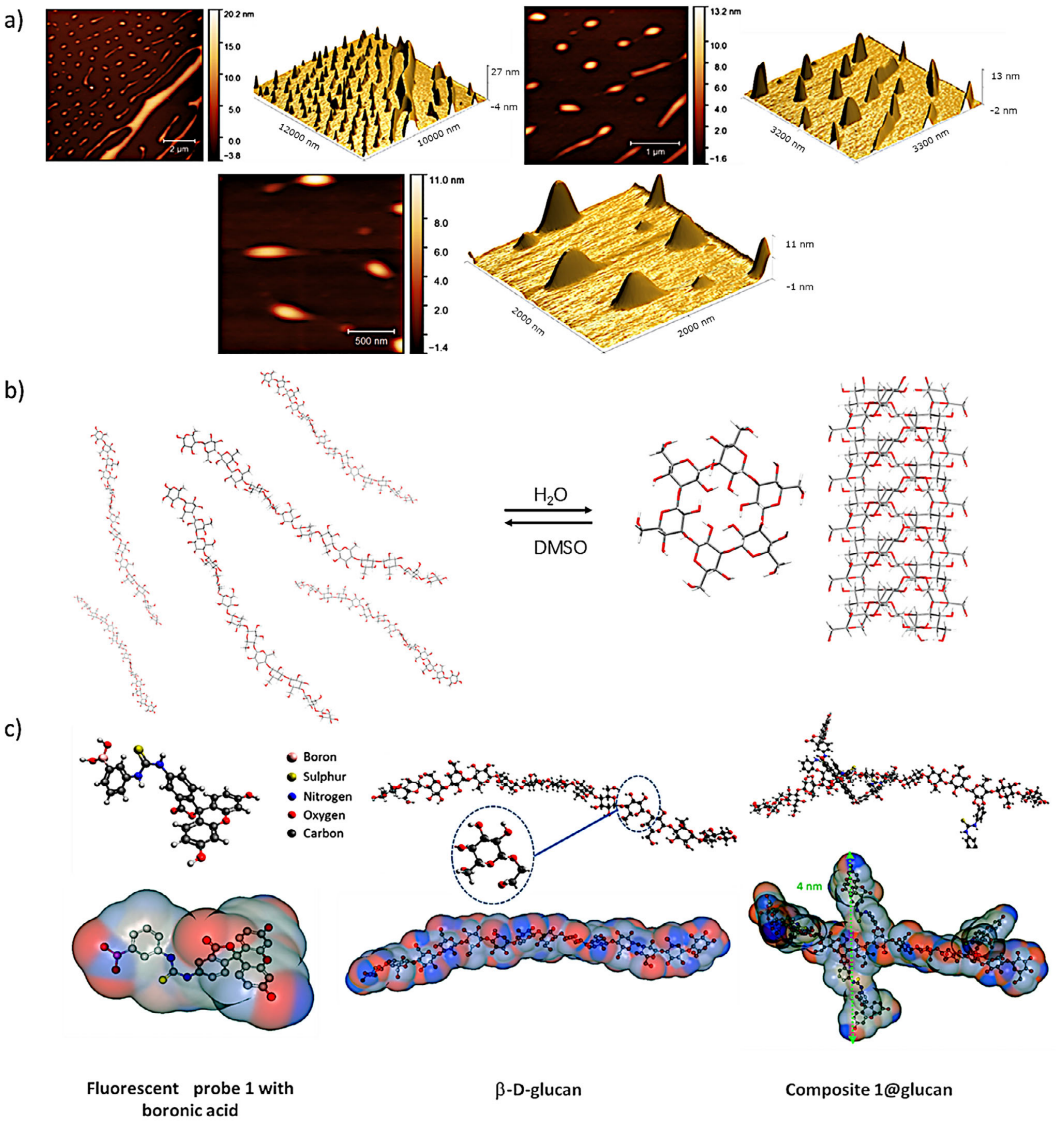
(a) Tapping mode AFM images of β‐d‐glucan spin‐coated (3000 rpm) onto a mica substrate and corresponding 3D tapping mode images. (b) Representation of the aggregation of β‐d‐glucan strands at the interface between H_2_O and DMSO from random strands to organised coils; (c) MM+ optimized structures of fluorescence probe 1, β‐d‐glucan, and composite 1@glucan (red denotes areas of relatively high electron density and blue denotes electron‐deficient areas), with the schematic representations of β‐d‐glucan self‐aggregated in water. The biopolymer utilised hereby (extracted from barley, *Hordeum Vulgare*, Aldrich, Mw ca. 20–60 kDa) comprises d ‐glucose units and displays mainly β‐1,3 and random β‐1,4 backbone linkages. Additional details are given in Figures S12.1–S12.3.

In parallel, computational modelling (MM^+^) was employed to evaluate the spatial conformation and electrostatic potential distribution of the glucan composites (Figure 4 and Figures S12.2–S12.3). Initial structural optimisations were performed using the GAFF Force Field in Avogadro (v 1.2.0). Electrostatic surface potentials were subsequently calculated in MarvinSpace (v 17.21.0), ChemAxon. Figure 4 shows optimised structures of the fluorescein‐boronic acid probe, β‐d‐glucan, and their composite 1@glucan as a representative example. The size of the aggregated composites was estimated to be ∼4–5 nm in the direction perpendicular to the polymer growth axis, consistent with AFM observations and within the order of magnitude suggested by DOSY evaluations of beta‐D‐glucan in D_2_O (Figure S12.1). Additionally, the MM+ analysis indicates that the fluorophore conjugation through boronic acid‐diol interaction does not disrupt the triple‐helical structure of the native glucan. To investigate the aggregated nature of glucan, atomic force microscopy in tapping mode (TM‐AFM) was employed, offering early insights into the likely dimensions of the resulting hybrids. Samples were prepared from aqueous solutions (∼1 mg/mL) and deposited onto mica substrates by spin‐coating at 3000 rpm. AFM imaging revealed supramolecular aggregation of the thin‐film biopolymer. As shown in Figure 3, the aggregates exhibited dimensions of 10–25 nm in height and width, and ∼500 nm in length, likely due to glucan fibre adhesion to the mica surface. These nanometric aggregates are promising for further functionalisation with fluorophores. DOSY experiments indicated that in solution such aggregates of β‐d‐glucan are in the region of 5 nm. Combined with theoretical predictions, these findings corroborate the complex fluorescence titration behaviour observed, characteristic of multiple equilibria in aqueous mixtures, pH‐responsive behaviour of the fluorescein tag, and aggregation effects at higher concentrations. To account for this complex behaviour, we ensured that all in vitro and in vivo experiments were performed under physiological conditions (ca. pH = 7.4), where the behaviour of xanthate‐based dyes is well understood.

CryoSEM imaging supported the hypothesis that β‐D‐glucan plays an active role in modulating the structural organization of the composite, promoting the formation of a heterogeneous, potentially self‐assembled matrix and the stabilization of compound 5. Analysis of the 5@β‐D‐glucan composite prepared in DMSO (1 mg mL^−^^1^) revealed a continuous yet heterogeneous matrix, consistent with the glucan‐mediated assemblies or domains observed by AFM. Such morphology is characteristic of polysaccharide‐driven organization, arising from intermolecular hydrogen bonding and solvent‐mediated interactions. Higher‐magnification images (Figure S9.6) revealed fine textural features embedded within the matrix, which may correspond to localized enrichment of compound 5 within the β‐D‐glucan framework. This observation is consistent with the glucan acting as a structuring scaffold, stabilizing the guest compound through non‐covalent interactions, including hydrogen bonding, hydrophobic effects, and steric confinement. In addition, cryo‐SEM analysis of the 6@Fe_3_O_4_@SiO_2_ system dispersed in Milli‐Q water (Figure S2.10) revealed particle morphologies and surface features consistent with those observed by HR‐TEM for both the Fe_3_O_4_@SiO_2_ scaffold and the corresponding 6@Fe_3_O_4_@SiO_2_ composite, further supporting the structural integrity of the nanocomposite following probe incorporation.

### 2.2 Time Correlated Single Photon Counting (TCSPC) Experiments

Time‐correlated single photon counting (TCSPC), a highly sensitive technique for measuring fluorescence decays on the picosecond timescale and beyond, was employed here to investigate variations in fluorescence lifetime resulting from changes in molecular conformation or solvent environment. Fluorescence lifetime is an intrinsic property of the fluorophore and is largely independent of concentration; however, it can be influenced by molecular aggregation. As such, lifetime measurements serve as a valuable tool for evaluating the sensitivity of fluorescent probes to subtle environmental changes.

**FIGURE 5:**
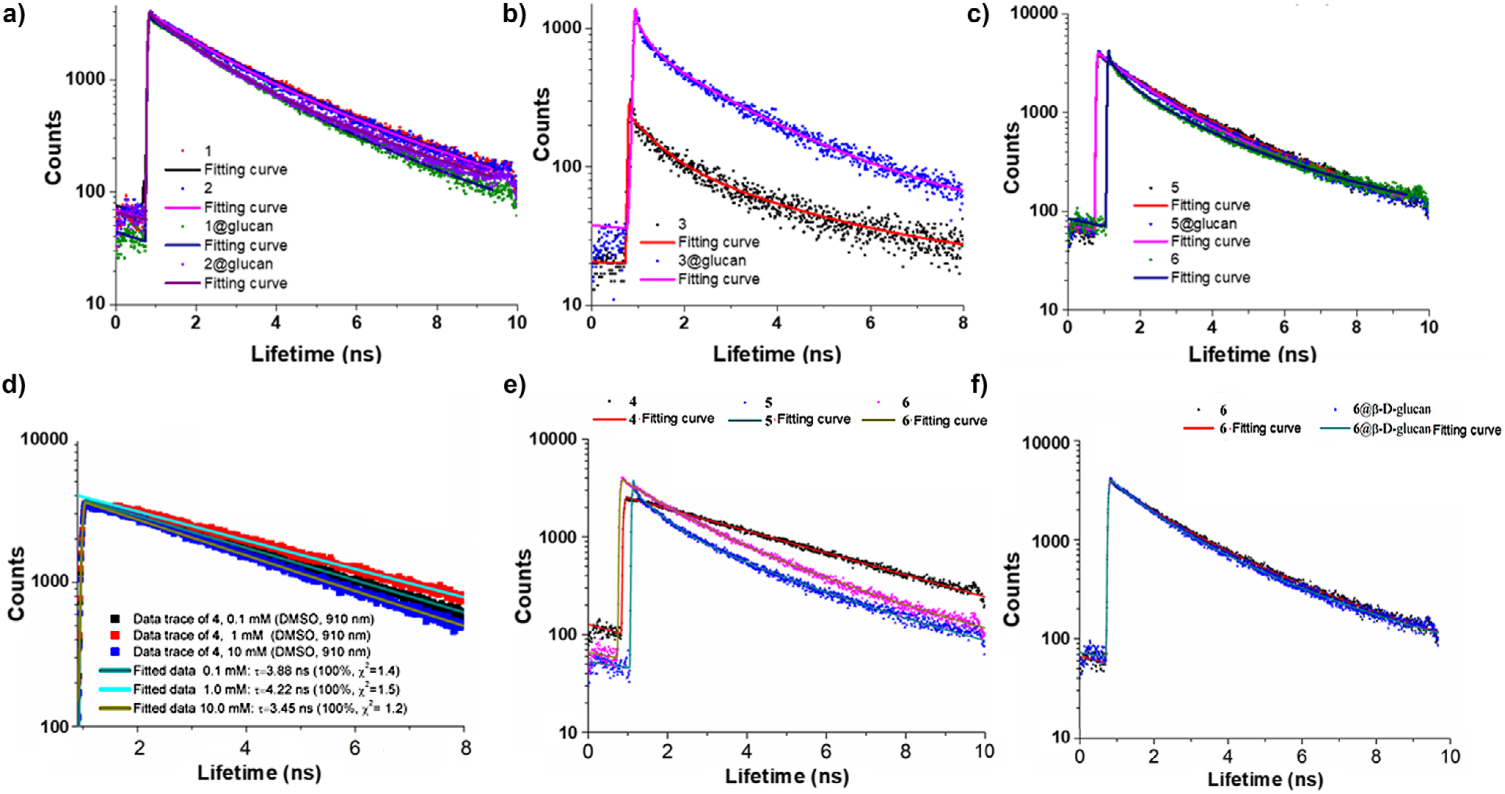
2‐Photon TCSPC Fluorescence lifetime decay curves of compounds 1‐ 6 recorded in the absence and presence of β‐d‐glucan, λ_ex_ = 910 nm, in 0.1 mM concentration DMSO, unless otherwise specified.

Experiments were conducted in commercial, aqueous DMSO to provide a controlled baseline. TCSPC was used to compare the fluorescence lifetimes of compounds and their corresponding glucan‐bound hybrids (denoted compound@glucan) in DMSO, and then to contrast these with the lifetimes observed in cellular environments. This allowed investigation of the effects of binding interactions and microenvironmental changes on the photophysical properties of these probes. These fluorescent probes with 2‐photon emission profiles indicative of the expected xanthate fluorophores (Figures S6.1–S6.14) exhibited biexponential lifetime decay, except for compound 4, which showed a single‐component lifetime of ca. 3.5 ns comparable to that of the fluorescein standard measured under the same conditions (4.1 ns), as shown in Figure 5 and Table 1. For example, compound 1 displayed a short lifetime component (τ_1_) of 1.67 ns (48.48%) and a long‐lived lifetime component (τ_2_) of 3.01 ns (51.52%), as shown in Table 1. Similarly, compound 2 exhibited τ_1_ = 1.23 ns (52.84%) and τ_2_ = 3.28 ns (47.16%). Upon functionalisation with β‐d‐glucan, both τ_1_ and τ_2_ values estimated across the range of compounds analysed showed a slight decrease, suggesting that binding to glucan through the boronic acid units induces a subtle quenching effect. Specifically, for compound 1@glucan, the lifetimes changed to τ_1_ = 1.32 ns (51.87%) and τ_2_ = 2.65 ns (48.13%), while for compound 2@glucan, the corresponding lifetime values also shifted downward subtly to τ_1_ = 1.02 ns (53.58%) and τ_2_ = 2.93 ns (46.42%). The fluorescence lifetime of compound 3 exhibited biexponential decay, in a free, as well as in its glucan‐bound hybrid form (denoted 3@glucan), however with only very subtle differences in the relative amplitudes obtained from decay profiles. These changes in the overall average lifetime values τm were also expected upon binding to glucan in solution. Notably, free fluorescein standards typically displayed a single exponential decay with a lifetime of approximately 4.1 ns, while rhodamine exhibited a characteristic decay of 1.8 ns, and these values were confirmed in control experiments under the 2‐photon excitation employed herein. The presence of multicomponent lifetime profiles in compounds 1–6 is an early indication of subtle alterations in the fluorophore's local environment, which may be extrapolated to their behaviour within cellular systems. Additionally, both compounds 5 and 6 exhibited biexponential decay profiles as shown in Table 1.

**TABLE 1:** Fluorescence lifetime of fluorescence compounds 1–6 and corresponding glucan hybrids measured by 2P TCSPC in DMSO solutions at 100 µM of compound. In the case of the hybrids analysed, compounds 1–6 (100 µM) were formulated in the presence of 1 mg/ml beta‐D‐glucan solutions containing the 1:1 DMSO:PBS matrix to ensure solubility. λ_ex_ = multiphoton at 910 nm, laser power 2–8 mW, Fluorescein standard (pH 11, 3 µM in 0.1 NaOH H_2_O solution): τ (ns) = 4.13 (100%, ch^2 = ^1.09); Rhodamine B standard (5 µM in MeOH): 1.76 ns (100%, ch2 = 1.02). τ_m_ = mean lifetime.

	τ_1_ (ns)	a_1_ (%)	τ_2_ (ns)	a_2_ (%)	τ_m_ (ns)	Chi^2^
1	1.62	48.48	3.01	51.52	2.34	1.11
1@glucan	1.32	51.87	2.65	48.13	1.96	1.02
2	1.23	52.84	3.28	47.16	2.22	1.18
2@glucan	1.02	53.58	2.93	46.42	1.91	1.14
3	0.28	46.03	2.10	53.97	1.26	1.05
3@glucan	0.23	48.29	2.14	51.71	1.22	1.20
4	3.48	100			3.48	1.27
5	1.15	48.43	2.80	51.57	1.99	1.10
5@glucan	1.03	53.68	2.69	46.32	1.79	1.16
6	0.81	74.89	2.72	25.11	1.29	1.11
6@glucan	0.38	44.53	2.11	55.47	1.34	1.34
6@Fe_3_O_4_@SiO_2_	0.29	50.03	2.24	49.97	1.27	1.49

The differences in lifetime values and their relative amplitudes were noticeable between 5 and 6, however resulting in highly comparable overall average lifetimes for these biotinylated and simultaneously boronated derivatives. The fluorescence lifetime of the biopolymer hybrid 5@glucan exhibited a biexponential decay with τ_1_ = 1.03 ns (53.68%) and τ_2_ = 2.69 ns (46.32%). Although these individual lifetimes are somewhat lower than the τ1 = 1.15 ns (48.43%) and τ2 = 2.80 ns (46.32%) values estimated for the parent compound 5, the shift in the distribution of component amplitudes led to the average lifetime of 5@glucan to 1.79 ns, only slightly reduced from the average lifetime of 1.99 in the free compound 5 at this concentration. Similarly, the lifetime parameters of compound 6, with τ_1_ = 0.81 ns (74.89%) and 2.72 ns (25.11%) and those corresponding to the respective glucan and Fe_3_O_4_@SiO_2_ hybrids, were fitted as multiexponential lifetime parameters with values indicative of more complex behaviours in the dispersed phase, as shown in Table 1. The difficulties in fitting for these composites were attributed to aggregation of these boronic acids, where the increased rigidity of the linker present in 6 facilitated the aromatic stacking behaviour.

### 2.3 Multiphoton FLIM and Correlated Confocal Microscopy Assays in Living Cells

Cytotoxicity of the boronic acid derivatives was first evaluated using MTT assays, indicating that they are generally well tolerated at the concentrations used, ranging between 10 and 100 µM over the cellular imaging conditions of up to 1 h incubation primarily used in the in vitro imaging investigations (Figures S3.1–S3.9). To assess potential effects associated with prolonged exposure, cytotoxicity and phototoxicity of the xanthene‐based boronate probes were further examined using 24 h assays. To further assess their suitability for live‐cell imaging. While compounds 2, 3, 5, and 6 reduced PC‐3 cell viability to approximately 70%–80% after 24 h exposure at 100 µM, these effects were observed under incubation conditions that substantially exceed the duration of the imaging experiments. Cellular uptake of fluorescein‐ and rhodamine‐tagged isothiocyanate boronic acid derivatives, as well as biotin‐tagged hybrids, was successfully demonstrated using a suite of assays, highlighting the facilitating role of the boronic acid tag in conjunction with hydrophobic organic linkers. Since these probes incorporate xanthene‐based chromophores, fluorescein (in compounds 1,2, 4–6) or rhodamine (compound 3) as fluorescent tags, both of which are known for excellent emissive characteristics, including high quantum yields (>85%), strong extinction coefficients, high brightness, and relatively low toxicity in biological systems, were expected. During standard confocal and 2P FLIM imaging protocols, cells were exposed to the probes for relatively short periods (typically 15–20 min and ≤1 h) to minimise photobleaching. To evaluate potential imaging‐induced damage, additional MTT assays were performed under 405 nm irradiation conditions and showed no significant decrease in cell viability during 1 h observation periods. Furthermore, confocal time‐lapse experiments conducted over 1 h revealed no evidence of progressive membrane blebbing, cell detachment, loss of membrane integrity, or other morphological changes commonly associated with acute phototoxicity. Cell morphology remained stable throughout the imaging period, supporting the conclusion that the probes are compatible with short‐term live‐cell imaging despite the moderate reduction in viability observed following prolonged 24 h exposure at the highest concentration tested. Representative confocal time‐lapse datasets as well as 15 min and 1 h incubation experiments by correlated confocal and 2P FLIM experiments (910 nm) demonstrating the preservation of cellular morphology during imaging are described below and have been included in the (Figures S4.1–S4.26 and S5.1–S5.12 and Timelapses .mov micrographs, included in Supporting Information).

**FIGURE 6:**
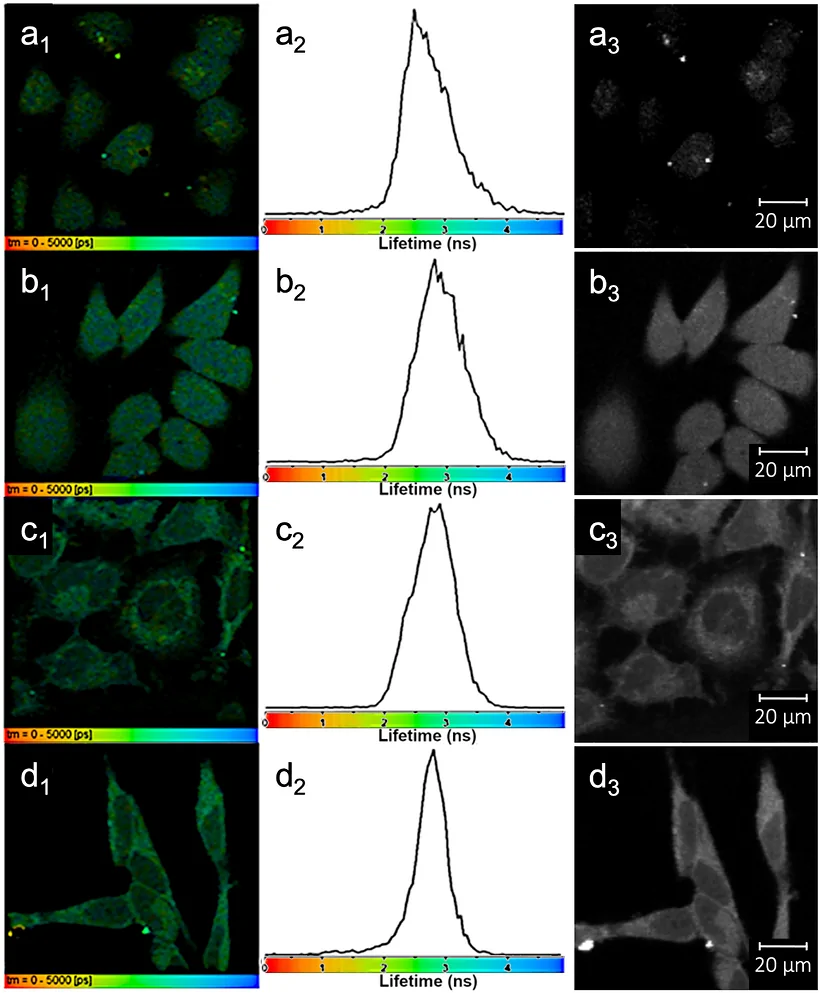
Multiphoton fluorescence‐lifetime imaging microscopy (FLIM) in cellular environments. FLIM images of HeLa cells treated with 100 µM of compounds in 1% DMSO; cells were incubated at 37°C or 4°C, where (a) 1, 37°C, (b) 1@glucan, 37°C, (c) 2, 37°C, (d) 2@glucan, 37°C. (a_1_–d_1_) are the fluorescence lifetime maps of compounds in HeLa cells, λ_ex_ = multiphoton at 910 nm. (a_2_–d_2_) are the distribution curves of the τ_m,_ and the colour represents the lifetime of compounds in (a_1_‐d_1_). The micrographs denoted a_3_‐d_3_ are the corresponding correlated intensity images from multiphoton confocal microscopy, λ_ex_ = 910 nm. Lifetime distribution and Scale bar: 0–5 ns and 20 µm.

**FIGURE 7:**
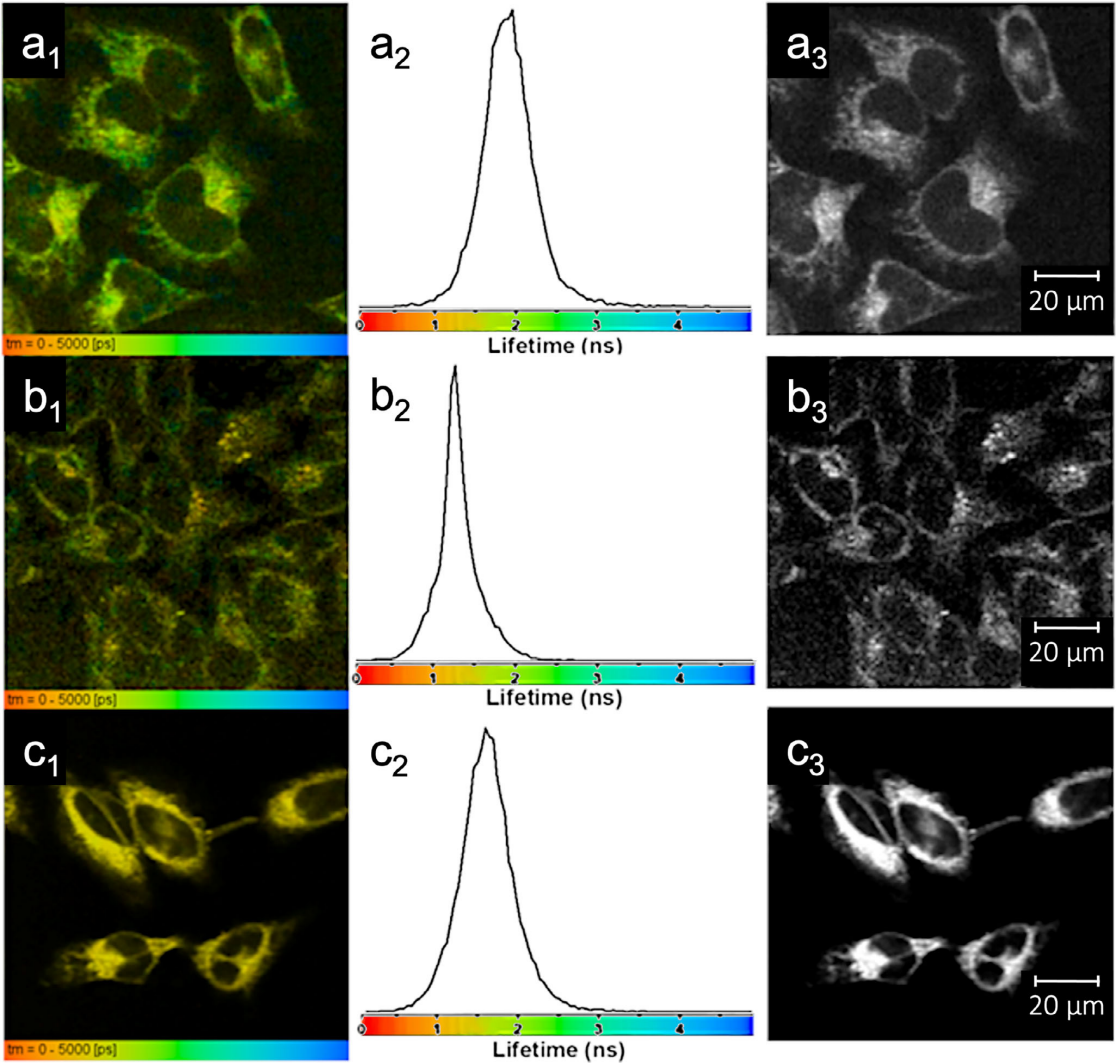
2P‐FLIM images of HeLa cells treated with 100 µM of compounds in 0.5% DMSO, where (a) 3, 37°C, (b) 3, 4°C, (c) 3@glucan, 37°C. (a_1_–c_1_) is the fluorescence lifetime map of compounds in HeLa cells λ_ex_ = multiphoton at 910 nm. (a_2_–c_2_) are the distribution curve of the τ_m_ and the colour represents the lifetime of compounds in (a_1_–c_1_). (a_3_–c_3_) are the corresponding correlated intensity images from multiphoton confocal microscopy, λ_ex_ = 910 nm. Scale bar: 0–5 ns and 20 µm, λ_ex_ = 910 nm. Scale bar: 0–5 ns and 20 µm. Alternative micrographs including for the uptake of 3@glucan in PC‐3 cells are given in the Supporting Information.

**FIGURE 8:**
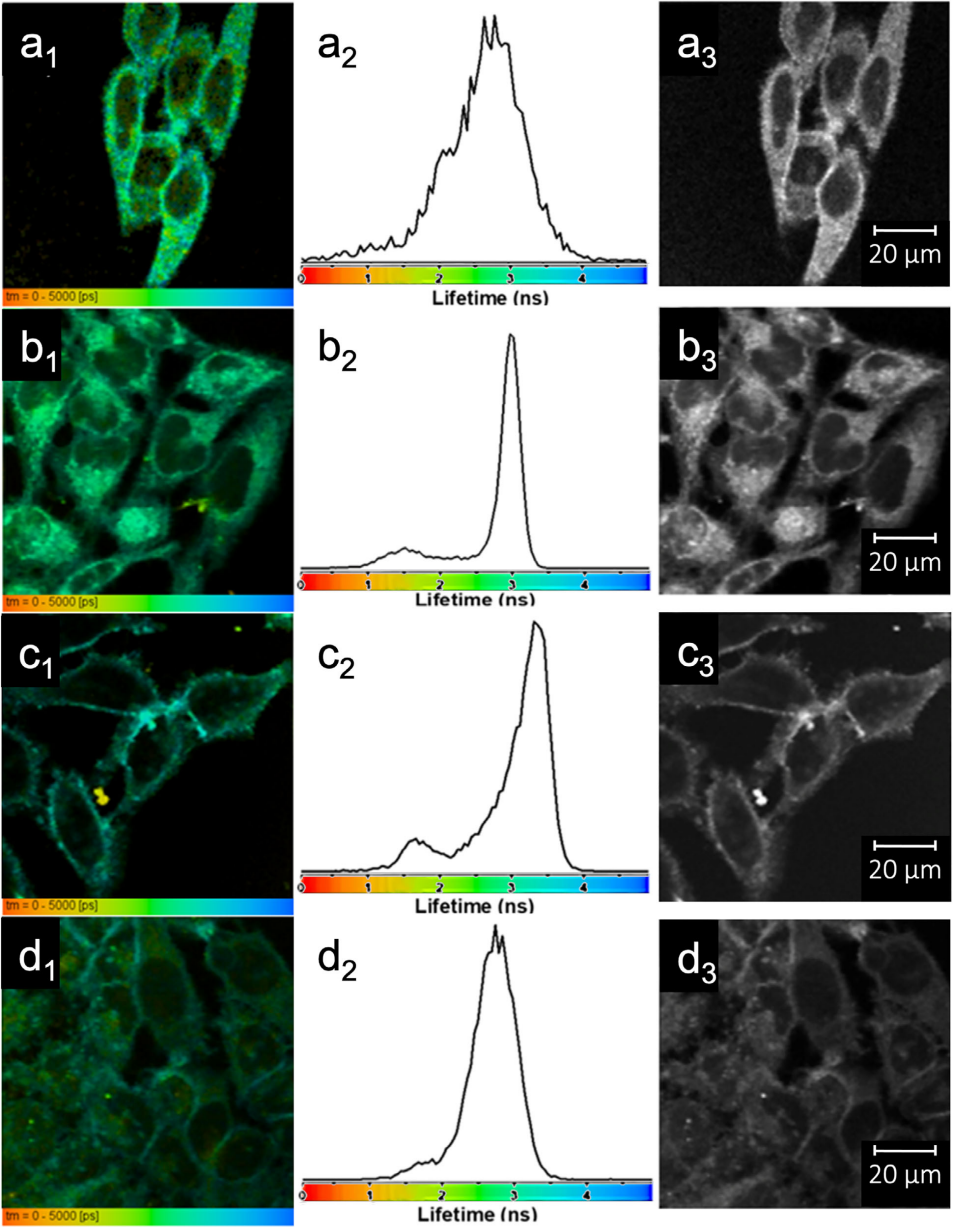
2P FLIM images of HeLa cells treated with compounds in 1% or 2% DMSO, cells were incubated at 37°C or 4°C, where (a) 5, 1% DMSO, 37°C, 15 min, (b) 5, 1% DMSO, 37°C, 60 min, (c) 5, 1% DMSO, 4°C, 15 min, (d) 5@glucan, 37°C, 15 min. (a_1_–d_1_) is the fluorescence lifetime map of compounds in HeLa cells, λ_ex_ = 910 nm. (a_2_–d_2_) are the distribution curves of the τ_m_, and the rainbow colour represents the lifetime distribution of compounds in a_1_‐d_1_. a_3_‐d_3_ are the corresponding correlated intensity images from multiphoton confocal microscopy, λ_ex_ = 910 nm. Scale bar: 0–5 ns and 20 µm. Alternative micrographs obtained under λ_ex_ 910 or 810 nm excitation, and for the uptake of 5 and 5@glucan in HeLa and in MCF‐7 cells are given in Supporting Information (i.e. confocal fluorescence micrographs: Figures S4.1–S4.17 and S5.1.–S5.13, 2 photon fluorescence emission data in DMSO Figures S6.1–S6.14, correlated 2P FLIM micrographs including fitted TCSPC lifetime point decays in cells: Figures S7.1–S7.47).

Time‐correlated single photon counting (TCSPC) and two photon FLIM techniques enabled investigation of the probes’ cellular environments, their interactions with β‐d‐glucan, and the influence of surrounding cellular components. In addition, colocalization experiments were conducted to explore the potential of these probes for cellular penetration as well as membrane binding and organelle‐specific targeting (Figures S7.1–S7.47). Importantly, all in vitro (and subsequently, in vivo work, vide infra) was performed at pH 7.4 to minimise isomerisation effects due to the pH responsiveness of xanthate dyes. To explore their potential as cell‐penetrating multimodal imaging tags, boronic acid derivatives 1–6 were investigated using confocal fluorescence microscopy in conjunction with multiphoton fluorescence lifetime imaging microscopy (MP‐FLIM), in correlated modes, using the same field of view. Cellular uptake and intracellular distribution were assessed by laser‐scanning confocal fluorescence microscopy under both single photon and two photon excitation conditions at 910 nm, taking advantage of near‐infrared excitation for enhanced tissue penetration and reduced photodamage (Figures 6, 7, 8 and Table 2). An initial comparison of imaging conditions revealed that prolonged irradiation at 810 nm resulted in some detectable cellular damage, not observable under 910 nm excitation. Specifically, for compounds 5 and 6, incubation extending beyond 1 h followed by excitation at 810 nm showed some evidence of membrane blebbing and localized membrane damage (Figures S7.40–S7.42). These relatively harsh excitation conditions were applied in this subset of analysis only, because the boronate derivatives exhibited rather weak emission under 810 nm excitation. In addition, significant cellular autofluorescence was observed at this wavelength, which could not be readily separated from the probe signal. Consequently, imaging at 405 (singlephoton excitation) or 810 nm (two‐photon excitation) was minimised for this class of compounds, and subsequent imaging studies were performed under conditions that reduced phototoxicity while maintaining sufficient signal intensity for fluorescence and FLIM analysis, at the available laser lines of 488 or 910 nm excitation.

**TABLE 2:** Fluorescence lifetime decay of fluorescence in boronic acid probes studied and of corresponding glucan hybrids measured by 2P FLIM in cellular environment of living HeLa cells under different temperatures and incubation times. Values correspond to the micrographs given in Figures 6, 7, 8, 9 and represent calculated 2P FLIM data from the in vitro fluorescence lifetime distribution histograms of HeLa cells treated with 100 µM of 1–6 and corresponding beta‐D‐glucan (1 mg/mL) formulations, incubation for 15 or 60 min using multiphoton excitation at 910 nm. Data presented as peak ± error (ns): The full width at half maximum (FWHM), calculated from the lifetime distribution curve within the focal area was used to assess the fluorescence lifetime distribution error. Additional 2P and 1P FLIM micrographs are given in Figures S7.1–S7.47 and Table S1).

	Peak ± FWHM (ns)		Peak ± FWHM (ns)
1, 37°C, 15 min	2.44 ± 0.41	2, 37°C, 15 min	2.85 ± 0.41
1@glucan, 37°C, 15 min	2.80 ± 0.43	2@glucan, 37°C, 15 min	2.80 ± 0.26
1@glucan, 4°C, 15 min	2.40 ± 0.41	2@glucan, 4°C, 15 min	2.66 ± 0.45
3, 37°C, 15 min	1.94 ± 0.32		
3, 4°C, 15 min	1.54 ± 0.33	4, 37°C, 60 min	1.33 ± 0.78
3@glucan, 37°C 15 min	1.24 ± 0.14		
5, 37°C, 15 min	2.81 ± 0.50	5@glucan, 37°C, 15 min	2.75 ± 0.34
5, 37°C, 60 min	3.00 ± 0.16	5@glucan, 37°C, 60 min	2.37 ± 0.28
5, 4°C, 15 min	3.35 ± 0.28	5@glucan, 4°C, 15 min	1.65 ± 0.14
5, 4°C, 60 min	3.40 ± 0.17	5@glucan, 4°C, 60 min	3.34 ± 0.24
6, 37°C, 15 min	3.52 ± 0.30	6@glucan, 37°C, 15 min	3.30 ± 0.25
6, 4°C, 15 min	3.31±0.25	6@glucan, 4°C, 15 min	3.15±0.24

We deliberately employed correlated fluorescence lifetime‐based imaging assays, rather than relying solely on emission intensity from conventional confocal microscopy, to characterise intracellular environments. Fluorescence lifetime imaging microscopy (FLIM), including multiphoton excitation mode (MP‐FLIM), has significantly advanced cellular sensing and diagnostics, especially in biophysical applications. FLIM measures the time a fluorophore remains in its excited state, providing insights into dynamic molecular interactions, membrane potential fluctuations, and metabolic activity within live cells [79]. Moreover, FLIM can distinguish and isolate specific aspects of a single fluorophore's behaviour, such as luminescence quenching by intracellular ions (e.g., Ca^2^^+^, Cl^−^) or oxygen, and detect binding events, molecular distances, or conformational changes through shifts in fluorescence lifetime. This technique is especially powerful for investigating environmental factors such as pH, ion concentration, molecular aggregation, viscosity, and interactions with nanoparticles, due to the extended excited‐state lifetimes often observed in these contexts. Two photon FLIM, which uses less phototoxic NIR light for deep tissue imaging, has driven recent advances with new chemical probes (e.g., endogenous molecules such as tryptophan, serotonin), fluorescent dyes (e.g., GFP‐tagged proteins, fluorescein derivatives), but remains underutilized for small‐molecule fluorophores in synthetic chemical biology. A significant advantage of (MP)‐FLIM, either alone or coupled with laser scanning confocal microscopy, lies in its capacity to characterise boronic acid‐based compounds and their saccharide or polysaccharide complexes within biologically relevant environments. The fluorescence lifetime parameters of both free boronic acid fluorophores and their diol‐based complexes depend on the structural integrity of the probe and its responsiveness to the biomolecular surroundings, as reflected in its lifetime “fingerprint”.

By correlating solution‐phase behaviours with intracellular localisation via TCSPC and MP‐FLIM, these techniques provide unique insight into cellular dynamics, offering significant potential in drug development and disease diagnostics. Extensive in vitro cellular imaging using multiphoton FLIM was a critical step before progressing to in vivo studies, with the most promising candidates identified through MP‐FLIM (Figure 6). For example, the fluorescence lifetime of compounds 1 and 2 increased in the cellular environment at 37°C, even when bound to glucan, indicating a conformational change upon cellular internalisation [80, 81]. However, when the temperature was lowered to 4°C, the FWHH distribution value for the lifetimes of 1@glucan and 2@glucan decreased from 2.80 to 2.40 ns and 2.66 ns, respectively. This reduction was attributed to increased cellular viscosity upon cooling, which influenced the excited‐state lifetimes [82, 83, 84, 85].

Similarly, compound 3 displayed a decrease in lifetime from 1.94 ± 0.32 ns at 37°C to 1.54 ± 0.33 ns at 4°C. Notably, when bound to glucan at 37°C, the fluorescence lifetime of compound 3 dropped further from 1.94 ± 0.32 ns to 1.24 ± 0.14 ns, suggesting that glucan‐induced conformational changes have a more pronounced effect on the probes’ photophysics than temperature variation alone. Most fluorescence emission was localised to the cytoplasm, with no nuclear staining observed (Figure 7a). It was also noticed that as a result of cooling, the viscosity of the cell was increased, resulting in an alteration of fluorescence lifetime [82, 83, 84, 85] and implying that the impact of conformational changes induced by the glucan is much greater than the impact of the temperature changes on the fluorescence lifetime. Control experiments in unfunctionalized fluorescein and rhodamine alone indicated that the former does not cell‐penetrate, and the latter localises in cellular mitochondria.

An overlay of the fluorescence image of compound 3 with MitoTracker Green revealed colocalization (Figure S5.9), suggesting that compound 3 localised within the cytoplasm and effectively stains mitochondria. To enhance biocompatibility while preserving cellular uptake efficiency, a β‐d‐glucan formulation of the probe (3@glucan) was employed in uptake studies (Figure 7). This hybrid showed only a modest increase in fluorescence intensity compared to compound 3 alone (Figure 7b), indicating improved performance. However, when cells were incubated at 4°C, a marked reduction in fluorescence intensity was observed, with comparable cellular morphology (Figure 7c), suggesting that uptake is temperature‐dependent and likely energy‐mediated.

Compound 5 was selected for cellular imaging studies owing to its improved structural design compared to compounds 1 and 2, featuring a longer hydrophobic linker and a biotin targeting moiety. The effect of biotin incorporation on cellular selectivity was assessed based on the hypothesis that biotin functionalization would increase affinity toward cancer cells. As shown in Figure 8, compound 5 readily permeated the cell membrane within 15 min at 37°C, with cells maintaining normal morphology and showing no evidence of membrane disruption. Fluorescence signals were predominantly localized within the cytoplasm, with minor punctate aggregates observed at the cell membrane. A clearly defined cytoplasmic–nuclear boundary indicated that compound 5 did not penetrate the nuclear envelope. Co‐localization with LysoTracker further confirmed the lysosomal localization of compound 5. When formulated with β‐d‐glucan (5@glucan), uptake was reduced in MCF‐7 cells, with mean fluorescence intensity decreasing from 94.50% to 27.69%, likely due to glucan‐induced interference with cellular activity in this cell line [75]. 2P FLIM analysis confirmed cytoplasmic fluorescence distribution and an absence of precipitation on the membrane. Cell morphology remained intact during the observation window employed of up to 2 h, with well‐defined membranes and strong adhesion, suggesting no cytotoxicity from the compound@glucan hybrids. (Figures S4.1–S4.17 and S5.1.–S5.13).

Both compound 5 and corresponding hybrid 5@glucan were evaluated across various cancer cell lines under a range of different conditions. Imaging results, summarised in Figure 8, show that after 15 min of incubation at 37°C, compound 5 predominantly associated with the cell membrane, consistent with efficient membrane interaction and internalisation. After 60 min of incubation at 37°C, most of the fluorescence emission characteristic of compound 5 was localized in the cytoplasm (Figure 8). At reduced temperature (4°C), compound 5 initially accumulated on the cell membrane within 15–20 min and after 60 min, fluorescence characteristic of the presence of the internalised probe gradually spread throughout the cytoplasm (Figure 8 and for additional micrographs in the series, mapped over a range of lifetime domains in the 0–5000 ps interval, see Figures S7.1–S7.47).

In the presence of glucan, the mean fluorescence intensity of compound 5 increased by a minimum of 15%. The 5@glucan hybrid rapidly accumulated in the cytoplasm within 15 min, with uptake completed by 60 min. Both in the absence and presence of glucan, compound 5 exhibited complex uptake behaviour, characterised by initial membrane association followed by cytoplasmic internalisation and eventual localisation to mitochondria and other intracellular organelles over time. These uptake subtleties were further analysed and confirmed via fluorescence lifetime imaging microscopy (2P FLIM, vide infra). Notably, this rapid membrane penetration persisted even at 4°C. Bar chart representation of fluorescence intensity analysis in the field of view indicated that low temperature enhanced short‐term absorption for both compound 5 and 5@glucan but inhibited long‐term fluorescence intensity increases (Supporting Information). Cellular morphology showed minimal damage for compounds 5 and 6 and under prolonged 810 nm irradiation which was assigned to the combined effects of low temperature, rapid uptake, and light exposure [76], however this damaging effect was not observed at 910 nm over 15 min or 1 h incubation assays, nor was it validated by standard MTT assays performed under UVA irradiation (Figure S.3.9) Supporting Information also contains a range of additional confocal fluorescence micrographs: Figures S4.1–S4.26 and Figures S5.1.–S5.13, and the corresponding correlated 2P FLIM images are given in Figures S7.1–S7.9.

**FIGURE 9:**
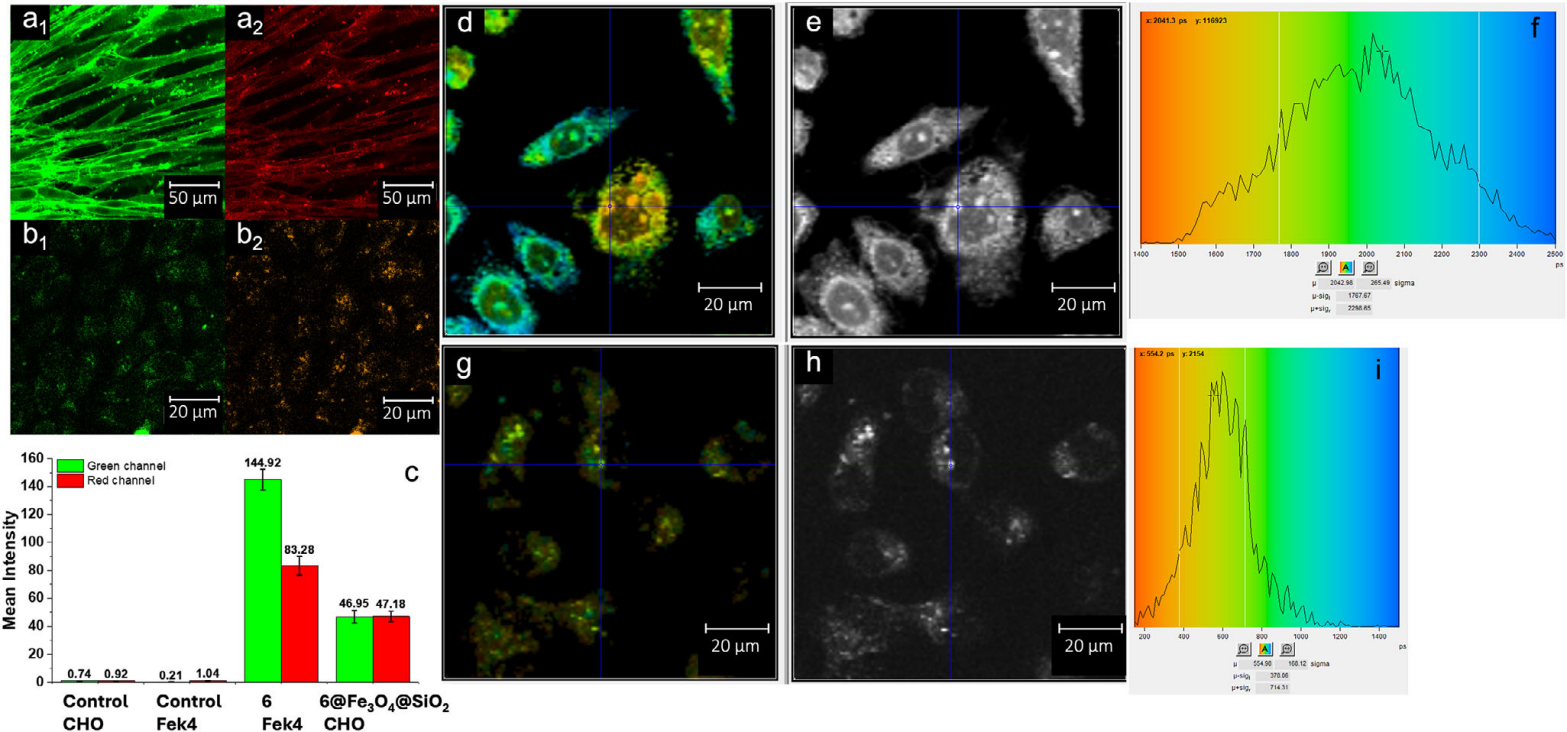
Overview of the distribution of compound 6 and the 6@Fe_3_O_4_@SiO_2_ hybrid in healthy and cancerous cells. Confocal microscopy was used to image uptake in healthy cells, while correlated confocal and two photon fluorescence lifetime imaging microscopy (2P‐FLIM, 910 nm excitation) were applied for cancer cells. (a_1_,a_2_) Uptake of compound 6 in Fek‐4 cells; (b_1_,b_2_) uptake of the nanohybrid 6@Fe_3_O_4_@SiO_2_ in CHO cells; (c) mean intensity estimated in fields of view shown in micrographs (a,b); (d–f) 2P‐FLIM images showing lifetime intensity and distribution of compound 6 in HeLa cells; (g–i) multiphoton FLIM analysis of 6@Fe_3_O_4_@SiO_2_ uptake in HeLa cells. Additional 2P FLIM micrographs for the uptake of 6@Fe_3_O_4_@SiO_2_ in living HeLa and CHO are given in Figures S11.1–S11.7, and compared to the 2P TCSPC lifetime point decay in dispersed phase, Figure S11.8.

Compound 6 features a more rigid organic linker, xylylenediamine, replacing hexamethylenediamine with the aim to modulate solubility and to reduce self‐assembly [68], which may otherwise contribute to quenching effects [69]. Similar to compound 5, compound 6 easily permeated the plasma membrane. Additionally, healthy non‐cancerous FEK‐4 cells readily internalized compound 6, with fluorescence primarily observed on the membrane and to a lesser extent in the cytoplasm. No membrane damage was evident, as suggested by a flat and intact cell profile (Figure 9).

Interestingly, compound 4 does not significantly cell penetrate on the 20 min incubation time and localises in the cellular cytoplasm over more than 1 h timescale (see Supporting Information for confocal fluorescence imaging and 2P FLIM). A clear increase in the fluorescence lifetime of compound 5 was observed when transitioning from DMSO to the cellular environment, both in short‐ and long‐term incubations at 37°C and 4°C. For example, the lifetime of compound 5 increased from 1.72 ns (in DMSO) to 2.81 ± 0.50 ns in cells; for 5@glucan, it increased from 1.88 ns to 2.75 ± 0.34 ns. These findings suggest that compound 5 interacts with intracellular components, such as proteins, leading to measurable changes in fluorescence lifetime. The glucan conjugation also appeared to modify uptake routes and subcellular localization, as evidenced by lifetime variations under different conditions. A comparison of the lifetime distributions across a range of environments and conditions in this class of compounds is given in Figures S7.1–S7.47.

**FIGURE 10:**
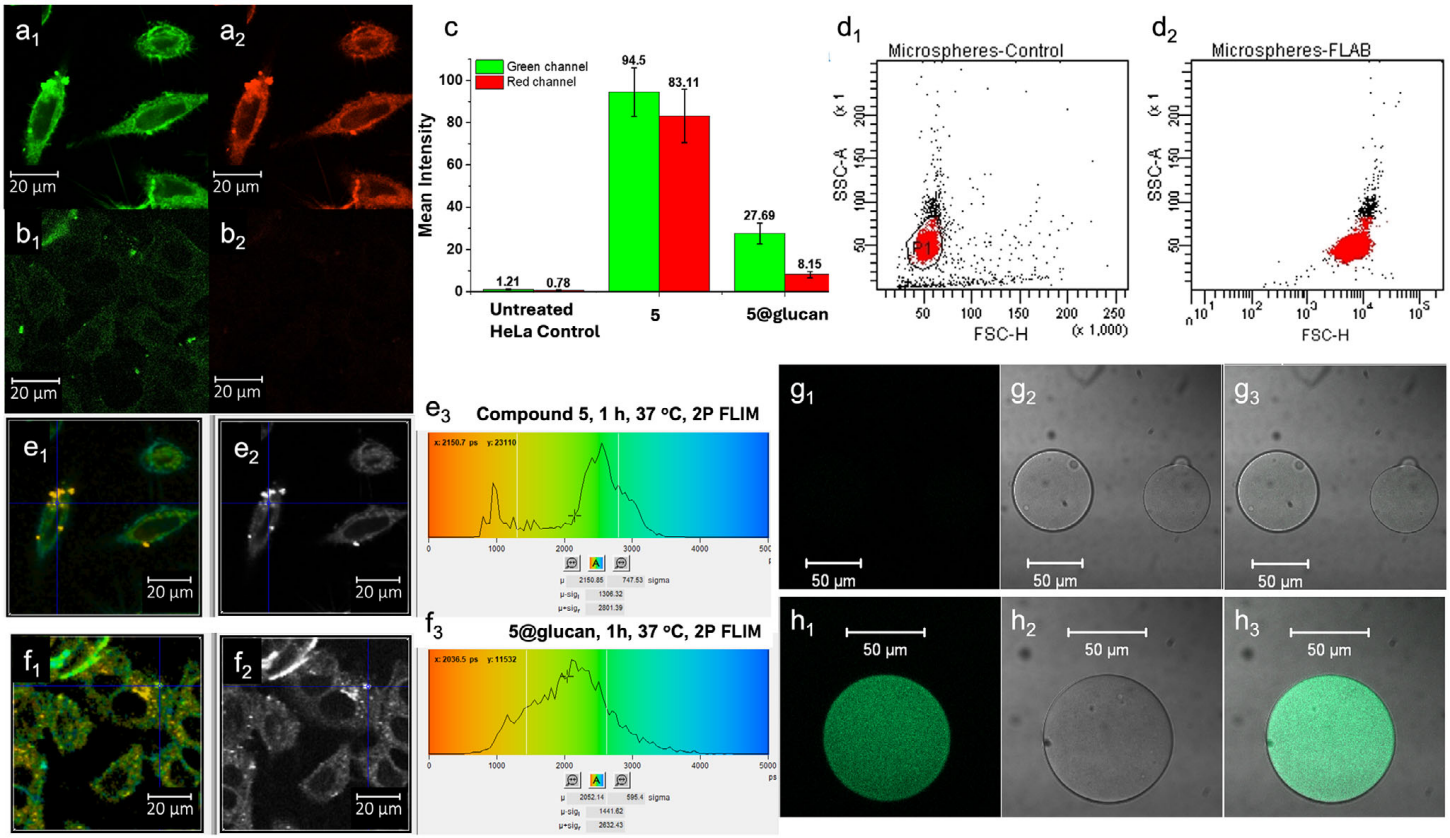
Correlated confocal and multiphoton fluorescence lifetime imaging microscopy (MP‐FLIM) of biotin‐conjugated compound 5 and 5@glucan after 1 h incubation at 37°C in live HeLa cells: (a_1_,a_2_) Confocal fluorescence images of compound 5 (excitation 488 nm; green and red emission channels); (b_1_,b_2_) Confocal fluorescence images of 5@glucan (excitation 488 nm; green and red emission channels); (c) a comparison of the fluorescence emissions of micrographs shown in a1,a2 and b1,b2, with controls (d_1_,d_2_) Flow cytometry analysis of compound 5 (d_2_) demonstrating specific recognition of avidin‐tagged microspheres relative to control samples (shown in d_1_). MP‐FLIM analyses showing lifetime mapping, intensity image, and lifetime distribution: (e_1_–e_3_)(e_3_ and f_3_)cellular behaviour of compound 5 after two photon excitation at 810 nm (laser power 4.5 mW) and (f_1_–f_3_) Correlated MP‐FLIM images displaying lifetime mapping, intensity image, and lifetime biodistribution of corresponding glucan formulation denoted 5@glucan. (h_1_–h_3_) Epifluorescence microscopy of compound 5‐decorated avidin microspheres confirming selective binding microscopy of free avidin‐decorated microspheres (control) and non‐treated beads: (g_1_–g_3_) Additional flow cytometry, epifluorescence imaging and kinetic stability data are given in Figures S9.4–S9.5 and S10.1–S10.2).

Compound 6 demonstrated a substantial lifetime increase from 1.77 ns (in solution) to 3.52 ± 0.30 ns in cells at 37°C. This reduced slightly to 3.30 ± 0.25 ns when the 6@glucan hybrid was incubated with cells (ESI) and further reduced for HeLa cells exposed to compound 6 overnight (2.54 ± 0.38). Interestingly, nanoparticle (NP) conjugates of boronic acids (likely associated through the boronate binding to ─[Si(OH)_2_]_x_ groups) showed fluorescence quenching with respect to free probe (compound 6) as observed via confocal and fluorescence microscopy in CHO and HeLa cells. There are virtually no morphological differences observed between cells treated with compound 6 and the 6@Fe3O4@SiO2 nanohybrid and, consistent with the MTT assays and the timelapse imaging observations there are minimal possible effects arising from nanoparticle interactions and cellular uptake pathways under the short observation times involved. This was supported by 2P FLIM measurements (e.g. FWHM of 2.13 ± 1.22 ns in HeLa cells over 1 h, Figure 9 and Figures S11.1–S11.8). Additionally, both compounds 5 and 6 demonstrated specific binding to avidin. For compound 5, this interaction was confirmed using epi‐fluorescence microscopy and flow cytometry on avidin‐functionalized microspheres via its biotin moiety (excitation at 488 nm), while compound 6 exhibited comparable avidin binding in solution, as verified by fluorescence assays. (Figure 10 and Figures S9.1–S9.5).

### 2.4 In Vivo Tests of Compounds 1–6 and Carbohydrate Co‐Incubation Assays in Zebrafish

**FIGURE 11:**
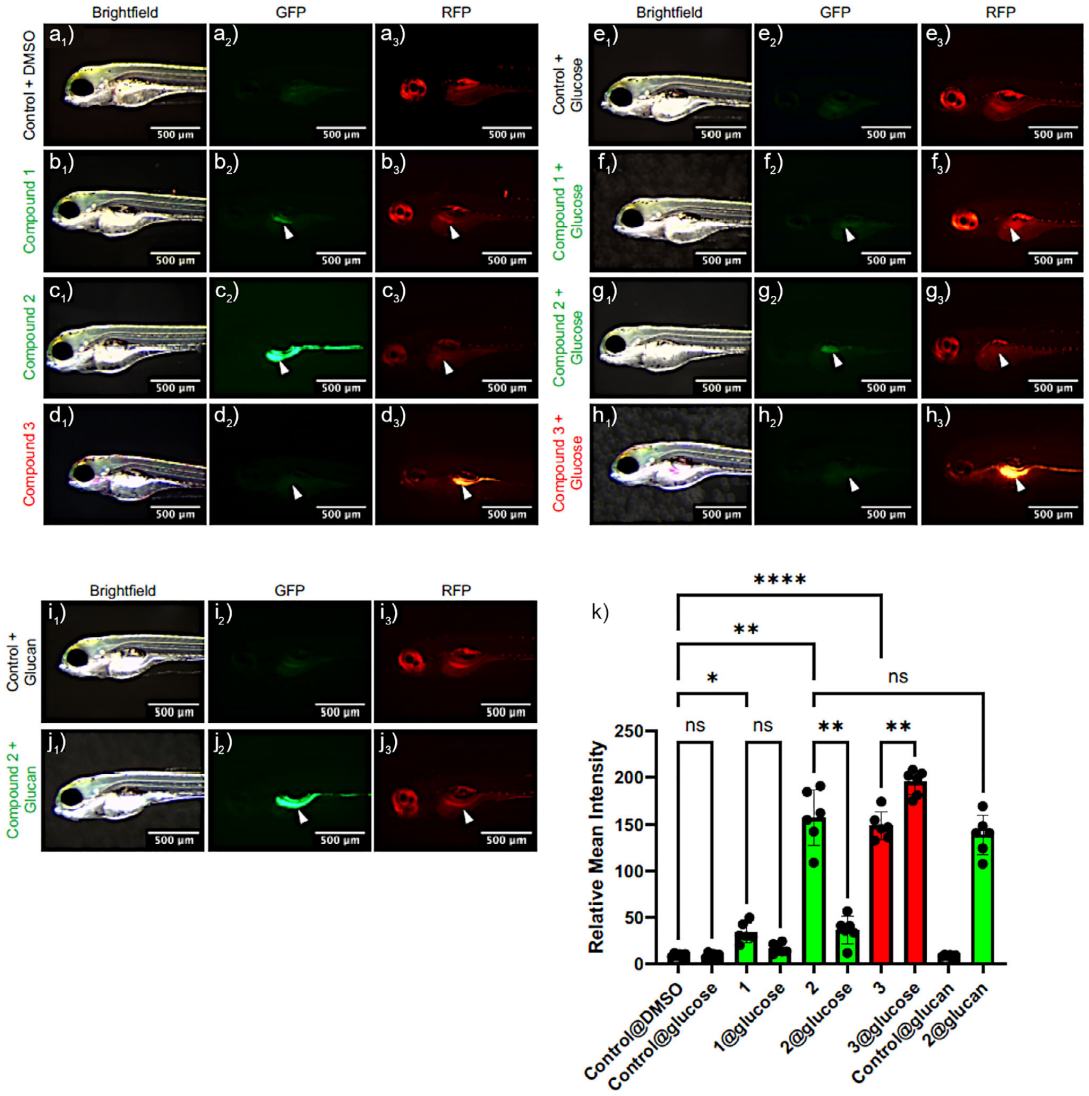
Representative brightfield, GFP and RFP stereomicroscope images of 4 dpf zebrafish larvae incubated with compounds at 50 µM for 120 min at 28.5°C, arrows indicate labelled digestive system, where (a) 1% DMSO in embryo media, (b) 1, 1% DMSO, (c) 2, 1% DMSO, (d) 3, 1% DMSO, (e) 1% DMSO @100 mM glucose, (f) 1, 1% DMSO @100 mM glucose, (g) 2, 1% DMSO @100 mM glucose, (h) 3, 1% DMSO @100 mM glucose, (i) glucan in embryo media, (j) 2, glucan, (k) mean fluorescence intensity of (a_2_–c_2_), (e_2_–g_2_), (d_3_) and (h_3_), (i_2_) and (j2), calculated from six treated larval fish per condition, error bar stands for the standard deviation. (a1–j1) are the micrographs corresponding to the brightfield channel; (a2–j2) is the green channel, compounds were excited at 488 nm and emitted at 510–530 nm; (a3–j3) is the red channel, compounds were excited at 561 nm and emitted at 575–615 nm. Significant difference determined via Brown Forsythe and Welch ANOVA testing and confirmed through post hoc Dunnett's multiple comparison. (^*^) <0.05, (^**^) <0.010, (^***^) <0.001 and (^****^) <0.0001.

To determine whether these compounds can be internalised and provide sensitive glucose detection in vivo, we performed uptake and imaging experiments on zelbrafish, selected for their strengths as a biomedical animal model for compound screening and carbohydrate metabolism [86]. Initially, we focused on identifying the optimal concentration and timing for compound uptake and imaging, as well as assessing zebrafish larvae tolerance to the compounds (see Figures S13.1 and S13.2). These experiments indicated that the optimal conditions for uptake of the compounds were 50 µM for 120 mins at 28.5°C, as denoted by fluorescence detection in digestive organs e.g., the intestinal bulb, which is analogous to the mammalian stomach, and which was assessed by acquiring images using a stereoscope. Once optimised, zebrafish larvae at four days post fertilisation (4 dpf) were incubated with compounds 1–3 in DMSO at (Figure 11), comparing them to fish co‐incubated with the indicated compound and either 100 mM glucose as previously performed [87] (Figure 9) or glucan (Figure S13.2). Figure 11 demonstrates that compounds 2 and 3 were significantly brighter than compound 1 (mean fluorescence intensity, MFI, of 157.12 ± 30.12 and 147.96 ± 15.02 compared to 33.25 ± 10.72), consistent with previous observations in living cells, e.g. HeLa, discussed above. Moreover, compound 2 showed significant quenching when co‐incubated with glucose (MFI decreasing from 157.12 ± 30.12 to 36.24 ± 14.68), while compound 3 displayed enhanced fluorescence in the presence of glucose (MFI increasing from 147.96 ± 15.02 to 195.02 ± 13.65) (Figure 11). Compound 1 appeared to have far less uptake, resulting in minimal change in fluorescence in the presence of glucose (MFI decreasing from 33.25 ± 10.72 to 16.09 ±  5.26) (Figure 11). All three compounds had negligible changes to their fluorescence when co‐incubated with glucan, suggesting a marked difference in uptake of glucan compared to glucose (see Figure S13.2).

**FIGURE 12:**
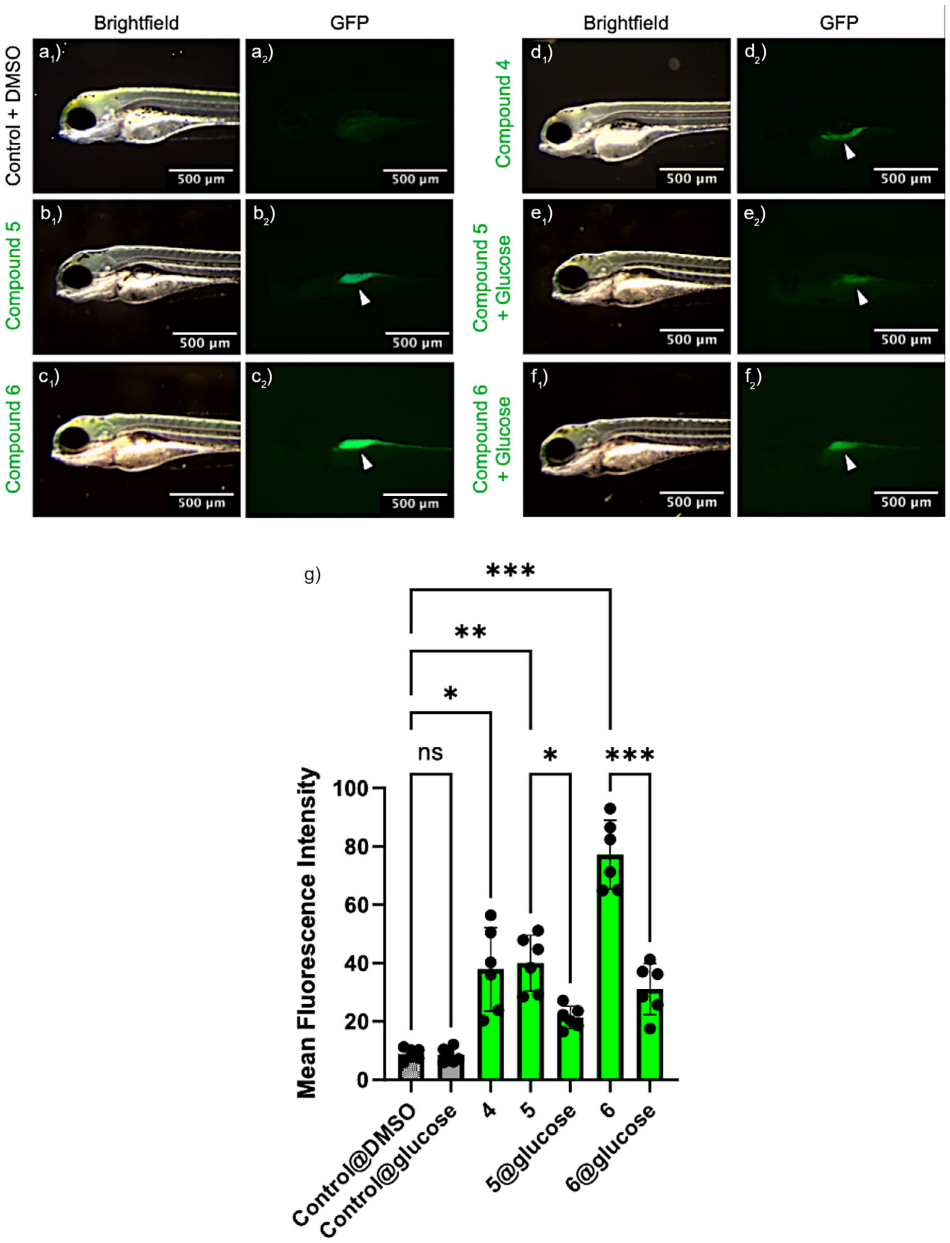
Representative brightfield and GFP stereomicroscope images of 4 dpf zebrafish larvae incubated with compounds at 50 µM for 120 min at 28.5°C, arrows indicate labelled digestive system, where (a) 1% DMSO @100 mM glucose, (b) 5, 1% DMSO, (c) 6, 1% DMSO, (d) 4, 1% DMSO, (e) 5, 1% DMSO @100 mM glucose, (f) 6, 1% DMSO @100 mM glucose, (g) mean green fluorescence intensity of (a_2_–f_2_), calculated from six treated larval fish per condition, error bar stands for the standard deviation. (a_1_–f_1_) is the brightfield channel; (a_2_–f_2_) is the green channel, compounds were excited at 488 nm and emitted at 510–530 nm. Significant difference determined via Brown‐Forsythe and Welch ANOVA testing and confirmed through post hoc Dunnett's multiple comparison. (^*^) <0.05, (^**^) <0.010, (^***^) <0.001 and (^****^) <0.0001.

Compounds 4–6 were also assessed for uptake and carbohydrate‐mediated response. Compound 4 demonstrates minimal uptake (MFI of 37.93 ± 14.28 compared to 8.87 ± 2.04 for Control + DMSO group), as expected from a control probe lacking a boronic acid group (Figure 12). Both compounds 5 and 6 underwent increased uptake into the larval fish, with compound 6 showing the highest uptake (Compound 5 MFI at 40.04 ± 9.59 and Compound 6 MFI at 77.22 ± 11.79) and greatest amount of quenching following glucose co‐incubation (Compound 5 decreasing to MFI of 21.44 ± 3.81 and Compound 6 decreasing to MFI of 31.12 ± 8.75), consistent with Figure 11. Again, co‐incubation of these compounds with glucan showed a lack of significant quenching (Figure S13.2). The strong digestive organ labelling and subsequent signal quenching following glucose treatment reflects the interplay between the alkaline nature of the zebrafish intestinal lumen relative to the rest of the fish (pH > 7.5 vs. pH = 7.2–7.4) [88], the role that the gut plays as the initial interface with the environment [89], the pH sensitivity for the fluorophores involved (e.g. fluorescein) [90] and the function of the boronic acid group responding to glucose. This results in a complex equilibrium that is difficult to deconvolute. By contrast, the lack of quenching following glucan treatment may be due to its structure as a large polysaccharide that cannot cross membranes intact and requires enzymatic breakdown before absorption, unlike glucose that is a small monosaccharide readily transported by glucose transporters expressed in the intestinal epithelium [91].

Therefore, we postulate that the preferential staining of the zebrafish gut by boronic acid‐based fluorophores likely arises from a combination of physiological and chemical factors, driven by the fact that the intestinal environment of zebrafish is known to be more alkaline than other tissues, which can significantly influence fluorophore brightness. For example, fluorescein derivatives exhibit enhanced fluorescence under basic conditions, whereas rhodamine‐based dyes show reduced emission intensity. Second, the gut and epidermis serve as key interfaces with the external environment, exposing them to higher concentrations of small, diffusible carbohydrates such as glucose. Boronic acid dyes, which interact reversibly with *cis*‐diols, are therefore more likely to engage in binding events at these surfaces. Finally, while glucose is readily available and can rapidly enter the gut lumen, larger polysaccharides such as glucans are less likely to be internalized during short exposure periods. Taken together these factors such as local pH effects, fluorophore‐specific photophysical responses, and differential accessibility of carbohydrate species may be responsible for the observed strong, selective gut staining observed in vivo.

**FIGURE 13:**
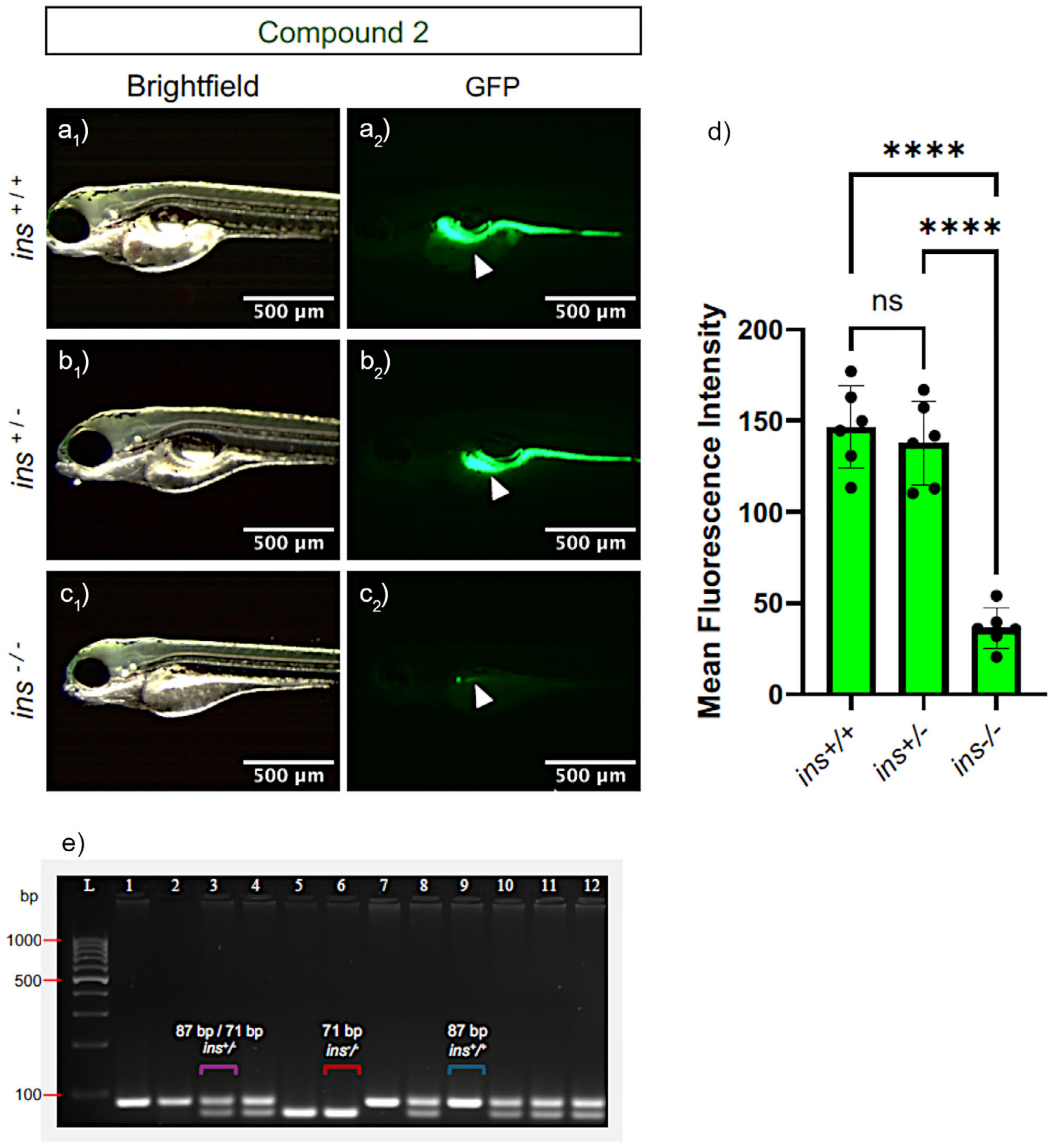
Representative brightfield and GFP stereomicroscope images of 4dpf zebrafish larvae incubated with compound 2 at 50 µM in 1% DMSO for 120 min at 28.5°C, arrows indicate labelled digestive system, where (a) ins^+^/^+^ wild type animal, (b) ins ^+^/^−^ heterozygous animal, (c) ins^−^/^−^ homozygous mutant animal, (d) mean green fluorescence intensity of a_2_–c_2_, calculated from six treated larval fish per condition, error bar stands for the standard deviation, (e) demonstrates post hoc genotyping strategy and identification by PCR and gel electrophoresis on a 3% agarose gel, where the 16 bp difference between the mutant allele (71 bp) and wild type allele (87 bp) is visible. a_1_–c_1_ is the brightfield channel; a_2_–c_2_ is the green channel; compounds were excited at 488 nm and emitted at 510–530 nm. Significant difference determined via Brown‐Forsythe and Welch ANOVA testing and confirmed through post hoc Dunnett's multiple comparison. (^*^) <0.05, (^**^) <0.010, (^***^) <0.001 and (^****^) <0.0001.

From these collective investigations, compound 2 was selected as having the optimal combination of high uptake and large fluorescence difference in the presence of exogenous glucose, to explore differences in endogenous glucose, a more disease‐relevant context. We utilised an incross of a heterozygous zebrafish line carrying the insulin mutation *ins^bns102^* previously shown to have approx. 10‐fold increase in total glucose levels in the homozygous animal developed by Mullapudi [66]. Incubating these fish with compound 2 revealed significant quenching of fluorescence (MFI of 36.35 ± 10.87) in animals that were subsequently genotyped and identified as homozygous for the *ins* mutation, compared to heterozygous (MFI of 137.82 ± 22.87) or wildtype siblings (MFI of 146.39 ± 22.65) from the same mating (Figure 13). The strong quenching seen in this diabetic mutant context can be explained by previous studies demonstrating that in diabetic animals, high blood glucose and inflammation induce abnormal distribution of the glucose transporter GLUT2 as part of the intestinal epithelial metabolic reprogramming, causing retrograde glucose transport from blood to gut lumen, disrupting normal absorption and contributing to metabolic dysregulation seen in diabetes [92].

## 3 Conclusions

Several boronic acid‐functionalised fluorophores were synthesised and evaluated across live cell cultures and zebrafish, demonstrating their capability for membrane permeation, selective organ labelling, and carbohydrate‐responsive behaviour. The compounds exhibited promising properties as fluorescent probes, with successful uptake of most compounds in live cells and substantial labelling of live zebrafish, especially in the digestive organs. *In cellulo*, uptake intricacies of lead fluorescein boronic acid‐based compounds (especially compounds 1 and 2) were resolved via complexation with β‑d‑glucan which significantly enhanced probe internalisation, while in vivo zebrafish experiments revealed that fluorescence signals were modulated predominantly by glucose availability. Further analysis revealed differences in fluorescence signal strength based on incubation time and compound bioavailability. For example, Compound 2 showed increased fluorescence intensity with longer incubation times, while Compound 6 displayed stable fluorescence emission in vitro and in vivo, suggesting differing pharmacokinetic properties. Fluorescence lifetime imaging (FLIM) combined with steady‐state confocal microscopy revealed environmentally responsive fluorescence lifetimes and multi‐exponential decay profiles, consistent with conformational changes upon binding saccharides, and highlighted the temperature‐dependence of uptake. Therefore, the sensitivity and molecular selectivity achieved through multiphoton FLIM imaging validated these constructs as robust tools for interrogating complex carbohydrate‐mediated signalling and transport mechanisms.

These combined results establish boronic acid–based fluorescent hybrids as versatile imaging agents capable of reporting real‐time glucose fluctuations in a living vertebrate organism and represents a pivotal advancement toward monitoring of metabolic processes at cellular and systemic levels. By deploying these compounds in both glucose‐challenged wild type and insulin‐deficient zebrafish, we demonstrate for the first time that synthetic carbohydrate‐recognising fluorescent probes can report on spatial and temporal variations in in vivo saccharide content and flux following either exogenously or endogenously derived perturbations. Beyond their immediate application to glucose surveillance, these results establish a versatile platform for engineering new classes of metabolite‐responsive probes that can function under a broad range of physiologically and pathologically relevant conditions. This convergence of supramolecular chemistry, advanced photonics, and biological modelling shown here sets a new benchmark for metabolic imaging tools in both research and translational medicine, offering a blueprint for developing next‐generation tools that can visualise real‐time metabolic dysregulation, such as that occurring in diabetes or tumour glycolysis. We are now focusing on further developing the theranostic capabilities of these precision bioimaging technologies, not only to observe but ultimately to modulate dysregulated saccharide levels in cells and organs, as well as utilising these compounds in living systems as a future drug discovery platform for screening carbohydrate uptake modulators.

## 4 Experimental Section

All synthetic procedures, characterisation and spectroscopic analysis are detailed in Supporting Information.

### 4.1 Cell Culture and Preparation

Cells were cultured under standard conditions at 37°C in a humidified atmosphere containing 5% CO_2_. Once cultures exceeded 70% confluence, cells were harvested for downstream applications. PC‐3 [93], HeLa [94], MCF‐7 [95], and CHO [96] cell lines were obtained from the American Type Culture Collection (ATCC). PC‐3 cells were maintained in Roswell Park Memorial Institute (RPMI) 1640 medium, while HeLa cells were cultured in Eagle's Minimum Essential Medium (EMEM), both following standard protocols. The media formulations included 10% foetal calf serum (FCS), 0.5% penicillin/streptomycin (10 000 IU/mL and 10 000 mg/mL, respectively), and 1% L‐glutamine (200 mM). All procedures were carried out without phenol red to avoid interference with experimental readouts.

To remove dead cells and excess proteins, the supernatant was aspirated, and adherent cells were rinsed with 10 mL phosphate‐buffered saline (PBS) to eliminate residual serum. Cells were then incubated with 6 mL of 0.25% trypsin in PBS for 8–10 min at 37°C. Trypsin activity was quenched by adding 6 mL of 10% RPMI medium, and the resulting suspension was centrifuged at 1000 rpm for 7 min at room temperature. After discarding the supernatant, the cell pellet was resuspended in 4 mL of 10% RPMI medium. Cell counts were performed using a haemocytometer, and cells were seeded according to experimental requirements.

The FEK‐4 line is widely used in the Pharmacy and Pharmacology Department at Bath and are a kind gift from Prof. Tyrrell's laboratory. This cell line is cultured at the University of Bath in line with local ethical approvals [97, 98, 99]. These cells were cultured using an established EMEM‐based protocol, with medium containing 15% FCS, 0.5% penicillin/streptomycin, and 1% L‐glutamine (200 mM). All cell lines were validated using standard mycoplasma screening protocols and confirmed to be free of contamination. Upon thawing, cell stocks were cultured and maintained in accordance with established guidelines [100], with routine mycoplasma testing and a strict passage limit of ten from the original certified frozen stocks.

### 4.2 Cell Culture for Imaging Procedures

Cells were cultured as monolayers in T75 tissue culture flasks (Falcon) using RPMI 1640 medium supplemented with 10% fetal bovine serum (FBS), L‐glutamine, penicillin, and streptomycin. Cultures were maintained at 37°C in a humidified incubator with 5% CO_2_ and passaged with trypsin once they reached 70%–80% confluence. For fluorescence microscopy, cells were seeded into glass‐bottom Petri dishes three days prior to imaging to ensure proper adhesion, using approximately 7.5 × 10^4^ cells per dish. Before imaging, cells were incubated for 48 h in sterile glass dishes. Fluorescent compounds were prepared as 1 mM stock solutions in DMSO and diluted in RPMI to a final concentration of 10 µM by adding 10 µL of stock to 990 µL of medium. After aspirating the growth medium, cells were washed five times with Hank's Balanced Salt Solution (HBSS), incubated with the compound‐containing medium for 20 h at 37°C, and subsequently washed three times with HBSS to remove non‐internalized compounds. Imaging was performed in 1 mL of serum‐free RPMI.

Confocal fluorescence microscopy was conducted using a Nikon Eclipse Ti2‐E inverted microscope equipped with LU‐N3 laser lines (405, 488, and 561 nm) or a Nikon A1Rsi laser scanning confocal system with a 60× oil‐immersion objective lens. The system included a motorized piezo Z‐stage, halogen lamp, and mercury lamp for widefield fluorescence imaging. Image acquisition and processing were carried out using the NIS‐Elements software package (Nikon Instruments). For cell uptake studies, live PC‐3 or HeLa cells were seeded on glass‐bottom Petri dishes and incubated with fluorescent compounds for 15, 20, or 30 min at 37°C. Control samples were treated with Dulbecco's Modified Eagle Medium (DMEM) containing 1% DMSO to account for autofluorescence. Compounds were dissolved in 1% DMSO and added to the culture medium to reach final concentrations between 10 and 100 µM. Fluorescence lifetime decays were recorded across the entire field of view at 5‐minute intervals, with uptake peaking at 30 min. Representative data were collected after 20 min of incubation.

Epifluorescence microscopy was performed using a Nikon Eclipse TE2000 microscope. Cells were cultured as previously described and seeded into 35 mm glass‐bottom dishes (1.5 mm thickness) at densities of 1.5 × 10^5^ to 2.5 × 10^5^ cells per dish, followed by a 48 h adhesion period. Prior to imaging, cells were washed three times with PBS and replenished with 990 µL of serum‐free medium. Compounds (10 µL in DMSO) were added to reach a final volume of 1 mL and incubated in 1% DMSO. After incubation at either 37°C or 4°C, cells were washed three times with PBS, refilled with fresh serum‐free medium, and imaged immediately. Image processing was performed using Nikon NIS‐Elements AR Analysis software (v4.30.02).

For co‐localization studies, cells previously incubated with compounds and washed were treated with 10 µL of organelle‐specific tracking dyes in 990 µL of serum‐free medium, using final concentrations recommended by the manufacturer. Dyes included ER‐Tracker Red (BODIPY TR Glibenclamide), MitoTracker Red CMXRos, LysoTracker Red DND‐99, ER‐Tracker Green (BODIPY FL Glibenclamide), and MitoTracker Green FM. Dishes were incubated for 20 min at 37°C, washed twice with PBS, and imaged in fresh serum‐free medium. Images were processed using Nikon NIS‐Elements AR Analysis software (v4.30.02), and Pearson correlation coefficients were calculated from three randomly selected fields of view across three independent experiments, with standard deviation used to report error.

Two photon fluorescence lifetime imaging microscopy (FLIM) was performed by raster scanning near‐infrared femtosecond pulsed laser light (910 nm, 200 fs, 76 MHz; Coherent Mira F900, pumped by Verdi V15) through a 60× water‐immersion objective lens (NA 1.2). Emission was filtered using a BG39 filter and detected with a Hamamatsu R3809U photomultiplier tube. Fluorescence decay profiles were recorded pixel‐wise (minimum resolution: 128 × 128 pixels), and lifetimes were calculated using Becker & Hickl SPCImage software (v4) following established protocols. To cater for the high repetition (>40 MHz) rate laser for TCSPC measurements the analysis software used SPCImage (version 4), contains the function ‘Incomplete multiexponentials’, that has the effect of providing correct lifetime calculations and avoids comprising lifetime extractions: all data sets were analysed using this function in line with all previously reported protocols [35, 36, 37, 38, 39, 40, 41, 42].

### 4.3 Zebrafish Imaging Experiments

Zebrafish husbandry: All experiments were conducted with approval from the local ethical review committee at the University of Bath and in accordance with the UK Home Office regulations (Guidance on the Operation of Animals, Scientific Procedures Act, 1986). All work was performed on zebrafish (Danio *rerio*) using wildtype AB strains or the insulin mutant ins^bns102^ strain [66] maintained on an AB background, which were reared at 28.5°C on a 14 h light/10 h dark cycle. ins^bns102^ mutants genotyped post‐imaging, utilising a HotSHOT DNA extraction approach [101], using primers 5′‐GTGCTCTGTTGGTCCTGTTGG‐3′ and 5′‐ CATCGACCAGATGAGATCCACAC‐3′, analysing the PCR products on a 3% agarose/TBE gel. Staging and husbandry were performed as previously described [102]. Embryos were raised in E3 media (5.0 mM NaCl, 0.17 mM KCl, 0.33 mM CaCl_2_, 0.33 mM MgSO_4_) with 0.1% methylene blue up to the point of experimentation, according to standard protocols.

Zebrafish compound treatment and imaging experiments: Compound treatment was performed at 50 µM in 1% DMSO for the indicated period of time (20, 60 or 120 min), using E3 media without additional methylene blue. Following treatment, larvae were washed for 3 × 5 min in E3 media, followed by immediate imaging. Prior to imaging, zebrafish larvae (4 dpf) were anaesthetised using Tricaine (Sigma–Aldrich). Fluorescent stereomicroscope images were generated on a Leica MZFLiii system (Leica Microsystems) and captured using an Olympus EP50 camera (Evident).

Zebrafish image analysis and statistics: Fiji (ver. 2.16.0) was used for all image analysis. Fluorescent regions of interest (ROIs) were selected by utilising the “wand” tool (Legacy mode), and these were used to quantify fluorescence intensity for the digestive system. Mean fluorescence intensity values were extracted for each ROI. Mean values were calculated and plotted using GraphPad Prism (ver. 10.6.1). Statistical analysis was carried out using a one‐way ANOVA test. Results are represented as mean ± SEM. The criterion for statistical significance was set at *p* < 0.05.

## Author Contributions

**Haobo Ge**: investigation. **David Gonzalez Calatayud**: writing – review and editing, investigation. **Melita Tvardauskaite**: investigation. **Max Jewell**: investigation. **Sabrina Wang**: writing – review and editing, investigation. **Tony D. James**: conceptualization, writing – review and editing, supervision, funding acquisition. **Stanley W. Botchway**: investigation, writing – review and editing, conceptualization. **Hanna Aitessaid**: investigation. **Charareh Pourzand**: writing – review and editing, supervision. **John S. Fossey**: writing – review and editing, investigation. **Julia Dudzic**: investigation. **David Gurevich**: investigation, writing – review and editing, conceptualization. **Sofia I. Pascu**: conceptualization, investigation, writing – review and editing, funding acquisition, supervision. **Stephen E. Flower**: writing – review and editing, investigation.

## Conflicts of Interest

The authors declare no conflicts of interest.

## Supporting information




**Supporting File 1**: advs77481‐sup‐0001‐SuppMat.pdf.

**Supporting File 2**: advs77481‐sup‐0002‐SuppMat.zip.

**Supporting File 3**: advs77481‐sup‐0003‐SuppMat.zip.

## Data Availability

The data that support the findings of this study are available in the supplementary material of this article.
