# Supplementary material for: Carbohydrate‐Mediated Cellular Uptake of Boronic Acid Hybrids In Cellulo and Insulin‐Deficient Zebrafish: Live Imaging and Application of Multiphoton FLIM
Source: Adv Sci (Weinh). 2026 Sep 10:e77481. Online ahead of print. doi: 10.1002/advs.77481 (PMC13562794; doi:10.1002/advs.77481)
Supplement: Supplementary file 1 [file ADVS-9999-e77481-s002.pdf]

Carbohydrate-mediated cellular uptake of boronic acid hybrids *in cellulo* and insulin-deficient zebrafish: live imaging and application of multiphoton FLIM

Haobo Ge,[a] John S. Fossey,[a,b] David Gonzalez Calatayud,[c,d] Stephen E. Flower,[a] Sabrina Wang,[a,e] Hanna Aitessaid,[f] Melita Tvardauskaite,[f] Julia Dudzic,[f] Max Jewell,[a] Charareh Pourzand,[f,g] Stanley W. Botchway,[h] David Gurevich,*[f,g,i] Tony D. James,*[a] and Sofia I. Pascu*[a,g,i]

[a.] *Department of Chemistry, University of Bath, Claverton Down, Bath, BA2 7AY, UK, s.pascu@bath.ac.uk, t.d.james@bath.ac.uk*
[b.] *School of Chemistry, University of Birmingham*
[c.] *Department of Inorganic Chemistry, Universidad Autónoma de Madrid, Campus de Cantoblanco, 28049, Madrid, Spain*
[d.] *Instituto de Cerámica y Vidrio – CSIC, Campus de Cantoblnaco, 28049, Madrid, Spain*
[e.] *Merry Life Biomedical Company, Ltd.: W308, No. 1, Nan-Tai St., Yung-Kang, Dist., Tainan City 71005, Taiwan.*
[f.] *Department of Life Sciences, University of Bath, Claverton Down, Bath, BA2 7AY*
[g.] *Centre of Therapeutic Innovation, University of Bath, Bath, BA2 7AY, UK*
[h.] *Central Laser Facility, STFC Rutherford Appleton Laboratory, and Research Complex at Harwell, Harwell Campus, Didcot, OX11 0QX, UK*
[i.] *Centre for Bioengineering & Biomedical Technologies (CBio), University of Bath, Bath, BA2 7AY, UK*

Table of Contents

1. Synthesis and characterisation of compounds S2
2. Synthesis and characterisation of nanoparticles S51
3. Cell viability tests S60
4. Laser scanning confocal fluorescence microscopy S69
5. Colocalisation Assays with Commercial Dyes S89
6. Two photon fluorescence spectroscopy S96
7. Fluorescence lifetime imaging microscopy S103
8. Fluorescence titrations S137
9. Avidin binding tests S141
10. Kinetic stability tests S145
11. Imaging of core-shell nanoparticles labelled with compound 6. .... S146
12. Aggregation by DOSY and proposed 3D structures of β-d-glucan strands labelled with compound **1** S151
13. In vivo assays S155
14. References S158

1. Synthesis and spectroscopic characterisation of compounds

All solvent and chemicals used were HPLC-grade (High-Performance Liquid Chromatography) unless otherwise stated and Milli-Q quality water was used. These were purchased from Fisher Scientific and Sigma Aldrich Company Ltd and were usually used without further purification unless otherwise specified. Most of the cell culture consumables, such as culture media, cell colocalisation dyes, sterile 96 well plates were obtained from Sigma-Aldrich Company Ltd, Invitrogen Company and Fisher Scientific. Carbon dioxide cylinders used in cell culture was supplied by BOC.

High Performance Liquid Chromatography (HPLC): HPLC analyses were performed using a ThermoFisher HPLC system. Samples were prepared at 1 mg/mL concentration in DMSO and injected onto a Symmetry® C-18 column (4.6 × 260 mm). Detection was carried out with an UltiMate 3000 Diode Array Detector measuring UV/visible absorbance at wavelengths of 200, 300, 400, 450, 500, 600, 700, and 800 nm. The flow rate was set at 0.8 mL/min. The eluents used were solvent A (Milli-Q water with 0.1% trifluoroacetic acid, TFA) and solvent B (acetonitrile with 0.1% TFA), with a gradient method applied as follows: starting at 95% A, reversed to 5% A over 7.5 minutes, maintained at 5% A until 15 minutes, then reversed back to 95% A by 17.5 minutes, and held until 21 minutes. Prior to sample injection, the system was equilibrated for 60 minutes, initially flowing at 0.2 mL/min to wash residuals. The Dionex UltiMate HPG3000 pump was maintained with a solution of 80% Milli-Q water, 15% acetonitrile, and 5% isopropanol. All solvents were HPLC grade, and water was deionized.

Fluorescence Spectroscopy: Fluorescence measurements were conducted on a Perkin Elmer Luminescence Spectrophotometer LS 55, using 1.2 mL quartz cuvettes with a 10 mm path length The instrument was warmed up for 30 minutes before use. Samples were prepared at concentrations ranging from 10^{-5} to 10^{-7} M depending on fluorescence intensity. A pre-scan from 200 to 900 nm was performed to identify optimal excitation and emission wavelengths. Emission spectra were then recorded using the maximum excitation wavelength. Data were processed using FLWinLab v1.60 and Origin 9.1 software.

UV-Visible Spectroscopy: Electronic absorption spectra were recorded on a Perkin-Elmer Lambda 35 spectrometer using 1.00 cm quartz cuvettes. The instrument was stabilized by warming up for 30 minutes prior to measurements. Calibration was performed with the solvent as a standard in the same solvent system, with recalibration required when changing solvents. Data were collected and analysed using UV Winlab software. All solvents were HPLC grade, and water was deionized.

Mass spectrometry data were acquired on a Bruker Daltonics electrospray ionisation – time-of-flight (ESI-TOF) mass spectrometer. Samples were dissolved in HPLC-grade methanol or distilled water, a small amount of dimethylsulfoxide (DMSO, one drop) was added to aid dissolution of the sample. Data acquisition and processing were conducted using the MicroTOF Control and Data Analysis 4.0 software (Bruker Daltonik GmbH).

NMR spectra were recorded in chloroform-*d*, methanol-*d*, dimethyl-d_6 sulfoxide or combination of deuterated solvents, depending on the sample solubility. Spectra were

recorded on a Bruker 250, 300 or 500 UltrashieldTM (^{1}H: 250, 300 or 500 MHz, ^{13}C: 75.5 MHz) at the room temperature. Chemical shifts (δ) of NMR spectra were expressed in parts per million (ppm) and using the residual solvent peak as an internal standard. The multiplicity and general assignments of the spectroscopic data were abbreviated to: singlet (s), doublet (d), triplet (t), quartet (q), doublet of doublets (dd), doublet of double of doublets (ddd), doublet of triplets (dt) triplet of triplets (tt), unresolved multiplet (m), apparent (app), broad (br) and aromatic (Ar). Coupling constants are expressed in Hertz (Hz).

Fluorescence Lifetime imaging experiments were carried out at the Rutherford Appleton Laboratory using the OCTOPUS cluster. Laser light at a wavelength of 580 - 630nm nm was obtained from an optical parametric oscillator pumped by a mode locked Mira titanium sapphire laser (Coherent Lasers Ltd) producing 180 fs pulses at 75 MHz. this laser was pumped by solid state continuous wave 532 nm laser (Verdi V18, Coherent Laser Ltd). The oscillator fundamental output of 915 ± 2 was also used. The laser beam was focused to a diffraction limited spot through a water immersion ultraviolet corrected objective (Nikon VC x60, NA1.2) and specimens illuminated at the microscope stage of a modified Nikon TE2000-U with UV transmitting optics. The focused laser spot was raster scanned using an X-Y galvanometer (GSI Lumonics). Fluorescence emission was collected without de-scanning, bypassing the scanning system and passed through a coloured glass (BG39) filter. The scan was operated in normal mode and line, frame and pixel clock signals were generated and synchronized with an external fast microchannel plate photomultiplier tube used as the detector (R3809-U, Hamamatsu, Japan). These were linked *via* a Time-Correlated Single Photon Counting (TCSPC) PC module SPC830. Lifetime measurements were recorded using time correlated single photon counting with an excitation wavelength of 910 nm and measuring the emission at 360-580 nm, for all compounds at three different concentrations, 10, 50 and 100µM in DMSO or PBS Buffer. mission spectral detection was carried out using an Acton Research Component 275 spectragraph and an Andor iDus 740-BU CCD camera. Data were represented as two photon emission lifetime decay spectra with corresponding lifetime decay values for each compound analysed. The data includes for each measurement the goodness of fit of the decay curves given by χ^2, the lifetime of each component and their respective weighting if more than one component is present. The χ^2 value of 1.0 indicates an optimal single exponential fit. Whilst χ^2 value of more than 1.3 indicates an incomplete single exponential fit. Such curves were subsequently fitted to more than one component. Lifetimes calculations were obtained using SPCImage analysis software (Becker and Hickl, Germany) or Edinburgh Instruments F900 TCSPC analysis software.

Atomic force microscopy (AFM) was performed using a Multimode Scanning Probe Microscope (Digital Instruments, Santa Barbara, CA, USA) equipped with a Nanoscope IIIa controller. Images were acquired in tapping mode using an 'E' scanner with a lateral scan range of 12 × 12 µm and a vertical range of 3.5 µm. Silicon cantilevers (Nascatec GmbH, model NST-NCHFR) with a nominal resonant frequency of approximately 320 kHz were employed for all measurements. Tapping mode was selected in preference to contact mode to minimise tip-induced displacement or removal of surface deposits during imaging while maintaining high-resolution topographical characterization.

Cryo-SEM. Samples were mounted onto aluminium stubs using a 1:1 mixture of colloidal graphite and adhesive before being plunge-frozen in liquid nitrogen slush using a cryogenic preparation system (Quorum PP3010T, Quorum Technologies, UK). The rapid cooling in liquid nitrogen slush (approximately −210 °C) enabled cryo-immobilization while minimizing ice crystal formation. Frozen specimens were transferred under vacuum to the cryo-preparation chamber, where they were sublimated for 4 min at −90 °C to remove surface frost. Samples were subsequently sputter-coated with platinum at 5 mA for 90 s to produce a conductive coating before being transferred under vacuum to a field-emission scanning electron microscope (JSM-7900F FE-SEM, JEOL, Japan) for imaging. Micrographs were acquired at an accelerating voltage of 5 kV using a probe current setting of 3 while maintaining cryogenic conditions throughout the imaging process to preserve native sample morphology.

Transmission electron microscopy images were recorded on a JEOL JEM1200, a transmission electron microscope with an operating voltage ranging from 40 to 120 keV and all elemental analysis were carried out on an Oxford Energy Dispersive X-ray Spectrometer (EDS). All spectra were calibrated against a copper standard. In EDX, copper and nickel peaks were consistently observed between 7 and 10 keV because of the grid used to place the samples on.

Dynamic Light Scattering (DLS) measurements were performed using a Zetasizer Nano S instrument (Malvern Instruments, UK). Measurements were carried out at 25 °C using a 633 nm laser (4 mW). Fe_3O_4 and Fe_3O_4@SiO_2 nanoparticle suspensions were dispersed in methanol and analysed in disposable semi-micro polystyrene cuvettes (Fisherbrand®). Hydrodynamic particle size distributions were obtained from three independent measurements, and the reported values represent the average hydrodynamic diameter and relative intensity distribution. The DLS-derived hydrodynamic diameters of both Fe_3O_4 and Fe_3O_4@SiO_2 nanoparticles were consistently larger than the particle sizes determined by TEM (approximately 50 nm for Fe_3O_4@SiO_2), reflecting the contribution of the solvation layer and the adsorbed stabilising surfactants (e.g. oleic acid) surrounding the nanoparticles in suspension.

Synthetic protocols:

Synthesis of (3-(3-(3',6'-dihydroxy-3-oxo-3H-spiro[isobenzofuran-1,9'-xanthen]-5-yl)thioureido)phenyl)boronic acid (compound 1)[1]

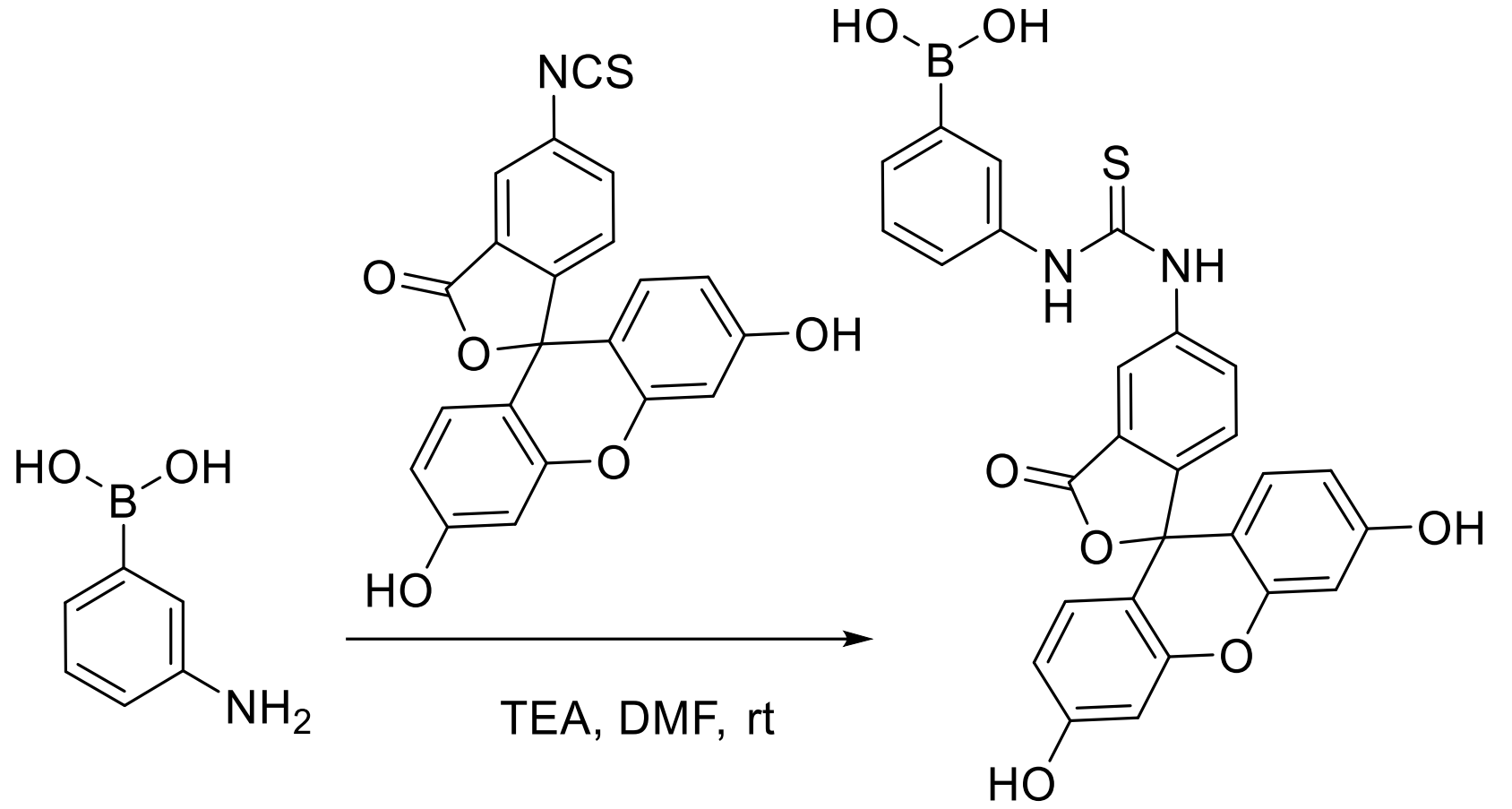

Scheme S1.1. Synthetic route to Probe **1** (Adapted from Reference 1)

3-Aminophenylboronic acid monohydrate (0.287 g, 1.85 mmol) and fluorescein isothiocyanate (0.72 g, 1.85 mmol) were dissolved in DMF (20 mL). Triethylamine (0.5 mL) was added into the solution and stirred at room temperature for 15 hours. Solvents were removed *in vacuo* and the residue was purified by flash column chromatography on silica using DCM:MeOH:hexane 8:1:1, to give boronic acid fluorescein isothiocyanate (FITC) derivative **1** (1.023 g, >99%).

Alternative Synthesis of fluorescein-5-[thioureidyl-(3-phenylboronic acid)] (Compound 1)
Fluorescein isothiocyanate (FITC, mixture of 5(6)isomers, 130 mg, 0.33 mmol) and *m*-aminophenylboronic acid (45.2 mg, 0.33 mmol) were added to a mixture of methanol (12 mL) and distilled water (2 mL). Triethylamine (1 mg) was added dropwise to the reaction mixture. The solution was stirred at room temperature for 1 h, after which an additional 45.2 mg of *m*-aminophenylboronic acid was added. Following a further 1 h of stirring, the crude product was purified by thin layer chromatography (eluent: dichloromethane:methanol, 9:1) resulting in the desired compound in quantitative yield.

^{1}H NMR (300 MHz, D_2O 1:1) δ 7.97 (1H, d, J = 2.7 Hz), 7.74 (1H, dd, J = 8.5, 2.7 Hz), 7.71 (1H, s), 7.60 (1H, d, J = 8.5 Hz), 7.36 (1H, m), 7.19–7.15 (2H, m), 6.63–6.59 (5H, m), 3.35 (2H, br s, NH).
^{13}C{^{1}H} NMR (75 MHz, D_2O) δ 182.2, 179.9, 160.0, 159.9, 141.9, 141.8, 139.7, 133.1, 132.9, 132.1, 131.7, 131.5, 129.7, 128.2, 127.1, 126.6, 123.6, 123.4, 115.5, 114.9, 104.7, 104.5.
^{11}B NMR (96 MHz, D_2O) δ 19.17.

HRMS (ESI$^-$): m/z calcd. for $C_{27}H_{18}BN_2O_7S^-$ [M − H]$^-$ 525.0928; found 525.0946.

Stepwise synthesis of FITC Based Boronic Acid Compound 2 (mixture of isomers)

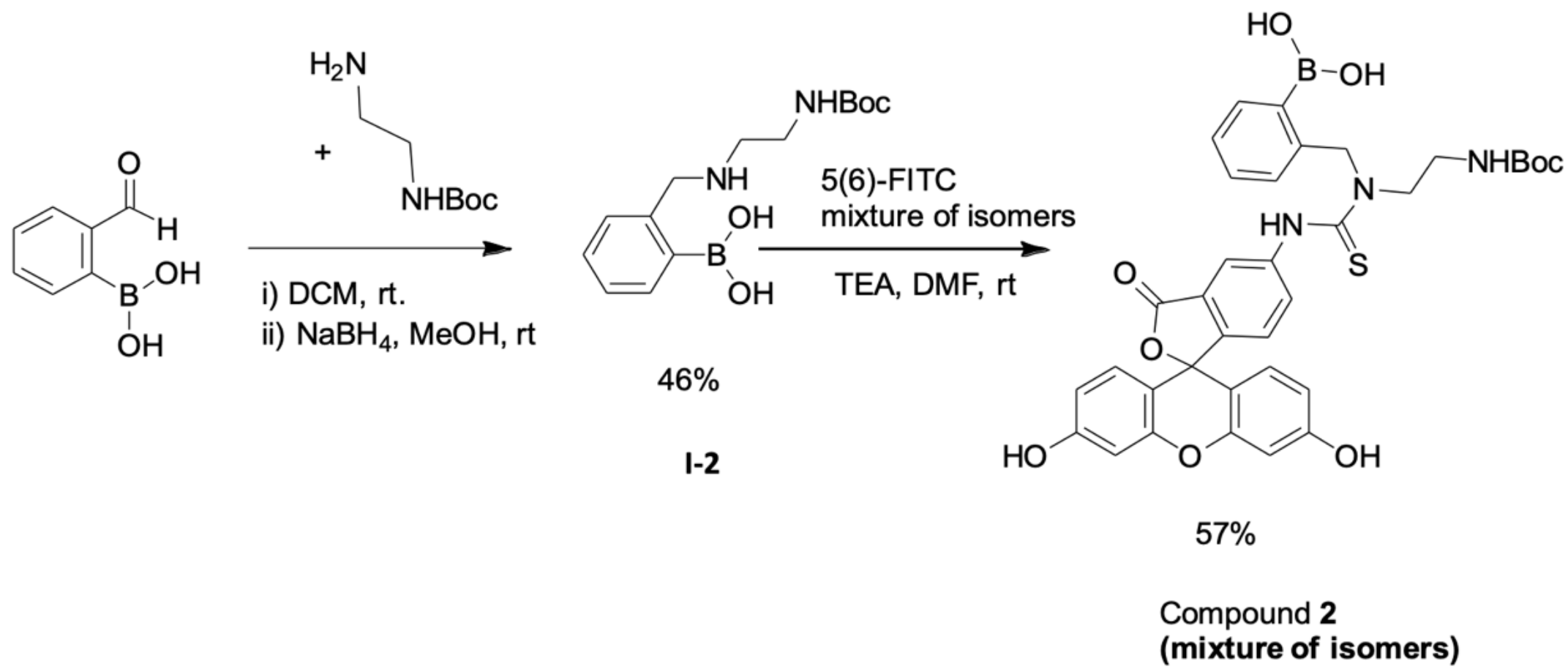

Scheme S1.2. The synthesis of fluorescein isothiocyanate-based boronic acid probe **2**

*Step 1. Synthesis of (2-(((2-((tert-butoxycarbonyl)amino)ethyl)amino)methyl)phenyl)boronic acid (intermediate compound **I**)*

N-Boc-ethylenediamine (1.26 g, 7.86 mmol) and 2-formylphenylboronic acid (1.18 g, 7.86 mmol) were dissolved in dichloromethane (50 mL) and stirred at room temperature for 15 hours. The NMR spectrum of the crude reaction confirmed that the imine had generated, sodium borohydride (0.3 g, 7.9 mmol) and MeOH (10 mL) were added to the solution and stirred for another 2 hours. The reaction was quenched with water. Most of the solvents were evaporated and the residue was dissolved in a small amount of acetone before being poured into water to form the precipitate. The precipitate was washed by water, ether then dried under vacuum to give compound **I-2,** (2-(((2-((*tert*-butoxycarbonyl)amino)ethyl)amino)methyl)phenyl) boronic acid (1.06 g, 46%). The compound was used directly in the next step without further purification.

ESI mass [M+Na+H]$^+$ $C_{14}H_{23}BN_2NaO_4^+$ calculated 317.1643 found 317.1670.

*Step 2. Synthesis of (2-(4-(3',6'-dihydroxy-3-oxo-3H-spiro[isobenzofuran-1,9'-xanthen]-5-yl)-10,10-dimethyl-8-oxo-3-thioxo-9-oxa-2,4,7-triazaundecyl)phenyl) boronic acid (Compound **2**)*

The (2-(((2-((*tert*-butoxycarbonyl)amino)ethyl)amino)methyl)phenyl) boronic acid isolated as described above (**Compound I-2**, 0.159 g, 0.54 mmol) and fluorescein isothiocyanate (FITC, 0.21 g, 0.54 mmol) were dissolved in DMF (20 mL). Triethylamine (1 mL) was added into the solution and stirred at room temperature overnight. After removal of DMF under reduced pressure, the residue was purified by flash column chromatography using DCM:MeOH 1:1 and containing 10 % triethylamine as eluent to give the compound **2** (0.209 g, 57 %).

^{1}H NMR (300 MHz, CD_3OD, ppm) δ 1.30 (s, 9H), 2.70 (br, OH), 3.19 (m, 2H), 3.75 (m, 2H), 4.73 (br, NH), 5.10 (s, 2H), 6.41 (dd, *J* 1.8 and 8.4, 3H), 6.53-6.56 (m, 4H), 6.97 (d, *J* 8.4, 1H), 7.24 (m, 2H), 7.82 (m, 3H)
$^{13}C\{^{1}H\}$ NMR (75.5 MHz, CD_3OD, ppm) δ 29.3, 31.2, 32.2, 37.5, 81.1, 104.0, 112.0, 114.1, 122.7, 125.4, 126.0, 126.2, 128.5, 128.9, 130.8, 133.6, 134.5, 142.8, 144.3, 150.3, 154.6, 159.3, 161.9, 165.3, 171.5, 171.6, 184.1
^{11}B NMR (96 MHz, CD_3OD, ppm) δ 13.67
ESI^- mass $[M-H]^-$ $C_{35}H_{33}BN_3O_9S^-$ calculated 682.2036 found 682.2076

Synthesis of 5-(3-(3-boronophenyl)thioureido)-2-(6-(diethylamino)-3-(diethyliminio)-3H-xanthen-9-yl)benzoate (compound 3, mixture of isomers)

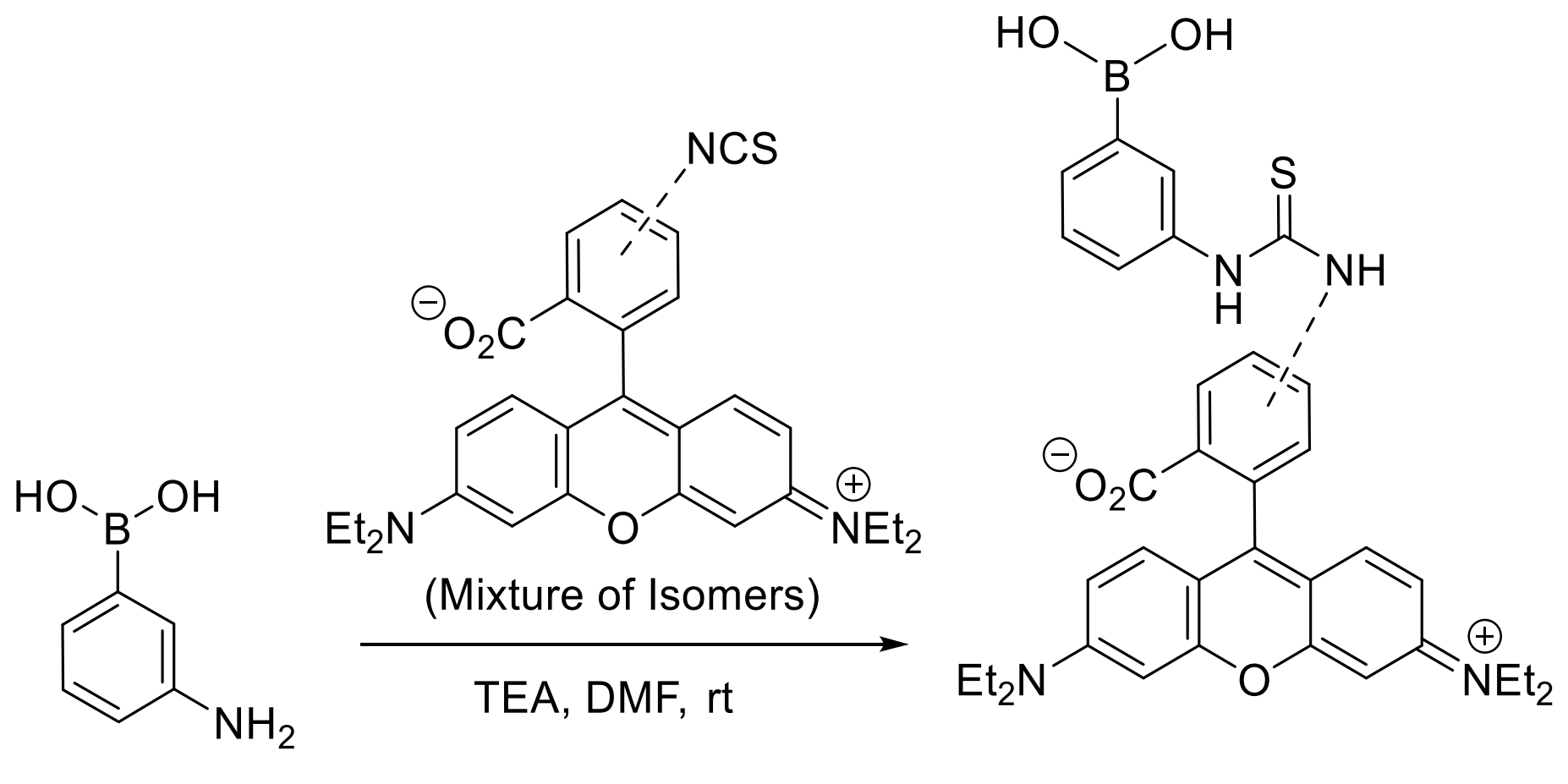

Scheme S1.3. Synthetic route to Probe **3** (Adapted from Reference [2])

Triethylamine (3 equiv., 80 mL, 0.56 mmol) was added to a solution of Rhodamine B isothiocyanate (mixture of isomers, Aldrich, 100 mg, 0.18 mmol) and 3-aminobenzene boronic acid (1.1 eq, 0.21 mmol, 28 mg) in dry DMF (5 mL) under N_2. The reaction mixture was stirred overnight, then the solvent removed in vacuo. The reaction mixture was passed through a short pad of silica eluting with DCM, DCM/methanol (1% to 10%). The solvent was removed to give the product as a dark purple-red solid (65 mg, 58% yield). Characterisation data was consistent with the previously reported compound.[2]

ESI^+ mass $[M]^+$ $C_{35}H_{37}BN_4O_5S$ calculated 636.2578, found 635.2737;
HPLC: 13.4 min (method A)

Synthesis of intermediates and precursors for the stepwise syntheses of Compounds 4, 5 and 6

Synthesis of 2, 5-dioxopyrrolidin-1-yl 5-{2-oxo-hexahydro-1H-thieno [3, 4-d] imidazolidin-4-yl} pentanoate (Biotin-NHS Ester, ***I-A****)*

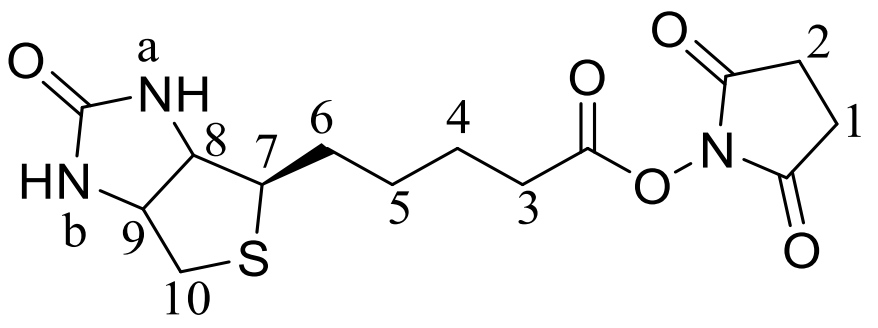

I-A

N-Biotin (488 mg, 2.00 mmol) and N-hydroxysuccinimide (230 mg, 2.00 mmol) were dissolved in dry dimethylformamide (DMF, 20 mL) and the mixture was heated to 100 °C until all solids were fully dissolved. N,N'-Dicyclohexylcarbodiimide (DCC, 412 mg, 2.00 mmol) was then added under a nitrogen atmosphere. The reaction mixture was stirred at room temperature overnight, during which a white suspension formed. The precipitate was removed by filtration, and the filtrate was evaporated under reduced pressure to afford Compound **I-A** (also denoted Biotin-NHS) a white solid (669 mg, 98% yield).[3]

^{1}H NMR (300 MHz, CD_3OD, 298 K) δ: = 6.42 (m, 2H, $H^{a, b}$), 4.29 (m, 1H, H9), 4.13 (m, 1H, H8), 2.83 (d, 2H, H10, J= 5.43 Hz), 2.89-2.54 (m, 4H, H1, 2), 1.87-1.26 (m, 9H, H3-7).
ESI-MS *m/z* calculated for $C_{14}H_{19}N_3O_5S$ $[M + H]^+$: 342.1182; found: 342.1097.
HPLC: *Rt* = 5.2 min.
IR (neat, cm^{-1}): 1711, 1645, 1552, 1061, 861.

Synthesis of N-(6-aminohexyl)-5-{2-oxo-hexahydro-1H-thieno [3, 4-d] imidazolidin-4-yl} pentanamide (I-5)

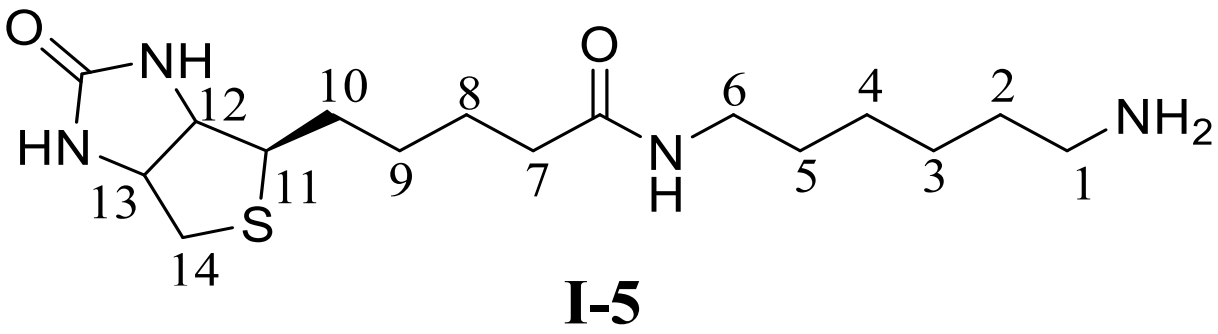

I-5

Route (a) to intermediate Compound ***I-5***

500 mg (1.46 mmol) of Biotin-NHS (Compound **I-A**) was dissolved into 20 mL of DMF solution and 0.3 mL of TEA added. The mixture was sonicated until all white solids were dissolved. Then 1.7 g (14.6 mol) of Hexamethylenediamine was added, dissolved into 50 mL Milli-Q water. Subsequently the mixture was stirred overnight under room temperature. Finally, 100 mL of diethyl ether was added to the mixture and the formation of a white solid was observed. This was separated from the supernatant by filtration and dried in the freeze dryer overnight, 200 mg of white solid of collected (40% yield). The formation of the desired product (Compound **I-5**) was confirmed by mass spectrometry (ESI^+) and ^{1}H NMR spectroscopy.

Route (b) to intermediate Compound ***I-5***

Biotin N-hydroxysuccinimide ester (Biotin-NHS, Compound **I-A**, 500 mg, 1.46 mmol) was dissolved in a mixture of pyridine/H_2O (10:90, 120 mL) and transferred to a 250 mL round-bottom flask. Hexamethylenediamine (1.7 g, 14.6 mol) was dissolved in 60 mL of the same solvent mixture and added dropwise *via* a pressure-equalising dropping funnel. The reaction mixture was stirred overnight at room temperature. The solvent

was evaporated under reduced pressure, and the crude residue was purified by thin layer chromatography followed by reversed phase semi-prep chromatography to isolate the desired compound I-5 in moderate yield.

*Route (c) to intermediate Compound **I-5***

1,6-Diaminohexane (1.7 g, 14.7 mmol) in pyridine/H_2O (9:1, 60 mL) was added dropwise to a solution of biotin N-hydroxysuccinimide ester (Biotin-NHS, Compound **I-A**, 500 mg, 1.47 mmol) in pyridine/H_2O (9:1, 120 mL) at room temperature. The reaction mixture was stirred for 24 h, after which the solvent was removed under reduced pressure. A white residue remained, which was dissolved in minimal methanol and analysed by TLC using 30% NH_3-saturated methanol in 70% $CHCl_3$ (v/v) as eluent, giving two distinct Rf values (Rf = 0.24 and 0.42). Silica gel column chromatography using the same solvent mixture allowed isolation of compound I-5 (200 mg, 40% yield).

*Route (d) to intermediate Compound **I-5***

Biotin N-hydroxysuccinimide ester (Biotin-NHS, Compound **I-A**, 500 mg, 1.46 mmol) was dissolved in DMF (20 mL), followed by the addition of triethylamine (TEA, 0.3 mL). The solution was sonicated until all solids fully dissolved. Hexamethylenediamine (1.7 g, 14.6 mmol) was dissolved separately in Milli-Q water (50 mL) and added to the reaction mixture. The combined solution was stirred overnight at room temperature. Diethyl ether (100 mL) was then added, leading to the formation of a white precipitate. The solid was isolated by filtration and dried under vacuum using a freeze dryer overnight to afford Compound I-5 as a white solid (200 mg, 40% yield).

^1H NMR (300 MHz, CD_3OD, 298 K) δ: 4.52 (t, 1H, H^{13}, J=3.92), 4.34 (s, 1H, H^{12}), 3.07 (m, 1H, H^{11}), 3.10 – 2.85 (m, 2H, H^{14}), 1.83–1.34 (m, 20H, H^{1-10}).
HRMS (FAB^+): *m/z* calcd for $C_{16}H_{31}N_4O_2S^+$ $[M + H]^+$ 343.2168; found 343.2024.
ESI-MS m/z calculated for $C_{16}H_{31}N_4O_2S$ $[M + Na]^+$: 365.1982; found: 365.2005.
HPLC: Rt = 6.7 min

<u>*Synthesis of 5-{2-oxo-hexahydro-1H-thieno[3,4-d]imidazolidin-4-yl}-N-[6-({[4-(tetramethyl-1,3,2-dioxaborolan-2-yl)phenyl]methyl}amino)hexyl]pentanamide (**II-5**)*</u>

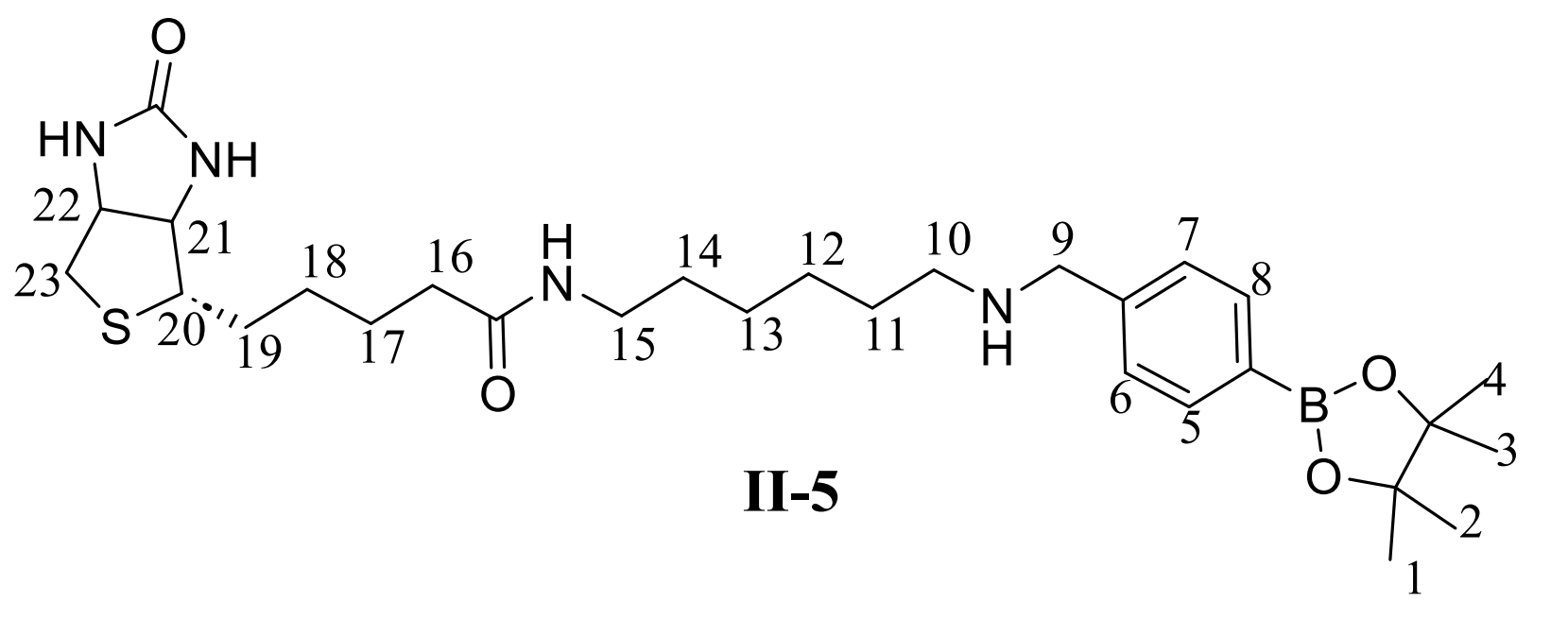

II-5

*Route (a) for the synthesis to intermediate of Compound **II-5***

200 mg (0.61 mmol) of compound **I-5** were dissolved in 20 mL of DMF. To this 92 mg (0.61 mmol) of 4-formylphenylboronic acid and 72 mg (0.61 mmol) of pinacol were added, as well as activated molecular sieves (3 Å). The mixture was then stirred for 16 hours under an inert atmosphere (N_2). Next, 0.61 mmol sodium triacetoxyborohydride was added to this, and the mixture was stirred for a further 24 hours. Finally, the solution was neutralised by excess ethyl acetate, filtered and evaporated under reduced

pressure to give 58.49 mg of compound **II-5** as a white solid (11% yield). The purity was verified by TLC and the identity was confirmed by mass spectrometry (ESI^+) and ^1H NMR spectroscopy.

Route (b) for the synthesis to intermediate of Compound ***II-5***

Compound **I-B** (200 mg, 0.61 mmol) was dissolved in DMF (20 mL). 44-formylphenylboronic acid (92 mg, 0.61 mmol) and pinacol (72 mg, 0.61 mmol) were added, followed by 3 Å powdered molecular sieves. The mixture was stirred under nitrogen for 16 h. Sodium triacetoxyborohydride (129.3 mg, 0.61 mmol) was then added, and the reaction was stirred for a further 24 h. The mixture was neutralised with ethyl acetate, filtered, and evaporated under reduced pressure to yield a white solid. TLC analysis (5% MeOH in DCM) revealed two spots (Rf = 0.15 and 0.73). Purification was performed by silica gel column chromatography, gradually increasing eluent polarity (1%, 5%, and 10% MeOH in DCM) to isolate compound II-B (36 mg).

Route (c) for the synthesis to intermediate of Compound ***II-5***

Compound **I-5** (200 mg) was dissolved in DMF (20 mL). 4-Formylphenylboronic acid (92 mg) and pinacol (72 mg) were added, followed by the addition of activated 3 Å molecular sieves. The mixture was stirred under nitrogen for 16 h. Sodium triacetoxyborohydride (129.3 mg, 0.61 mmol) was added, and stirring continued for another 24 h. The reaction was quenched with excess ethyl acetate, and solvents were removed under high vacuum. The crude residue was purified by TLC and column chromatography to afford compound **II-5** in moderate yield.

Route (d) for the synthesis to intermediate of Compound ***II-5***

Compound **I-5** (200 mg, 0.61 mmol) was dissolved in DMF (20 mL), followed by the addition of 4-formylphenylboronic acid (92 mg, 0.61 mmol), pinacol (72 mg, 0.61 mmol), and activated molecular sieves (3 Å). The reaction mixture was stirred for 16 h under a nitrogen atmosphere. Sodium triacetoxyborohydride (0.61 mmol) was then added, and stirring was continued for a further 24 h. The reaction was quenched with excess ethyl acetate, followed by filtration and evaporation under reduced pressure to yield Compound II-5 as a white solid (58 mg, 11% yield). Purity was verified by TLC, and the identity of the product was confirmed by ^1H NMR and ESI^+ mass spectrometry.

^1H NMR (300 MHz, $(CD_3)_2SO$, 298 K) δ:7.89 (d, 2H, H-5,8, J = 8.32 Hz), 7.54 (d, 2H, H-6,7, J = 8.32 Hz), 4.21 (m, 1H, H-22), 4.04 (m, 1H, H-21, J = 6.94 Hz), 3.41 (d, 2H, H-9, J = 6.93 Hz), 2.75 (dd, 1H, H-23a, J = 5.12 Hz), 2.48 (d, 1H, H-23b, J = 12.28 Hz), 1.79–1.30 (m, 21H, H-10–15, H-16–20), 1.24 (s, 12H, H-1–4).
ESI-MS (m/z): calcd for $C_{29}H_{47}BN_4O_4S$ $[M + H]^+$ 559.3411, found 559.3679. HPLC Rt = 6.4 min.

*Synthesis of 4-(tetramethyl-1, 3, 2-dioxaborolan-2-yl) benzaldehyde (**III**)*

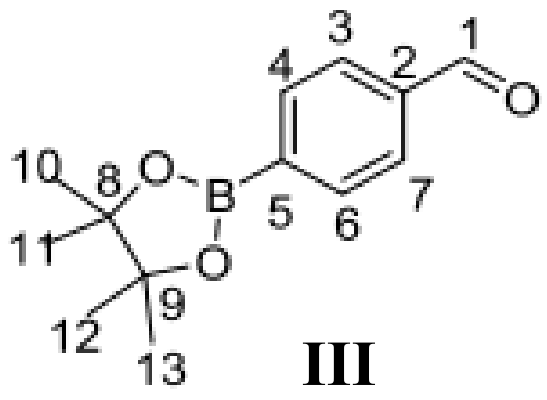

III

14-formylphenylboronic acid (149 mg**,** 1 mmol) and pinacol **(**118 mg**,** 1 mmol) were added to 10 mL THF and 10 mL toluene. The mixture was concentrated to dryness under reduced pressure, and this procedure (solvent addition and evaporation) was repeated three times, affording the product as a white powder in 82% yield. Characterisation details were consistent with literature**.**[4]

^{1}H NMR (300 MHz, $CDCl_3$, 298 K) δ = 10.05 (s, 1H, H1), 7.98–7.79 (m, 4H, H3, H4, H6, H7, J = 8.2 Hz), 1.35 (s, 12H, H10–H13).
^{13}C{^{1}H} (75.5 MHz, $(CD_3)_2OS$, 298 K) δ = 194.8 (C1), 136.6 (C3, C7), 130.1 (C4, C6), 127.0 (C2), 86.0 (C8, C9), 25.6 (C10–C13).
Note: The resonance of C5 was not observed due to broadening caused by quadrupole relaxation effects from the attached boron, which cannot be detected at 300 MHz and 298 K.
Mass Spectrum ESI-MS calculated for $C_{13}H_{17}BO_3$ $[M + H]^+$ 255.1116, found 255.1164.
ESI-MS calculated for $C_{13}H_{17}BNaO_3^+$ $[M+Na]^+$, 255.1163, found 255.1164.
HPLC Rt = 6.6 min.
IR spectrum (cm^{-1}): 1662, 1564, 1505, 1339, 1200, 1184, 1167, 600.

*Synthesis of tert-butyl (4-((5-((3aS,4S,6aR)-2-oxohexahydro-1H-thieno[3,4-d]imidazol-4-yl)pentanamido)methyl)benzyl)carbamate (Intermediate Compound **IV**)*

Route A:
Boc protected *p*-xylylenediamine (141 mg, 0.6 mmol) and compound I (139 mg, 0.6 mmol) were added a flask containing dichloromethane (18 mL), to which sodium triacetoxyborohydride (178.03 mg, 0.6 mmol) was subsequently added. The mixture was stirred 15 h at room temperature under nitrogen. Then 50 mL Milli-Q water was added to eliminate sodium triacetoxyborohydride and compound was washed by 50 mL water three times. The dichloromethane phase was evaporated to dryness to yield 202 mg of white solid (73% yield).

Route B:
250 mg (0.73 mmol) of Biotin-NHS (Compound **I-A**) and 173.2 mg (0.73 mmol) boc protected p-xylylenediamine were dissolved in 10 mL DMF, and then triethylamine (TEA, 4 eq.) was subsequently added. The mixture was stirred at room temperature overnight. The product was evaporated and yielded 220 mg of white solid. (83% yield)

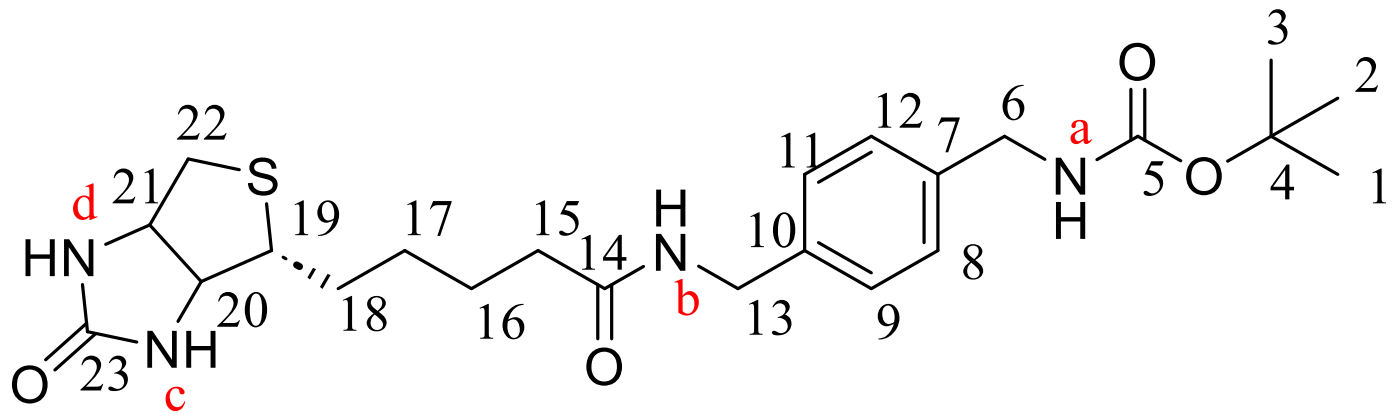

IV

^{1}H NMR (500 MHz, $(CD_3)_2OS$) δ = 8.25 (t, 1H, H^a, J = 5.6 Hz), 7.34 (t, 1H, H^b, J= 5.8 Hz), 7.1 (m, 4H, $H^{8\text{-}12}$), 6.42 (s, 1H, H^c), 6.35 (s, 1H, H^{20}), 4.30 (t, 1H, H^d, J= 5.3 Hz), 4.21 (d, 2H, H^{13}, J = 5.5 Hz), 4.11 (m, 1H, H^{21}), 4.06 (d, 2H, H^6, J = 5.5 Hz), 3.08 (m, 1H, H^{19}), 2.82 (dd, 1H, H^{22}, J = 12.2 Hz, J = 5.0 Hz), 2.58 (m, 1H, H^{22}), 2.13 (t, 2H, H^{15}, J = 7.6 Hz), 1.5 (m, 2H, H^{16}), 1.8 – 0.98 (m, 4H, $H^{17,18}$), 1.39 (s, 9H, $H^{1\text{-}3}$).

^{13}C{^{1}H} NMR ($(CD_3)_2OS$, 500MHz, 298 K) δ = 173.3 (C^5), 172.4 (C^{14}), 163.2 (C^{23}), 139.1 (C^7), 138.5 (C^{10}), 127.5 ($C^{8,12}$), 127 ($C^{9,11}$), 61.5 (C^{21}), 61.2 ($C^{1\text{-}3}$), 55.9 (C^{19}), 43.6 (C^6), 42.2 (C^{13}), 39.5 (C^{22}), 35.6 (C^{15}), 33.7 (C^{16}), 28.7 (C^4), 28.5 (C^{17}), 22.8 (C^{18}).

ESI-MS$^+$ m/z calculated for $C_{31}H_{43}BN_4O_4S$ $[M + Na]^+$ 485.5988, found 485.2300.
HPLC: Rt = 5.1 minutes.

IR spectrum (cm^{-1}): 2980, 1695, 1564, 1508, 1357, 1200, 1140, 1086, 600 cm^{-1} .

Synthesis of FITC–p-xylylenediamine conjugate denoted FITC-diamine intermediate, 4-[({[4-({[(tert-butoxy) carbonyl] amino} methyl) phenyl] methyl} carbamothioyl) amino]-2-(6-hydroxy-3-oxo-3H-xanthen-9-yl) benzoic acid (V)

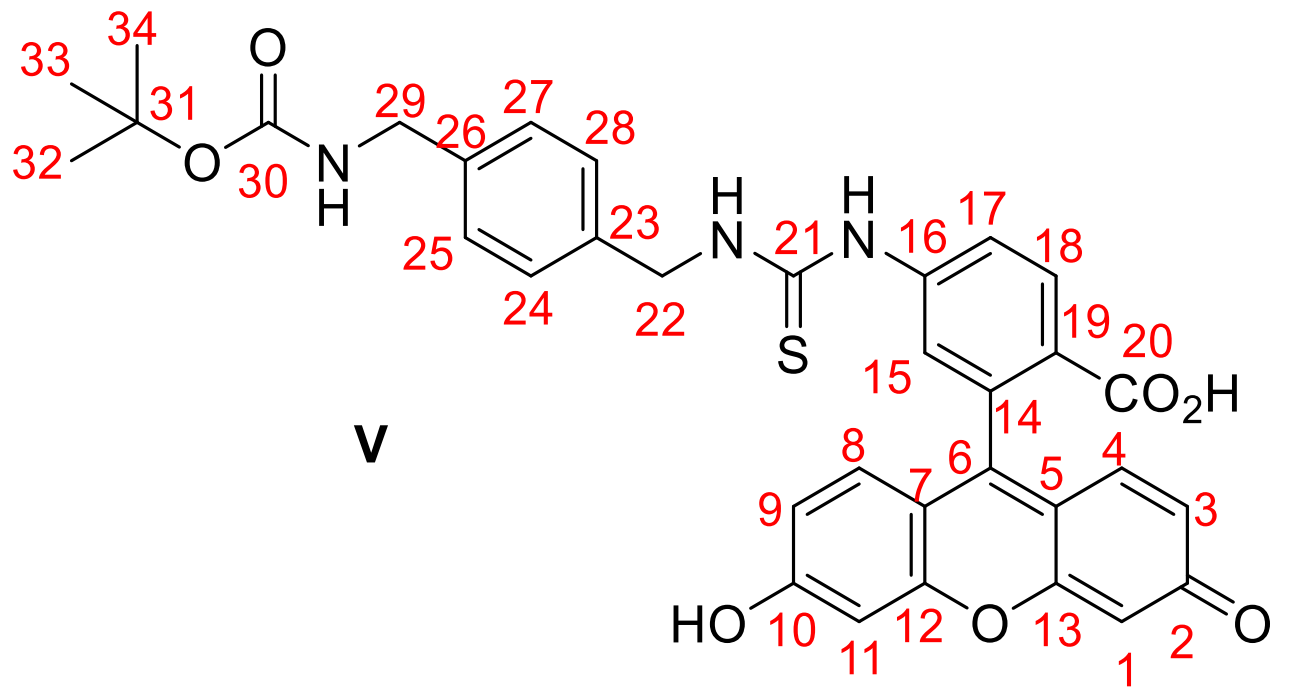

V

FITC (mixture of 5(6) isomers, 196 mg, 0.50 mmol) and *tert*-butoxycarbonyl (Boc) protected *p*-xylylenediamine (118 mg, 0.50 mmol) were dissolved in DMF (10 mL), followed by the addition of triethylamine (TEA, 146.8 µL, 4.0 equiv.). The reaction mixture was stirred overnight at room temperature under protection from light (wrapped in aluminium foil). After completion, the solvent was removed under reduced pressure to afford the desired compound **V** as a yellow solid (200 mg, 63% yield).

^{1}H NMR (300 MHz, $(CD_3)_2SO$, 298 K): δ = 8.29 (s, 1H, H^1), 7.79 (d, 1H, H^3, J = 8.90 Hz), 7.30 (d, 2H, H^{25}, H^{27}, J = 8.22 Hz), 7.21 (d, 2H, H^{24}, H^{28}, J = 7.95 Hz), 7.18 (d, 1H, H^4, J = 7.95 Hz), 6.70 (m, 4H, H^8, H^9, H^{11}, H^{18}), 6.60 (m, 2H, H^{15}, H^{17}), 4.75 (s, 2H, H^{29}), 4.12 (s, 2H, H^{22}), 1.34 (s, 9H, H^{32}–H^{34}).
^{13}C{^{1}H} NMR (300 MHz, $(CD_3)_2SO$, 298 K): δ = 181.1 (C^{21}), 169.1 (C^{20}), 160.0 (C^2), 156.3 (C^{30}), 152.3 (C^{13}), 141.6 (C^{16}), 139.4 (C^{26}), 137.4 (C^{23}), 130.0 (C^4, C^8), 129.6 (C^4,

C[8]), 127.7 (C[25], C[27]), 127.3 (C[25], C[27]), 127.2 (C[24], C[28]), 127.0 (C[17]), 117.2 (C[15]), 113.0 (C[3], C[9]), 102.7 (C[1], C[11]), 48.1 (C[22]), 43.5 (C[29]), 36.6 (C[31]), 28.7 (C[32]–C[34]). ESI-MS (m/z): calculated for $C_{39}H_{37}N_5O_7S_2$ $[M - H]^-$ = 624.1882; found = 624.1861.
HPLC: Rt = 9.0 min.
IR (neat): ν = 3256, 1751, 1696, 1608, 1535, 1247, 1164 cm^{-1}.

Stepwise synthesis of Compound 4

Route (A): Synthesis of 2-(6-hydroxy-3-oxo-3H-xanthen-9-yl)-4-{[({4-[(5-{2-oxo-hexahydro-1H-thieno [3, 4-d] imidazolidin-4-yl} pentanamido) methyl] phenyl} methyl) carbamothioyl] amino} benzoic acid (Compound 4)

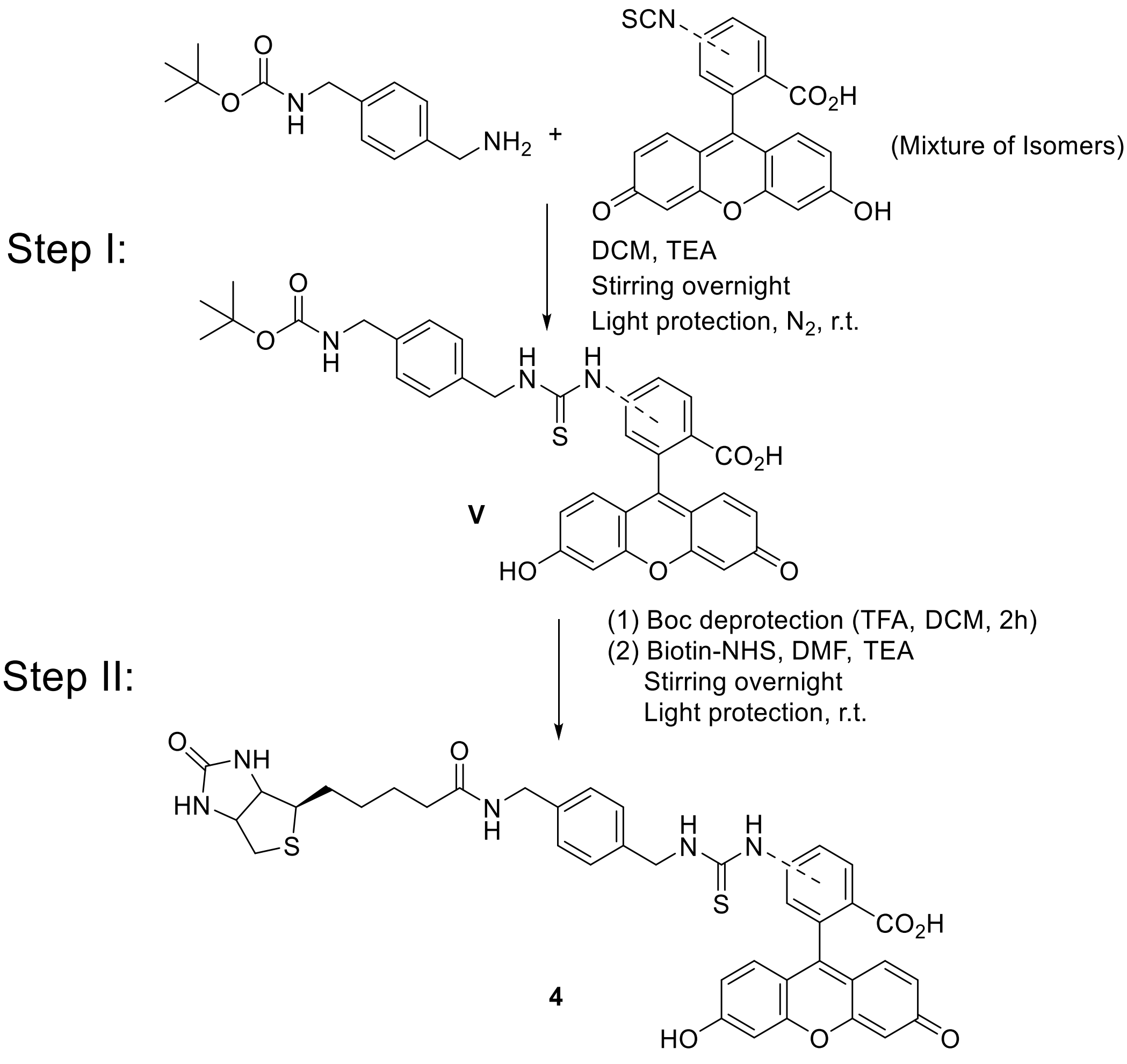

Scheme S1.4. The synthesis of compound **4**

Step I: 118.15 mg (0.5 mmol) Boc protected p-xylylenediamine was dissolved in DCM, 195.69 mg (0.5 mmol) FITC and 4 eq. of triethylamine was subsequently added. The mixture was stirred overnight under room temperature under nitrogen atmosphere and the container was wrapped with foil to protect the mixture from light. The mixture was then evaporated and yielded a yellow solid (intermediate compound **V**).

Step II: 170.69 mg (0.5 mmol) of biotin-NHS was weighed and dissolved into 6 mL of DMF; 262.78 mg (0.5 mmol) of deprotected compound **V** were subsequently added.

Then the mixture was stirred overnight at room temperature with foil protection. After solvent evaporation under reduce pressure, the final product, desired compound **4** was isolated as a scarlet powder (30 % yield).

*<u>Route (B): Synthesis of 2-(6-hydroxy-3-oxo-3H-xanthen-9-yl)-4-{[({4-[(5-{2-oxo-hexahydro-1H-thieno [3, 4-d] imidazolidin-4-yl} pentanamido) methyl] phenyl} methyl) carbamothioyl] amino} benzoic acid (Compound **4**)</u>*

376.0 mg (0.5 mmol) of compound **V** was added to a solution of 2 mmol TFA and 6 mL DCM, the mixture was stirred at room temperature for two hours. Then solvent was removed under reduced pressure, yielding 262.79 mg yellow solid. Subsequently, 262.79 mg (0.5 mmol) of the resulting (de-protected) compound was treated with a solution of 170.69 mg (0.5 mmol) Biotin-NHS (Compound **I-A**) dissolved into 6 mL DMF. The resulting mixture was stirred overnight at room temperature, followed by the removal of volatiles under reduced pressure, to yield 117 mg of the desired Compound **4** as a scarlet-coloured solid (31% yield).

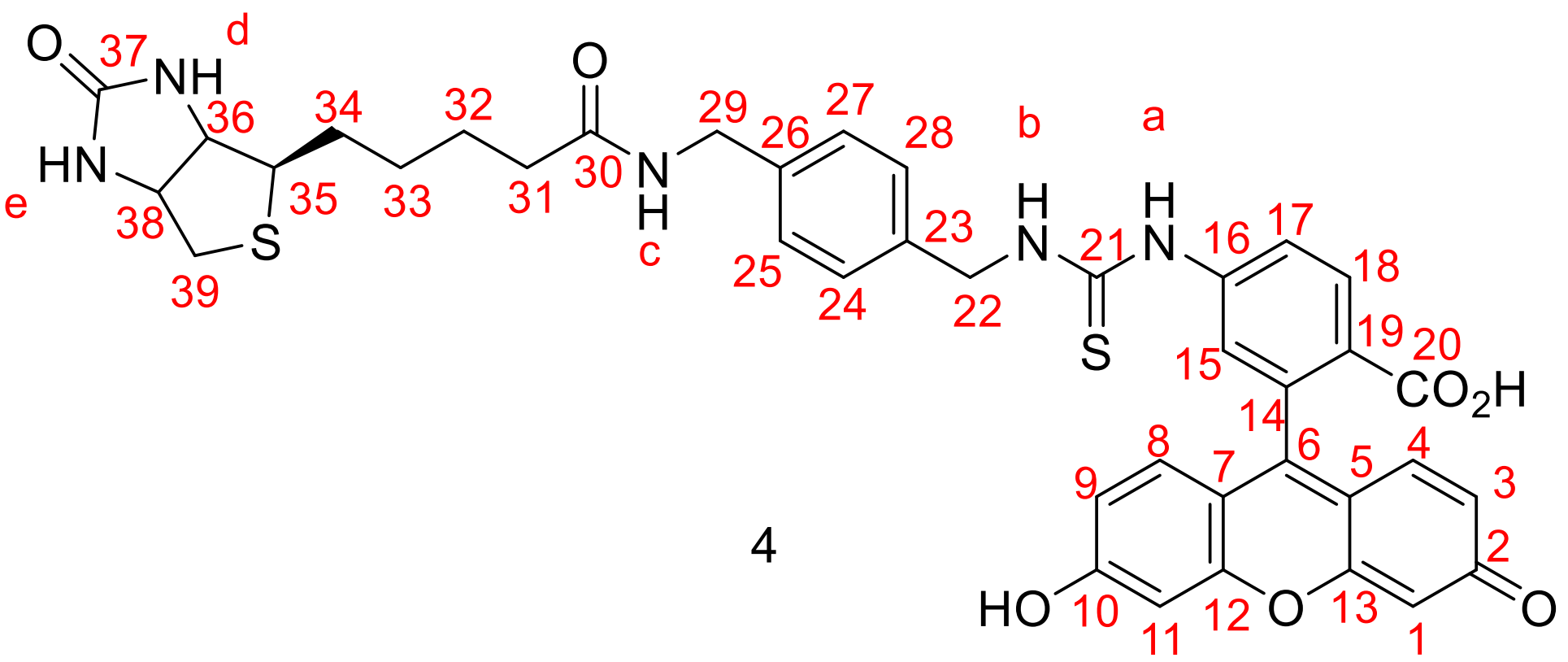

4

^{1}HNMR (300 MHz, CD_3OD, 298 K) δ = 10.70 (s, 1H, H^{20}), 9.03 (s, 1H, H^c), 8.84 (s, 1H, H^b), 8.03 (s, 1H, H^{15}), 7.75 (d, 1H, H^{17}, J = 8.64 Hz), 7.40 – 7.07 (m, 4H, $H^{24, 25, 27, 28}$), 6.93 (d, 1H, H^{18}, J = 8.40 Hz), 6.63 (s, 2H, $H^{4, 8}$), 6.47 (s, 1H, H^d), 6.41 (s, 1H, H^e), 6.02 (dd, 2H, $H^{3,9}$, J^{orth} = 9 Hz, J^{meta} = 2 Hz), 5.92 (s, 2H, $H^{1,11}$), 4.70 (m, 2H, H^{22}), 4.27 (m, 1H, H^{38}), 4.21 (d, 2H, H^{29}, J = 5.36 Hz), 4.10 (m, 1H, H^{36}), 3.07 (m, 1H, H^{35}), 2.80-2.55 (dd, 2H, H^{39}, J = 5.02 Hz), 2.12 (t, 2H, H^{34}), 1.71 (m, 6H, $H^{31,32,33}$).

$^{13}C\{^1H\}$ NMR ($(CD_3)_2OS$, 300MHz, 298 K) δ = 181.2 (C^{20}), 172.6 (C^{21}), 170.2 (C^{30}), 163.8 (C^{37}), 162.9 ($C^{2, 10}$) 158.8 (C^{19}), 141.4 (C^{16}), 139.2 (C^{26}), 138.7($C^{23, 26}$), 130.0 ($C^{4, 8}$), 129.4 (C^{18}), 127.5 ($C^{24, 25, 27, 28}$), 124.3 (C^{15}), 123.2 (C^{17}), 123.1 ($C^{3,9}$), 122.7, 102.9 ($C^{1,11}$), 61.4 (C^{38}), 59.6 (C^{36}), 55.8 (C^{35}), 46.8 (C^{22}), 42.5 (C^{39}), 42.2 (C^{29}), 35.4 (C^{34}), 29.5 (C^{31}), 28.6 (C^{32}), 23.4 (C^{33}).

ESI-MS calculated for $C_{39}H_{37}N_5O_7S_2$ $[M - H]^-$ 750.2062, found 750.2128.

HPLC R_t= 8.7 minutes.

General procedure for the synthesis of Compound 5

The synthesis of Compound **5** involved the stepwise formation of precursor compound **I-5** from the corresponding Biotin-NHS ester (Compound **I-A**) and hexamethylenediamine followed by the reductive amination with 4-formylphenylboronic acid and pinacol to afford intermediate compound **II-5**, and the final coupling with fluorescein isothiocyanate (FITC) to yield the desired compound **5**.

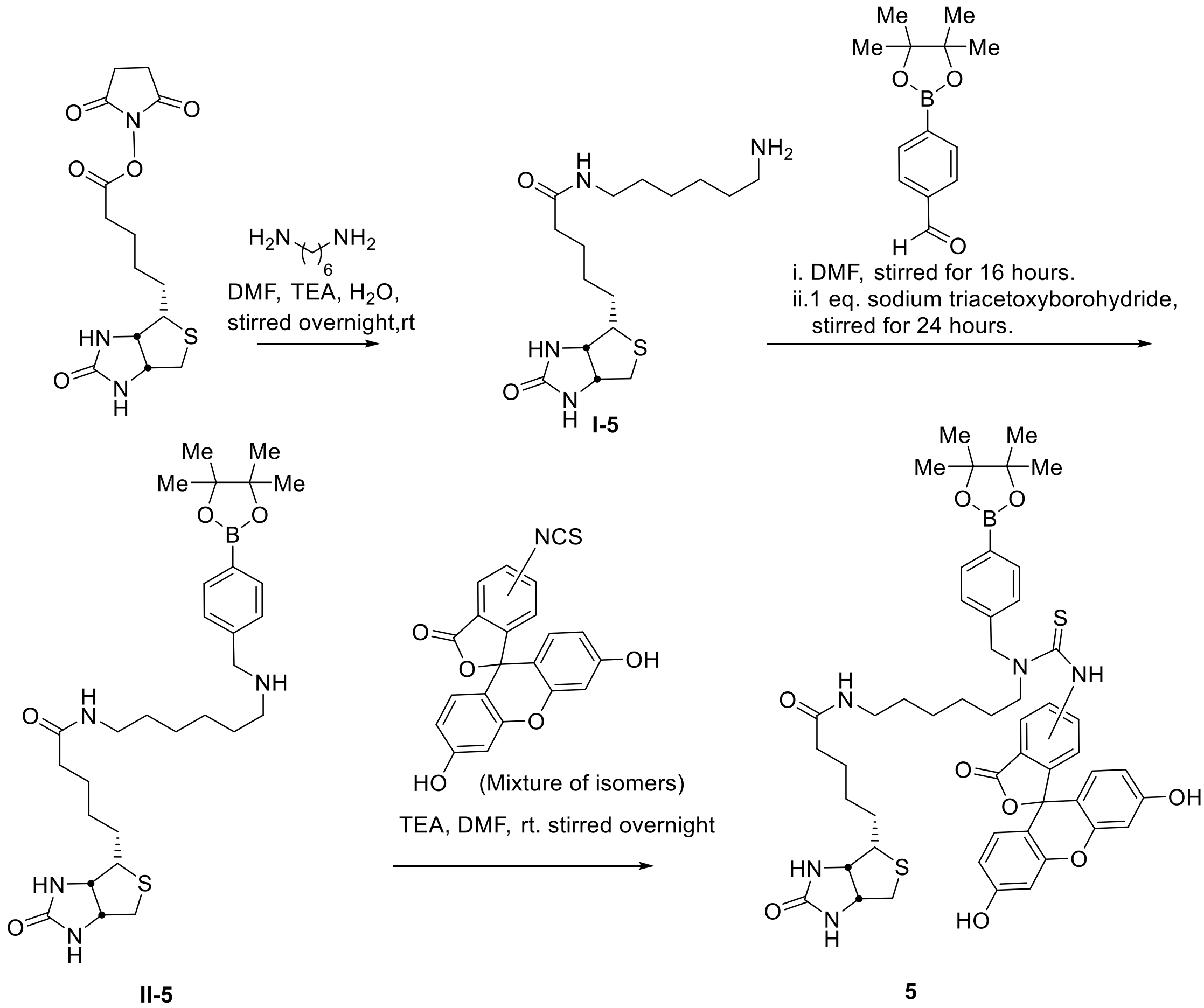

Scheme S1.5. Overview of the synthesis of fluorescein isothiocyanate-based boronic acid compound **5** (mixture of 5(6) FITC isomers).

Route A for the synthesis of the fluorescein-based probe 5:

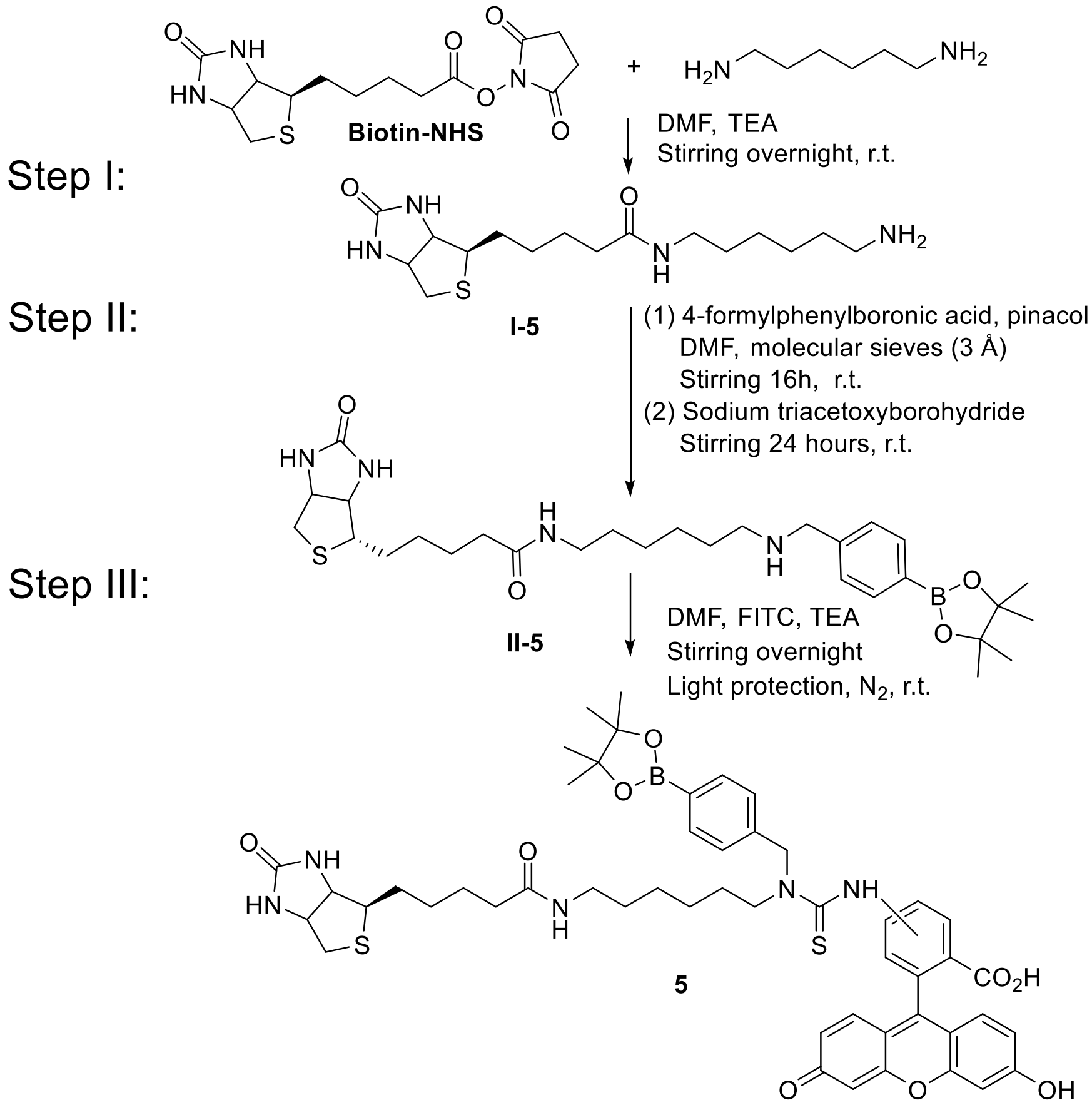

Scheme S1.6. Alternative synthesis of fluorescent compound **5** (mixture of isomers)

Step 1: 500mg (1.46 mmol) of Biotin-NHS **(Compound I-A)** was dissolved into 20 mL of DMF solution and 0.3 mL of TEA was added. Then 1.7 g (14.6 mmol) of Hexamethylenediamine was dissolved into 50 mL Milli-Q water. Subsequently the biotin mixture was loaded into the Hexamethylenediamine solution slowly *via* a pressure-equalising dropping funnel. The mixture was stirred overnight at 1000 rpm under room temperature. Finally, 100 mL of diethyl ether was added to the mixture and a white solid was formed (40% yield). This was separated from the supernatant by filtration and dried in the freeze dryer overnight.

Step II: Compound **I-5** (200 mg,0.61 mmol) was dissolved in 20 mL of DMF. To this, 92 mg (0.61 mmol) of 4-formylphenylboronic acid and 72 mg (0.61 mmol) of pinacol were added, as well as activated molecular sieves (3 Å). The mixture was then stirred for 16 hours under inert atmosphere (N_2). Subsequently, sodium triacetoxyborohydride (129.2 mg, 0.61 mmol) was added and the mixture was stirred for a further 24 hours. Finally, the solution was neutralised by excess ethyl acetate, filtered, and evaporated under reduced pressure to give Compound **II-5** as a white solid (11% yield).

Step III: Synthesis of 2-(6-hydroxy-3-oxo-3H-xanthen-9-yl)-4-({[6-(5-{2-oxo-hexahydro-1H-thieno[3,4-d]imidazolidin-4-yl}pentanamido)hexyl]({[4-(tetramethyl-1,3,2-dioxaborolan-2-yl)phenyl]methyl}) carbamothioyl}amino)benzoic acid

35.8 mg (0.064 mmol) compound **II-5** synthesised as above was dissolved in 1 mL of DMF and excess TEA (1 mL) was added. To this, 30.05 mg (0.064 mmol) of 5(6)-fluorescein isothiocyanate (FITC, mixture of isomers) was added and the mixture was stirred overnight under a nitrogen atmosphere whilst covering the round bottom flask with foil to protect it from light. After evaporation and purification, a 15 mg yellow oily residue was collected (2.5% yield). This compound was characterised by mass spectrometry (ESI^+) and HPLC.

Route B for the synthesis of Compound ***5***

Compound **I-5** (490.5 mg, 1.45 mmol), 4-formylphenylboronic acid (217.4 mg, 1.45 mmol), and pinacol (171.4 mg, 1.45 mmol) were added to DMF (10 mL) in the presence of 3 Å molecular sieves under nitrogen. The reaction mixture was stirred for 16 h, followed by the addition of sodium triacetoxyborohydride (461 mg, 2.175 mmol). Stirring was continued for an additional 24 h. The mixture was neutralised with ethyl acetate, and solvents were evaporated under vacuum to yield compound **II-5** (342.5 mg, white solid). Subsequently fluorescein isothiocyanate (194.7 mg, 0.5 mmol, FITIC, 5(6)-fluorescein isothiocyanate mixture of isomers) was dissolved in DMF (20 mL) and added to a solution of compound **II-5** (236 mg, 0.5 mmol), followed by triethylamine (2 mL). The reaction was stirred overnight under nitrogen, protected from light. The final product (compound 5) was obtained as an orange-yellow solid after drying under reduced pressure, in less than 10% yield after semi-prep HPLC purification.

Route C for the synthesis of Compound ***5***

Compound II-5 (35.8 mg) was dissolved in DMF (1 mL) with triethylamine (1 mL), followed by the addition of fluorescein isothiocyanate (25 mg 5(6)-FITIC, mixture of isomers). The mixture was stirred overnight under nitrogen, protected from light. After reaction completion, the solvent was evaporated under vacuum, leaving a yellow oily residue. A minimal amount of methanol was added, followed by excess pentane to induce recrystallisation. The resulting yellow solid was collected by filtration and washed with pentane to afford compound 5 as an orange solid (35 mg).

^{1}H NMR (300 MHz, MeOD-*d₄*) δ: 8.09 (1H, s, NH), 7.80 (2H, d, J = 2.3 Hz), 7.77 (1H, d, J = 2.2 Hz), 7.11 (3H, d, J = 7.5 Hz), 6.80 (1H, d, J = 9.2 Hz), 6.58 (1H, d, J = 2.7 Hz), 6.49 (1H, dd, J = 2.3, 8.7 Hz), 3.24 (1H, s), 3.21 (2H, m), 3.05 (2H, q, J = 7.4, 14.7 Hz), 1.83 (2H, d, J = 1.4 Hz), 1.20 (12H, s), 1.18 (4H, d, J = 14.5 Hz).
^{13}C{^{1}H} NMR (75 MHz, MeOD-d_4) δ: 180.2, 172.8, 169.1, 160.3, 155.9, 145.1, 135.7, 132.3, 129.6, 105.8, 56.9, 36.0, 32.5, 31.9, 30.1, 28.9, 24.6.
^{11}B NMR (96 MHz, MeOD-d_4) δ: 160.46.
ESI-MS (m/z) calc. for $C_{50}H_{58}BN_5O_9S_2$ $[M+Na]^+$ 969.3688, found 969.4586.
MALDI-TOF MS: *m/z* calcd. for $C_{50}H_{57}BN_5O_9S_2$ $[M - H]^-$ 946.962; found 947.2.
HRMS (low res ESI^-) *m/z* calcd. for $C_{50}H_{58}BN_5O_9S_2Cl$ $[M + Cl]^-$ 982.420; found 982.2.
HPLC R_t= 8.9 minutes.

The stepwise synthesis of 2-(6-hydroxy-3-oxo-3H-xanthen-9-yl)-4-{[({4-[(5-{2-oxo-hexahydro-1H-thieno[3,4-d]imidazolidin-4-yl}pentanamido)methyl]phenyl}methyl)({[4-(tetramethyl-1,3,2-dioxaborolan-2-yl)phenyl]methyl})carbamothioyl]amino}benzoic acid (Compound 6)

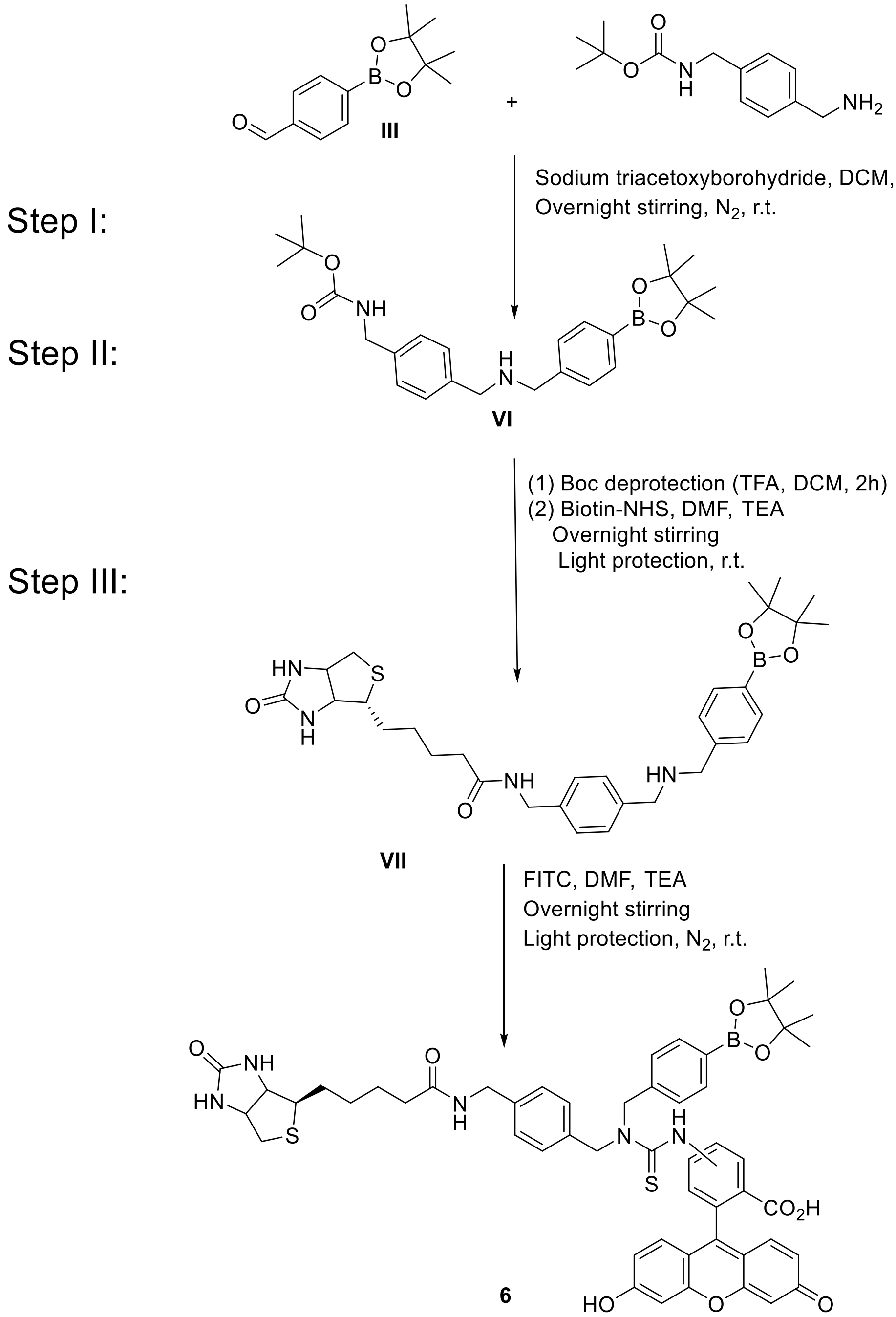

Scheme S1.7. Route A for the synthesis of Compound **6** (mixture of 5(6) fluorescein isomers)

Step 1. A mixture of 149.05 mg (1 mmol) 1, 4-formyl-phenyl-Boronic acid and 118.18 mg (1 mmol) pinacol were added to 10 mL of THF and 10 mL Toluene. The mixture was concentrated to dryness under reduced pressure and this procedure (solvents addition/evaporation) was repeated three times, resulting in the corresponding pinacol protected boronic acid, which was used without further purification.

Step 2. 141.79 mg (0.6 mmol) Boc protected p-xylylenediamine and 139.25 mg (0.6 mmol) pinacol protected boronic acids were added into 18 mL DCM; 178.03 mg (0.6 mmol) Sodium triacetoxyborohydride was subsequently added to this mixture. The resulting mixture was stirred overnight at room temperature under a stream of nitrogen. Then HPLC-quality water (100 mL) was added to eliminate Sodium triacetoxyborohydride and subsequently the compound **VI** was extracted using 50 mL CH_2Cl_2. The volatiles including DCM were evaporated under reduced pressure to yield compound VI as a white solid which was used without further purification.

Step 3. 200 mg (0.85 mmol) of compound **VI** was added to 8 mL TFA / DCM mixture (1:3), the mixture was stirred at room temperature for two hours to remove the boc group. The solvent was evaporated and 150.21 mg (0.85 mmol) of biotin-NHS was added. 6 mL DMF and 4 eq. triethylamine (TEA) were subsequently added to dissolve both compounds, the mixture was stirred overnight at room temperature with the solvent evaporated to yield white solid (compound **VII**).

Step 4. 172.04 mg (0.44 mmol) FITC (mixture of isomers) and 231.43 (0.44 mmol) compound **VII** were dissolved in 10 mL of DMF, 4 eq. of TEA was added subsequently. The mixture was stirred overnight at room temperature covered with foil. The mixture was separated by DCM and water, products were extracted in the organic phase, DCM was evaporated, which yielded the desired compound **6** as a scarlet colour solid (4.4% yield).

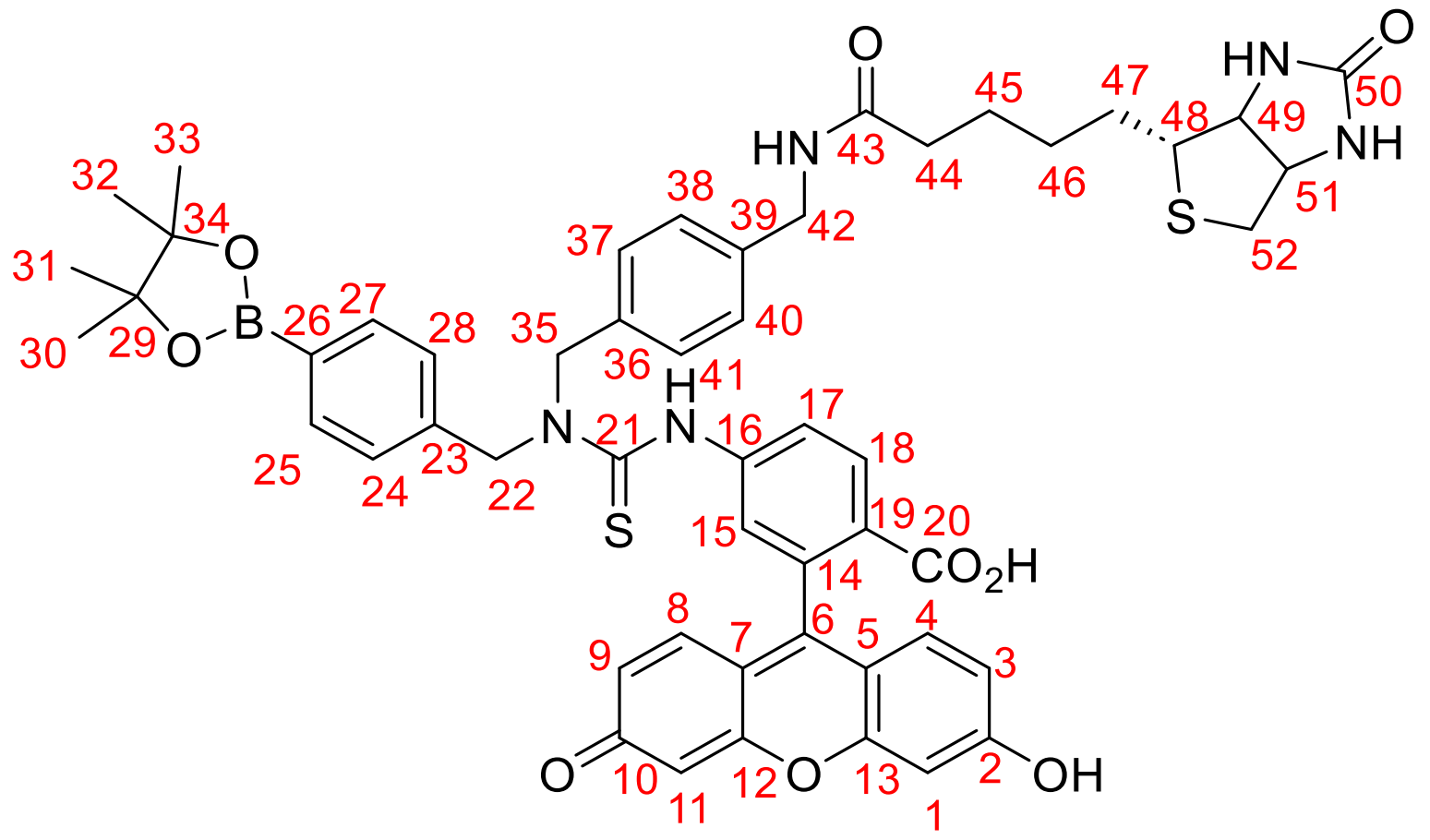

6

^{1}HNMR (300 MHz, CD_3OD, 298 K) δ = 7.65 (d, 2H, $H^{25, 27}$, J=7.83 Hz), 7.28 (m, 5H, $H^{1, 3, 4, 24, 28}$), 6.53 (m, 10H, $H^{8, 9, 11, 15, 17, 18}$), 4.95 (s, 2H, H^{22}), 4.37 (m, 1H, H^{51}), 4.25 (d, 2H, H^{35}, J=8.21Hz), 4.14 (m, 1H, H^{49}), 3.98 (s, 2H, H^{42}), 3.07 (m, 1H, H^{48}), 2.79 (d, 2H, H^{52}, J=4.93Hz), 2.14 (m, 8H, H^{44-47}), 1.27(m, 12H, H^{30-33}).
$^{13}C\{^1H\}$ NMR ($(CD_3)_2OS$, 300MHz, 298 K) δ = 172.4 (C^{21}), 169.3 (C^{43}), 169.0 (C^{20}), 163.1 (C^{50}), 162.6, 157.5 ($C^{2, 10}$), 156.1 ($C^{12, 13}$), 152.7 (C^{14}), 146.4 (C^{43}), 142.9 (C^{16}), 141.6, 141.0 (C^{39}), 139.0 (C^{26}), 138.5 (C^{23}), 134.9 (C^{18}), 134.6 (C^{36}), 129.5 ($C^{4, 8}$), 128.8, 128.4 (C^{17}), 127.4 ($C^{24, 28}$), 127.2 (C^{19}), 126.6 ($C^{38, 40}$), 126.4 ($C^{25, 27}$), 126.0, 125.9, 125.2 (C^{15}), 83.6 ($C^{29, 34}$), 35.5($C^{49, 51}$), 33.7 ($C^{22, 35}$), 28.5 (C^{42}), 28.4 (C^{52}), 25.7 (C^{44}), 25.3 (C^{47}), 25.0 (C^{46}), 24.8(C^{45}), 24.4 (C^{30-33}),
ESI-MS: Calculated for $C_{52}H_{53}N_5O_9S_2B$ $[M - H]^-$ 966.3383, found 966.3471;
MALDI: Calculated $C_{52}H_{54}N_5O_9S_2B$ [M] 968.35, found 968.90.
HPLC R_t= 8.9 min.

Selected NMR data

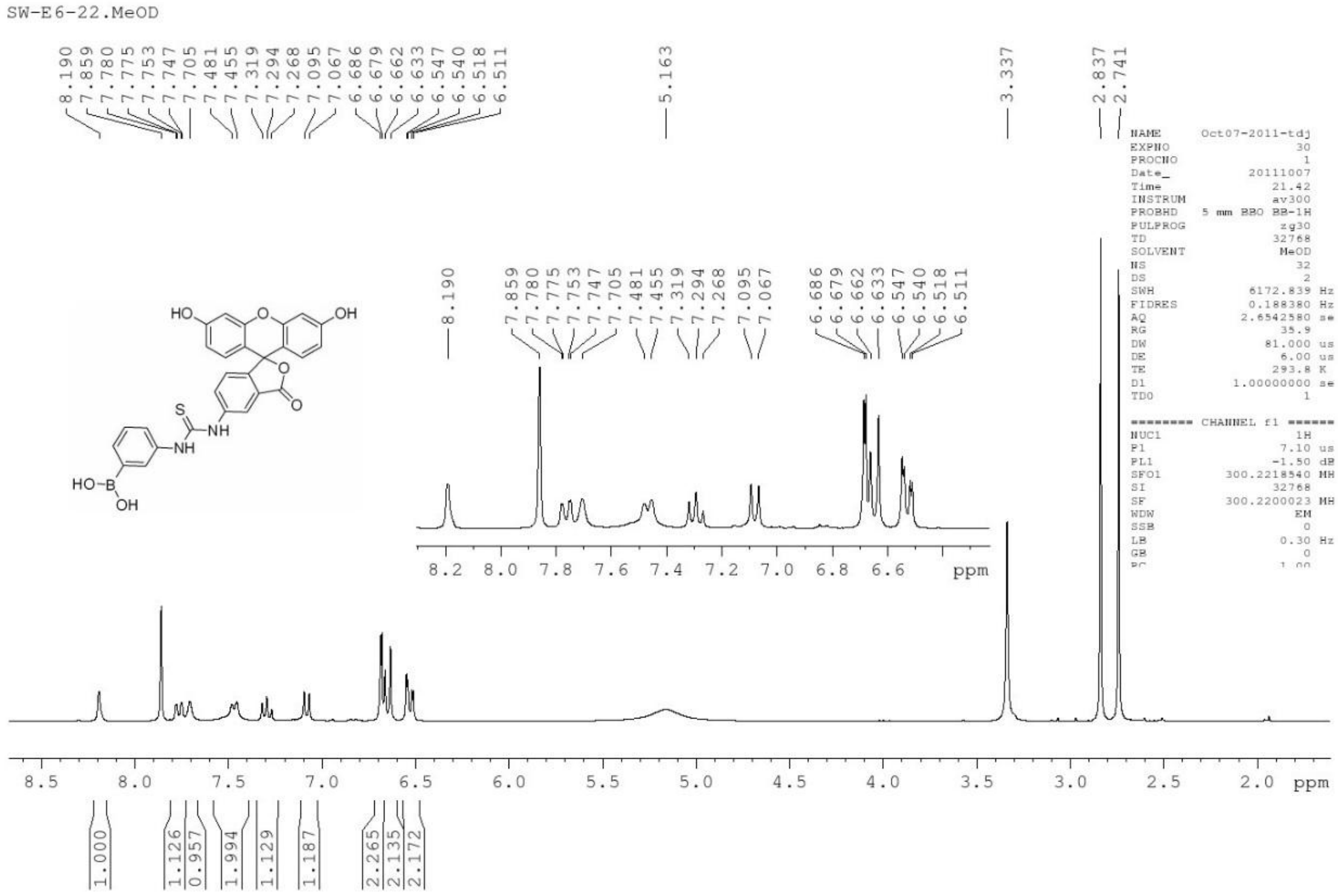

Figure S1.1. ^{1}H NMR spectrum of **1**, in methanol-d_4, 300 MHz, 298 K

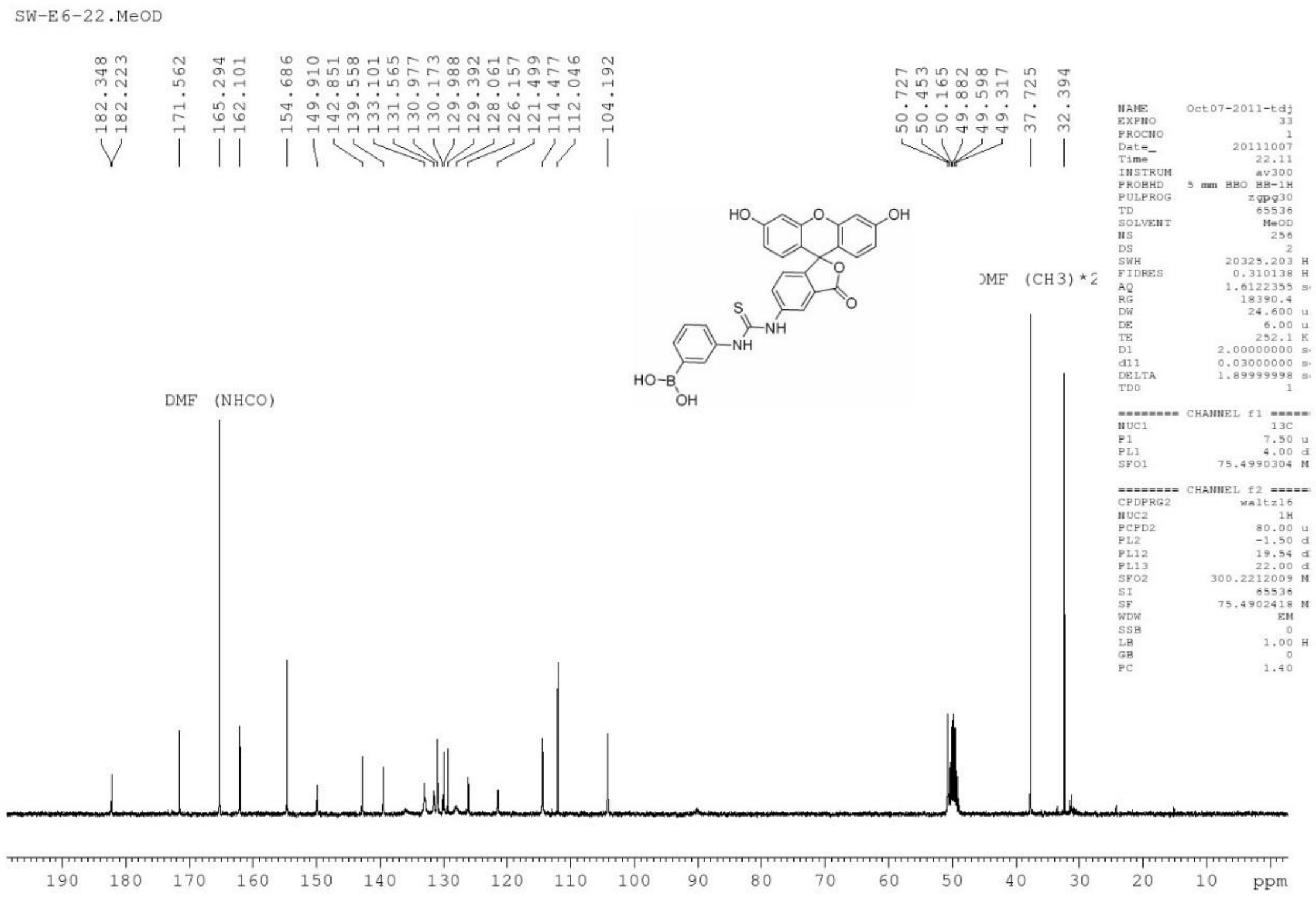

Figure S1.2. $^{13}C\{^{1}H\}$ NMR spectrum of **1**, in methanol-d_4, 75.5 MHz, 298 K

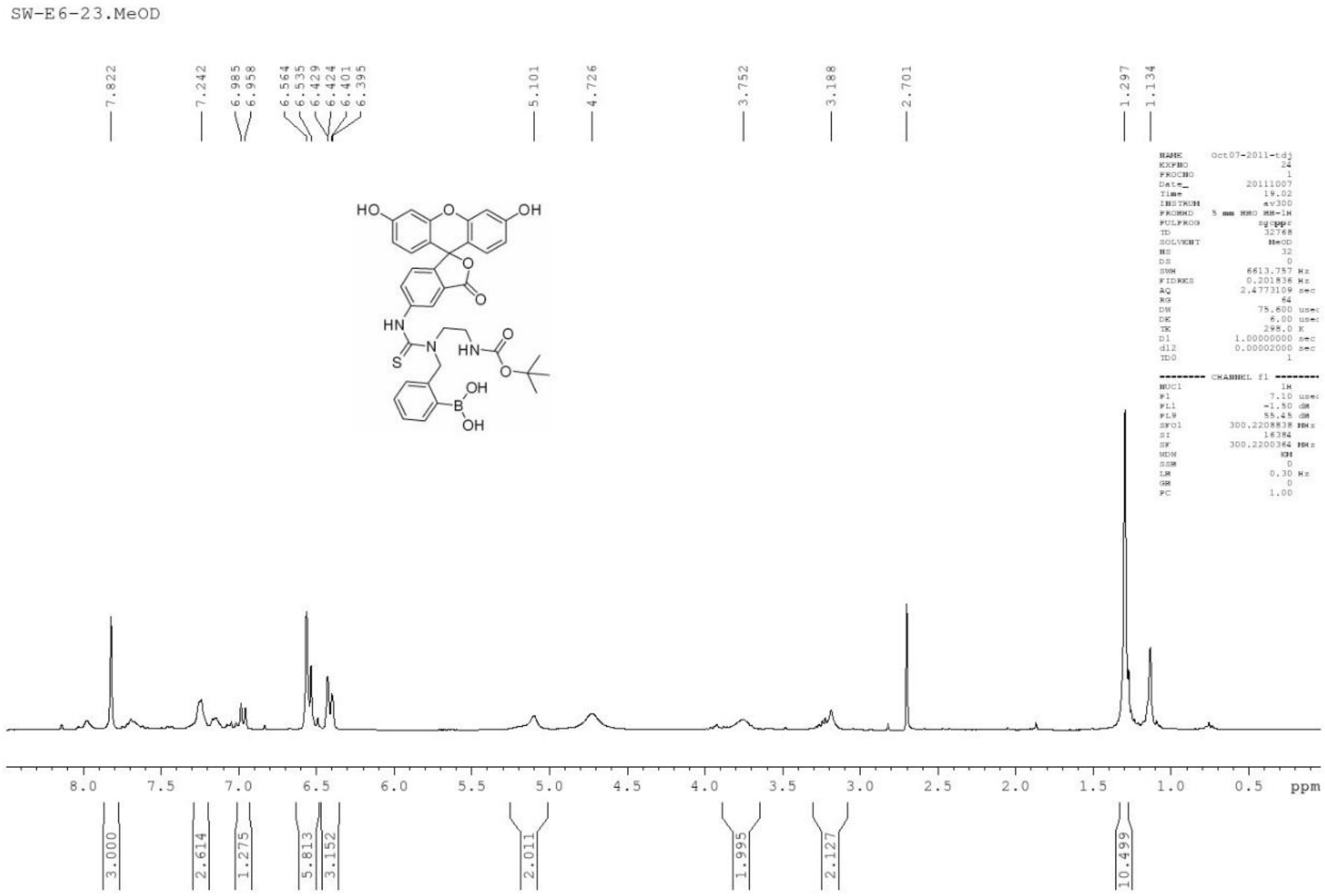

Figure S1.3. ^{1}H NMR spectrum of **2**, in methanol-d_4, 300 MHz, 298 K

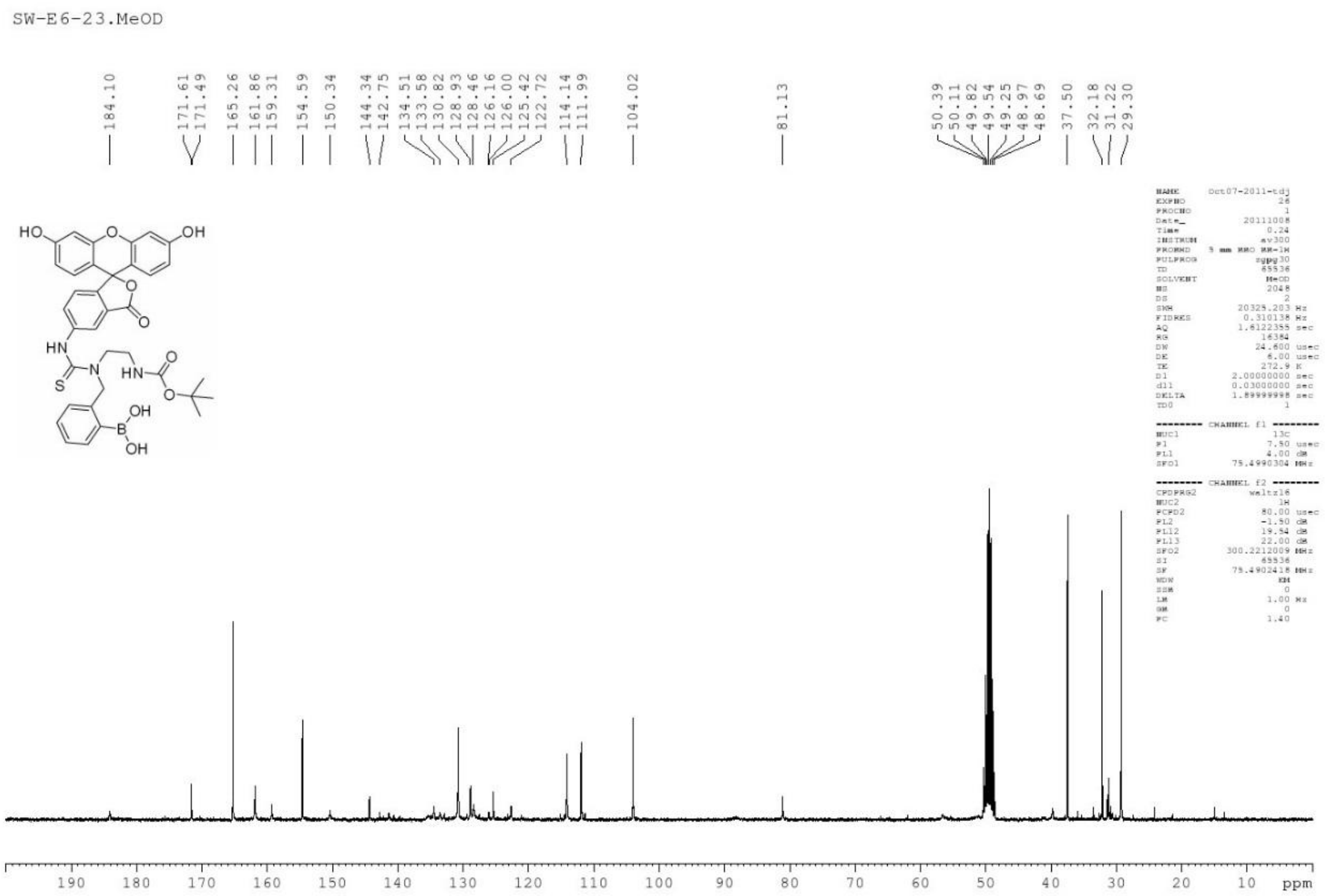

Figure S1.4. $^{13}C\{^{1}H\}$ NMR spectrum of **2**, in methanol-d_4, 75.5 MHz, 298 K

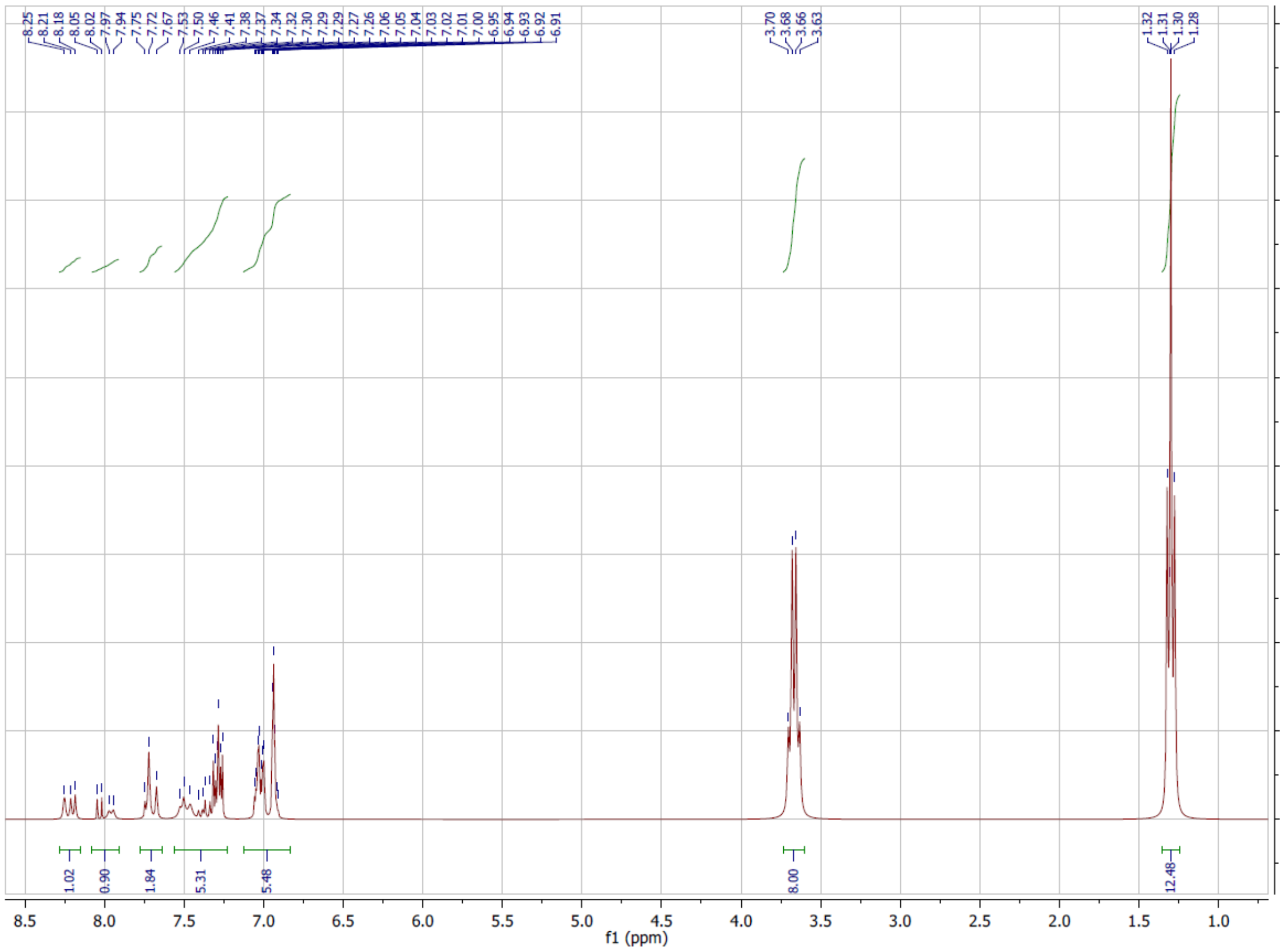

Figure S1.5. ^{1}H NMR spectrum of **3**, in methanol-d_4, 300 MHz, 298 K

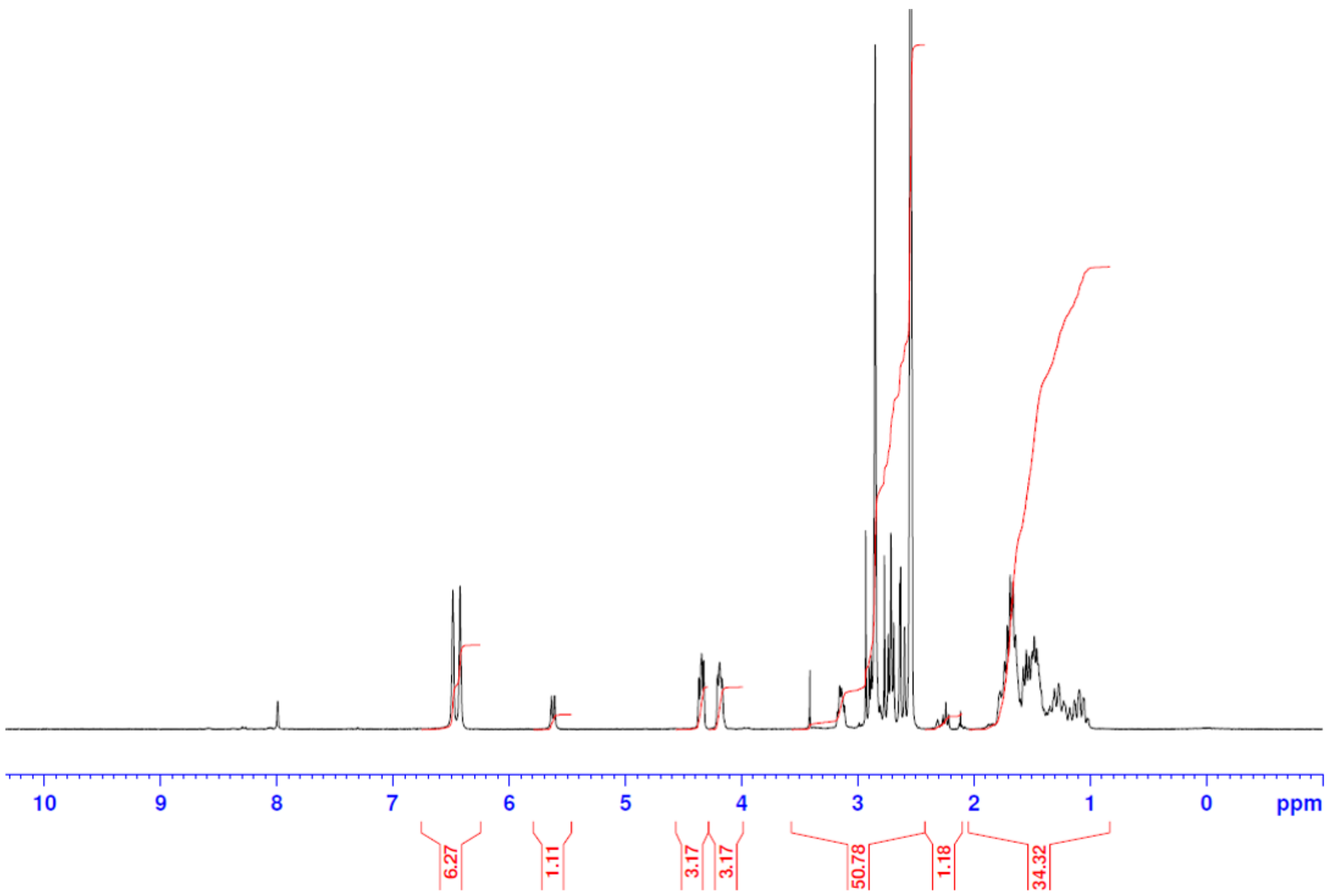

(a)

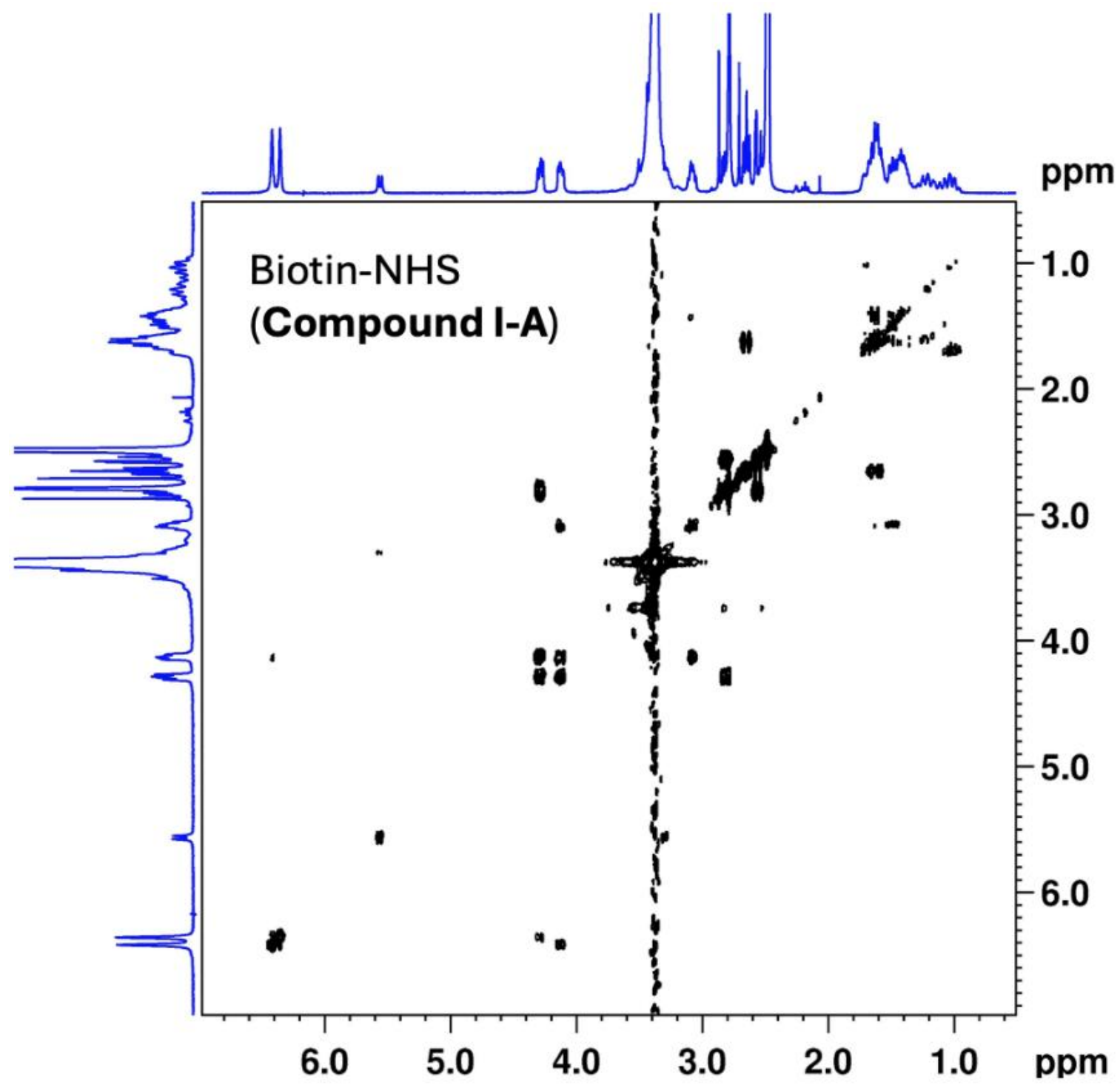

(b)

Figure S1.6. (a) ^{1}H NMR spectrum of **I-A** (Biotin NHS) and (b) corresponding ^{1}H-^{1}H COSY in DMSO-d_6, 300 MHz, 298 K

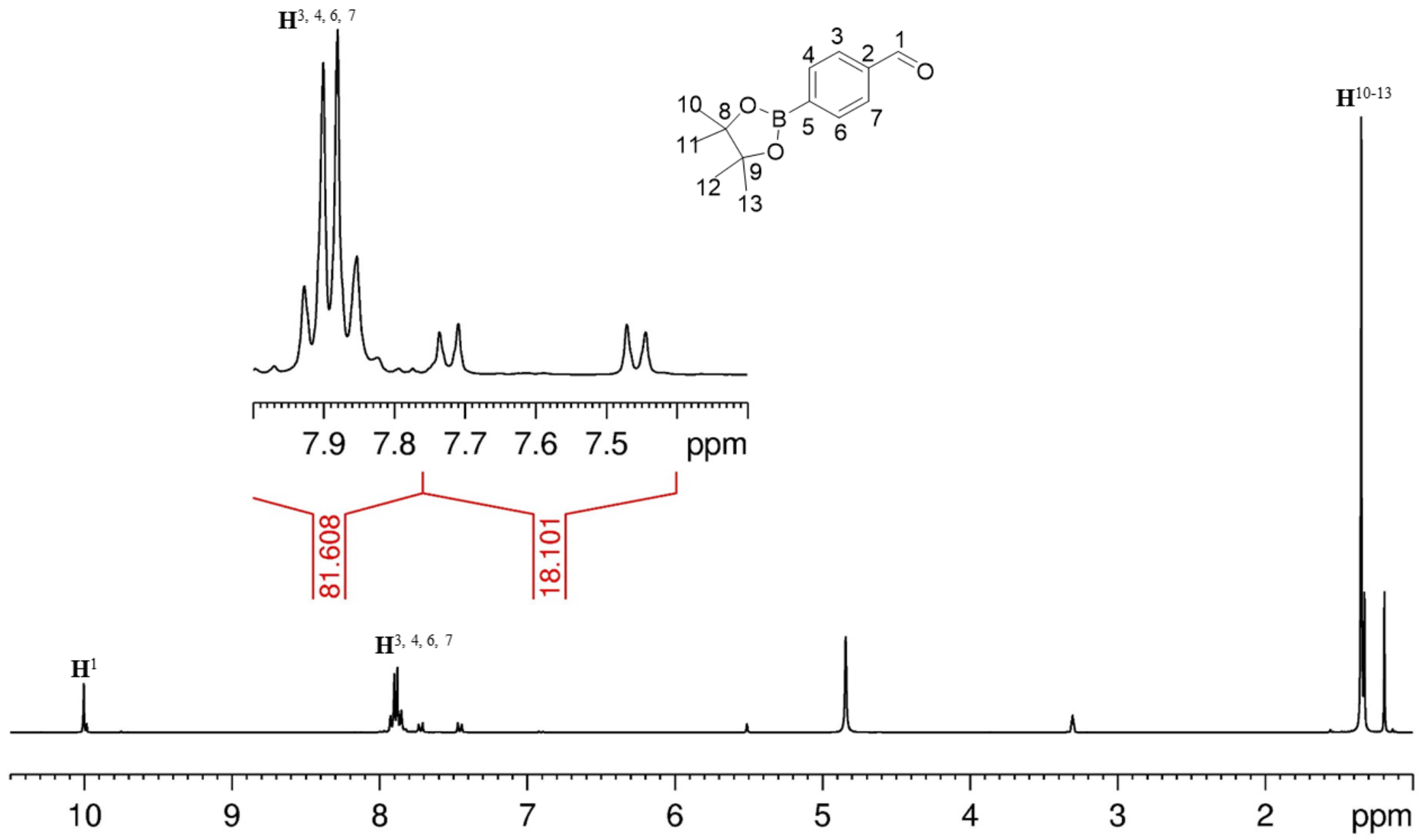

Figure S1.7. ^{1}H NMR spectrum of **III**, in $CDCl_3$, 300 MHz, 298 K

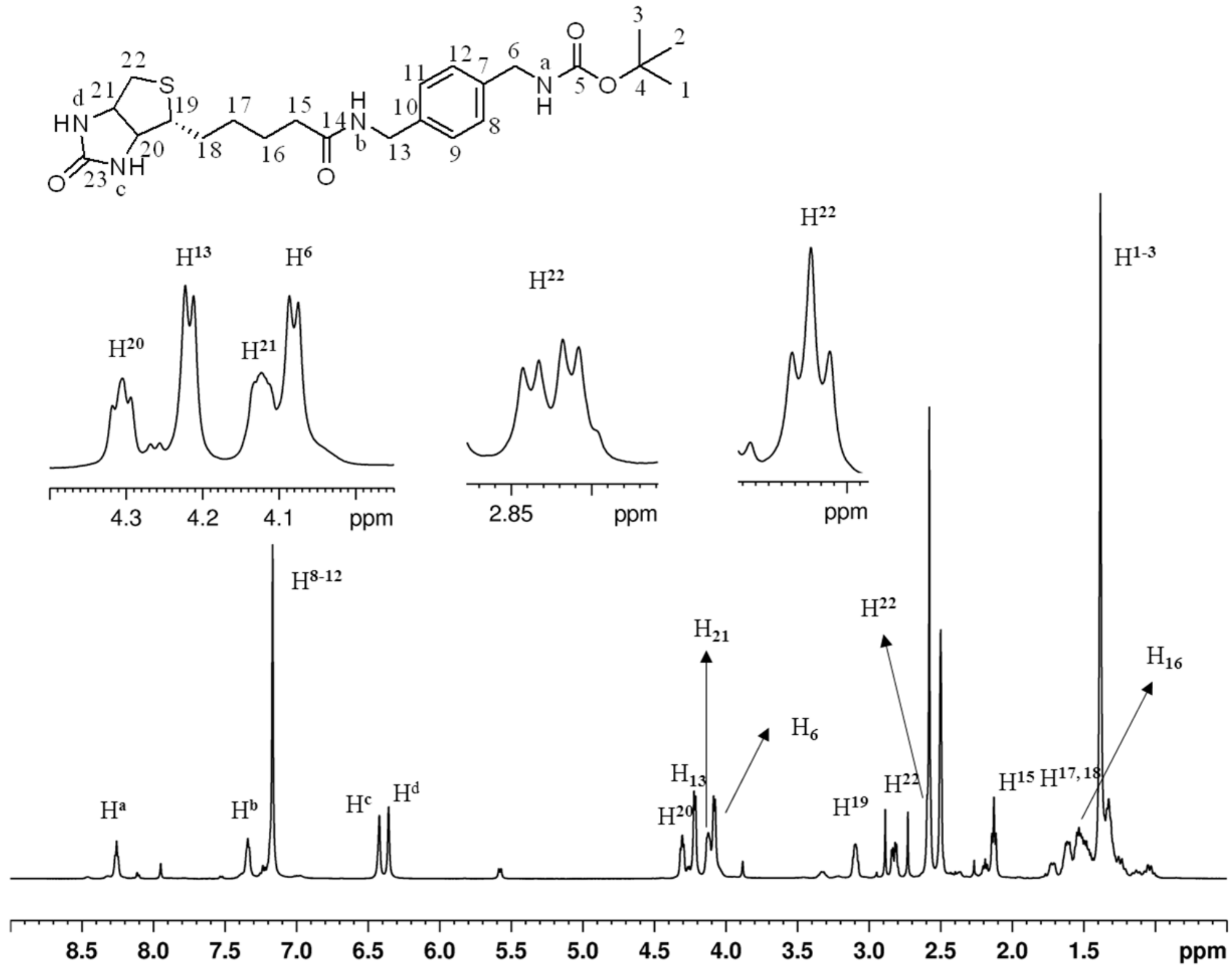

Figure S1.8. ^{1}H NMR spectrum of **IV**, in DMSO-d_6, 300 MHz, 298 K

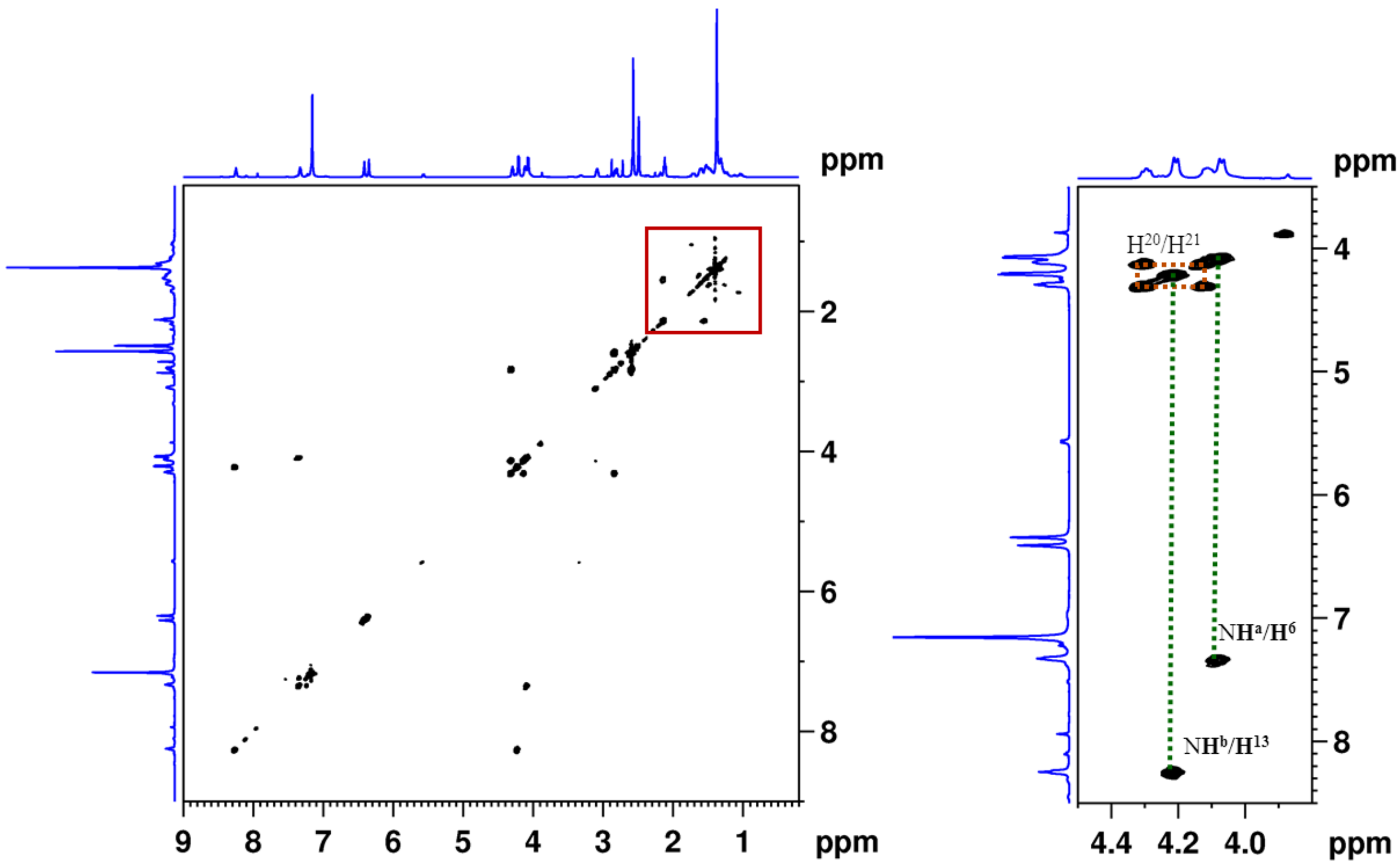

Figure S1.9. ^{1}H COSY NMR spectroscopy of compound **IV** with corresponding and correlation of N-H to the alkyl protons and N-H to α protons of Biotin functionality r (red), in DMSO-d_6, 300 MHz, 298 K

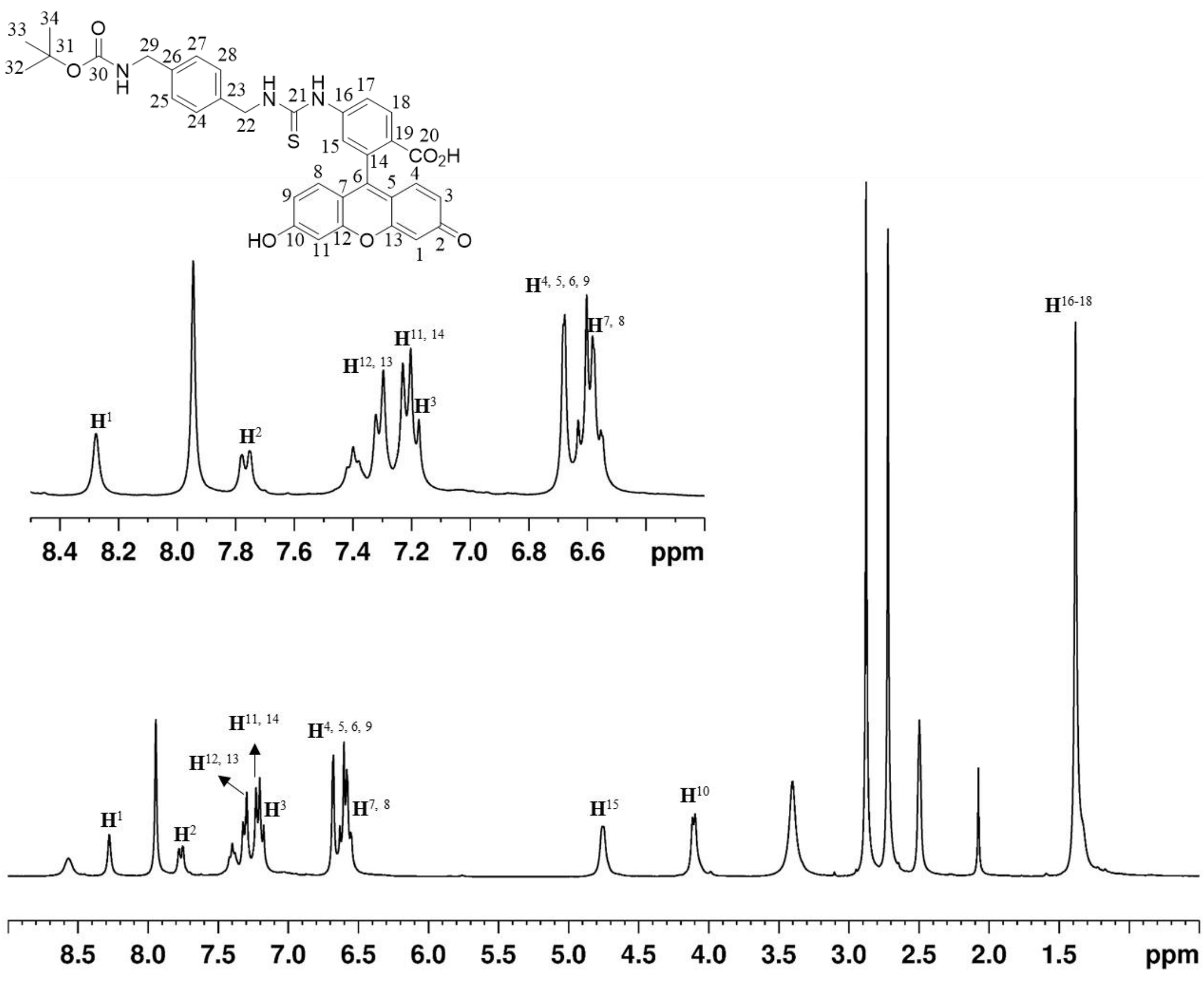

Figure S1.10. ^{1}H NMR spectrum of **V**, in DMSO-d_6, 300 MHz, 298 K

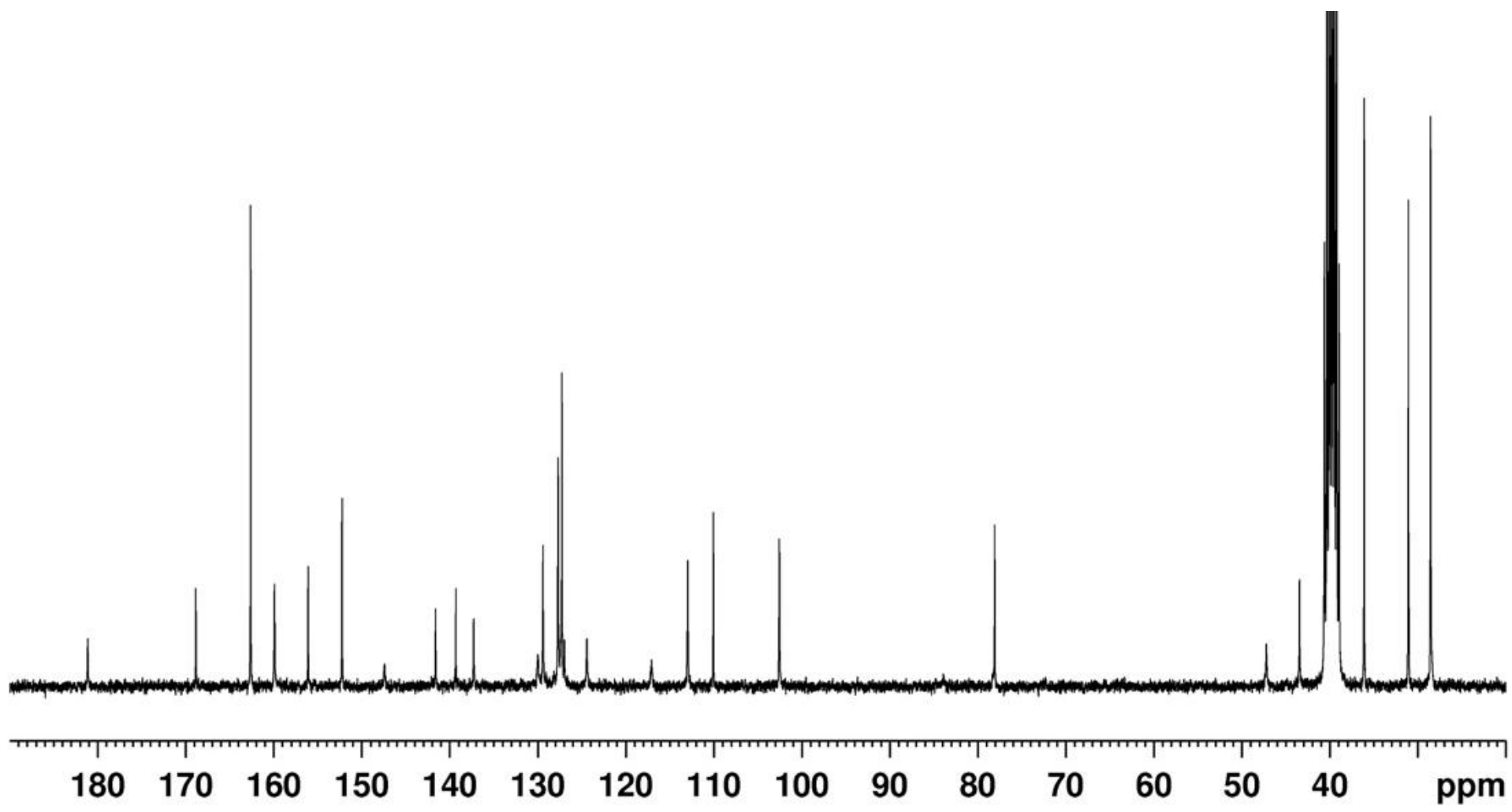

Figure S1.11. $^{13}C\{^{1}H\}$ NMR spectrum of **V**, in DMSO-d_6, 300 MHz, 298 K

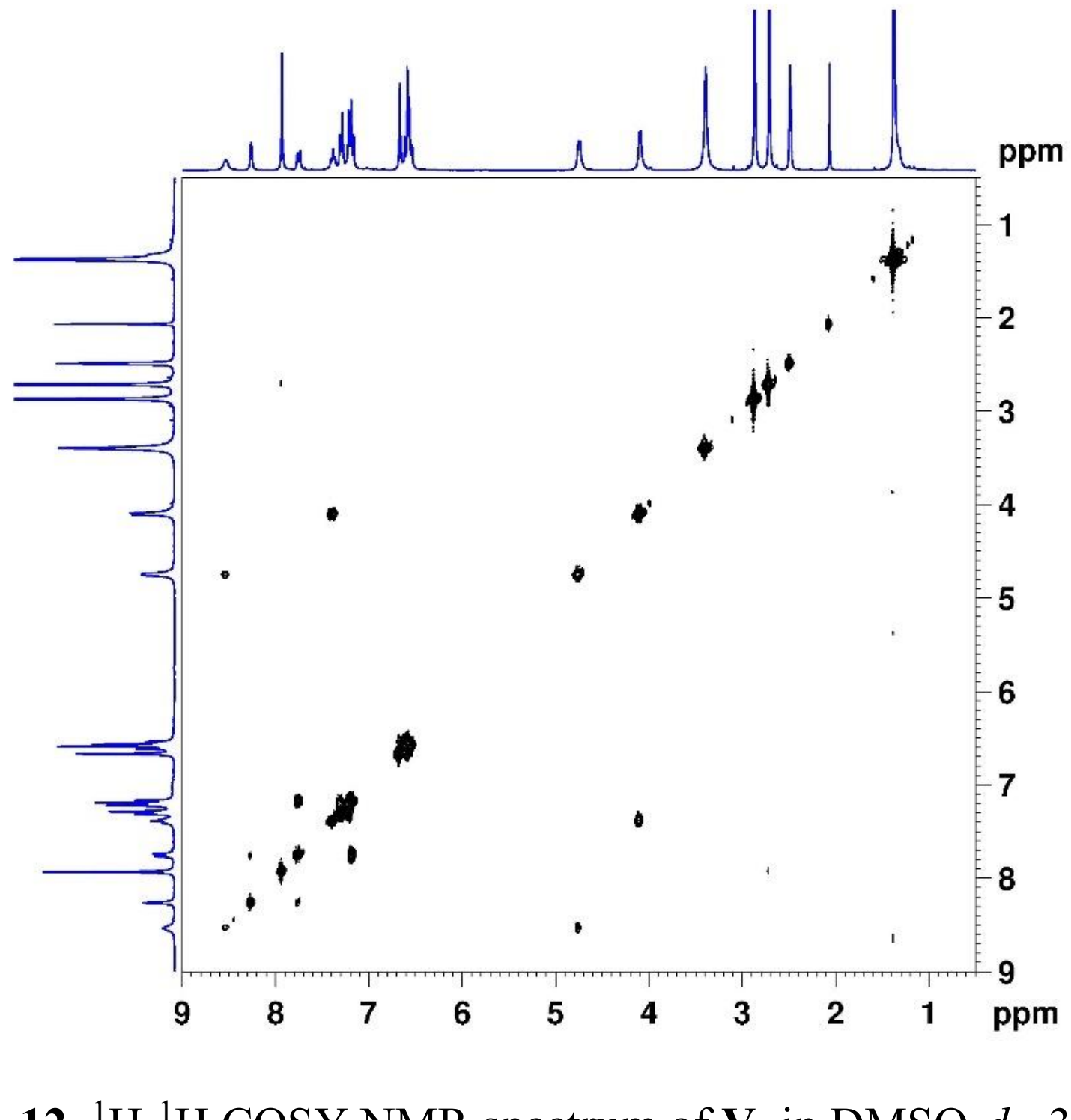

Figure S1.12. ^{1}H-^{1}H COSY NMR spectrum of **V**, in DMSO-d_6, 300 MHz, 298 K

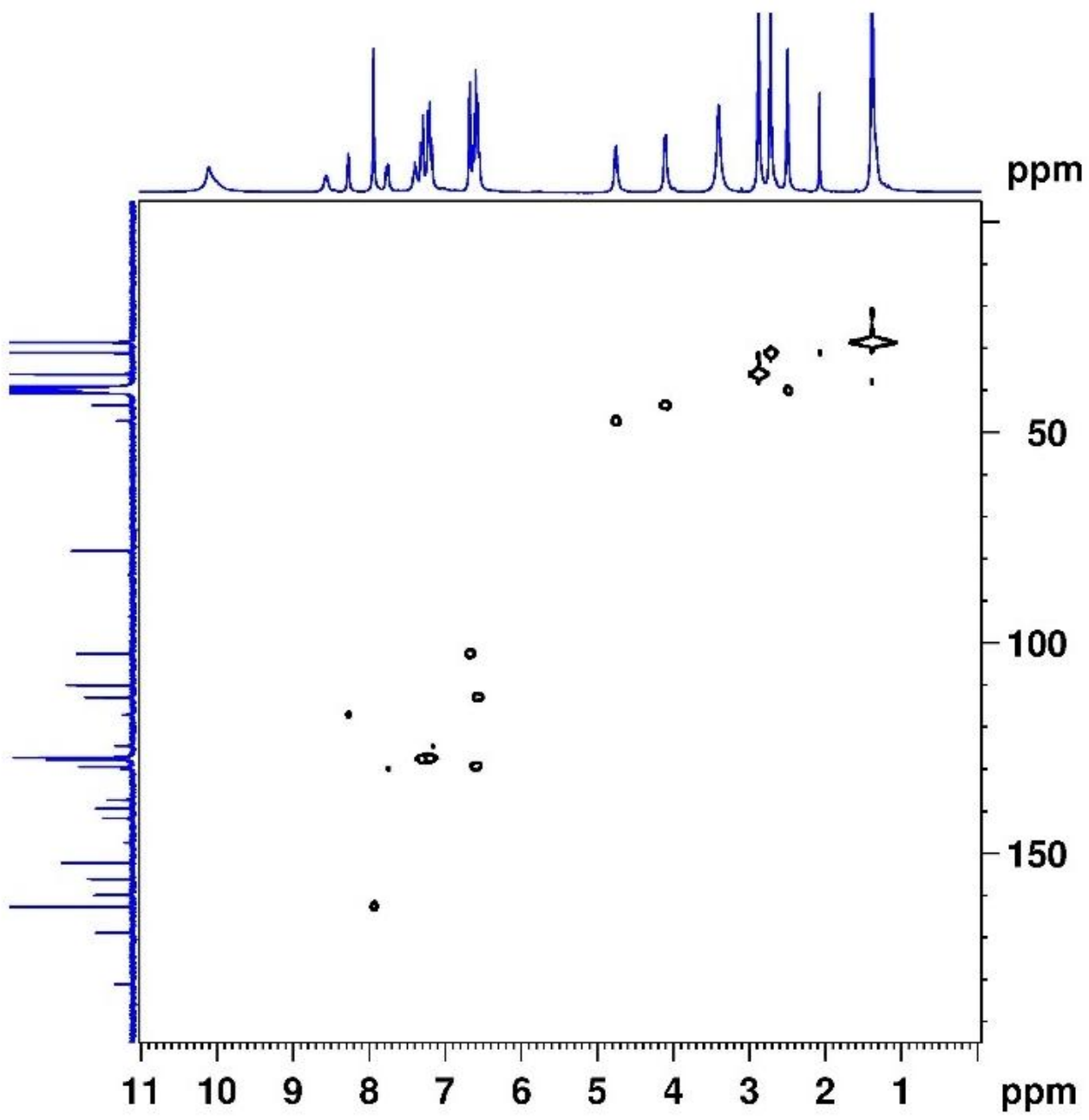

Figure S1.13. ^{1}H-^{13}C HMBC NMR spectrum of **V**, in DMSO-d_6, 300 MHz, 298 K

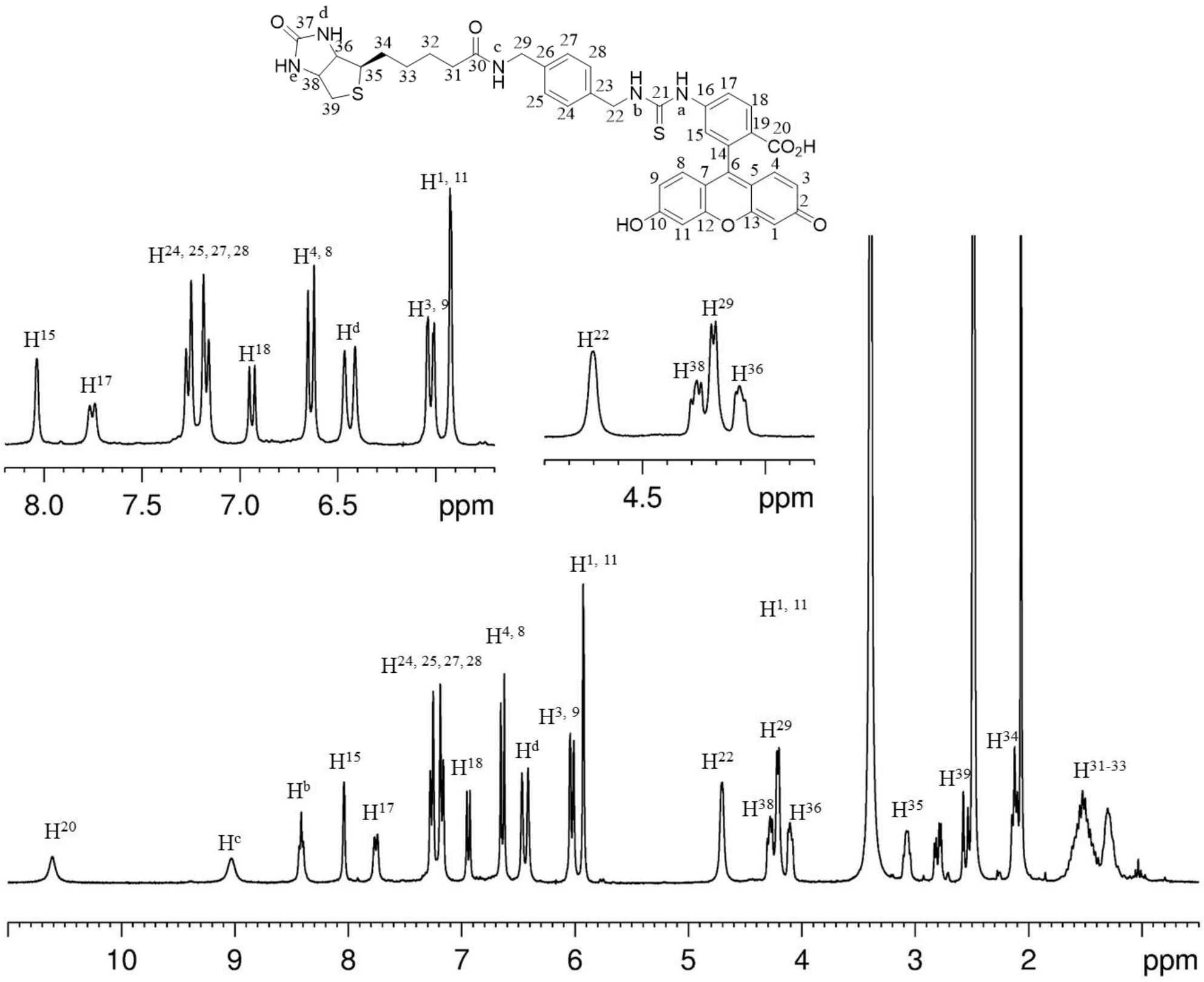

Figure S1.14. ^{1}H NMR spectrum of **4**, in methanol-d_4, 300 MHz, 298 K

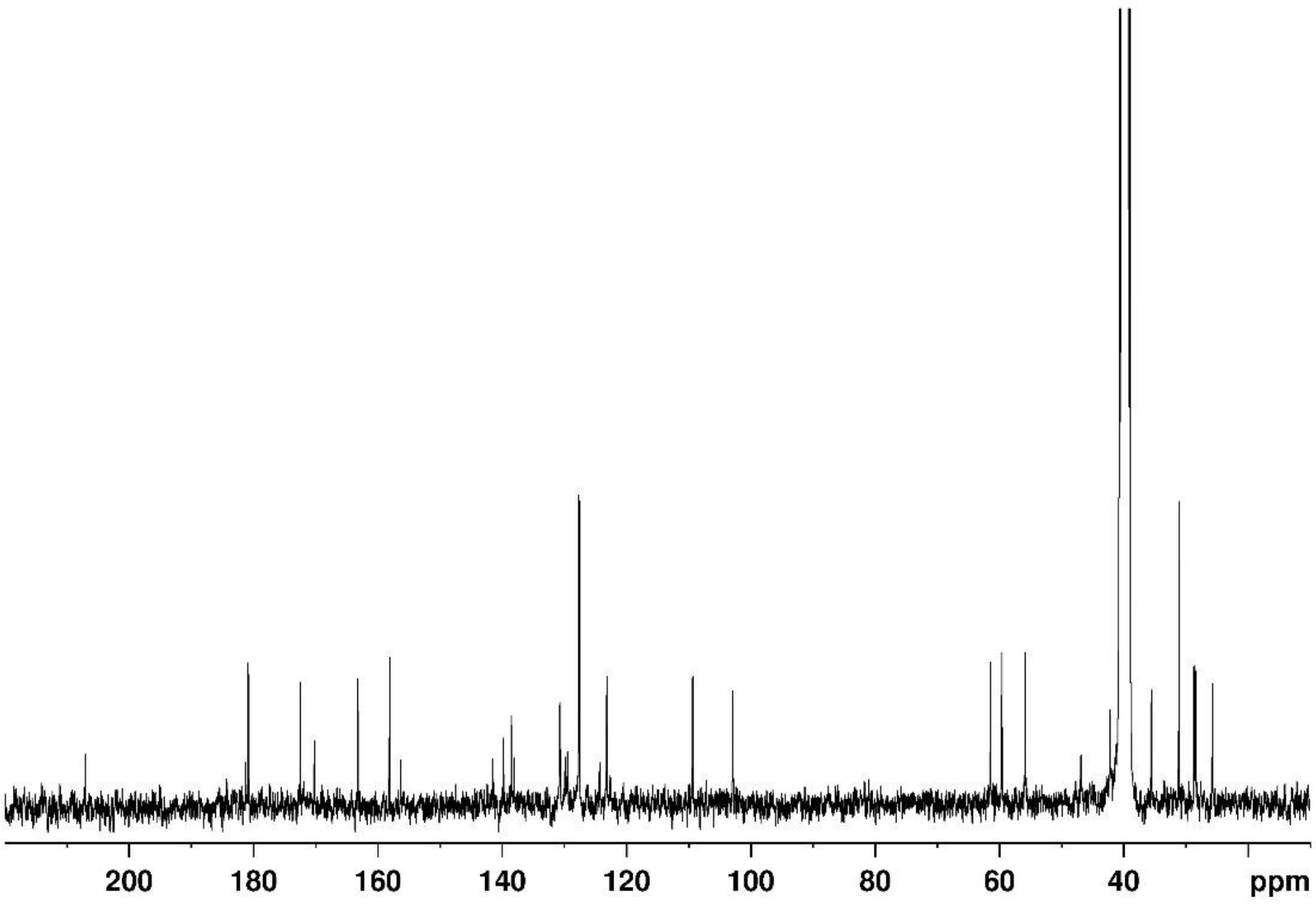

Figure S1.15. $^{13}C\{^{1}H\}$ NMR spectrum of **4**, in methanol-d_4,

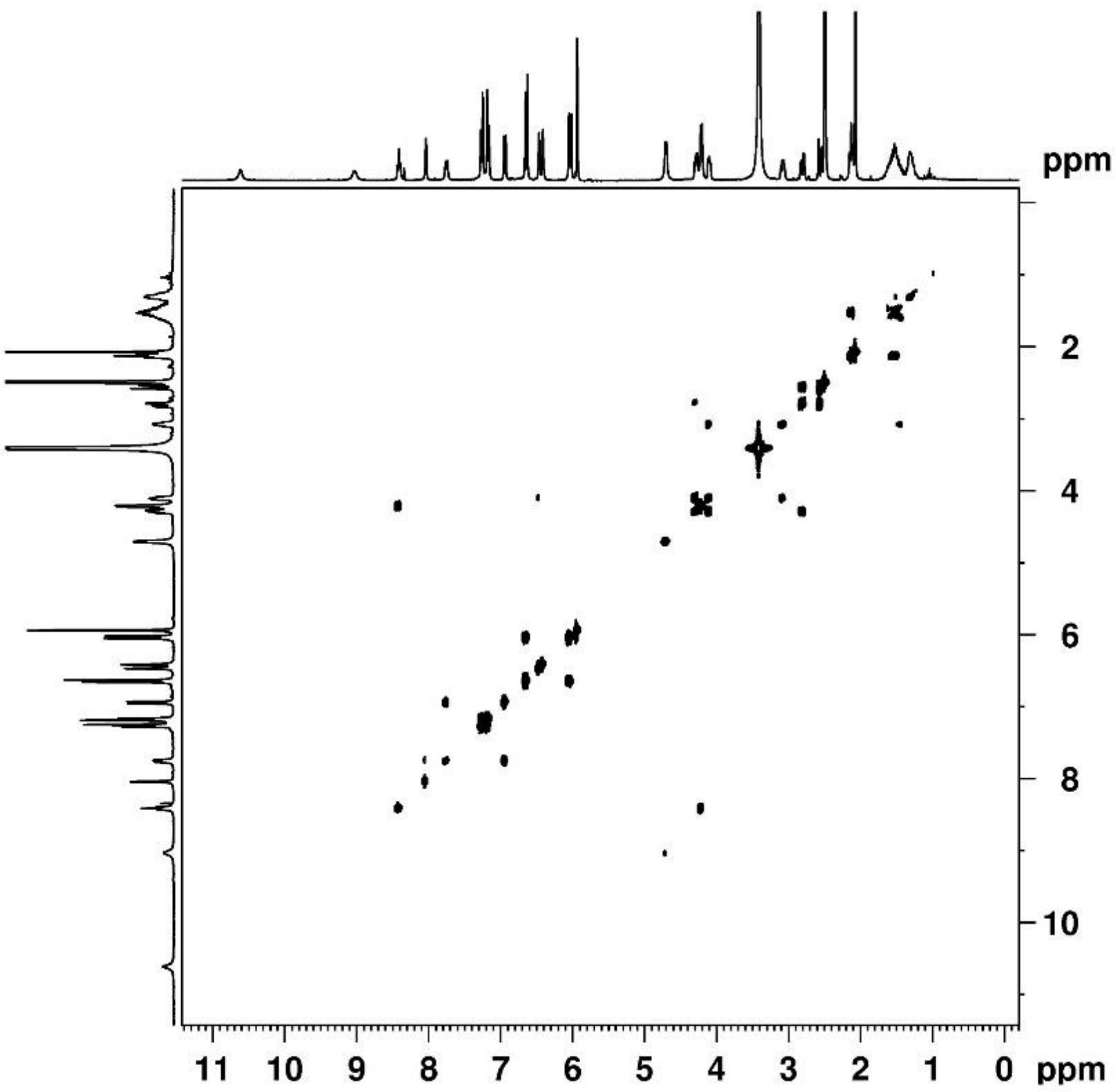

Figure S1.16. ^{1}H-^{1}H COSY NMR spectrum of **4**, in methanol-d_4, 300 MHz, 298 K

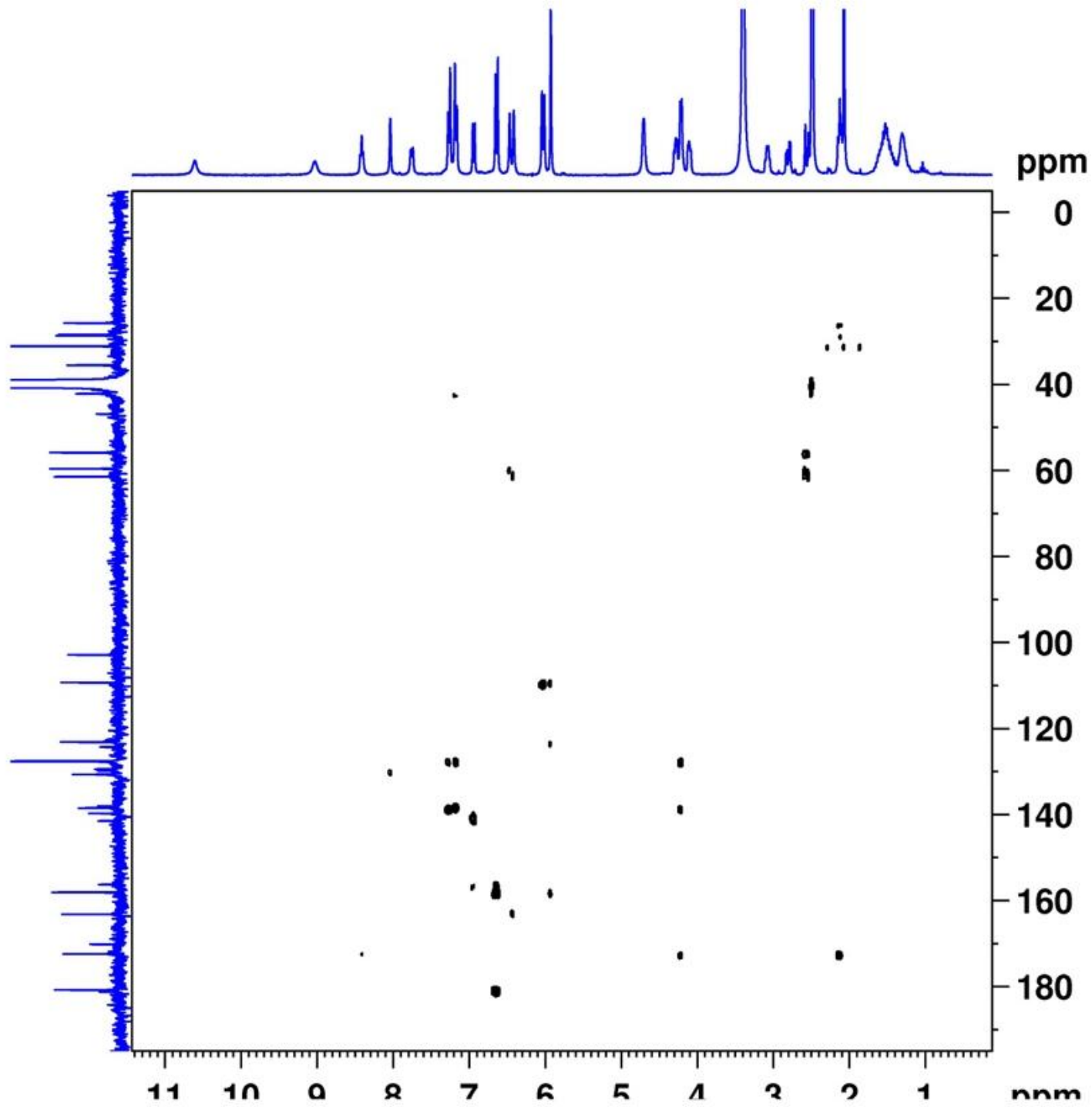

Figure S1.17. (a) ^{1}H-$^{13}C\{^{1}H\}$ HMBC NMR spectroscopy of **4**, in methanol-d_4, 298 K

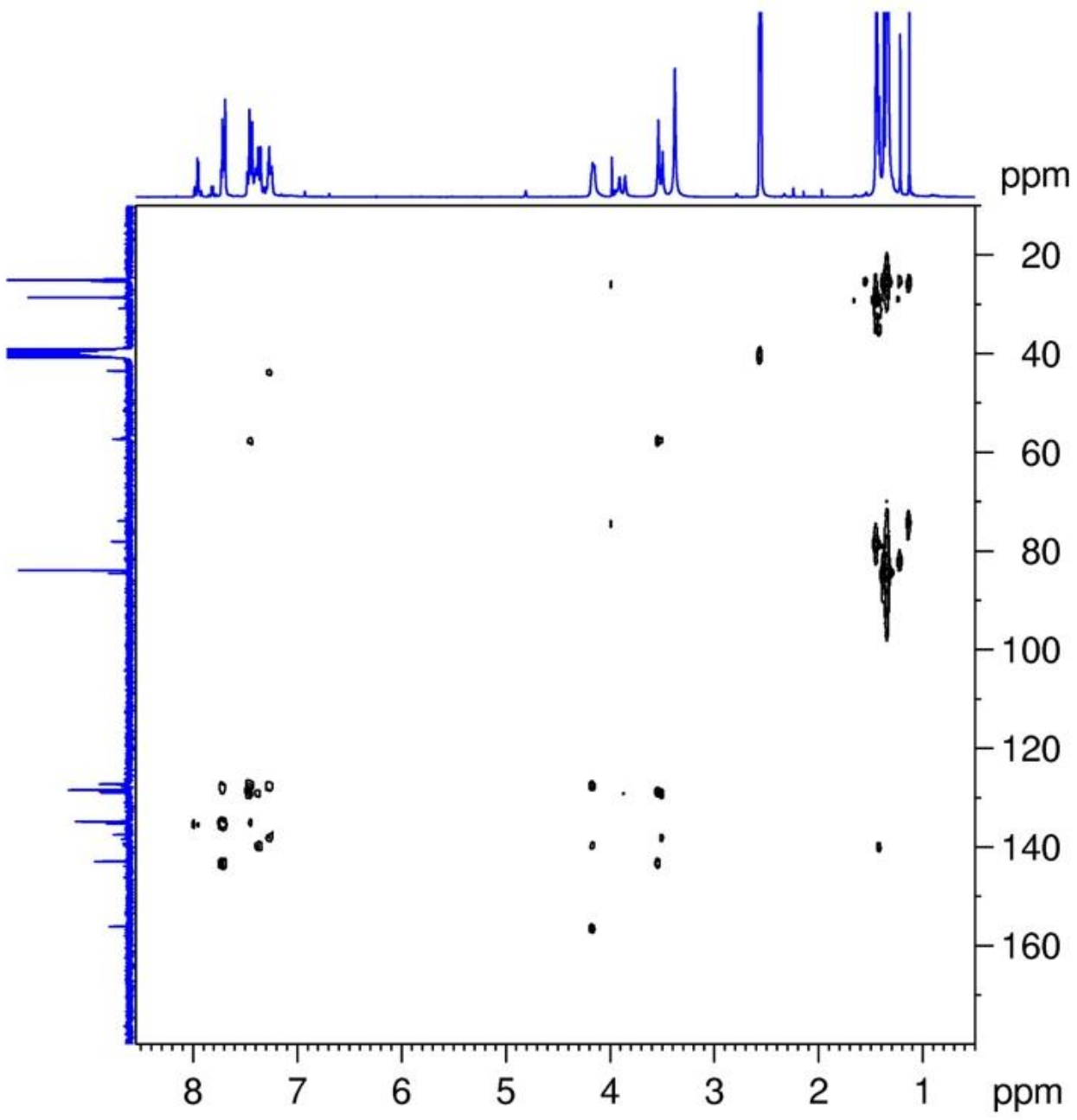

(a)

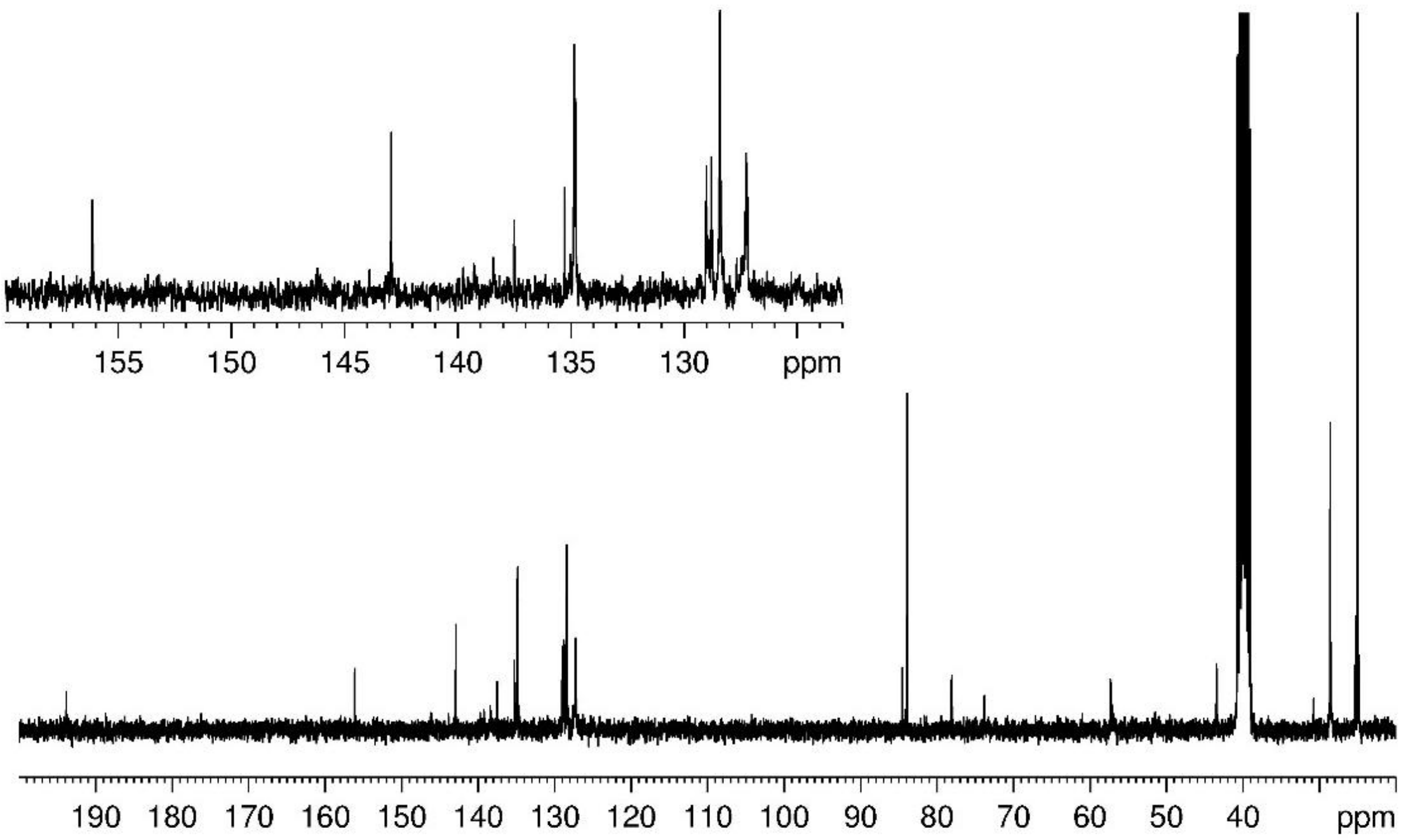

(b)

Figure S1.18. (a) ^{1}H-$^{13}C\{^{1}H\}$ HMBC and (b) $^{13}C\{^{1}H\}$ NMR spectroscopy of **5**, in methanol-d_4, 298 K NMR

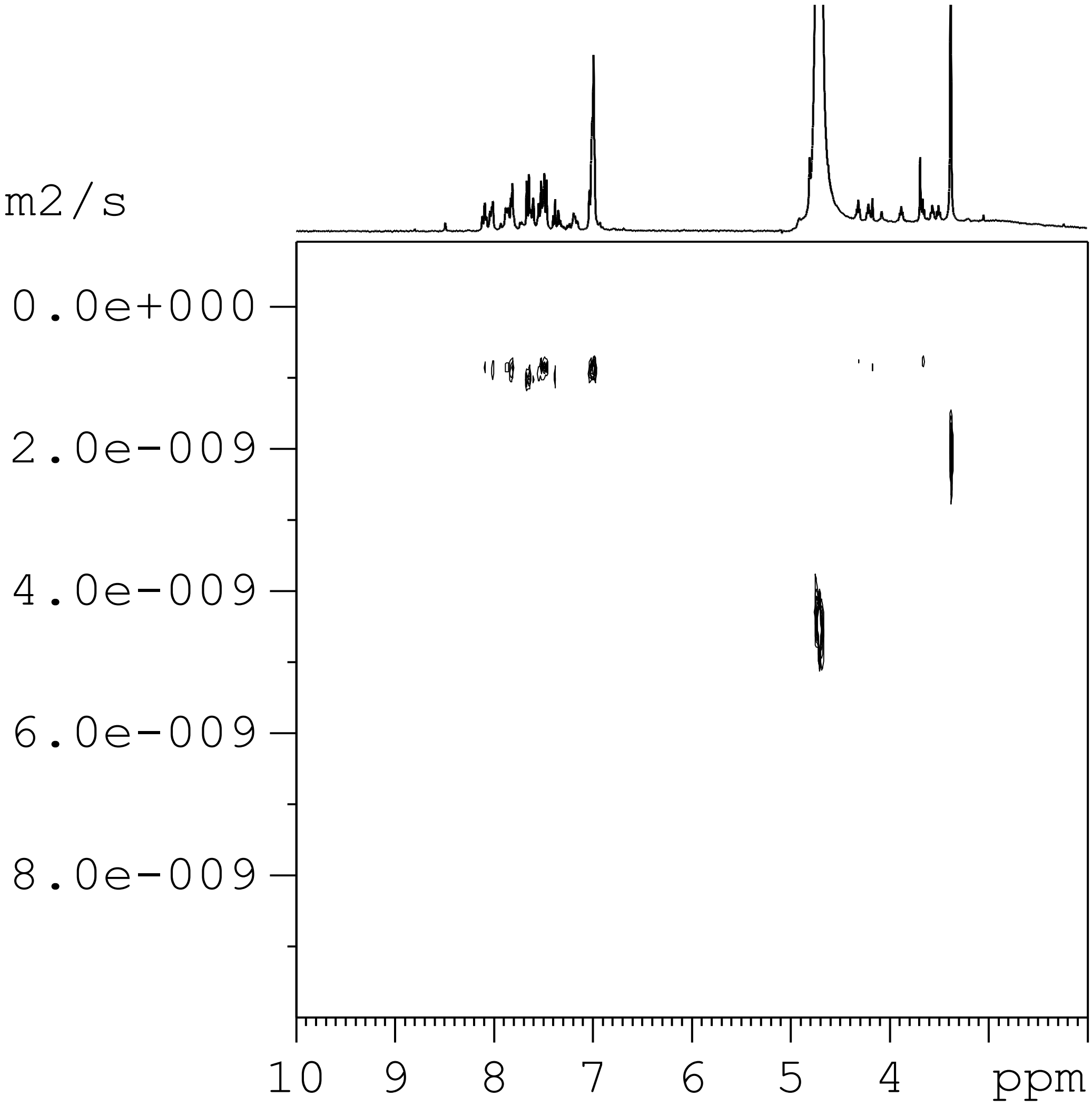

Figure S1.19. DOSY of Compound **5** in methanol-d_4, 300 MHz, 333 K.

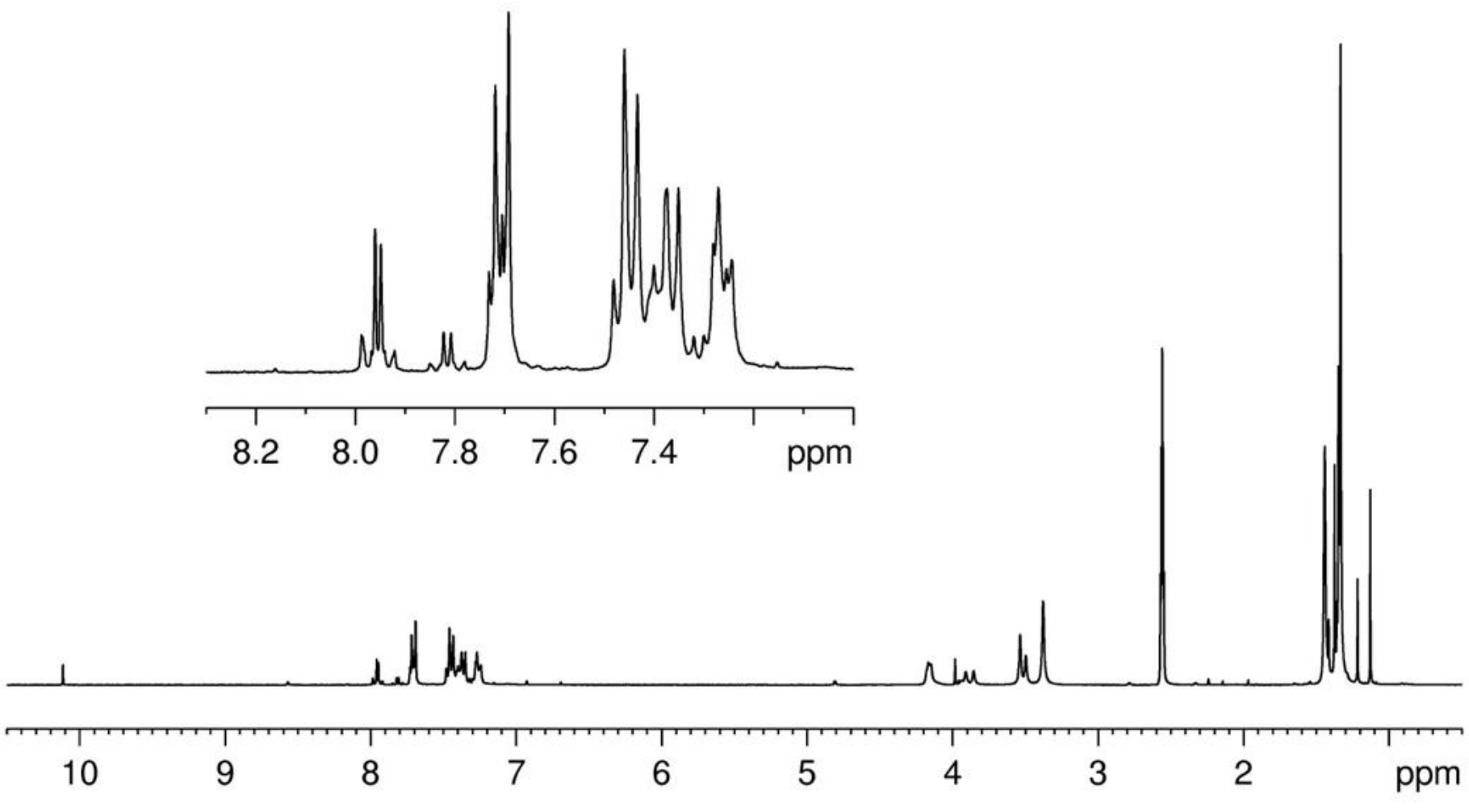

Figure S1.20. ^{1}H NMR spectrum of **VI**, in methanol-d_4, 300 MHz, 298 K

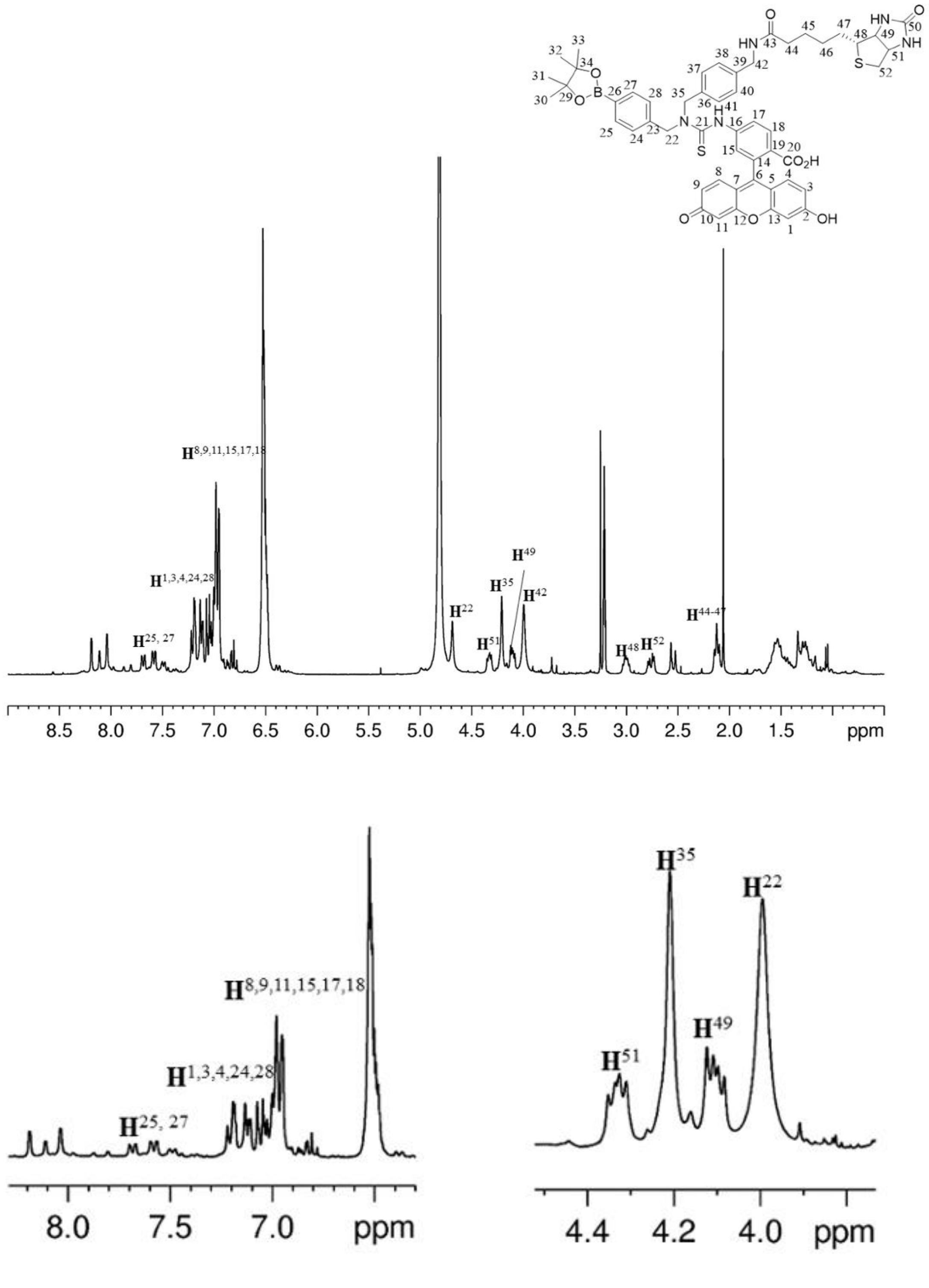

Figure S1.21. ^{1}H NMR spectrum of Compound **6**, and corresponding expansions in methanol-d_4, 300 MHz, 298 K

Selected HPLC traces:

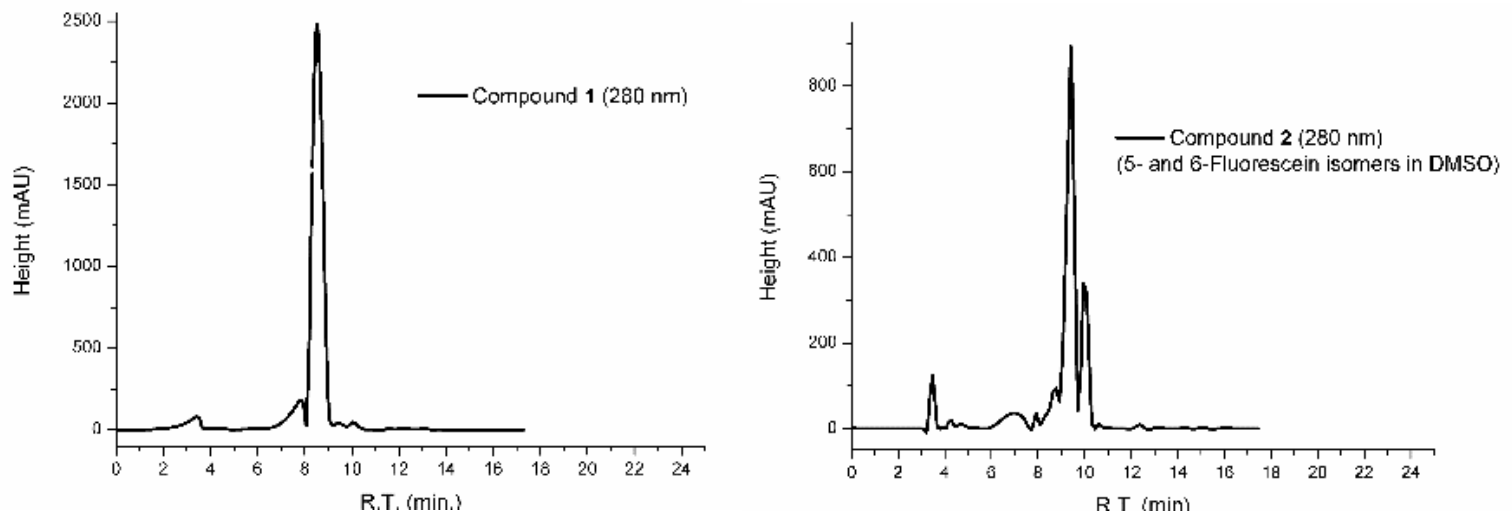

(a) **(b)**

Figure S1.22. Reverse phase HPLC trace and gradient method applied at the analysis of Compounds **1** (a) and **2** (b), as mixtures of 5(6)-fluoresceine isomers. Samples were injected from DMSO:MeOH 1:1. Mobile phase: CH_3CN:H_2O 95:5 (0.1% TFA)

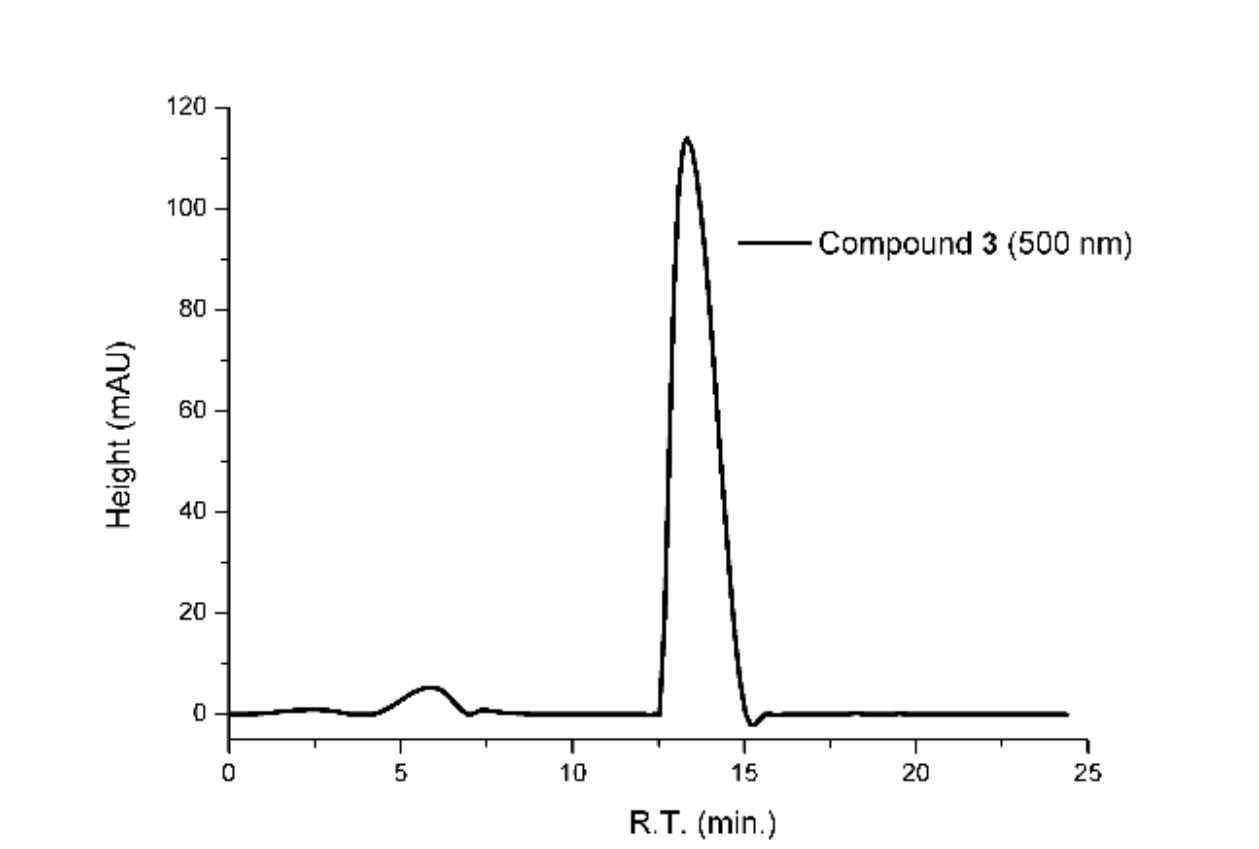

Figure S1.23. Reverse phase HPLC trace and gradient method applied at the analysis of Compound **3** (mixture of isomers, sample injected from DMSO, mobile phase: CH_3CN:H_2O 95:5 and 0.1% TFA)

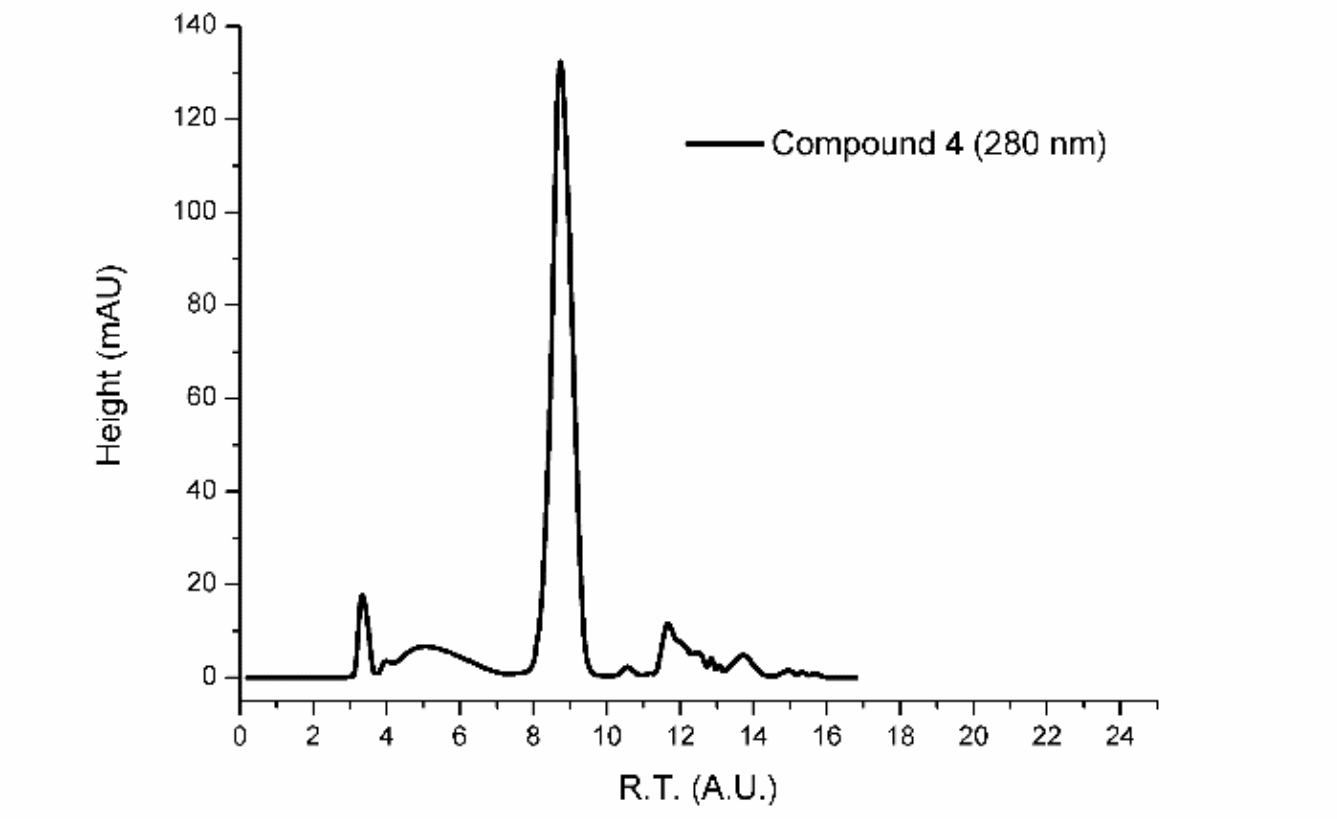

Figure S1.24. Reverse phase HPLC trace and gradient method applied at the analysis of Compound **4** (mixture of 5(6)-fluorescein isomers, sample injected from DMSO:MeOH 1:1, mobile phase: CH_3CN:H_2O 95:5 and 0.1% TFA).

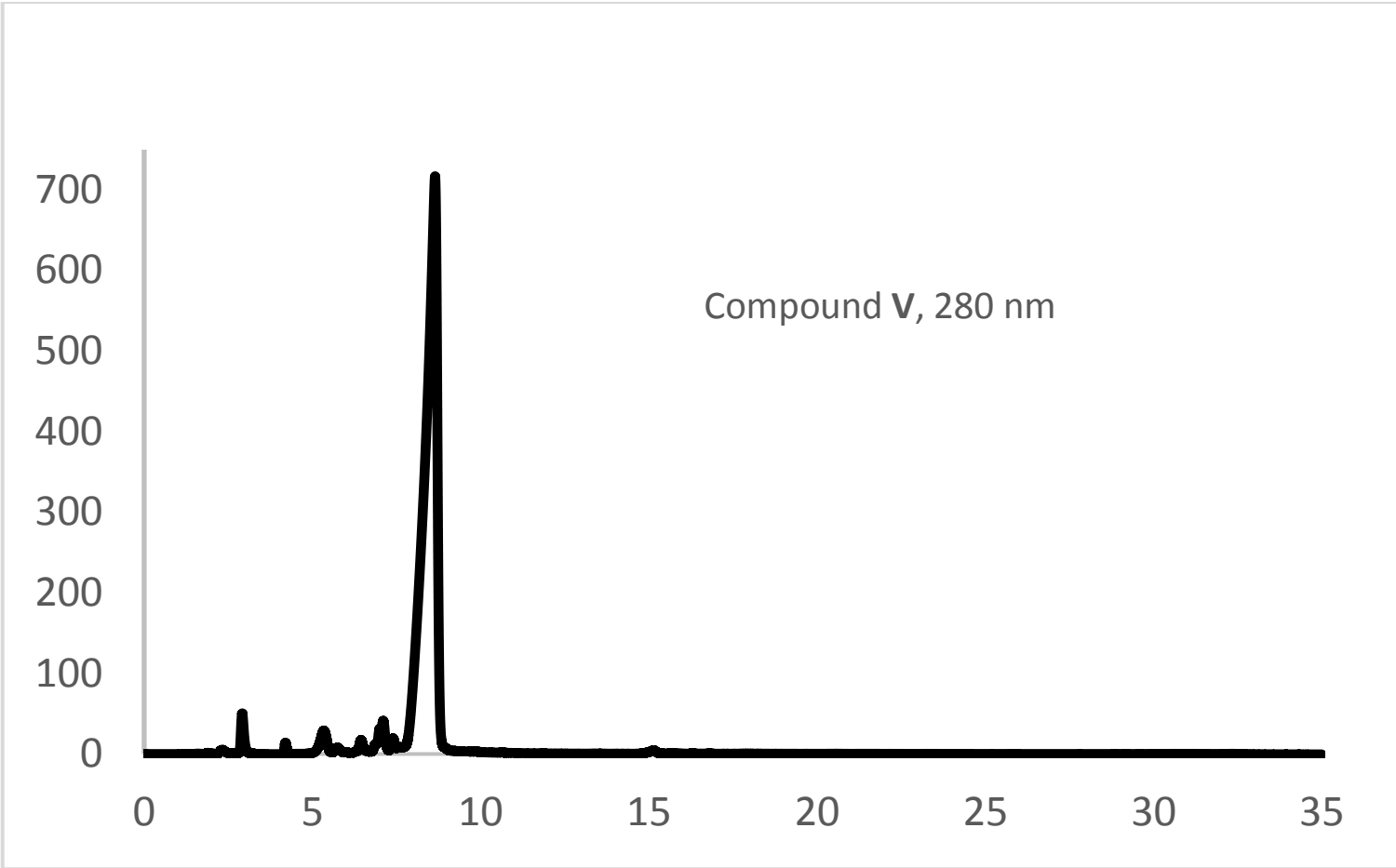

Figure S1.25. Reverse phase HPLC trace and gradient method applied at the analysis of Compound **V** (as a mixture of 5(6)-fluorescein isomers, sample injected from DMSO:MeOH 1:1, mobile phase: CH_3CN:H_2O 95:5 and 0.1% TFA).

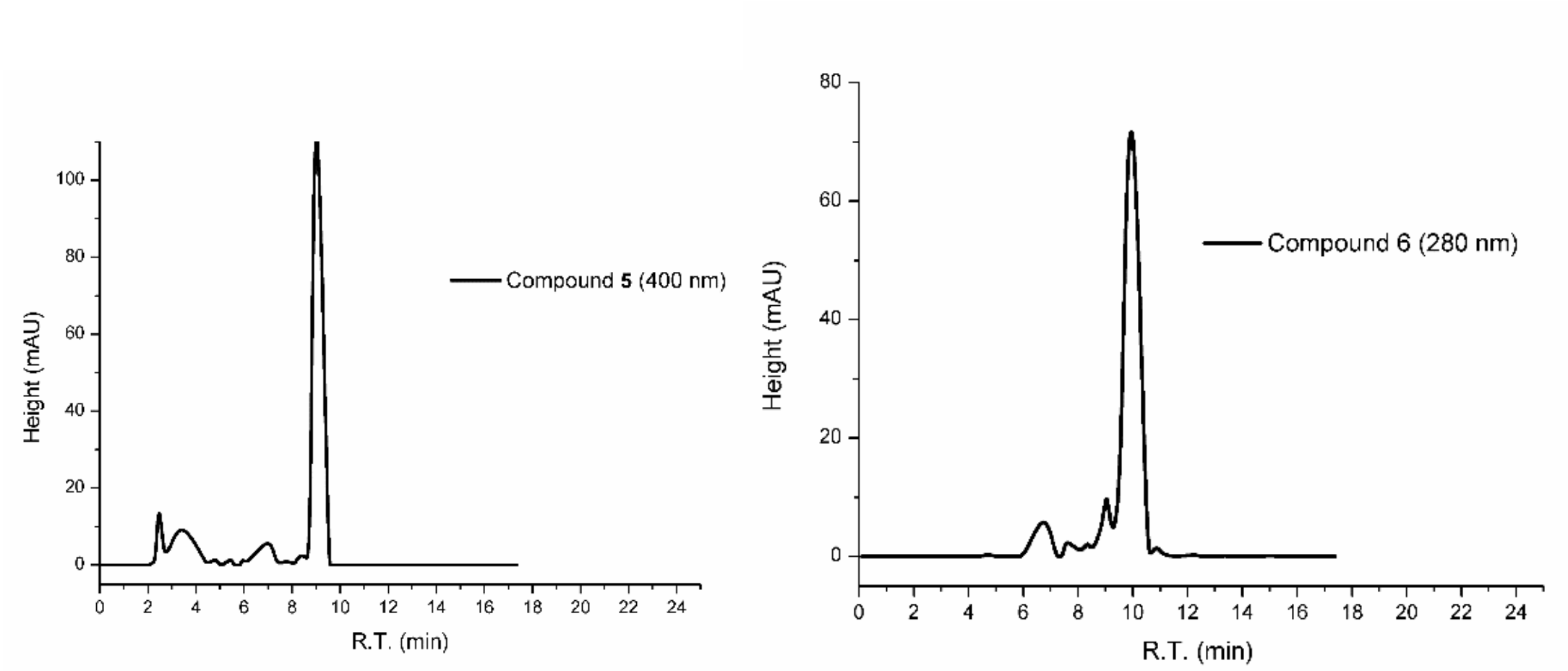

Figure S1.26. Overview and comparison of the reverse phase HPLC behaviour for compounds **5** and **6** featuring broad bands due to fluorescein isomerism, boronic acid deprotection of pinacol group and protonation.

Selected Mass Spectrometry:

(1) Mass spectrometry of selected intermediates and precursors:

Ionisation Mode positive electrospray (ESI)

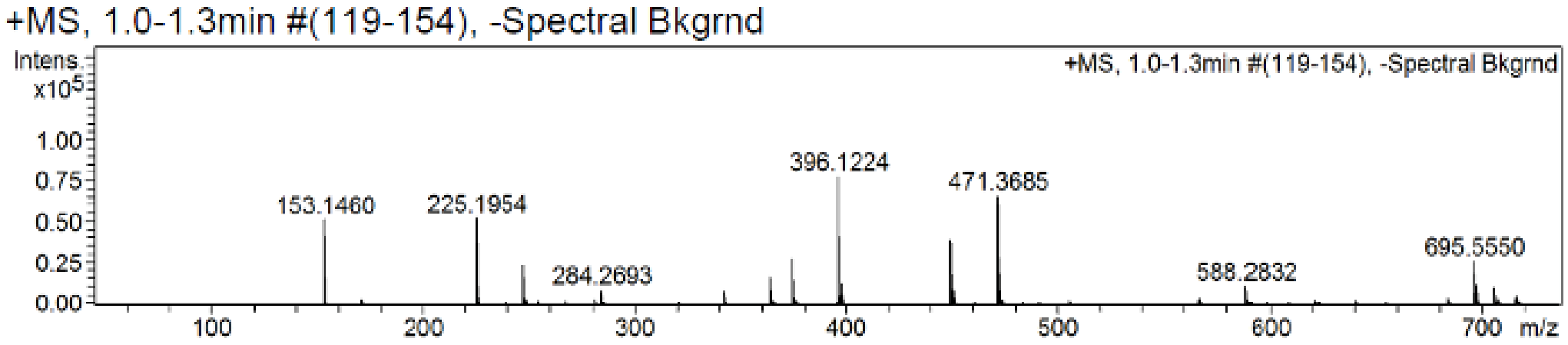

#	m/z	I	I %	Area	S/N
1	153.1460	51367	66.3	459	21400.0
2	225.1954	52188	67.3	1615	14192.7
3	247.1770	23528	30.3	865	5555.6
4	364.0940	16362	21.1	848	1919.0
5	374.1374	27713	35.7	1512	2535.4
6	396.1224	77523	100.0	3537	5683.1
7	449.3863	39169	50.5	2305	4592.8
8	471.3685	65447	84.4	3822	5622.6
9	472.3719	20257	26.1	1289	1719.3
10	695.5550	26226	33.8	2493	1882.5

Biotin-NHS (**Compound I-A**)

Generate Molecular Formula Parameters

Charge	Tolerance	SearchRadius	H/C Ratio min.	H/C Ratio max.	Electron Conf.	Nitrogen Rule	sigma limit
positive	10 ppm	0.05 m/z	0	3	both	true	0.05

Expected Formula C14 H19 N3 O5 S **Adduct(s):** H, Na

#	meas. m/z	theo. m/z	Err[ppm]	Sigma	Formula
1	342.1097	342.112366	6.10	0.0165	C 14 H 20 N 3 O 5 S 1
1	364.0940	364.094311	-0.70	0.0100	C 14 H 19 N 3 Na 1 O 5 S 1

(a)

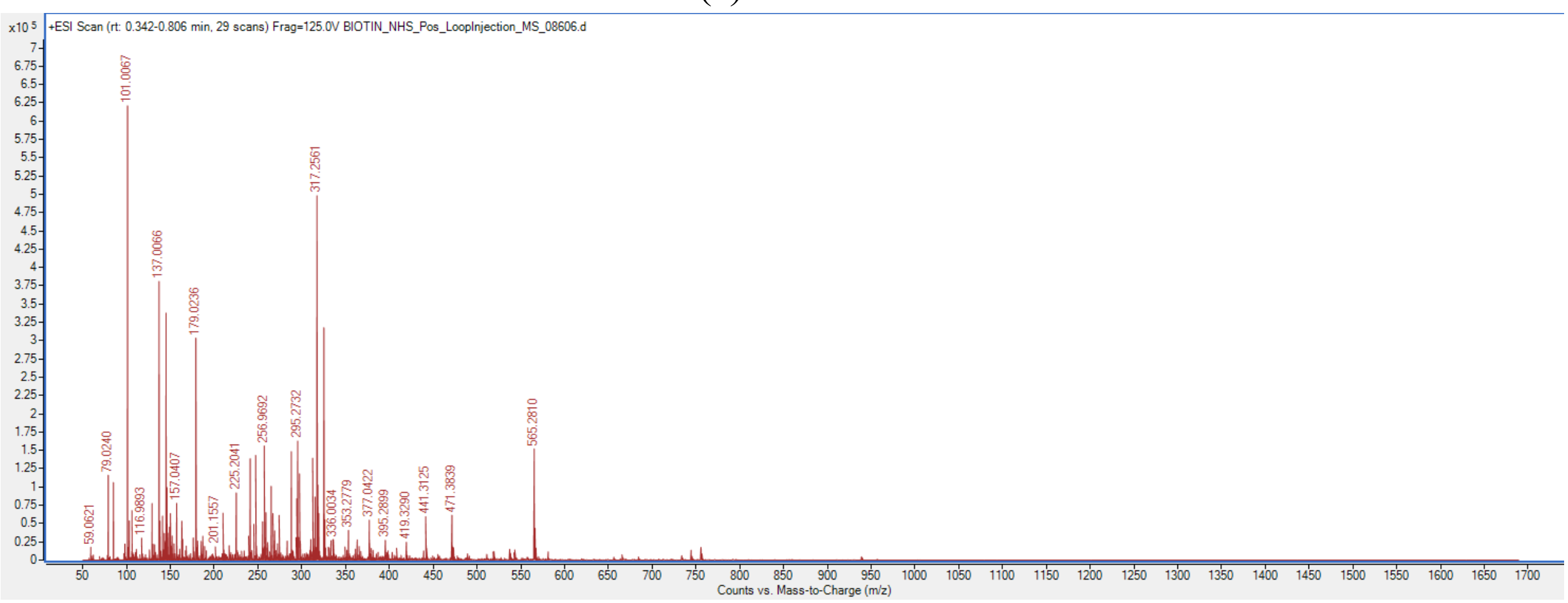

(b)

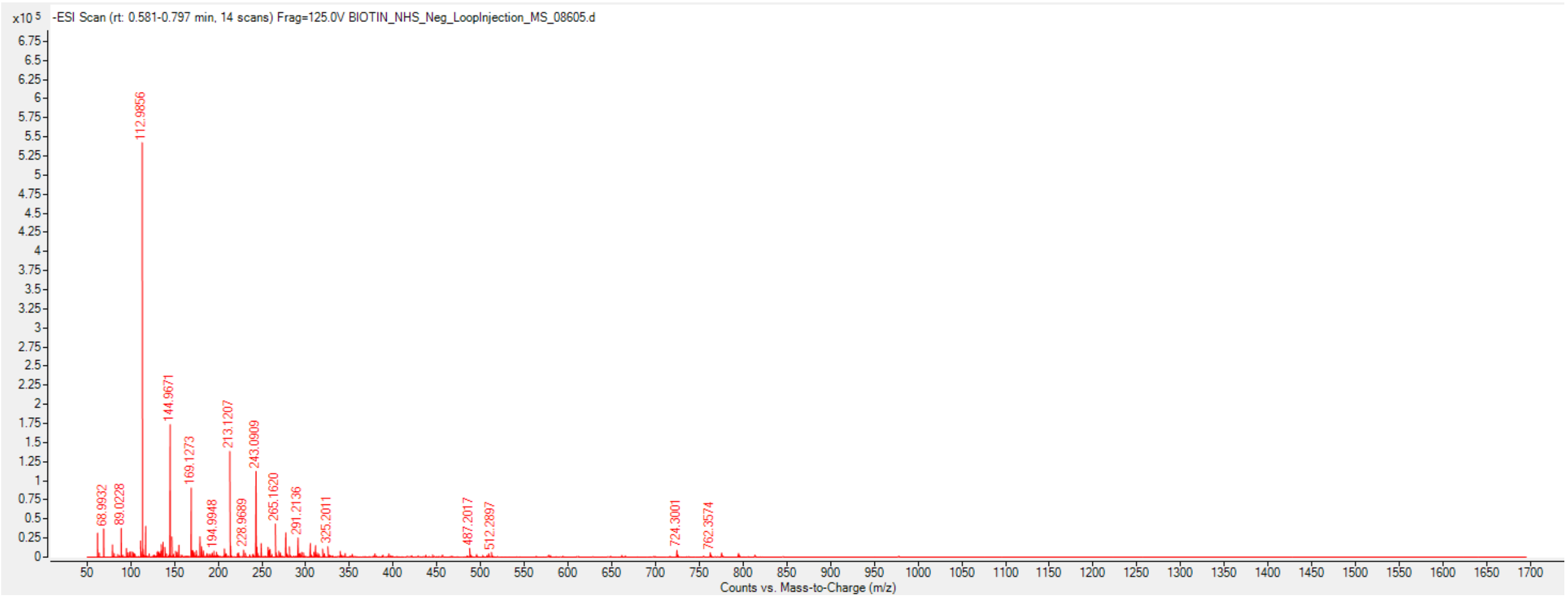

(c)

Figure S1.27. The ESI$^+$ (a and b) and ESI$^-$ (c) mass spectrometry analysis of Compound **I-A** (Biotin-NHS).

Ionisation Mode positive electrospray (ESI)

+MS, 1.0-1.3min #(119-154), -Spectral Bkgrnd

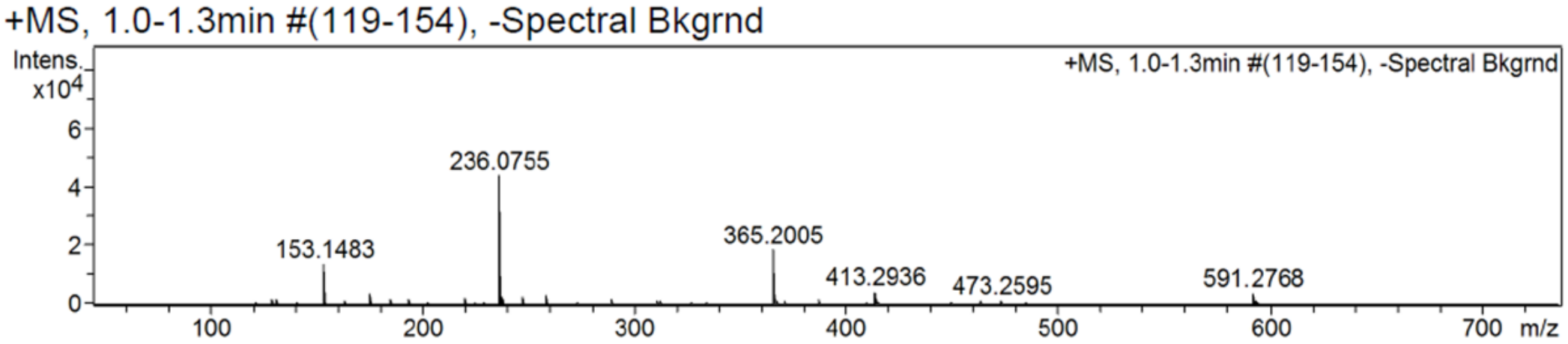

#	m/z	I	I %	Area	S/N
1	153.1483	13933	31.9	126	9331.1
2	175.1235	3509	8.0	31	1978.6
3	236.0755	43736	100.0	437	6517.2
4	237.0807	2801	6.4	48	407.7
5	247.1780	2715	6.2	109	320.7
6	258.0609	3271	7.5	46	390.7
7	365.2005	18781	42.9	863	2996.3
8	366.2029	3459	7.9	180	553.7
9	413.2936	4346	9.9	103	656.3
10	591.2768	3841	8.8	307	277.9

Compound I-5

Generate Molecular Formula Parameters

Charge	Tolerance	SearchRadius	H/C Ratio min.	H/C Ratio max.	Electron Conf.	Nitrogen Rule	sigma limit
positive	10 ppm	0.05 m/z	0	3	both	true	0.05

Expected Formula C16 H30 N4 O2 S **Adduct(s):** H, Na

#	meas. m/z	theo. m/z	Err[ppm]	Sigma	Formula
1	365.2005	365.198717	-6.30	0.0098	C 16 H 30 N 4 Na 1 O 2 S 1

Figure S1.28. The ESI$^+$ mass spectrometry analysis of intermediate Compound **I-5.**

Ionisation Mode positive electrospray (ESI)

+MS, 1.0-1.3min #(93-120), -Spectral Bkgrnd

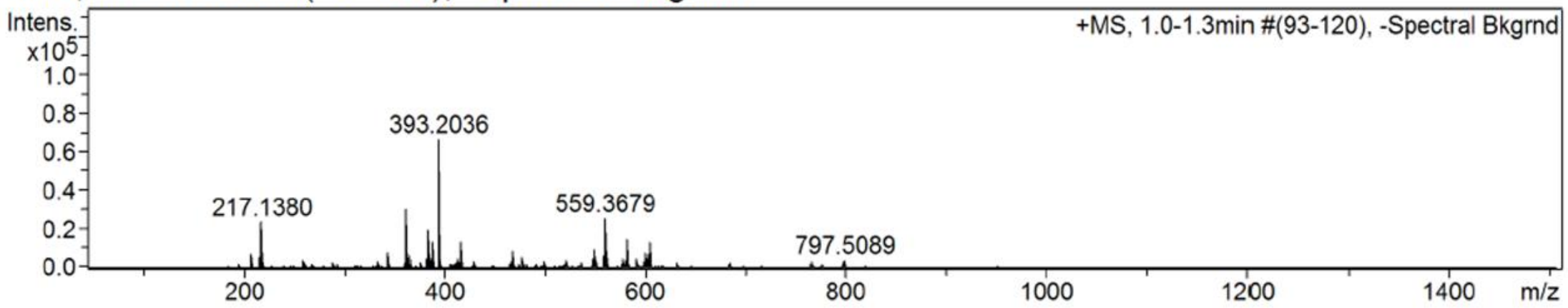

#	m/z	I	I %	Area	S/N
1	217.1380	24051	36.2	721	2256.4
2	361.2671	30086	45.3	1377	720.8
3	383.2474	19592	29.5	1113	395.1
4	387.2435	13513	20.4	764	269.6
5	393.2036	66380	100.0	2980	1359.4
6	394.1983	15354	23.1	1096	315.8
7	415.2390	12986	19.6	1012	294.6
8	559.3679	25929	39.1	1533	548.5
9	581.3600	15111	22.8	1488	291.6
10	603.3883	13562	20.4	1182	308.5

Compound II-5

Generate Molecular Formula Parameters

Charge	Tolerance	SearchRadius	H/C Ratio min.	H/C Ratio max.	Electron Conf.	Nitrogen Rule	sigma limit
positive	10 ppm	0.05 m/z	0	3	both	true	0.05

Expected Formula C29 H47 B N4 O4 S **Adduct(s):** H, Na

#	meas. m/z	theo. m/z	Err[ppm]	Sigma	Formula

Figure S1.29. The ESI$^+$ mass spectrometry analysis of intermediate Compound **II-5.**

Ionisation Mode positive electrospray (ESI)

+MS, 1.0-1.3min #(119-154), -Spectral Bkgrnd

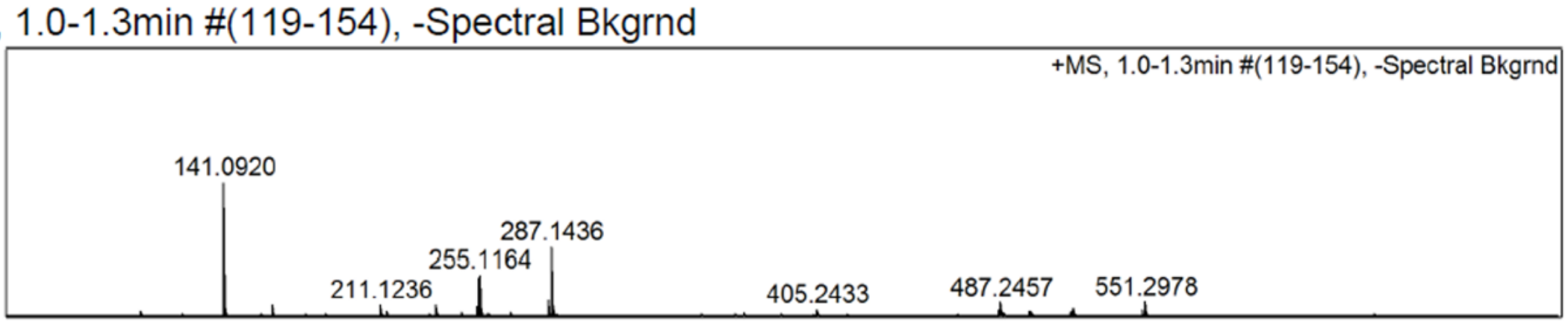

#	m/z	I	I %	Area	S/N
1	141.0920	196638	100.0	4232	15745.2
2	163.1256	15927	8.1	346	885.4
3	211.1236	17059	8.7	589	1383.9
4	236.0738	16497	8.4	324	709.0
5	255.1164	60353	30.7	2452	2029.4
6	286.1462	23805	12.1	1174	956.0
7	287.1436	100560	51.1	4135	4064.2
8	288.1461	15228	7.7	785	619.3
9	487.2457	21060	10.7	1790	666.9
10	551.2978	22267	11.3	2193	756.6

Compound III

Generate Molecular Formula Parameters

Charge	Tolerance	SearchRadius	H/C Ratio min.	H/C Ratio max.	Electron Conf.	Nitrogen Rule	sigma limit
positive	10 ppm	0.05 m/z	0	3	both	true	0.05

Expected Formula C13 H17 B O3 **Adduct(s):** H, Na

#	meas. m/z	theo. m/z	Err[ppm]	Sigma	Formula
1	233.1360	233.134900	-6.10	0.0096	C 13 H 18 B 1 O 3
1	255.1164	255.116845	0.40	0.0012	C 13 H 17 B 1 Na 1 O 3

Figure S1.30. The ESI$^+$ mass spectrometry analysis of intermediate Compound **III.**

Ionisation Mode positive electrospray (ESI)

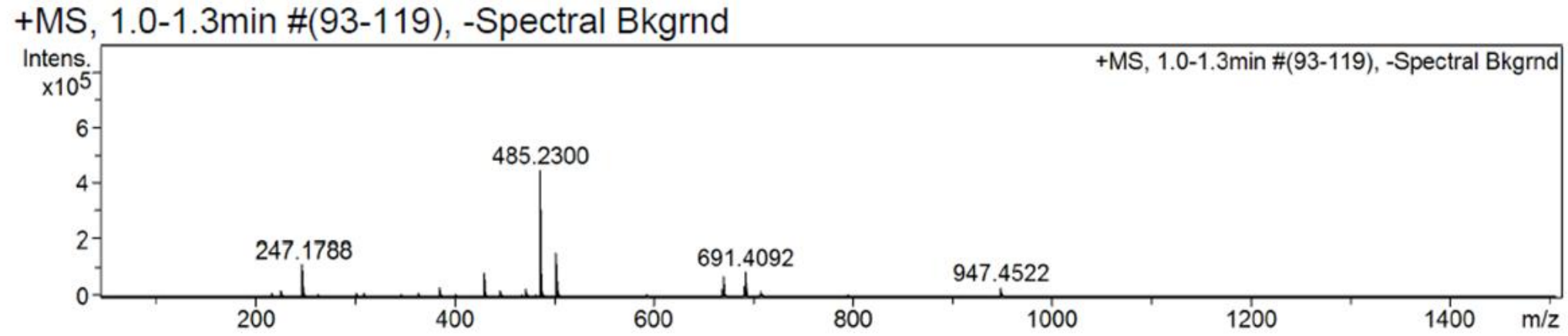

#	m/z	I	I %	Area	S/N
1	247.1788	114004	25.6	3956	5842.5
2	429.1593	85990	19.3	4653	1387.8
3	485.2300	445361	100.0	15647	4928.9
4	486.2253	106765	24.0	5822	1190.0
5	501.1993	158124	35.5	7324	1973.2
6	502.1988	41674	9.4	2912	524.2
7	669.4273	73085	16.4	5223	1172.3
8	690.4103	38327	8.6	3450	640.2
9	691.4092	87667	19.7	6601	1475.3
10	692.4114	35043	7.9	3221	594.1

Compound IV

Generate Molecular Formula Parameters

Charge	Tolerance	SearchRadius	H/C Ratio min.	H/C Ratio max.	Electron Conf.	Nitrogen Rule	sigma limit
positive	10 ppm	0.05 m/z	0	3	both	true	0.05

Expected Formula C31 H43 B N4 O4 s **Adduct(s):** H, Na

Figure S1.31. The ESI$^+$ mass spectrometry analysis of intermediate Compound I**V.**

Ionisation Mode negative electrospray (ESI)

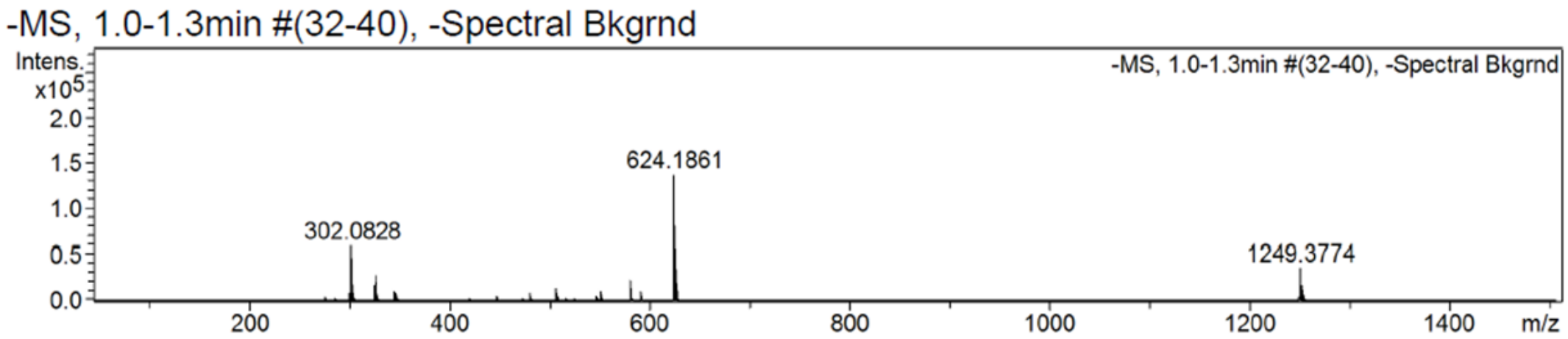

#	m/z	I	I %	Area	S/N
1	302.0828	59699	43.5	2667	641.5
2	325.0649	16150	11.8	636	195.7
3	326.0704	26819	19.5	1381	327.1
4	580.1944	23445	17.1	1863	445.1
5	624.1861	137186	100.0	10482	4702.6
6	625.1886	57986	42.3	4322	2025.1
7	626.1866	20324	14.8	1582	723.3
8	1249.3774	35856	26.1	6044	2965.9
9	1250.3790	29778	21.7	5394	2436.7
10	1251.3803	15666	11.4	2818	1268.2

Compound V

Generate Molecular Formula Parameters

Charge	Tolerance	SearchRadius	H/C Ratio min.	H/C Ratio max.	Electron Conf.	Nitrogen Rule	sigma limit
negative	10 ppm	0.05 m/z	0	3	both	true	0.05

Expected Formula C34 H31 N3 O7 S **Adduct(s):** H, Na

#	meas. m/z	theo. m/z	Err[ppm]	Sigma	Formula
1	624.1861	624.180446	-8.20	0.0197	C 34 H 30 N 3 O 7 S 1

Figure S1.32. The ESI$^-$ mass spectrometry analysis of Compound **V.**

Ionisation Mode positive electrospray (ESI)

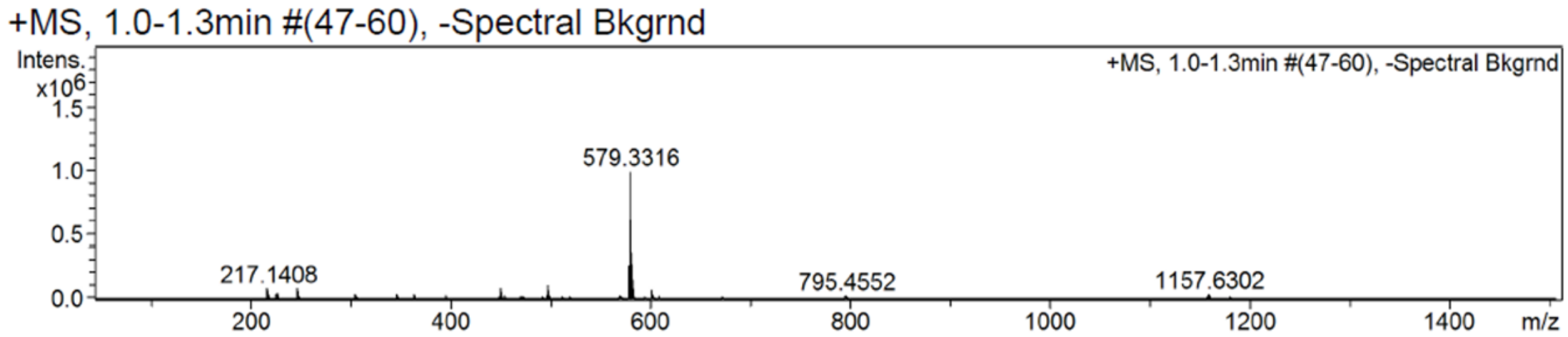

#	m/z	I	I %	Area	S/N
1	217.1408	82477	8.4	2958	5725.2
2	227.0865	45093	4.6	1700	2965.6
3	247.1793	81252	8.3	3330	4830.6
4	449.2245	82667	8.4	6184	1034.2
5	497.2417	102010	10.4	7643	780.1
6	578.3279	265871	27.0	19366	946.9
7	579.3316	984507	100.0	43258	3483.2
8	580.3289	370409	37.6	24496	1305.3
9	581.3225	129675	13.2	11646	460.5
10	601.3056	71099	7.2	6116	298.4

Compound VII

Generate Molecular Formula Parameters

Charge	Tolerance	SearchRadius	H/C Ratio min.	H/C Ratio max.	Electron Conf.	Nitrogen Rule	sigma limit
positive	10 ppm	0.05 m/z	0	3	both	true	0.05

Expected Formula C31 H43 B N4 O4 S **Adduct(s):** H, Na

#	meas. m/z	theo. m/z	Err[ppm]	Sigma	Formula

Figure S1.33. The ESI$^+$ mass spectrometry analysis of intermediate Compound **VII.**

Ionisation Mode positive electrospray (ESI)

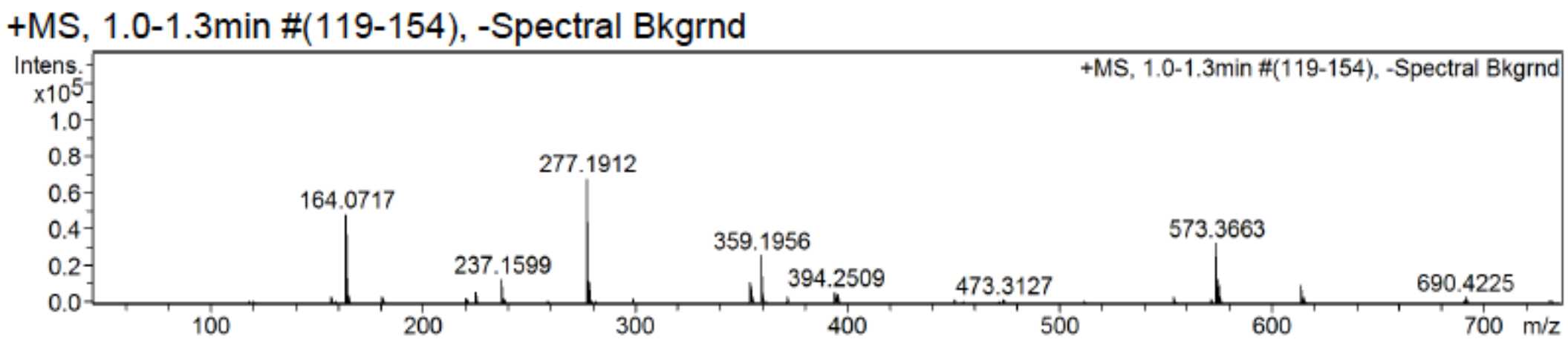

#	m/z	I	I %	Area	S/N
1	164.0717	48553	71.3	1320	12895.1
2	225.0866	5903	8.7	238	2151.4
3	237.1599	12916	19.0	531	4313.5
4	277.1912	68143	100.0	2979	9378.1
5	278.1941	12333	18.1	651	1661.3
6	354.2268	11170	16.4	799	1524.4
7	359.1956	26040	38.2	1721	3615.8
8	573.3663	32777	48.1	3546	1839.4
9	574.3692	13043	19.1	1458	725.6
10	613.3970	9341	13.7	1156	864.9

Generate Molecular Formula Parameters

Charge	Tolerance	SearchRadius	H/C Ratio min.	H/C Ratio max.	Electron Conf.	Nitrogen Rule	sigma limit
positive	10 ppm	0.05 m/z	0	3	both	true	0.05

Expected Formula C13H20N2O2 **Adduct(s):** H, Na

#	meas. m/z	theo. m/z	Err[ppm]	Sigma	Formula
1	237.1599	237.160303	-0.40	0.0118	C 13 H 21 N 2 O 2

Figure S1.34. The ESI$^+$ mass spectrometry analysis of intermediate Boc-protected p-xylylenediamine used.

(II) Mass spectrometry characterisation of Compounds 1-6

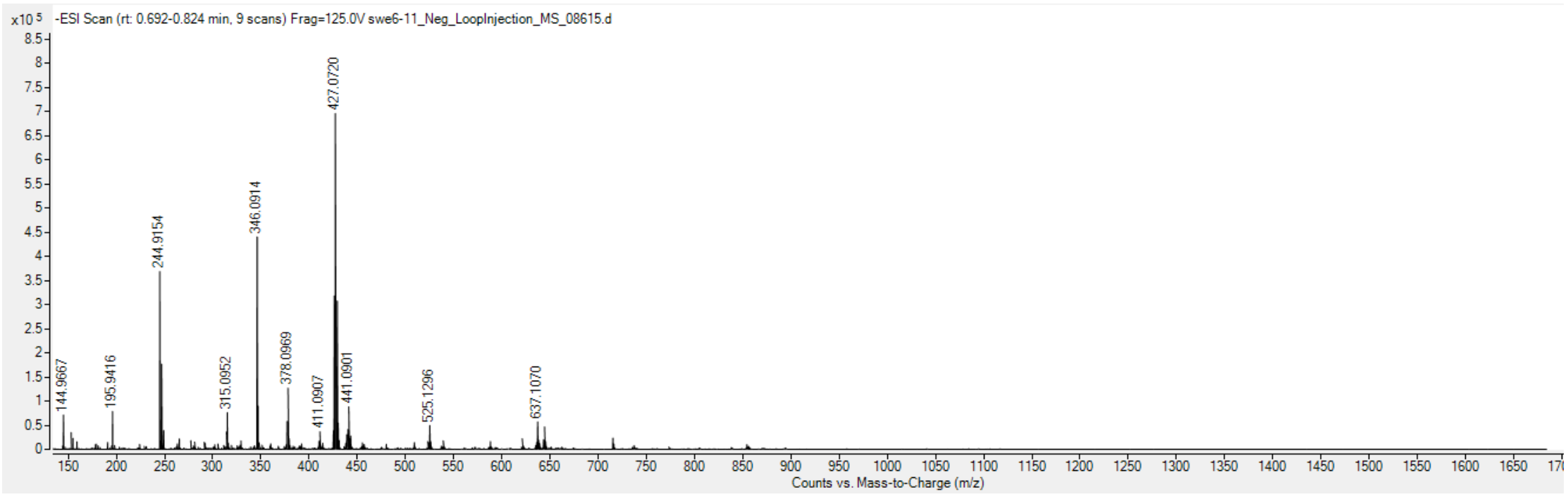

(a)

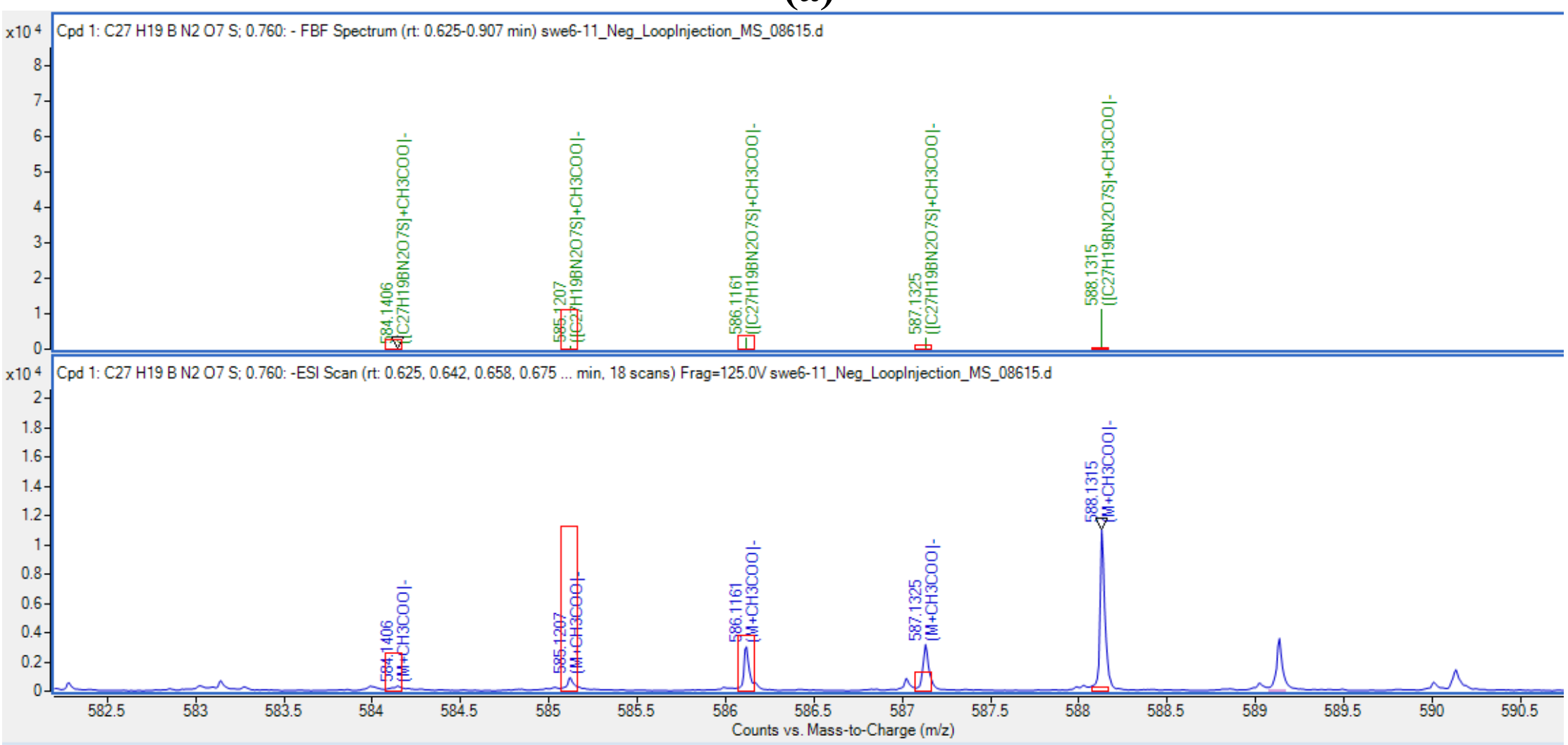

(b)

Figure S1.35. High resolution ESI+ mass spectrometry of Compound **1** (a) and corresponding isotopes matching (b).

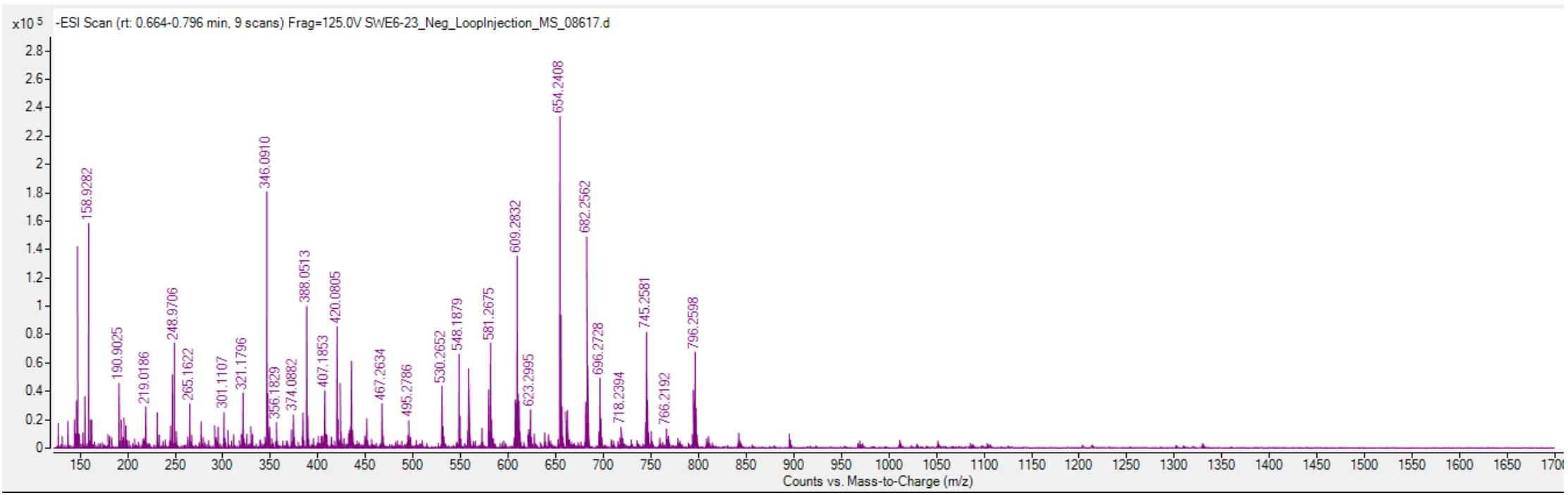

Figure S1.36. High resolution ESI+ mass spectrometry of Compound **2**

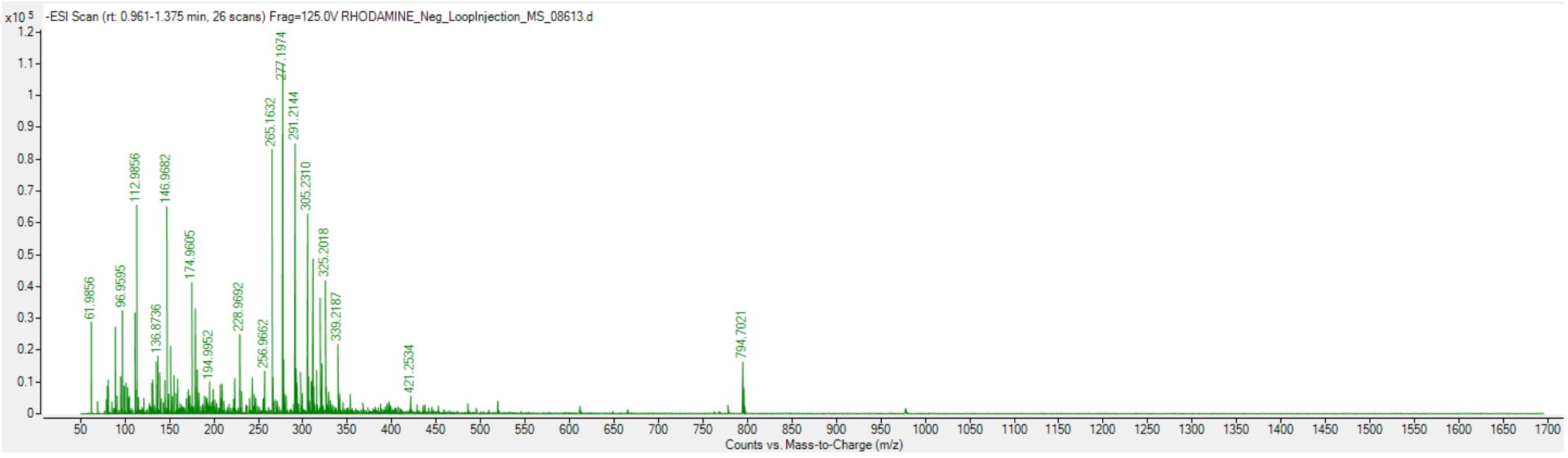

(a)

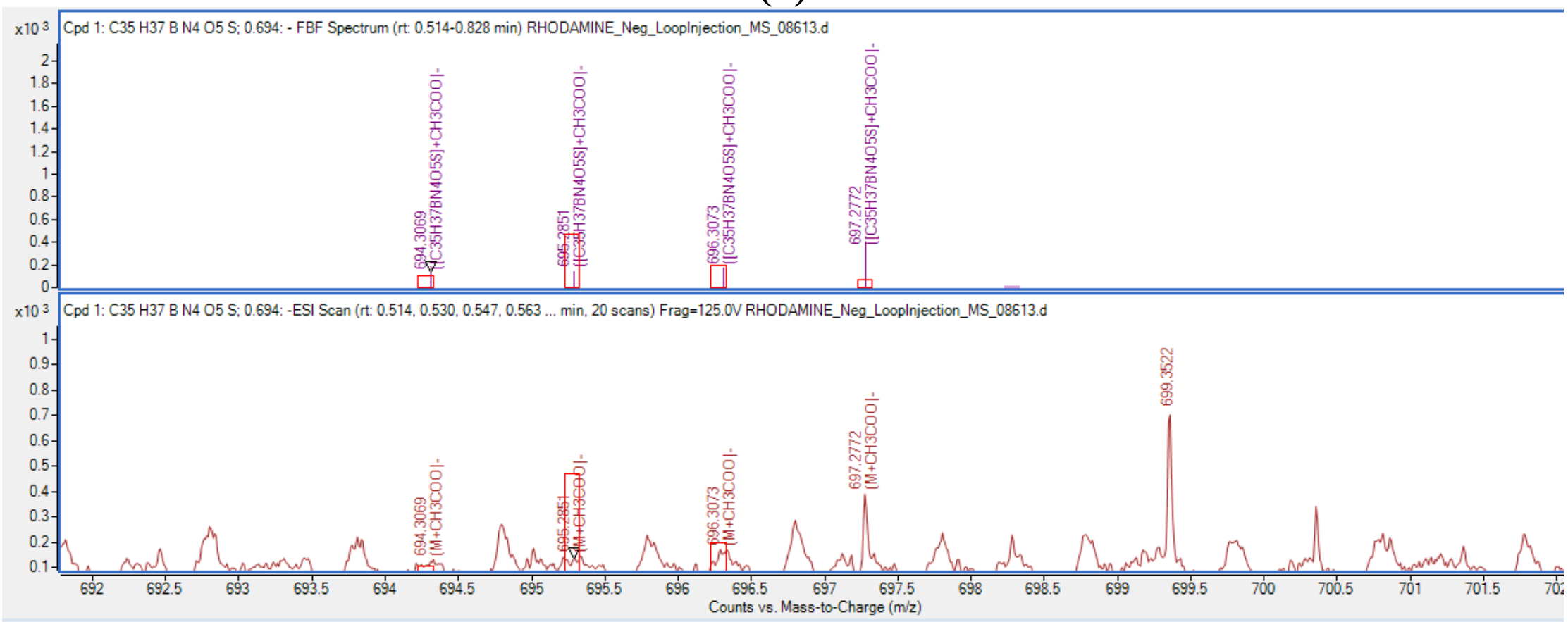

(b)

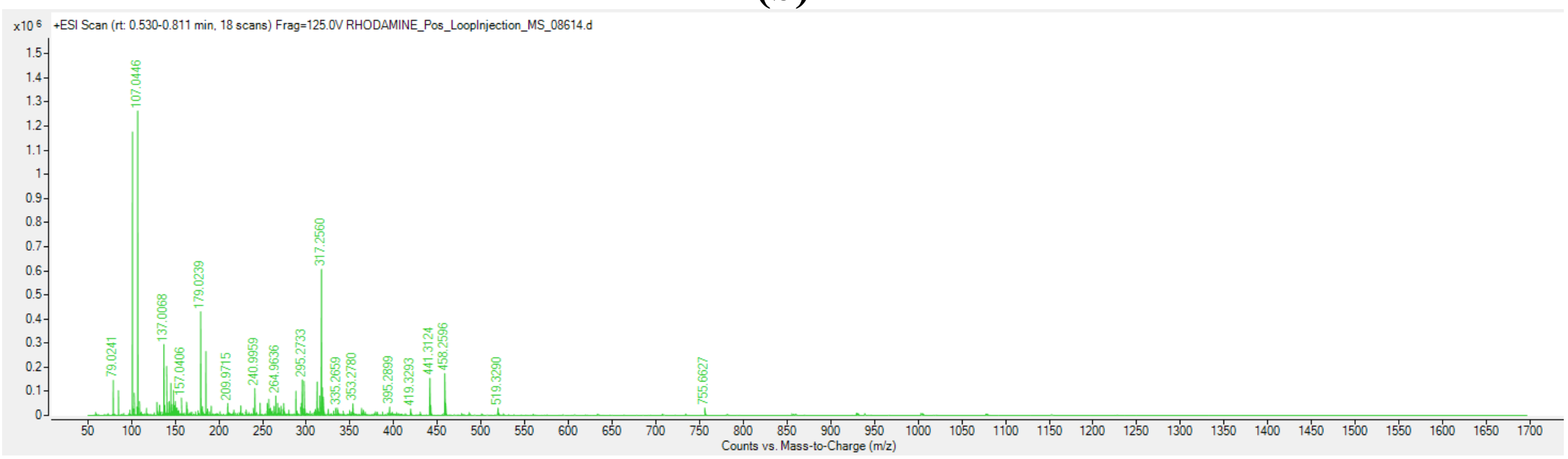

(c)

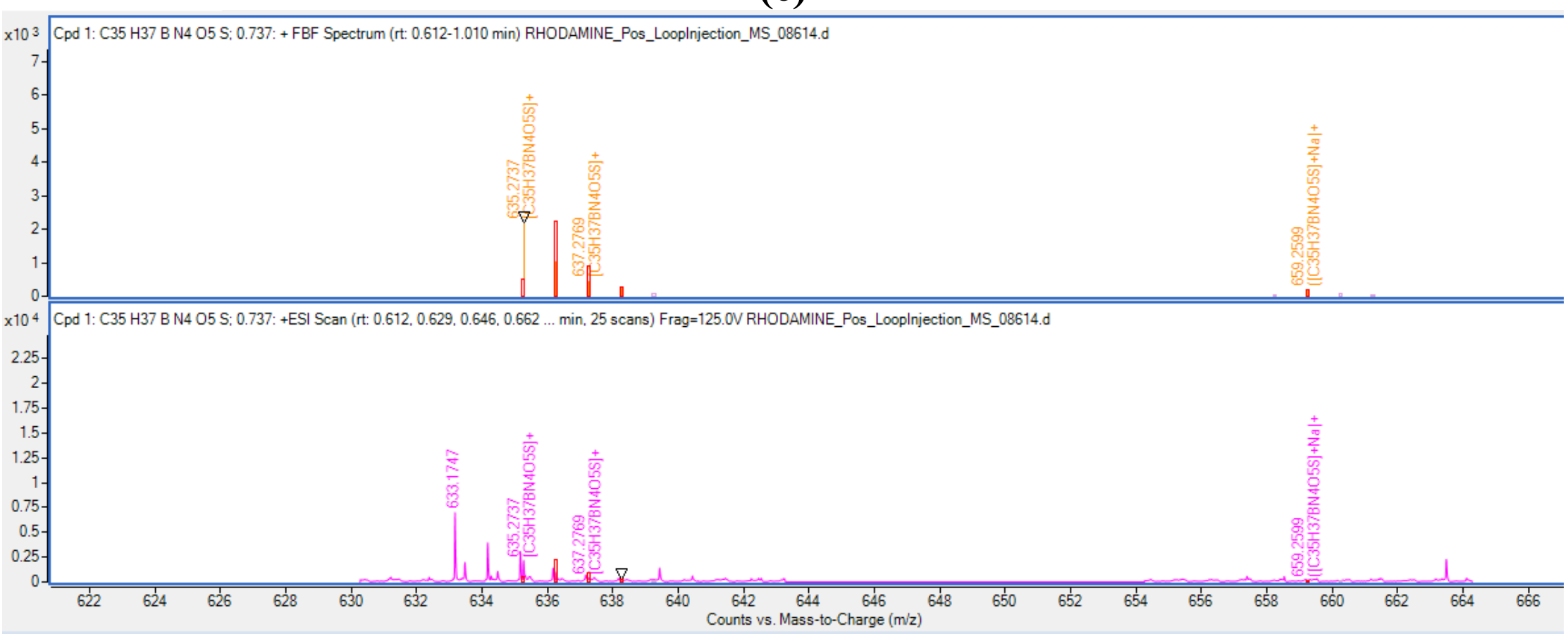

(d)

Figure S1.37. High resolution ESI- (a-b) and ESI+ (c-d) mass spectrometry of Compound **3** and corresponding isotopes matching.

-MS, 1.0-1.3min #(31-39), -Spectral Bkgrnd

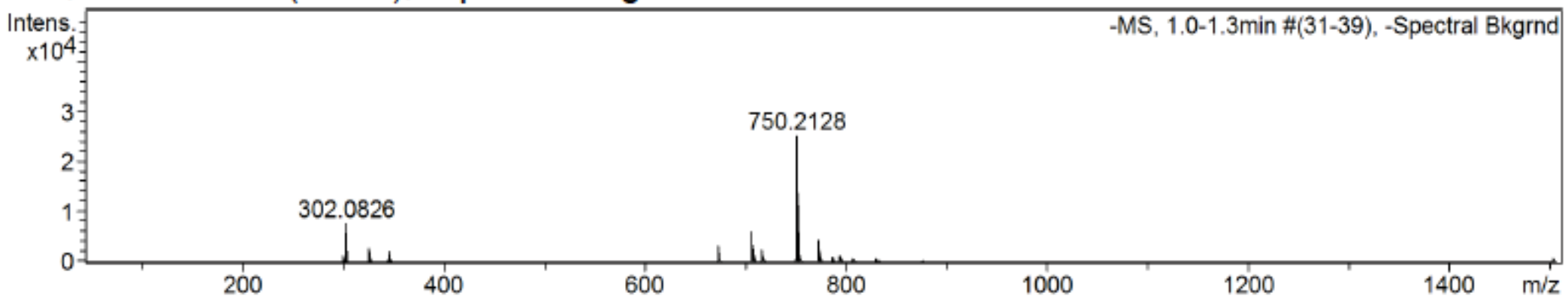

#	m/z	I	I %	Area	S/N
1	302.0826	7625	30.3	370	2526.6
2	326.0716	2715	10.8	125	875.4
3	672.2341	3239	12.9	245	401.8
4	706.2249	6049	24.1	478	603.9
5	707.2259	2705	10.8	234	268.4
6	716.2283	2325	9.3	194	218.9
7	750.2128	25123	100.0	2519	1982.4
8	751.2149	11385	45.3	1116	894.2
9	752.2143	5484	21.8	511	428.7
10	772.1947	4383	17.4	393	313.1

Generate Molecular Formula Parameters

Charge	Tolerance	SearchRadius	H/C Ratio min.	H/C Ratio max.	Electron Conf.	Nitrogen Rule	sigma limit
negative	10 ppm	0.05 m/z	0	3	both	true	0.05

Expected Formula C39 H37 N5 O7 S2 **Adduct(s):** H, Na

#	meas. m/z	theo. m/z	Err[ppm]	Sigma	Formula
1	750.2128	750.205615	-8.90	0.0056	C 39 H 36 N 5 O 7 S 2

Figure S1.38. High resolution ESI- (mass spectrometry of Compound **4**, indicating the presence of expected peaks at M/z 750.2128. assignable to $[M-H^{+}]^{-}$.

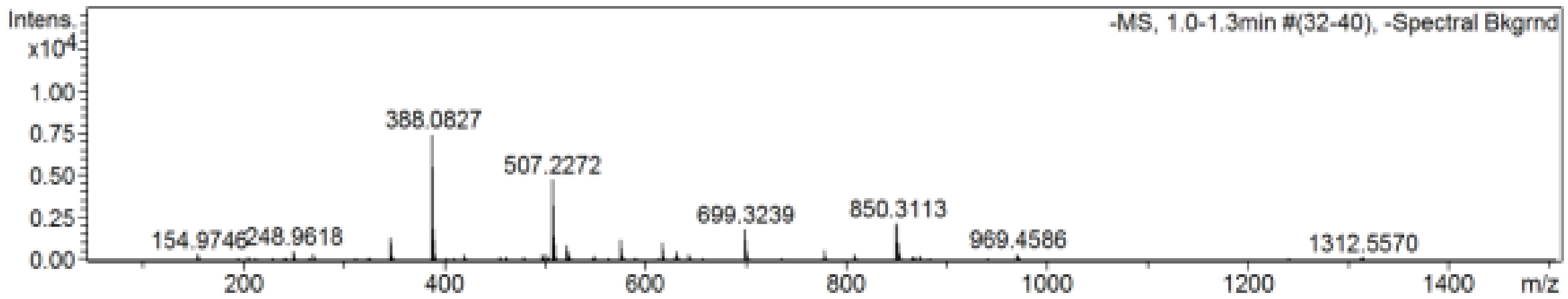

Figure S1.39. The ESI-MS spectrum of Compound **5**, indicating the presence of expected peaks at M/z 969.4586, assignable to $[M+Na-H]^{-}$

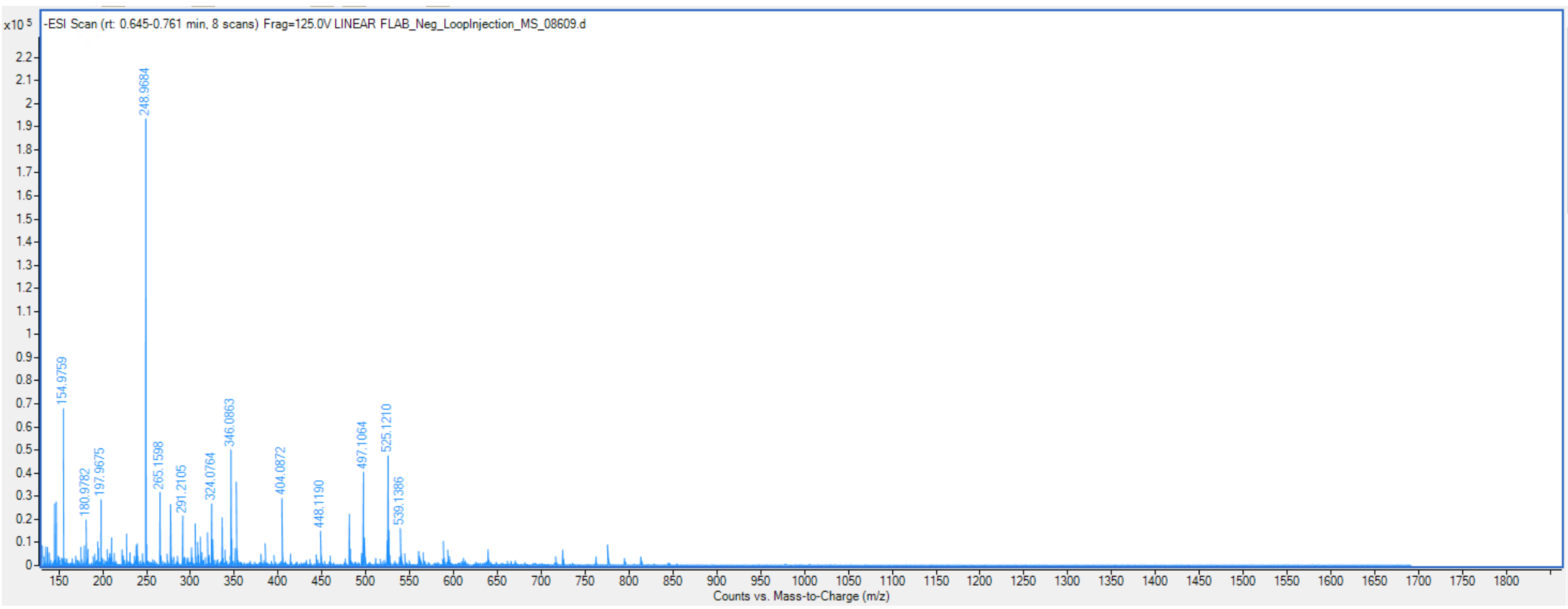

(a)

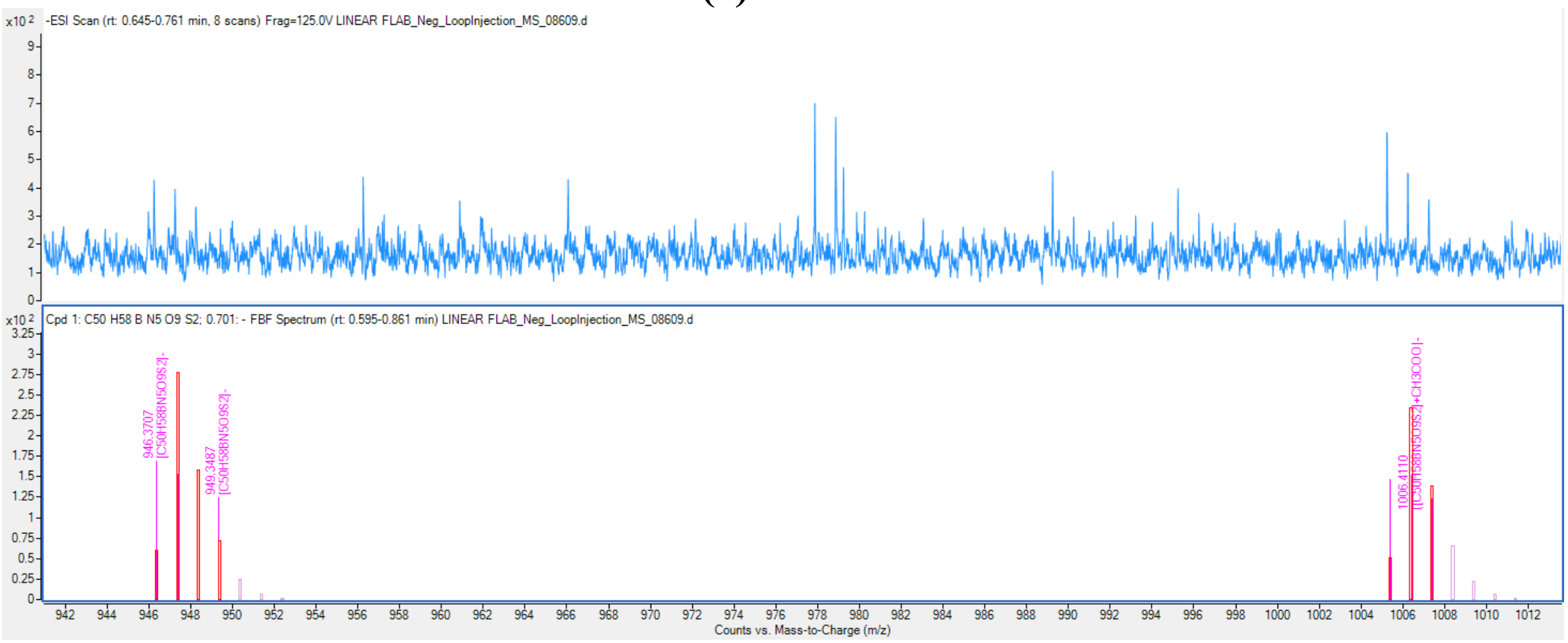

(b)

Figure S1.40. High resolution ESI⁻ mass spectrum of Compound **5** (a) and corresponding isotopes matching (b).

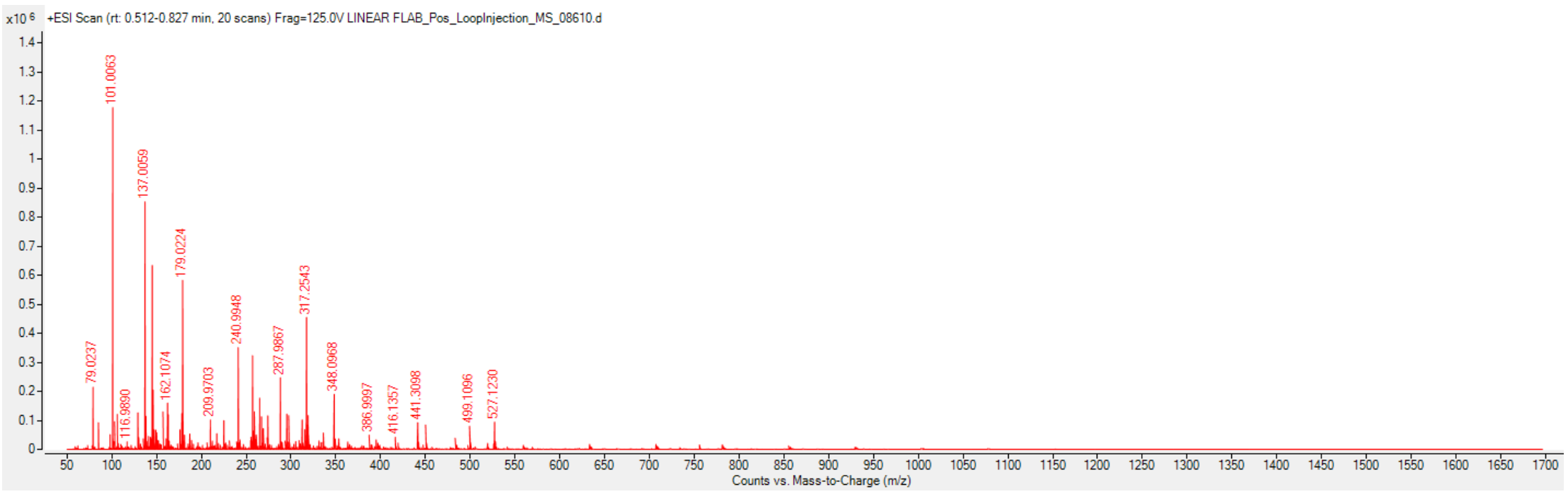

(a)

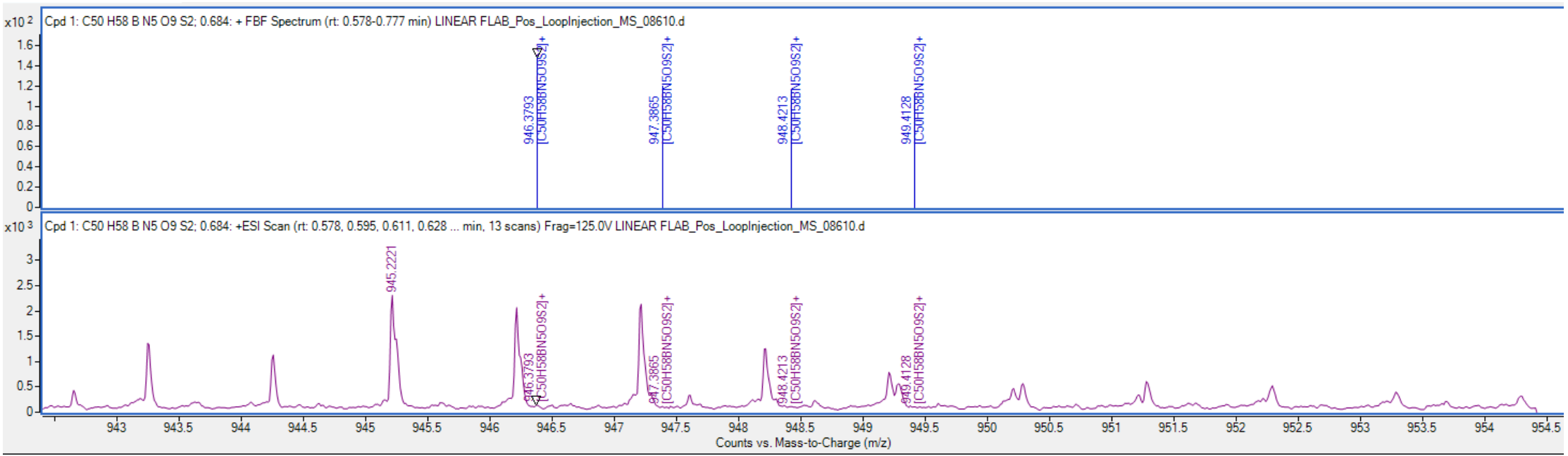

(b)

Figure S1.41. High resolution ESI^+ mass spectrum of Compound **5** (including expansion) and corresponding isotopes matching.

Ionisation Mode negative electrospray (ESI)

-MS, 1.0-1.3min #(32-40), -Spectral Bkgrnd

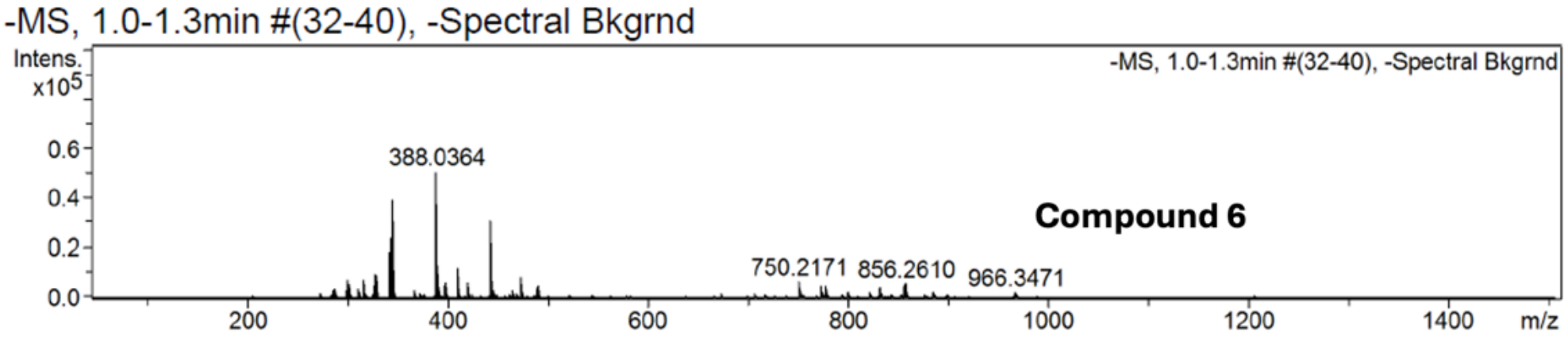

#	m/z	I	I %	Area	S/N
1	328.0631	9419	18.6	479	87.1
2	342.0288	18016	35.6	853	165.9
3	343.0360	23449	46.3	1278	216.0
4	344.0426	39244	77.5	2007	361.8
5	345.0434	8254	16.3	495	75.6
6	388.0364	50647	100.0	3003	459.4
7	389.0396	13020	25.7	751	118.0
8	410.0179	11996	23.7	588	114.6
9	442.0582	30833	60.9	1855	322.5
10	473.2875	8320	16.4	507	95.1

Generate Molecular Formula Parameters

Charge	Tolerance	SearchRadius	H/C Ratio min.	H/C Ratio max.	Electron Conf.	Nitrogen Rule	sigma limit
negative	10 ppm	0.05 m/z	0	3	both	true	0.05

Expected Formula C52 H54 B N5 O9 S2 **Adduct(s):** H, Na

#	meas. m/z	theo. m/z	Err[ppm]	Sigma	Formula
1	966.3471	966.337775	-8.10	0.0362	C 52 H 53 B 1 N 5 O 9 S 2

(a)

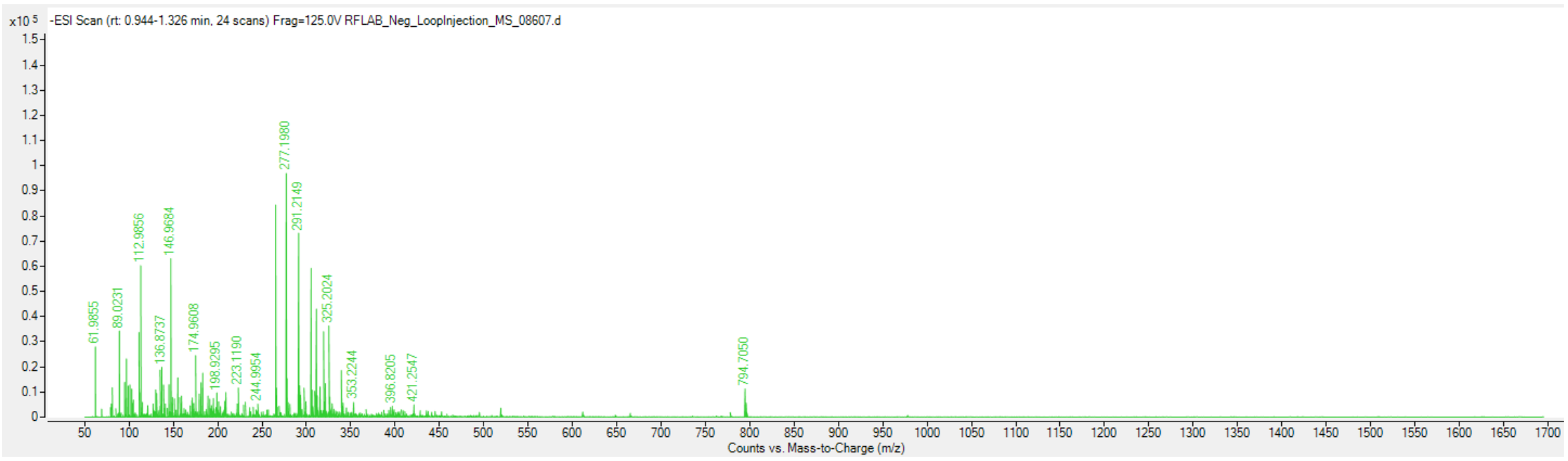

(b)

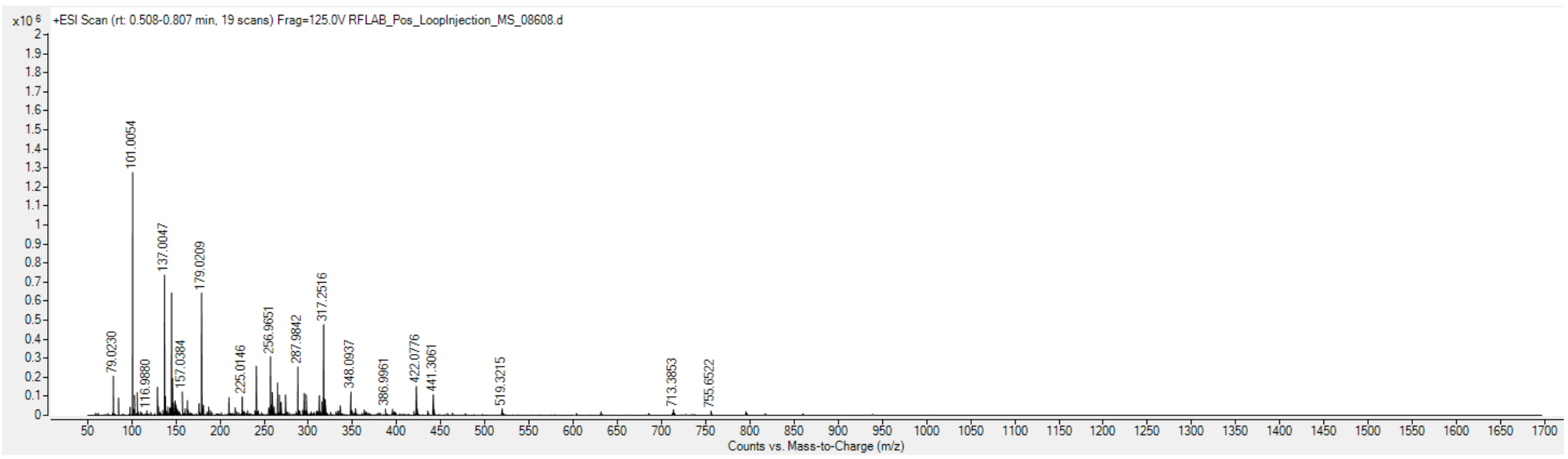

(c)

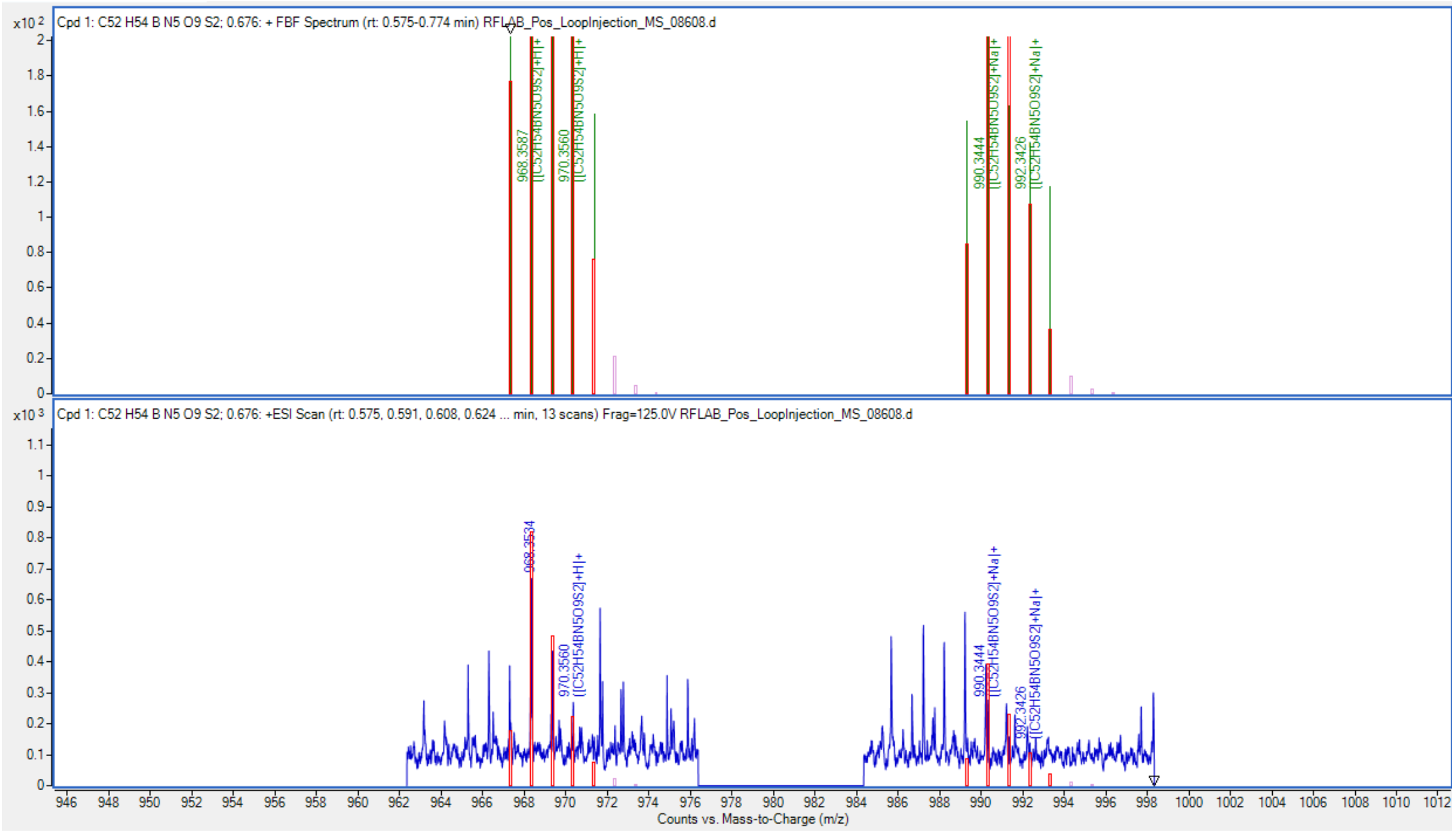

(d)

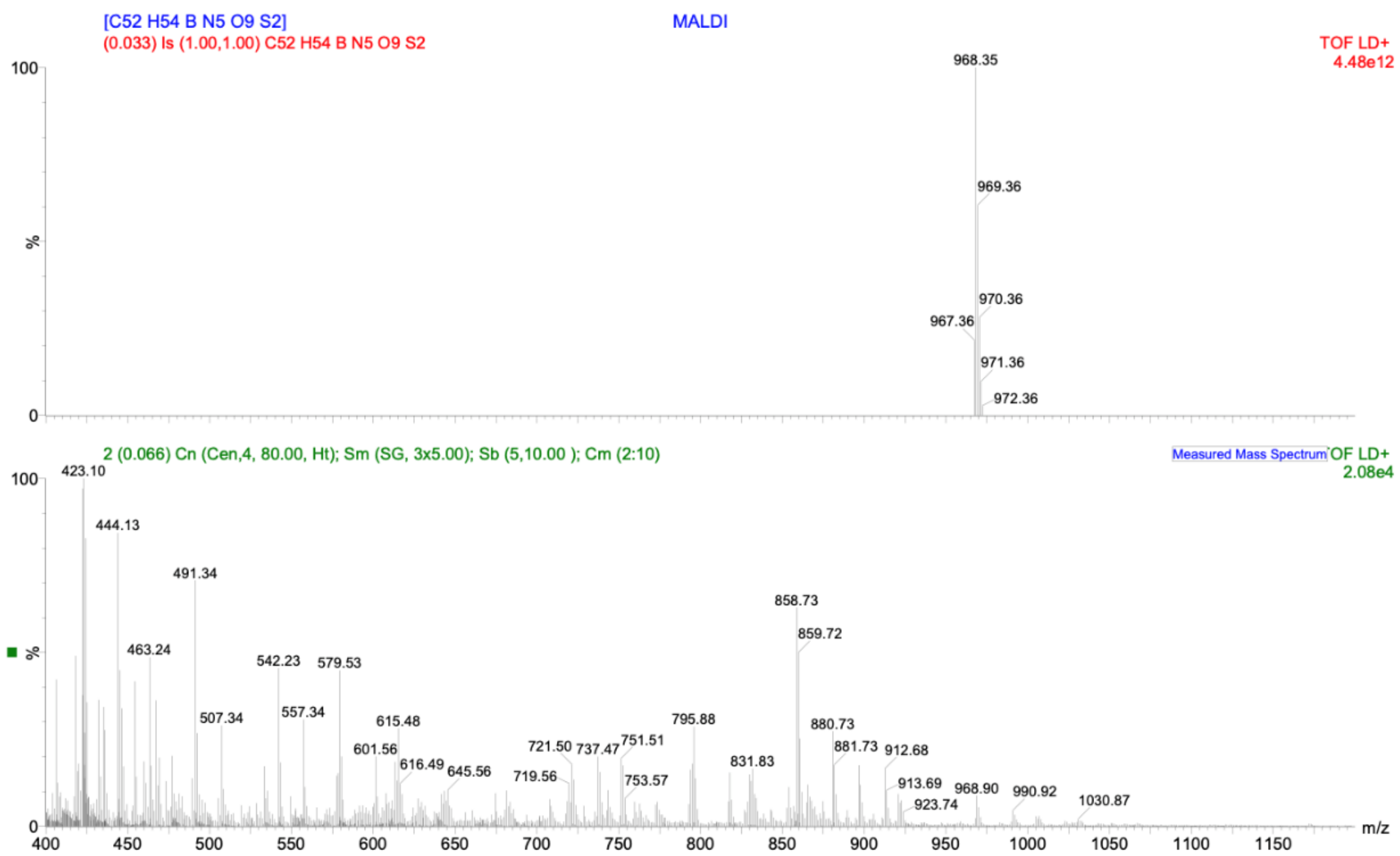

(e)

Figure S1.42. High resolution ESI^+ mass spectrometry of Compound **6** (including expansion) and corresponding isotopes matching (a-d) and MALDI spectroscopy confirming formation of desired species (e), alongside pinacol-deprotected derivative.

Solid-stated Infrared spectra of selected precursors synthesised

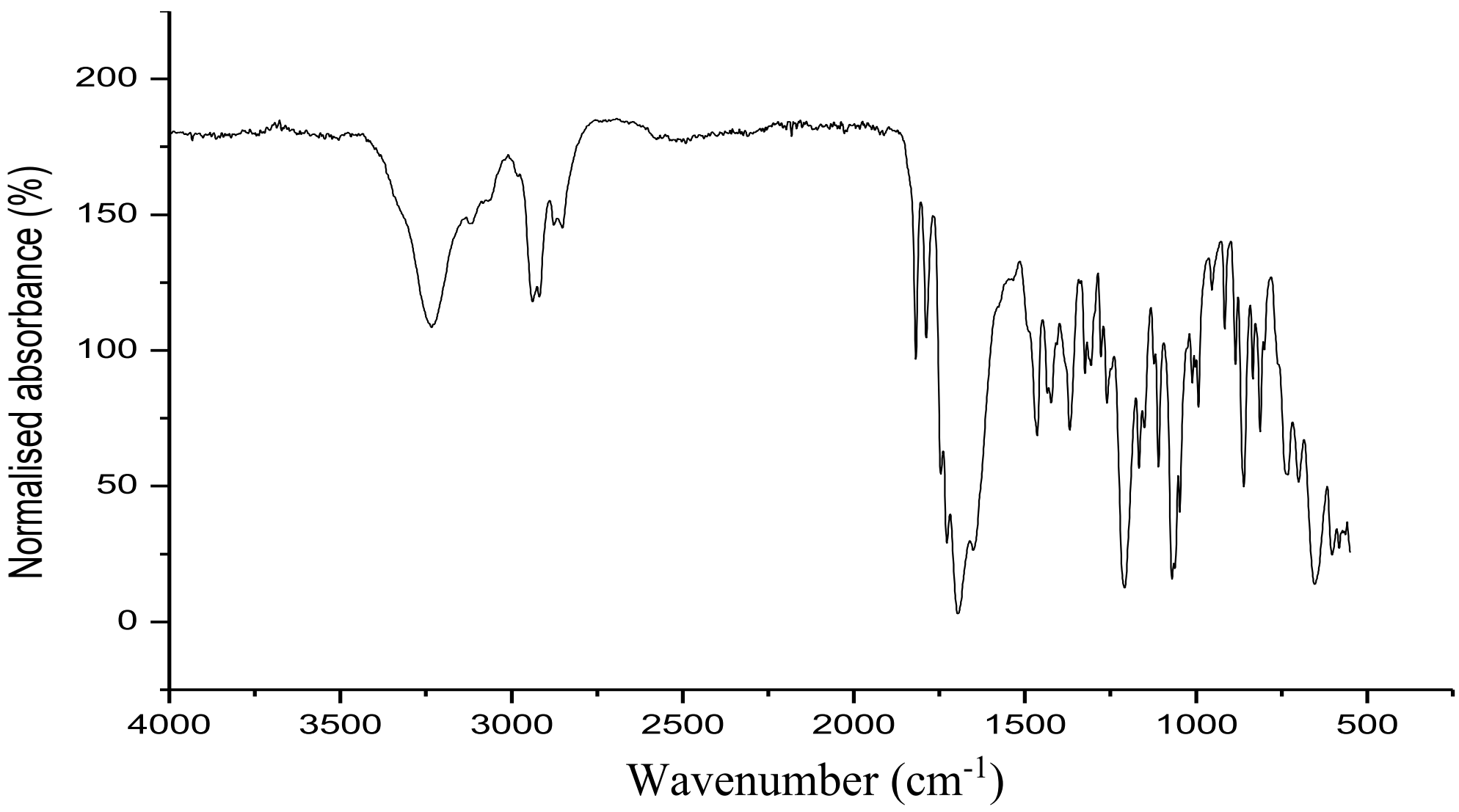

Selected IR band and Assignment	Wavenumber (cm^{-1})
biotin ureido C=O	1711
-NH-C=O	1645
N-H bend with C-N stretch	1552
cycloalkane	1061
Alkane chain	861

Figure S1.43. Infrared spectrum of compound **I-A.**

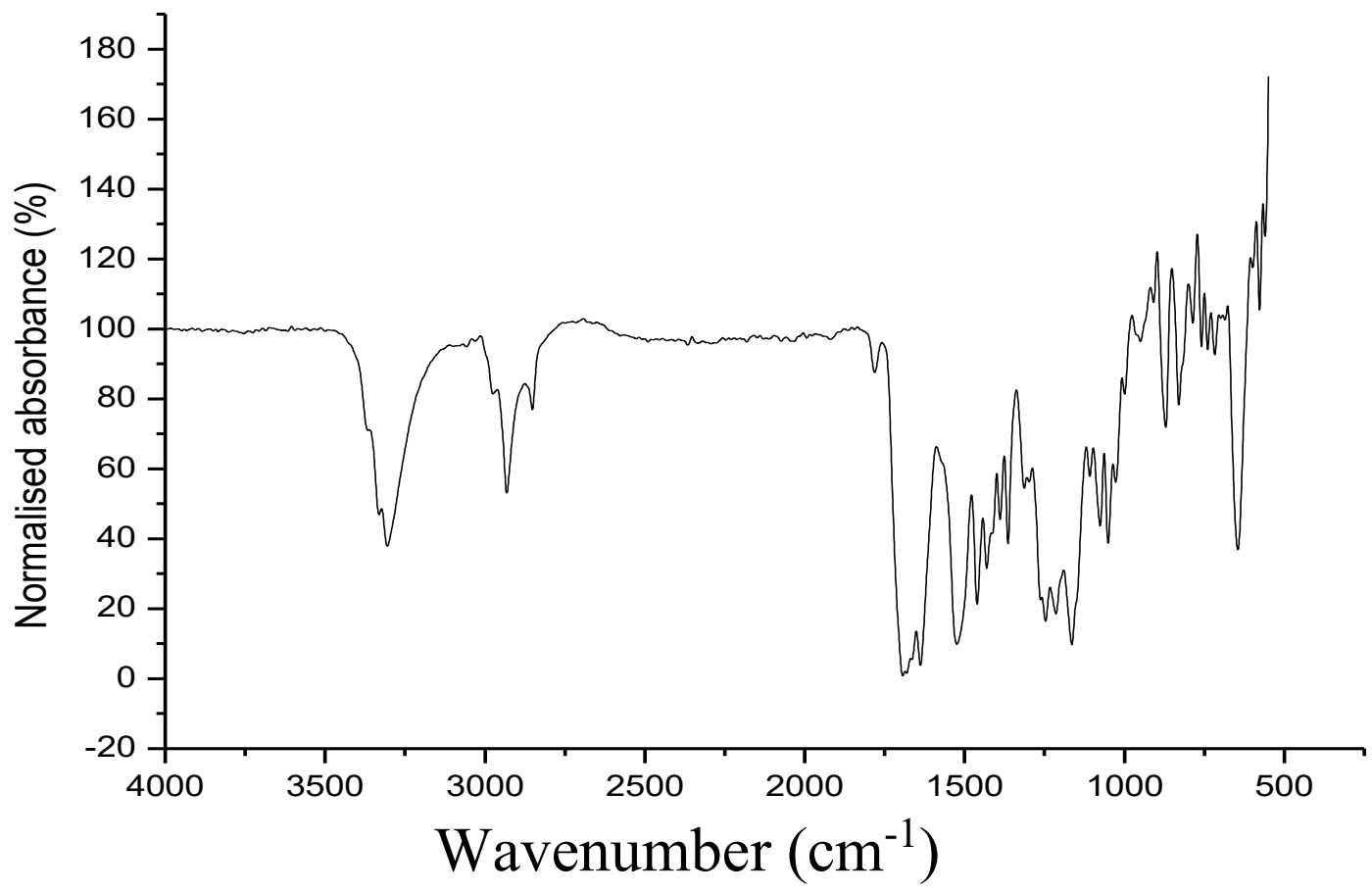

Selected IR band and Assignment	Wavenumber (cm^{-1})
Ar C-H	3298
biotin ureido C=O	1778
-NH-C=O	1695
Ar C-C	1522;1637
C-N	1163
Alkane chain	871

Figure S1.44. Infrared spectrum of compound **IV.**

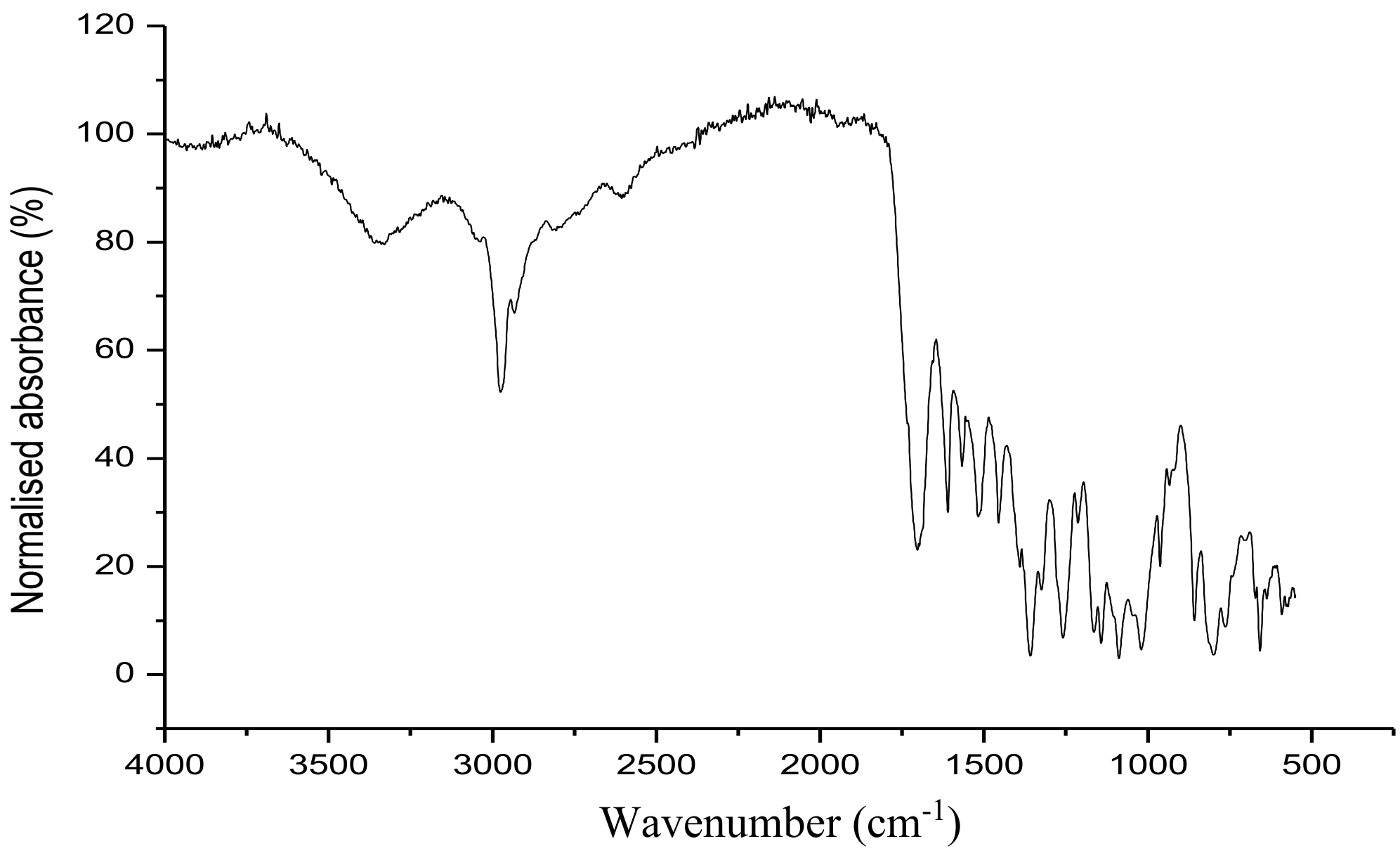

Selected IR band and Assignment	Wavenumber (cm^{-1})
Ar C-H	2973
biotin ureido C=O	1704
-NH-C=O	1610
Ar C-C	1510;1566
B-O	1357
C-N	1164
$-C(CH_3)_2$	1084;1141
Alkane chain	857
B-C	658

Figure S1.45. Infrared spectrum of compound **VII**

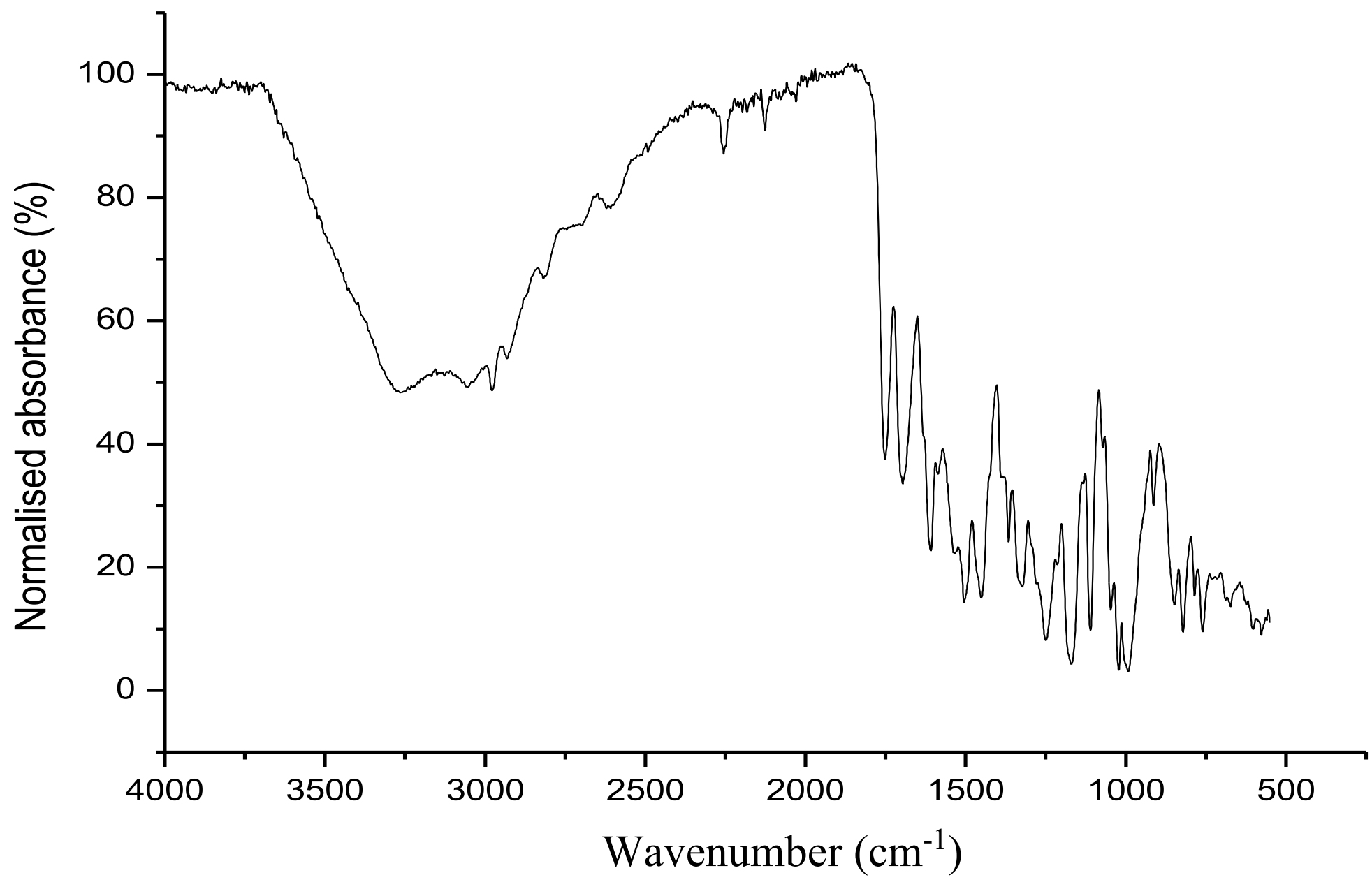

Selected IR band and Assignment	Wavenumber (cm^{-1})
COO-H	3256
Ar C=O	1751
-NH-C=O	1696
Ar C-C	1608;1535
C=S	1247
C-N	1164

Figure S1.46. Infrared spectrum of compound **V.**

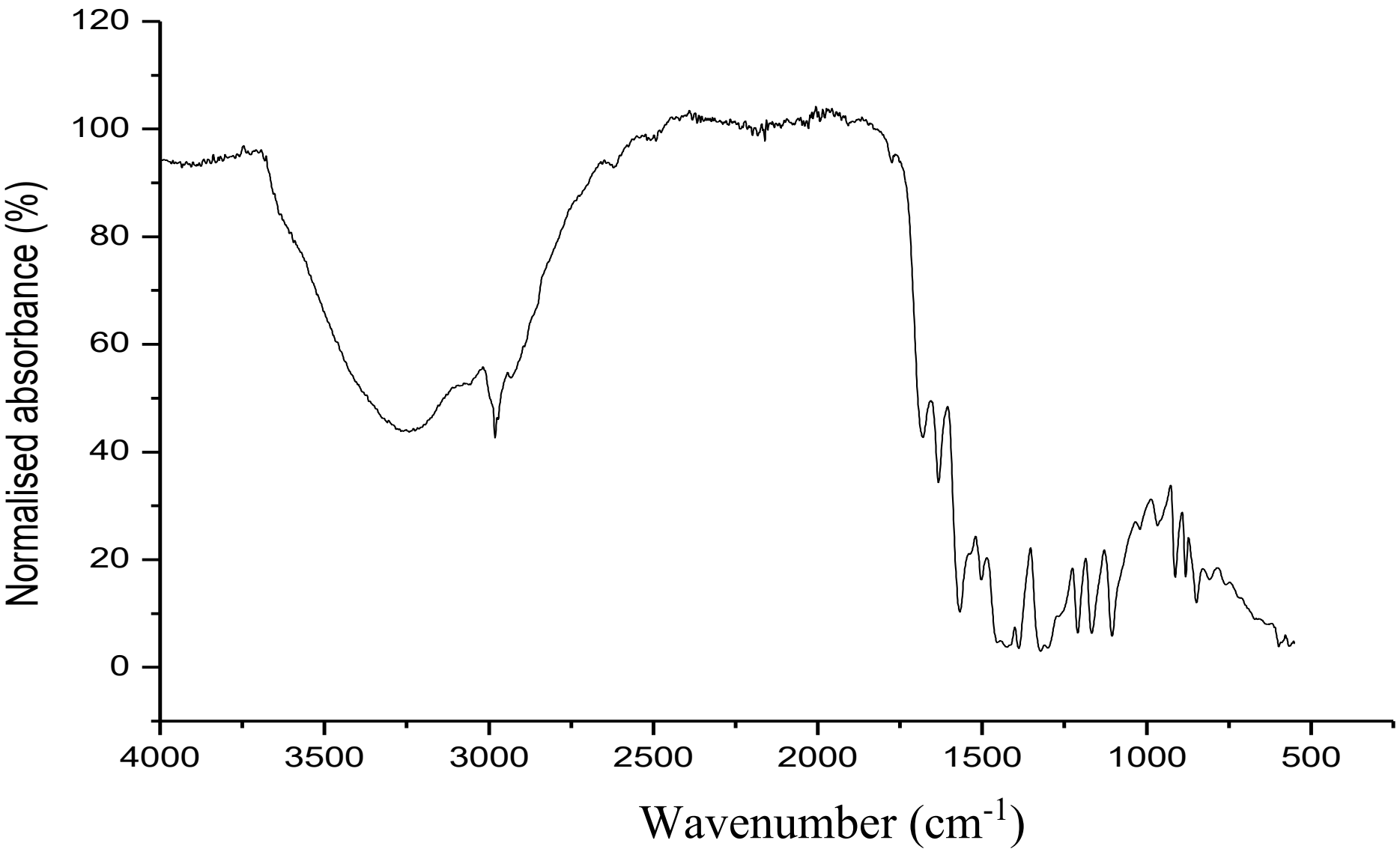

Selected IR band and Assignment	Wavenumber (cm^{-1})
COO-H	3244
Ar C=O	1751
-NH-C=O	1632
Ar C-C	1608;1535
C=S	1247
C-N	1166
Alkane chain	849

Figure S1.47. Infrared spectrum of compound **4.**

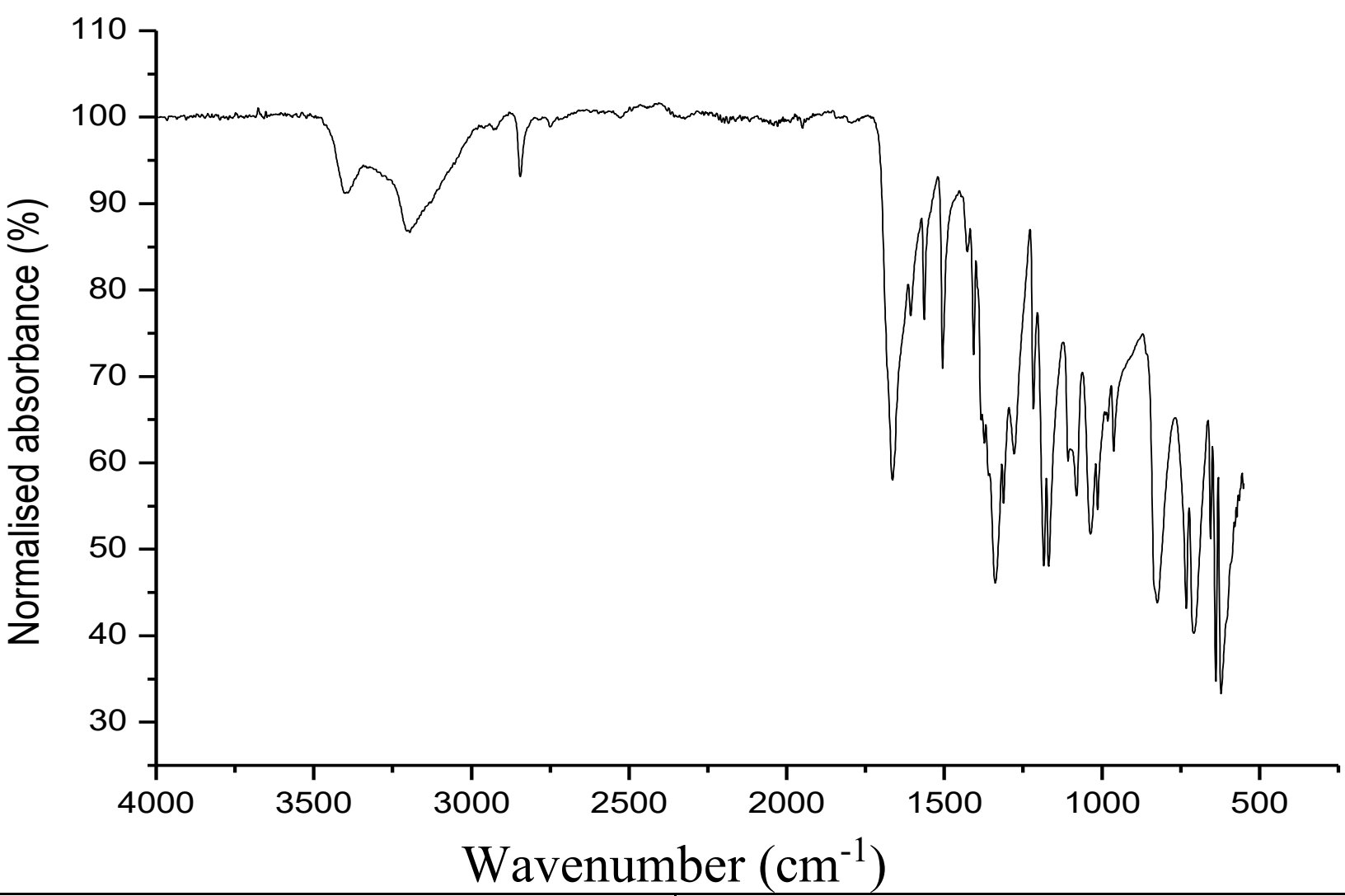

Selected IR band and Assignment	Wavenumber (cm^{-1})
C=O	1662
Ar C-C	1505;1564
$-C(CH_3)_2$	1167;1184
B-O	1339
B-C	600-1200

Figure S1.48. Infrared spectrum of compound **III.**

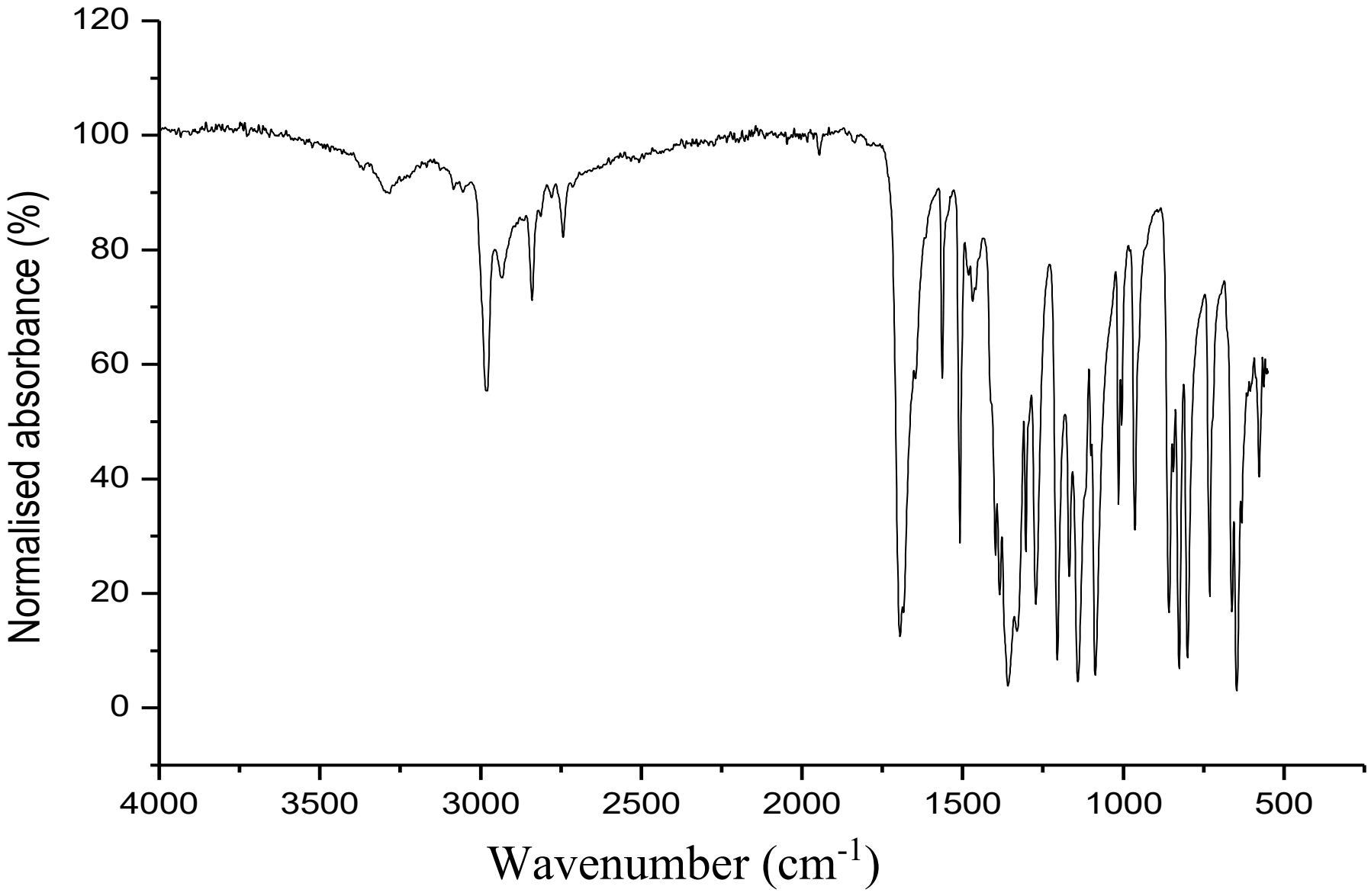

Selected IR band and Assignment	Wavenumber (cm^{-1})
C-H	2980
C=O	1695
Ar C-C	1508;1564
$-C(CH_3)_2$	1086;1140
B-O	1357
B-C	600-1200

Figure S1.49. Infrared spectrum of compound **VI**

2. Synthesis and characterisation of nanoparticles

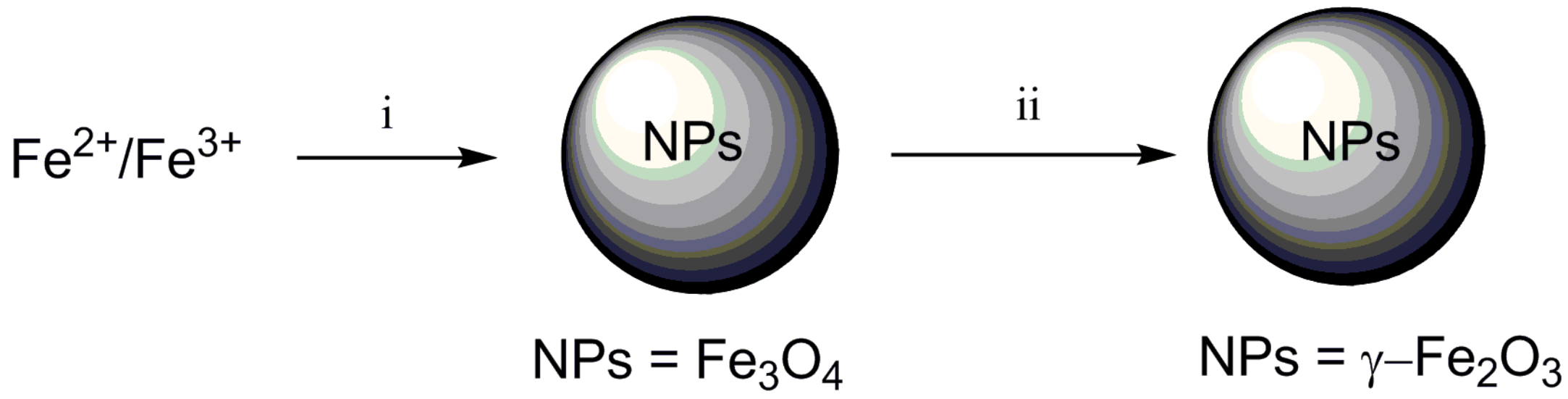

Scheme S2.1. Assemble of Fe_3O_4 nanoparticles

General considerations: Core-shells nanoparticles were obtained by encapsulating the synthesised magnetic Fe_3O_4 with amorphous SiO_2 using the Stöber process,[5] following our previously established methodology.[6] To evaluate the characteristics of the produced coatings, the FTIR characterisation was carried out. The peaks at ~799, 944, 1047 and 1152 cm^{-1} are attributed to the stretching vibration of the Si-O, Si-O-H, Si-O-Si and Si-O-Si bonds, respectively, indicating that the SiO_2 layer was encapsulated on the surface of the Fe_3O_4 nanoparticles.

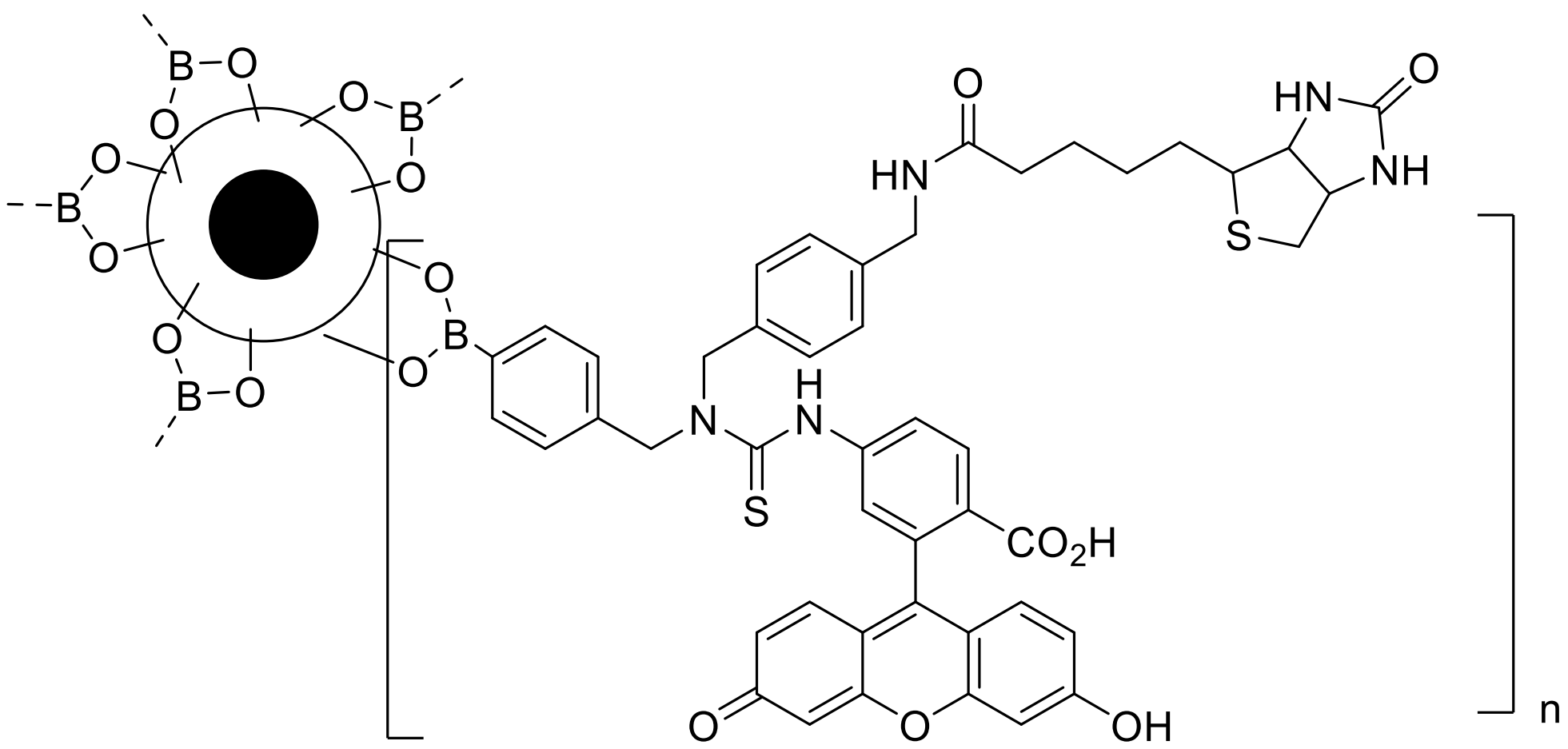

Figure S2.1. Proposed connectivity of multiple units of Compound **6** to the -Si(OH)$_2$ groups on the surface of Fe_3O_4@SiO_2 core-shell nanoparticles

Fe_3O_4@SiO_2 nanoparticles (5 mg) were dispersed in methanol (6 mL) in a pressure tube, followed by the addition of a methanolic solution of compound 6 (10 mg in 3 mL). The reaction mixture was gently agitated at 37 °C for 48 h. The resulting functionalised magnetic nanoparticles were isolated using a permanent magnet and washed with methanol (3 × 5 mL) to remove unreacted material. Raman spectroscopy was subsequently employed to confirm the composition of the nanoparticles. A characteristic band at approximately 689 cm^{-1}, assigned to the A_1g symmetric stretching vibration of Fe–O bonds, together with the $T_2g(3)$ mode at approximately 300 cm^{-1},

confirmed the presence of the Fe_3O_4 core. Additional Raman bands at approximately 493, 355, and 186 cm^{-1} were attributed to SiO_2, providing further evidence for the formation of the Fe_3O_4@SiO_2 core–shell structure.

The morphological features of Fe_3O_4@SiO_2 were observed by TEM (Figure S10.3 a and b). The encapsulation with silica renders an archetypal core-shell hierarchical structure is obtained consisting of a core of a few units of the Fe_3O_4 nuclei (sizes ca. 10 nm), homogeneously encapsulated in a SiO_2 shell whose thickness (ca. 40 nm) can be easily controlled throughout the process. The statistical treatment of the micrographs reveals a mean size for the whole composite displays in the order as that of 150 nm on average), indicating that the tendency of the synthesized nanoparticles to agglomerate is effectively prevented during the formation procedure of the composites.

After the functionalization of the core-shell with compound **6**, it can be seen in the micrographs of TEM that the morphology is maintained after the treatment. These MNPs were fund to be weakly fluorescent: 2D Excitation/Emission Spectrum in 1 mg/mL H_2O showed a maximum Excitation at 500 nm and a maximum emission at 515 nm. These also showed significant fluorescence quenching with respect to free compound **6** in 2 photon fluorescence spectroscopies.

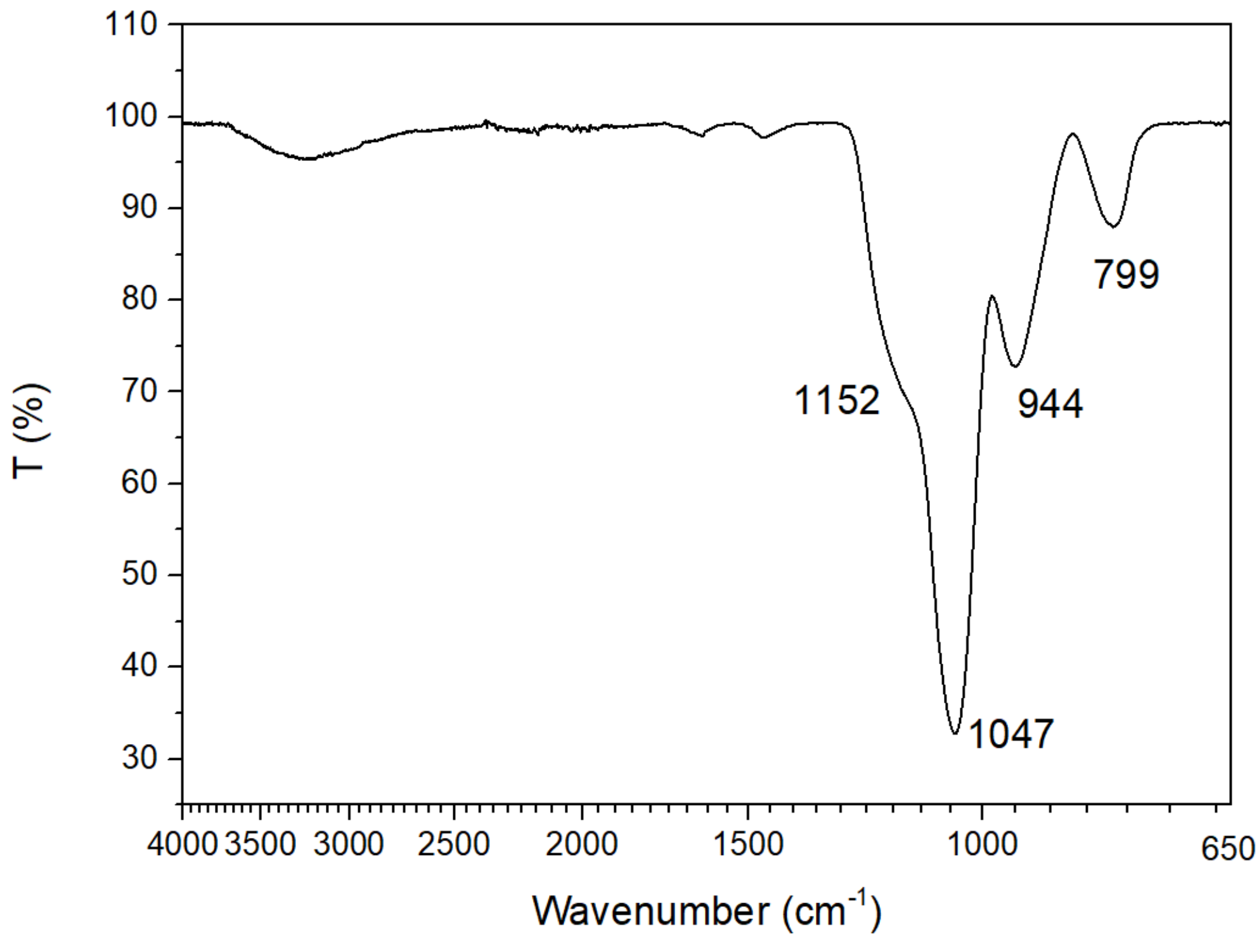

Figure S2.2. FTIR of Fe_3O_4@SiO_2 core-shell nanoparticles

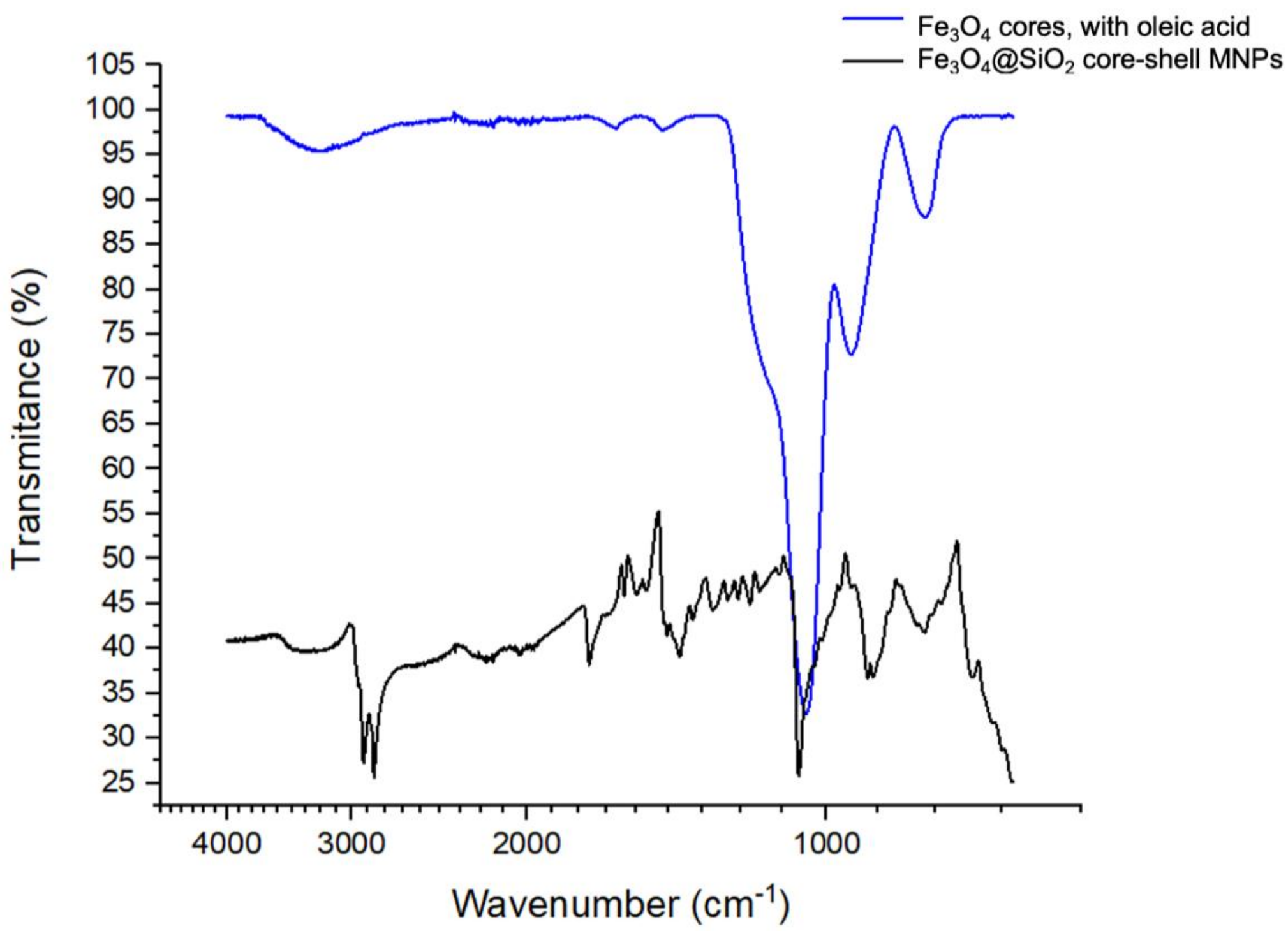

Figure S2.3. Overlay of the FTIR spectra of the Fe_3O_4 nanoparticles stabilised with oleic acid and Fe_3O_4@SiO_2 core-shell nanoparticles.

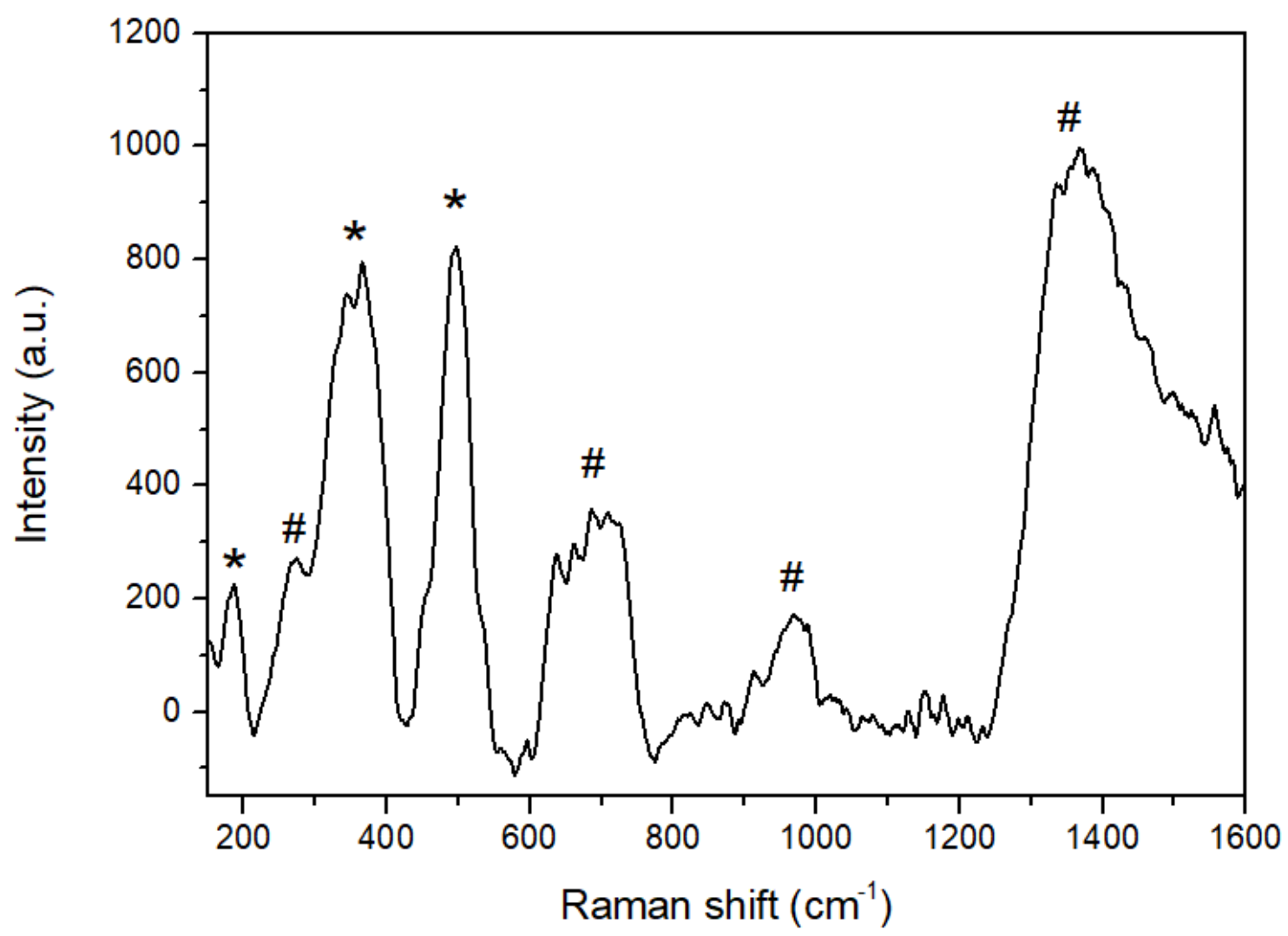

Figure S2.4. Raman spectra of Fe_3O_4@SiO_2 core-shell nanoparticles. # and * indicate the peaks assignable to Fe_3O_4 and SiO_2, respectively.

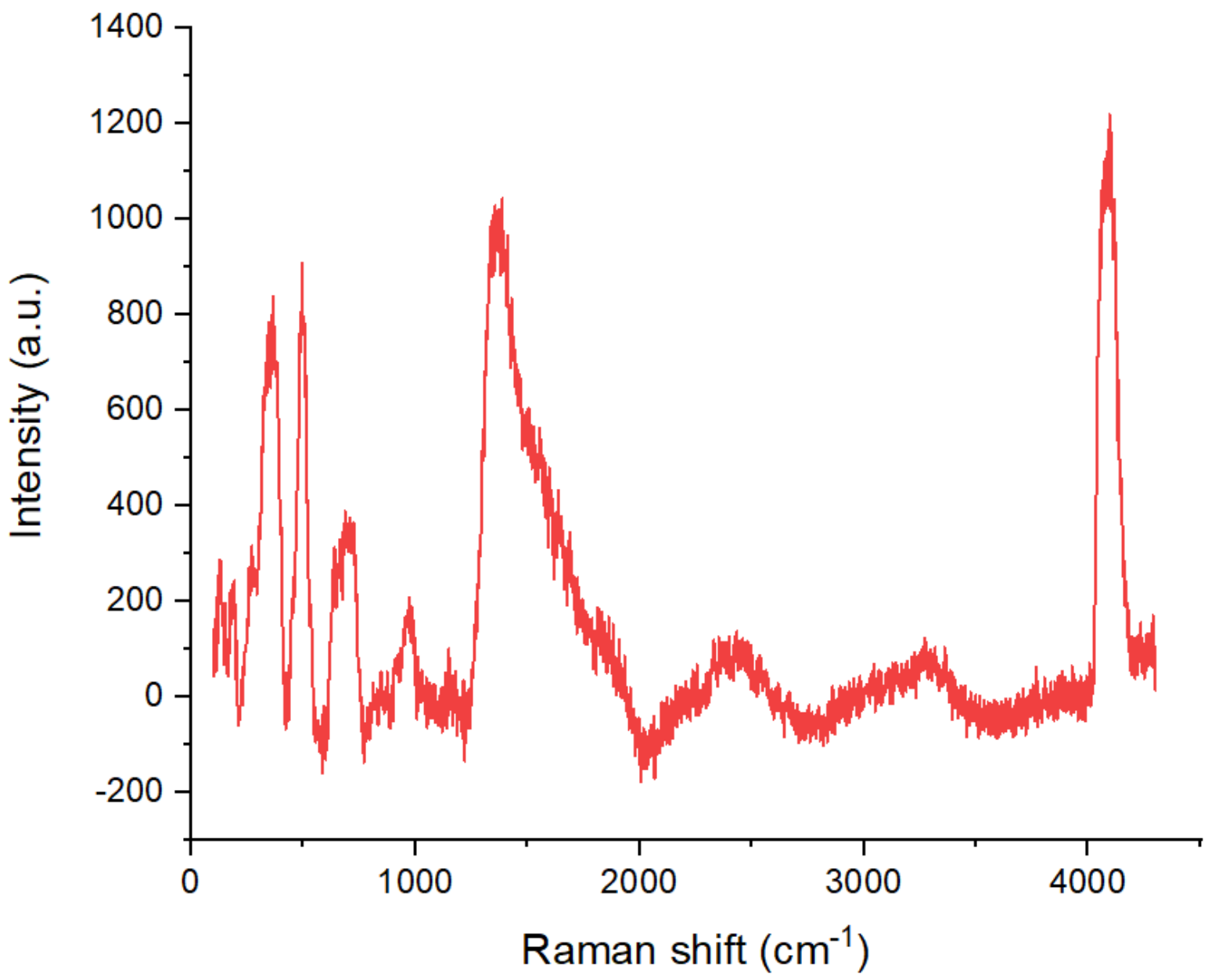

Figure S2.5. RAMAN spectrum of the Fe_3O_4@SiO_2 core-shell nanoparticles (λ_{exc} = 785 nm).

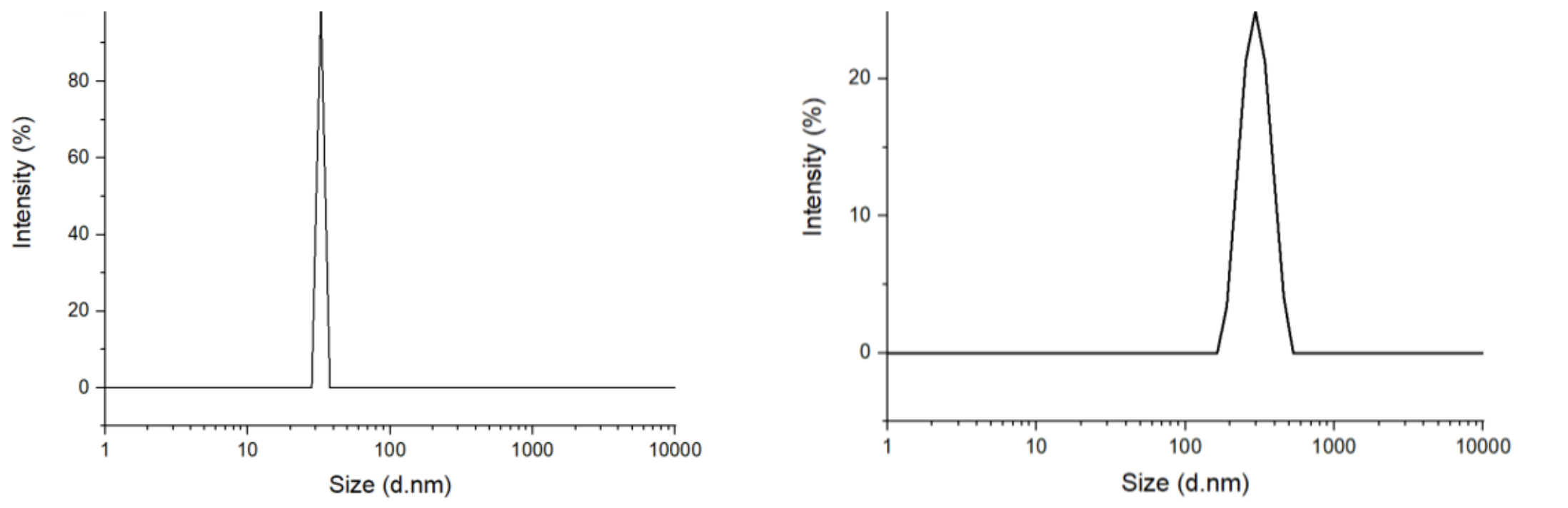

(a) **(b)**

Figure S2.6. DLS of precursor nano-particles used, with perceptual distributions for Fe_3O_4 core (in MeOH) and for the Fe_3O_4@SiO_2 core-shell nanoparticles (in oleic acid:MeOH dispersion)

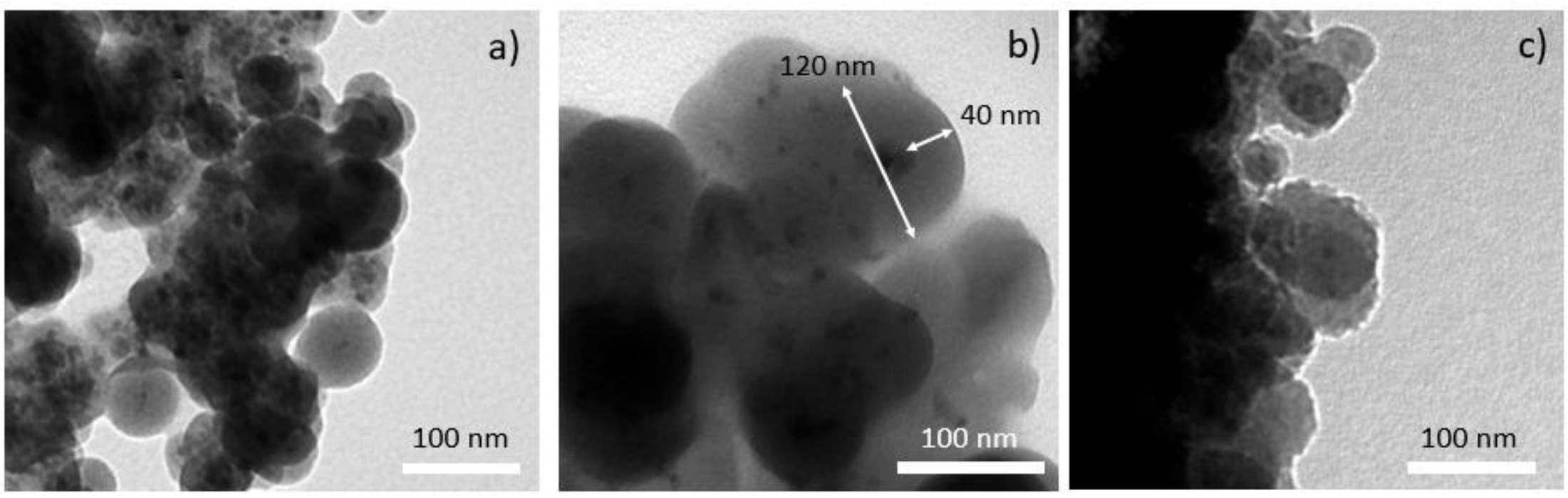

Figure S2.7. TEM micrographs of Fe_3O_4@SiO_2 core-shell nanoparticles (a and b), and **6**@Fe_3O_4@SiO_2 (c)

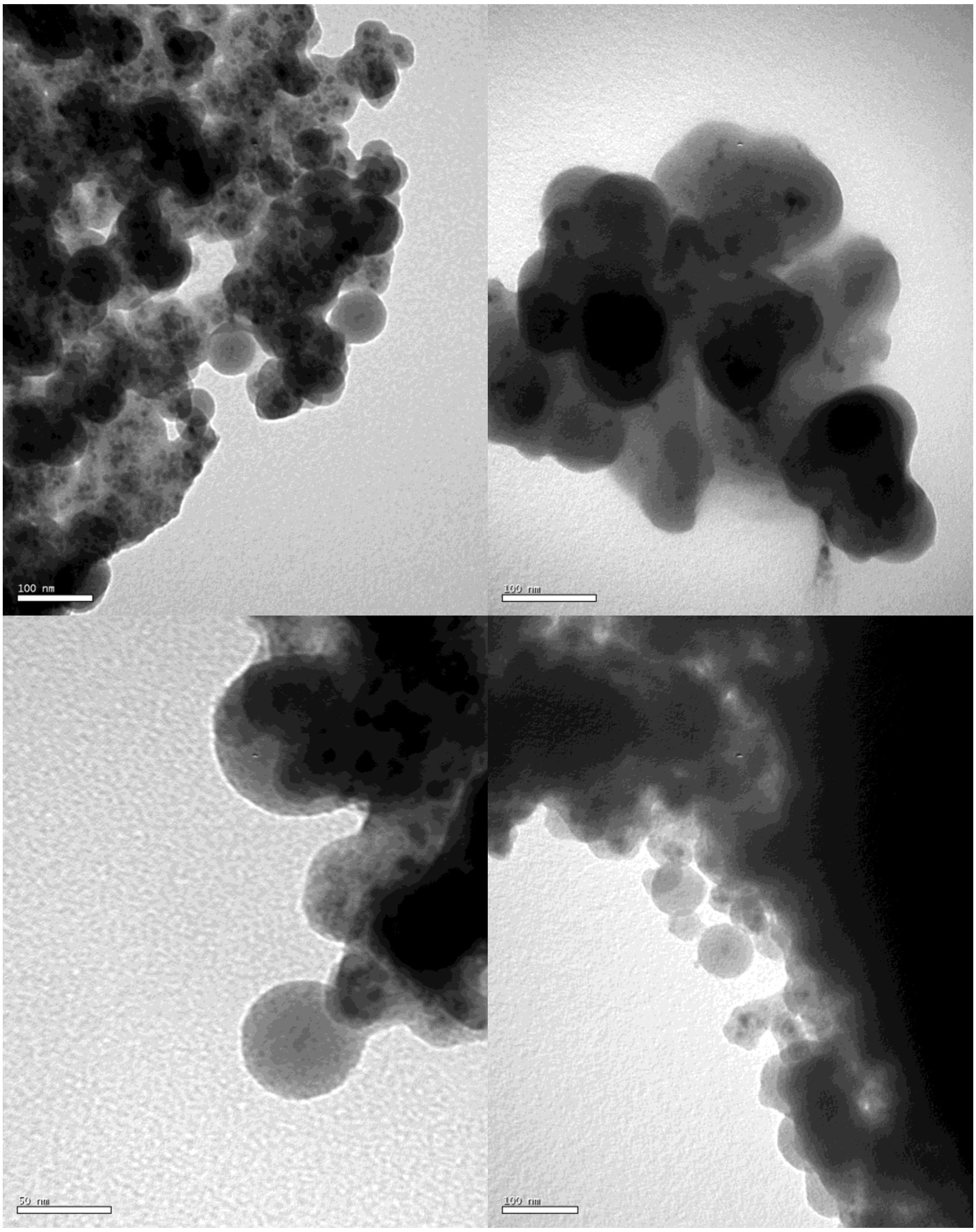

Figure S2.8. Additional TEM micrographs of the **6**@Fe_3O_4@SiO_2 core-shell nanoparticles.

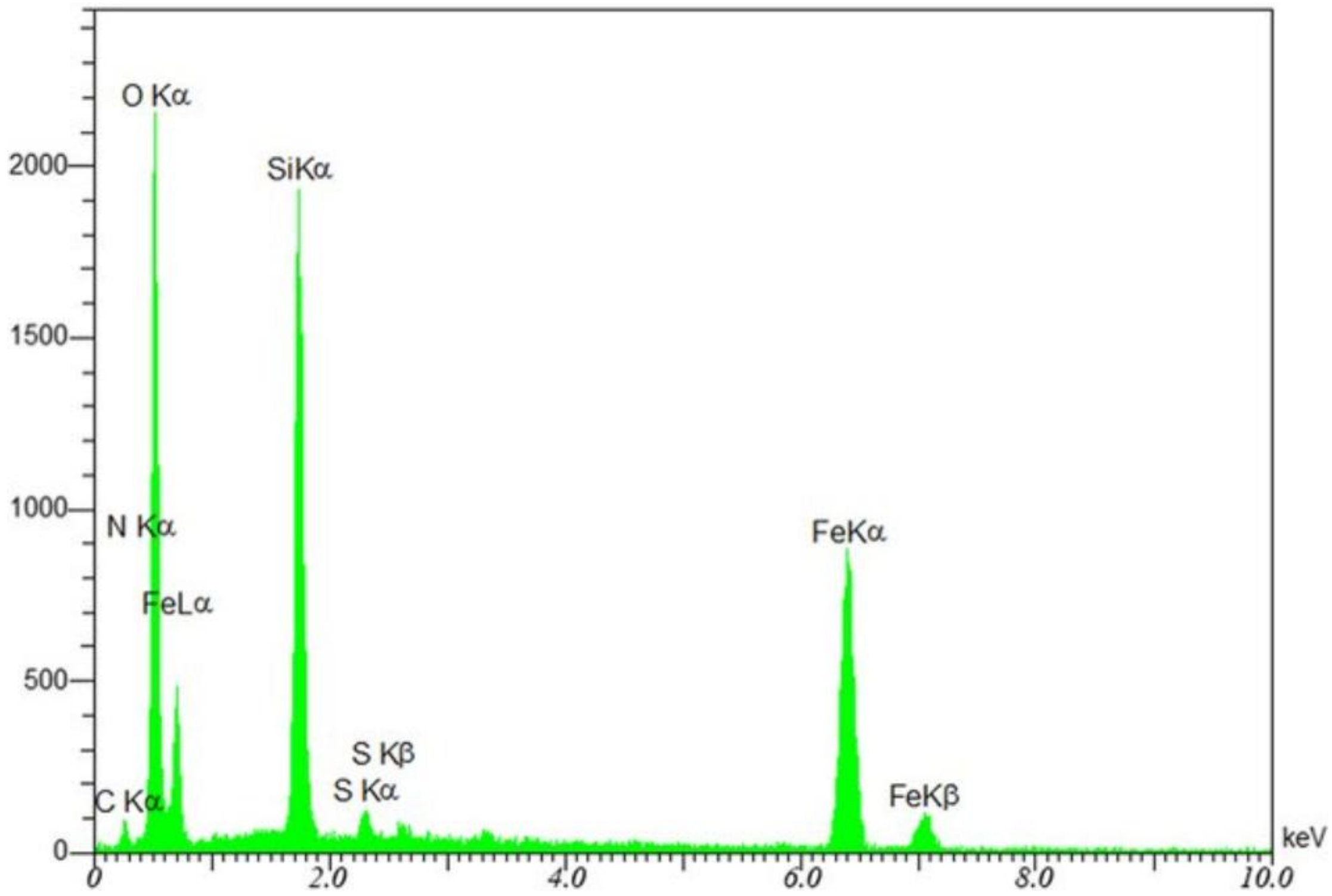

Figure S2.9. EDX analysis of the **6**@Fe_3O_4@SiO_2 core-shell nanoparticles corresponding to the TEM analysis above.

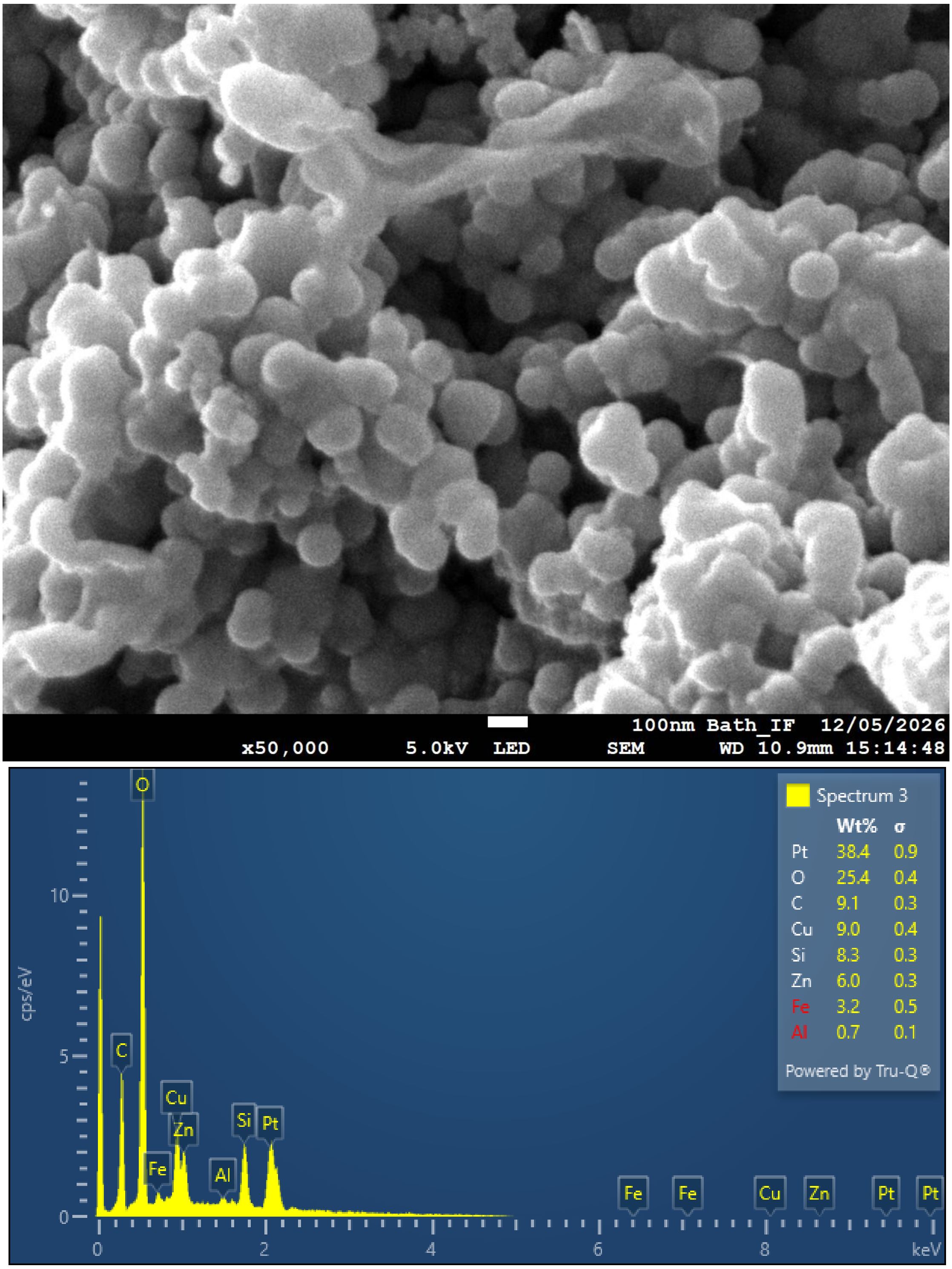

Figure S2.10. Cryo-SEM and corresponding EDX analysis of the **6**@Fe_3O_4@SiO_2 analysed as platinum-coated nanoparticles.

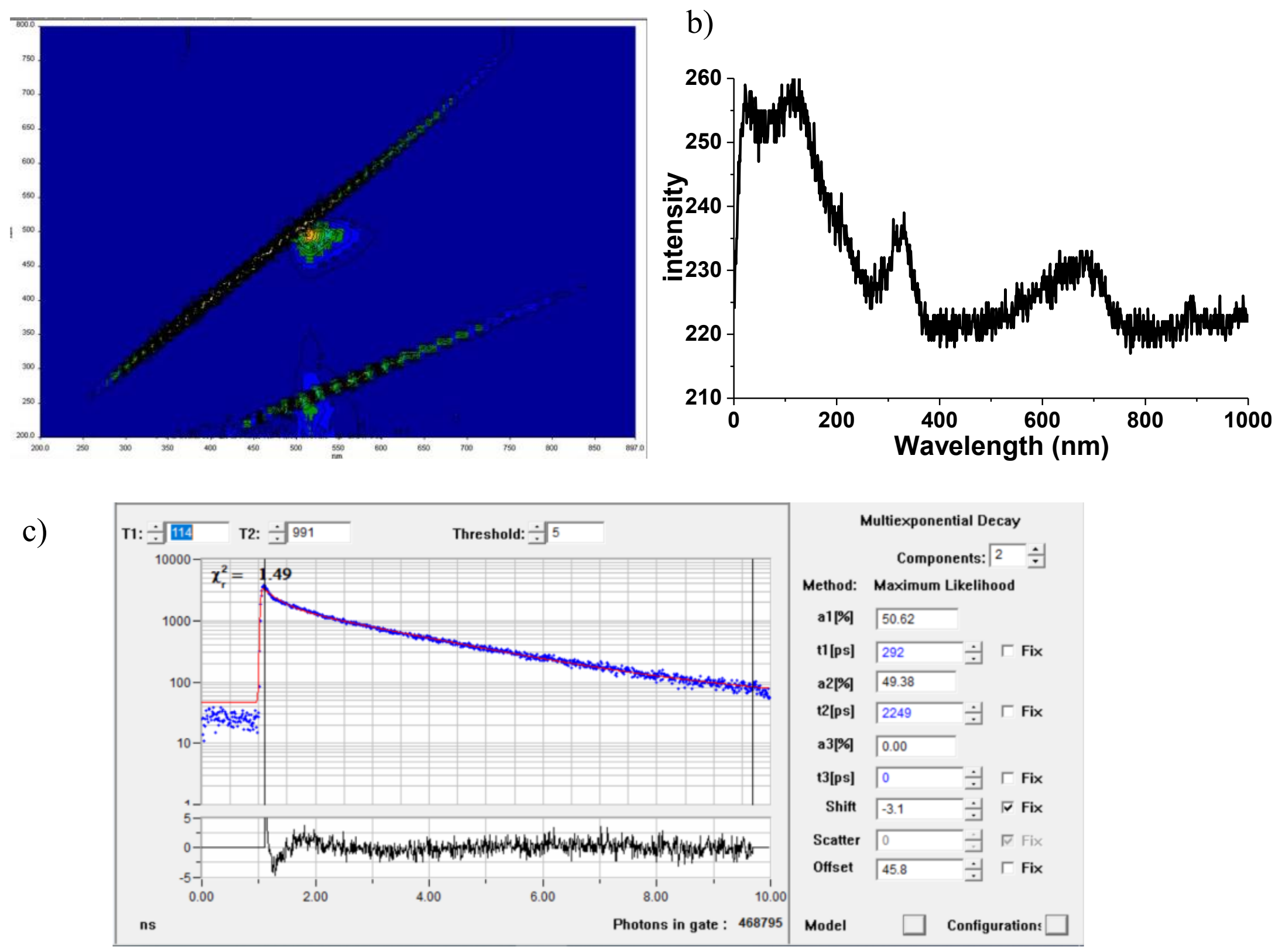

Figure S2.11. (a) 2D excitation-emission mapping of **6**@Fe_3O_4@SiO_2 nanoparticles (1 mg/mL dispersed in water); (b) Two-photon fluorescence spectroscopy of **6**@Fe_3O_4@SiO_2 (1 mg/mL) dispersed in water at λ_{em} = 910 nm. (c) 2P TCSPC of **6**@Fe_3O_4@SiO_2 (1 mg/mL) dispersed in water at λ_{em} = 910 nm

3. Cell viability tests

General cell culture methods:

Cell lines used in live cell imaging include PC-3, Hela, MCF-7 and CHO which are all available from ATCC Repository [9] (https://www.lgcstandards-atcc.org/search?title=All%20Products#sort=relevancy&f:contentTypeFacetATCC=[Products]). These are tested for identity and contaminating microorganisms that commonly occur in culture, so they are unlikely to be contaminated or misidentified, unless so stated in the accompanying literature. The cell culture collections from this repository routinely authenticate their cell line stocks and provide a Certificate of Analysis, including an STR profile, for each line they produce. Once the cultures are received, they are cultured and maintained according to the guidelines for the use of cell lines in biomedical research. [10] https://www.nature.com/articles/bjc2014166.pdf

Healthy cells (control) involved established fibroblast cells routinely in culture at the University of Bath, Department of Pharmacy and Pharmacology, University of Bath). The FEK-4 line is widely used in the Pharmacy and Pharmacology Department at Bath and are a kind gift from Prof. Rex Tyrrell's laboratory. This cell line is cultured at the University of Bath according to published protocols and information therein. [11-13]

Cells were normally frozen at -196 °C in liquid nitrogen until required, then thawed quickly and incubated after the addition of fresh media at 37 °C under 5% carbon dioxide environment. Eagle's Minimum Essential Medium (EMEM), Park Memorial Institute (RPMI) medium and Dulbecco's Modified Eagle's Medium (DMEM) were used as culture media. All media contained activated foetal calf serum (FCS) (10% for HeLa, PC-3, Fek-4 and CHO), 0.5% penicillin/streptomycin (10,000 IU mL^{-1}/10,000 mg mL^{-1}) and 2.5% L-glutamine.

Cell subculture was performed once or twice per week depending on the confluence of the cells in the flask, the supernatant was aspirated. All attached cells were washed twice using phosphate buffered saline (PBS, 2 x 10 mL), (all solvents, buffer solutions and media were warmed to 37 °C in the water bath prior to addition) 3 mL of 0.25% trypsin in PBS was subsequently loaded and incubated at 37 °C for 5 minutes. After trypsinisation, 6 mL serum medium was added to neutralise the excess trypsin and solution was centrifuged at 1000 rpm for 5 minutes. Afterwards, the supernatant was aspirated and resuspended with 5 mL serum medium. Cells were counted in a haemocytometer and seeded appropriately for different applications.

General details for MTT assays

Cells were cultured at 37 °C in a humidified atmosphere in the air with 5% CO_2 and harvested once >70% confluence had been reached. PC3 (prostate cancer cells) were cultured in RPMI (Roswell Park Memorial Institute) 1640 medium and HeLa cells (cervix cancer cells) were cultured in EMEM (Eagle's Minimum Essential Medium) medium. Both media contained 10% foetal calf serum (FCS), 0.5% penicillin/streptomycin (10,000 IU mL^{-1}/10,000 mg mL^{-1}) and 1% 200 mM L-Glutamine. All steps were performed in absence of phenol red.

Once cells reached more than 70% confluence, the supernatant containing dead cell matter and excess protein were aspirated. The live adherent cells were then washed with 10 mL of phosphate buffer saline (PBS) solution twice to remove any remaining media. Cells were incubated in 3 mL of trypsin solution (0.25% trypsin) for 5 to 7 min at 37 °C. After trypsinisation, 6 mL of medium containing 10% serum medium was added to inactivate the trypsin and the solution was centrifuged for 5 min (1000 rpm, 25 °C). The supernatant liquid was aspirated, and 5 mL of cell medium (10% FCS) was added to the cell matter left behind. Cells were counted using a haemocytometer and then seeded as appropriate.

After cell splitting, cells were seeded on a sterile 96 well plate (7×10^3 cells per well) and incubated for 48 hours to adhere. Compounds were subsequently loaded at different concentration into wells and cultured for another 48 hours. The concentration used were ranging from 250 µM (1% DMSO, 99% culture media (10% FCS)) to 100 µM, 50 µM, 10 µM, 1 µM, 0.5 µM, 0.1 µM and 1 nM. After 48 hours incubation, cells were washed three times with PBS (1 mL) and 3-(4, 5-dimethylthiazol-2-yl)-2, 5-diphenyltetrazolium bromide (MTT) was added (0.5 mg/ mL, 1:9 PBS: serum-free medium (SFM)) followed by a two-hour incubation. After aspiration, DMSO (100 µL) was added and 96 well plates were read by an ELISA plate reader, Molecular Devices Versa Max (BN02877). The absorption wavelength was at 570 nm and 630 nm wavelength was used as a reference.

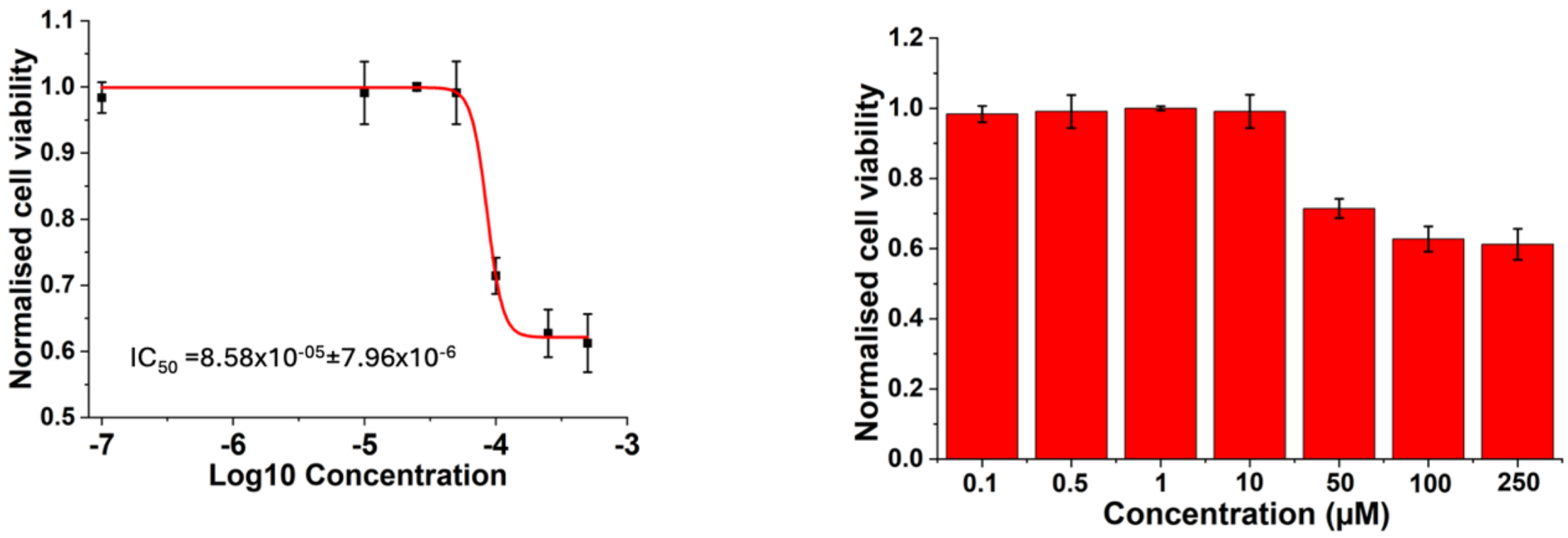

(a)

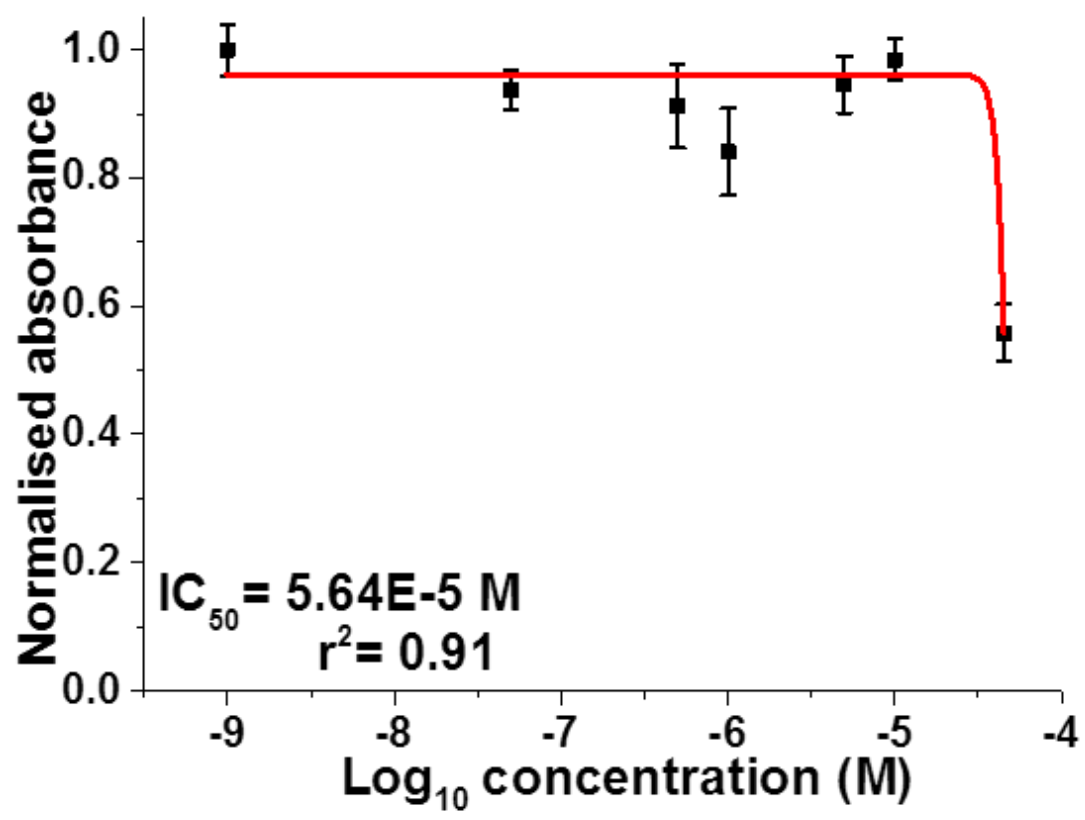

(b)

Figure S3.1. 48 h MTT assays of HeLa cells **(a)** and PC-3 cells **(b)** treated with **1**. Error bars stand for standard error calculated from the six repeats.

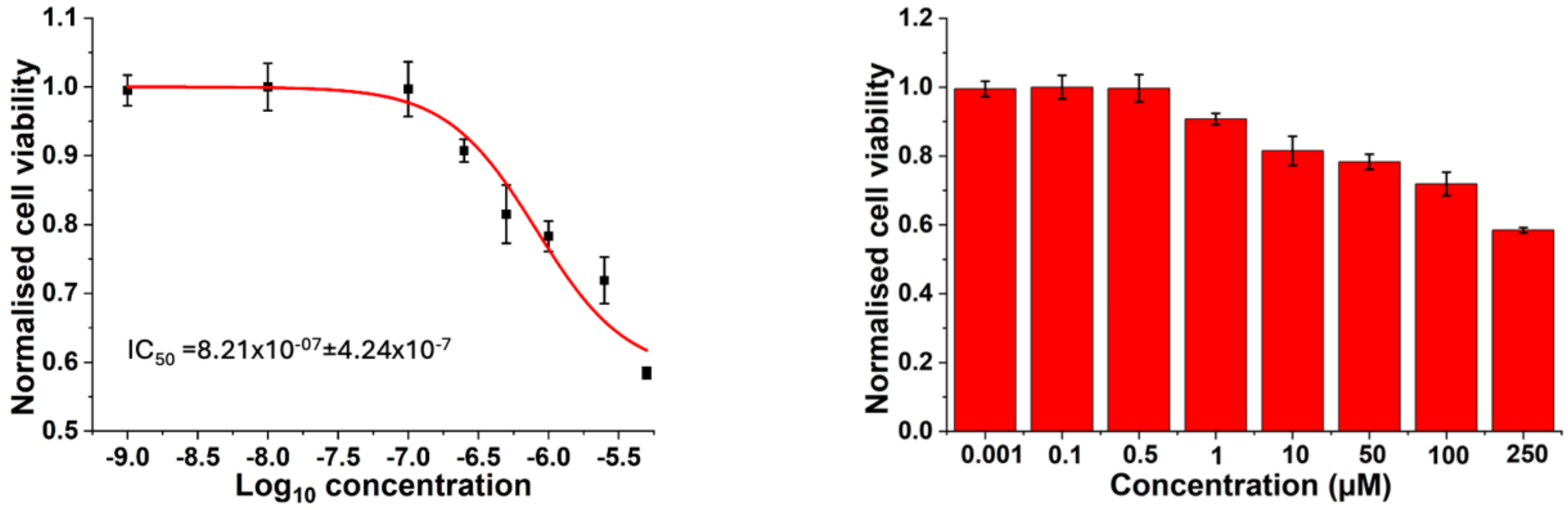

Figure S3.2. MTT assays of HeLa cells treated with **2** over 48 h, showing the dose response curve for **2**. Error bars stand for standard error calculated from the six repeats. Close analysis in context of concentrations relevant for molecular imaging, indicated a general trend suggestive compound **2** was generally nontoxic over the timeline and concentration range used, e.g. at maximum concentration of 5×10^{-6} M, the cell survival was still ca. 58.4 %. However, the initial drop in viability caused by compound **2** appeared at the concentration of 2.5×10^{-7} M, the survival rate of cells then reduces slowly followed by a sharper decrease at the concentration of ca. 2.5×10^{-4} M. Absorption data collected for each compound was compared with control, statistical significance was determined by Student's t-test (*P<0.005, **P<0.05).

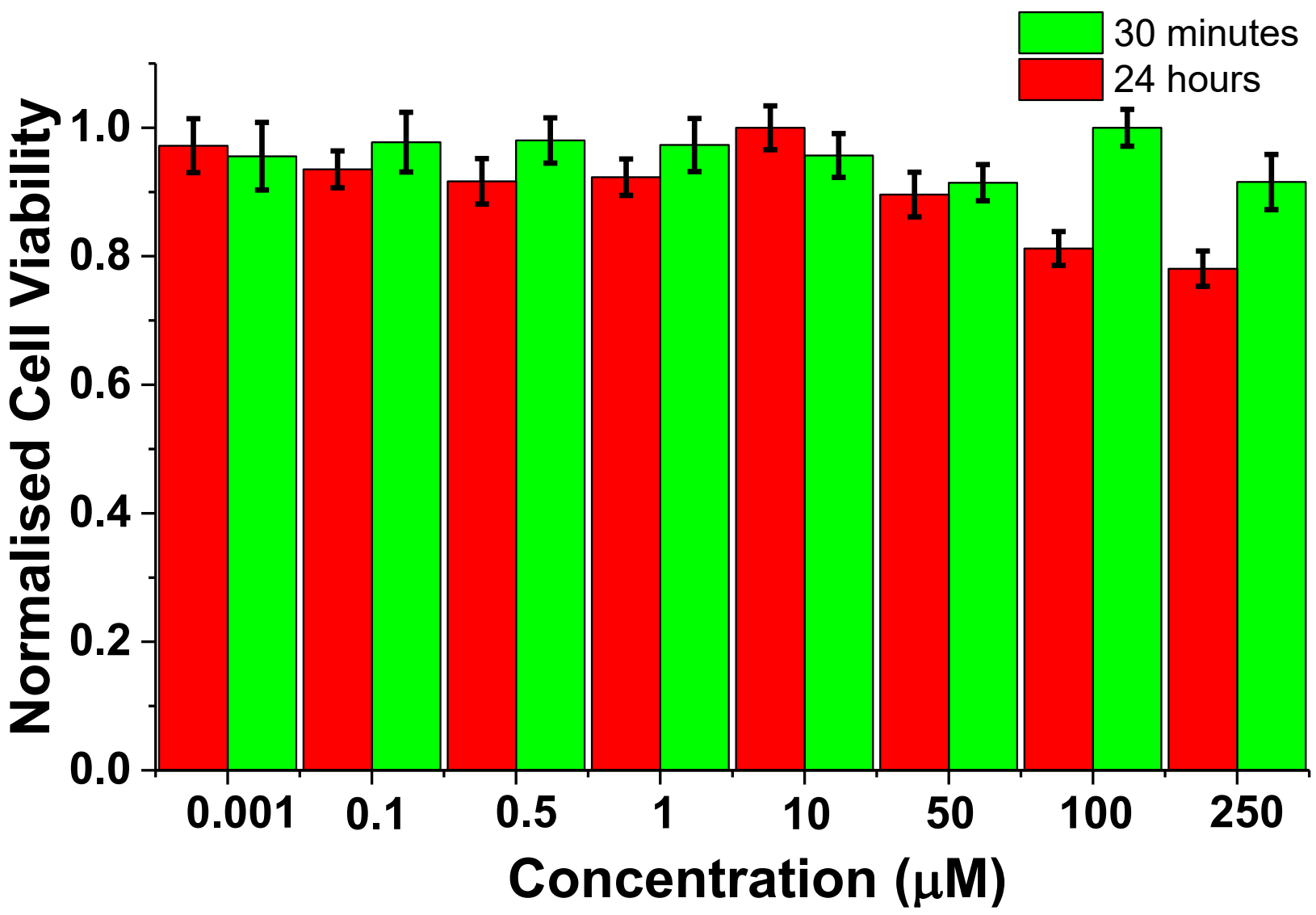

Figure S3.3. MTT assays of HeLa cells treated with **3**. Cell viability graph for **3**. Error bars stand for standard error calculated from the six repeats. Absorption data collected for each compound was compared with control, statistical significance was determined by Student's t-test (*P<0.005, **P<0.05).

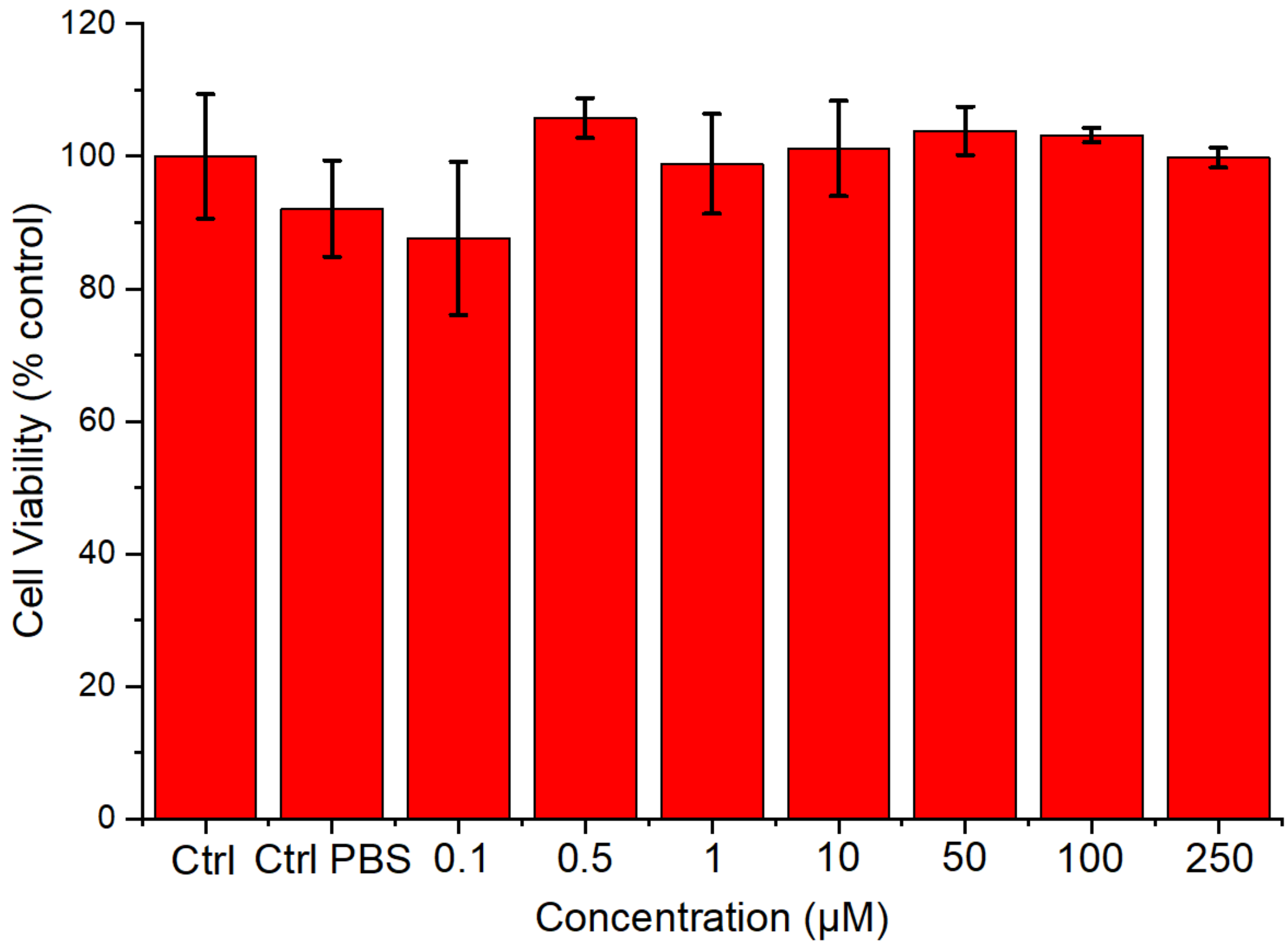

Figure S3.4. (a) 48 h MTT assays of PC-3 cells treated with compound **4**. Absorption data collected for each compound was compared with control (1% DMSO or 1% PBS), statistical significance was determined by Student's t-test (*P<0.005, **P<0.05).

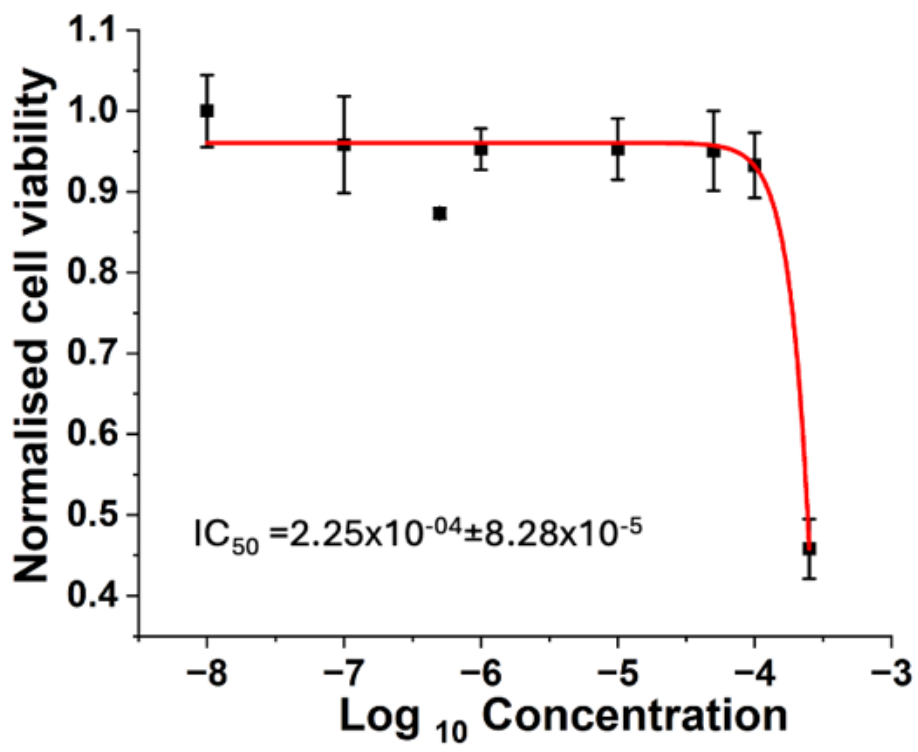

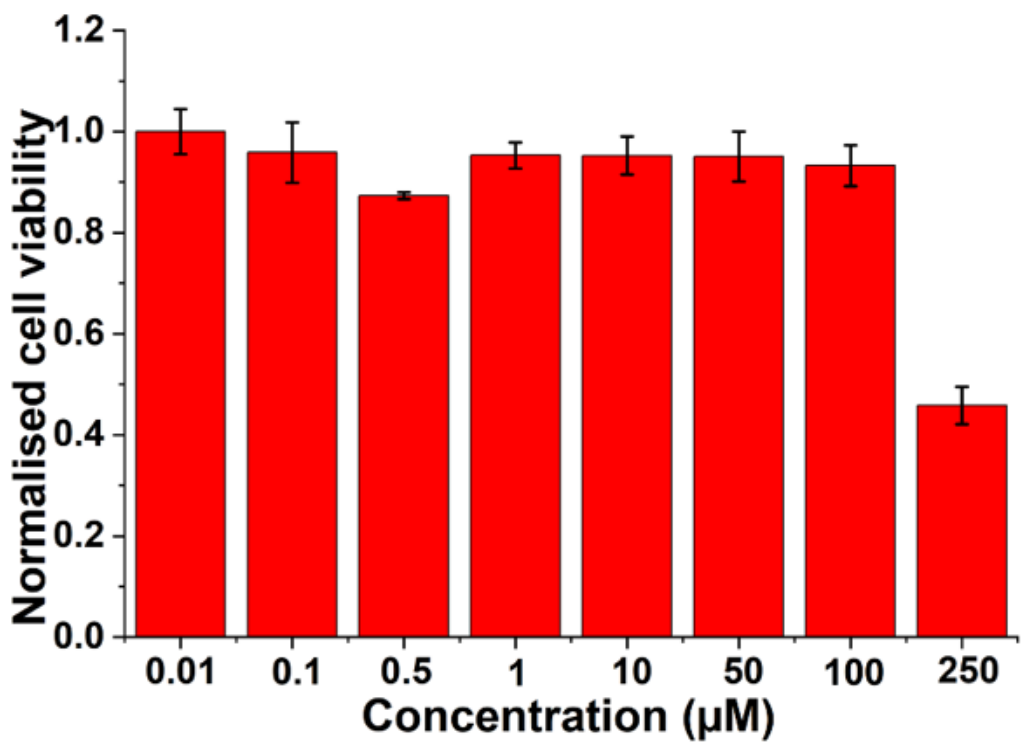

Figure S3.5. MTT assays of HeLa cells treated with **5**. Dose response curve. Error bars stand for standard error calculated from the six repeats. Absorption data collected for each compound was compared with control, statistical significance was determined by Student's t-test (*P<0.005, **P<0.05).

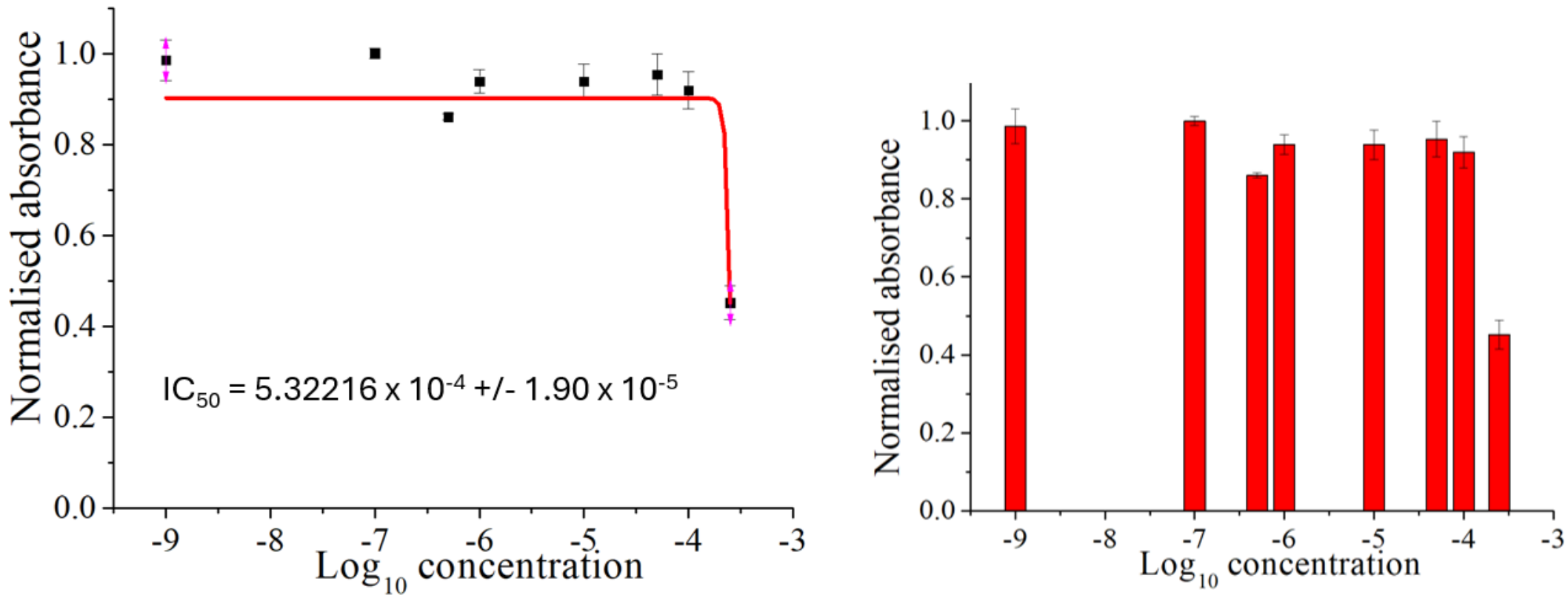

(a)

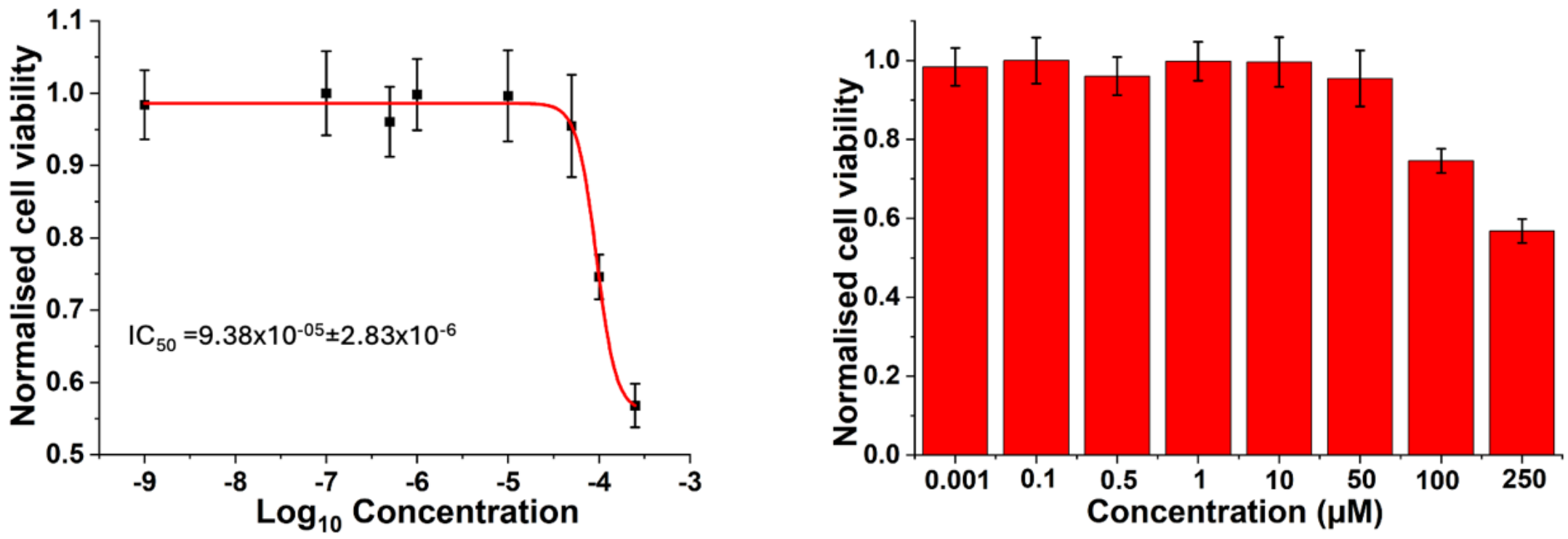

(b)

Figure S3.6. MTT assays of HeLa (a) and PC-3 (b) cells treated with **6**. Dose response curve at 48 h observation. Error bars stand for standard error calculated from the six repeats. Note that in HeLa cells used for imaging, and over the 24 h survey, there was more than 90% cell survival at 1×10^{-6} M concentration when cells were treated with compound **6** whereas only 78% of cells remained when treated with compound **2** at the concentrations and timescales used for imaging. Absorption data collected for each compound was compared with control, statistical significance was determined by Student's t-test (*P<0.005, **P<0.05).

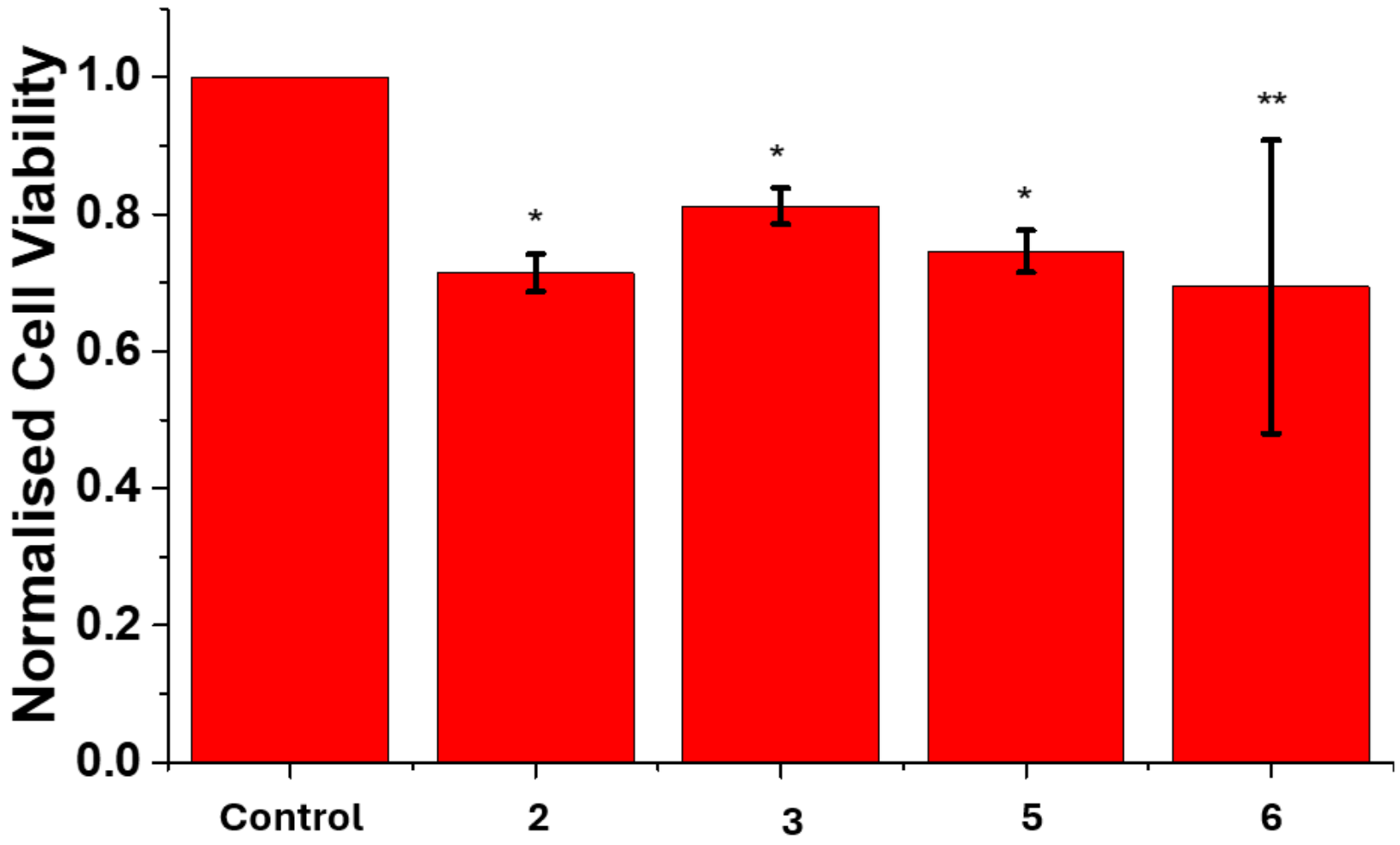

(b)

Figure S3.7. (a) MTT assays of PC-3 cells: Normalised cell viability of PC-3 cells treated with 100 μM individual compounds that were imaged at 37 °C over 24 hours. Absorption data collected for each compound was compared with control (1% DMSO), statistical significance was determined by Student's t-test (*P<0.005, **P<0.05).

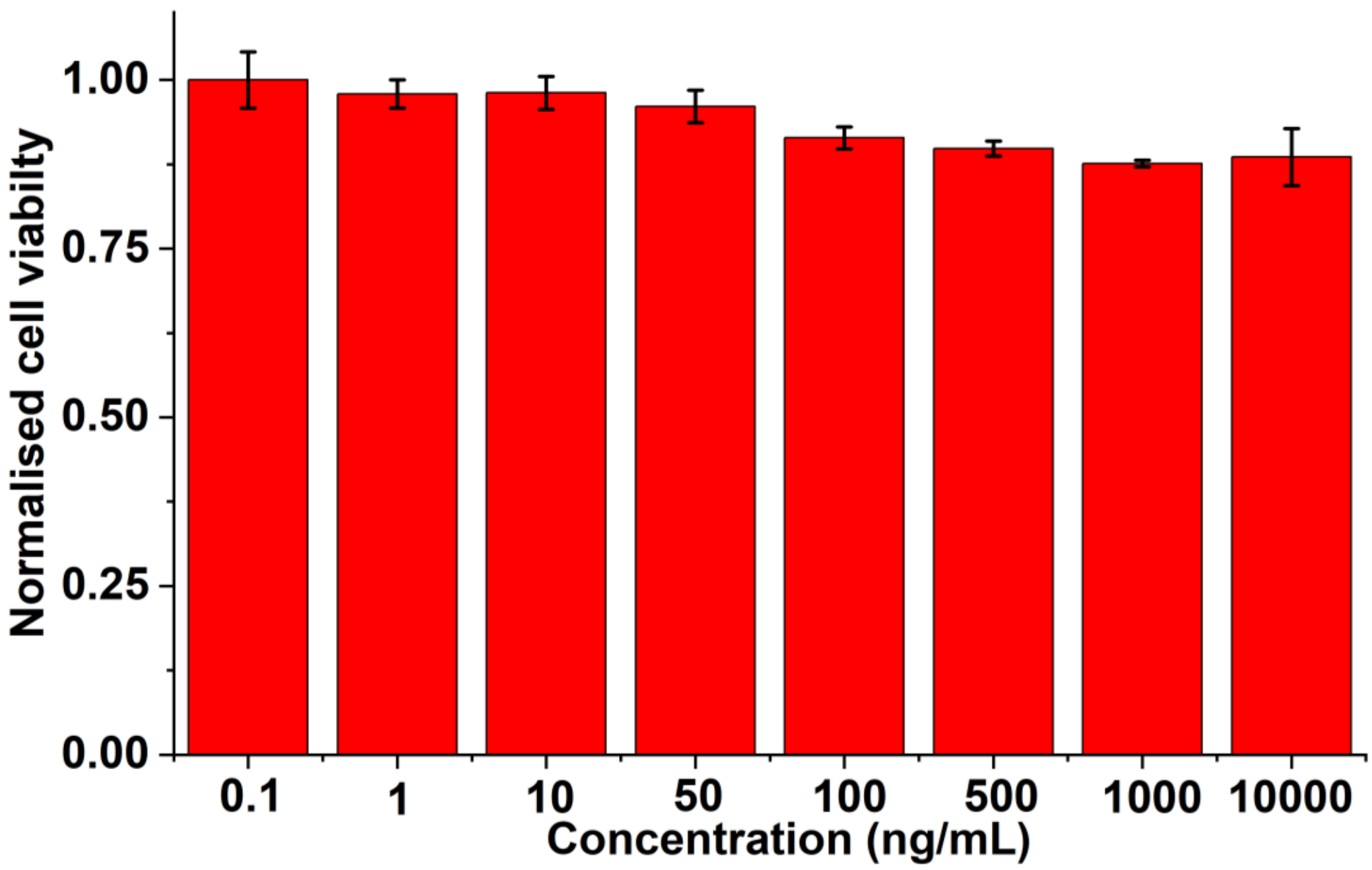

Figure S3.8. MTT assays of PC-3 cells treated with beta-D-glucan: cell viability graph for 48 h observation. Error bars stand for standard error calculated from the six repeats. Statistical significance was determined by Student's t-test (*P<0.005, **P<0.05).

Experimental details for MTT assays under photoactivation with UVA light

Cells were seeded into 18 × 3.5 cm Petri dishes at a density of (3 x 10^5 PC-3 cells per dish in a final volume of 5 mL complete culture medium and incubated for 48 h at 37°C in a humidified atmosphere containing 5% CO_2 to allow cell attachment. Following incubation, cells were treated with the indicated compounds or experimental conditions (Ctrl, Ctrl + UVA, Sample **1-6** + UVA, and Sample 6@Fe_3O_4@SiO_2 NP + UVA) at a final concentration of 100 µM in molecular compound **1-6** (and in the case of 6@Fe_3O_4@SiO_2 100 µM formulated with 1 mg/mL Fe_3O_4@SiO_2) in presence of 1% DMSO and incubated for 20 minutes. Then cells were washed once with PBS and replenished with 3 mL serum-free medium prior to UVA exposure. Cells were then irradiated with UVA light (340 nm) for 20 min. Immediately after irradiation, 1 mL MTT assay solution was added to each dish to obtain a final MTT concentration of 0.5 mg/mL, followed by incubation for 3 h at 37°C in the dark to allow formation of formazan crystals. After incubation, the culture medium was carefully removed without disturbing the crystals, and 700 µL DMSO was added to each dish to dissolve the formazan crystals. Dishes were gently shaken for 10 min at room temperature to ensure complete solubilization. Subsequently, three 200 µL aliquots from each dish were transferred into a 96-well plate for absorbance measurement. Absorbance was measured at 570 nm using a microplate reader, with a reference wavelength of 630–690 nm used for background correction where applicable.

MTT analysis demonstrated that cell viability remained above 95% in all experimental groups following UVA irradiation and compound treatment. Although slight reductions in viability were observed compared with the untreated control, no substantial cytotoxic effects were detected under these conditions. UVA exposure alone reduced viability to approximately 96.9%, while compound-treated groups ranged from 95.6% to 97.3% viability. Overall, the tested compounds exhibited promising biocompatibility at 100 µM under the UVA irradiation conditions applied.

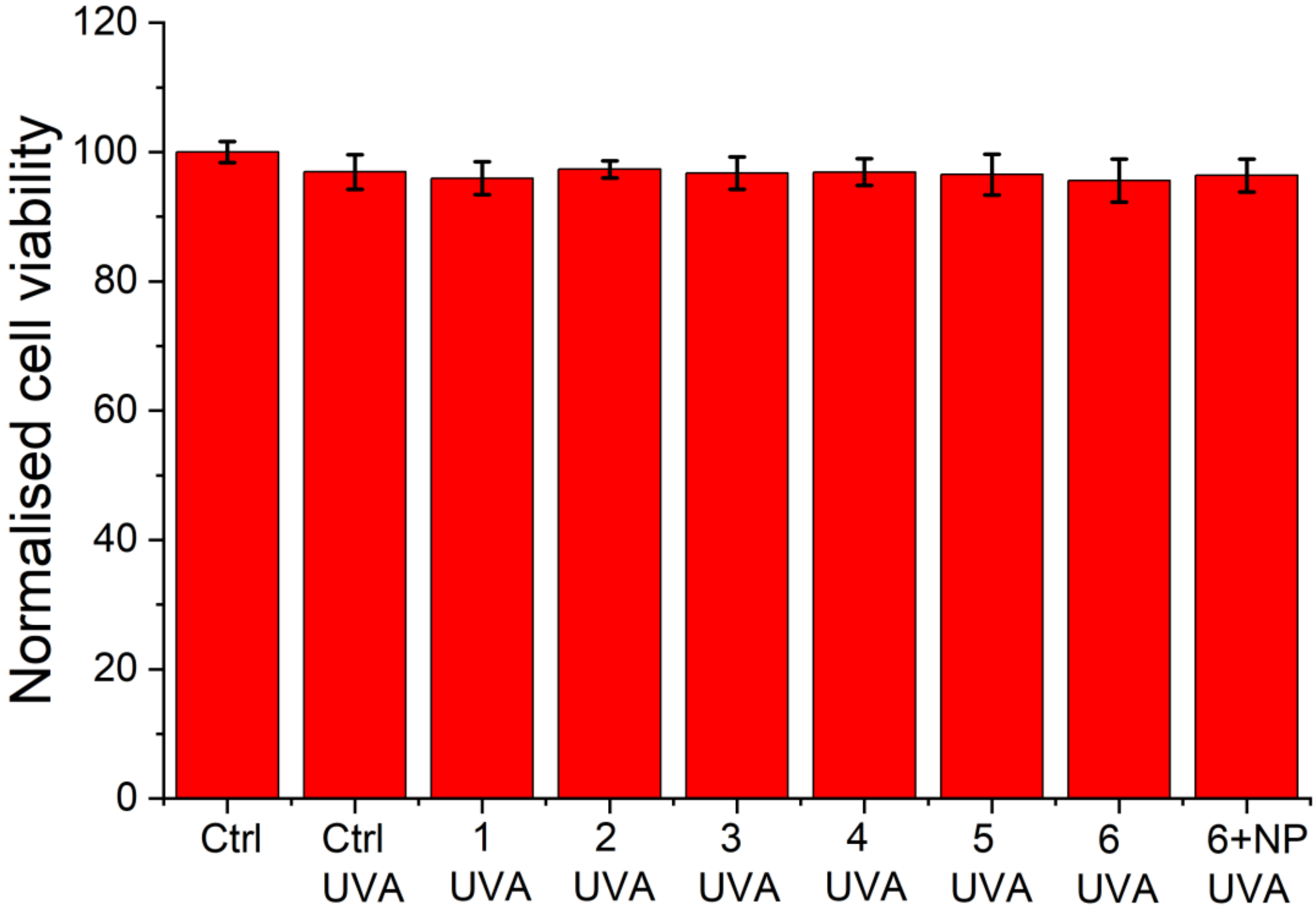

Figure S3.9. Normalised cell viability of PC-3 cells treated with 100 μM individual compounds **1-6** in 1 % DMSO in serum free medium (and in the case of 6@Fe_3O_4@SiO_2 100 μM, 1 % DMSO compound **6** in serum free medium containing 1 mg/mL Fe_3O_4@SiO_2) at 37 °C, over 20 minutes under UVA irradiation, as described above. (b) Absorption data collected for each compound was compared with control (1% DMSO), statistical significance was determined by Student's t-test (*P<0.005, **P<0.05).

4. Laser scanning confocal fluorescence microscopy

Fluorescence microscopy images were captured on a Nikon Eclipse TE2000-E epifluorescence or a Zeiss LSM510META microscope at University of Bath, whereas at the Rutherford Appleton Laboratory a modified Nikon TE2000-U was utilised for correlated imaging. HeLa, PC-3 or CHO cells were cultured as above and plated in a glass bottomed petri dish (MaTek, 35 mm diameter and 1.5 mm thickness) at 1.5×10^5 - 2.5×10^5 cells per dish depending on the cell line and culture time. Briefly, HeLa cells required 12 hours to attach onto the dish, whereas CHO and PC-3 needed 48 hours to adhere. Prior to the microscopy observation, cultured cells were washed with Hank's solution (HBSS) (5×1 mL) and refilled with serum-free medium (990 µL). Prior to the probe labelling, a small volume of compound (10 mM or 1 mg/mL stock solution, 10 µL) in DMSO was loaded to make the final volume 1 mL at the appropriate concentration. The final concentration on the plate was usually at 100 µM with 1% - 2% DMSO depending on the compound solubility. After 15 min to 1-hour incubation at 37 °C or 4 °C, cells were washed with HBSS three times and refilled with fresh SFM (1 mL). Confocal images were captured immediately afterwards.

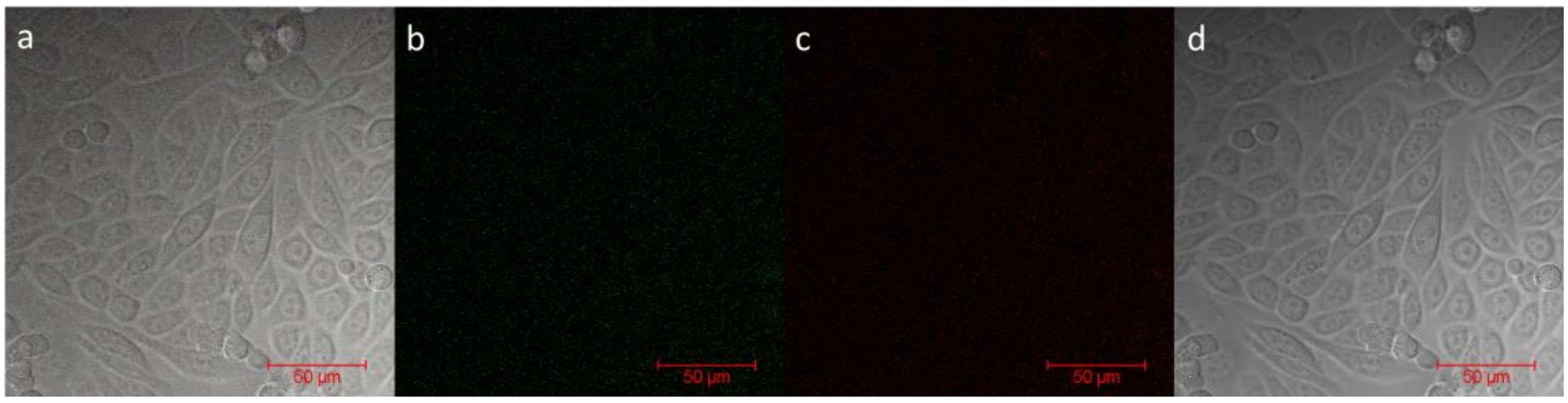

Figure S4.1. Confocal image of CHO cells incubated with 1% DMSO for 15 min at 37 °C; a is the overlay images of green channel, red channel and DIC; b is the green channel, compounds were excited at 488 nm, emission 516-530 nm; c is the red channel, compounds were excited at 488 nm, emission at 615-650 nm; d is the bright field channel.

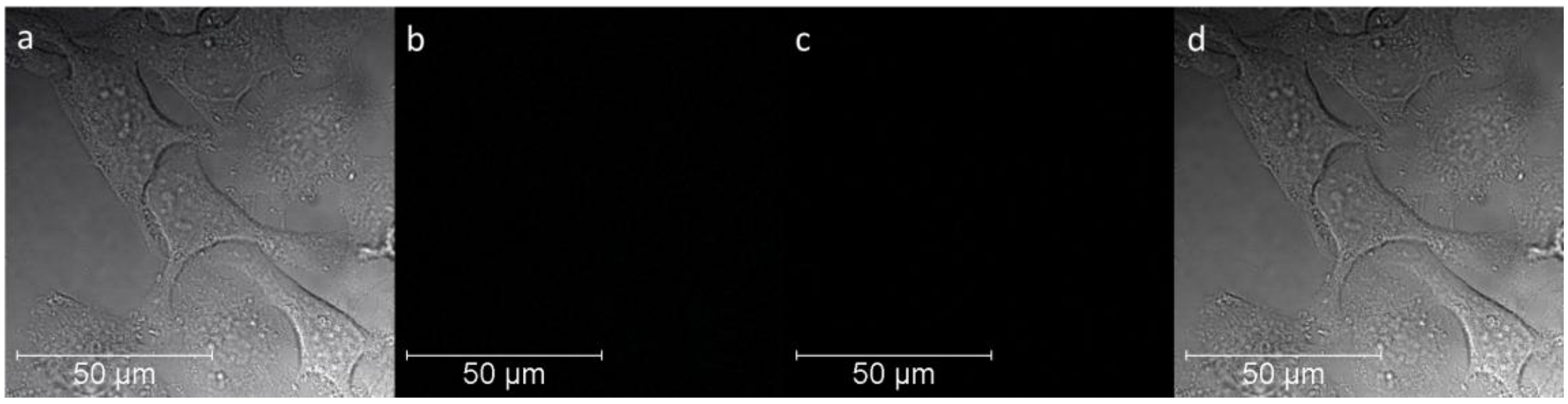

Figure S4.2. Confocal image of HeLa cells incubated with 1% DMSO for 15 min at 37 °C; a is the overlay images of green channel, red channel and DIC; b is the green channel, compounds were excited at 488 nm and emitted at 516-530 nm; c is the red channel, compounds were excited at 488 nm and emitted at 615-650 nm; d is the bright field channel.

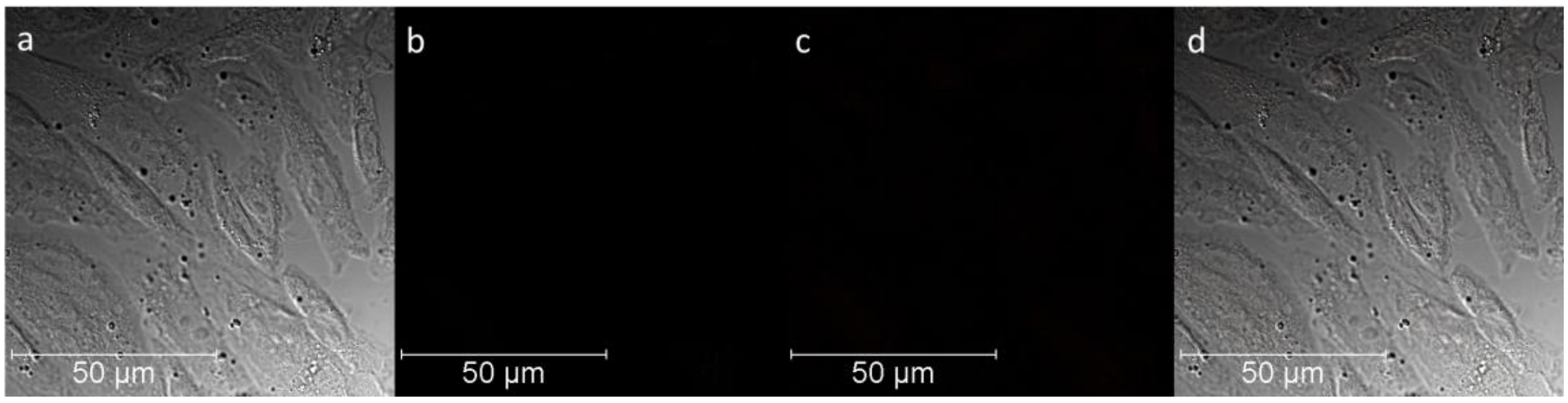

Figure S4.3. Confocal image of PC3 cells incubated with 1% DMSO for 15 min at 37 °C; a is the overlay images of green channel, red channel and DIC; b is the green channel, compounds were excited at 488 nm, emission 516-530 nm; c is the red channel, compounds were excited at 488 nm, emission at 615-650 nm; d is the bright field channel.

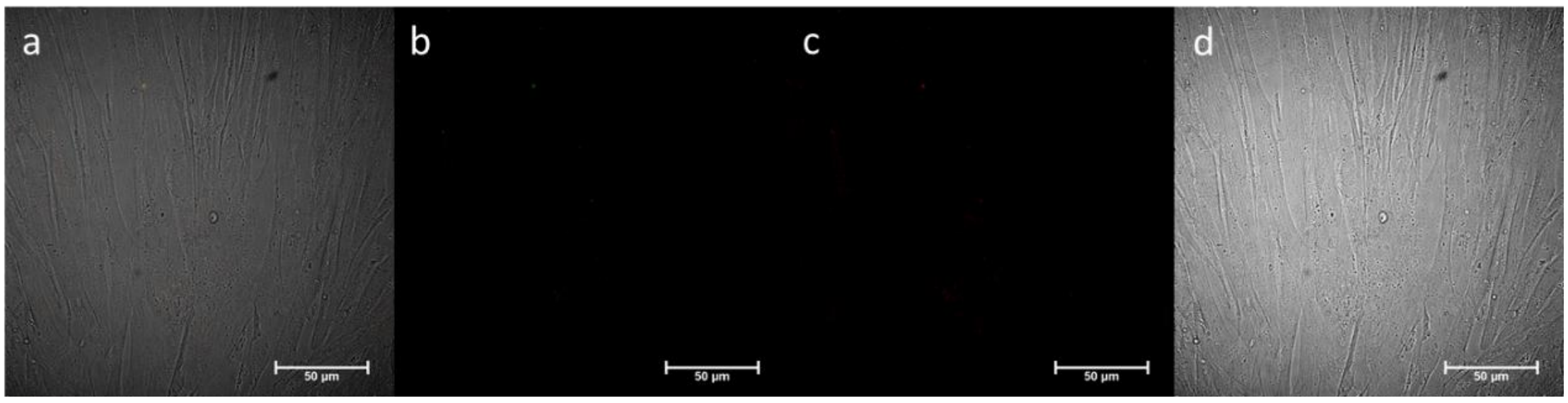

Figure S4.4. Confocal image of FEK4 cells incubated with 1% DMSO for 15 min at 37 °C; a is the overlay images of green channel, red channel and DIC; b is the green channel, excitation at 488 nm and emission at 516-530 nm; c is the red channel, excitation at 488 nm and emission at 615-650 nm; d is the bright field channel.

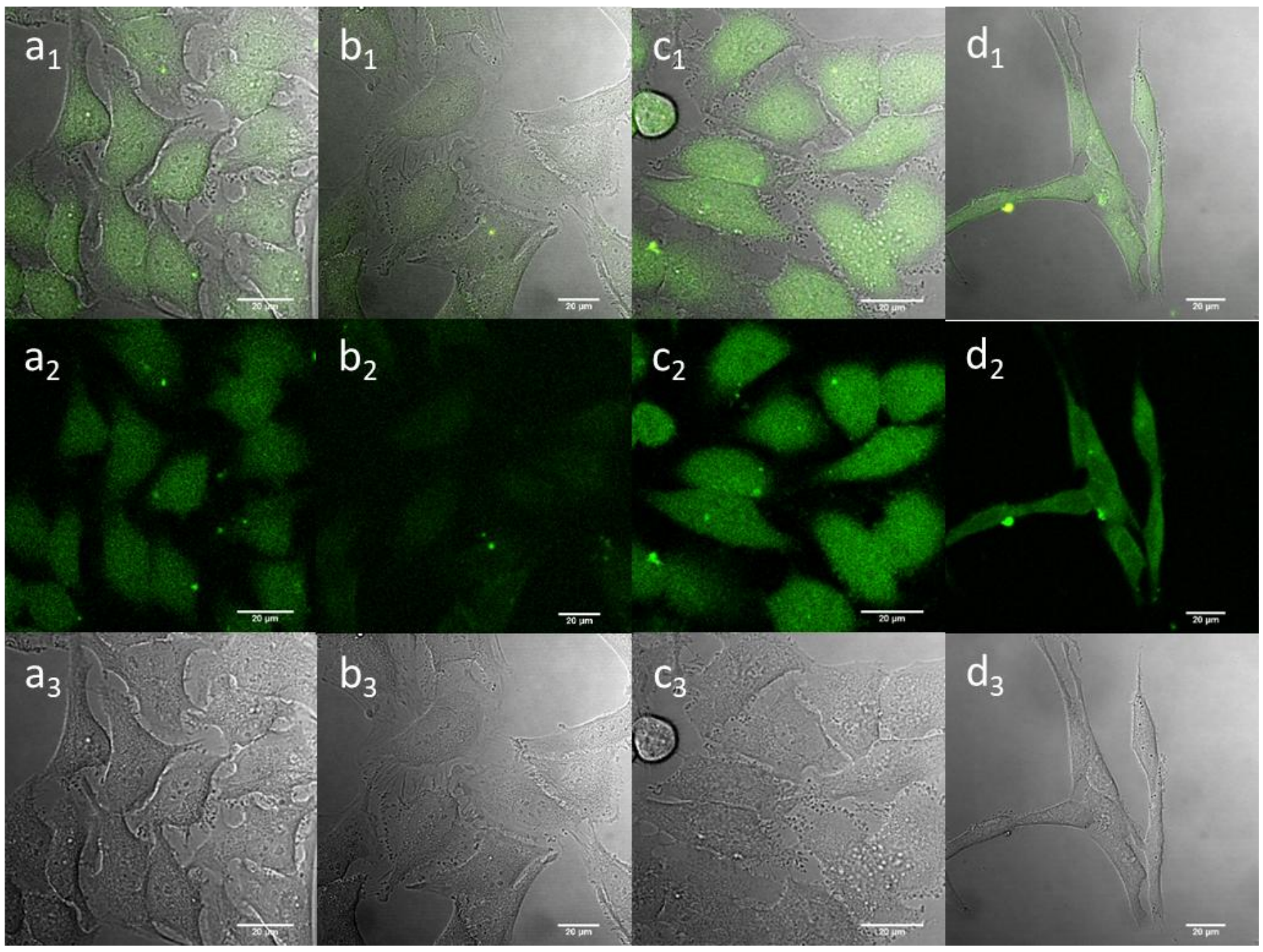

Figure S4.5. Confocal image of HeLa cells incubated with FITC based compounds at 100 µM for 15 min under various conditions. a) compound 1, at 37 ºC; b) compound

2, at 37 ºC; c) **1**@glucan, at 37 ºC; d) **2**@glucan, at 37 ºC. a_1-d_1 is the overlay images of green channel and DIC; a_2-d_2 is the green channel, compounds were excited at 488 nm and emitted at 516-530 nm; a_3-d_3 is the Differential interference contrast (DIC), which is a non-fluorescence channel.

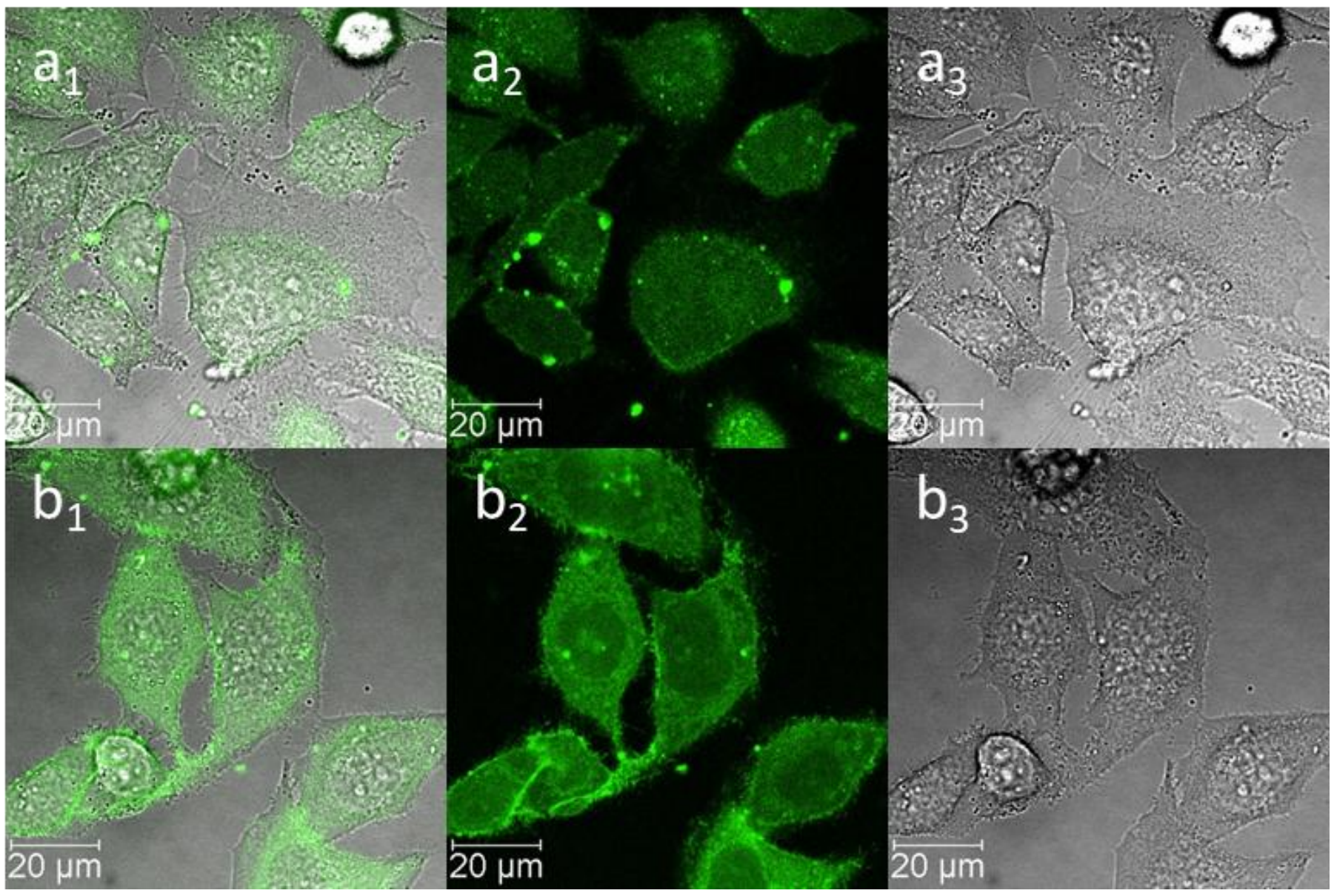

Figure S4.6. Confocal image of HeLa cells incubated with FITC-based compounds at 100 μM for 15 min under various conditions from another view of interest. a) compound **1**, at 37 ºC; b) compound **2**, at 37 ºC; a_1-b_1 is the overlay images of green channel and DIC; a_2-b_2 is the green channel, compounds were excited at 488 nm and emitted at 516-530 nm; a_3-c_3 is the DIC, which is a non-fluorescence channel.

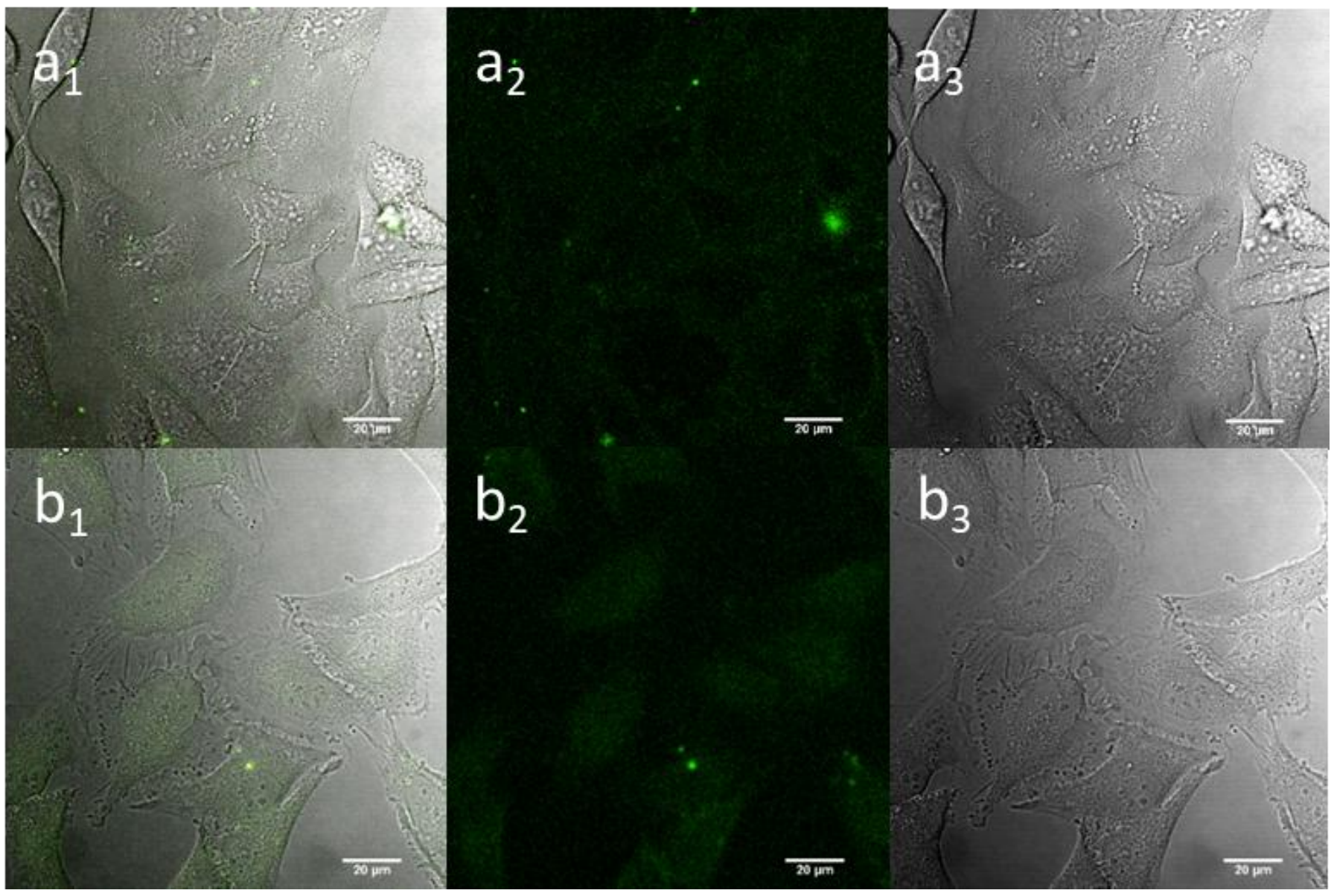

Figure S4.7. Confocal image of HeLa cells incubated with hybrid species at 100 μM for 15 min under various conditions. a) compound **1**@glucan, at 4 °C; b) compound **2**@glucan, at 4 °C; a_1-b_1 is the overlay images of green channel and DIC; a_2-b_2 is the

green channel, compounds were excited at 488 nm and emitted at 516-530 nm; a_3-b_3 is the bright field channel.

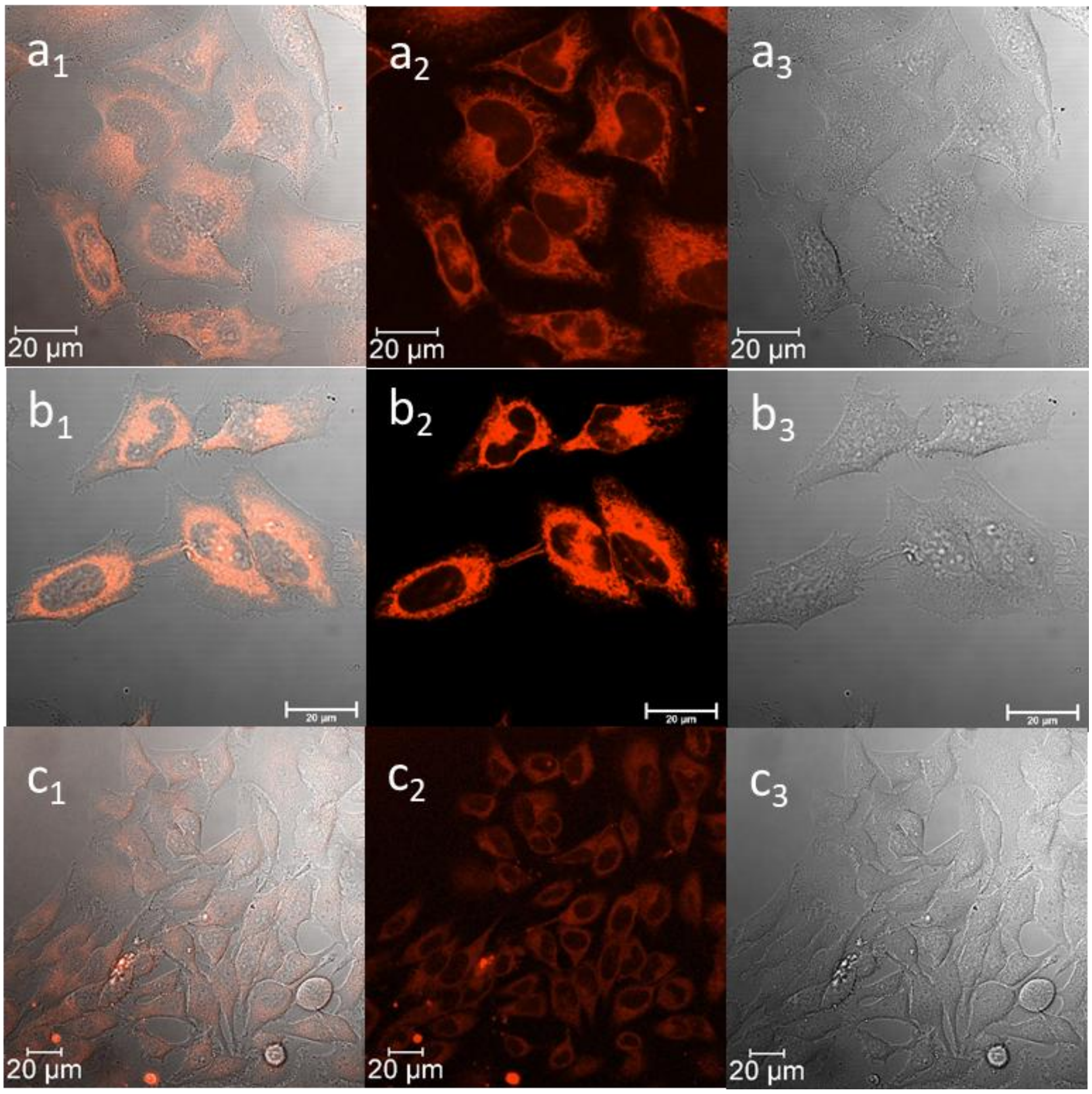

Figure S4.8. Confocal image of HeLa cells incubated with rhodamine-based compounds for 15 min under various conditions. a) 100 µM compound **3**, at 37 ºC; b) 200 µM 3@glucan, at 37 ºC; c) 100 µM compound **3**, at 4 ºC; a_1-c_1 is the overlay images of red channel and DIC; a_2-c_2 is the red channel, Excitation wavelength was 561 nm and emission at 615-650 nm; a_3-c_3 is the bright field channel.

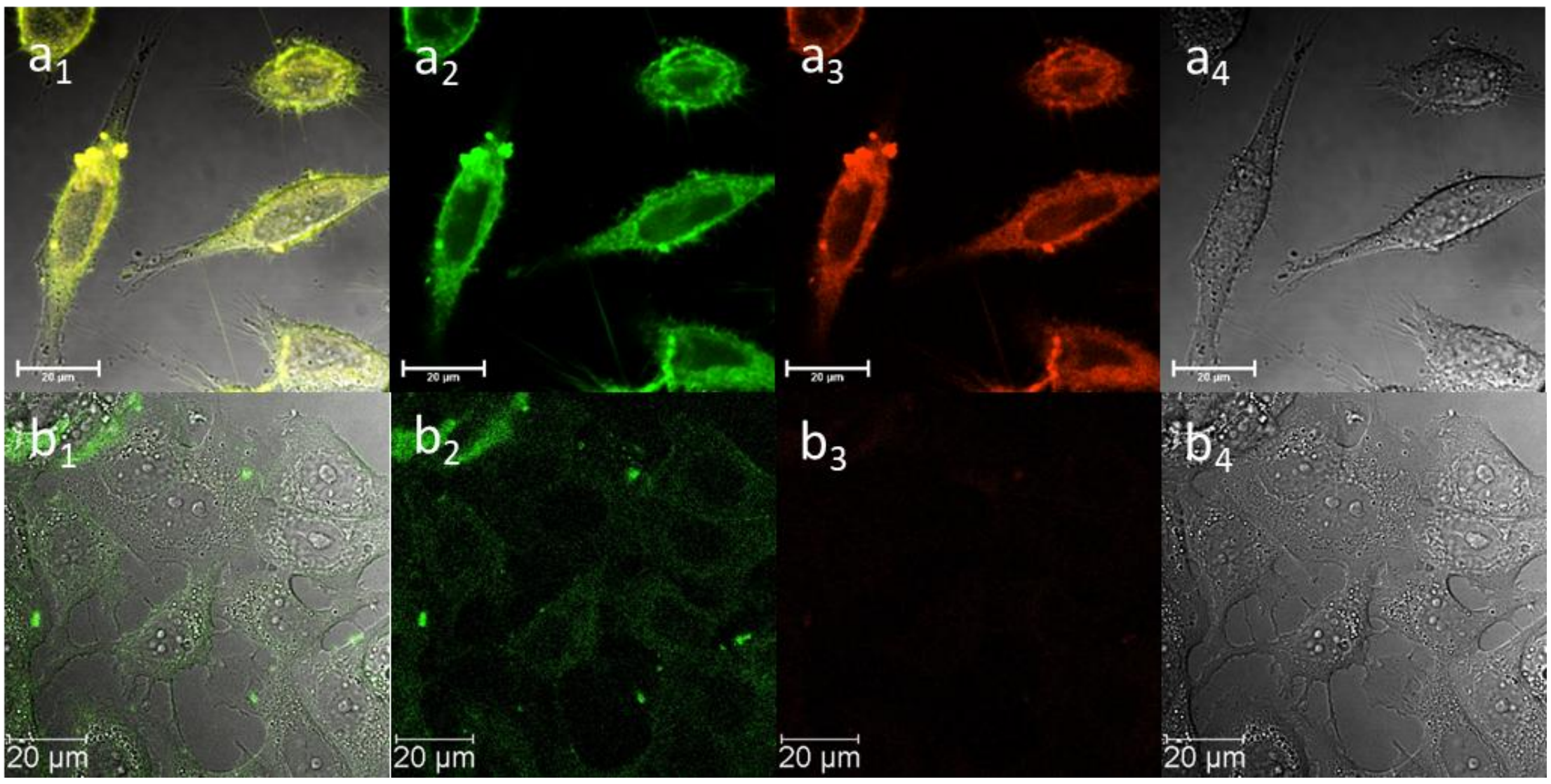

Figure S4.9. Confocal image of cells incubated with FITC-based compounds for 15 min under various conditions. a) 100 µM compound **5**, at 37 °C, HeLa cells; b) 100 µM **5**@glucan, at 37 °C, MCF-7 cells; a_1-b_1 is the overlay images of green channel, red channel and DIC; a_2-b_2 is the green channel, compounds were excited at 488 nm and emitted at 516-530 nm; a_3-b_3 is the red channel, excitation was at 488 nm and emission at 615-650 nm; a_4-b_4 is the bright field channel.

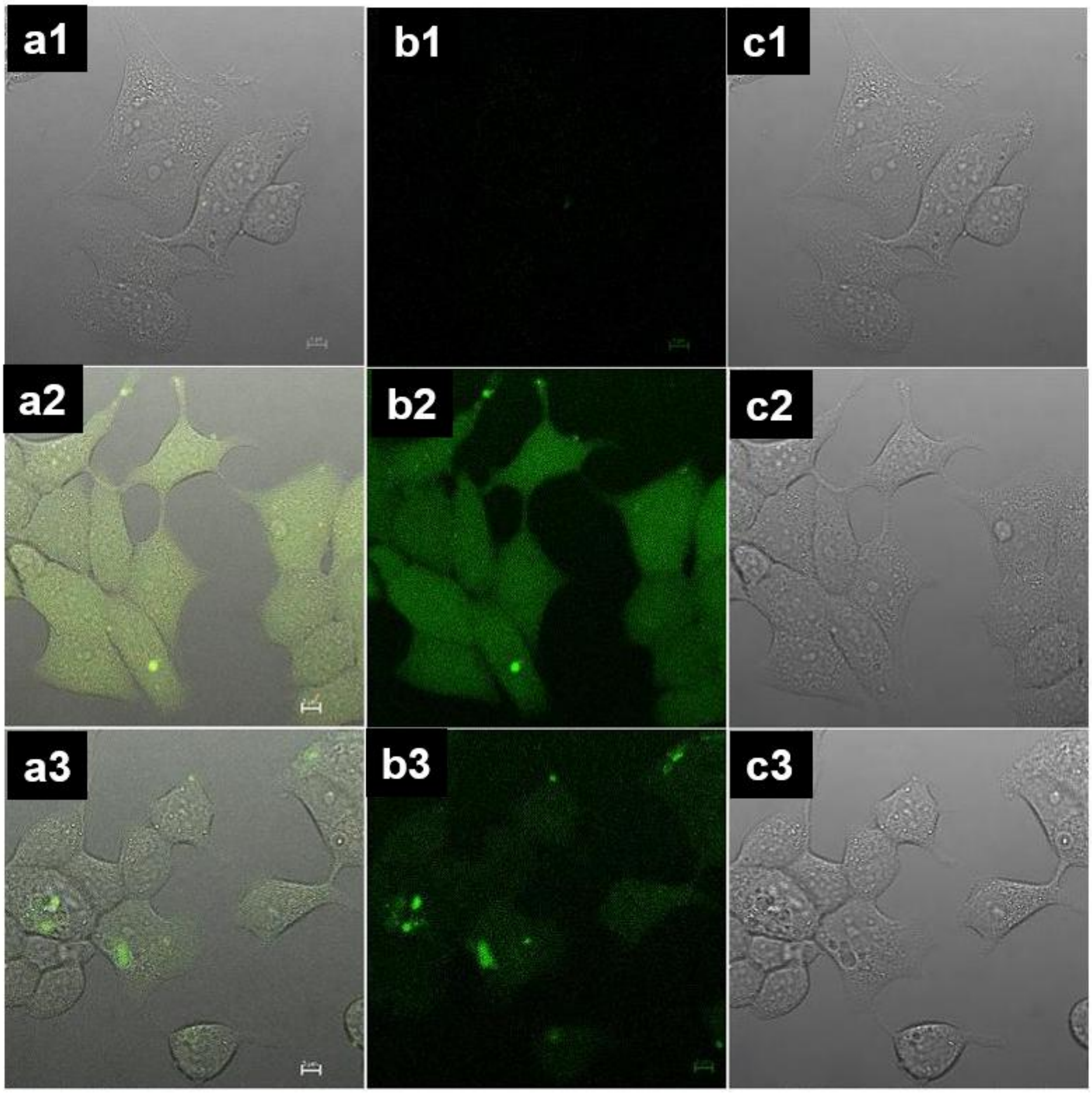

Figure S4.10. Confocal image of cells incubated with FITC-based compounds for 60 min under various conditions. a) MCF-7, control, DMEM with 1% pH 8.4 buffer; (b) 1 μM compound **5** in DMEM with 1% pH 8.4 buffer at 37 °C, MCF-7 cells; c) **5**@glucan formulation containing 1 μM compound **5** in DMEM with 1% pH 8.4 buffer at 37 °C in 1 mg/mL beta-D-glucan, MCF-7 cells; a_1-c_1 is the overlay images of green channel, red channel and DIC; a_2-c_2 is the green channel, excitation wavelength was 488 nm and emission was 516-530 nm; a_3-b_3 is the bright field.

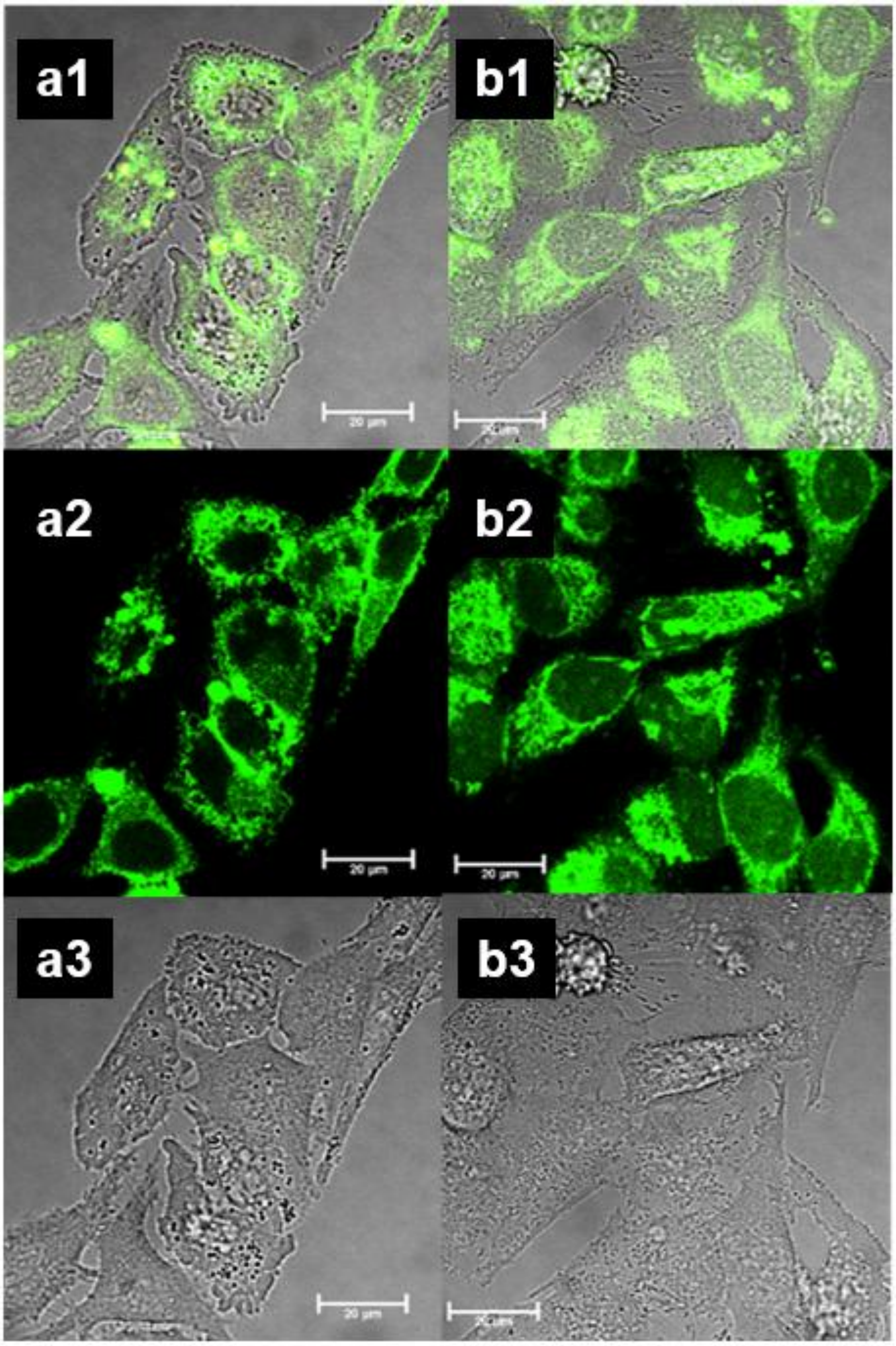

Figure S4.11. Confocal image of HeLa cells incubated with hybrid **5**@glucan containing 100 μM compound **5** in presence of excess beta-D-glucan under various conditions. a_1-a_3) **5**@glucan, 2% DMSO, at 37 ºC, 15 minutes; b_1-b_3) **5**@glucan, 2% DMSO, at 37 ºC, 60 minutes. a_1-b_1 are the overlay images of the green channel and DIC; a_2-b_2 are the green emission channel whereby excitation wavelength was 488 nm and emission at 516-530 nm; a_3-b_3 is the bright field channel.

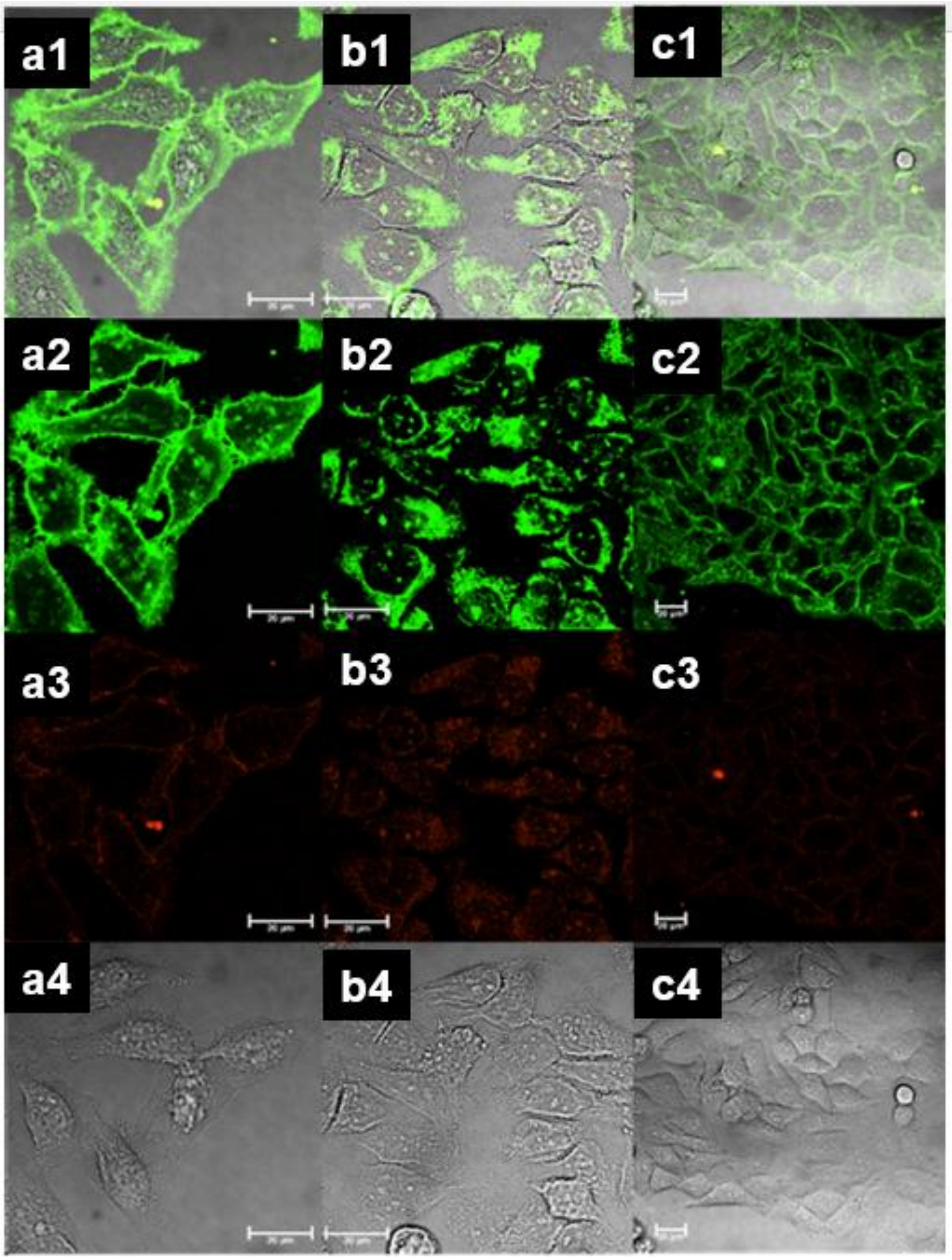

Figure S4.12. Confocal image of HeLa cells incubated with compound **5** (100 μM, 1% DMSO or 200 μM, 2% DMSO) alone, or in the presence of excess beta-D-glucan, under various conditions. a_1-a_4) compound **5**, 1% DMSO, at 4°C, 60 minutes b1-b4) **5**@gucan, 2% DMSO, at 4 °C, 15 minutes; c_1-c_4) **5**@gucan, 2% DMSO, at 4 °C, 60 minutes. a_1-c_1 are the overlay images of the green channel, red channel and DIC; a_2-c_2 are the green channel, compounds were excited at 488 nm and emission was at 516-530 nm; a_3-c_3 is the red channel; excitation wavelength was 488 nm and emission at 615-650 nm; a_4-c_4 represent the bright field channel.

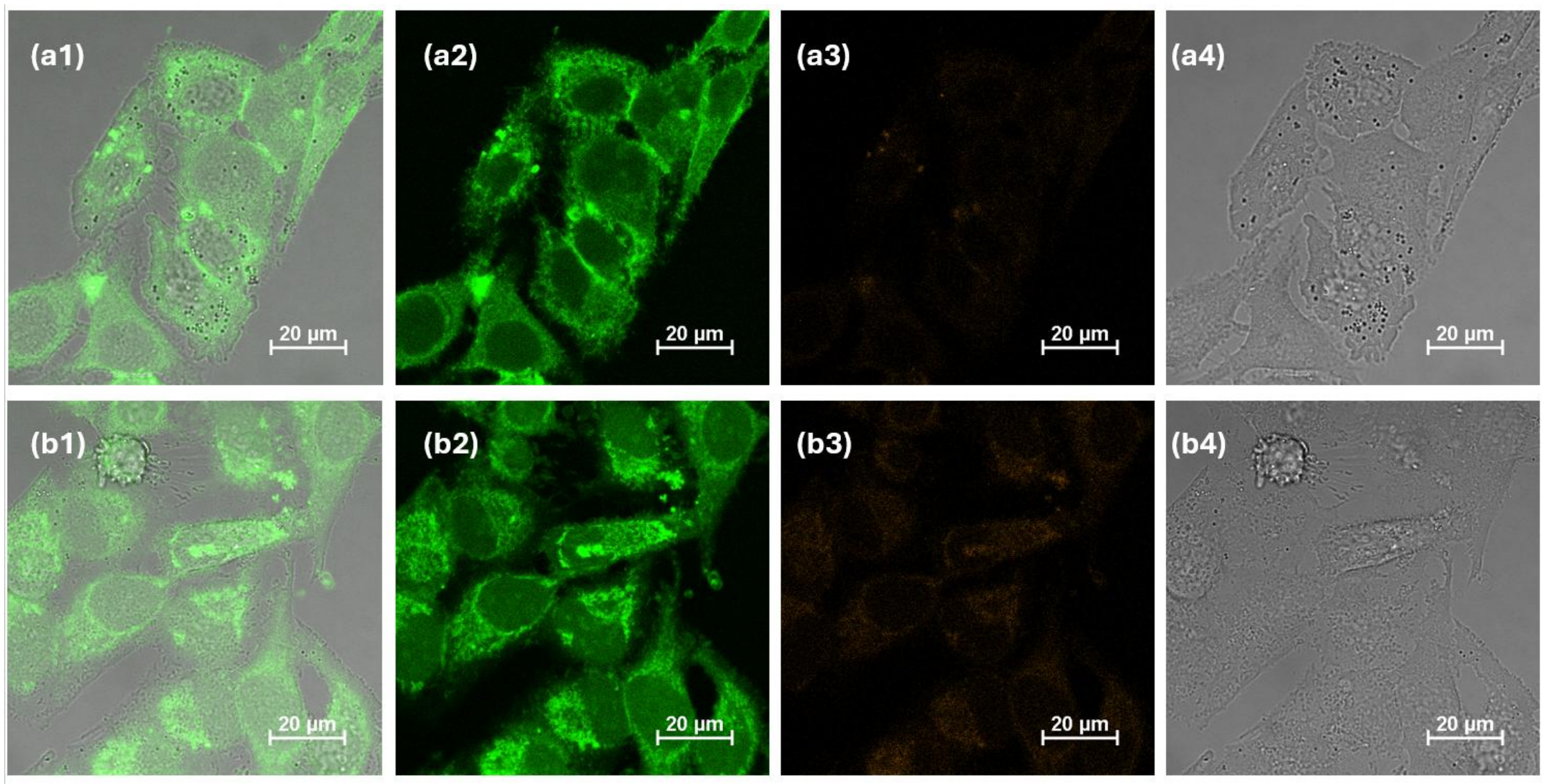

Figure S4.13. Comparison of the confocal images of HeLa cells incubated with compound **5** (100 µM, 1% DMSO) in the presence of excess beta-D-glucan, under various conditions. a_1-a_4) **5**@gucan, 2% DMSO, at 37 °C, 15 minutes and b_1-b_4) **5**@gucan, 2% DMSO, at 37 °C, 60 minutes, where a_1-b_1 are the overlay images of the green and red emission channels and DICs; a_2-b_2 are the green channels, compounds were excited at 488 nm and emission was at 516-530 nm; a_3-b_3 are the red channels; excitation wavelength was 488 nm and emission at 615-650 nm; a_4-b_4 represent the bright field channels.

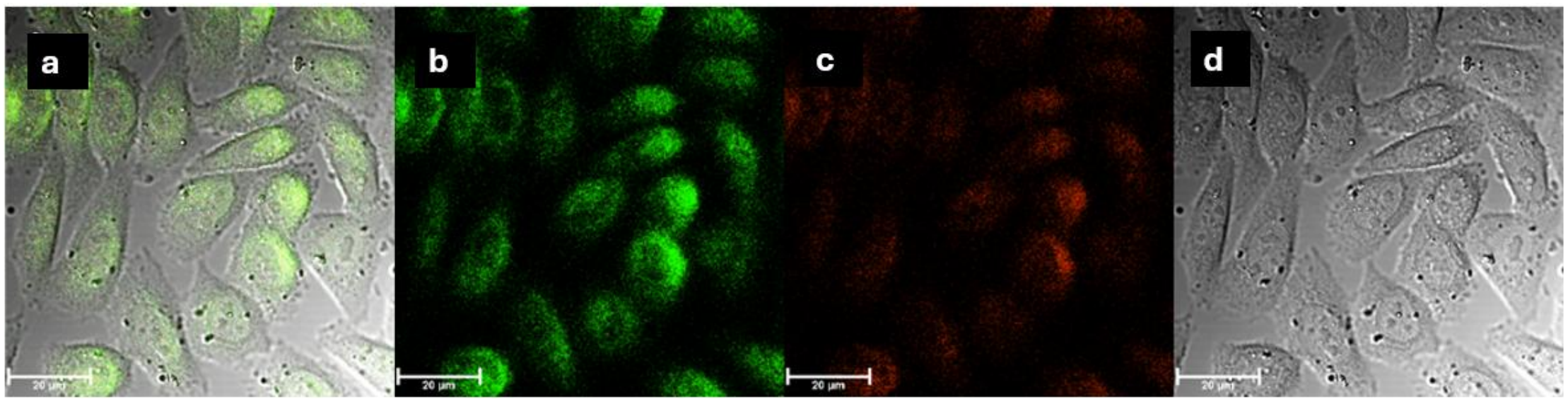

Figure S4.14. Confocal image of CHO cells incubated with compound **6** for 15 min at 37 ºC in CHO cells; a is the overlay images of green channel, red channel and DIC; b is the green channel, excitation at 488 nm and emission at 516-530 nm; c is the red channel, excitation 488 nm and emitted at 615-650 nm; d is the bright field channel.

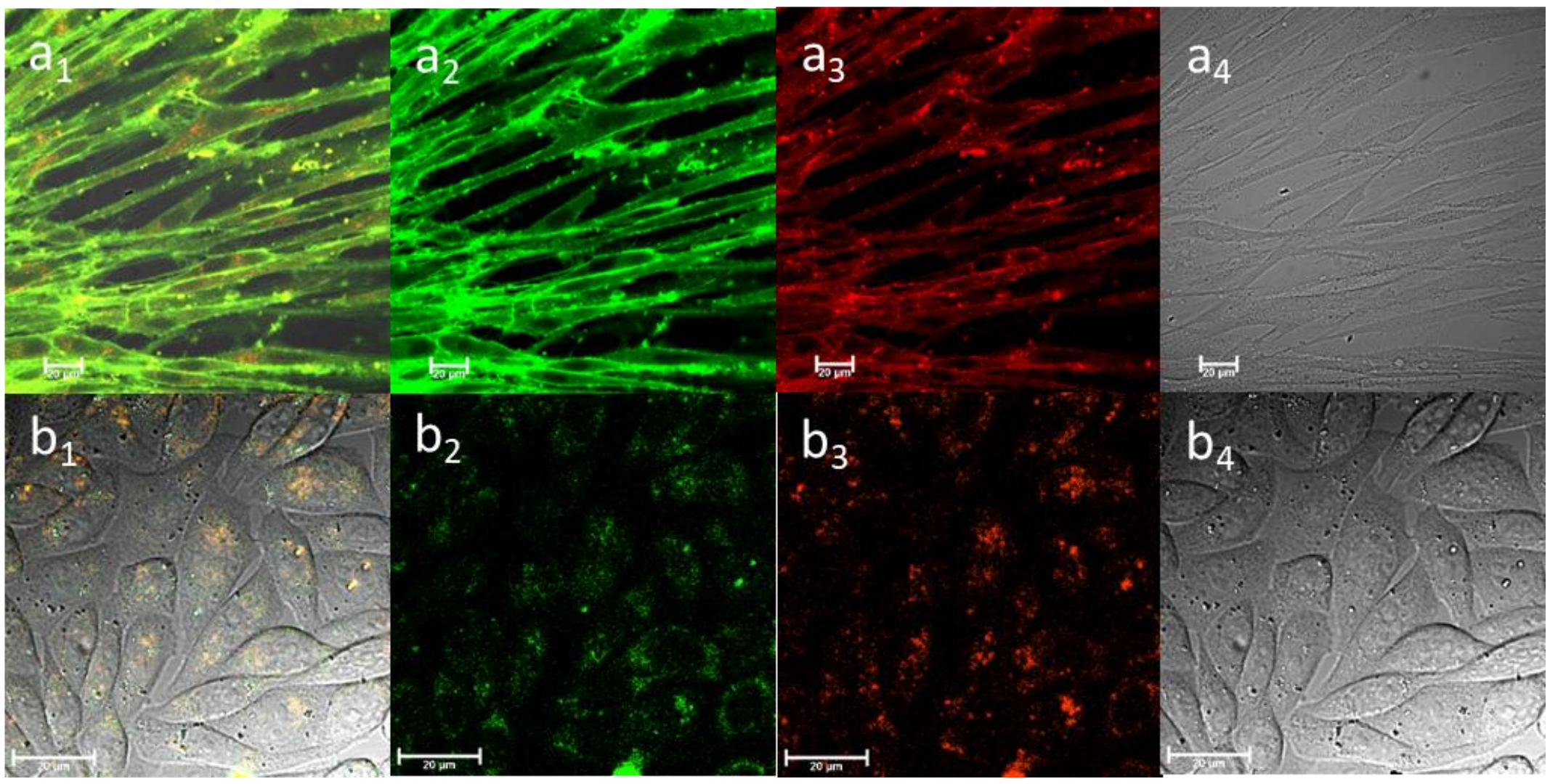

Figure S4.15. Confocal image of living healthy cells incubated for 15 min with compound **6** and corresponding nanohybrid incorporating compound **6** under various conditions. a_1-a_4) 100 μM compound **6**, at 37 ºC, FEK4 cells; b_1-b_4) 10 μg/mL **6**@SiO_2@Fe_3O_4, at 37 ºC, CHO cells; a_1-b_1 is the overlay images of green channel, red channel and DIC; a_2-b_2 is the green channel, excitation at 488 nm and emission at 516-530 nm; a_3-b_3 is the red channel, excitation 488 nm and emitted at 615-650 nm; b_4-c_4 is the bright field channel.

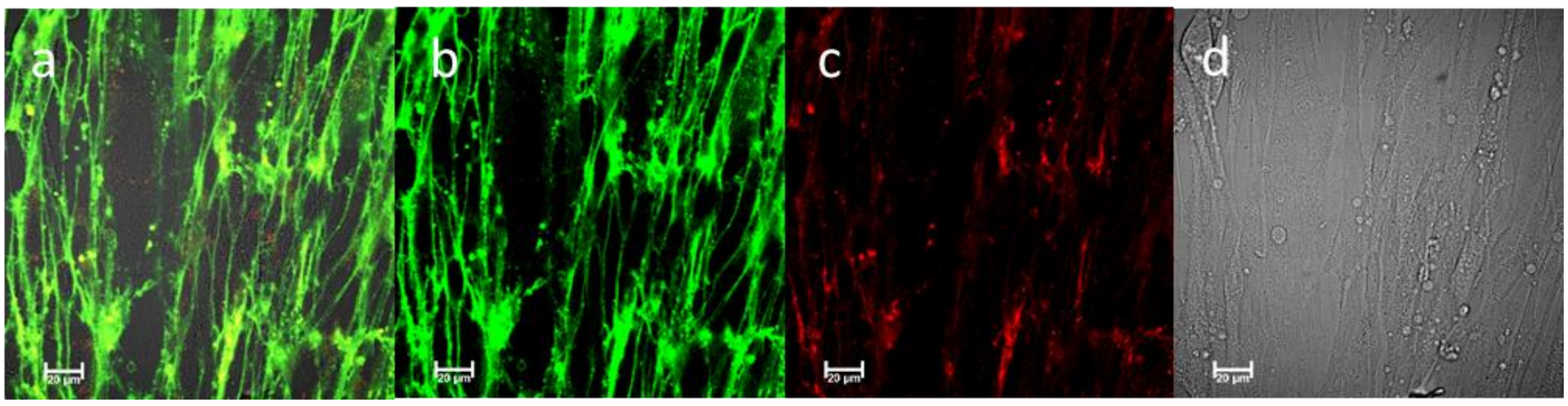

Figure S4.16. Confocal image of cells incubated with compound **6** for 15 min at 37 ºC in FEK4 cells; a is the overlay images of green channel, red channel and DIC; b is the green channel, excitation at 488 nm and emission at 516-530 nm; c is the red channel, excitation at 488 nm and emission at 615-650 nm; d is the bright field channel.

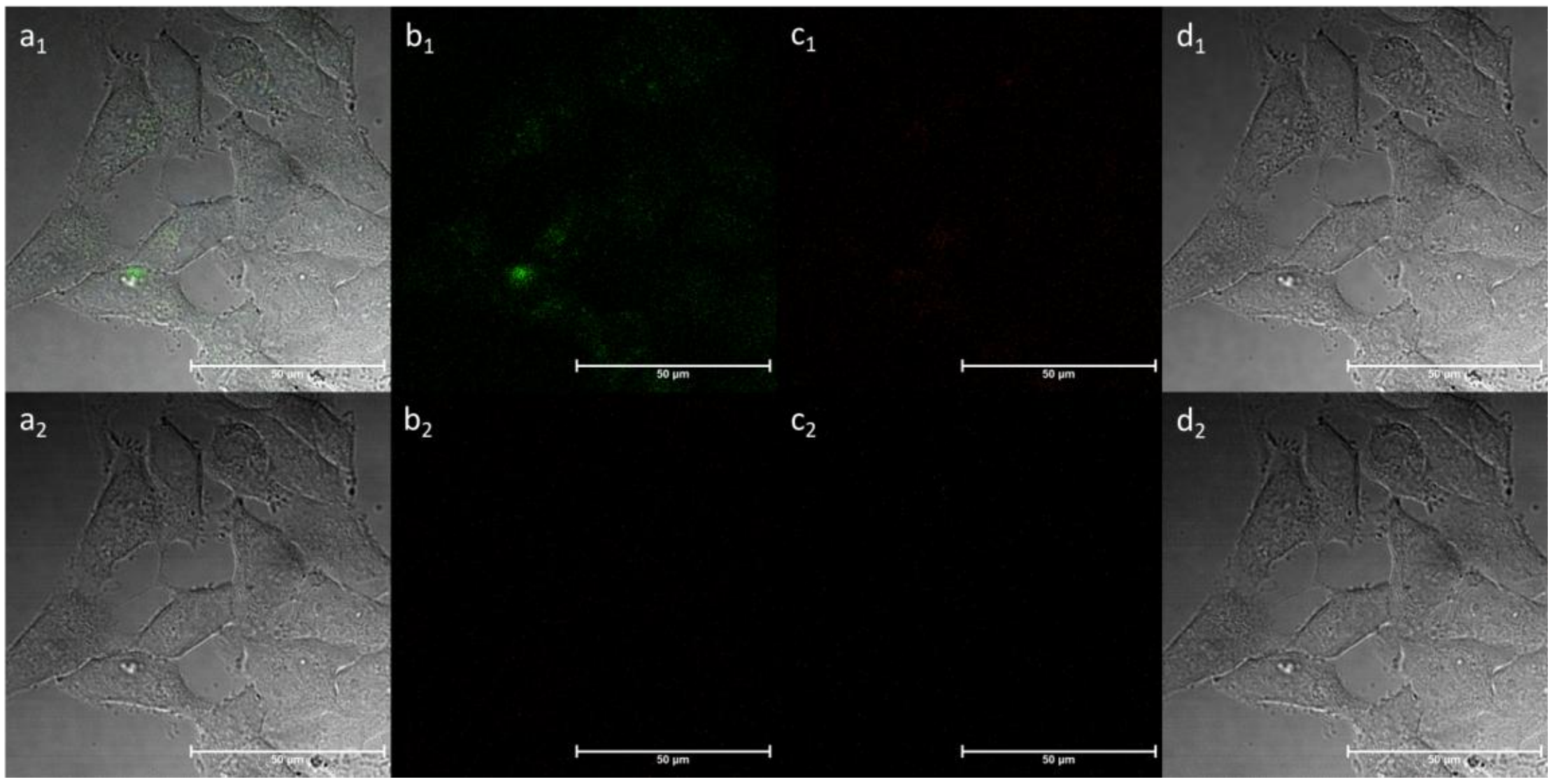

Figure S4.17. Confocal image of HeLa cells incubated with the FITC-based compound **4** at 100 μM for 15 min at 37 °C. a_1-a_2 is the overlay images of green channel, red channel and DIC; b_1-b_2 is the green channel, compounds were excited at 488 nm and 561 nm, emitted at 516-530 nm; c_1-c_2 is the red channel, compounds were excited at 488 nm and 561nm, emitted at 615-650 nm; d_1-d_2 is the bright field channel.

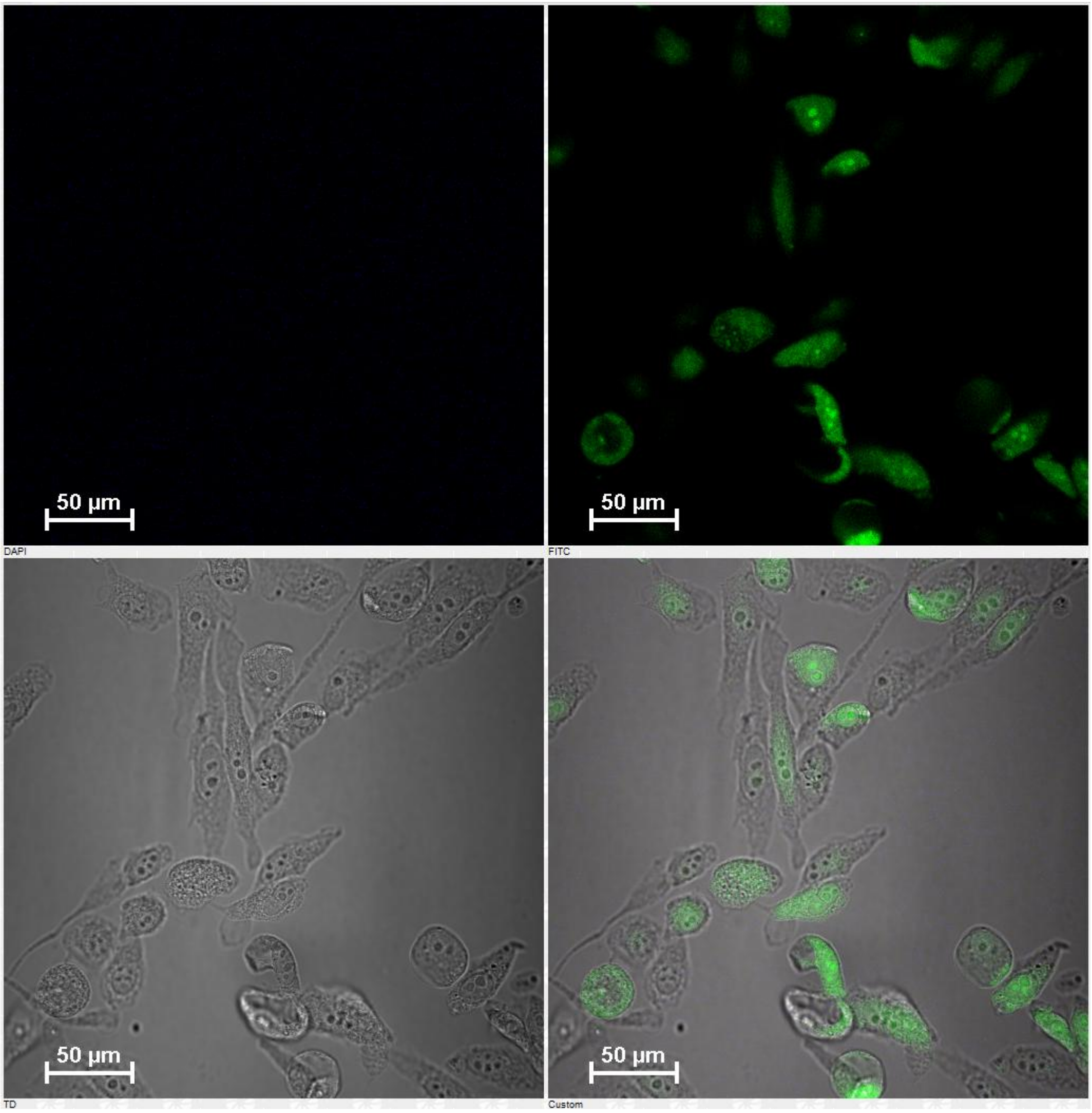

Figure S4.18. Confocal fluorescence imaging of PC-3 cells incubated with Compound **4**, 100 µM 1% DMSO for 15 min at 37 °C. Clockwise from the top: blue channel, excitation at 405 nm and emission at 430 nm; green channel, excitation at 488 nm and emission at 516-530 nm; the bright field channel and overlay of micrographs.

Confocal fluorescence imaging micrographs corresponding to timelapse observations over 1 h:

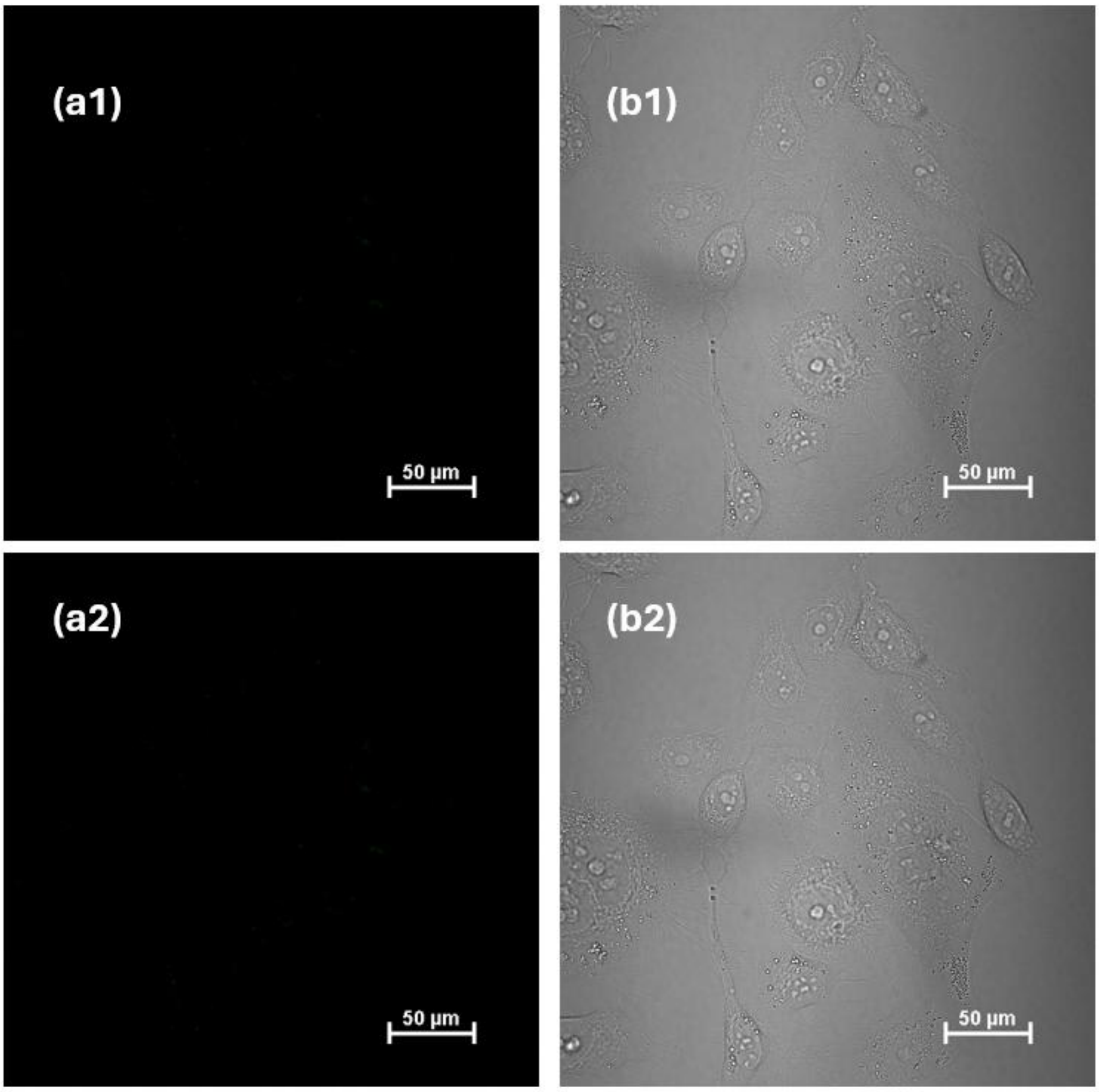

Figure S4.19. Confocal fluorescence imaging of PC-3 cells incubated with 1% DMSO for 20 min at 37 °C. (a1-b1): Timelapse t = 0 min imaging, green channel, excitation at 488 nm (a1) and the bright field channel (b1); (a1-b1): Timelapse t = 60 min under imaging conditions, green channel, excitation at 488 nm (a1) and the bright field channel (b1). Corresponding timelapse images as .mov are provided in Supplementary data.

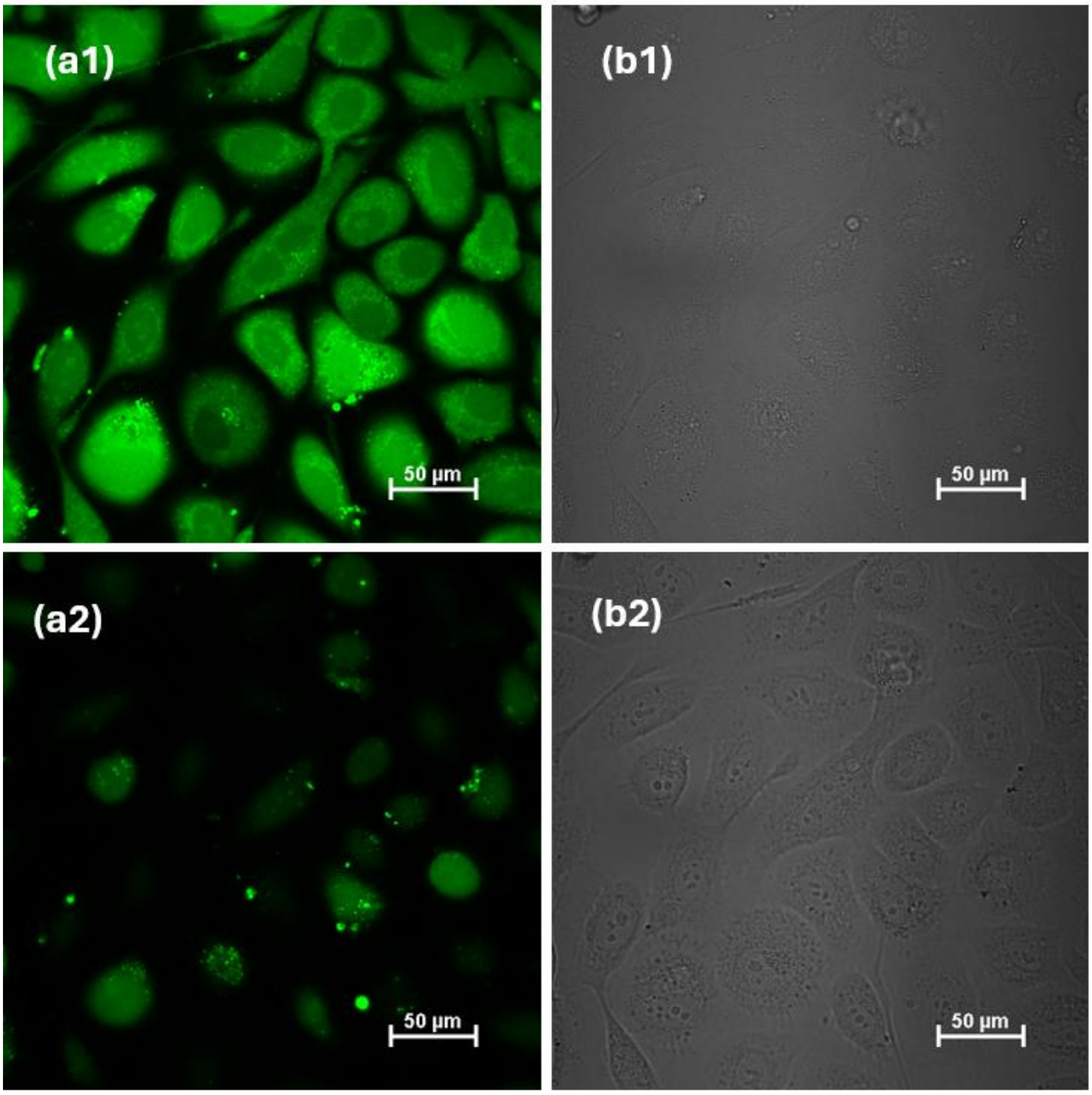

Figure S4.20. Confocal fluorescence imaging of PC-3 cells incubated with compound 1, 100 μM conc. and 1% DMSO for 20 min at 37 °C and without PBS washing, showing minimal cellular uptake. (a1-b1): Timelapse t = 0 min imaging, green channel, excitation at 488 nm (a1) and the bright field channel (b1); (a1-b1): Timelapse t = 60 min under imaging conditions, green channel, excitation at 488 nm (a1) and the bright field channel (b1). Images showed significant photobleaching in this interval but no cellular membrane damage. Corresponding timelapse images as .mov are provided in Supplementary data.

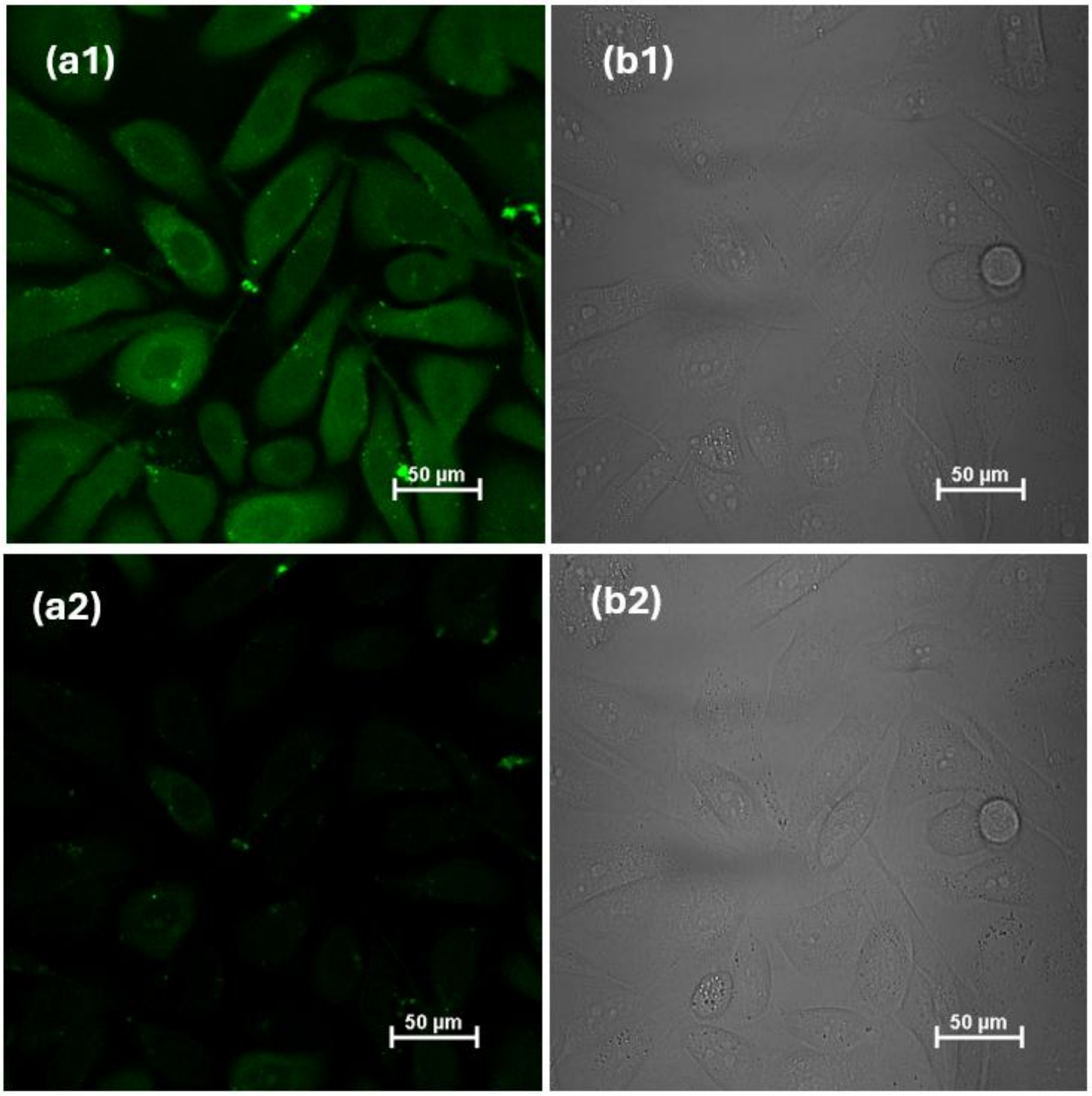

Figure S4.21. Confocal fluorescence imaging of PC-3 cells incubated with compound 1, 100 µM conc. and 1% DMSO for 20 min at 37 °C and without PBS washing, showing minimal cellular uptake. (a1-b1): Timelapse t = 0 min imaging, green channel, excitation at 488 nm (a1) and the bright field channel (b1); (a1-b1): Timelapse t = 60 min under imaging conditions, green channel, excitation at 488 nm (a1) and the bright field channel (b1). Images showed significant photobleaching in this interval but no cellular membrane damage. Corresponding timelapse images as .mov are provided in Supplementary data.

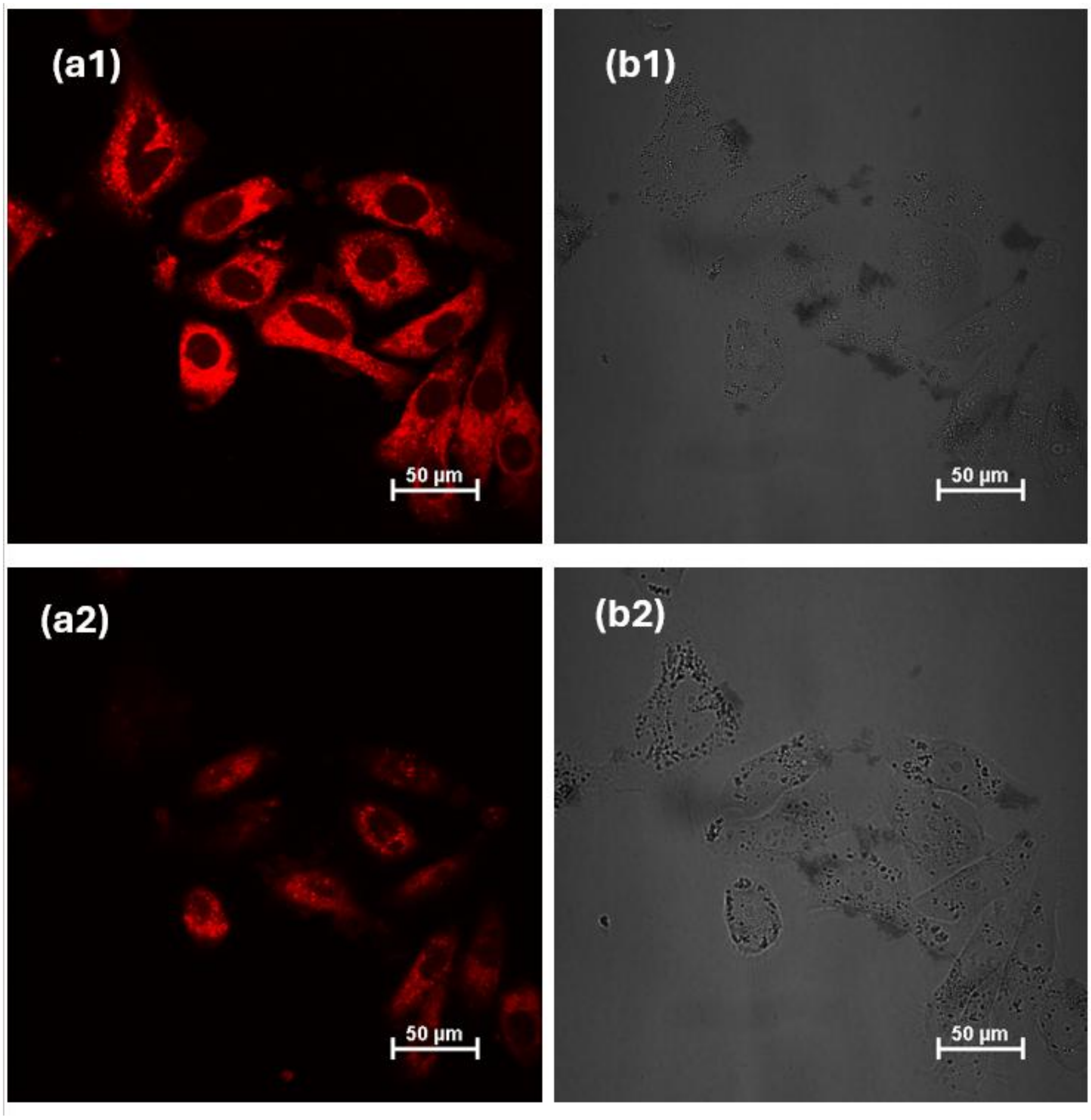

Figure S4.22. Confocal fluorescence imaging of PC-3 cells incubated with compound 3, 100 μM conc. and 1% DMSO for 20 min at 37 °C and without PBS washing, showing minimal cellular uptake. (a1-b1): Timelapse t = 0 min imaging, red emission channel, excitation at 563 nm (a1) and the bright field channel (b1); (a1-b1): Timelapse t = 60 min under imaging conditions, red emission channel, excitation at 563 nm (a1) and the bright field channel (b1). Images showed significant photobleaching in this interval but no cellular membrane damage. Corresponding timelapse images as .mov are provided in Supplementary data.

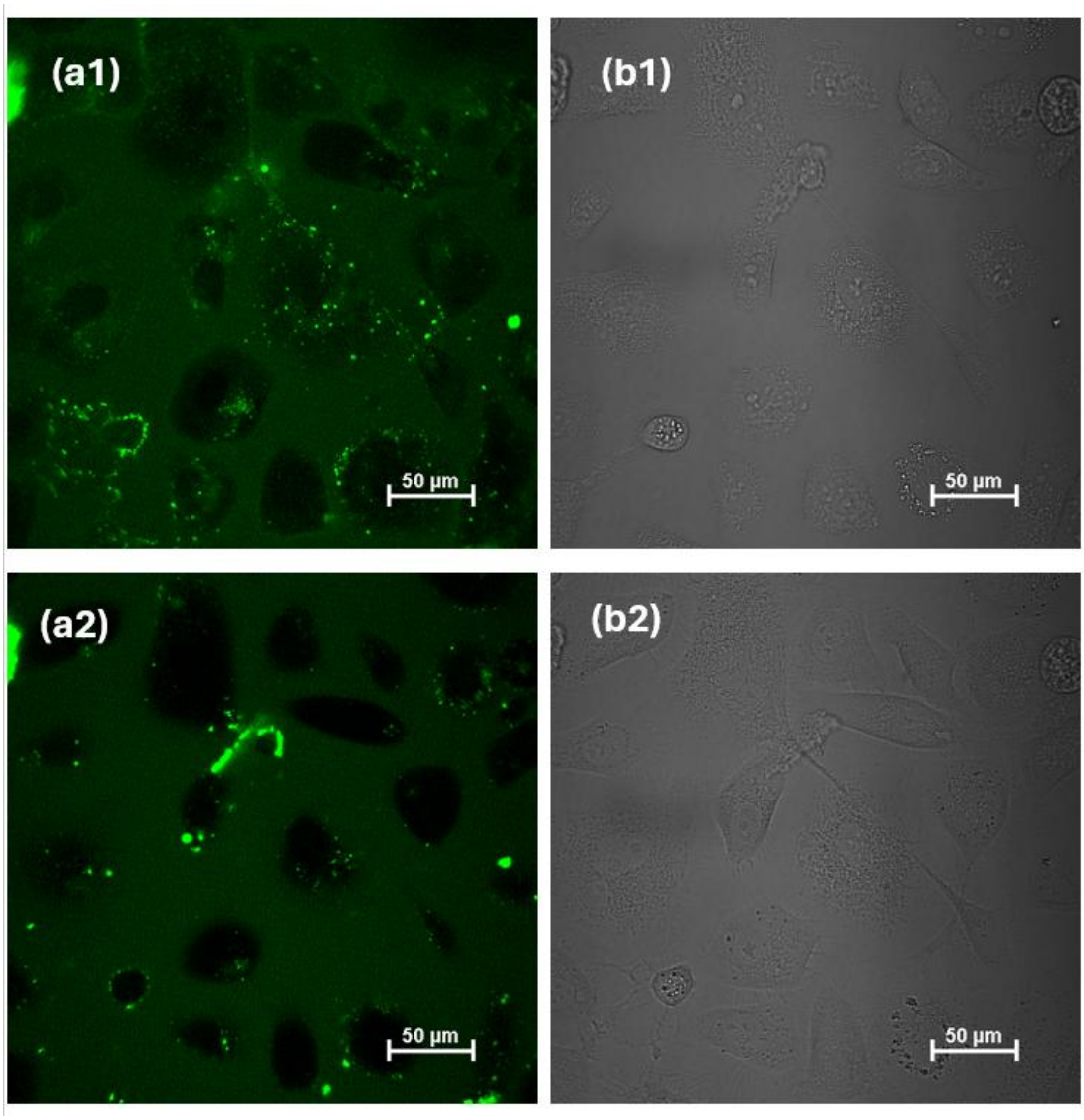

Figure S4.23. Confocal fluorescence imaging of PC-3 cells incubated with compound 4, 100 µM conc. and 1% DMSO for 20 min at 37 °C and without PBS washing, showing minimal cellular uptake. (a1-b1): Timelapse t = 0 min imaging, green channel, excitation at 488 nm (a1) and the bright field channel (b1); (a1-b1): Timelapse t = 60 min under imaging conditions, green channel, excitation at 488 nm (a1) and the bright field channel (b1). Corresponding timelapse images as .mov are provided in Supplementary data.

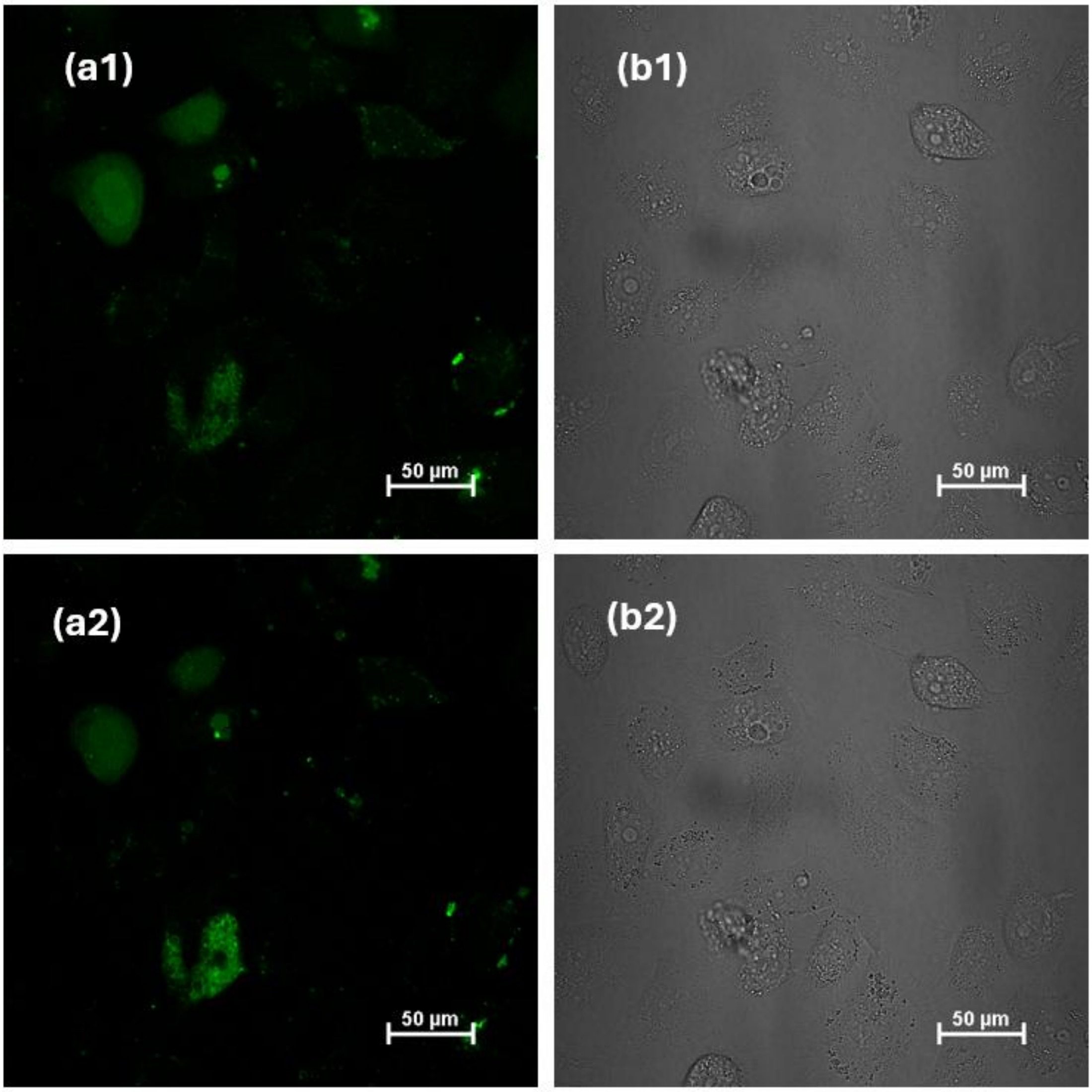

Figure S4.24. Confocal fluorescence imaging of PC-3 cells incubated with compound 5, 100 µM conc. and 1% DMSO for 20 min at 37 °C. (a1-b1): Timelapse t = 0 min imaging, green channel, excitation at 488 nm (a1) and the bright field channel (b1); (a1-b1): Timelapse t = 60 min under imaging conditions, green channel, excitation at 488 nm (a1) and the bright field channel (b1). Corresponding timelapse images as .mov are provided in Supplementary data.

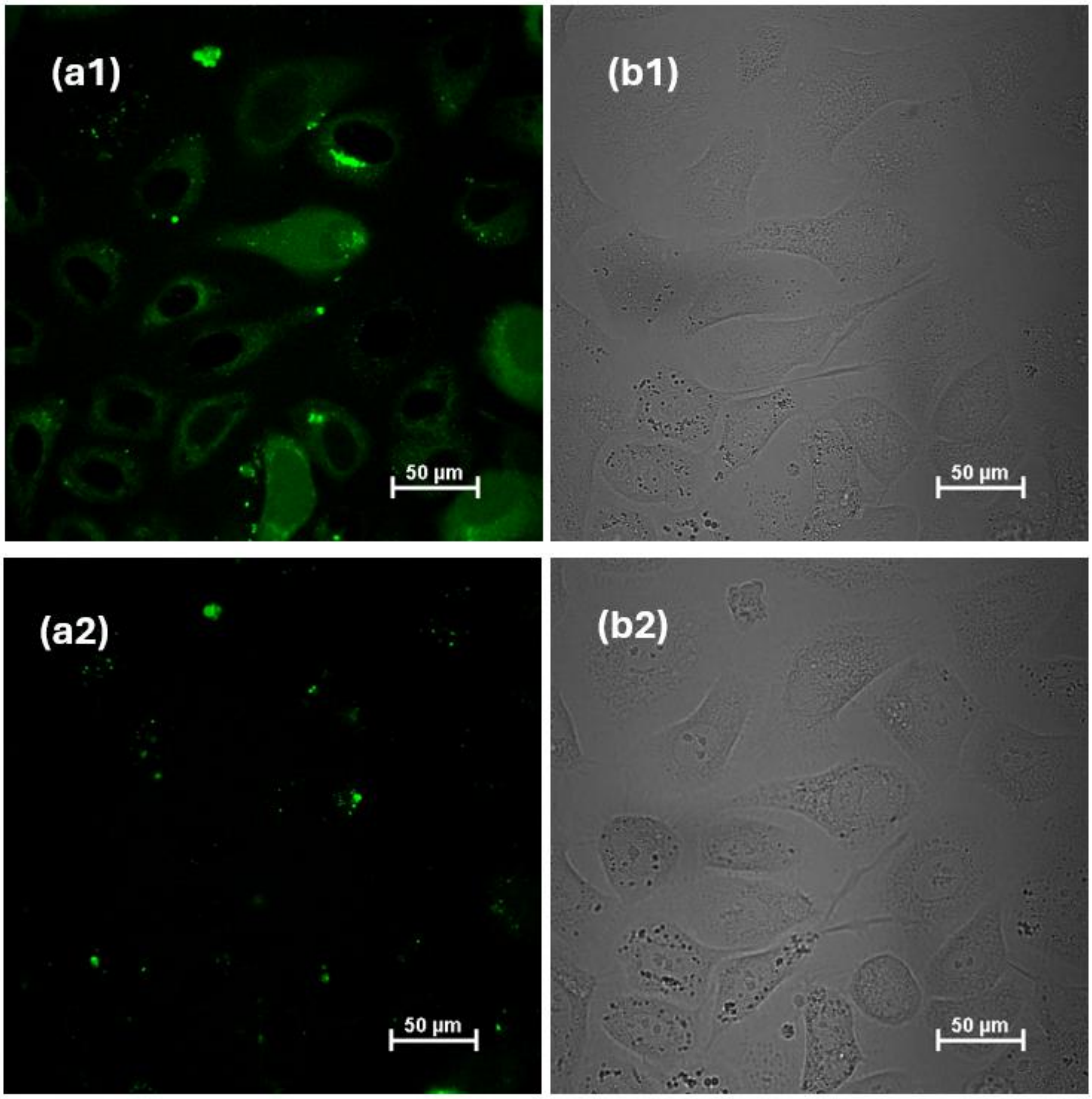

Figure S4.25. Confocal fluorescence imaging of PC-3 cells incubated with compound 6, 100 µM conc. and 1% DMSO for 20 min at 37 °C. (a1-b1): Timelapse t = 0 min imaging, green channel, excitation at 488 nm (a1) and the bright field channel (b1); (a1-b1): Timelapse t = 60 min under imaging conditions, green channel, excitation at 488 nm (a1) and the bright field channel (b1). Corresponding timelapse images as .mov are provided in Supplementary data.

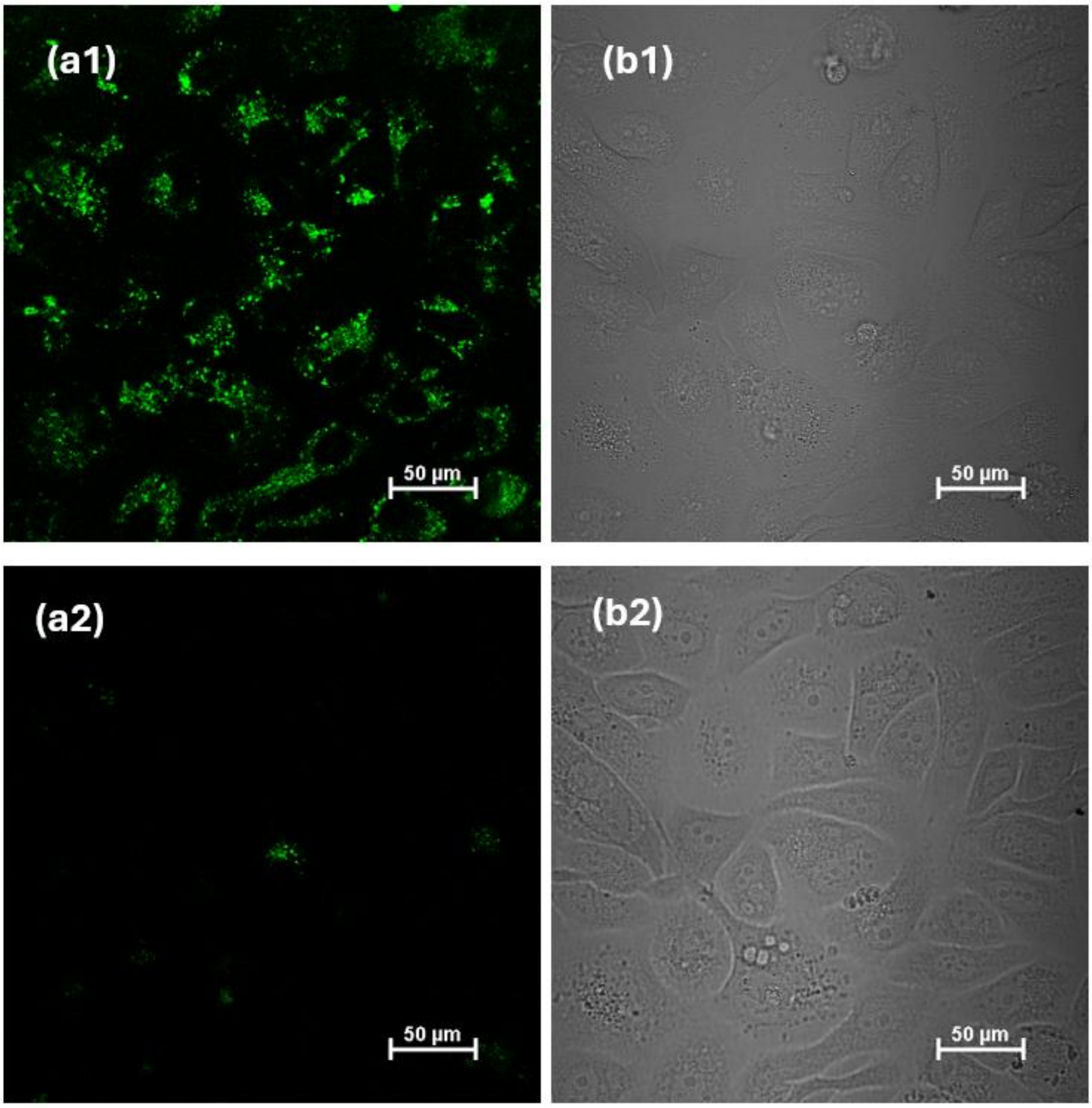

Figure S4.26. Confocal fluorescence imaging of PC-3 cells incubated with compound 6, 100 µM conc. and 1% DMSO anchored onto 1 mg/ml Fe_3O_4@SiO_2 nanoparticle in H2O, for 20 min at 37 °C. (a1-b1): Timelapse t = 0 min imaging, green channel, excitation at 488 nm (a1) and the bright field channel (b1); (a1-b1): Timelapse t = 60 min under imaging conditions, green channel, excitation at 488 nm (a1) and the bright field channel (b1). Images showed significant photobleaching in this interval but no cellular membrane damage. Corresponding timelapse images as .mov are provided in Supplementary data.

5. Colocalisation Assays with Commercial Dyes

A range of commercial of organelles tracking dyes were used to localise distribution of compounds 1-6 *via* confocal microscopy; all dyes in the following studies were purchased from Invitrogen/Thermo Fischer and used as supplied. Cells were cultured in Eagle's Minimum Essential Medium (EMEM) or Roswell Park Memorial Institute (RPMI)-1640 medium using standard protocols described above. Prior to addition of any commercial co-localisation dye, cells were washed five times with HBSS (1 mL).

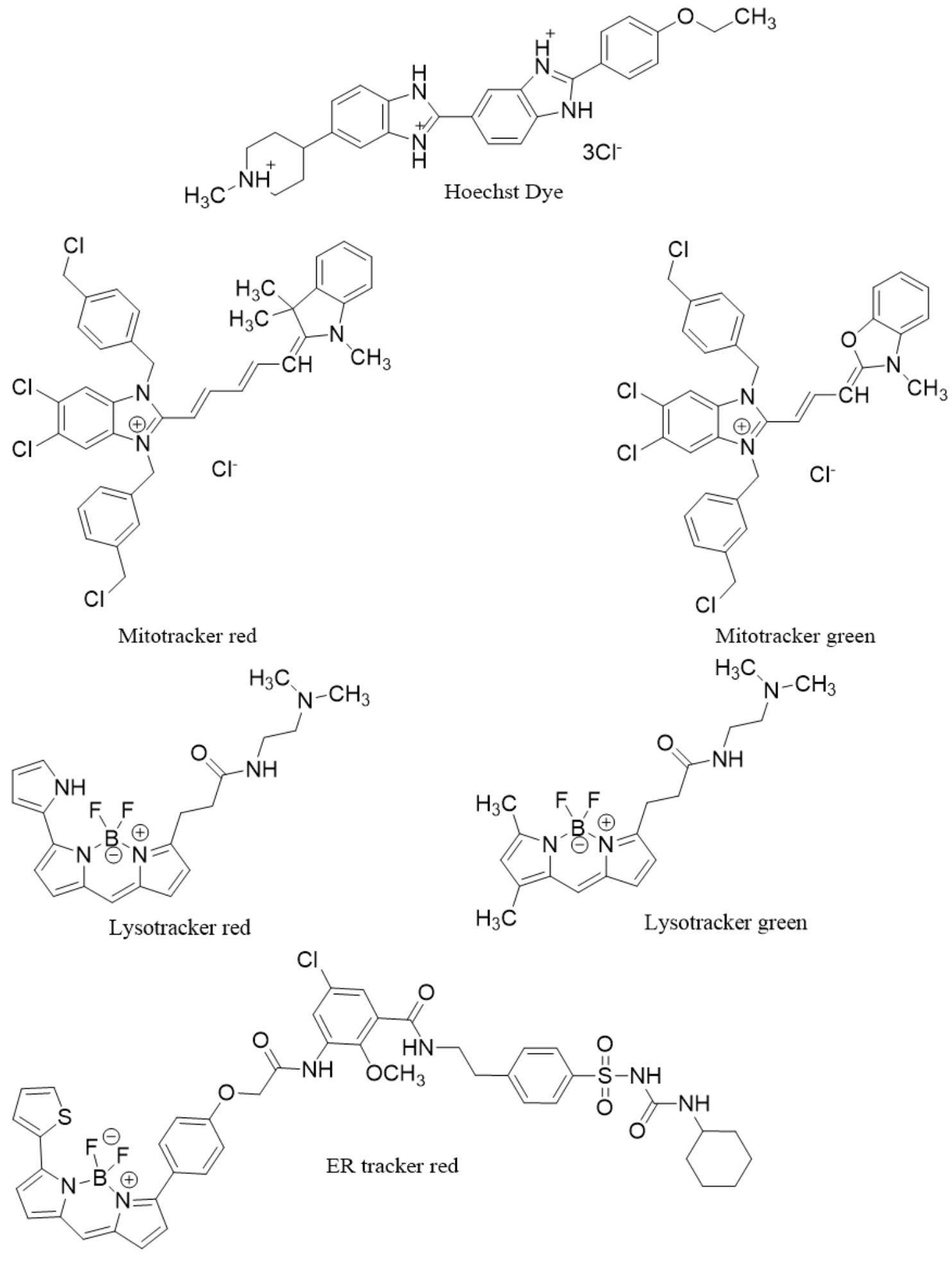

Figure 5.1. structure of dyes used in the colocalisation studies

Hoechst solution (Nuclear DNA localisation dye):

The Hoechst solution (10 mg/mL) was prepared by dissolving Hoechst 33342 (Invitrogen) (10mg) in sterile Milli-Q water (1 mL), The 10 mg/mL solution (10 µL) was dissolved into sterile water (990 µL) to make a 100 µg/mL stock solution. Cells were then washed five times with HBSS, and then refilled with SFM (990 µL). The 100 µg/mL stock solution (10 µL) was then added to the SFM (990 µL). Cells were then incubated for 20 to 30 minutes. Subsequently, cells were washed three times with HBSS (1 mL) and refilled with SFM (1 mL) to take control images. Then, cells were washed and the compounds of interest were loaded. After 15 min incubation with the

compound, cells were washed three times with HBSS (1 mL) and refilled with fresh SFM (1 mL). Confocal images were recorded immediately afterwards.

MitoTracker (mitochondrial tracing dye):

Two MitoTracker dyes were used (Invitrogen) in the colocalisation, one was MitoRed and the other one was MitoGreen, both dyes could label mitochondria with the former excited at 543 nm and the later excited at 488 nm. In each case, to prepare stock solutions, to the as-bought MitoTrackers (50 µg), DMSO (69.06 µL) was added to MitoTracker red and DMSO (74.42 µL) to Mitotracker green each resulting in a 1 mM stock solution.
Subsequently, the 1 mM stock solution (20 µL) was diluted into DMSO (980 µL) to make 20 µM stock solution. Prior to the test, cells were washed five times with HBSS (1 mL), then the cell plate was refilled with SFM (990 µL). To this, the 20 µM stock solution (10 µL) was added. Cells were incubated for 15 to 45 minutes and subsequently washed three times with HBSS (1 mL). The dishes were then refilled to 1 mL fresh SFM to take control images of the cells with dye-stained mitochondria. Then, cells were washed and the imaging probe of interest was loaded. After 15 min incubation with the compound, cells were washed three times with HBSS (1 mL) and refilled with fresh SFM (1 mL). Confocal images were recorded immediately afterwards.

Lysotracker (lysosomes tracing dye):

Two lysotracker dyes were used (Invitrogen) in the lysosome tracking: Lysotracker red and Lysotracker green. The excitation wavelength for each Lysotracker was 577 nm and 488 nm respectively. The stock solution available from the supplier (Invitrogen) is 1 mM. For co-localisation experiments, this 1 mM stock (20 µL) was dissolved into DMSO (980 µL) to obtain 20 µM stock solutions. Cells were washed five times with HBSS (1 mL), and then plates were refilled with SFM (990 µL). The 20 µM stock solution (10 µL) was prepared as above and were added to the SFM (990 µL) to make a 200 nM final concentration. Cells were incubated for 30 to 120 minutes then washed three times with HBSS (1 mL). Plates were refilled with SFM (1 mL) to take control images of lysosomes stained cells. Then, cells were washed with HBSS and refilled with fresh SFM and the desired compound for co-localisation images were loaded. After 15 min incubation with the compound, cells were washed three times with HBSS and refilled with fresh SFM (1 mL). Confocal images were recorded immediately afterwards.

ER tracker (dye that selectively stains the endoplasmic reticulum):

The commercially available ER tracker from the supplier (Invitrogen) was 1 mM. From this 10 µL was diluted with DMSO (90 µL) to obtain 100 µM stock solutions. Then, cells were washed five times with HBSS and plates were refilled with SFM (990 µL). Subsequently, the 100 µM stock solution (10 µL) was added to make 1 µM solutions on the cell plate. Cells were incubated for 15 to 30 minutes and subsequently cells were washed three times with HBSS. The plates were refilled with 1 mL SFM to take control images of ER (endoplasmic reticulum) -stained cells. Finally, cells were washed and the compound of interest for co-localisation studies was loaded. After 15 min

incubation with the compound, cells were washed three times with HBSS and refilled with fresh SFM (1 mL). Confocal images were recorded immediately afterwards.

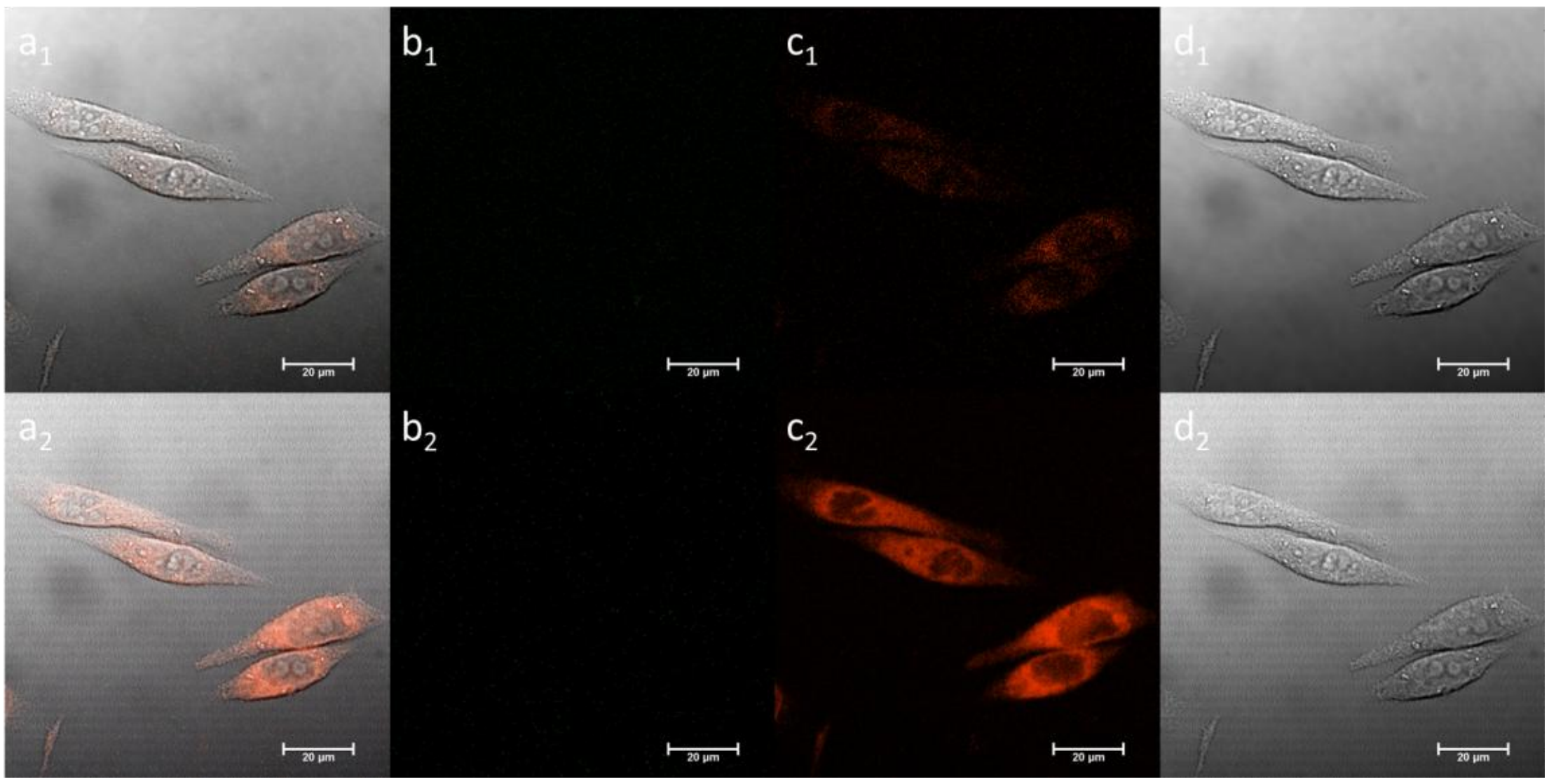

Figure S5.2. Confocal image of CHO cells incubated with 1 μM endoplasmic reticulum (ER)-tracker red for 30 min at 37 °C; a_1-a_2 is the overlay images of green channel, red channel and DIC; b_1-b_2 is the green channel, excitation wavelength 488 nm (top row) 561 nm (bottom row), emission was at 516-530 nm; c_1-c_2 is the red channel, excitation at 488 nm (top row) and 561 nm (bottom row) and emission was at 615-650 nm; d_1-d_2 is the bright field channel.

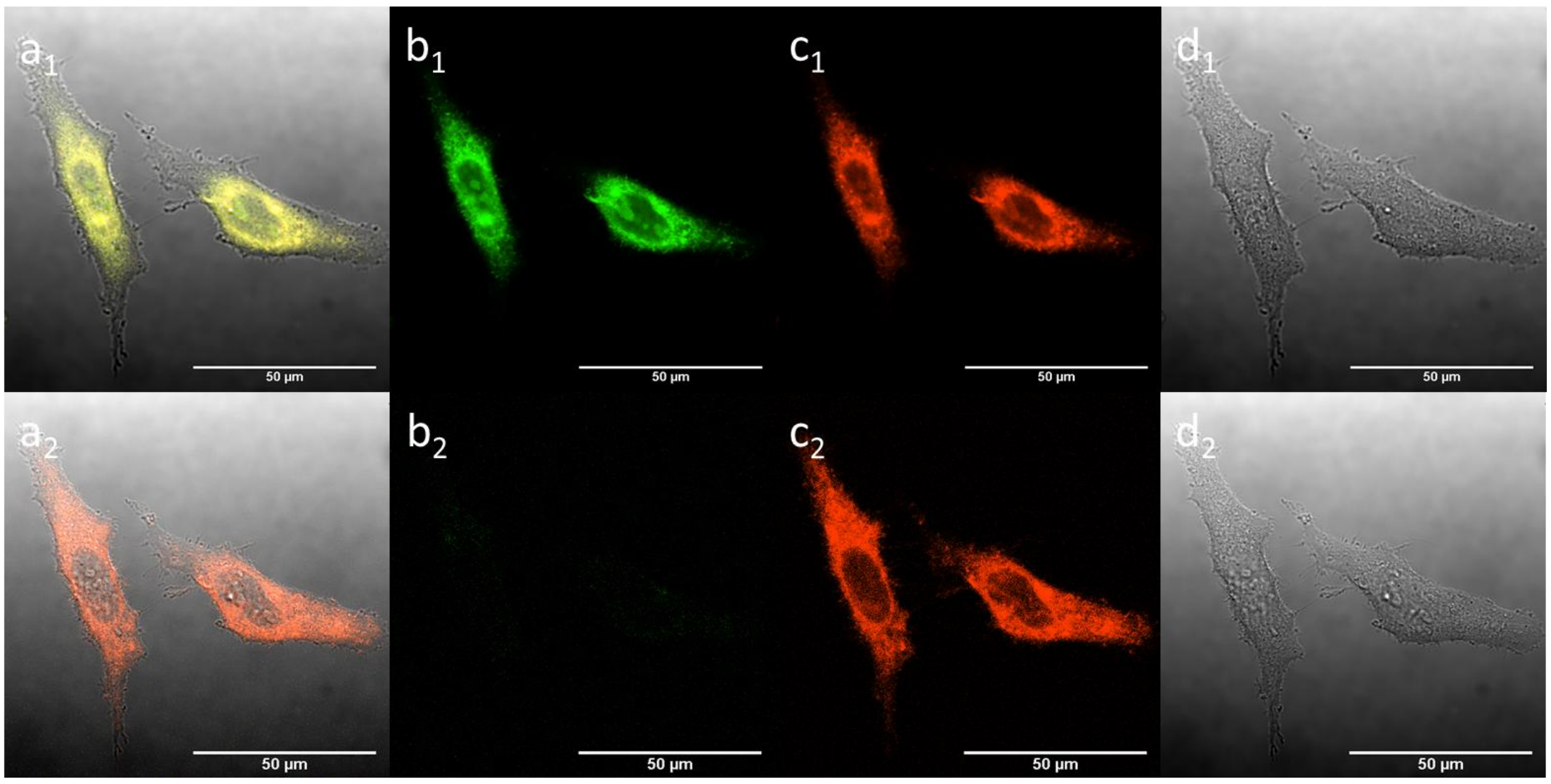

Figure S5.3. Confocal image of HeLa cells incubated with 100 nM lysosome-tracker green for 30 min at 37 °C; a_1-a_2 is the overlay images of green channel, red channel and DIC; b_1-b_2 is the green channel, excitation wavelength 488 nm (top row) 561 nm (bottom row), emission was at 516-530 nm; c_1-c_2 is the red channel, excitation at 488 nm (top row) and 561 nm (bottom row) and emission was at 615-650 nm; d_1-d_2 is the bright field channel.

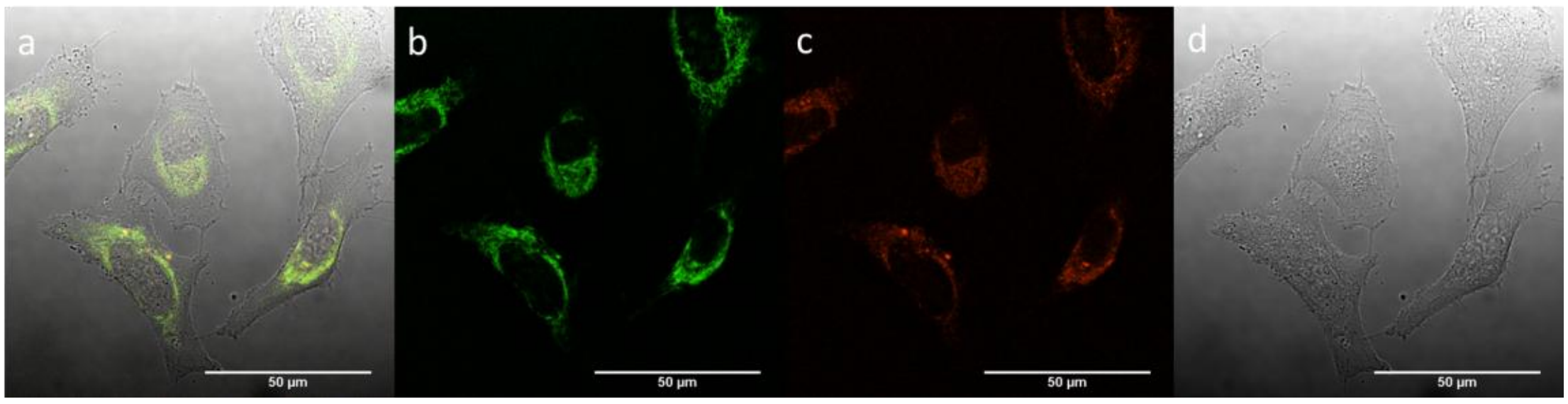

Figure S5.4. Confocal image of HeLa cells incubated with 200 nM Mitochondria-tracker Green for 30 min at 37 °C; a is the overlay images of green channel, red channel and DIC; b is the green channel, excitation at 488 nm and emission at 516-530 nm; c is the red channel, excitation at 488 nm and emission at 615-650 nm; d is the bright field channel.

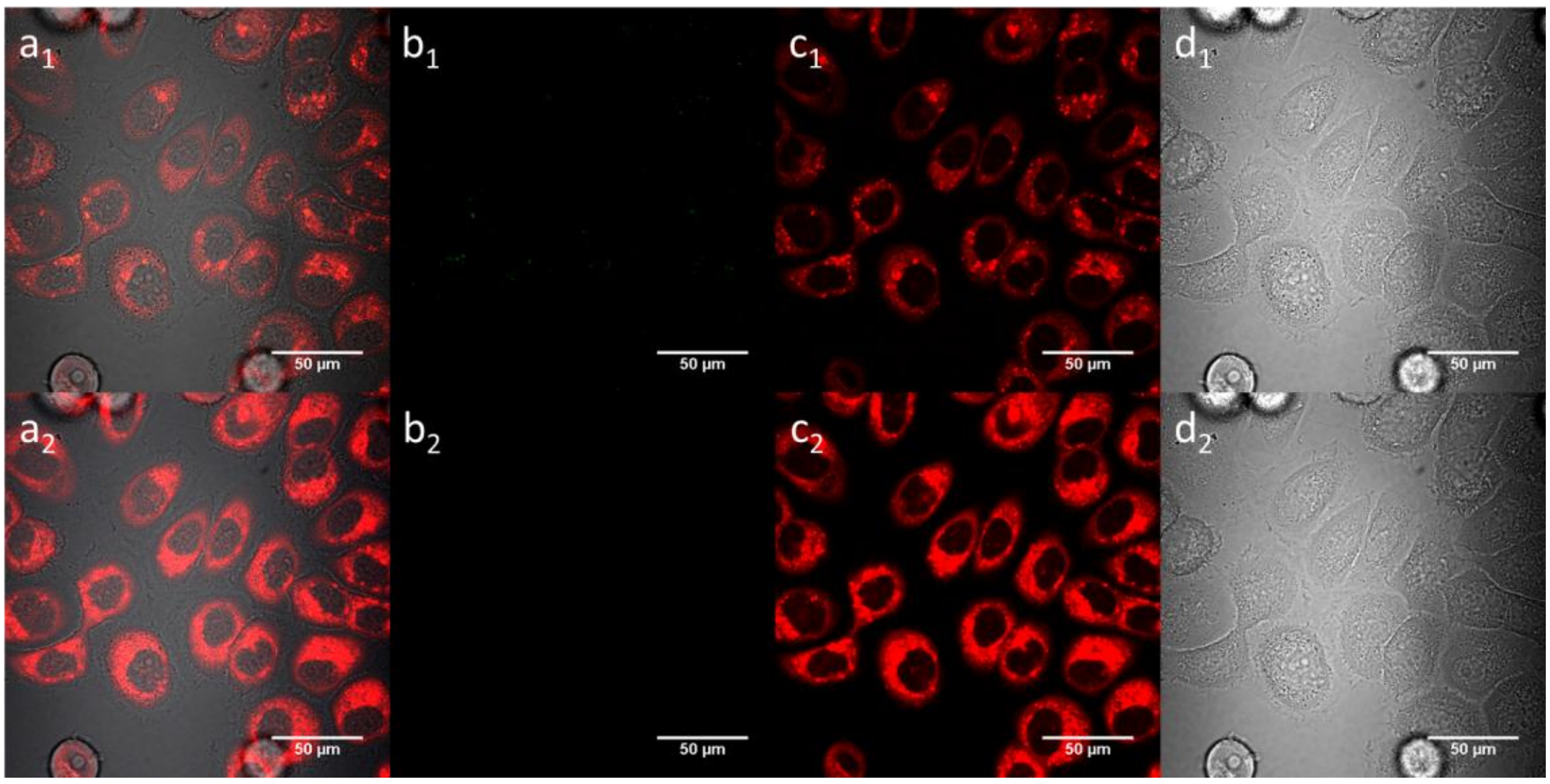

Figure S5.5. Confocal image of PC3 cells incubated with 100 nM Lysosome-tracker Red for 30 min at 37 °C; a_1-a_2 is the overlay images of green channel, red channel and DIC; b_1-b_2 is the green channel, excitation wavelength 488 nm (top row) 561 nm (bottom row), emission was at 516-530 nm; c_1-c_2 is the red channel, excitation at 488 nm (top row) and 561 nm (bottom row) and emission was at 615-650 nm; d_1-d_2 is the bright field channel.

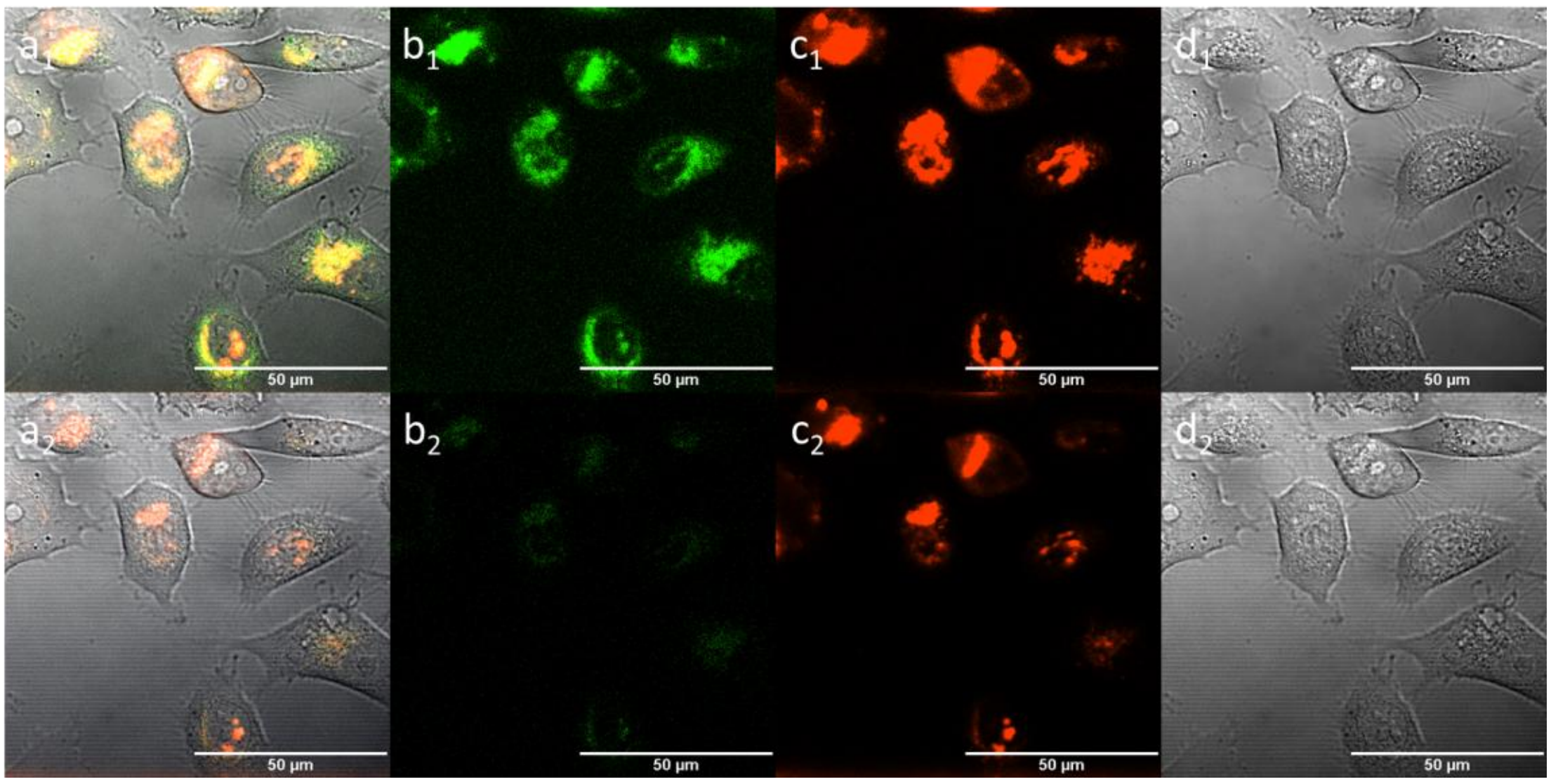

Figure S5.6. Confocal image of PC3 cells incubated with 200 nM Mito-tracker red for 30 min at 37 °C; a_1-a_2 is the overlay images of green channel, red channel and DIC; b_1-b_2 is the green channel, excitation wavelength 488 nm (top row) 561 nm (bottom row), emission was at 516-530 nm; c_1-c_2 is the red channel, excitation at 488 nm (top row) and 561 nm (bottom row) and emission was at 615-650 nm; d_1-d_2 is the bright field channel.

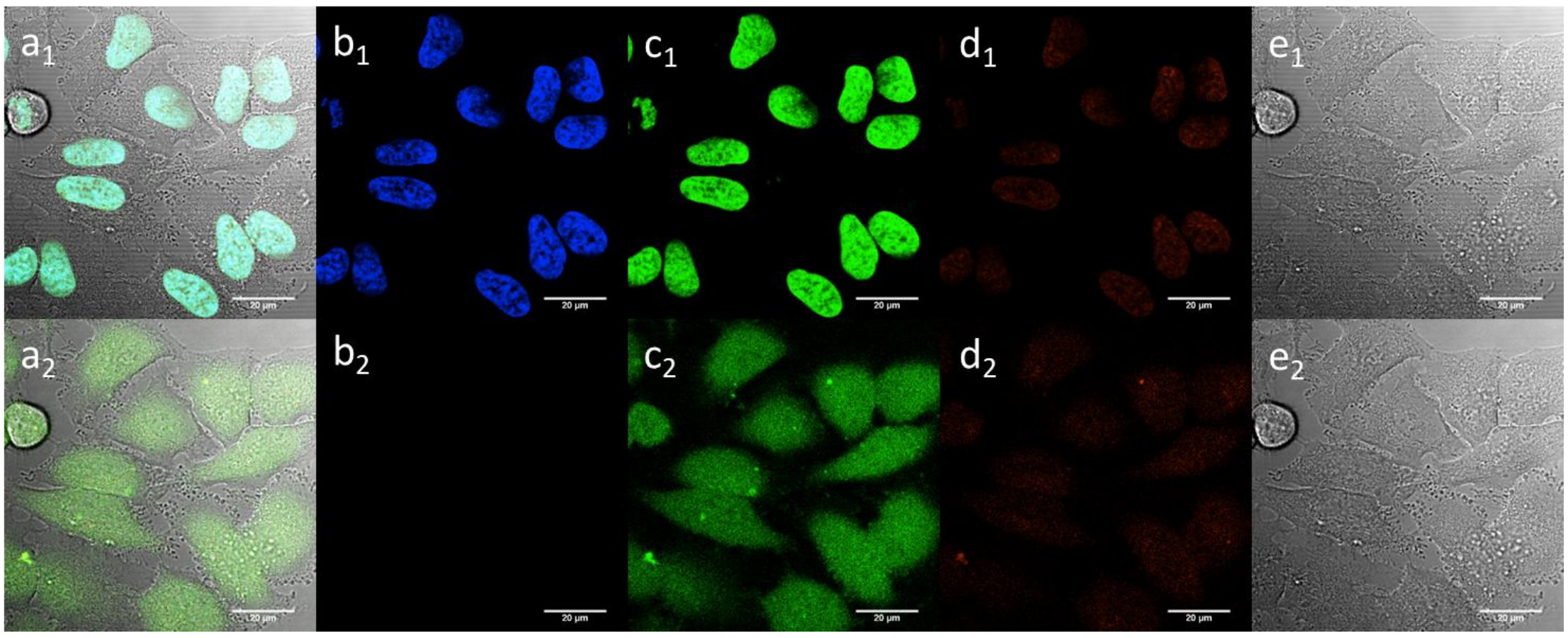

Figure S5.7. Confocal image of HeLa cells pre-incubated with 1 µg/mL Hoechst dye for 30 min at 37 °C; afterwards, cells were incubated with 100 µM compound **1**@glucan for 15 min at 37 °C; a_1-a_2 is the overlay images of blue channel, green channel, red channel and DIC; b_1-b_2 is blue channel, excitation was at 405 nm (top row) and 488 nm (bottom row), emission was at 420-480 nm; c_1-c_2 is the green channel, excitation was at 405 nm (top row) and 488 nm (bottom row), emission was at 516-530 nm; d_1-d_2 is the red channel, excitation was 405 nm (top row) and 488 nm (bottom row) and emission at 615-650 nm; e_1-e_2 is the bright field channel.

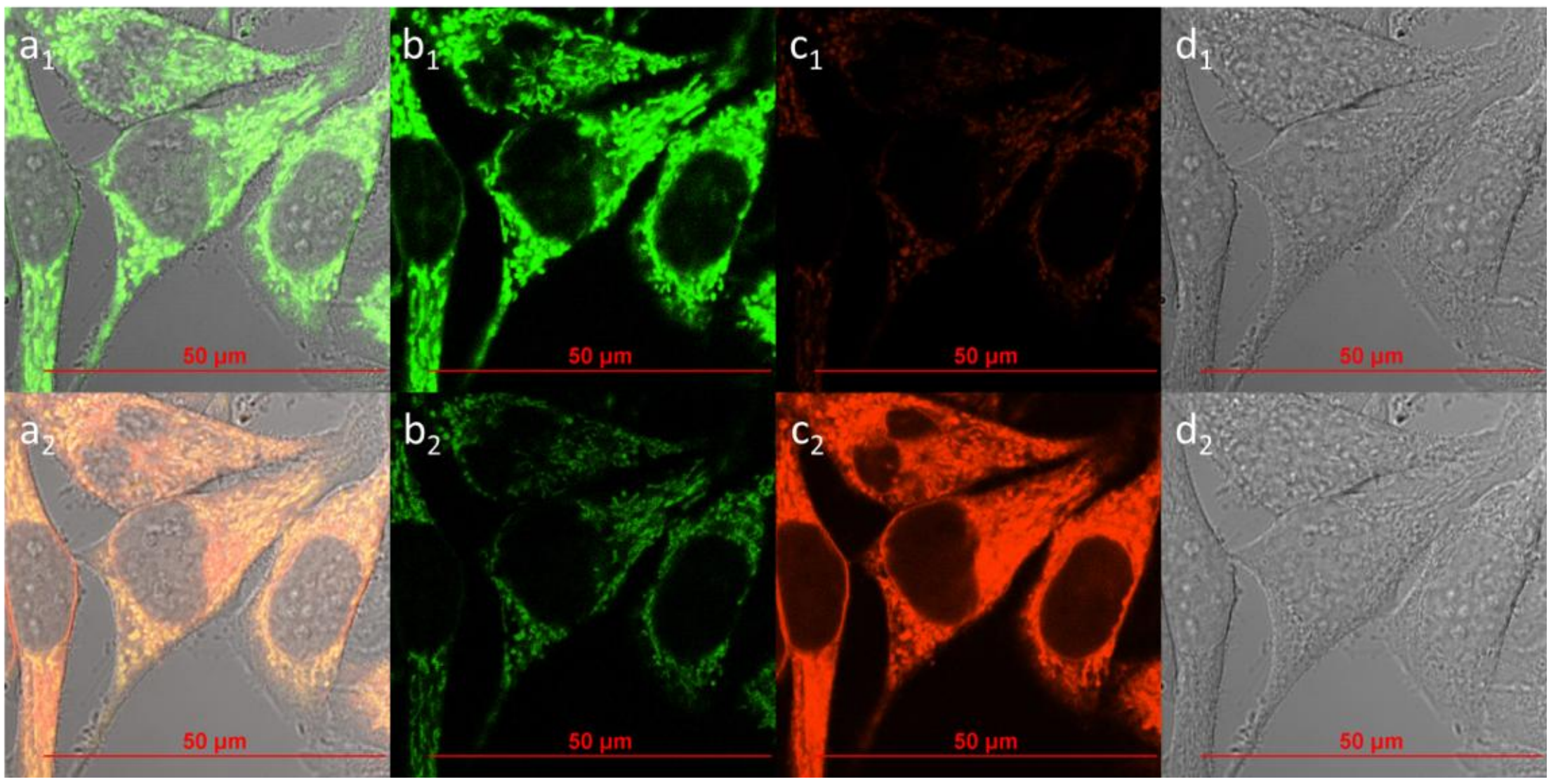

Figure S5.8. Confocal image of HeLa cells pre-incubated with 200 nM mito-tracker green for 30 min at 37 °C; afterwards, cells were incubated with 100 µM compound **3** for 15 min at 37 °C; a_1-a_2 is the overlay images of green channel, red channel and DIC; b_1-b_2 is the green channel, excitation was at 488 nm (top row) and 561 nm (bottom row), emission at 516-530 nm; c_1-c_2 is the red channel, excitation was at 488 nm (top row) and 561 nm (bottom row) and emission at 615-650 nm; d_1-d_2 is the bright-field channel.

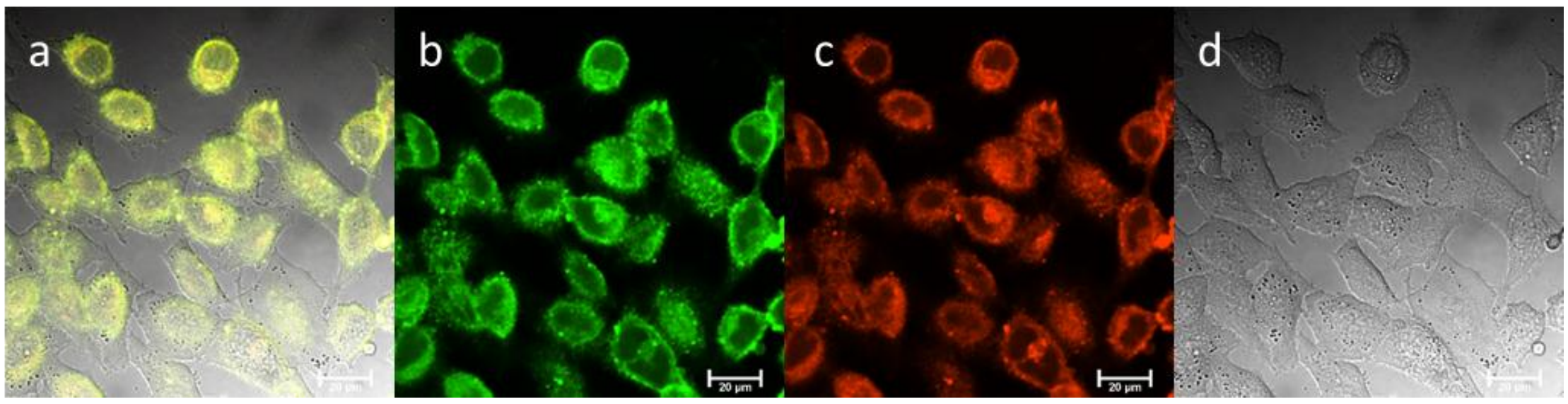

Figure S5.9. Confocal image of HeLa cells incubated with 100 nM lysosome-tracker red for 30 min at 37 °C; afterwards, cells were incubated with 100 µM compound **5** for 60 min at 37 °C; a is the overlay images of green channel, red channel and DIC; b is the green channel, excitation at 488 nm and emission at 516-530 nm; c is the red channel, excitation at 488 nm and emission at 615-650 nm; d is the bright field channel.

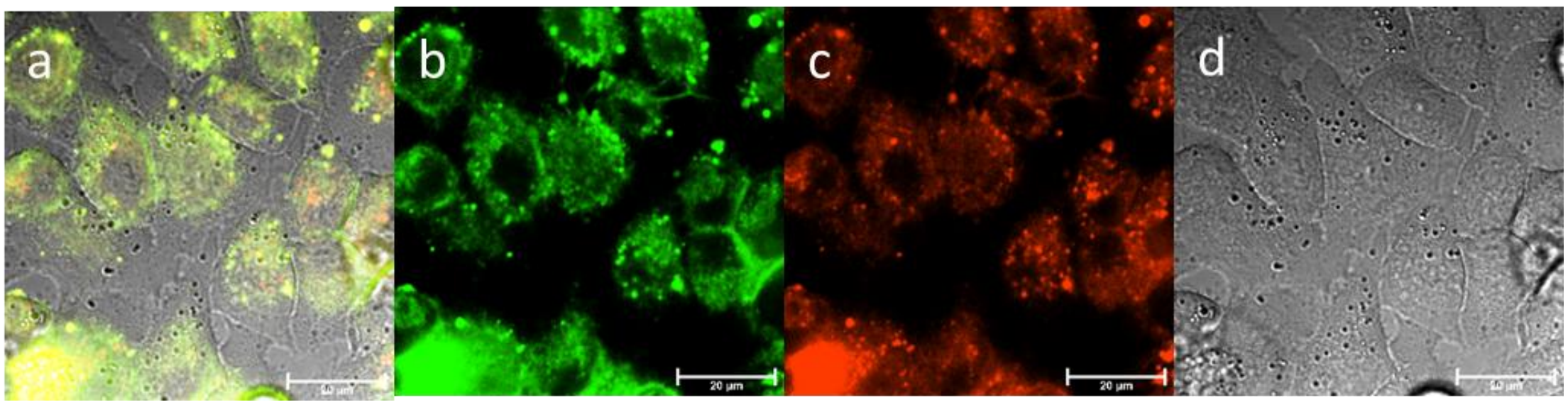

Figure S5.10. Confocal image of HeLa cells pre-incubated with 100 nM lyso-tracker green for 30 min at 37 °C; afterwards, cells were incubated with 100 µM compound **5** for 60 min at 37 °C; a is the overlay images of green channel, red channel and DIC; b is the green channel, excitation at 488 nm and emission at 516-530 nm; c is the red channel, excitation at 488 nm and emission at 615-650 nm; d is the bright field channel.

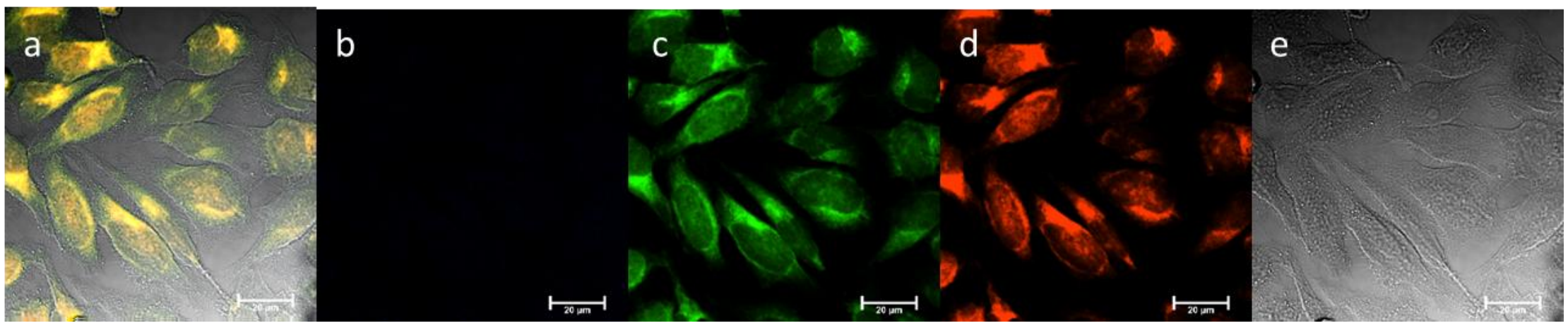

Figure S5.11. Confocal image of HeLa cells incubated with 100 nM lysosome-tracker red for 30 min at 37 °C; afterwards, cells are incubated with 100 µM compound **6** for 60 min at 37 °C; a is the overlay images of green channel, red channel and DIC; b is the green channel, excitation at 488 nm and emission at 516-530 nm; c is the red channel, excitation at 488 nm and emission at 615-650 nm; d is the bright field channel.

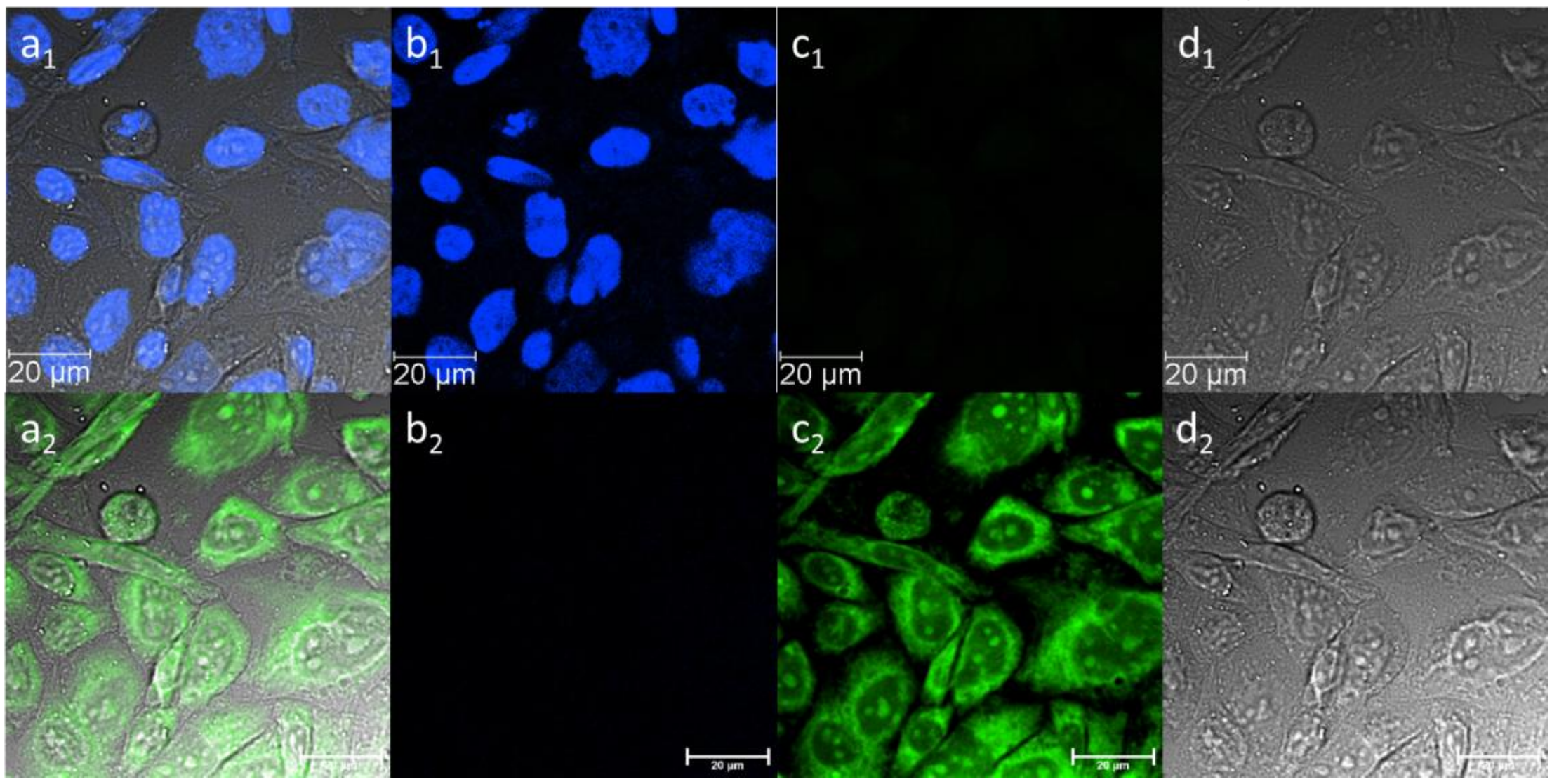

Figure S5.12. Confocal image of CHO cells pre-incubated with 1 µg/mL Hoechst dye for 30 min at 37 °C; afterwards, cells are incubated with 100 µM compound **6** for 15 min at 37 °C; a_1-a_2 is the overlay images of blue channel, green channel and DIC; b_1-b_2 is blue channel, compounds were excited at 405 nm and 488 nm, emitted at 420-480 nm; c_1-c_2 is the green channel, compounds were excited at 405 nm and 488 nm, emitted at 516-530 nm; d_1-d_2 is the bright field channel.

6. Two-photon fluorescence spectroscopy

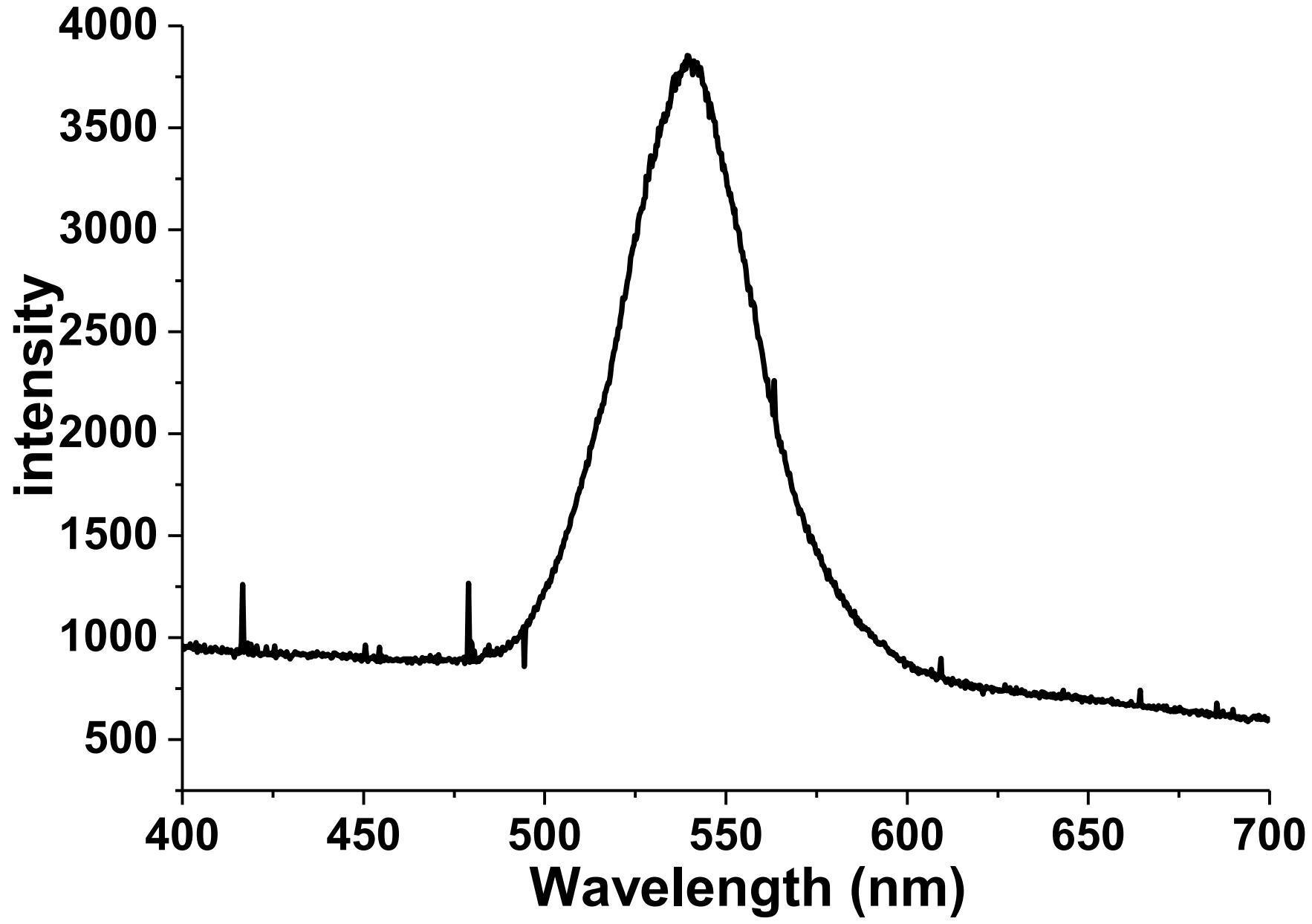

Figure S6.1. Two-photon fluorescence spectroscopy of compound **1**, 100 µM in DMSO at λ_{em} = 910 nm

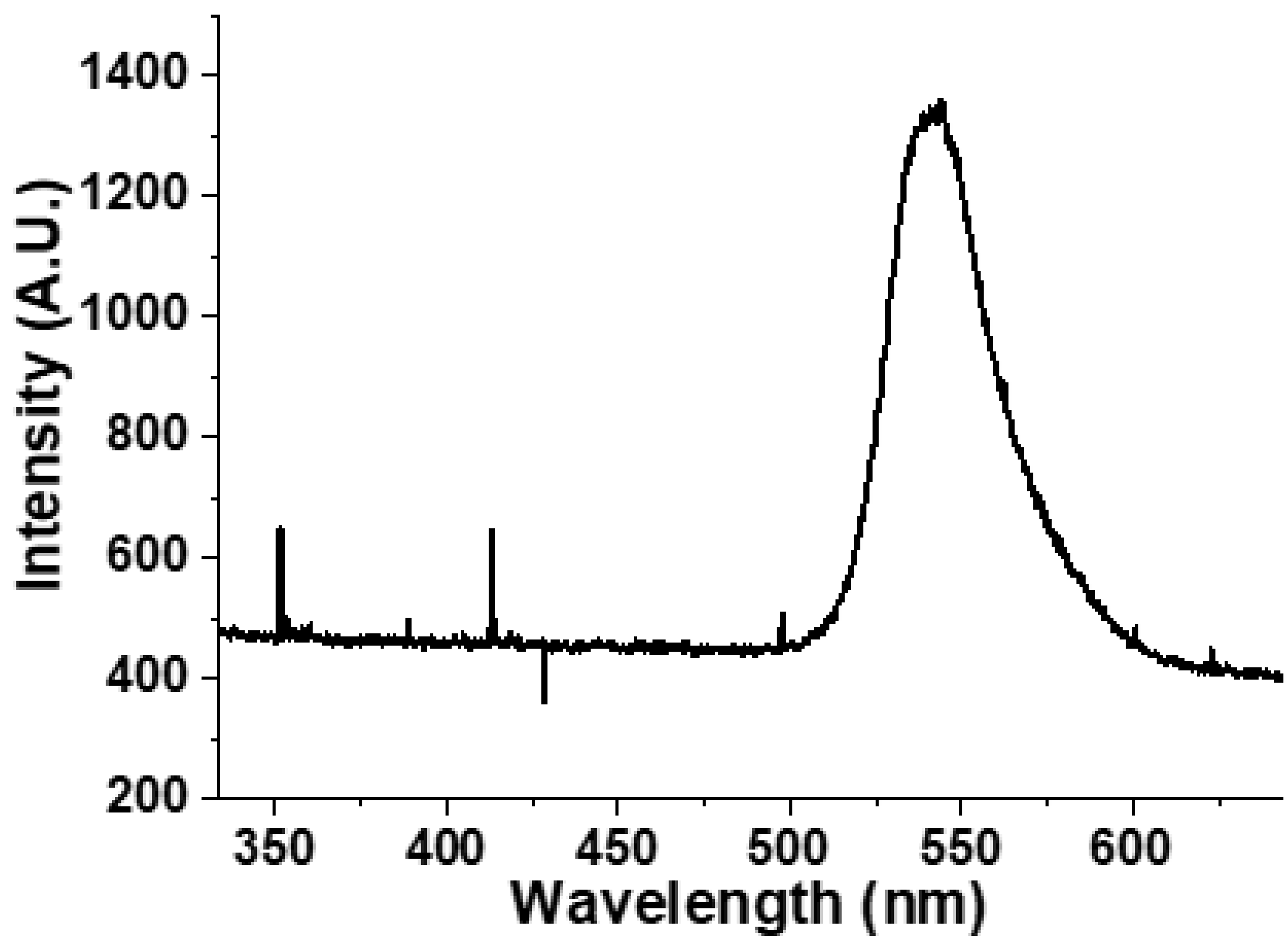

Figure S6.2. Two-photon fluorescence spectroscopy of compound **1** 100 µM in DMSO at λ_{em} = 810 nm

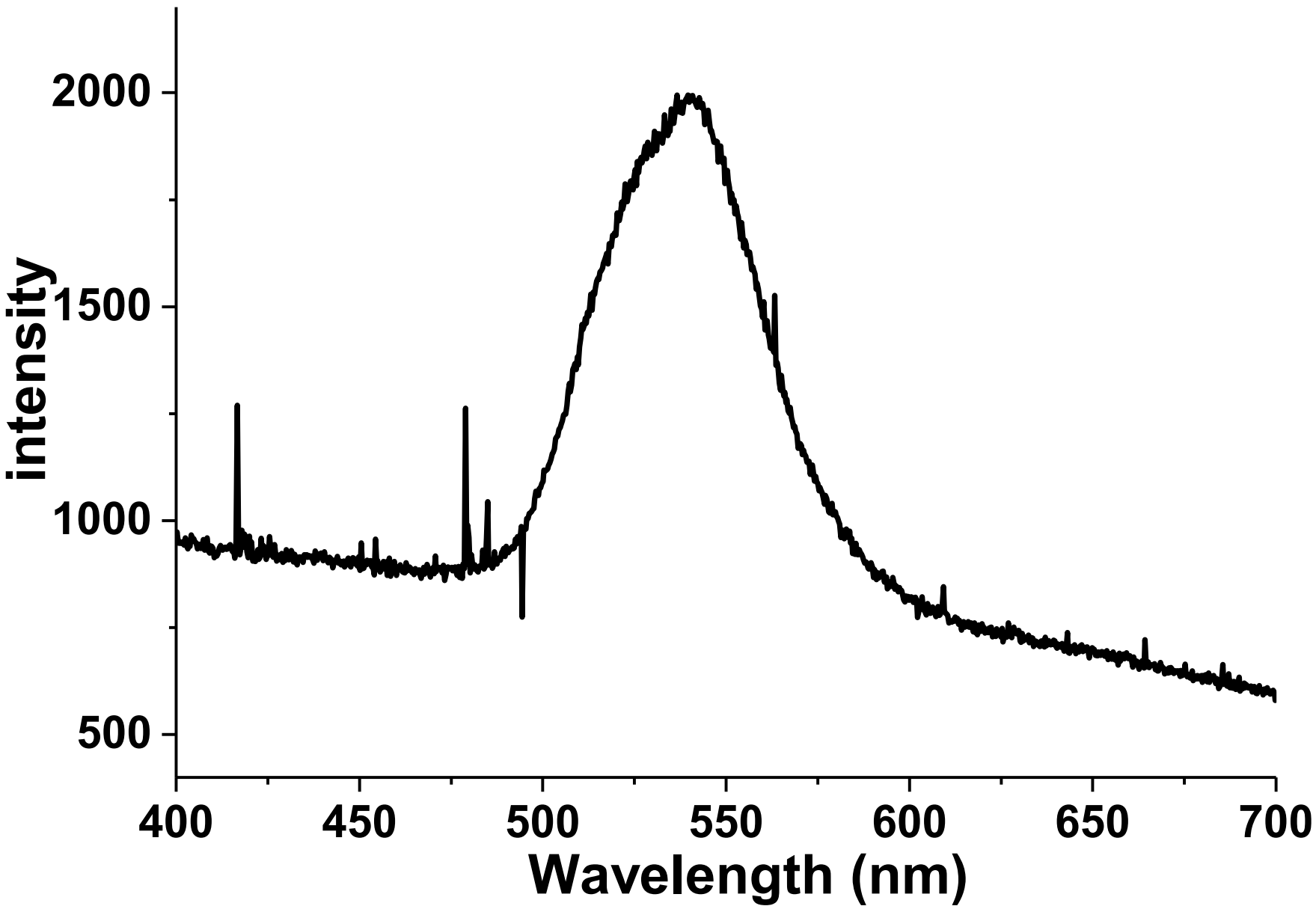

Figure S6.3. Two-photon fluorescence spectroscopy of **1@glucan** (1 mg/mL composite containing 100 μM of **1** in DMSO at λ_{em} = 910 nm)

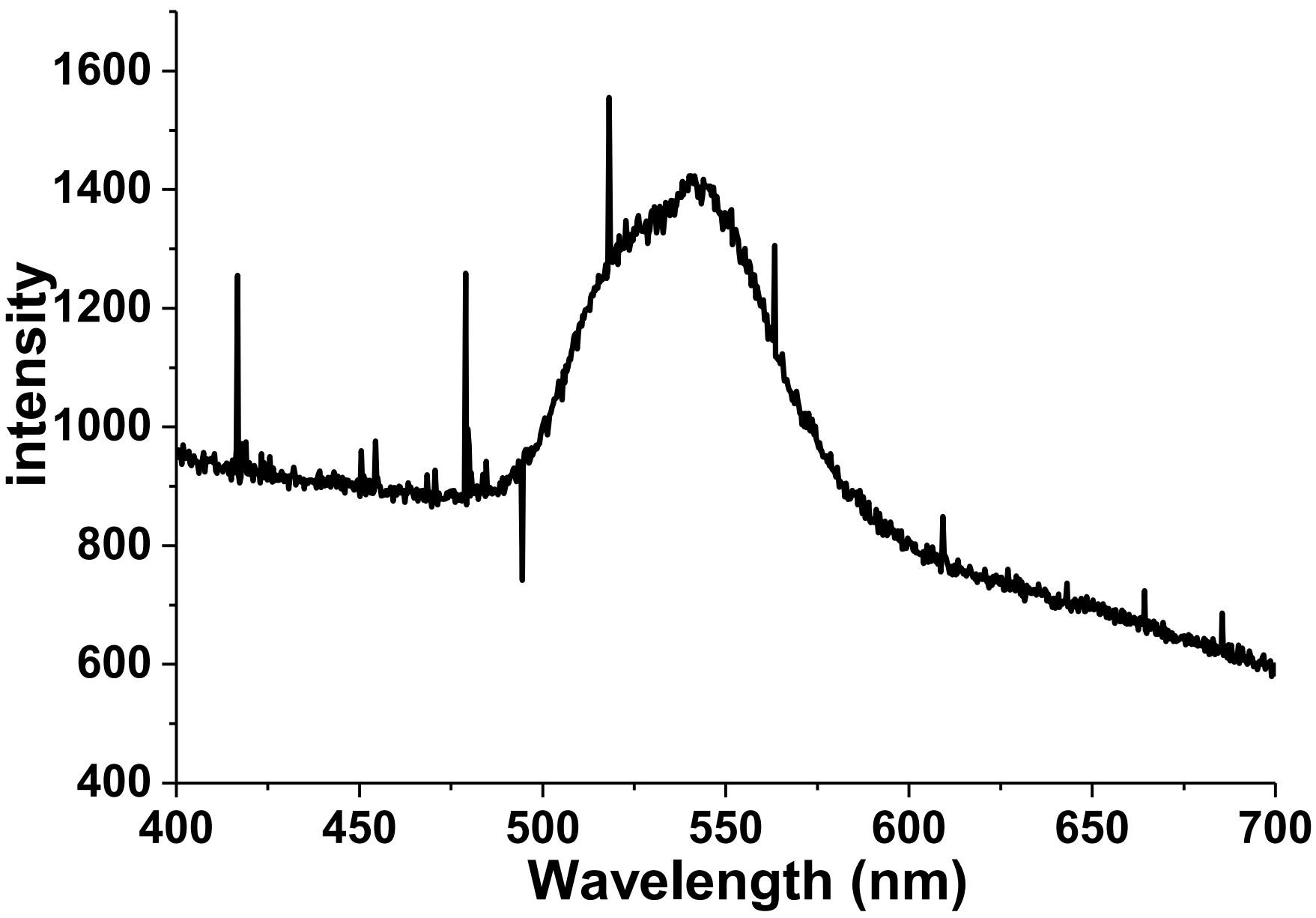

Figure S6.4. Two-photon fluorescence spectroscopy of compound **2** (100 μM DMSO at λ_{em} = 910 nm)

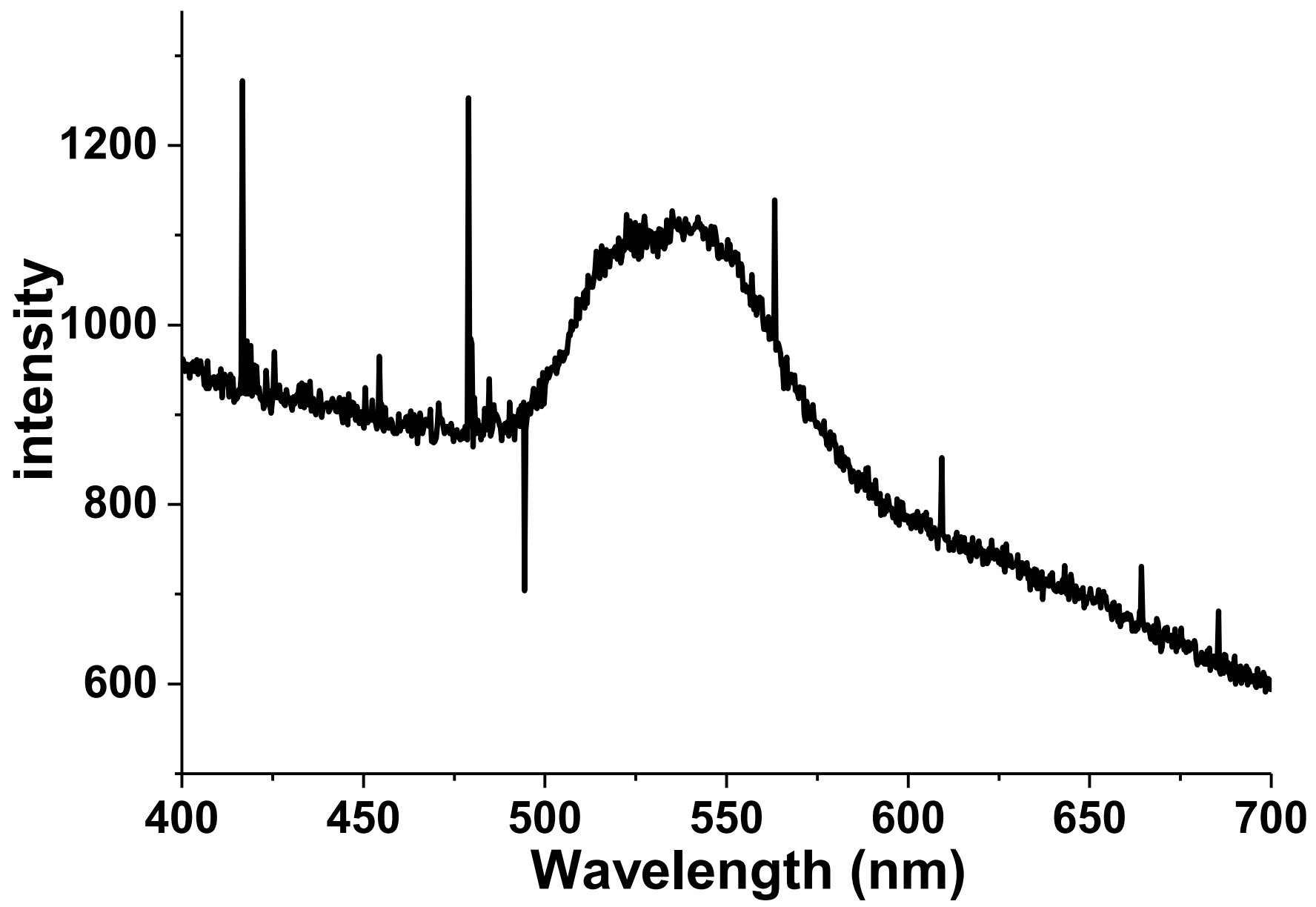

Figure S6.5. Two-photon fluorescence spectroscopy of **2**@glucan (1 mg/mL composite containing 100 μM of **2** in DMSO at λ_{em} = 910 nm).

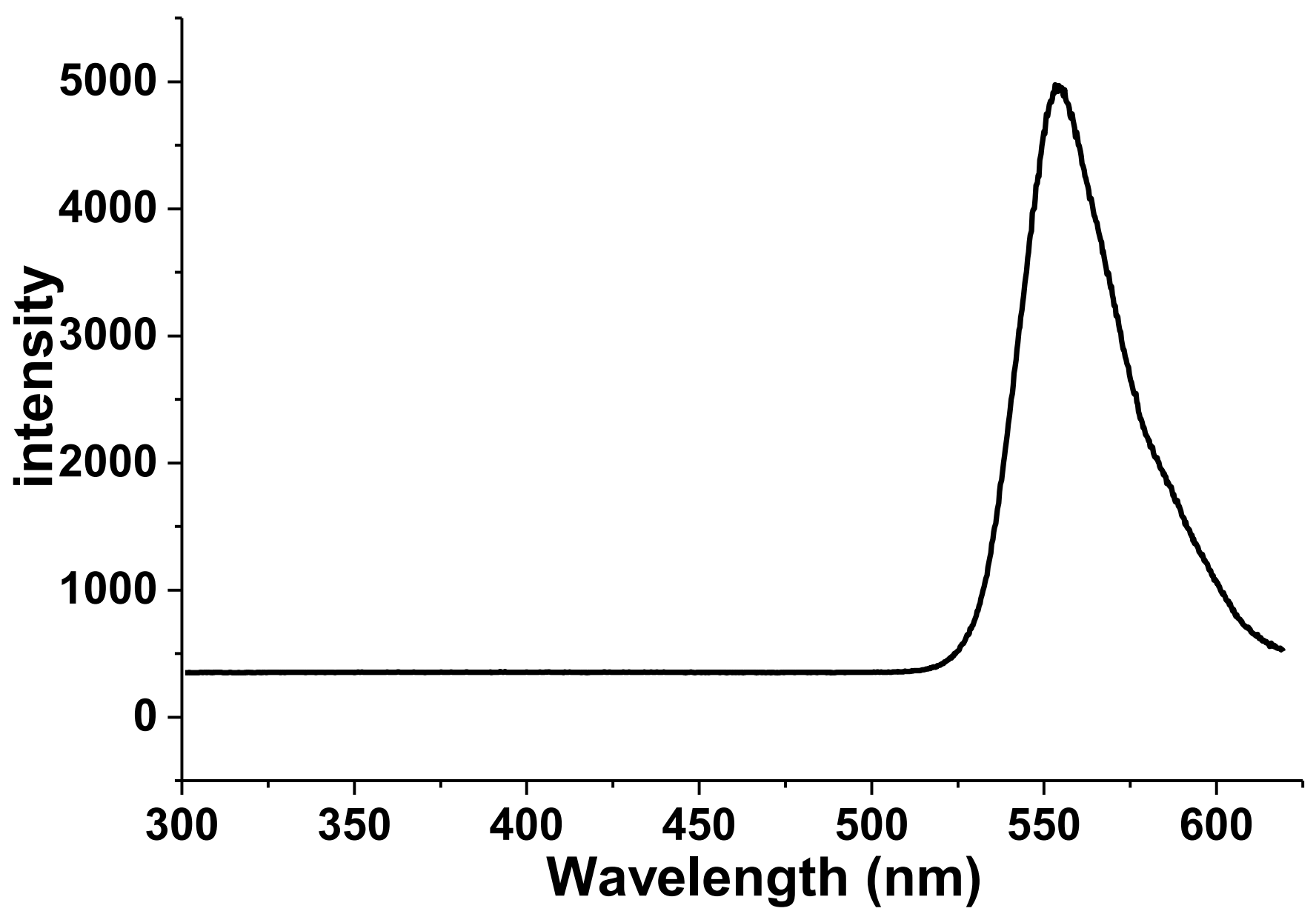

Figure S6.6. Two-photon fluorescence spectroscopy of compound **3** (1 mM) in DMSO at λ_{em} = 910 nm

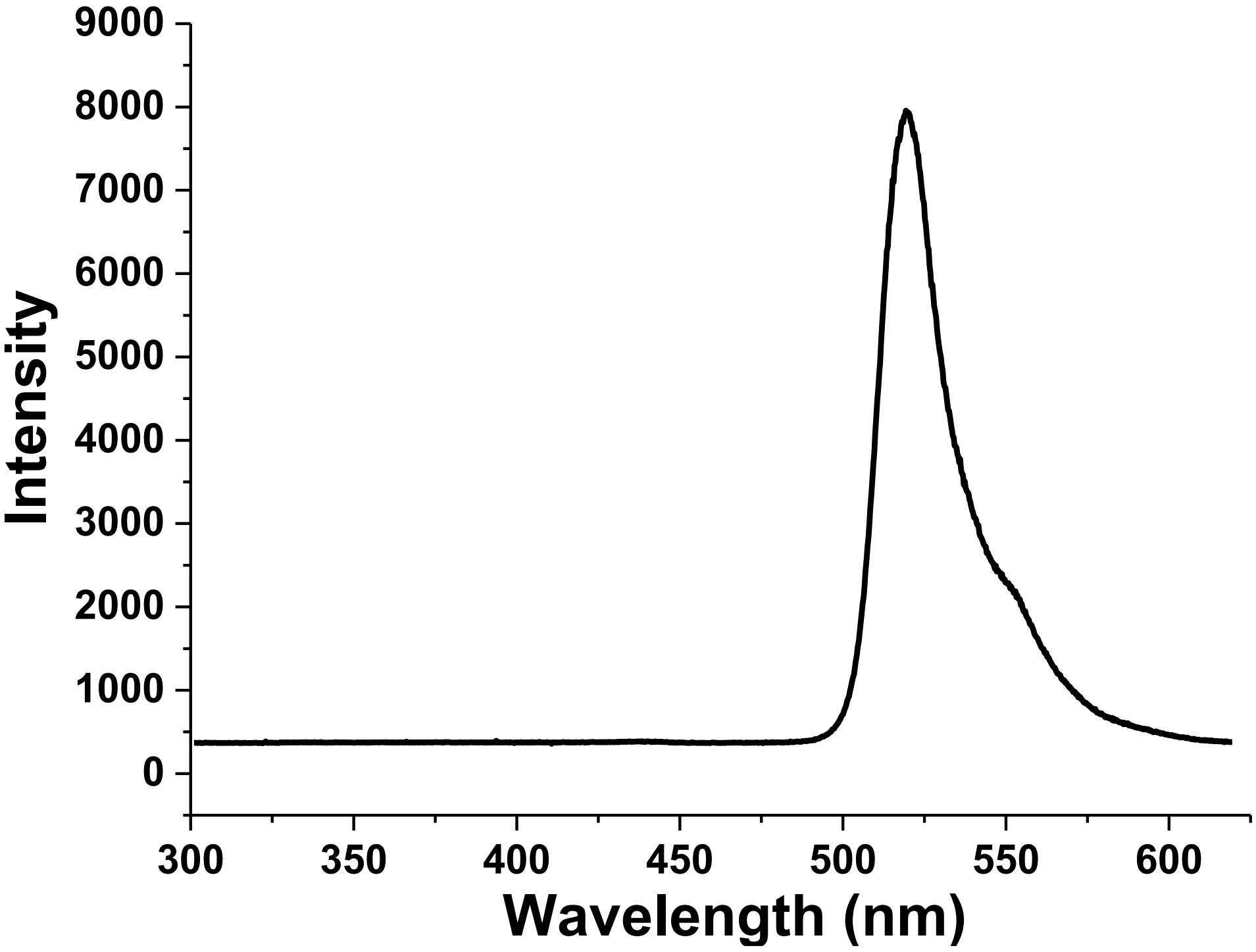

Figure S6.7. Two-photon fluorescence spectroscopy of compound **4** (1 mM) in DMSO

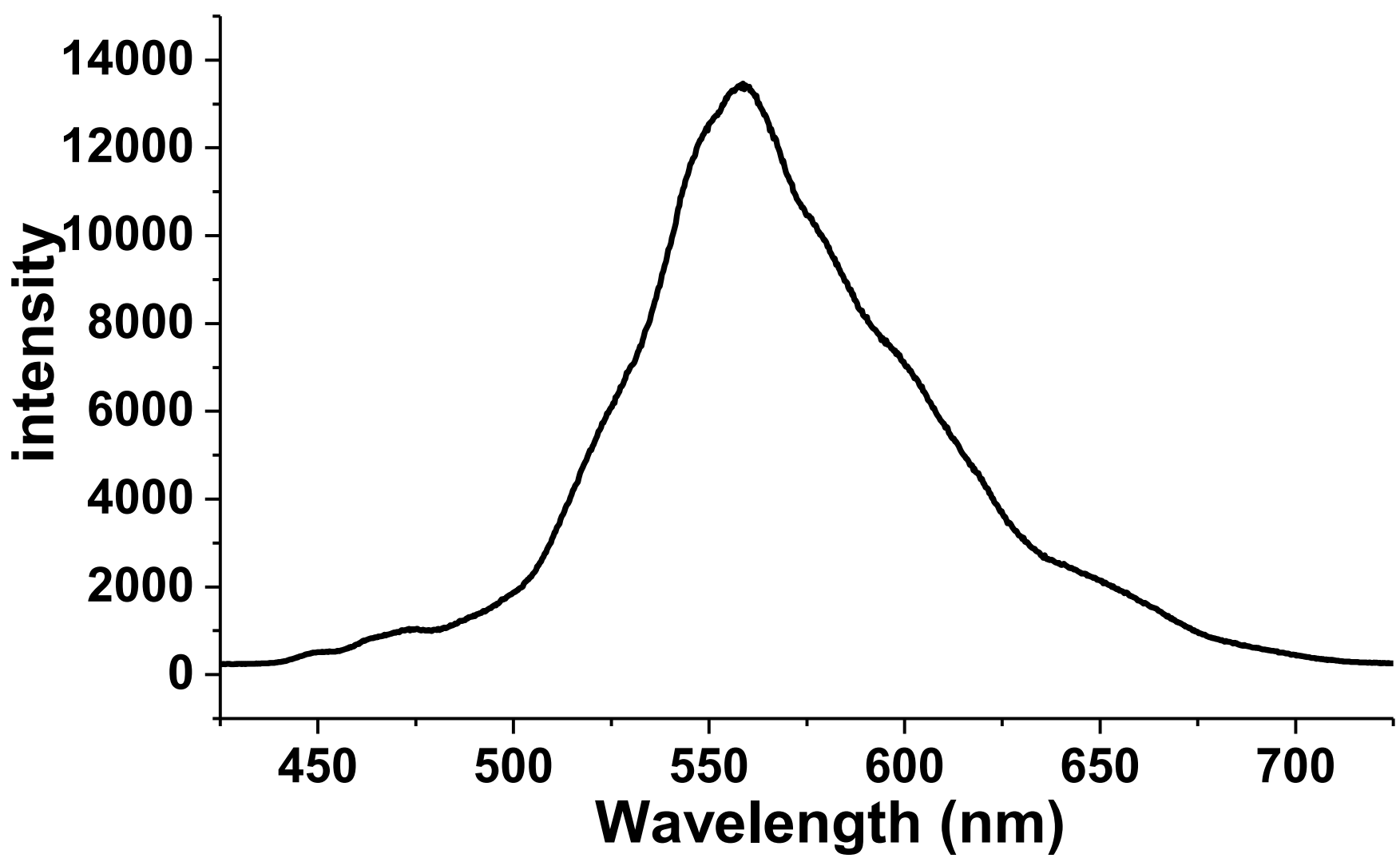

Figure S6.8. Two-photon fluorescence spectroscopy of compound **5** (10 mM) in DMSO at λ_{em} = 910 nm

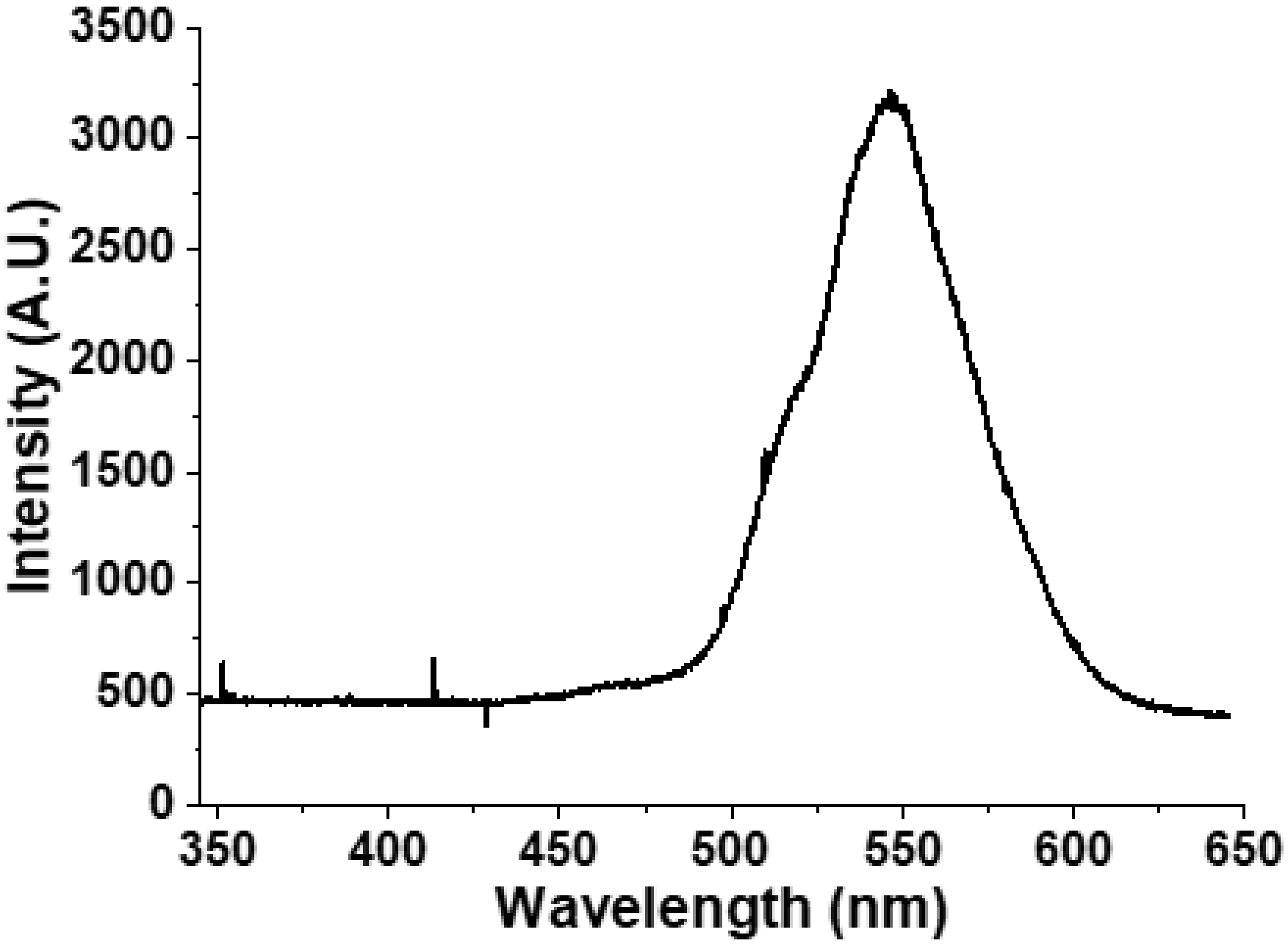

Figure S6.9. Two-photon fluorescence spectroscopy of compound **5** (500 μM) in DMSO at λ_{em} = 810 nm

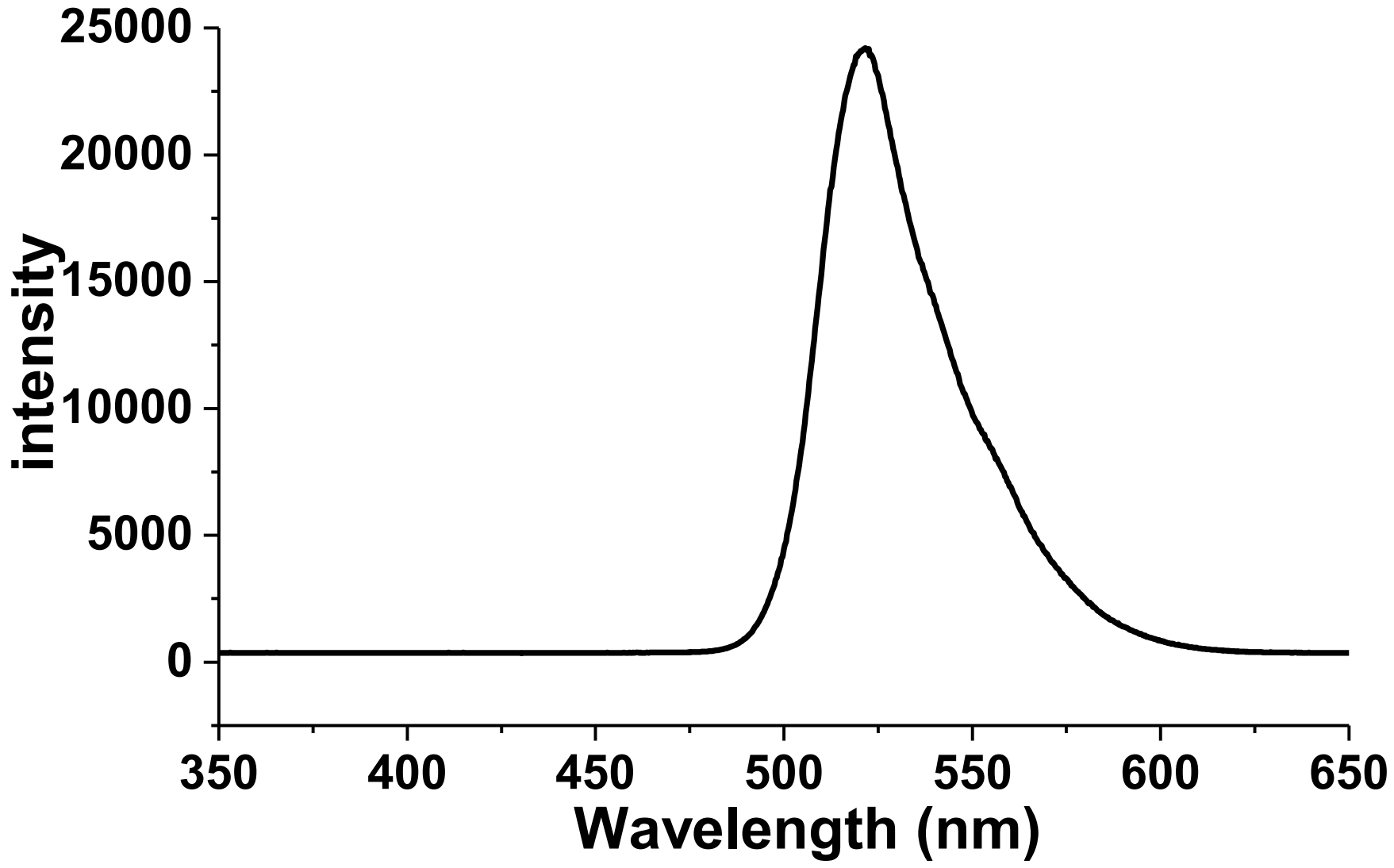

Figure S6.10. Two-photon fluorescence spectroscopy of compound **6** (1 mM) in DMSO at λ_{em} = 910 nm

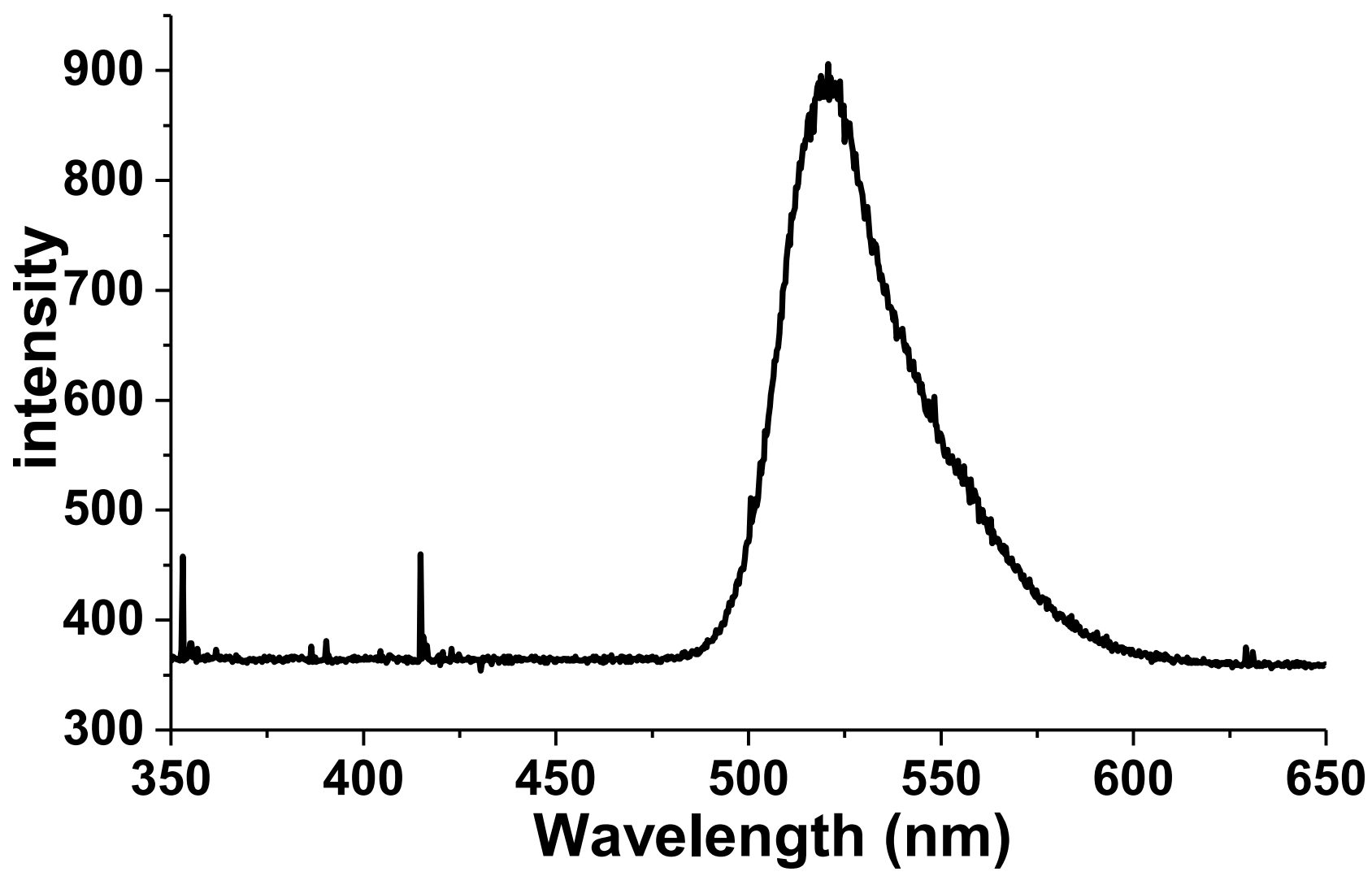

Figure S6.11. Two-photon fluorescence spectroscopy of **6**@glucan (1 mg/mL composite containing 100 μM of **6)** in DMSO at λ_{em} = 910 nm

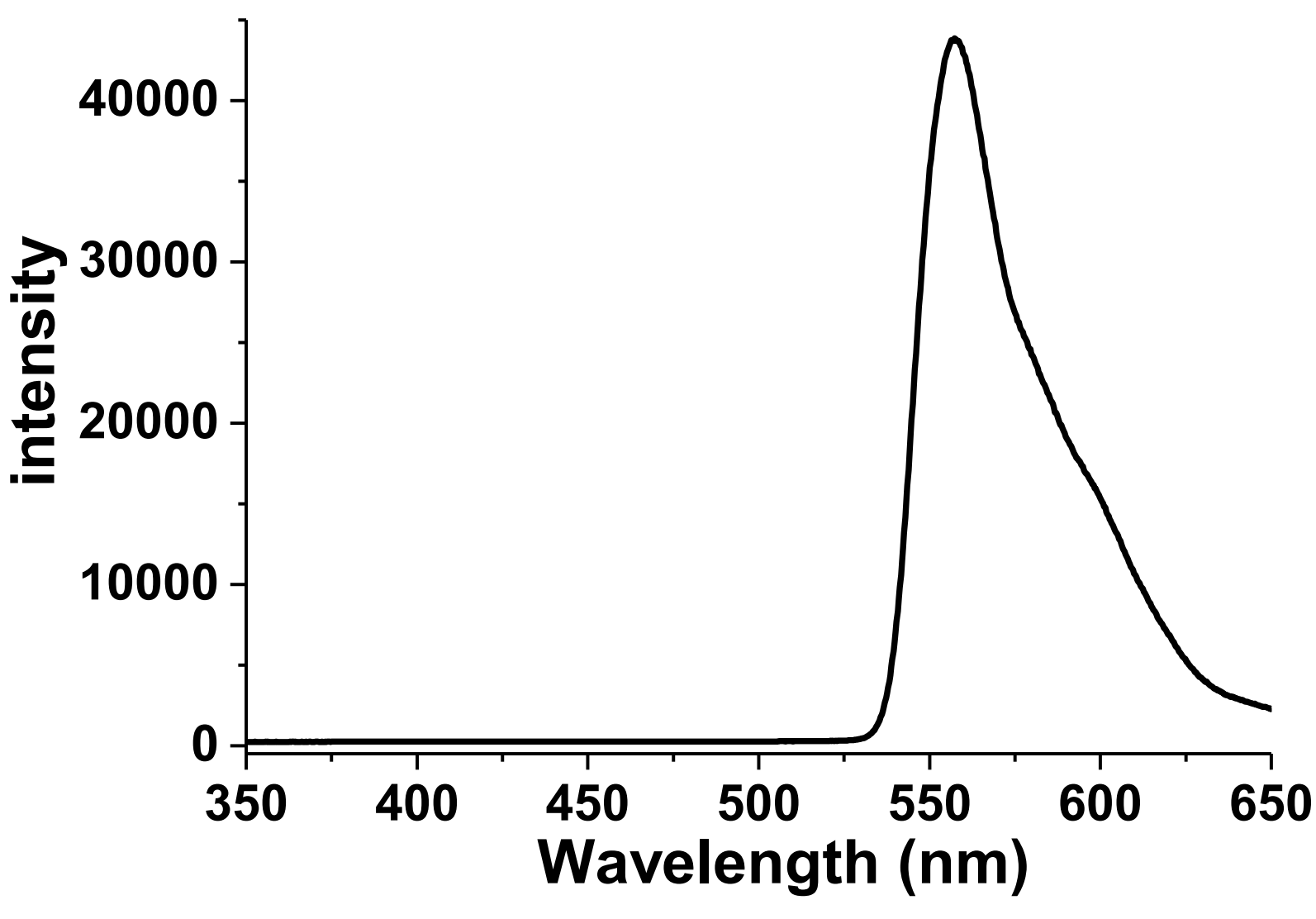

Figure S6.12. Two-photon fluorescence spectroscopy of compound **V** (1 mM) in DMSO at λ_{em} = 910 nm

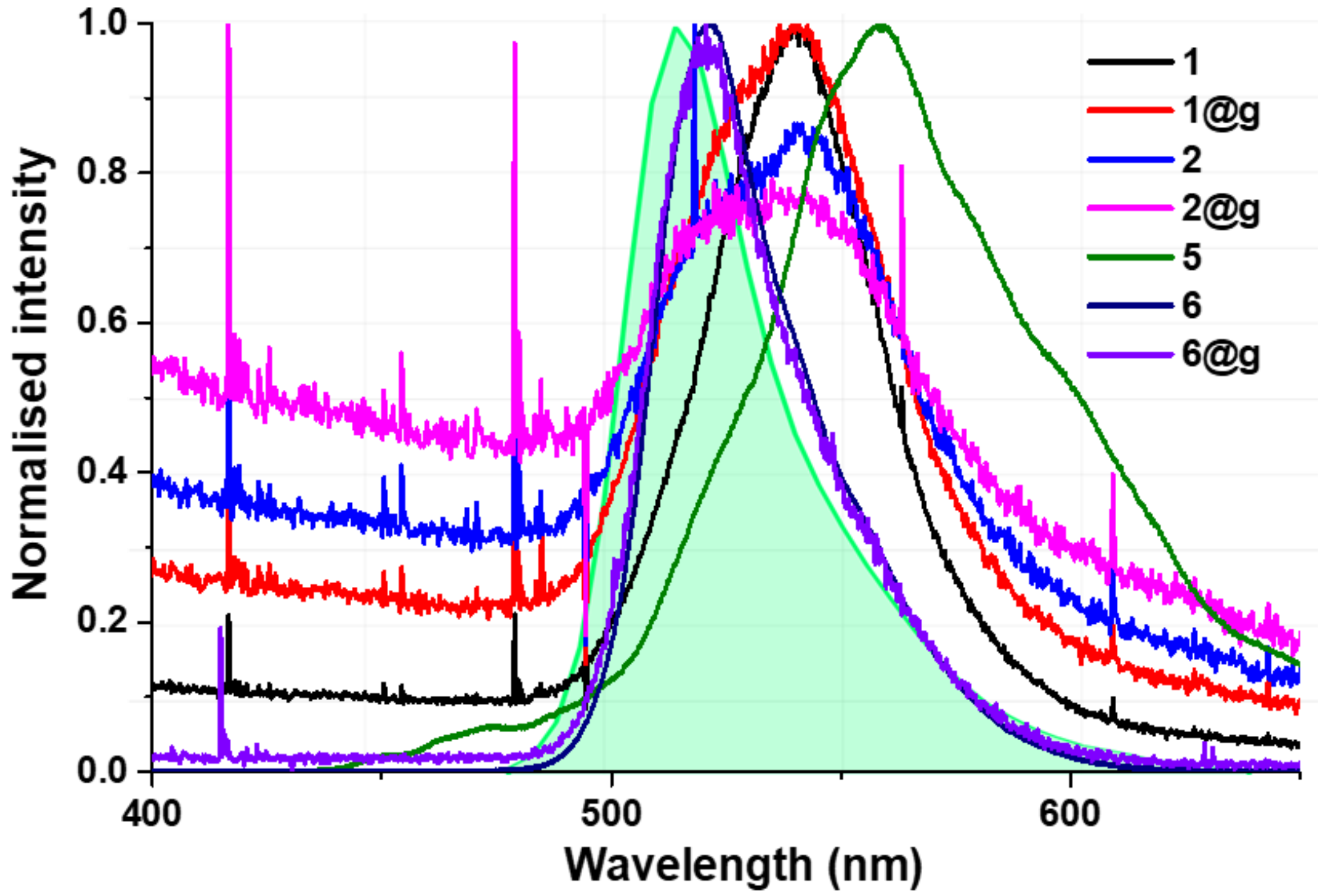

Figure S6.13 Normalised 2P- fluorescence emission of FITC based boronic acid fluorophores and their glucan hybrids compared with the commercially available FITC (1 mM in DMSO, green band).

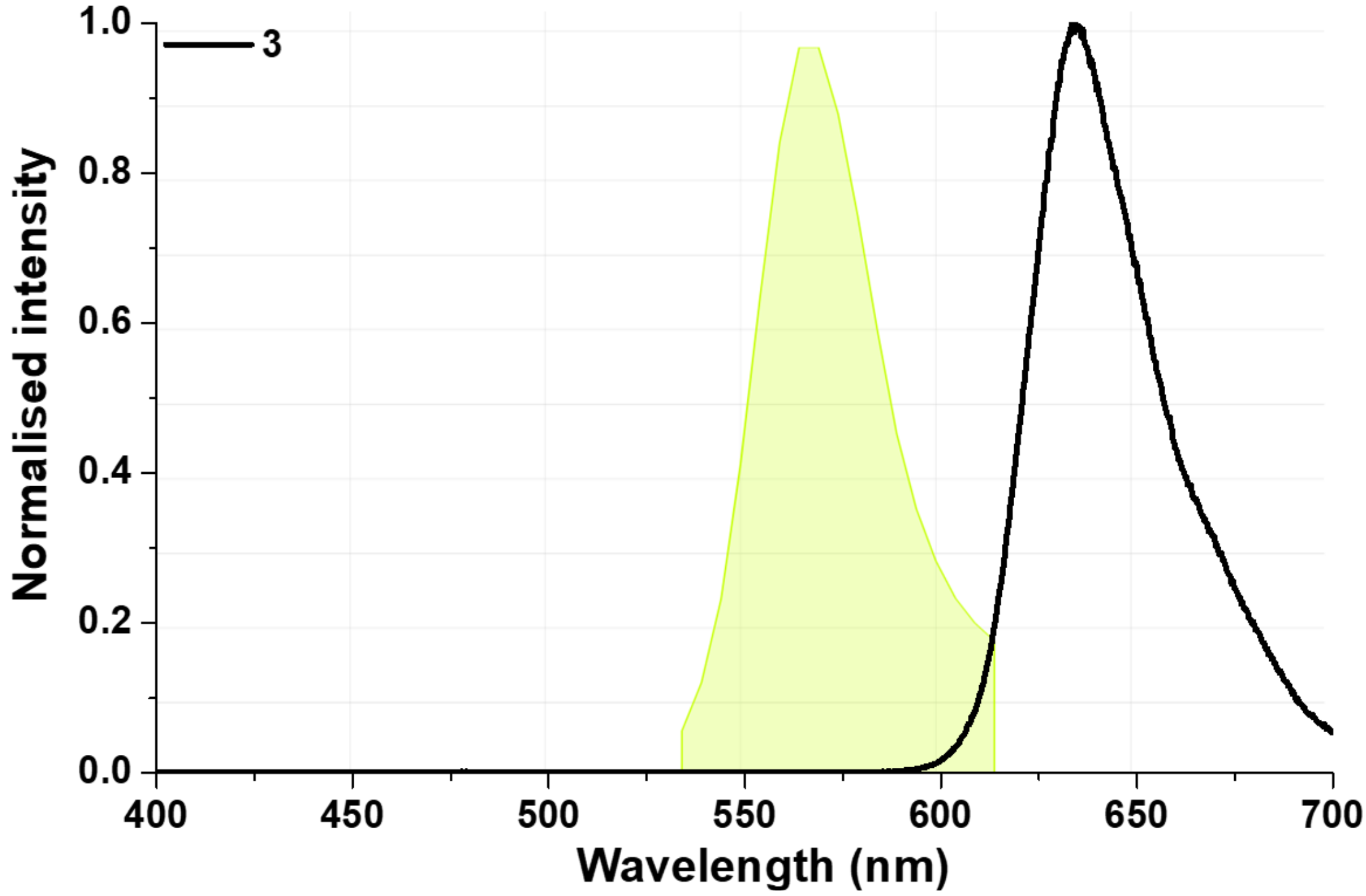

Figure S6.14 Normalised 2P- fluorescence emission of Rhodamine based fluorophore **3** compared with the commercially available Rhodamine B (yellow, 1 mM in DMSO).

7. Fluorescence lifetime imaging microscopy

Experimental preparation for confocal fluorescence imaging:

In standard protocols, 300,000 cells in 2 mL cell medium were added to glass bottom Petri dish suitable for microscopy and left for 24 to 48 hours to adhere. Once cells attached firmly, cells were washed with 990 µL PBS three times and refilled with 990 µL of serum free medium (SFM), then, in each case 10 µL of compound from the 10 mM stocks solutions were added. Cells were incubated with compounds for 15 minutes at 37 °C. Afterward, cells washed with 990 µL PBS three times to rinse the compound remain in the medium and 990 µL of SFM was added. Once the cell dish was ready for the confocal fluorescence imaging, cells were excited at 405 nm, 488 nm and 561 nm wavelength, then at each wavelength, there were five images captured namely a merged image, image between 420 and 480 nm wavelength (blue), image between 516 and 530 nm wavelength (green), image between 615 and 650 nm wavelength (red) and Differential Interference Contrast (DIC) image (no fluorescence). The region of interest (ROI) was linked *via* a time-correlated single-photon-counting PC module SPC830 (Becker and Hickl) to generate the multiphoton excited image with the associated characteristic decay at each pixel position. The decays were subsequently analysed to generate the FLIM images.

Time-correlated single-photon counting (TCSPC): Lifetime measurements were recorded using a Becker and Hickl SPC830 TCSPC PCI module with an excitation wavelength of 810 nm or 910 nm and the emission measured at 360-580 nm (BG39, Comar Optics, UK). The measurement of compounds was carried out in DMSO/ water solvent with concentration of 10 mM or less. Emission spectral detection was carried out using an Action Research Component 275 spectrograph and an Andor iDus 740-BU CCD camera.

Fluorescence lifetime imaging microscopy (FLIM): Cell uptake studies were performed using HeLa, PC-3 and CHO cells. Cells were plated on a glass petri dishes and left cells to attach for 12-24 hours depending on the growth rate. Control FLIM were recorded before the addition of compound. Plates were then mounted on the microscope stage and kept at 37 ºC.

Compounds were dissolved in DMSO and added to cells to achieve final concentration of 100 µM in EMEM/RPMI medium containing 1% of DMSO. Cells were incubated for 15 minutes prior to confocal imaging, FLIM was carried out immediately after the confocal imaging with the same area as the confocal imaging. The fluorescence decay curve was fitted to a multiexponential function as:

$$I(t)=\sum_{i=1}^{M} a_i \, exp\left(\frac{-t}{\tau_i}\right)$$

Where I(t) is the fluorescence decay, τ_i is the lifetime of M exponential component and a_i is the amplitudes of the fluorescence decay.

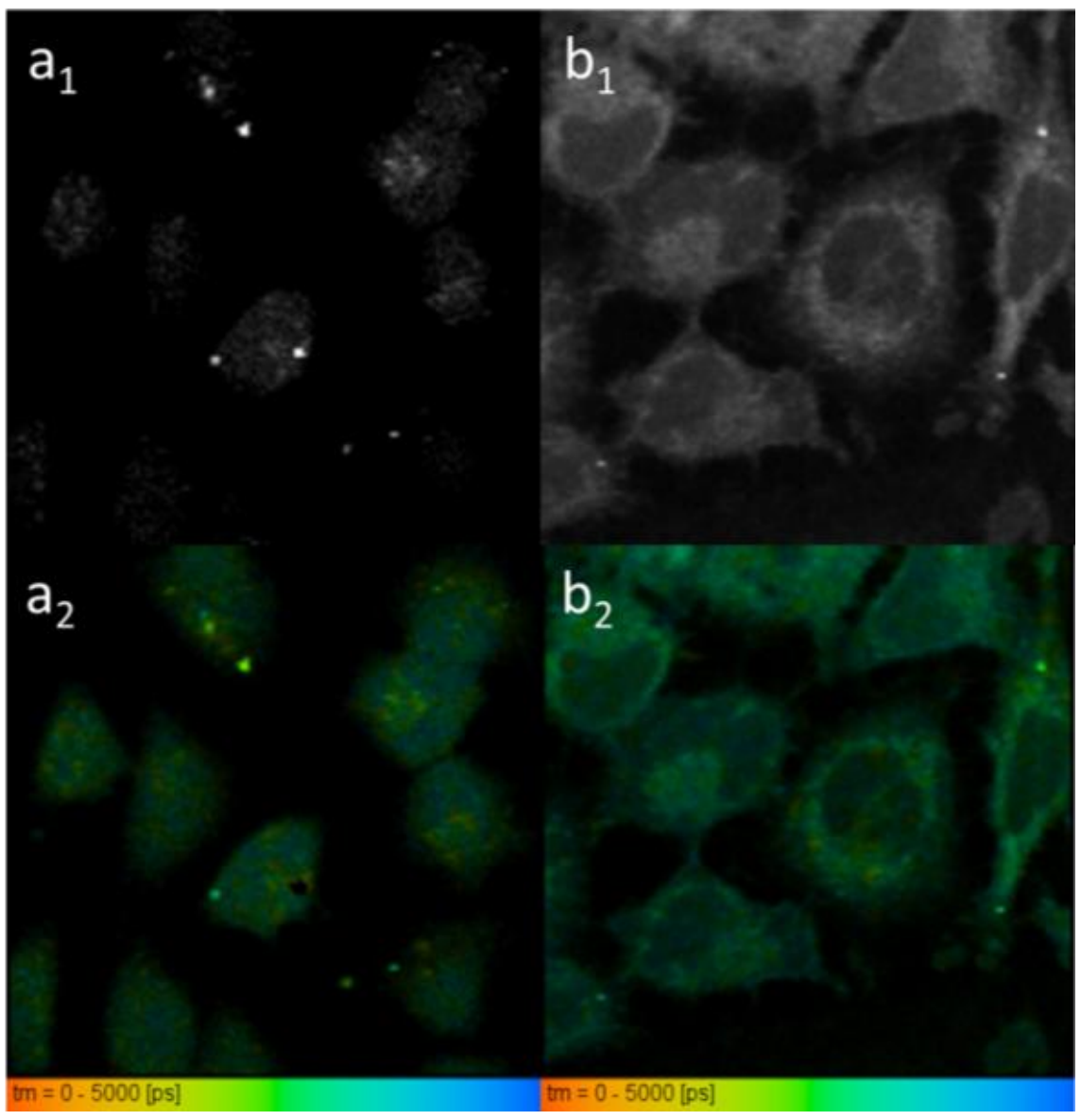

Figure S7.1. FLIM images of cells treated with 100 μM of compounds in 1% DMSO, cells were incubated at 37 ºC or 4 ºC, where (a) **1**, 37 ºC (b) **2**, 37 ºC, λex = 910 nm. a_1-b_1 is the two-photon fluorescence intensity diagram of compounds, a_2-b_2 is the fluorescence lifetime map of τ_m of compounds in HeLa cells. Scale bar: τ_m=0-5000 ps.

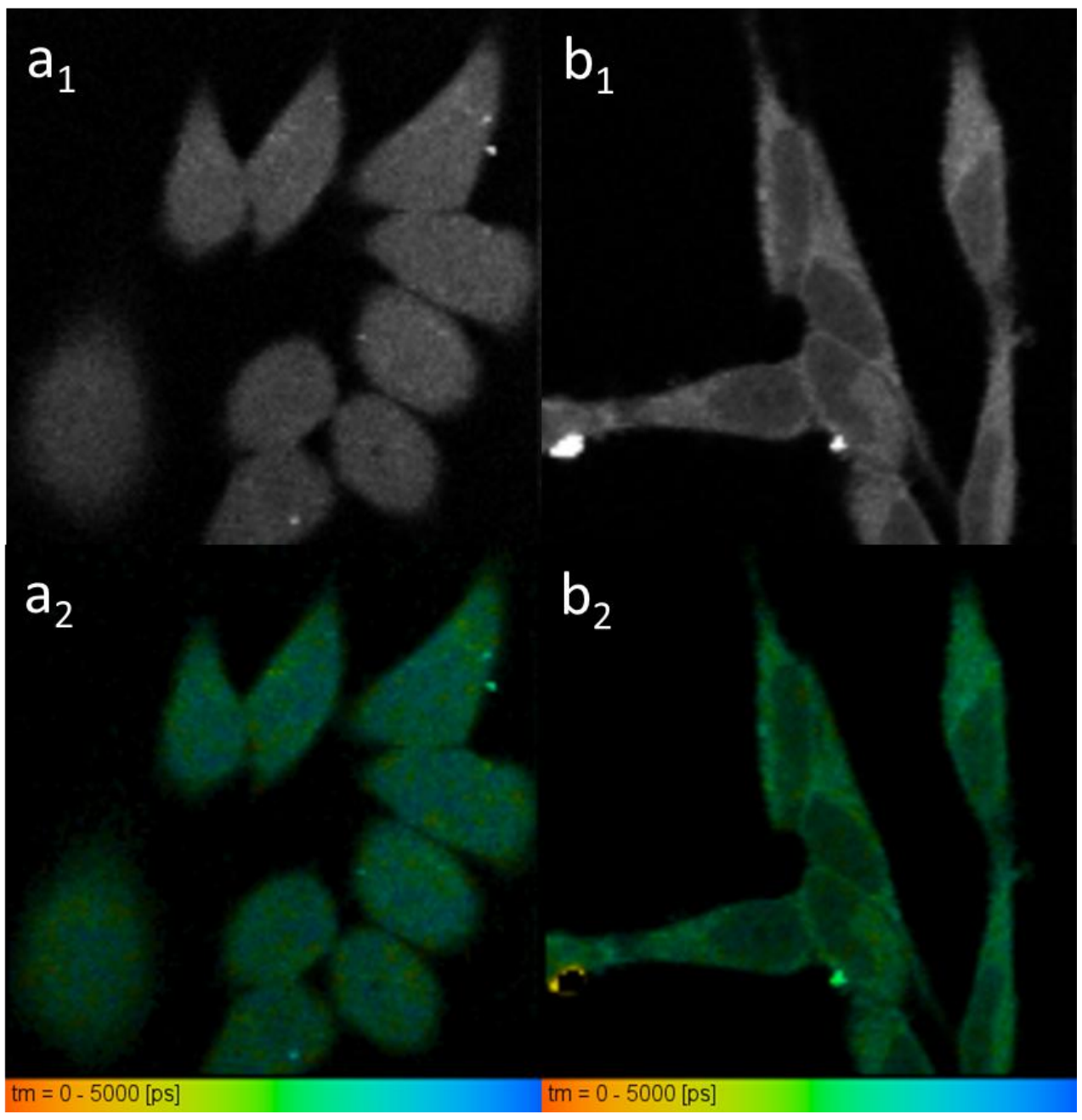

Figure S7.2. FLIM images of cells treated with 100 μM of compounds in 1% DMSO, cells were incubated at 37 ºC or 4 ºC, where (a) **1**@glucan, 37 ºC (b) **2**@glucan, 37 ºC, λex = 910 nm. a_1-b_1 is the two-photon fluorescence intensity diagram of compounds, a_2-b_2 is the fluorescence lifetime map of τ_m of compounds in HeLa cells. Scale bar: τ_m=0-5000 ps.

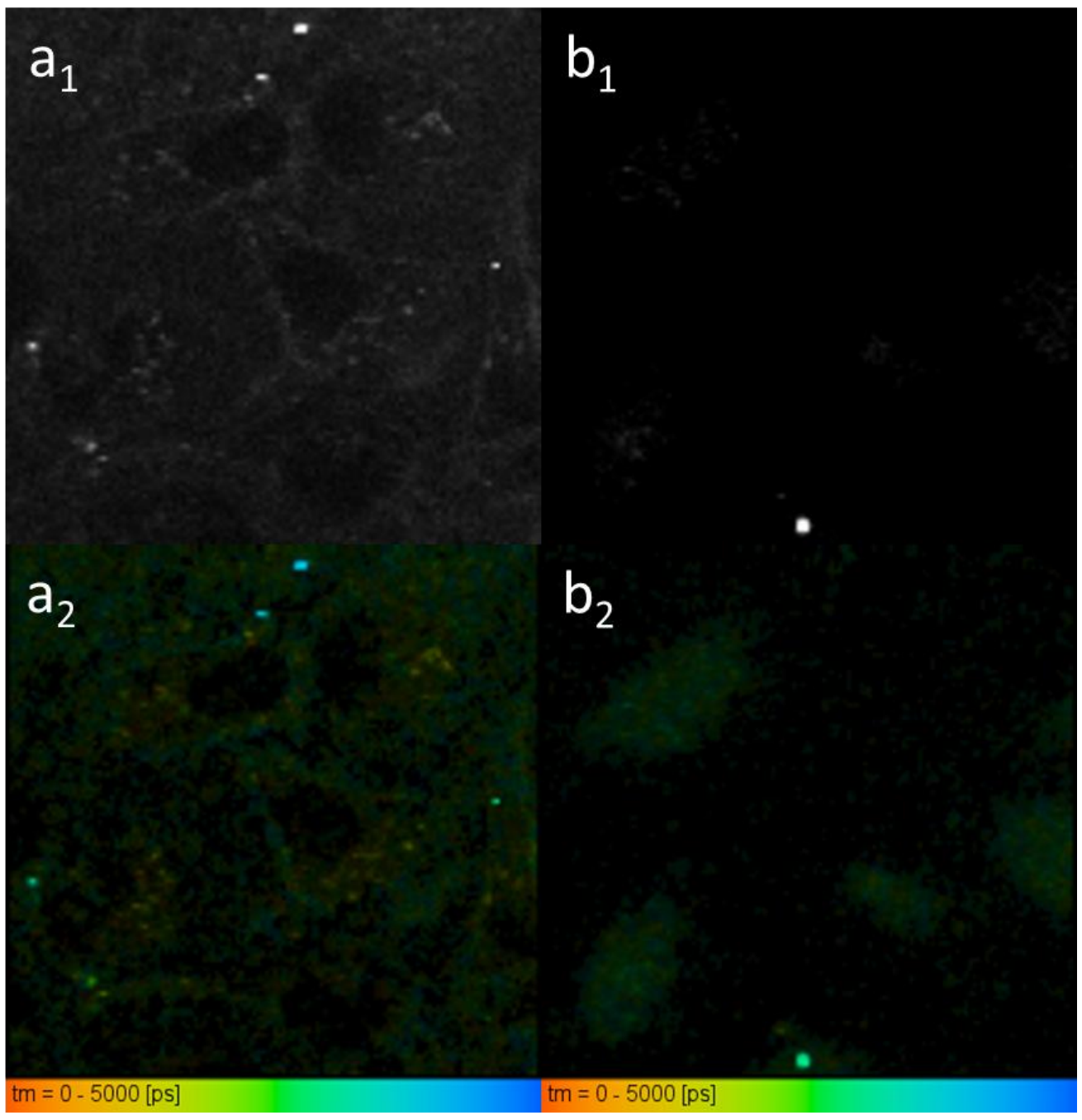

Figure S7.3. FLIM images of cells treated with 100 μM of compounds in 1% DMSO, cells were incubated at 37 ºC or 4 ºC, where (a) **1**@glucan, 4 ºC (b) **2**@glucan, 4 ºC, λex = 910 nm. a_1-b_1 is the two-photon fluorescence intensity diagram of compounds, a_2-b_2 is the fluorescence lifetime map of τ_m of compounds in HeLa cells. Scale bar: τ_m=0-5000 ps.

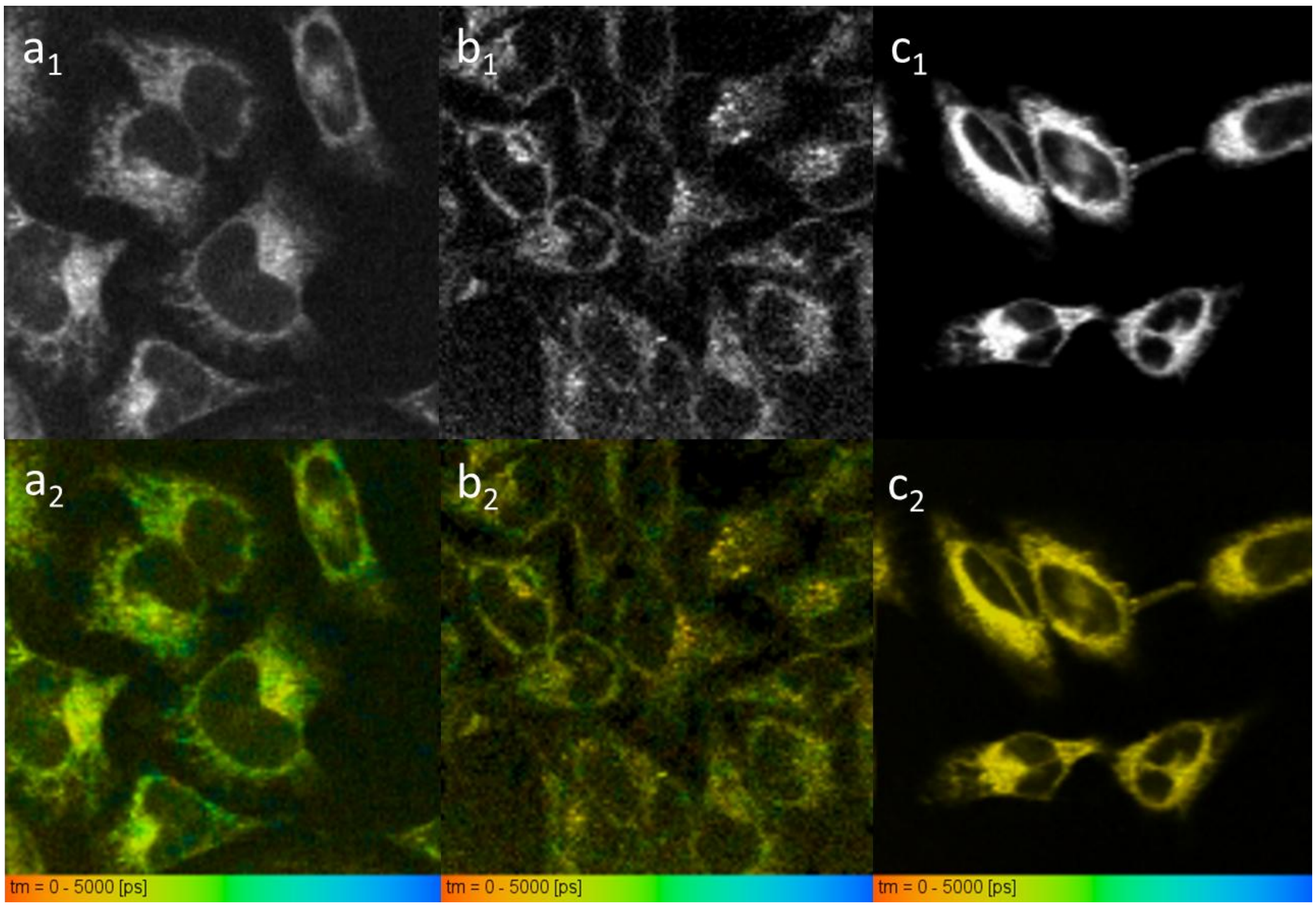

Figure S7.4. FLIM images of cells treated with 100 μM of compounds in 0.5% DMSO, cells were incubated at 37 ºC or 4 ºC, where (a) 3, 37 ºC (b) **3**, 4 ºC (c) **3**@glucan, 37 ºC, λex = 910 nm. a_1-c_1 is the two-photon fluorescence intensity diagram of compounds,

a_2-c_2 is the fluorescence lifetime map of τ_m of compounds in HeLa cells. Scale bar: τ_m=0-5000 ps.

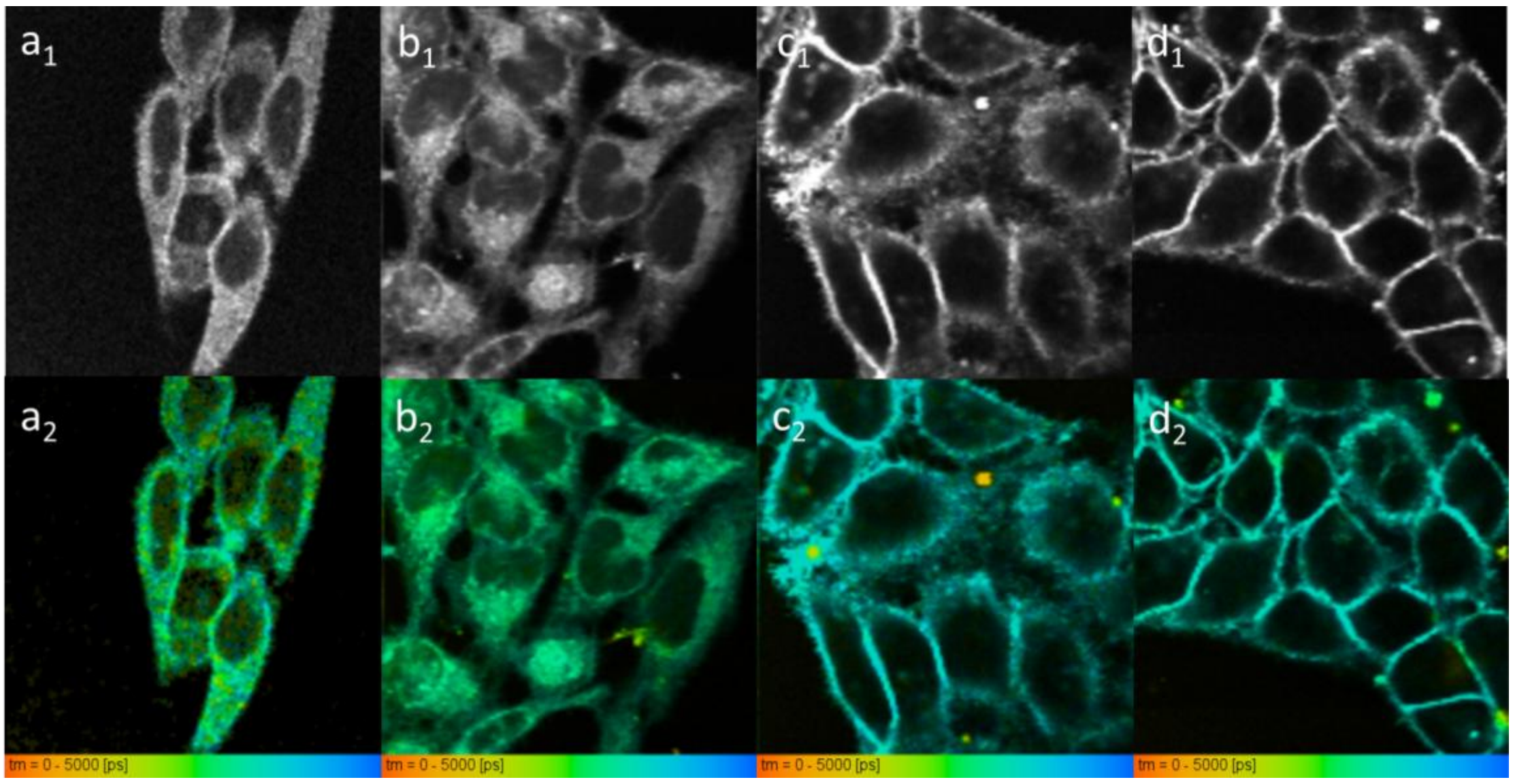

(A)

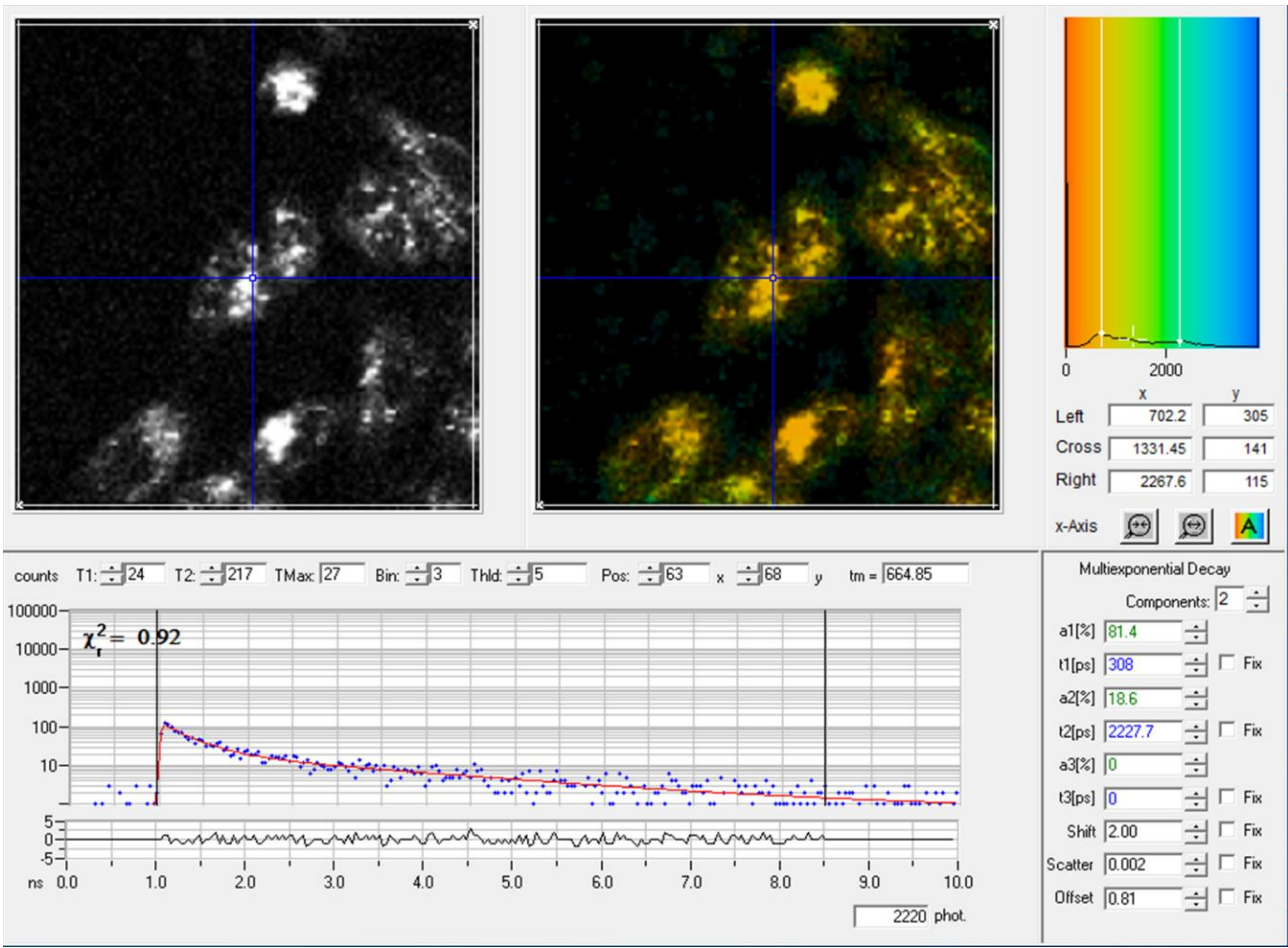

(B)

Figure S7.5. Panel A: 2P FLIM images of cells treated with 100 μM compounds in 1% or 2% DMSO, cells were incubated at 37 ºC or 4 ºC, where: (a) **5**, 1% DMSO, 37 ºC, 15 minutes (b) **5**, 1% DMSO, 37 ºC, 60 minutes (c) **6**, 1% DMSO, 4 ºC, 15 minutes

(d) **6**, 1% DMSO, 4 ºC, 60 minutes, λex = 910nm. a_1-d_1 is the two-photon fluorescence intensity diagram of compounds, a_2-d_2 is the fluorescence lifetime map of τ_m of compounds in HeLa cells. Scale bar: τ_m=0-5000 ps; **Panel B:** Overview of 2P FLIM experiment of compound **4** in HeLa, 37 ºC, 1 h incubation, 910 nm, 8.7 mW laser power (clockwise: intensity image, lifetime distribution map and parameters including TCSPC in a random spot in cytoplasm; Scale bar: τ_m=0-5000 ps.

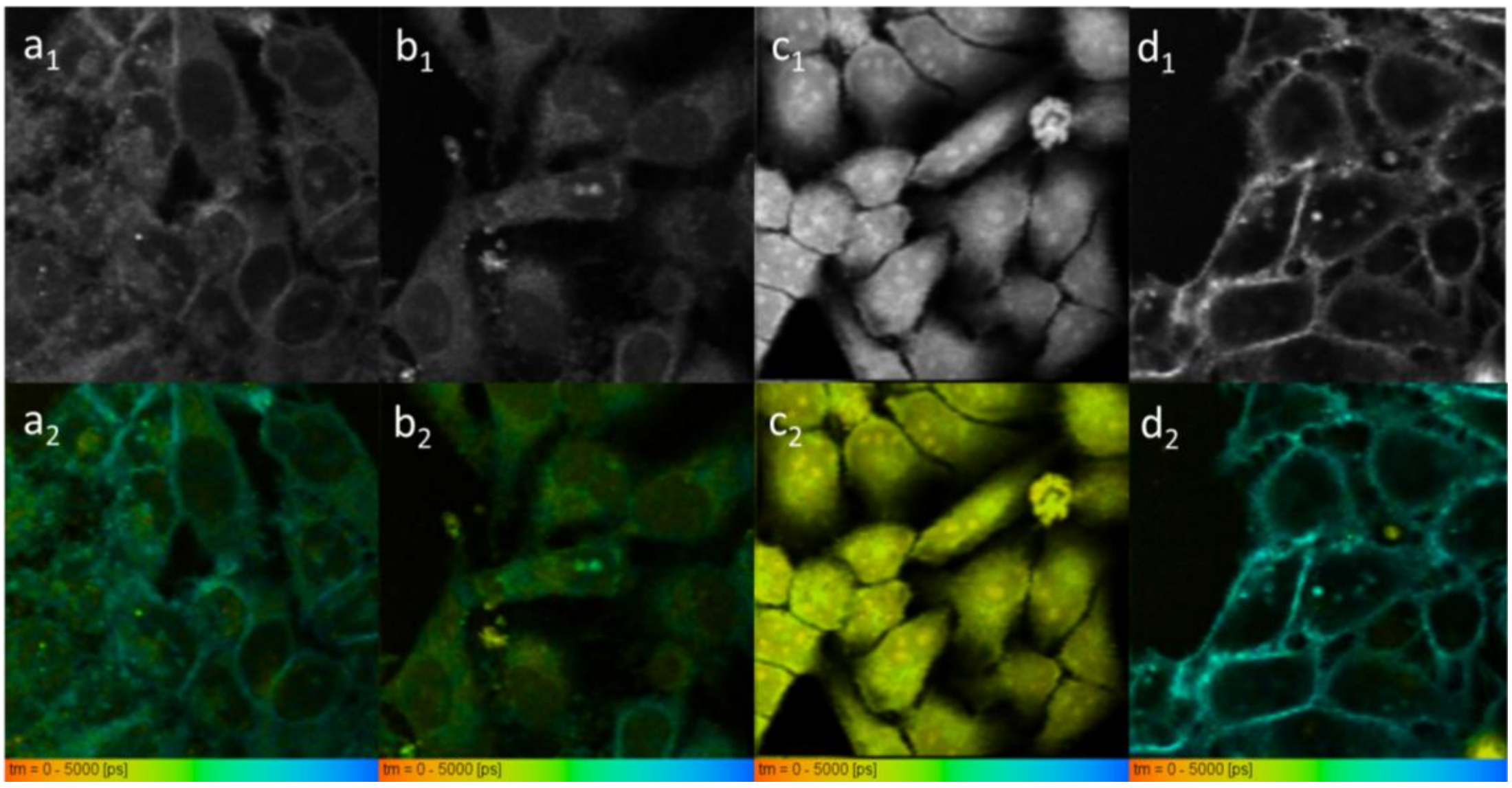

Figure S7.6. FLIM images of cells treated with 100 µM compounds in 1% or 2% DMSO, cells were incubated at 37 ºC or 4 ºC, where (a) **5**@glucan, 2% DMSO, 37 ºC, 15 minutes (b) **5**@glucan, 2% DMSO, 37 ºC, 60 minutes (c) fixed cells: **5**@glucan, 2% DMSO, 37 ºC, 15 minutes (d) **5**@glucan, 2% DMSO, 4 ºC, 60 minutes, λex = 910nm. a_1-d_1 is the two-photon fluorescence intensity diagram of compounds, a_2-d_2 is the fluorescence lifetime map of τ_m of compounds in HeLa cells. Scale bar: τ_m=0-5000 ps.

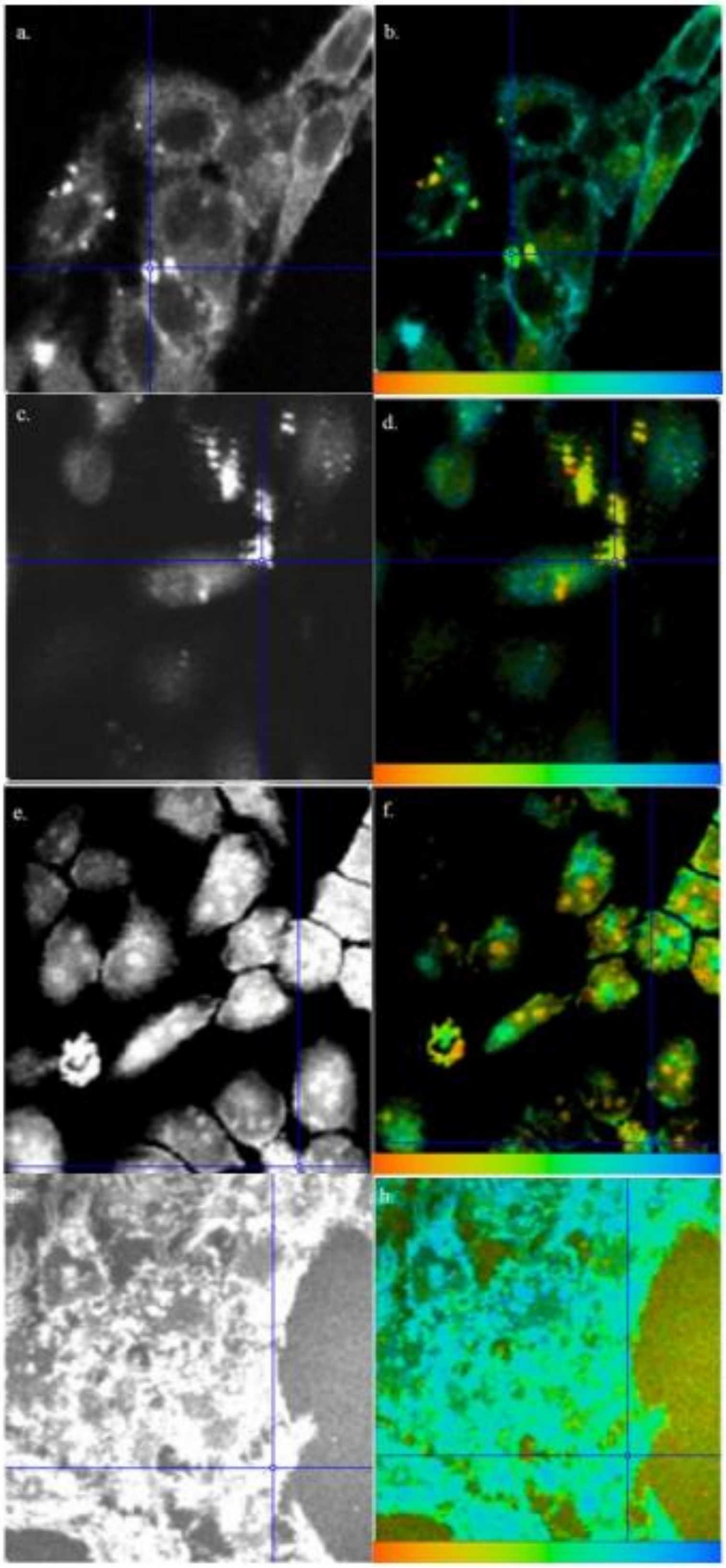

Figure S7.7. 2P-FLIM images of HeLa cells treated with 100 μM of compound **6** formulated with 1 mg/mL beta-D-glucan in 1% DMSO. Cells were incubated at 37 ºC or 4 ºC for 15 minutes or 60 min where (a) two-photon fluorescence intensity diagram of compound **6** @glucan in HeLa cells (37 ºC, 15 minutes incubation), (b) the

fluorescence lifetime map of τ_m of **6@glucan** in HeLa cells (37 ºC, 15 minutes incubation) (c) two photon fluorescence intensity diagram of **6@glucan** in HeLa cells (37 ºC, 60 minutes incubation), (d) the fluorescence lifetime map of τ_m of **6@glucan** in HeLa cells (37 ºC, 60 minutes incubation), (e) two photon fluorescence intensity diagram of compound 6@glucan in HeLa cells (4 ºC, 15 minutes incubation, fixed cells) and (f) the fluorescence lifetime map of τ_m of compound **6@glucan** in HeLa cells (4 ºC, 15 minutes incubation), (g) two photon fluorescence intensity diagram of **6 @glucan** in HeLa cells (4 ºC, 60 minutes incubation) and (h) the fluorescence lifetime map of τ_m of **6 @glucan** in HeLa cells (4 ºC, 60 minutes incubation), τ_m=0-5000 ps.

Table S1. Two-photon TCSPC and FLIM parameters in HeLa cells treated with 10 µM of compound **6** formulated in 1 mg/mL beta-D-glucan (denoted **6@glucan**) in 1% DMSO. Cells were incubated at 37 ºC or 4 ºC for 15 minutes or 60 min (λ = 910 nm laser power 2.5 mW).

Temperature (ºC)	37	37	4	4
Incubation time (min)	15	60	15	60
χ^2	1.03	1.01	1.05	0.99
τ_1 (ns)	1.0	0.5	0.7	0.7
τ_1 %	50	59.8	56.2	34.5
FWHM (ns)	1.0	0.6	1.7	1.4
τ_2 (ns)	3.8	3.1	3.0	3.4
τ_2 %	50	40.2	43.8	65.5
FWHM (ns)	1.8	1.6	0.3	1.3

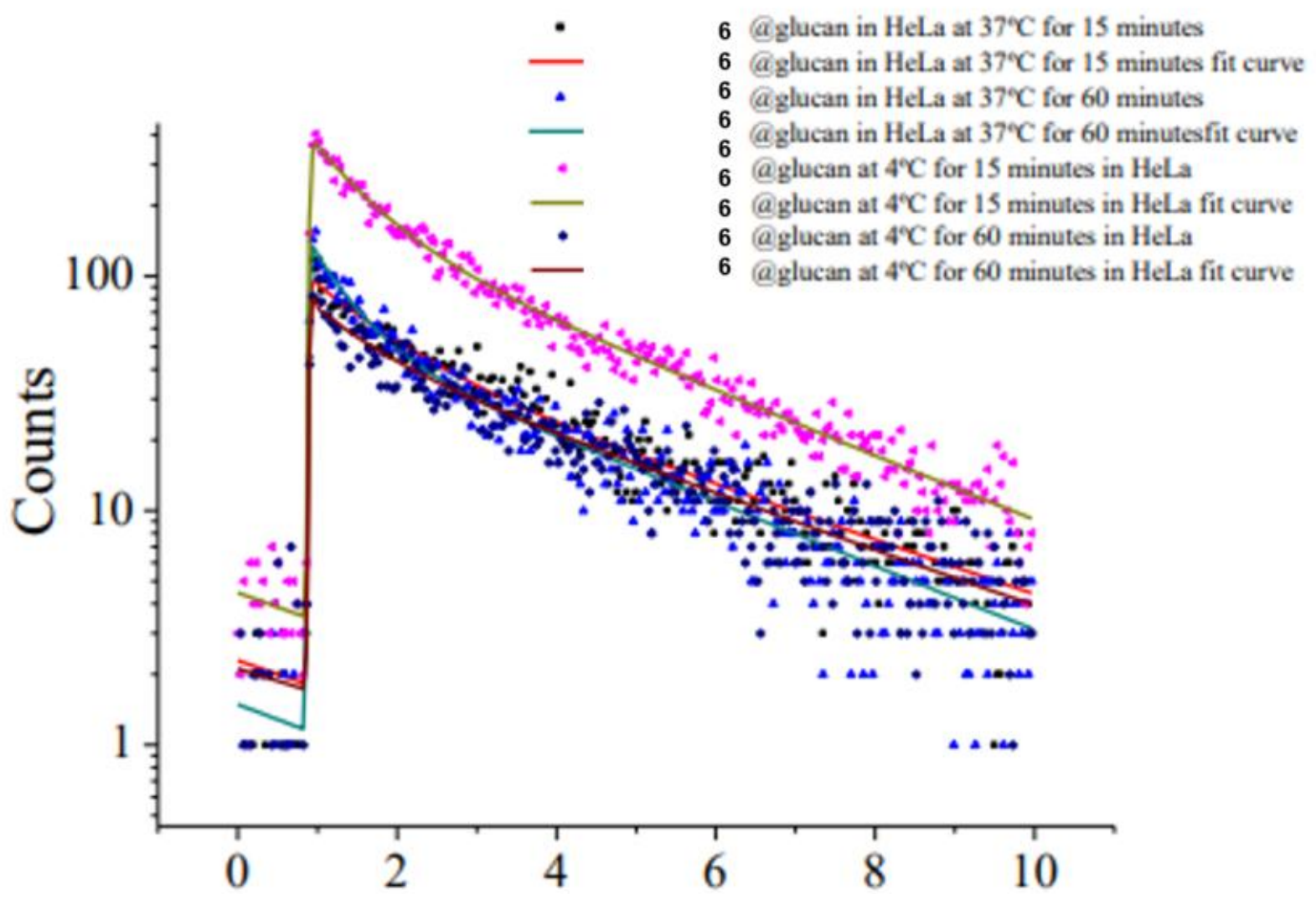

Figure S7.8. FLIM decay curves for compound **6** (100 µM) and **6@glucan** in HeLa recorded under different incubation conditions. λex = 910 nm, laser power 2.5 mW.

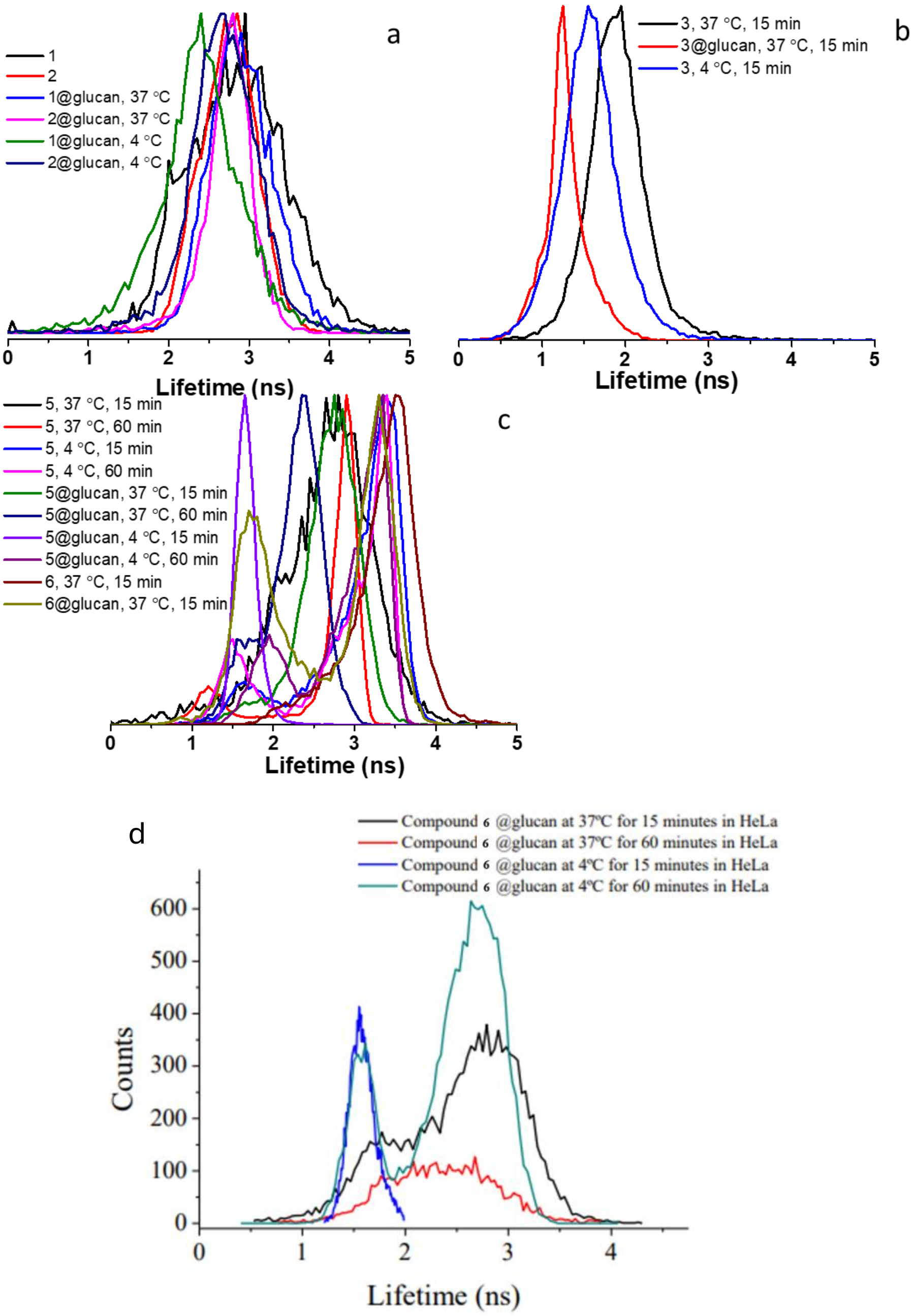

Figure S7.9. Overview of the normalised MP FLIM fluorescence lifetime distributions in HeLa cells under a range of conditions: a) compounds **1, 2**, 100 µM and their corresponding formulations as glucan hybrids under either 37 °C and 4 °C incubation, b) compound **3** and **3@glucan**, c) compound **5** and **5@glucan** under 37 °C and 4 °C compared with the analogous **6** and **6@glucan** at 37 °C, 15 min incubation, λex = 910 nm, d) Fluorescence lifetime distribution curves of HeLa cells treated with compound 6 @glucan under different conditions. λex = 910 nm, laser power 2.5 mW.

Additional 2P FLIM and fitted data at 15 min and 1 h incubation times:

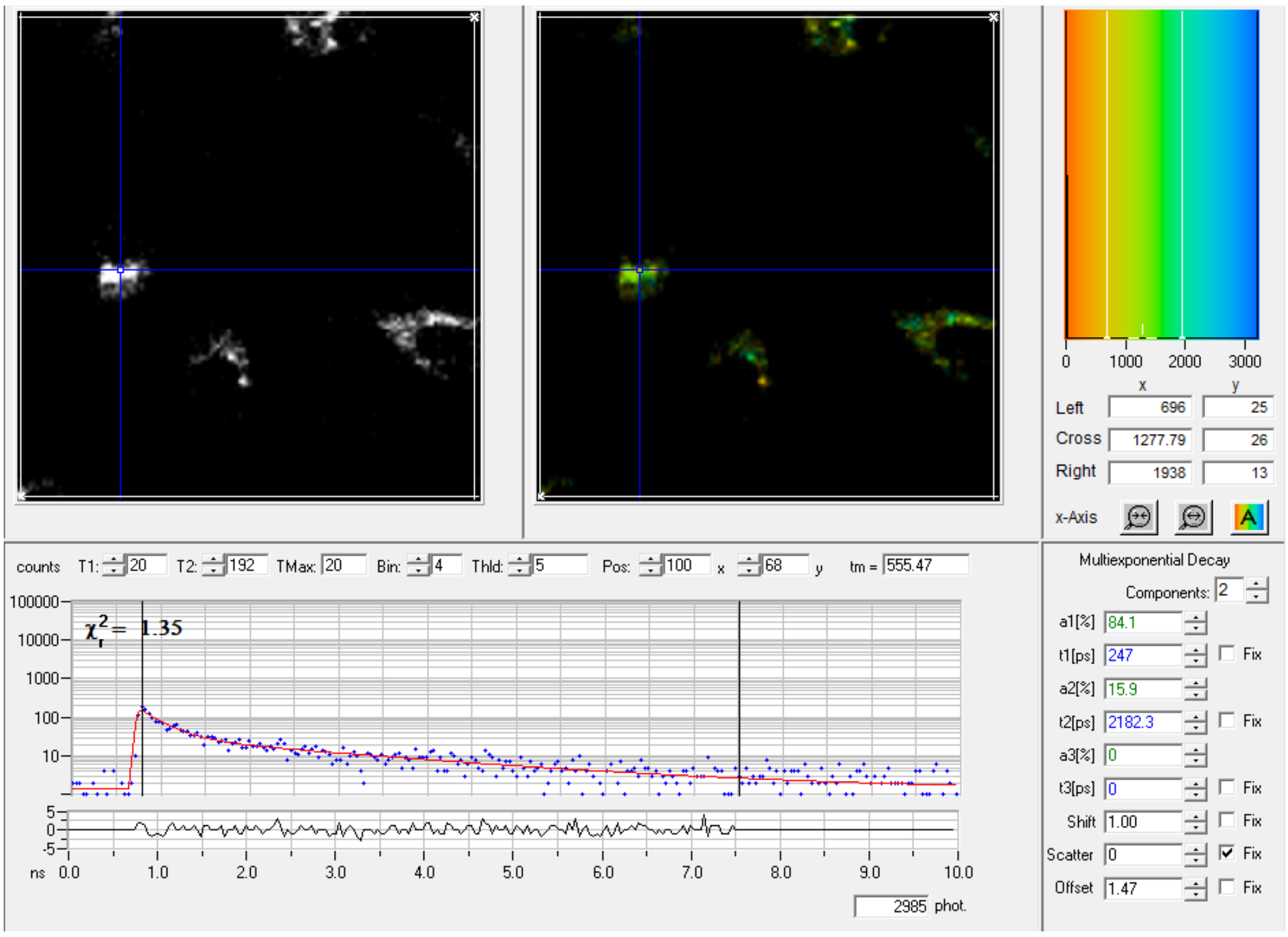

Figure S7.10. 2P FLIM control experiment of 2% DMSO in serum-free medium, PC-3, 15 min incubation at 37 ºC, 910 nm, (clockwise: intensity image, lifetime distribution map and parameters including TCSPC in a random spot in cytoplasm 100 µm field of view, Lifetime scale bar: τ_m=0-3000 ps

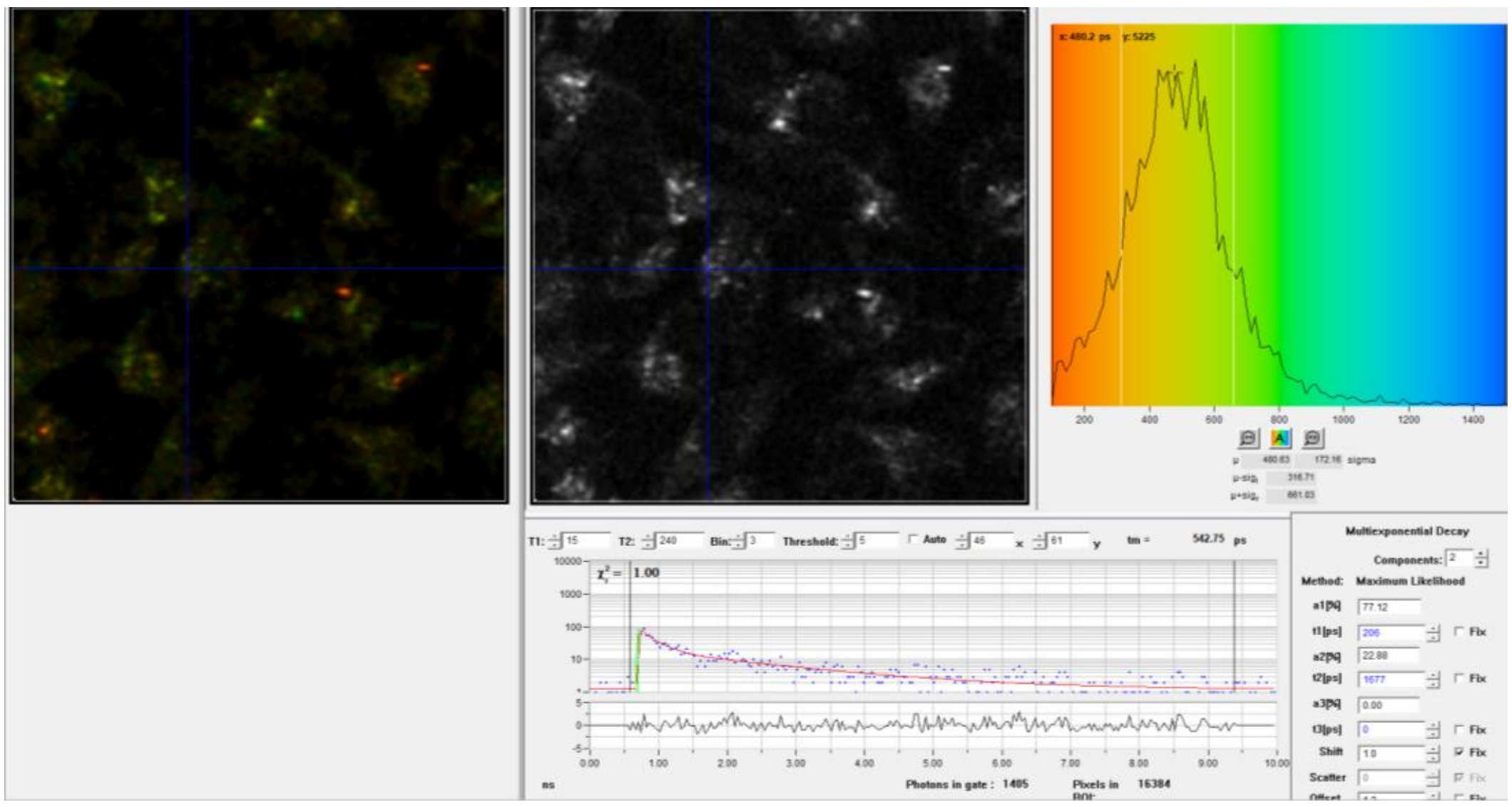

Figure S7. 11. 2P FLIM control experiment of 1% DMSO in HeLa, 15 min incubation at 37 ºC, 910 nm, (clockwise: intensity image, lifetime distribution map and parameters including TCSPC in a random spot in cytoplasm). 100 µm field of view, Lifetime scale bar: τ_m=0-1500 ps

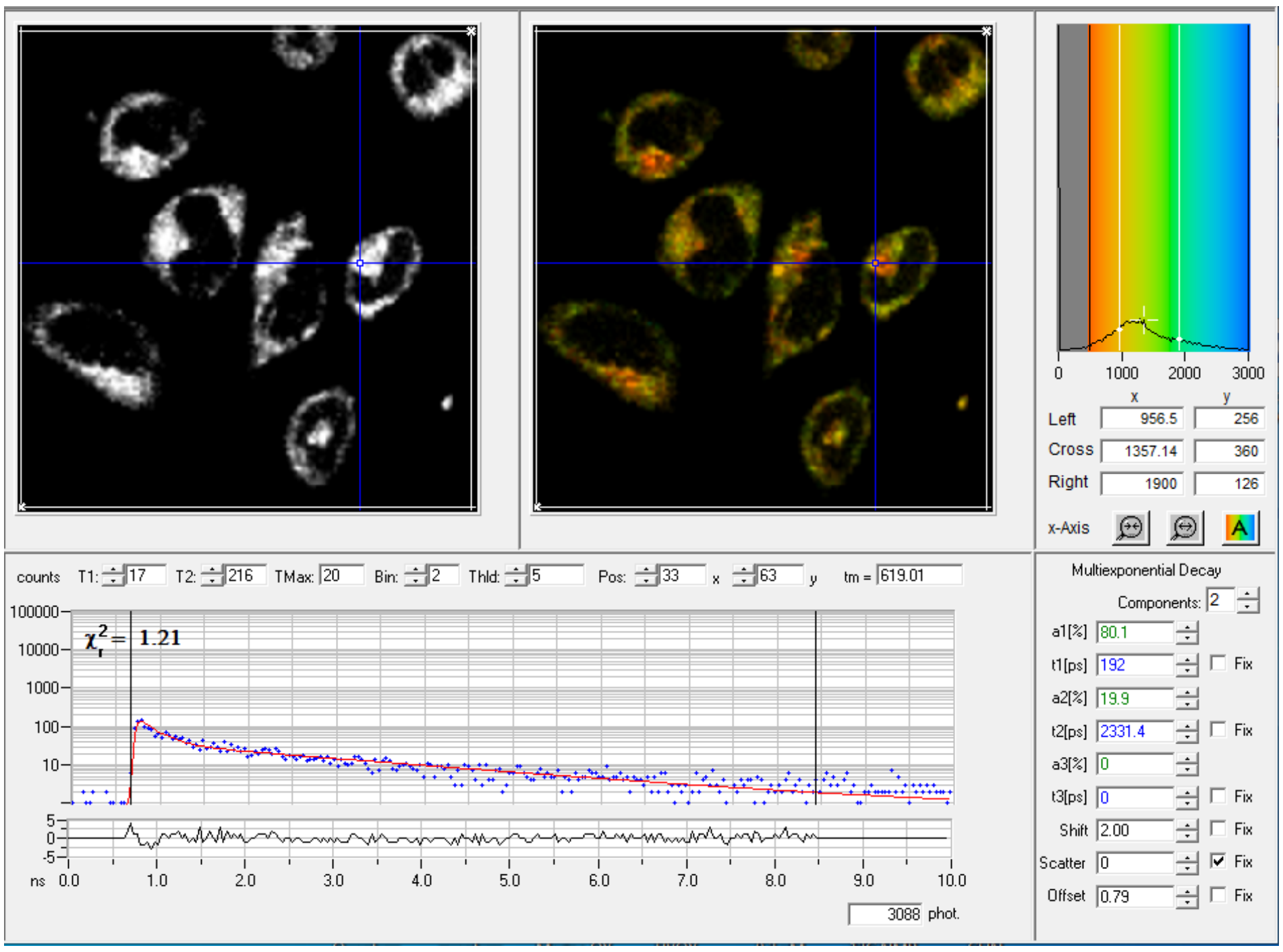

Figure S7.12. 2P FLIM control experiment of 2% DMSO in PC-3, 15 min incubation at 37 °C, 810 nm, (clockwise: intensity image, lifetime distribution map and parameters including TCSPC in a random spot in cytoplasm). 100 µm field of view, Lifetime scale bar: τ_m=0-3000 ps.

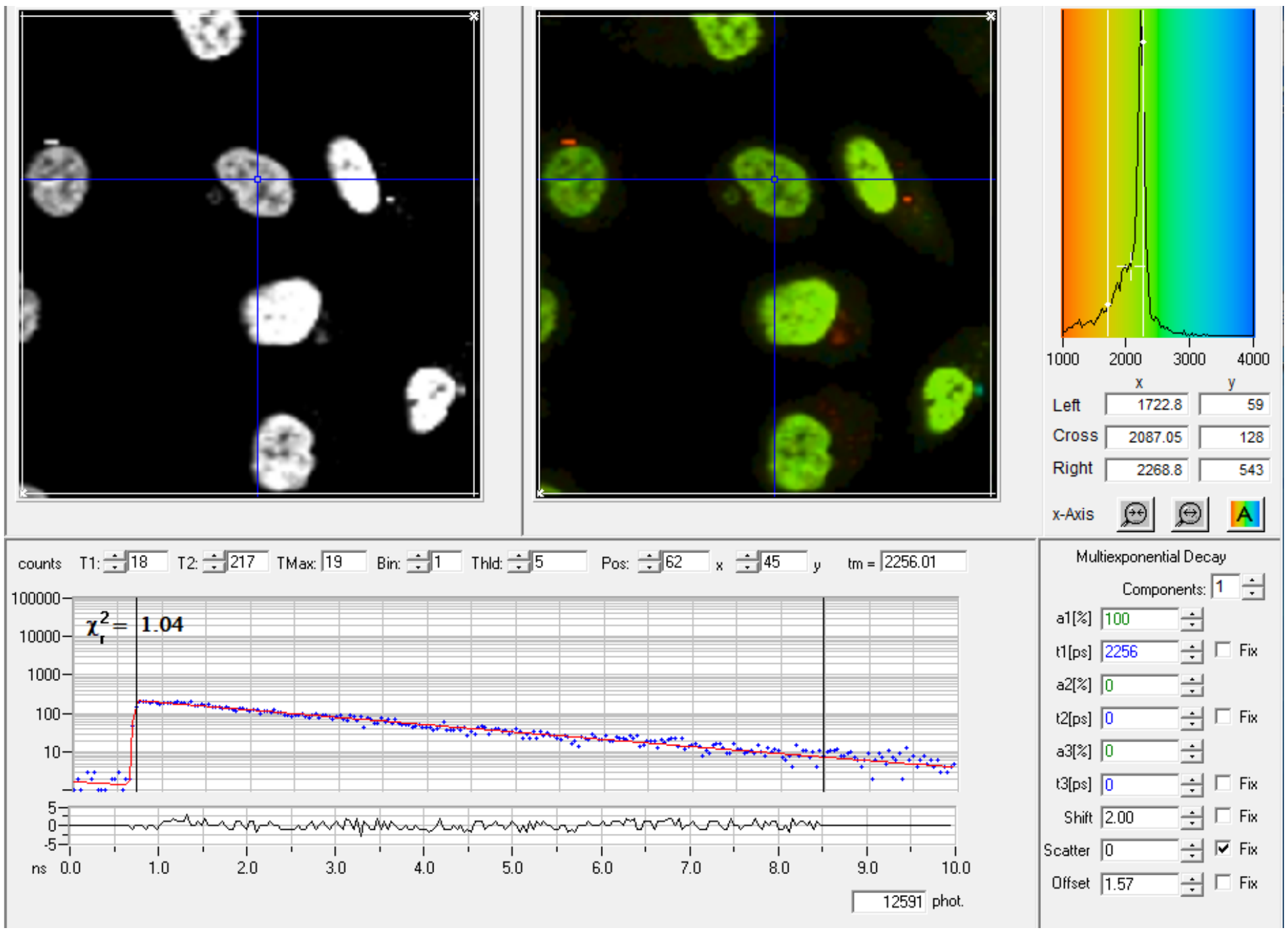

Figure S7. 13. 2P FLIM control experiment of Hoechst dye in PC-3, 15 min incubation at 37 °C, 810 nm, (clockwise: intensity image, lifetime distribution map and parameters including TCSPC in a random spot in cytoplasm). 100 µm field of view, Lifetime scale bar: τ_m=0-4000 ps

Compound 1 - 2P FLIM

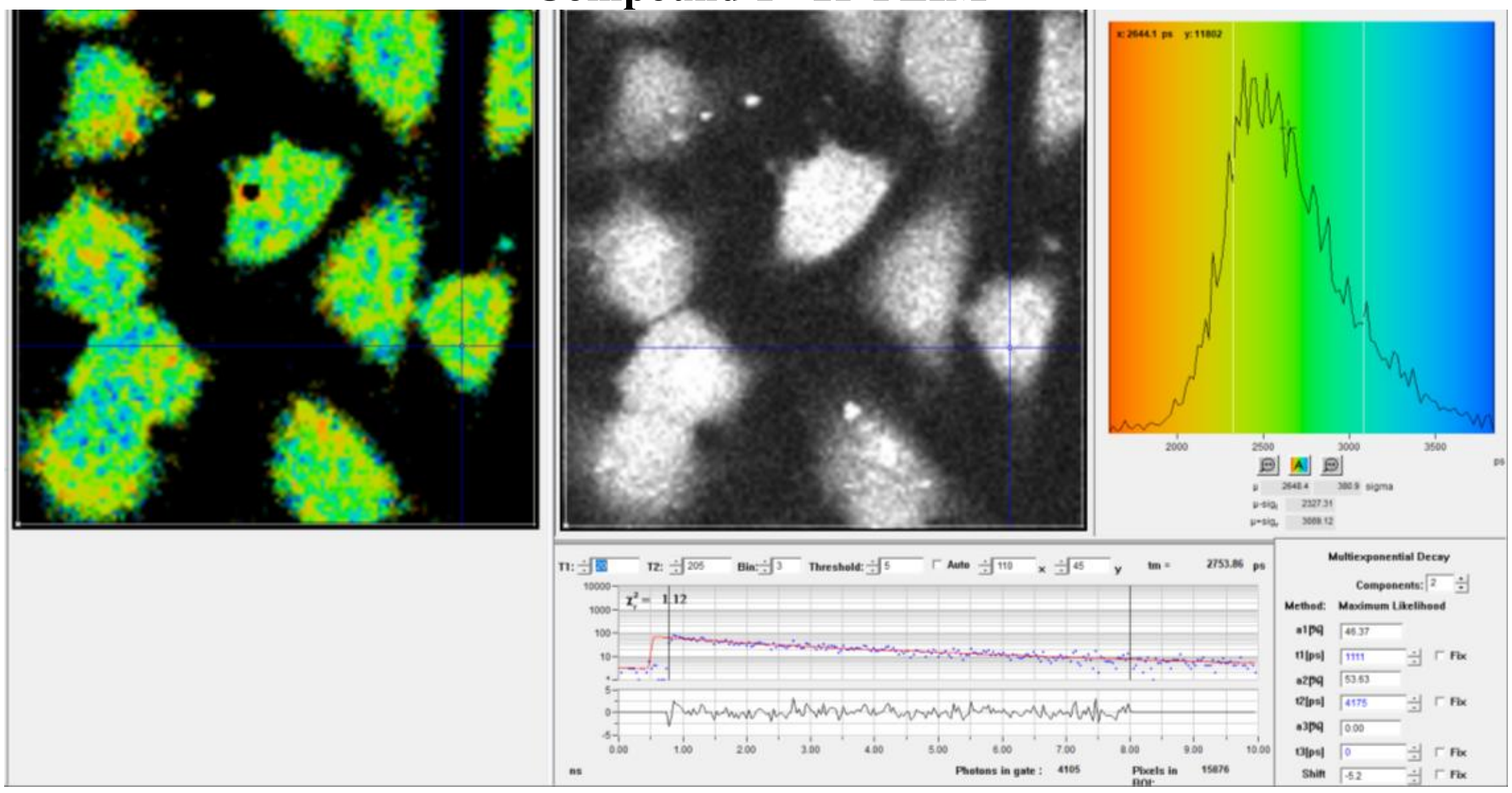

Figure S7.14. 2P FLIM of compound **1**, 100 µM in 1% DMSO in HeLa, 15 min incubation at 37 ºC, 910 nm, (clockwise: lifetime distribution map, intensity image, and FLIM parameters including TCSPC in a random spot in cytoplasm). Field of view: 100 µm, Lifetime scale bar: τ_m=1500-4000 ps.

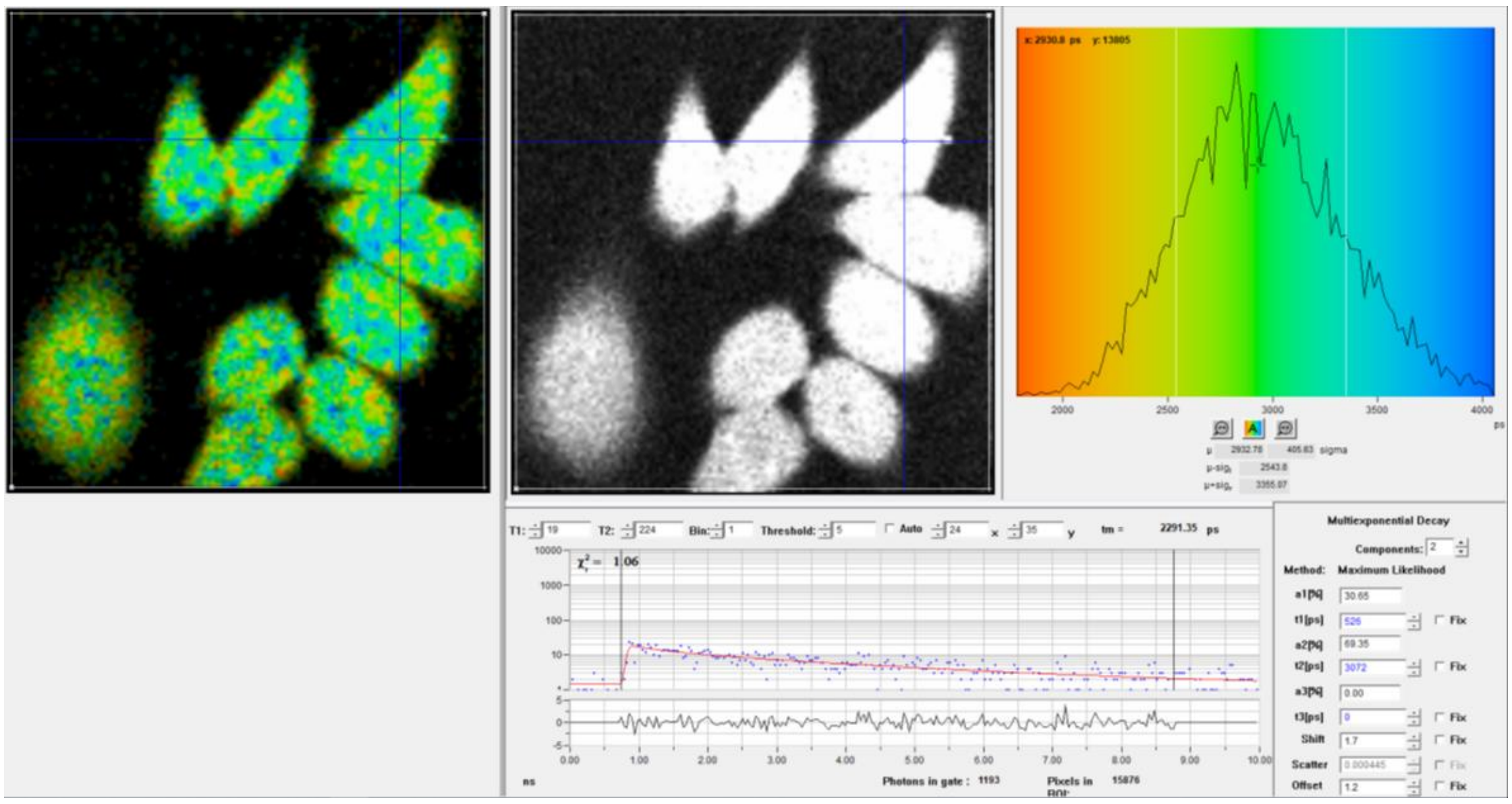

Figure S7.15. 2P FLIM of the hybrid **1@glucan** in 100 µM of **1** anchored in 1 mg/mL beta-D-glucan in serum-free medium containing 1% DMSO in HeLa, 15 min incubation at 37 ºC, 910 nm (clockwise: lifetime distribution map, intensity image, and FLIM parameters including TCSPC in a random spot in cytoplasm). Field of view: 100 µm, Lifetime scale bar: τ_m=1500-4000 ps.

Compound 2 – 2P FLIM

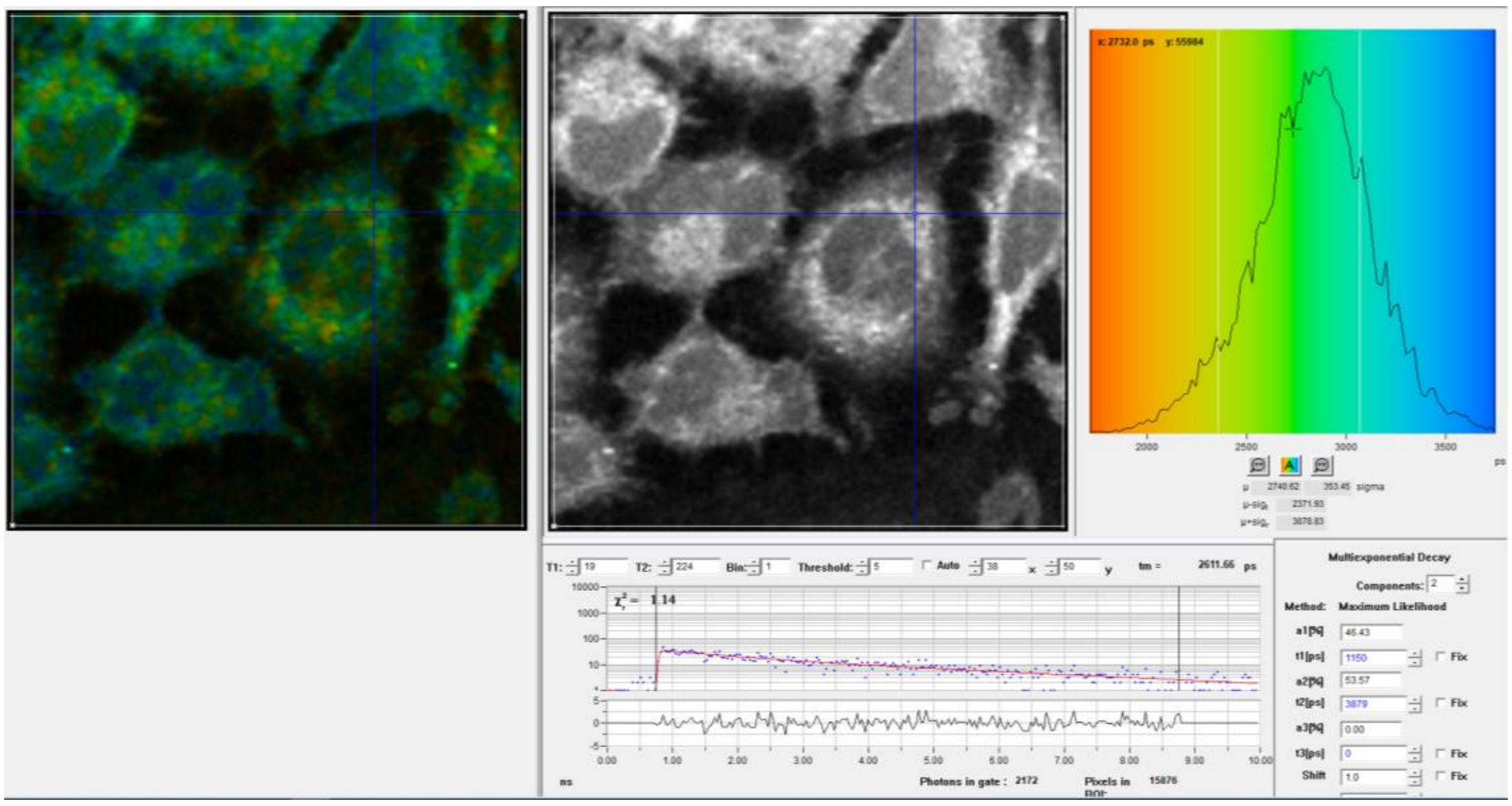

Figure S7.16. 2P FLIM of compound **2**, 100 µM in 1% DMSO in HeLa, 15 min incubation at 37 ºC, 910 nm, (clockwise: lifetime distribution map, intensity image, and FLIM parameters including TCSPC in a random spot in cytoplasm). Field of view: 100 µm, Lifetime scale bar: τ_m=1500-4000 ps.

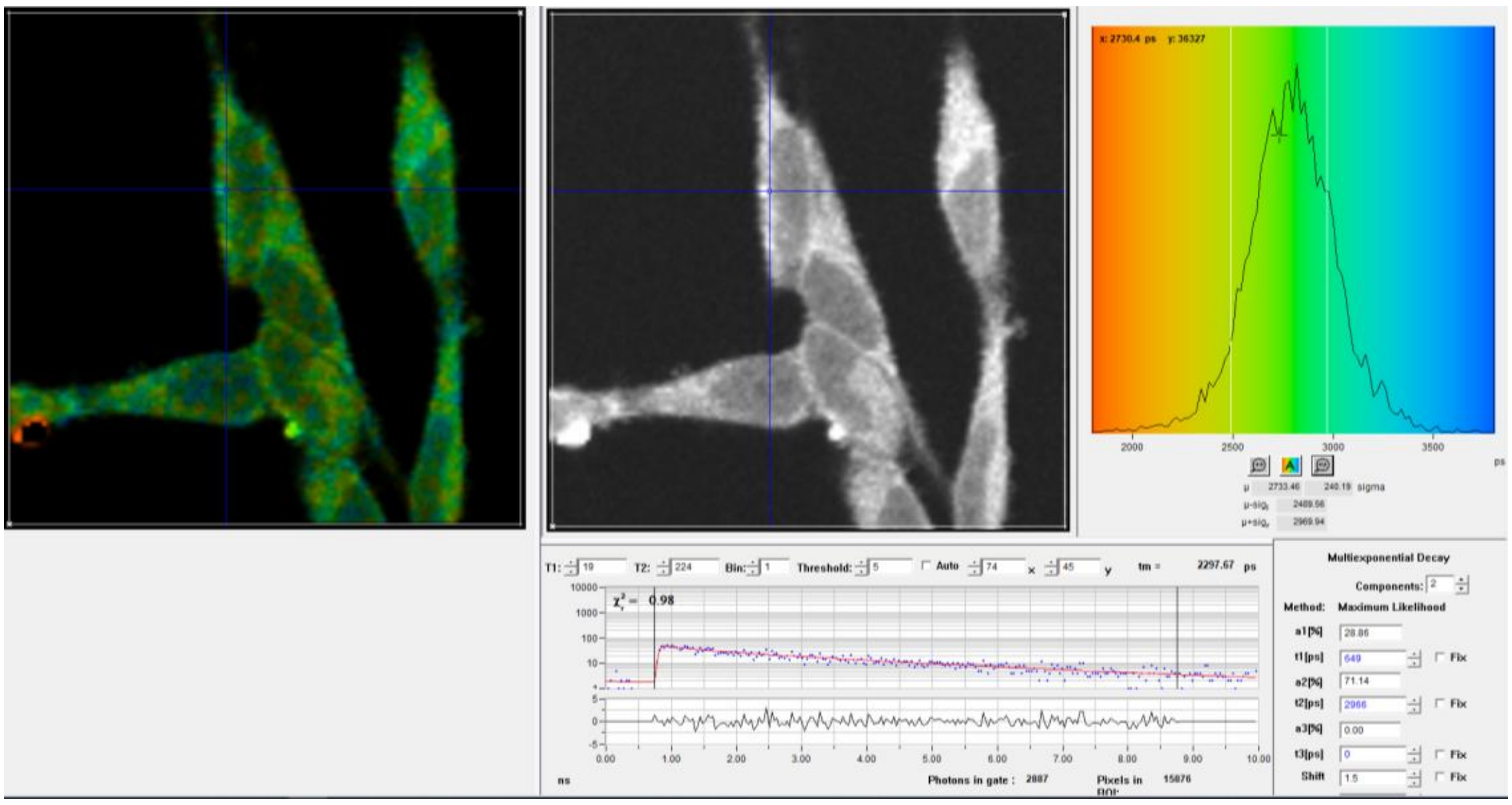

Figure S7.17. 2P FLIM of compound **2**@glucan, containing 100 µM of **2** anchored in 1 mg/mL beta-D-glucan in serum-free medium containing 1% DMSO, 15 min incubation at 37 ºC, 910 nm, (clockwise: lifetime distribution map, intensity image and FLIM parameters including TCSPC in a random spot in cytoplasm). Field of view: 100 µm, Lifetime scale bar: τ_m=1500-4000 ps.

Compound 3 FLIM

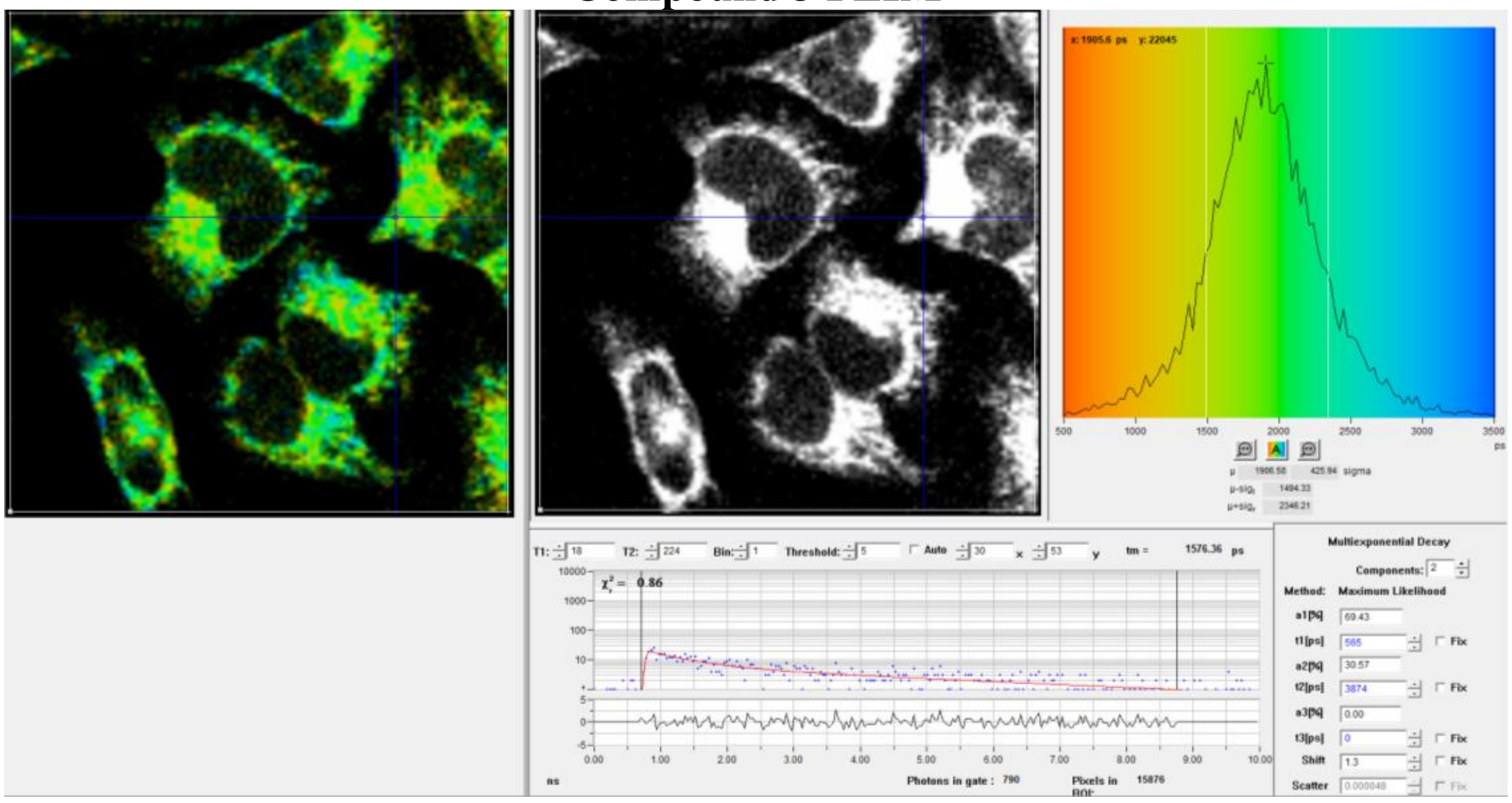

Figure S7.18. 2P FLIM of compound **3**, 100 µm and 1% DMSO in HeLa, 15 min incubation at 37 ºC, 910 nm, (clockwise: lifetime distribution map, intensity image and FLIM parameters including TCSPC in a random spot in cytoplasm). Field of view: 100 µm, Lifetime scale bar: τ_m=500-3500 ps.

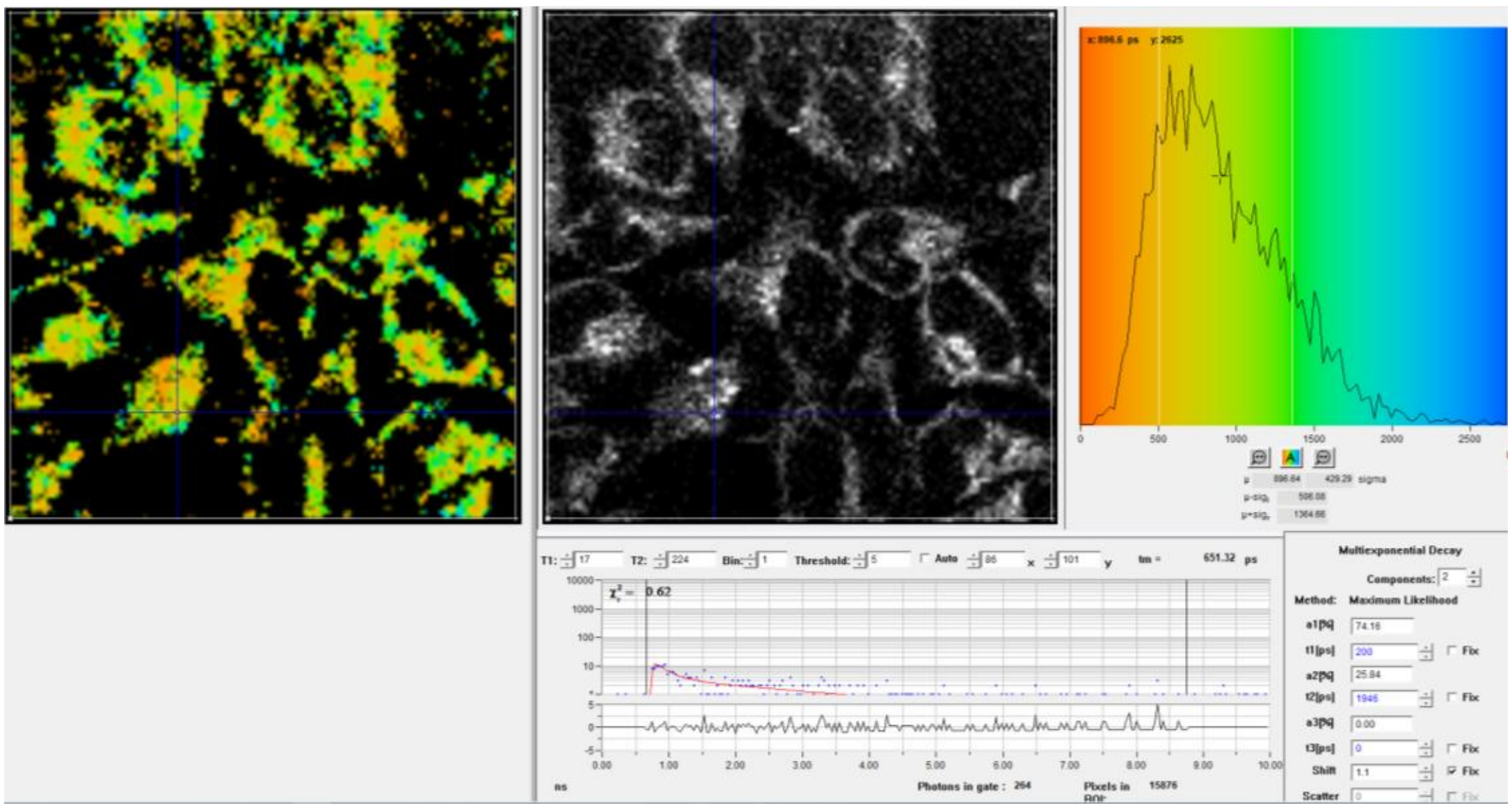

Figure S7.19. 2P FLIM of compound **3** 100 µm in 0.5% DMSO in HeLa, 15 min incubation at 4 ºC, 910 nm, (clockwise: lifetime distribution map, intensity image and FLIM parameters including TCSPC in a random spot in cytoplasm). 100 um field of view, Lifetime scale bar: τ_m=0-2750 ps.

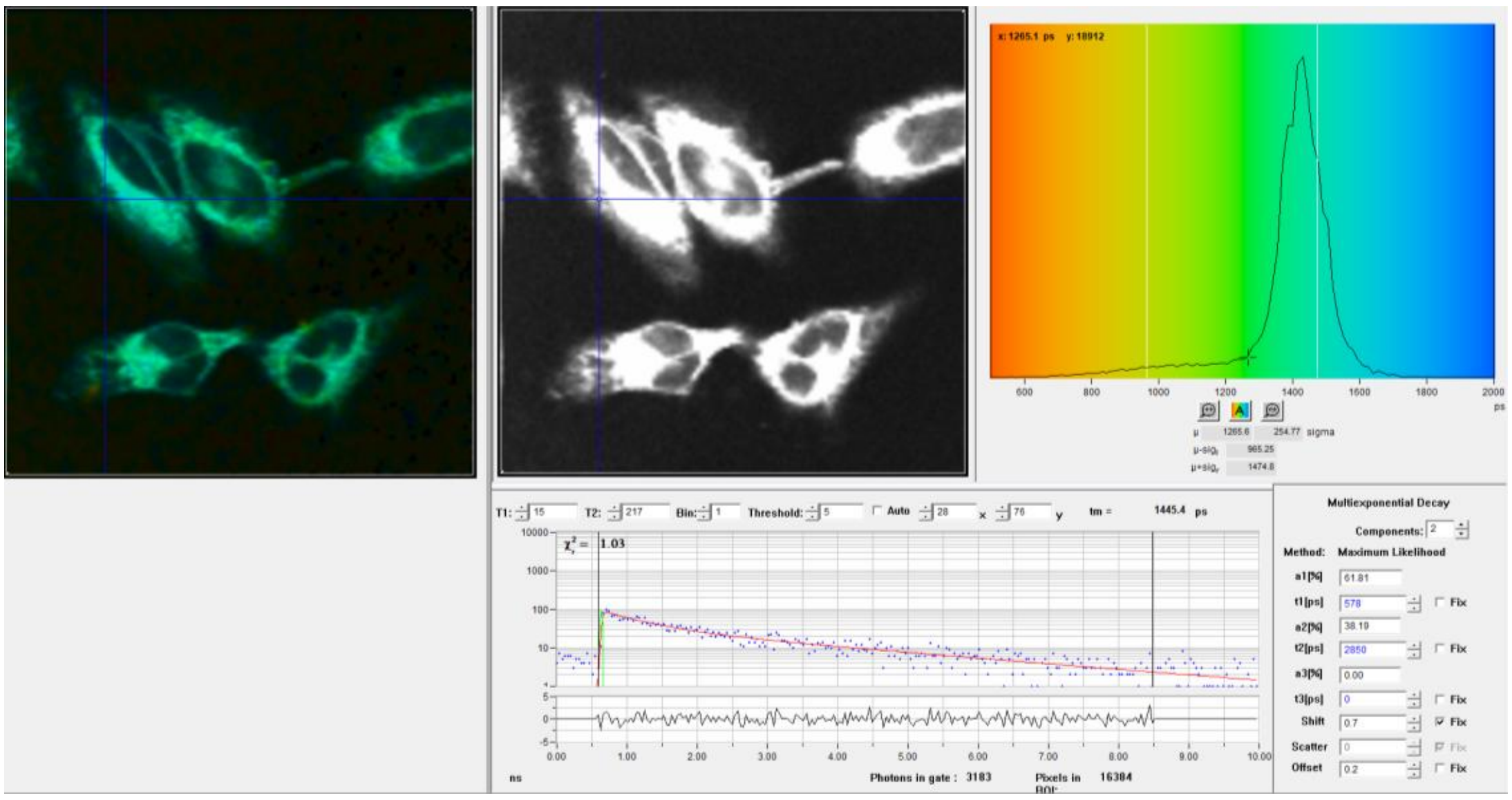

Figure S7.20. 2P FLIM of hybrid **3@glucan**, containing 100 µM of **3** anchored on 1 mg/mL beta-D-glucan in serum free medium with 2% DMSO in HeLa cells, 15 min incubation at 37 ºC, 910 nm, (clockwise: lifetime distribution, intensity image, map and parameters including TCSPC in a random spot in cytoplasm). Field of view: 100 µm, Lifetime scale bar: τ_m=400-2000 ps.

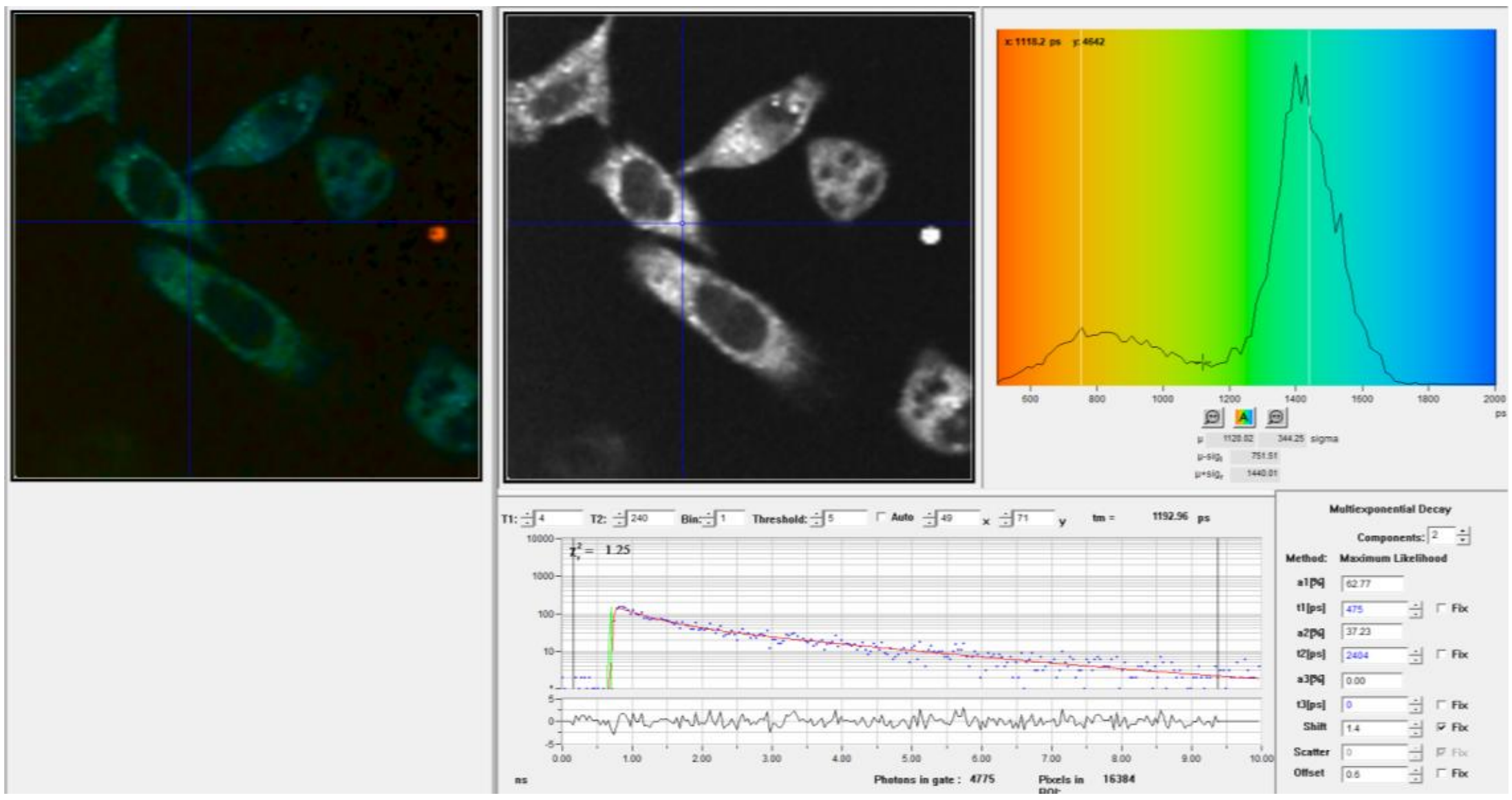

Figure S7.21. 2P FLIM of the hybrid denoted **3@glucan**, containing 100 µM of **3** anchored on 1 mg/mL beta-D-glucan in serum free medium with 2% DMSO in PC-3 cells, 15 min incubation at 37 ºC, 910 nm, (clockwise: lifetime distribution, intensity image, map and parameters including TCSPC in a random spot in cytoplasm). Field of view: 100 µm, Lifetime scale bar: τ_m=400-2000 ps.

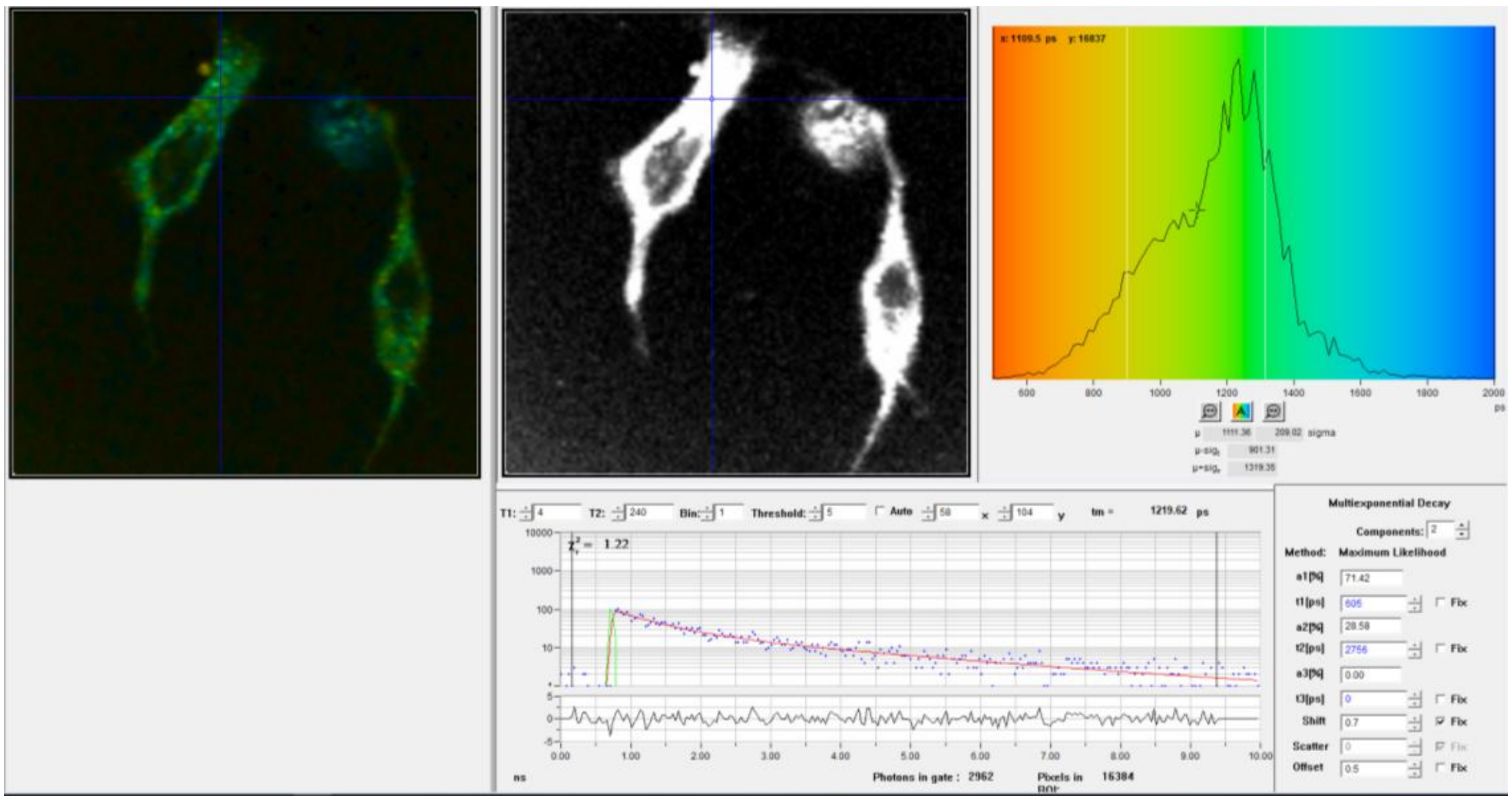

Figure S7.22. 2P FLIM of the hybrid denoted **3@glucan**, containing 100 μM of **3** anchored on 1 mg/mL beta-D-glucan in serum free medium with 2% DMSO in PC-3 cells, 15 min incubation at 37 °C, 910 nm, (clockwise: lifetime distribution, intensity image, map and parameters including TCSPC in a random spot in cytoplasm). Field of view: 100 μm, Lifetime scale bar: τ_m=400-2000 ps.

Compound 4 – 2P FLIM

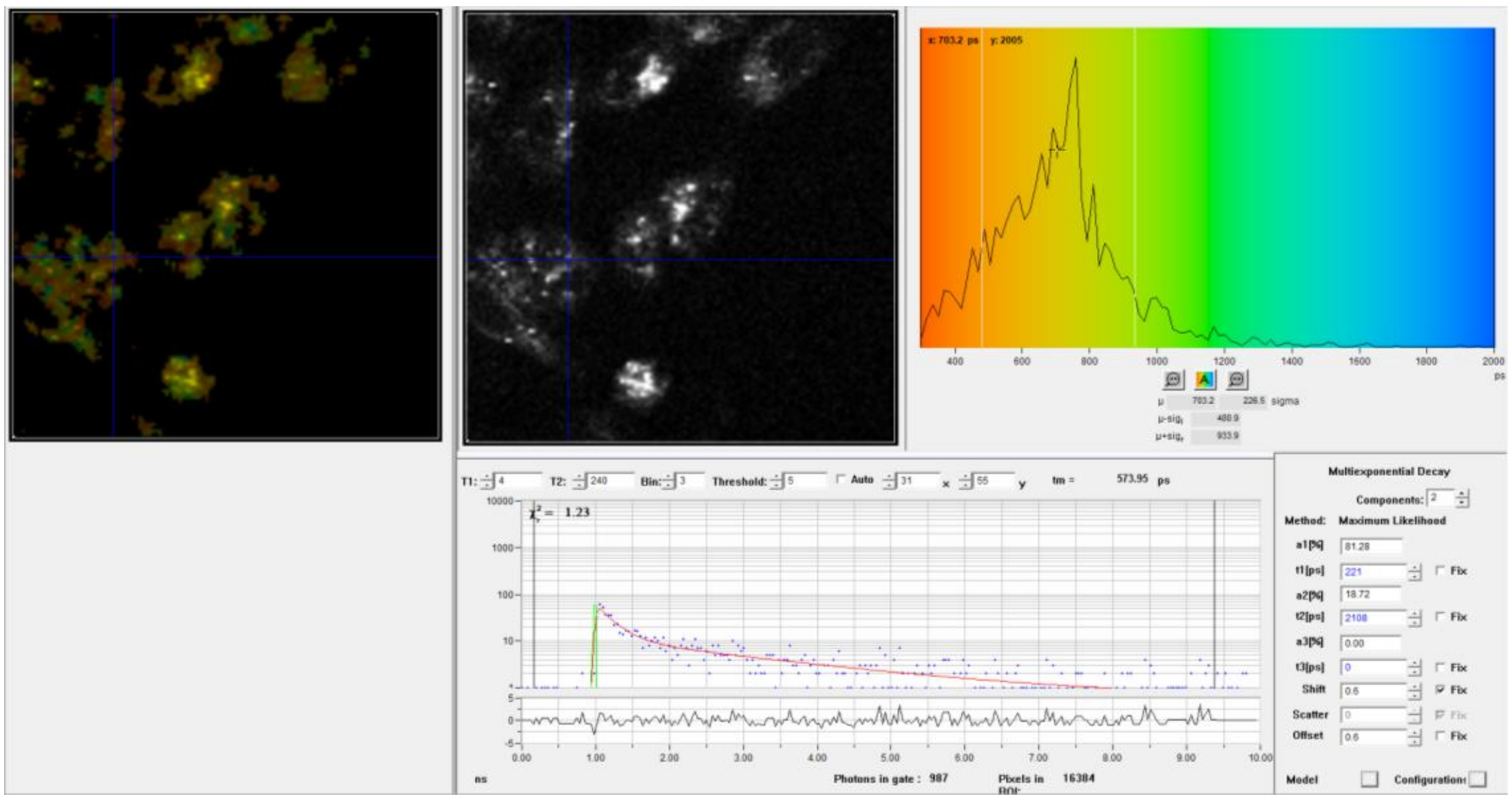

Figure S7.23. 2P FLIM of compound **4**, 100 μM in 0.5% DMSO in HeLa, 15 min incubation at 37 °C followed by washing with H_2O, 910 nm and 8.7 mW power, (clockwise: lifetime distribution map, intensity image, and parameters including TCSPC in a random spot in cytoplasm). Field of view: 100 μm, Lifetime scale bar: τ_m=0-2000 ps.

Compound 5 2P FLIM at 910 nm

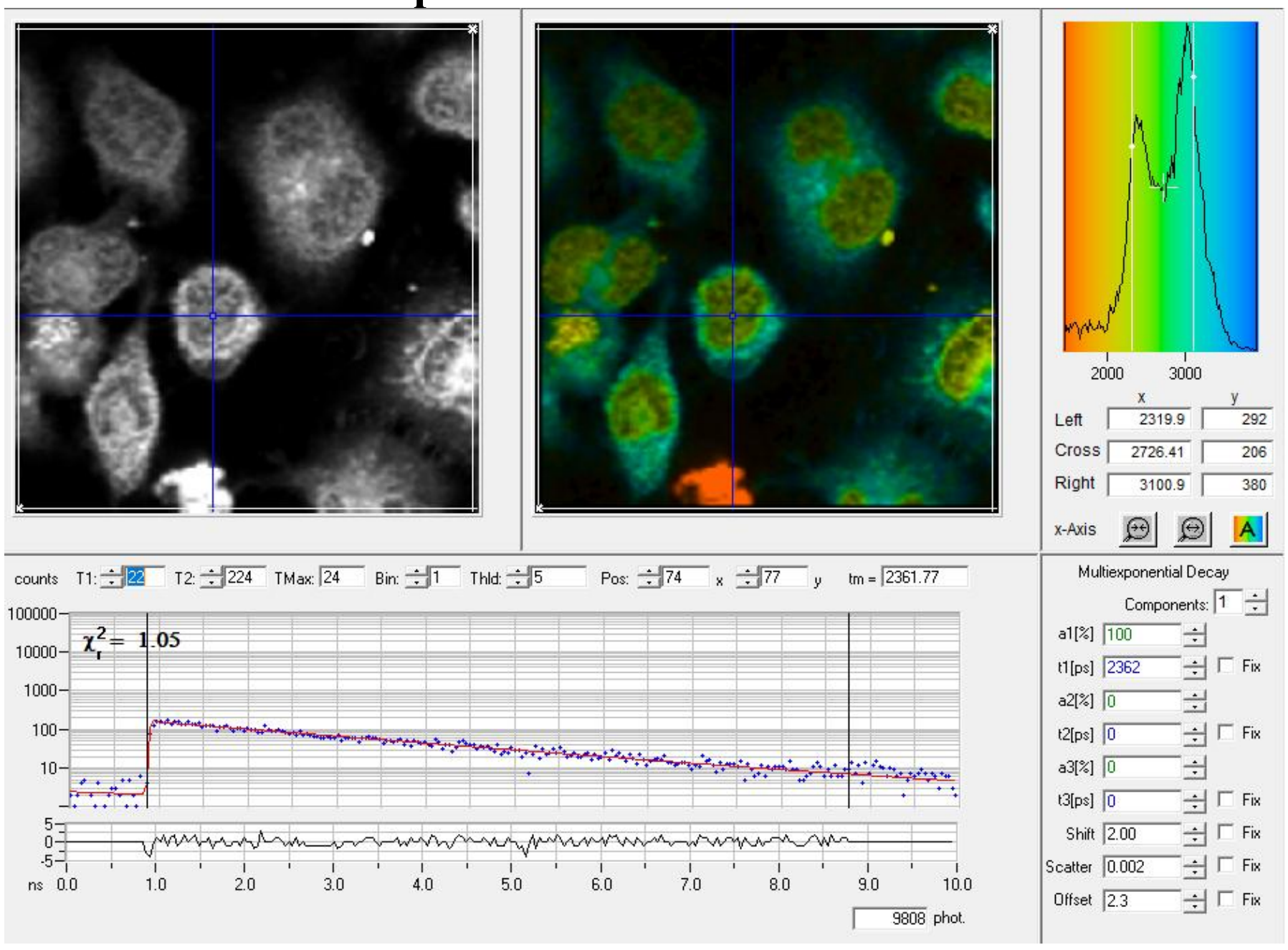

(a) Spot 1

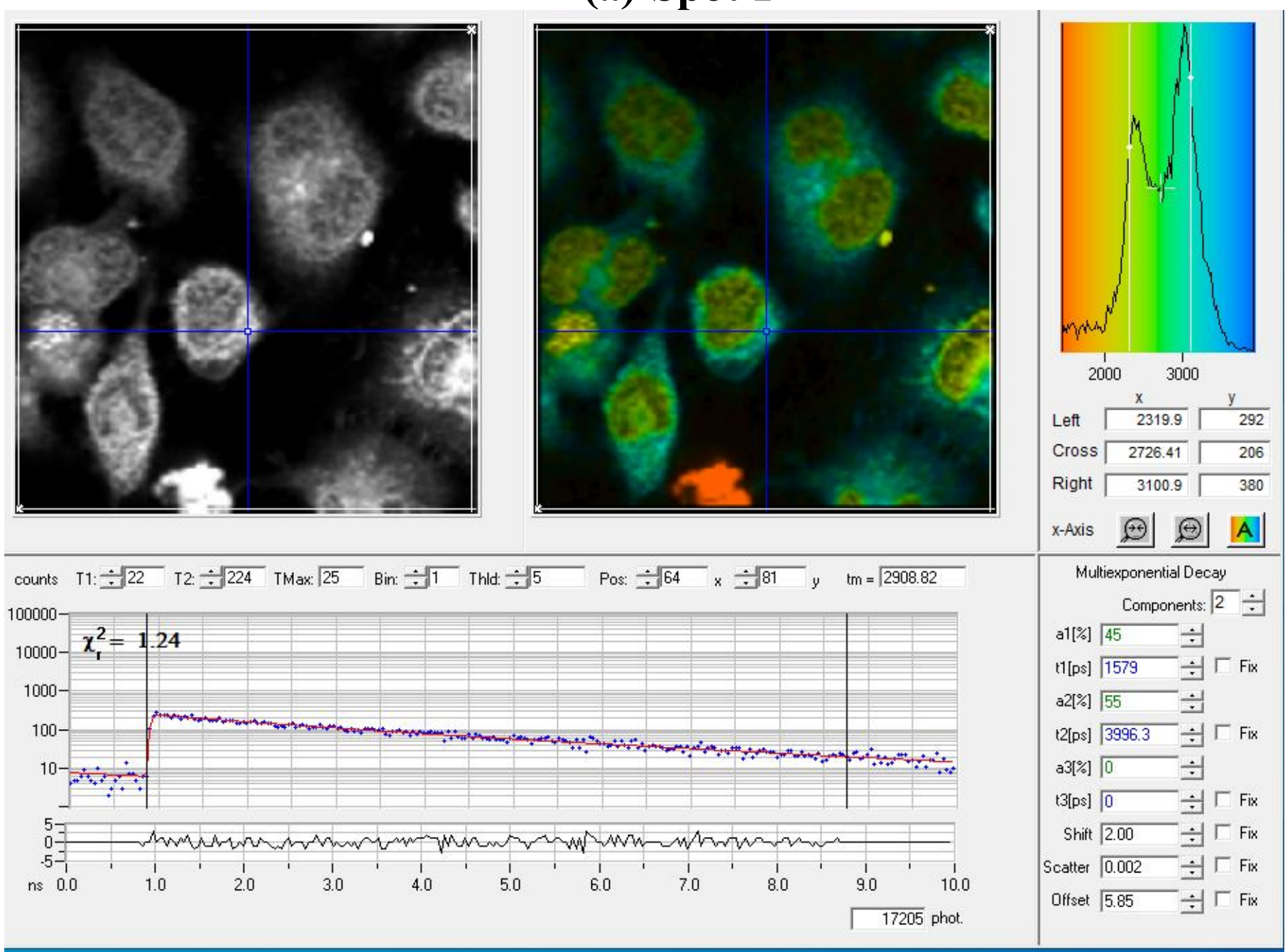

(b) Spot 2

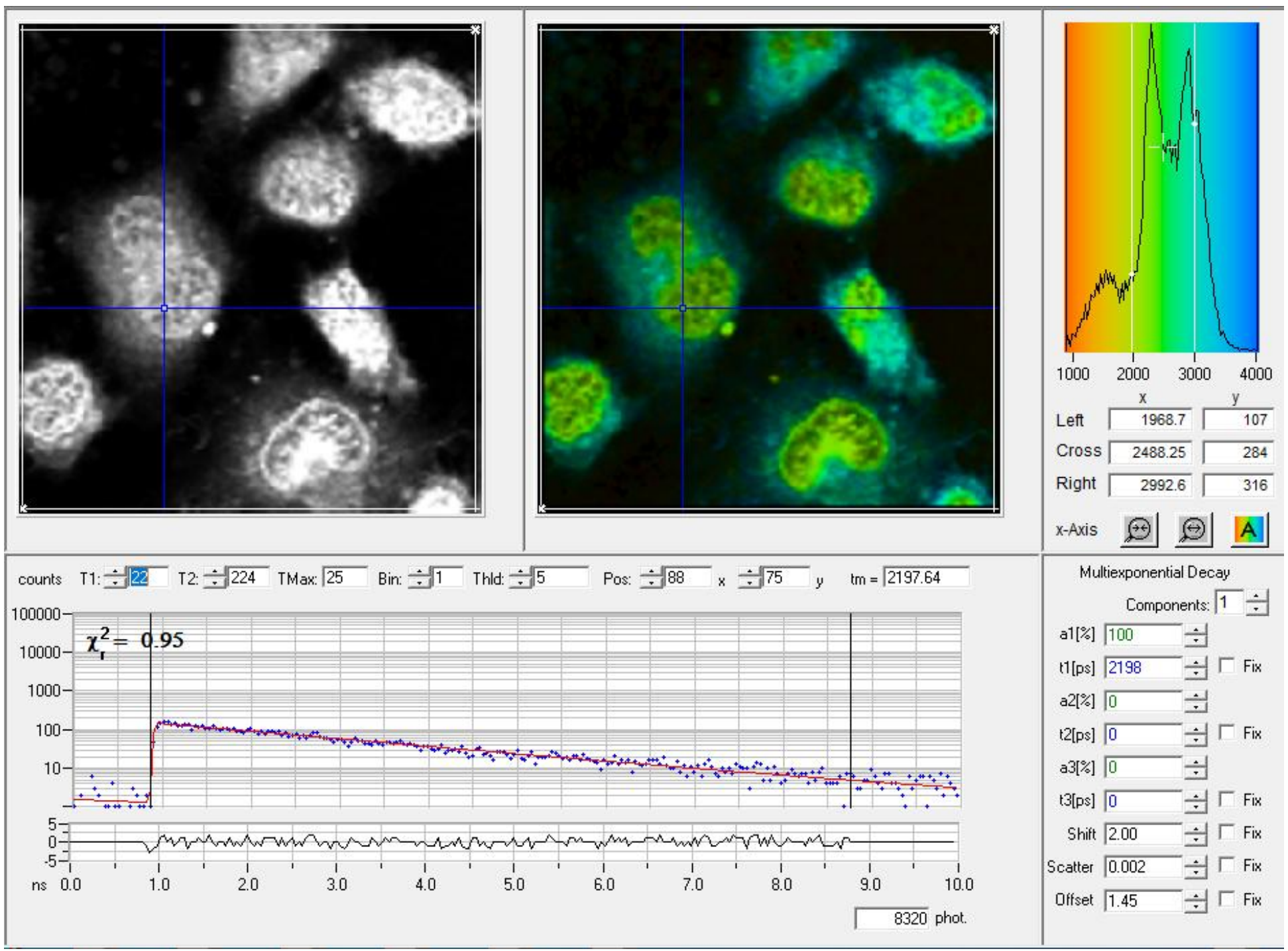

(c) Spot 3

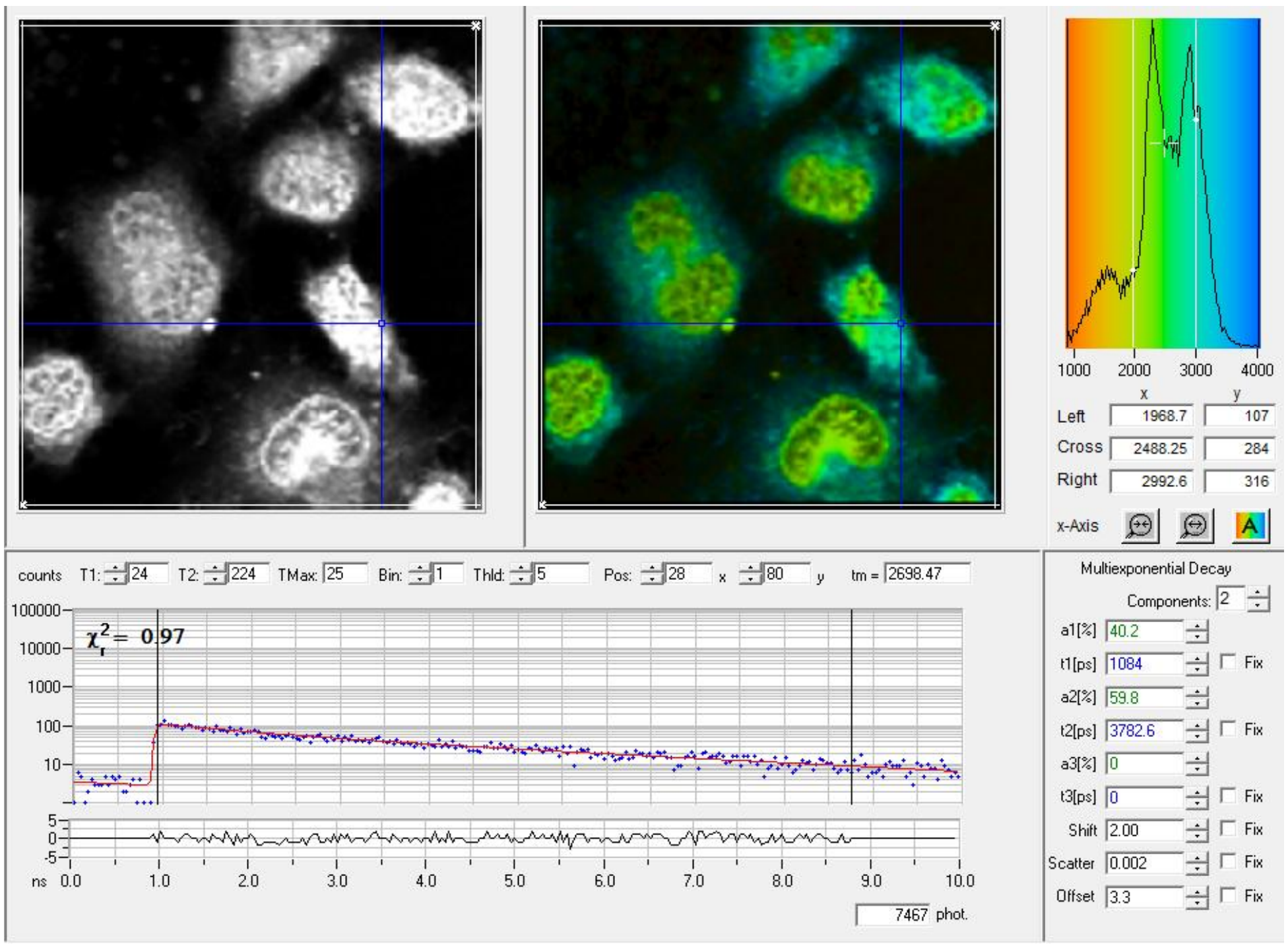

(d) Spot 4

Figure S7.24. 2P FLIM of compound **5** 100 µM in 1% DMSO in HeLa, colocalized with Hoechst nuclear stain, 60 min incubation at 37 ºC, 910 nm. For (a)-(d) and Spots 1-4, clockwise: intensity images, lifetime distribution maps and parameters including TCSPC in 4 selected spots in the nucleus and cytoplasm. Field of view: 100 µm, Lifetime scale bar: τ_m=1500-4000 ps (a-b) and τ_m=1000-4000 ps (c-d).

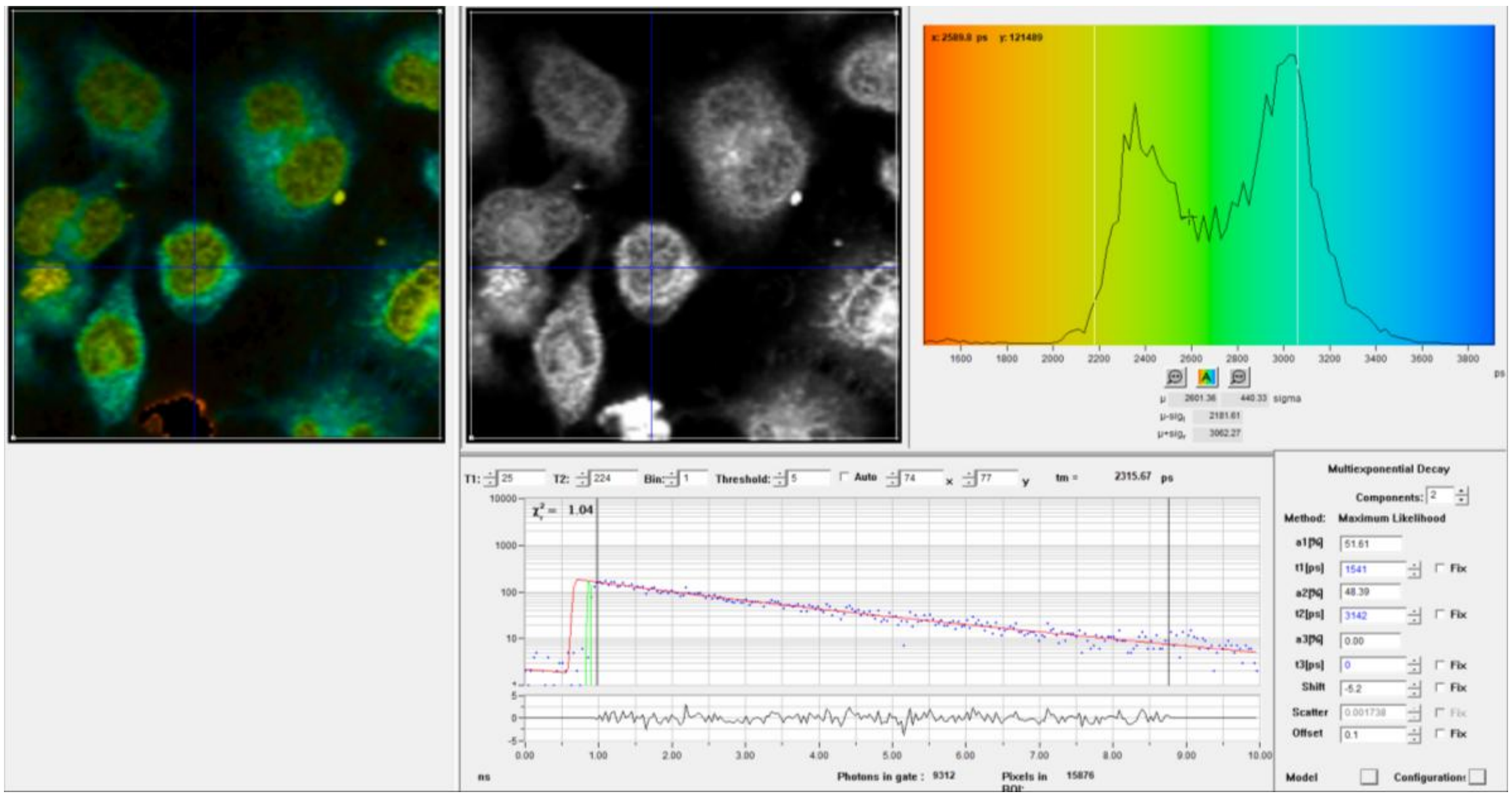

(a) Spot 5

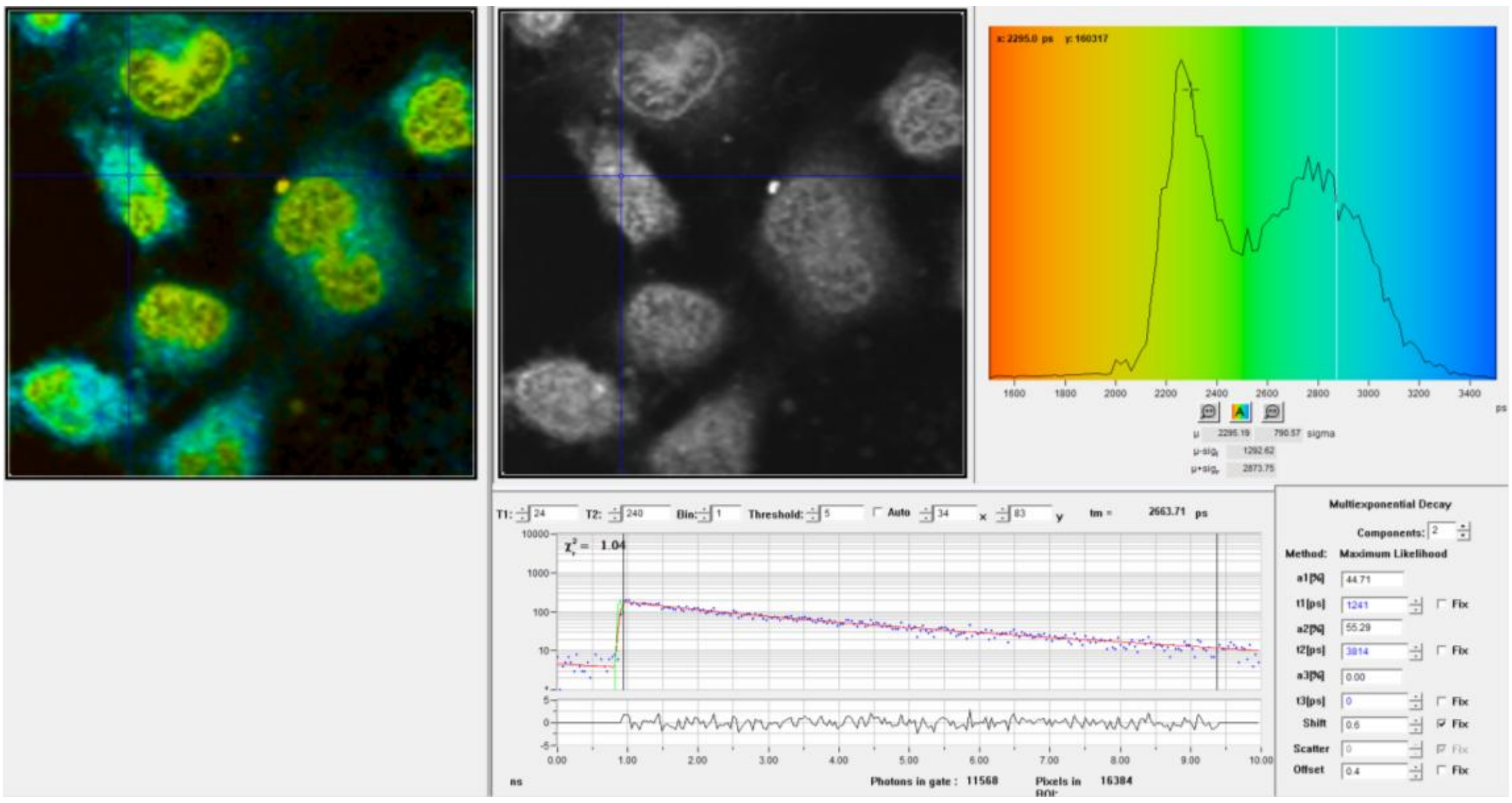

(b) Spot 6

Figure S7.25. Alternative lifetime ranges and selected spots: 2P FLIM of compound **5** 100 µM in 1% DMSO in HeLa, colocalized with Hoechst nuclear stain, 60 min incubation at 37 ºC, 910 nm. In (a)-(b) and Spots 5-6, respectively, clockwise: lifetime distribution maps, intensity images, and FLIM parameters including TCSPC in selected spots. Field of view: 100 µm, Lifetime scale bars: τ_m=1400-3900 ps (a) and τ_m=1400-3500 ps (b).

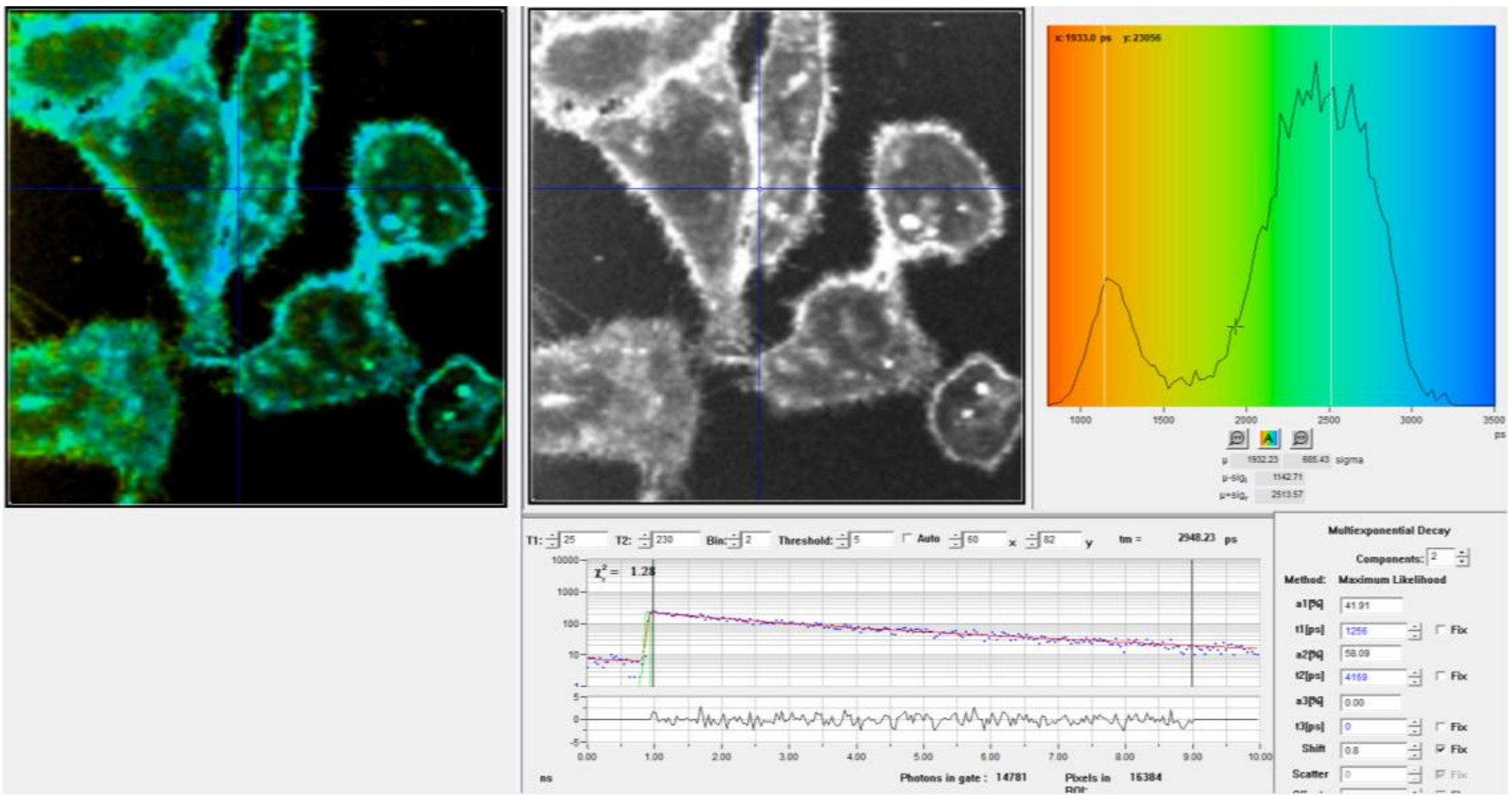

Figure S7.26. 2P FLIM of compound **5**, 100 µm in 1% DMSO in HeLa, 15 min incubation at 37 ºC, 910 nm, (clockwise: intensity image, lifetime distribution map and parameters including TCSPC in a random spot in cytoplasm). Field of view: 100 µm, Lifetime scale bars: τ_m=500-3500 ps.

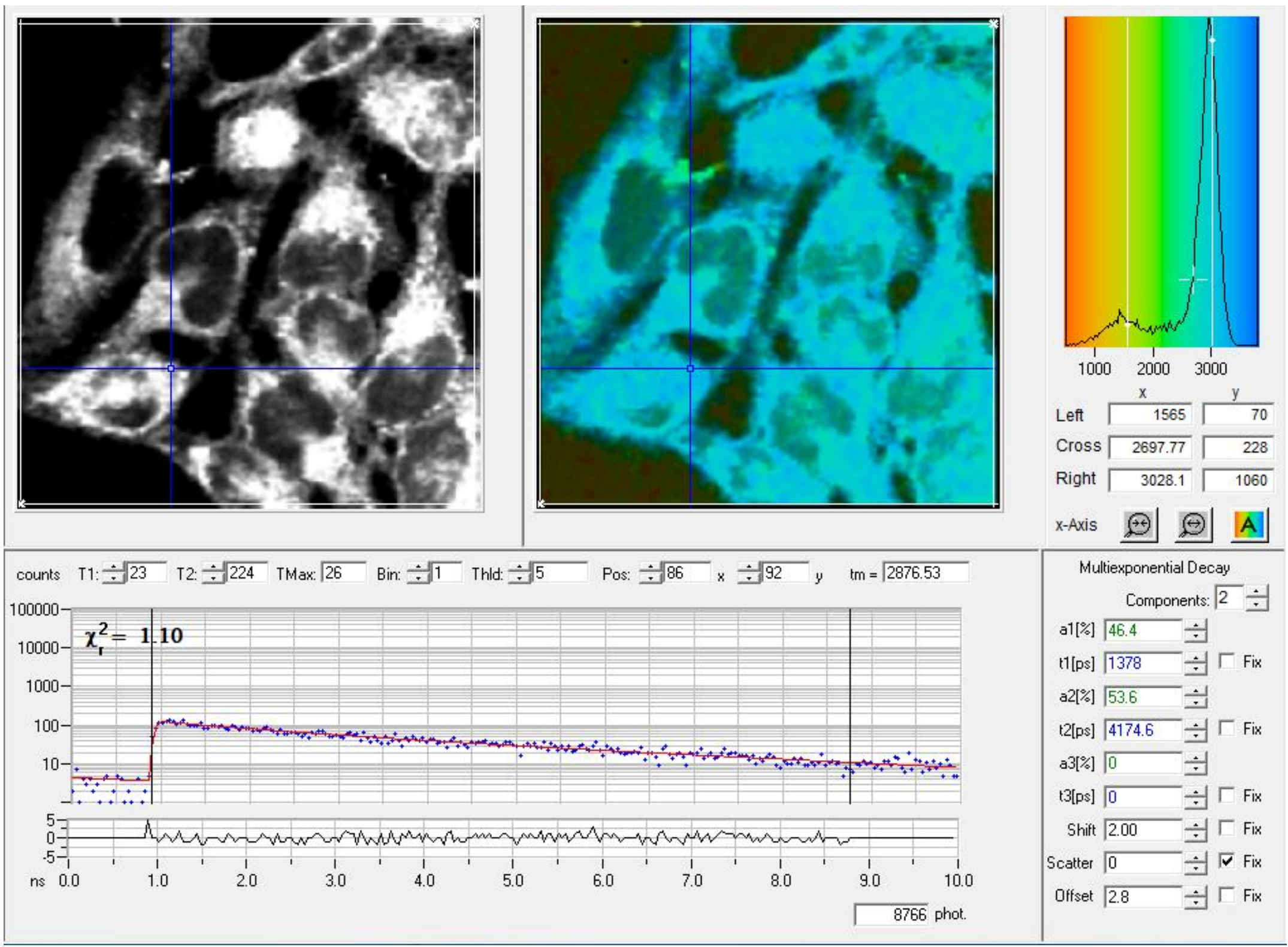

Figure S7.27. 2P FLIM of compound **5**, 100 µM in 1% DMSO in HeLa, 60 min incubation at 37 ºC, 910 nm, (clockwise: intensity image, lifetime distribution map and parameters including TCSPC in a random spot in cytoplasm). Field of view: 100 µm, Lifetime scale bars: τ_m=500-4000 ps.

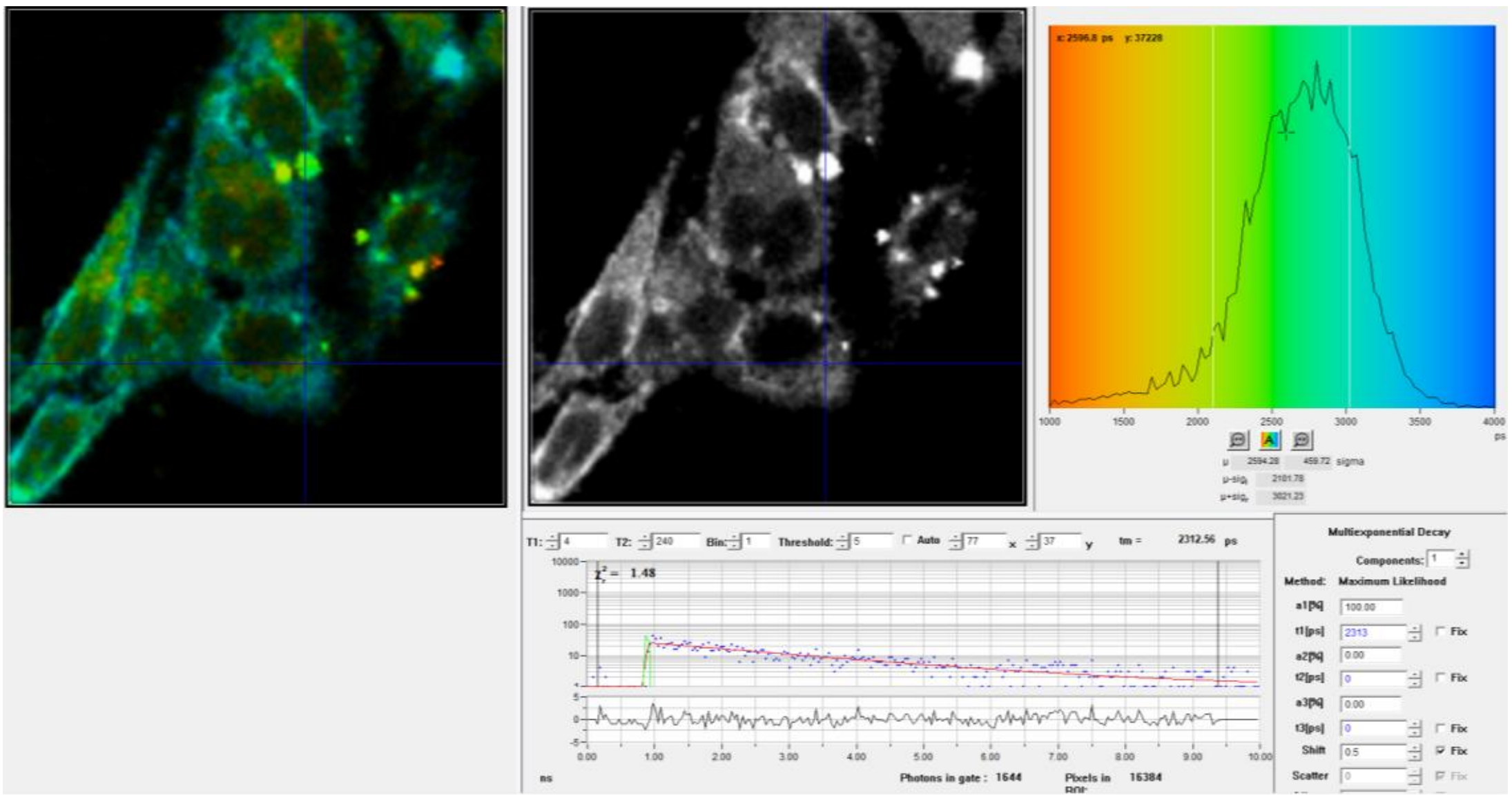

Figure S7.28. 2P FLIM of the hybrid **5@glucan** in 2% DMSO in HeLa, containing 100 µM of **5** formulated with excess (1 mg/mL) beta-D-glucan, 15 min incubation at 37 ºC, 910 nm, (clockwise: lifetime distribution map and intensity image, and parameters including TCSPC in a random spot in cytoplasm). Field of view: 100 µm, Lifetime scale bars: τ_m=1000-4000 ps.

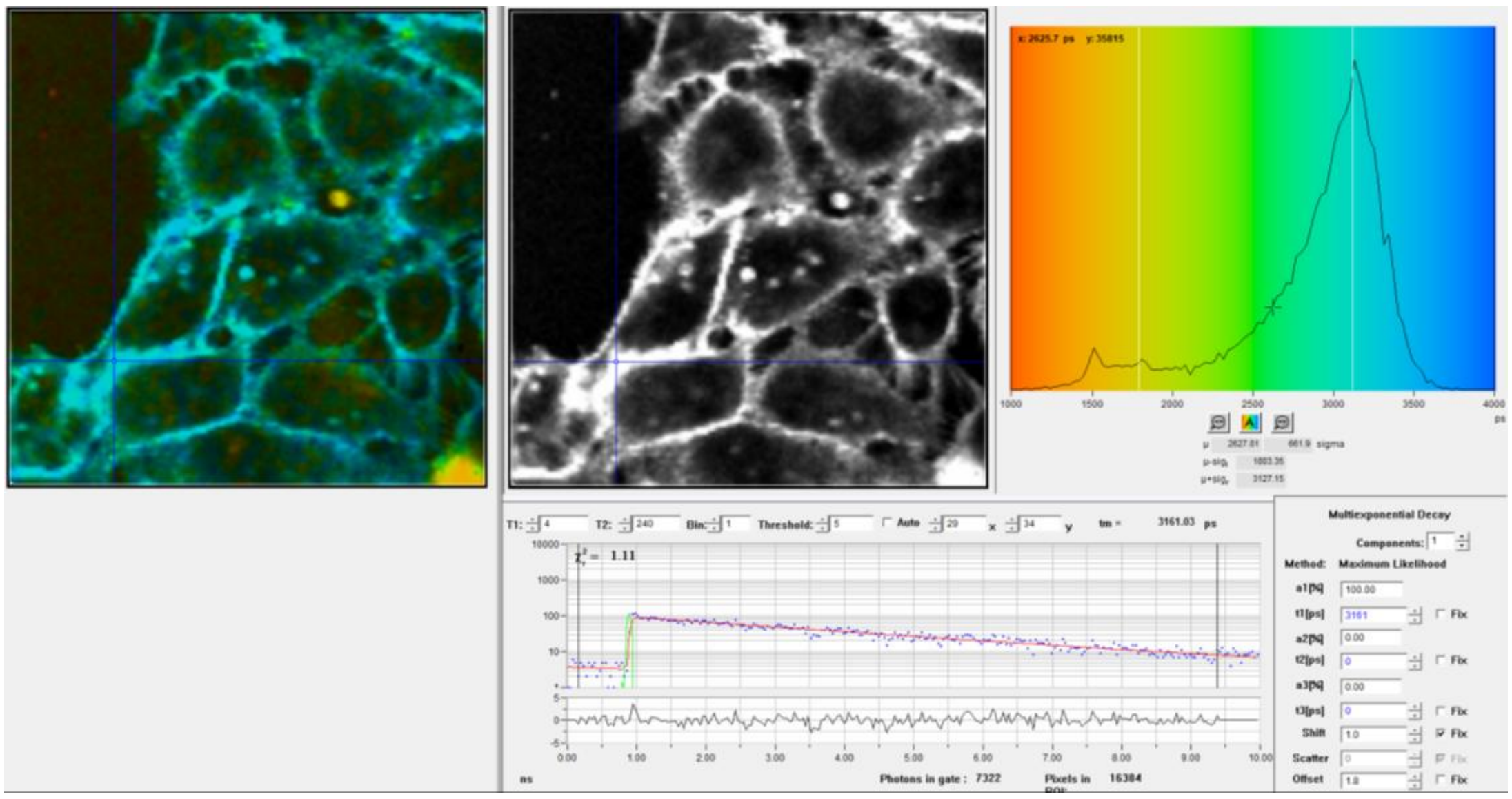

Figure S7.29. 2P FLIM of the hybrid **5@glucan** in 2% DMSO in HeLa, containing 100 µM of **5** formulated with excess (1 mg/mL) beta-D-glucan, 4 ºC incubation, 60 min, 910 nm, (clockwise: intensity image, lifetime distribution map and parameters including TCSPC in a random spot in cytoplasm). Field of view: 100 µm, Lifetime scale bars: τ_m=1000-4000 ps.

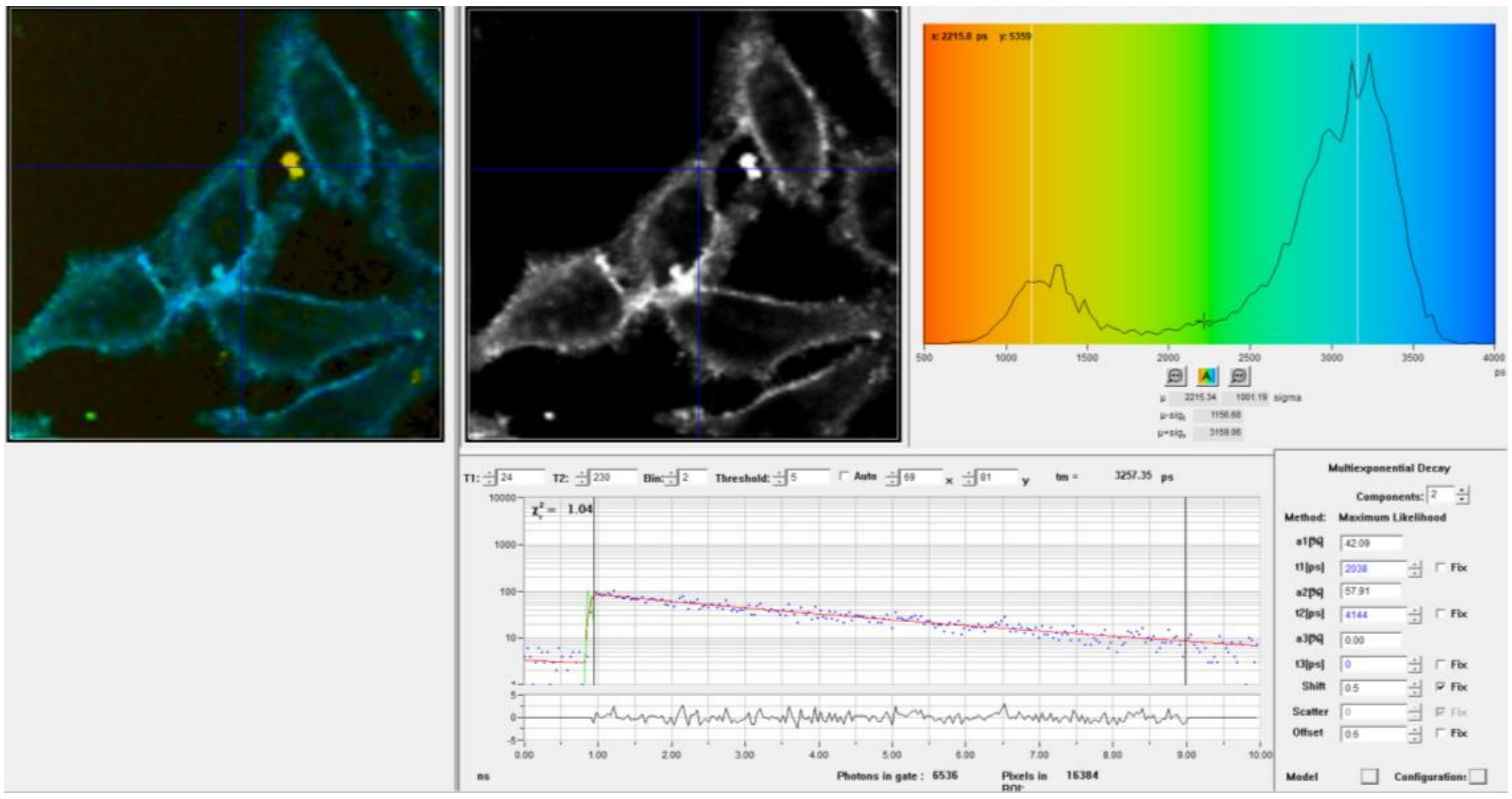

Figure S7.30. 2P FLIM of compound **5**, 100 µm in 1% DMSO in HeLa, 15 min incubation at 4 ºC, 910 nm, (clockwise: intensity image, lifetime distribution map and parameters including TCSPC in a random spot in cytoplasm). Field of view: 100 µm, Lifetime scale bars: τ_m=500-4000 ps.

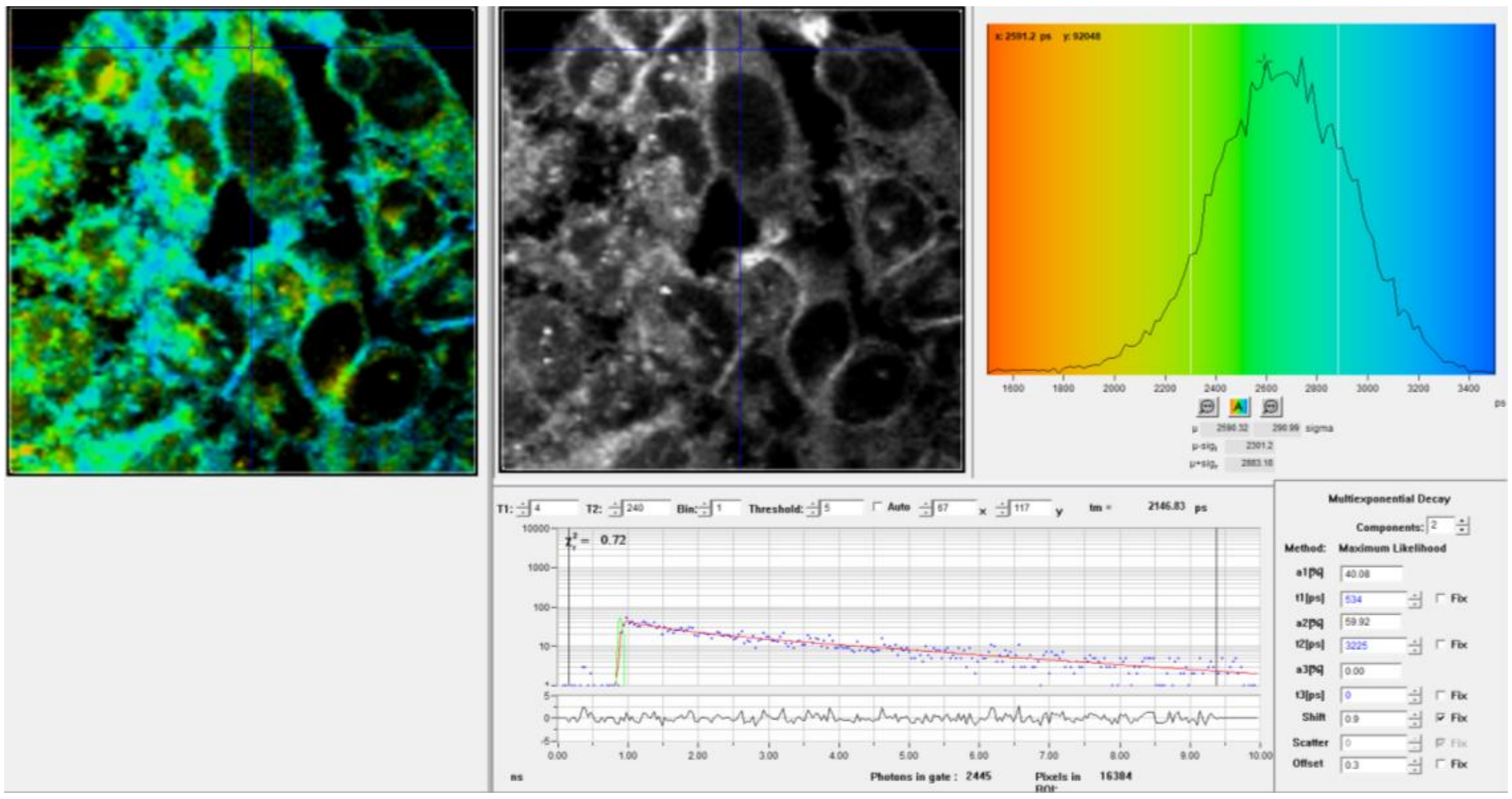

Figure S7.31. 2P FLIM of the hybrid **5@glucan** in 2% DMSO in HeLa, containing 100 µM of **5** formulated with excess (1 mg/mL) beta-D-glucan, 37 ºC incubation, 15 min, 910 nm, (clockwise: intensity image, lifetime distribution map and parameters including TCSPC in a random spot in cytoplasm). Field of view: 100 µm, Lifetime scale bars: τ_m=1500-3500 ps.

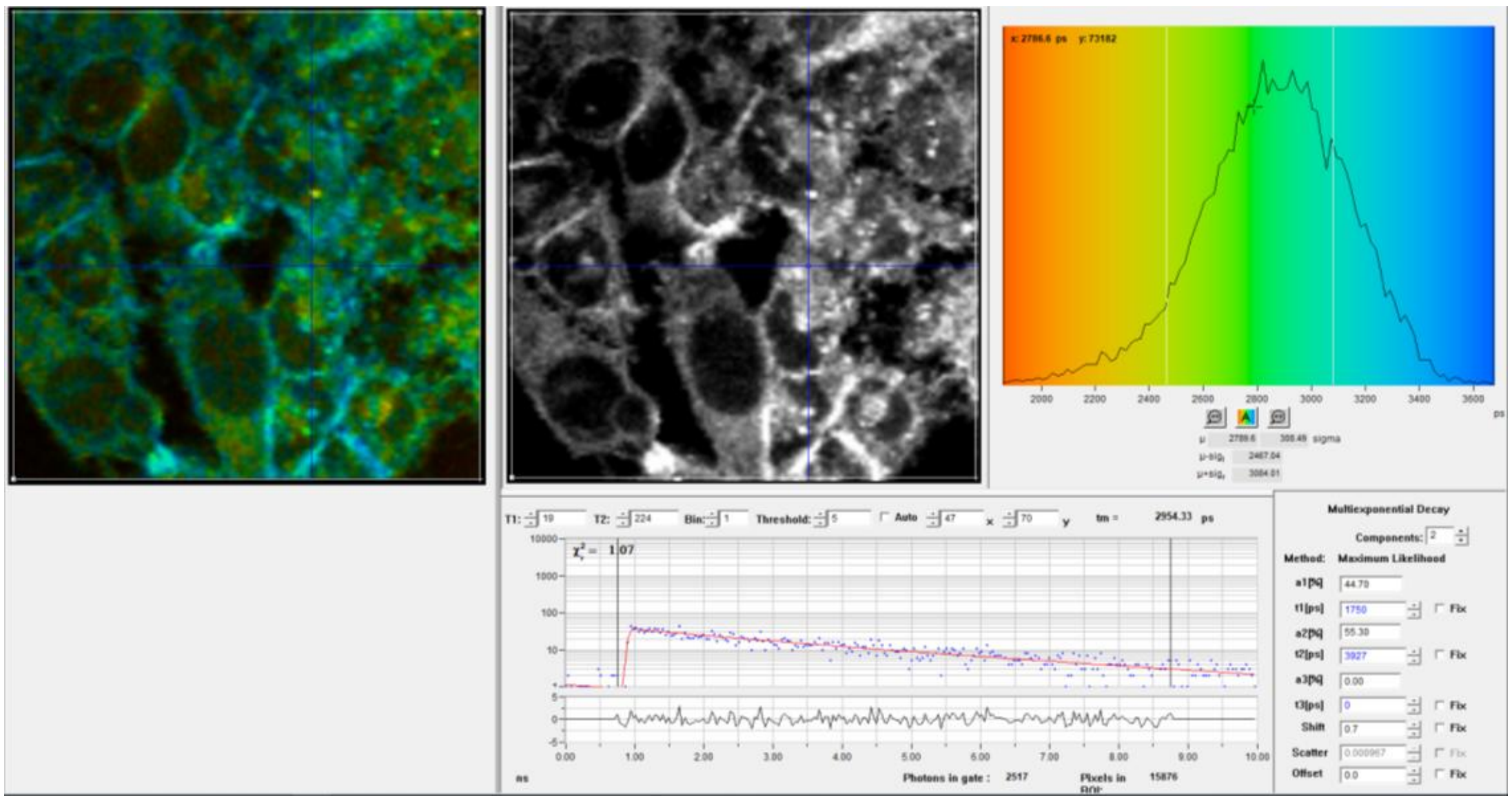

Figure S7.32. 2P FLIM of the hybrid **5@glucan** in 2% DMSO in HeLa, containing 100 µM of **5** formulated with excess (1 mg/mL) beta-D-glucan, 37 ºC incubation, 15 min, 910 nm, (clockwise: intensity image, lifetime distribution map and parameters including TCSPC in a random spot in cytoplasm). Field of view: 100 µm, Lifetime scale bars: τ_m=1500-3500 ps.

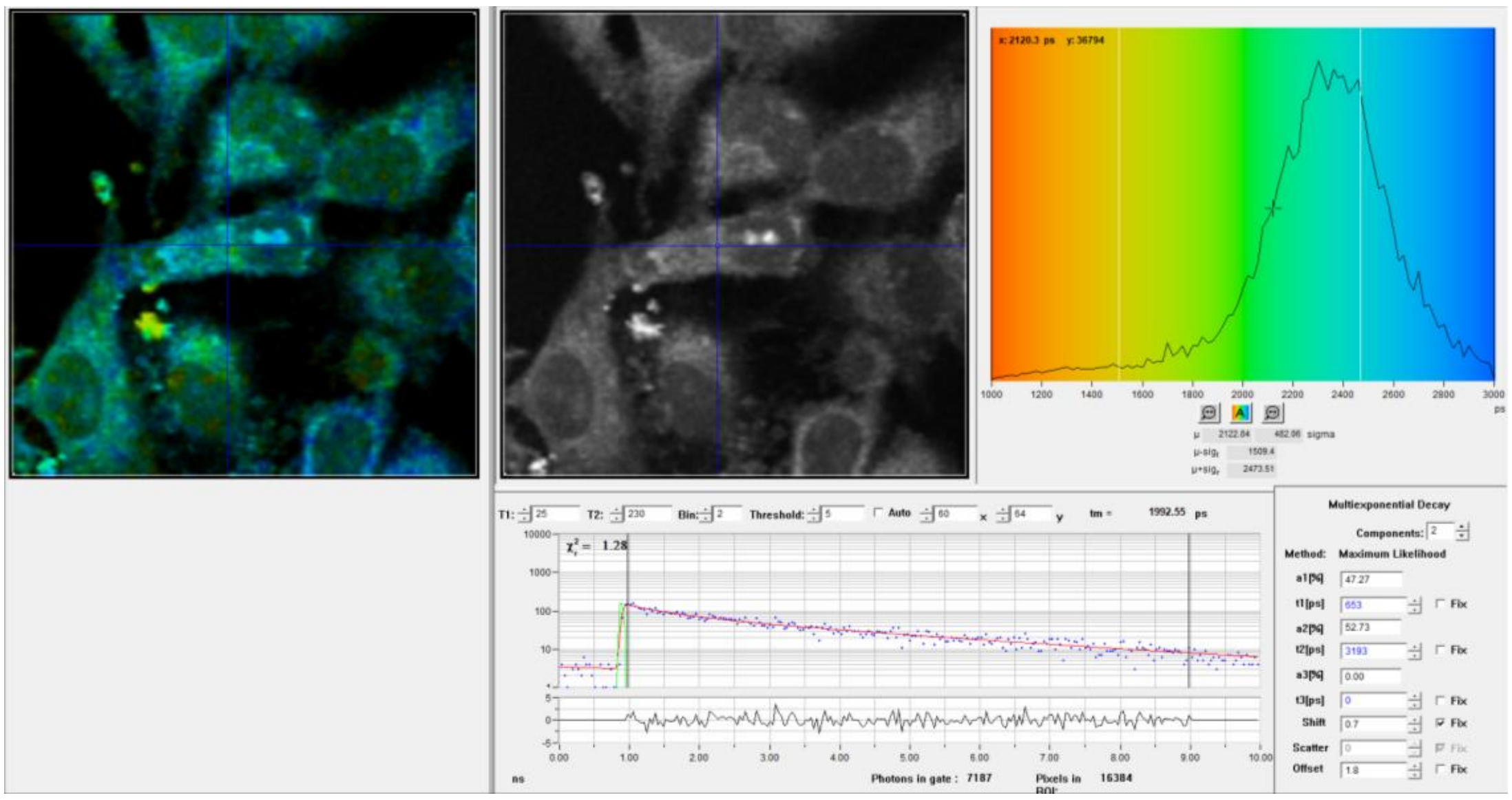

Figure S7.33. 2P FLIM of the hybrid **5@glucan** in 2% DMSO in HeLa, containing 100 µM of **5** formulated with excess (1 mg/mL) beta-D-glucan, 37 ºC incubation, 60 min, 910 nm, (clockwise: intensity image, lifetime distribution map and parameters including TCSPC in a random spot in cytoplasm). Field of view: 100 µm, Lifetime scale bars: τ_m=1000-3500 ps.

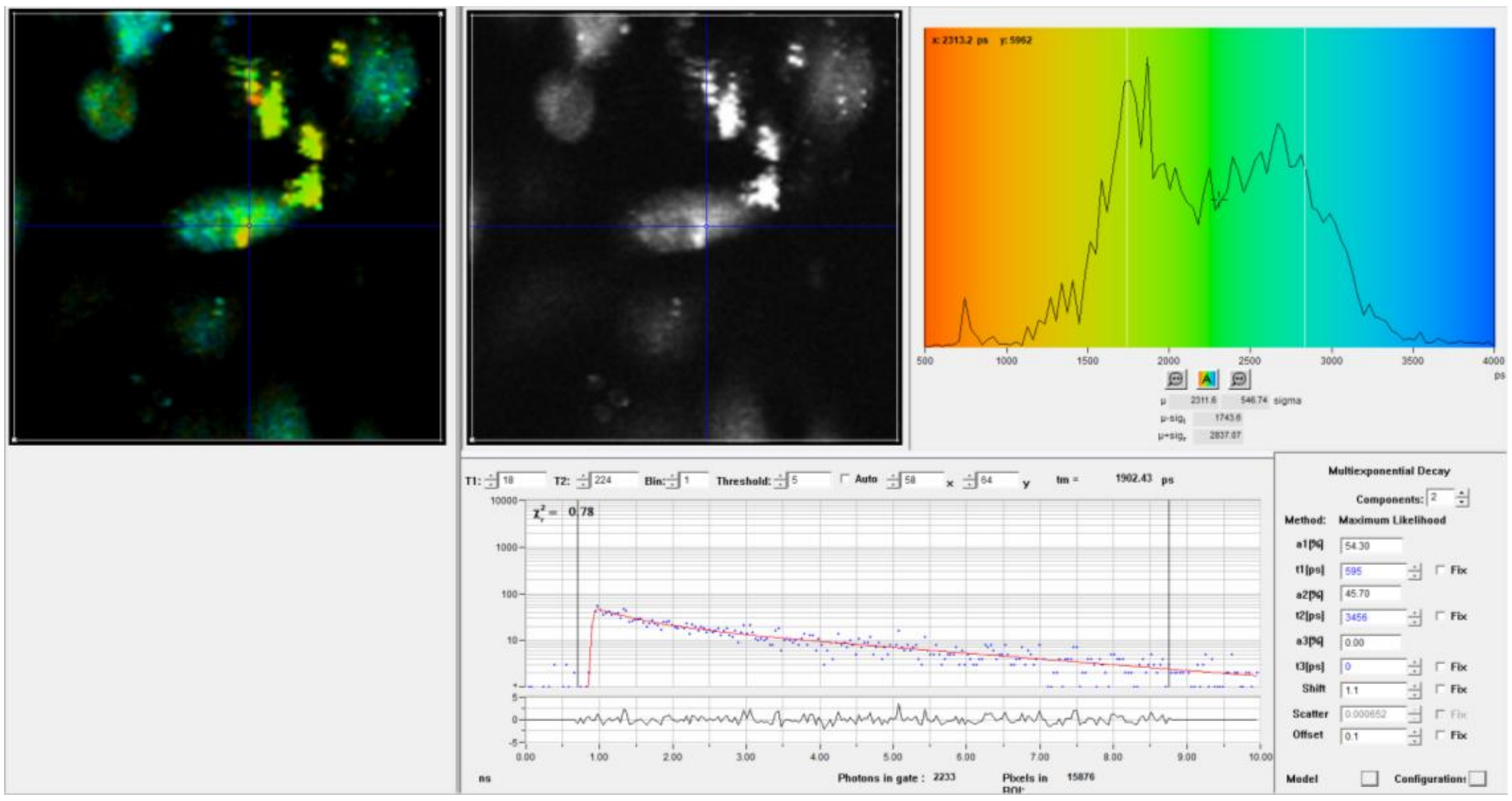

Figure S7.34. 2P FLIM of the hybrid **5@glucan** in 2% DMSO in HeLa, containing 100 µM of **5** formulated with excess (1 mg/mL) beta-D-glucan, 37 ºC incubation, 60 min, 910 nm, (clockwise: intensity image, lifetime distribution map and parameters including TCSPC in a random spot in cytoplasm). Field of view: 100 µm, Lifetime scale bars: τ_m=500-4000 ps.

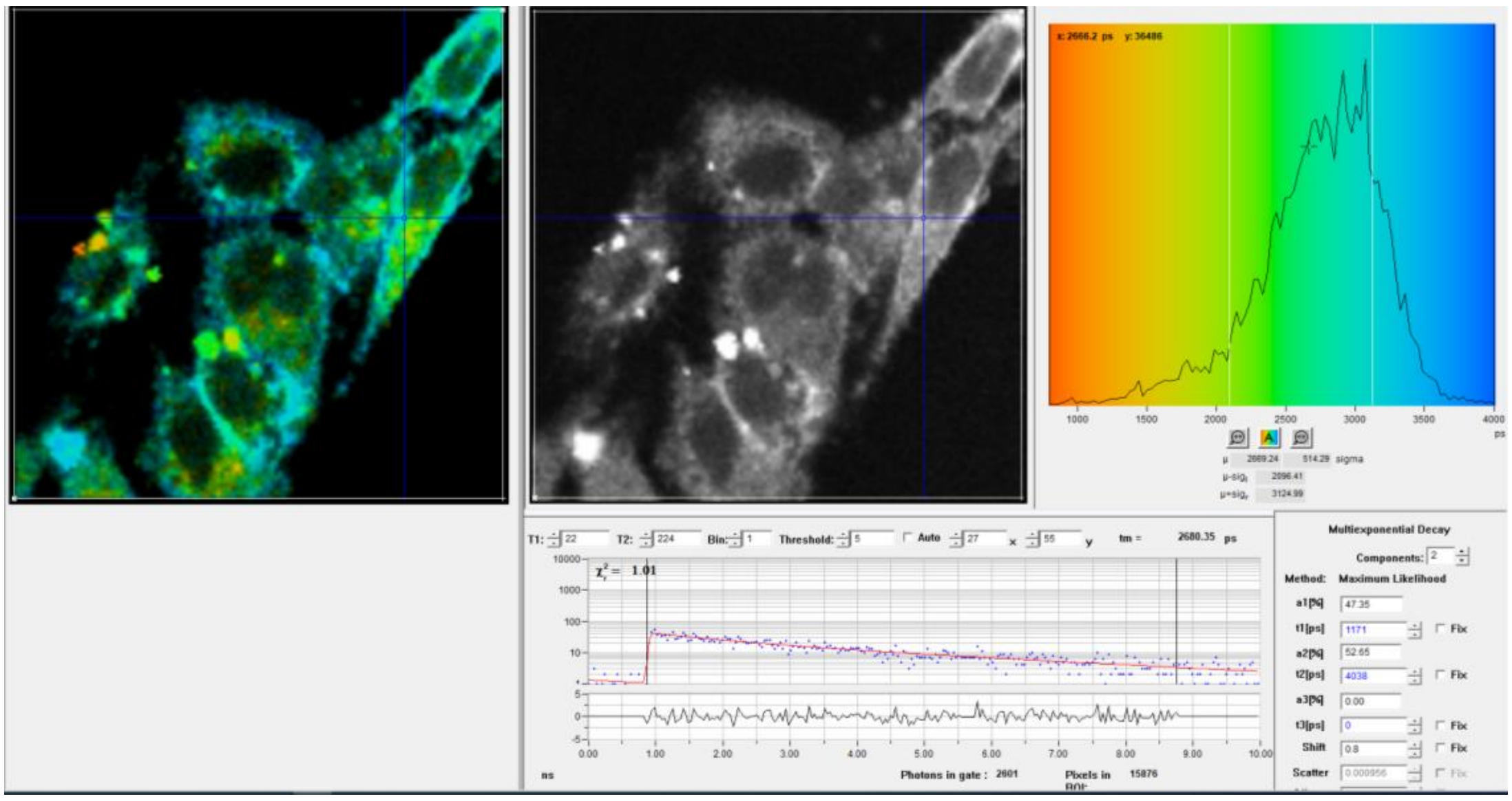

Figure S7.35. 2P FLIM of the hybrid **5@glucan** in 2% DMSO in HeLa, containing 100 µM of **5** formulated with excess (1 mg/mL) beta-D-glucan, 37 ºC incubation, 15 min, 910 nm, (clockwise: intensity image, lifetime distribution map and parameters including TCSPC in a random spot in cytoplasm). Field of view: 100 µm, Lifetime scale bars: τ_m=500-4000 ps.

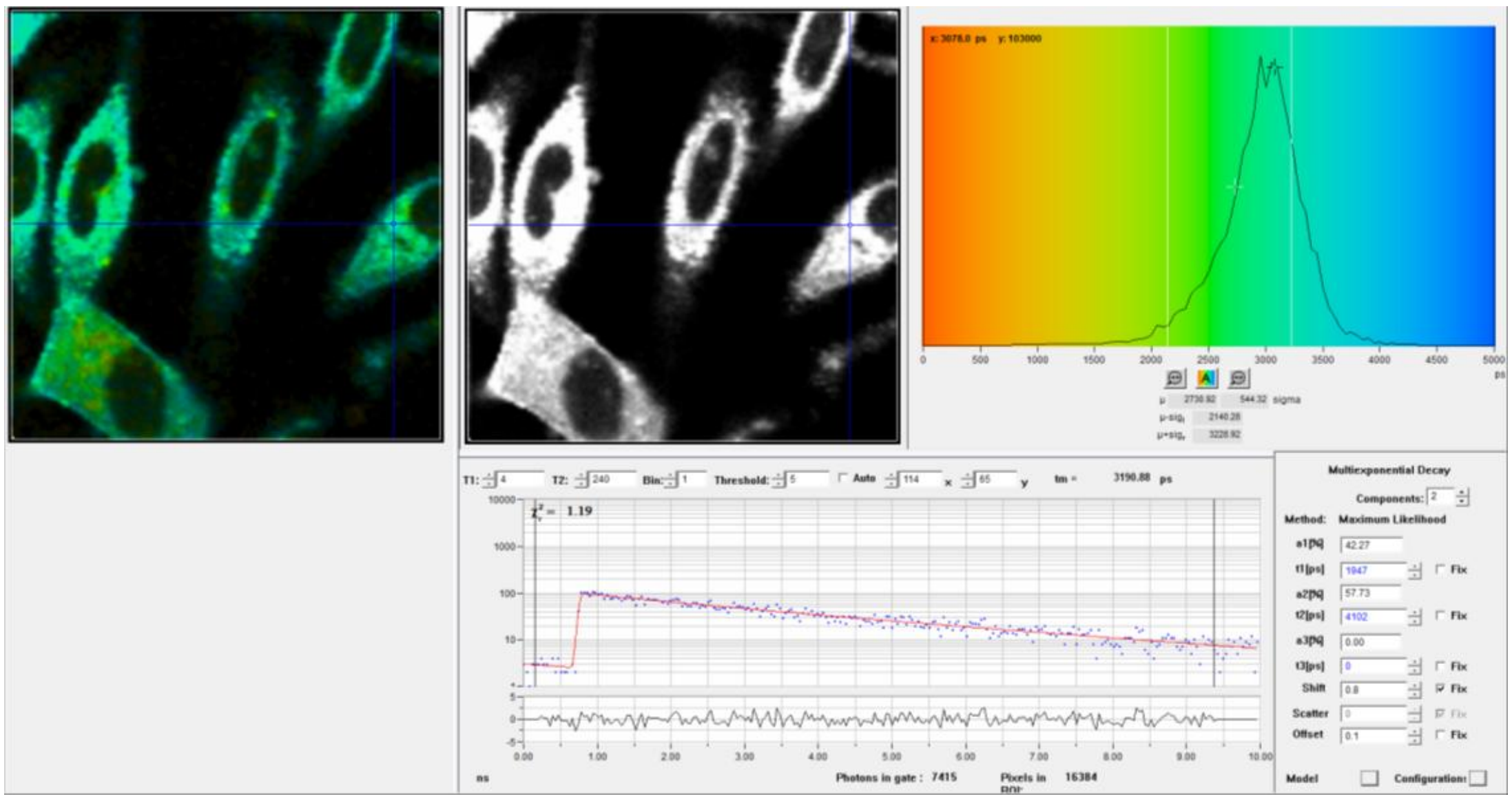

Figure S7.36. 2P FLIM of the hybrid **5@glucan** in 2% DMSO in PC-3, containing 100 µM of **5** formulated with excess (1 mg/mL) beta-D-glucan, 37 °C incubation, 15 min, 910 nm, (clockwise: intensity image, lifetime distribution map and parameters including TCSPC in a random spot in cytoplasm). Field of view: 100 µm, Lifetime scale bars: τ_m=0-5000 ps.

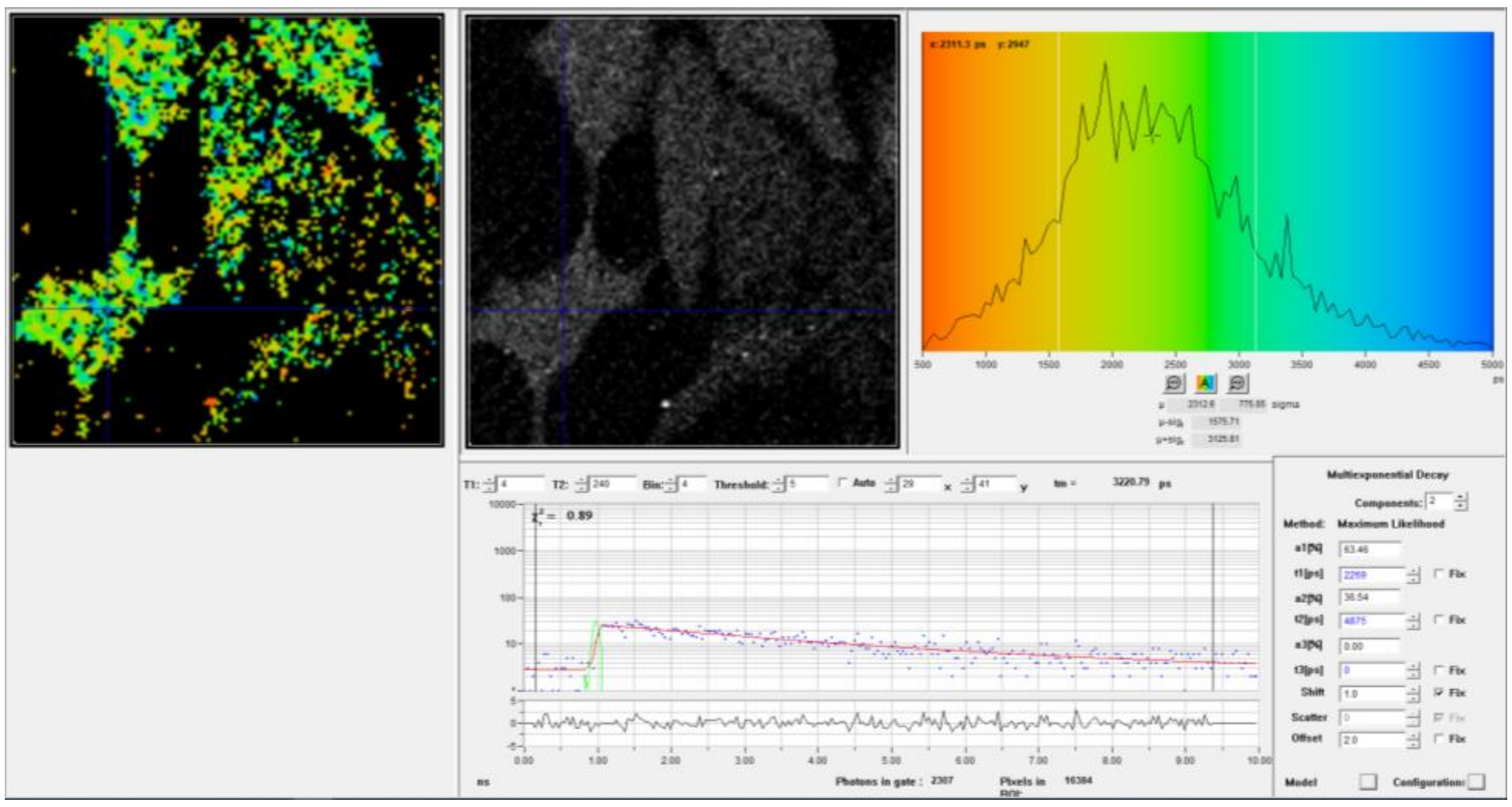

(A)

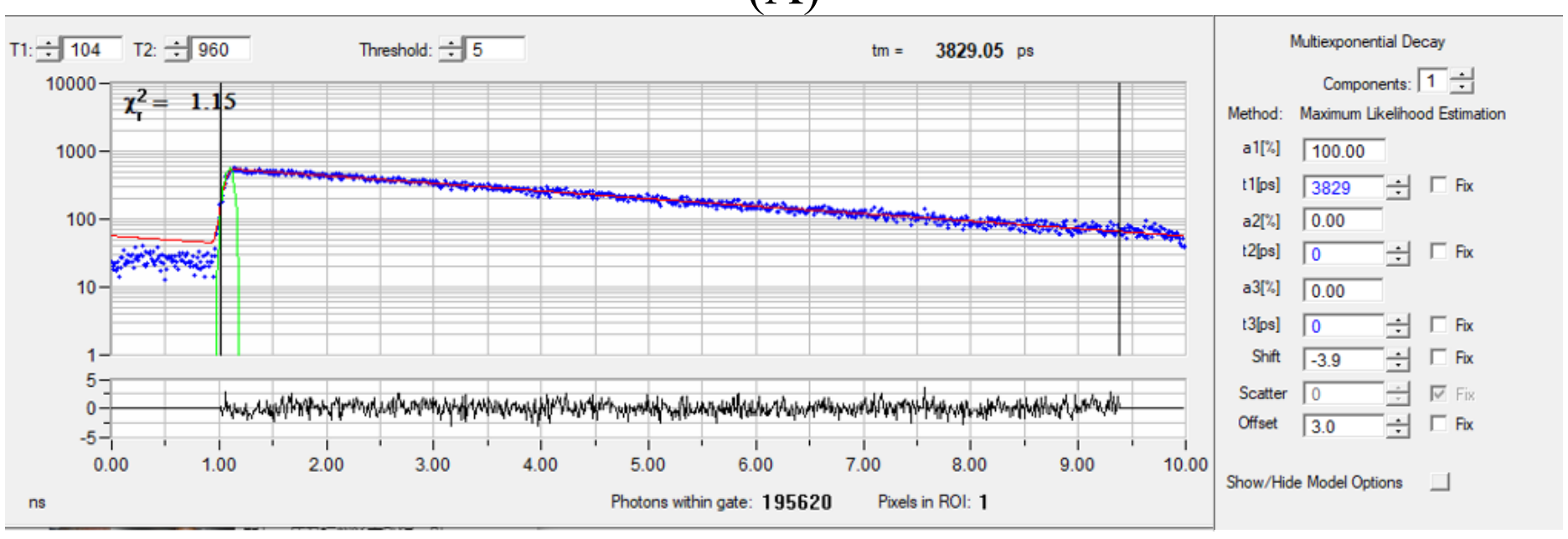

(B)

Figure S7.37. (A) 2P FLIM of the hybrid **5** in 1% DMSO in MCF-7 cells, containing 10 µM of **5**, 60 min incubation at 37 °C, 910 nm, (clockwise: lifetime distribution map and intensity image, and parameters including TCSPC in a random spot in cytoplasm).

Field of view: 100 µm, Lifetime scale bars: τ_m=500-5000 ps; **(B)** 2P TCSPC lifetime measurement of **5** 10 µM in 1:1 DMSO:PBS.

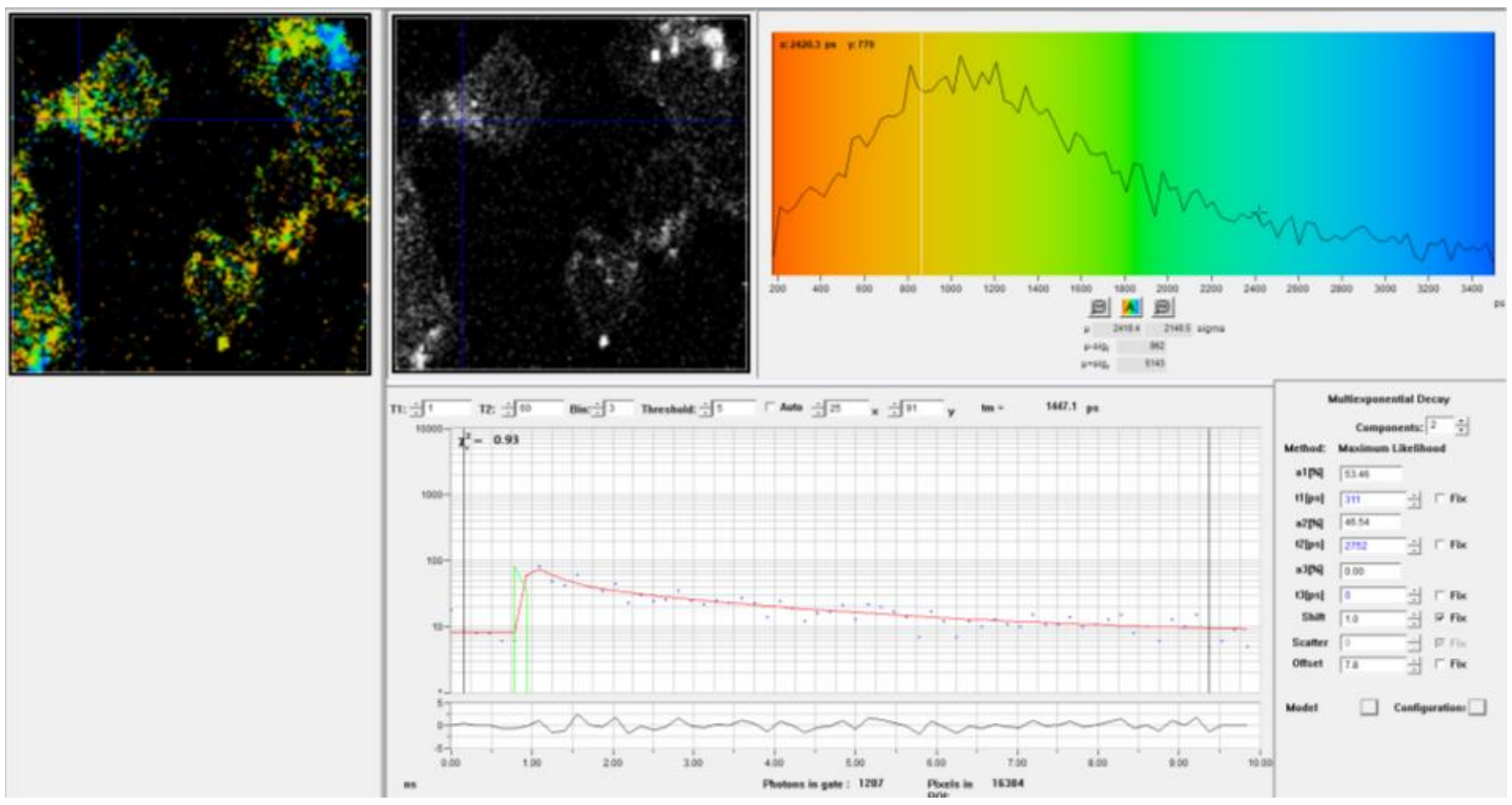

(A)

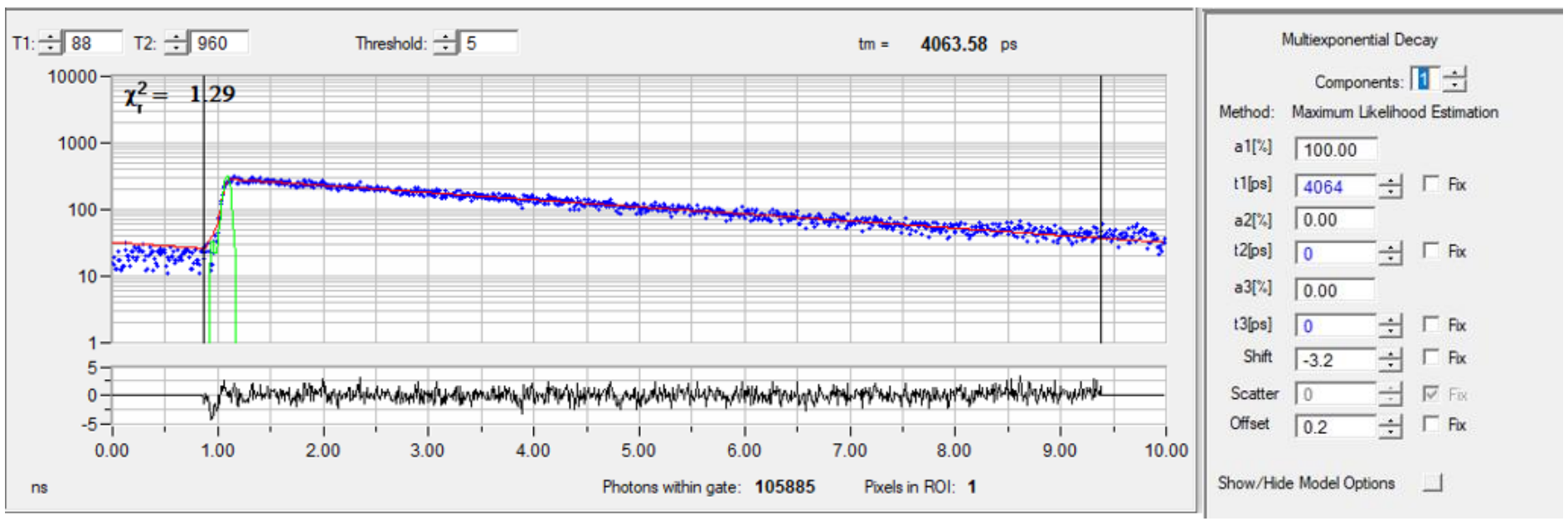

(B)

Figure S7.38. (A) 2P FLIM of the hybrid **5@glucan** in 1% DMSO in MCF-7 cells, containing 10 µM of **5** formulated with excess (1 mg/mL) beta-D-glucan, 15 min incubation at 37 ºC, 910 nm, (clockwise: lifetime distribution map and intensity image, and parameters including TCSPC in a random spot in cytoplasm). Field of view: 100 µm, Lifetime scale bars: τ_m=200-3500 ps; **(B)** 2P TCSPC lifetime measurement of **5@glucan** containing 10 µM of **5** formulated with excess (1 mg/mL) beta-D-glucan in 1:1 DMSO:PBS.

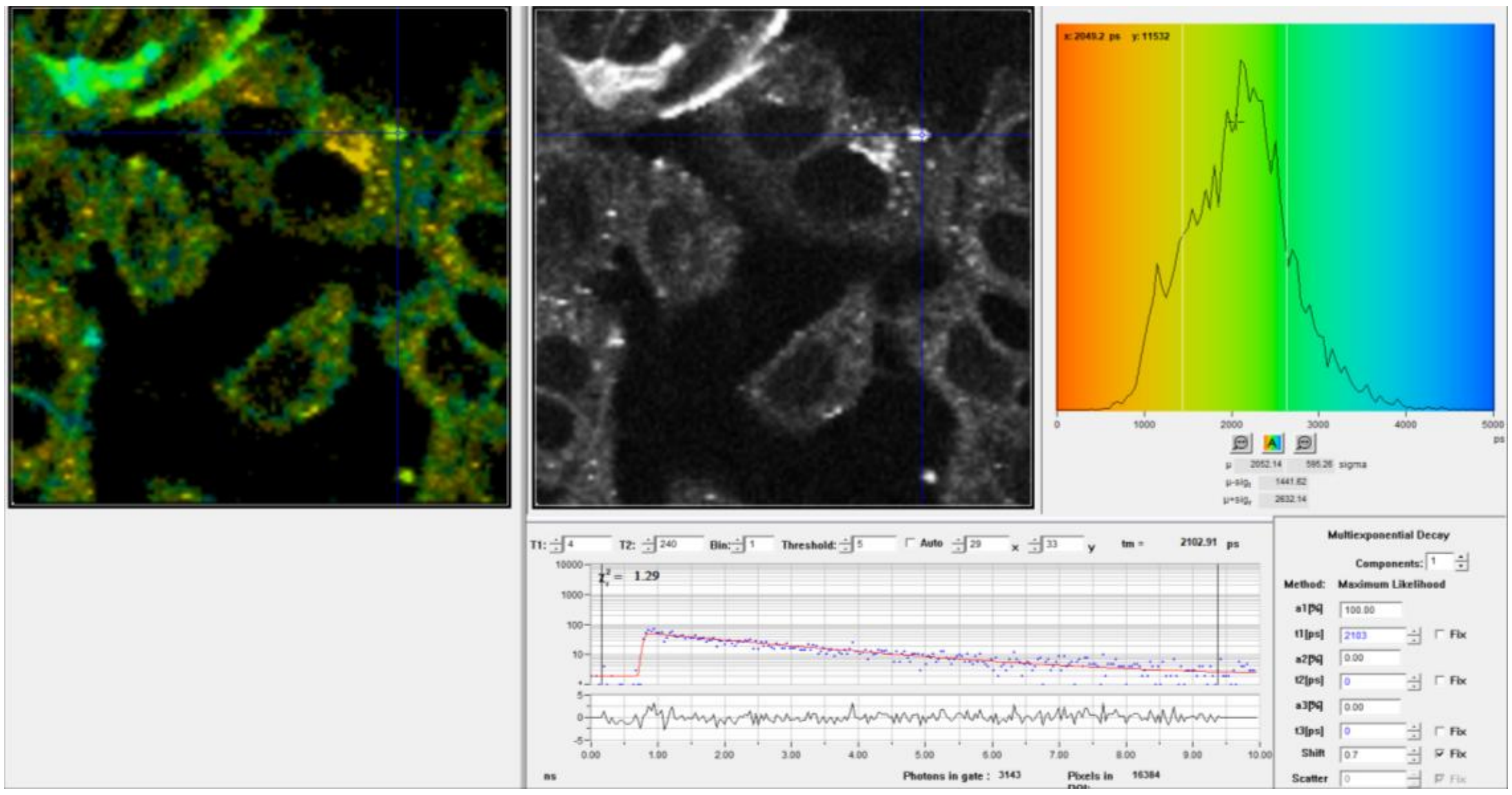

(A) Spot 1

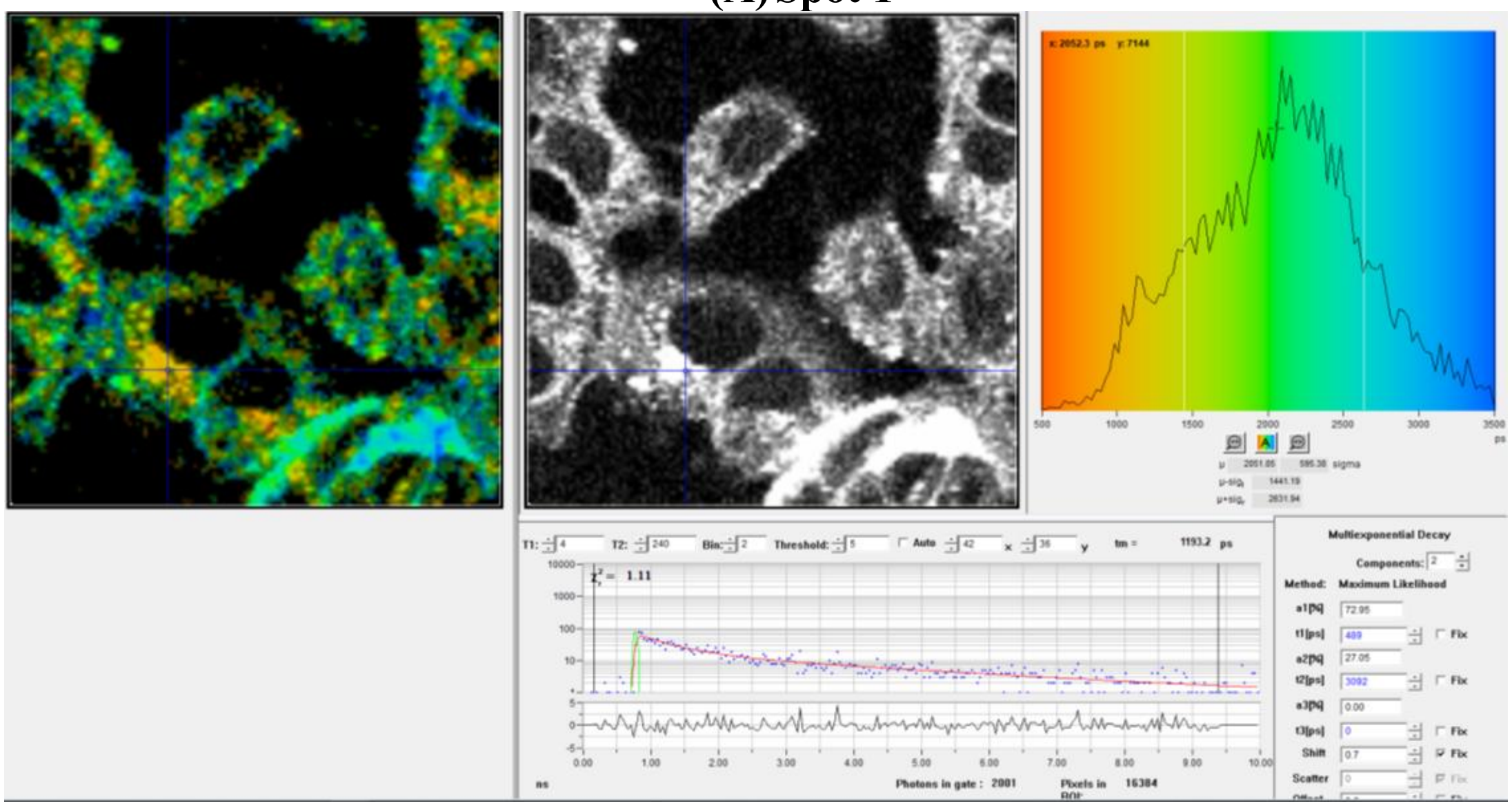

(B) Spot 2

Figure S7.39. 2P FLIM of the hybrid **5@glucan** in 1% DMSO in MCF-7 cells, containing 100 µM of **5** formulated with excess (1 mg/mL) beta-D-glucan, 15 min incubation at 37 °C, 910 nm, (clockwise: lifetime distribution map and intensity image, and parameters including TCSPC in two random spots in cellular cytoplasm). Field of view: 100 µm, Lifetime scale bars: τ_m= 0-5000 ps (A) and 500-3500 ps (B).

Compound 5 2P FLIM at 810 nm

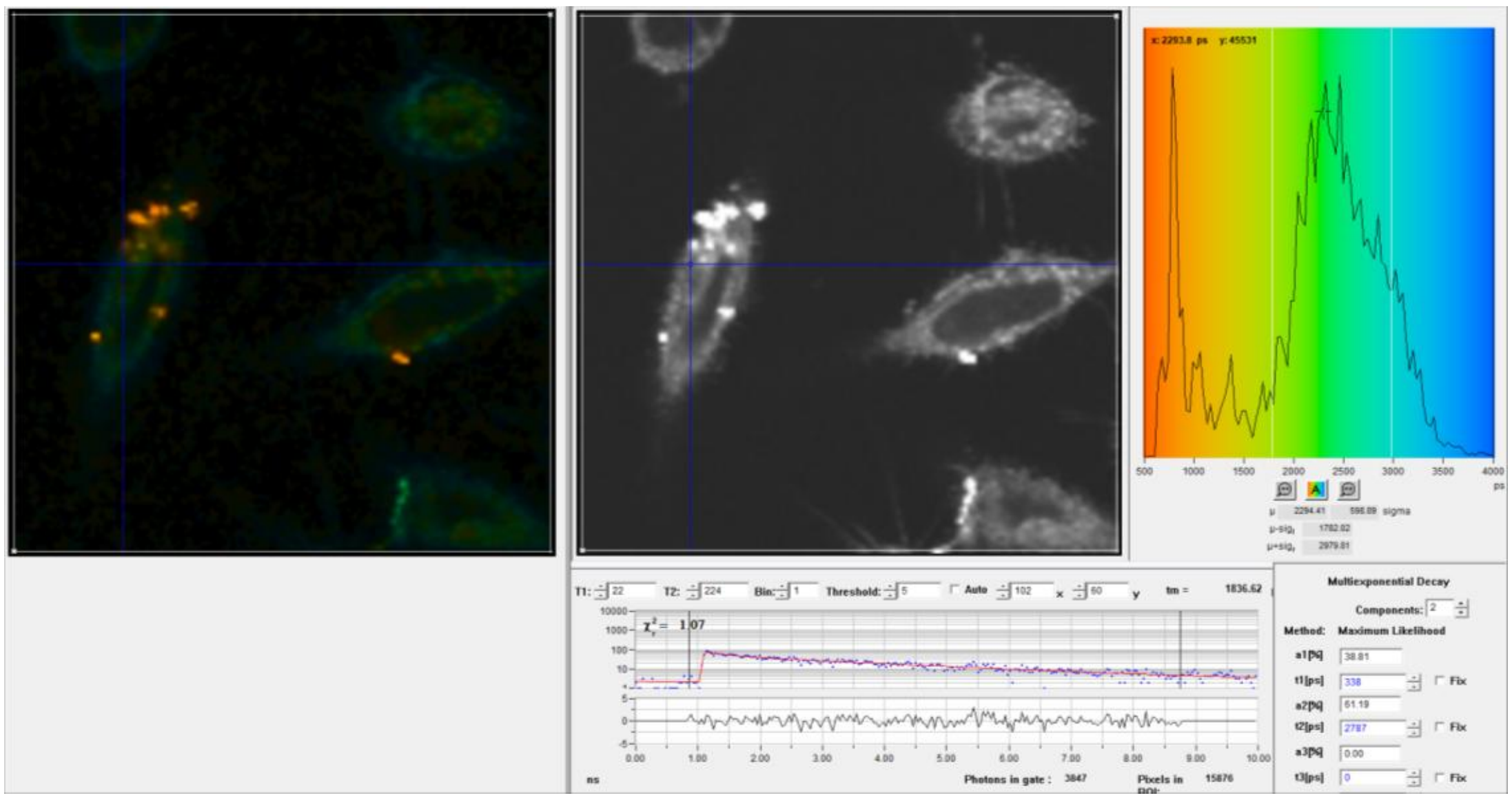

Figure S7.40. 2P FLIM of compound **5** in 1% DMSO in HeLa, 15 min incubation at 37 °C, 810 nm and 4.3 mW power (clockwise: lifetime distribution map, intensity image, and FLIM parameters including TCSPC in a random spot in cytoplasm). Field of view: 100 µm, Lifetime scale bars: τ_m=500-4000 ps.

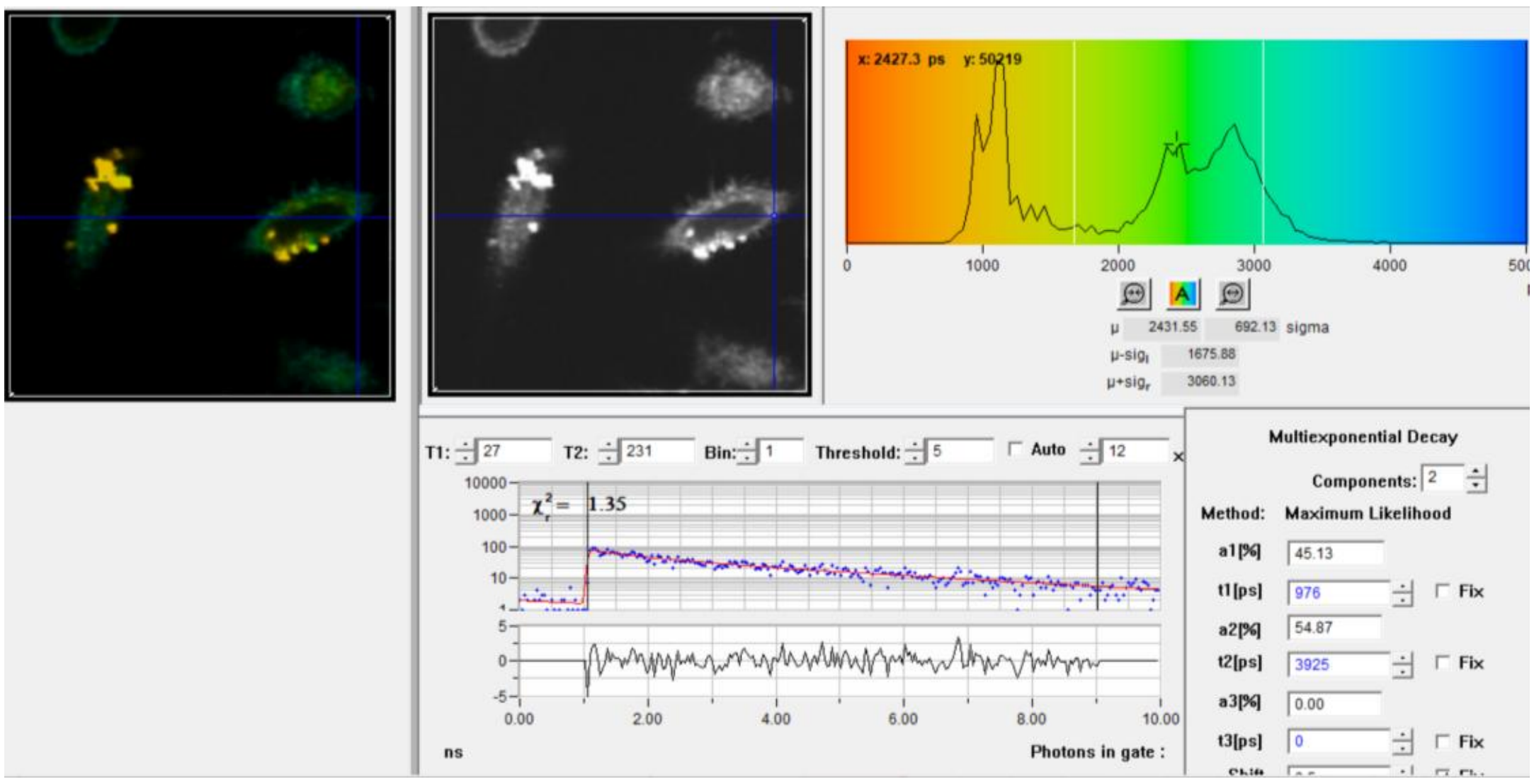

(a) Spot 1

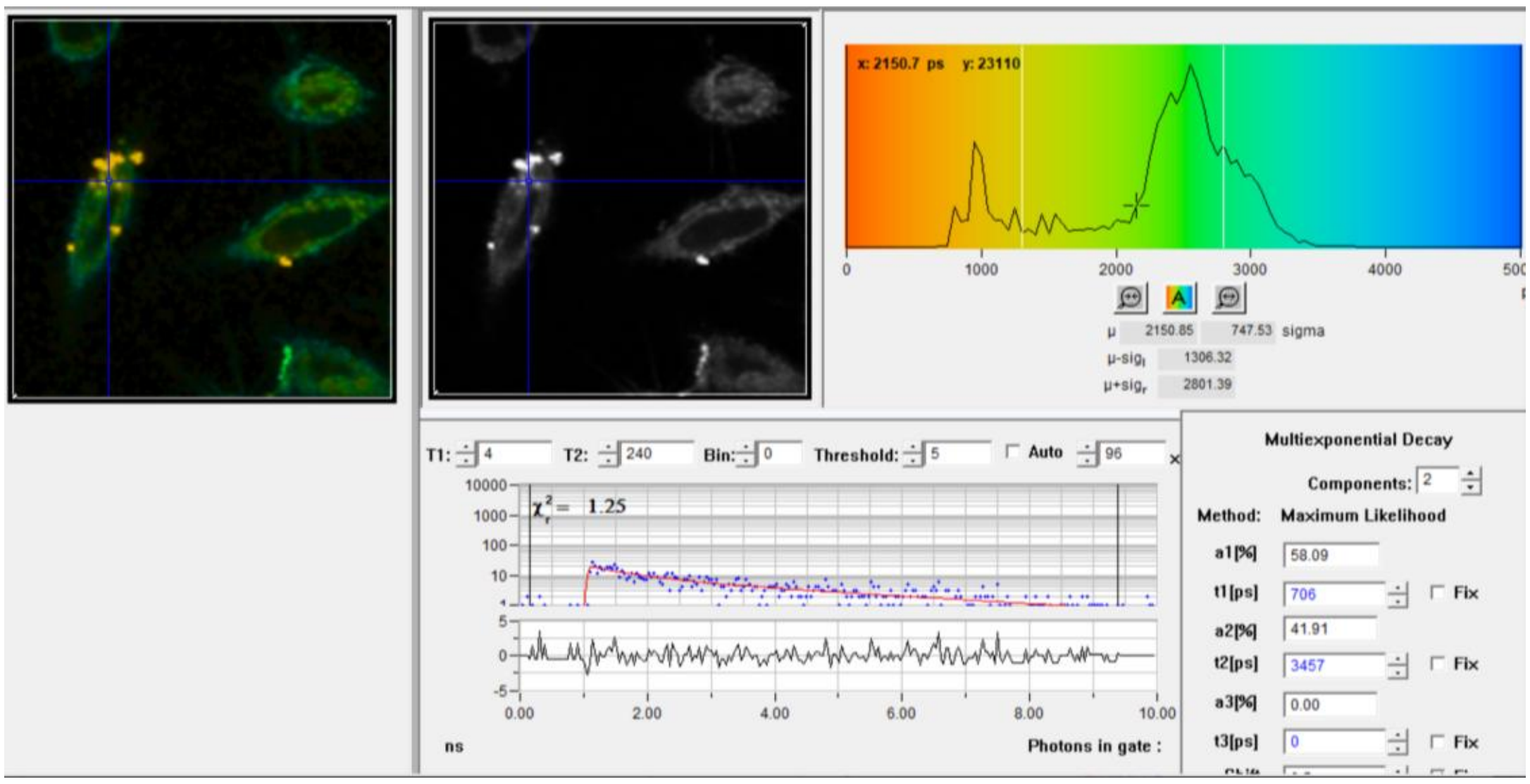

(b) Spot 2

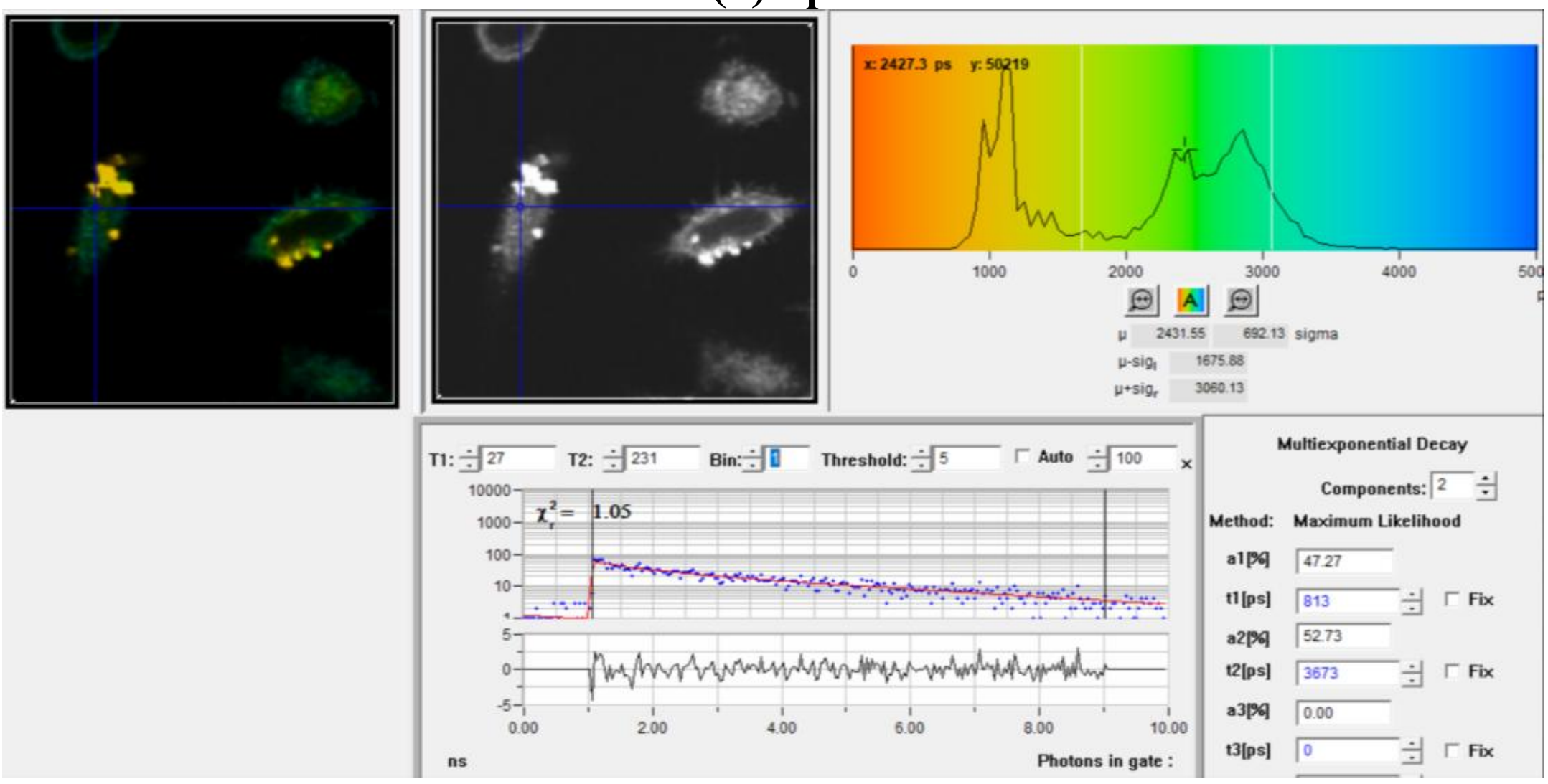

(c) Spot 3

Figure S7.41. Alternative view: 2P FLIM of compound **5** in 2% DMSO in HeLa, 15 min incubation at 37 °C, 810 nm and 4.3 mW power (clockwise: lifetime distribution map, intensity image, and FLIM parameters including TCSPC in three random spots in the cytoplasm). Field of view: 100 µm, Lifetime scale bars: τ_m=0-5000 ps

Compound 6 2P FLIM at 810 nm

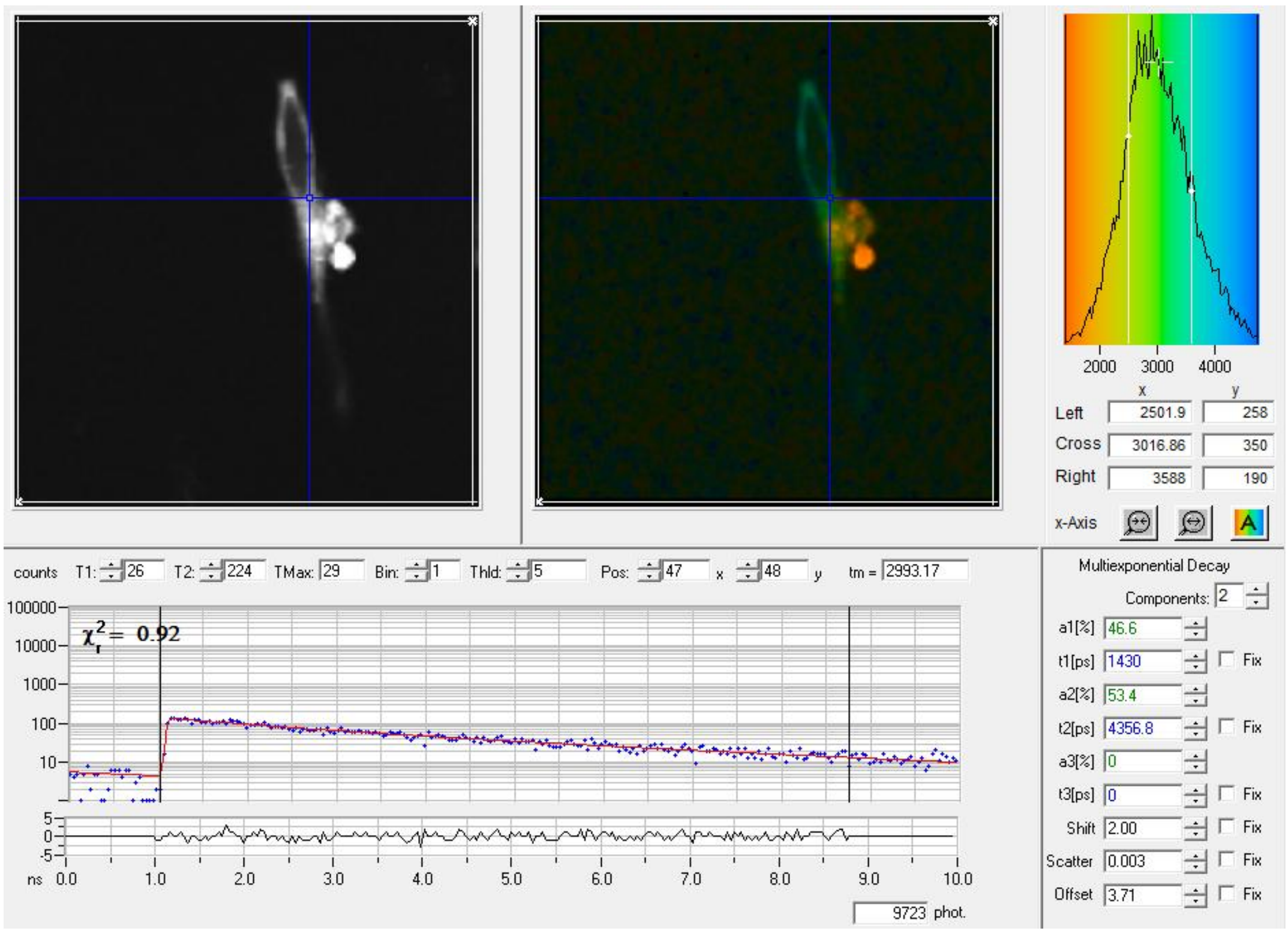

Figure S7.42. Overview of 2P FLIM experiment of compound **6** in HeLa, 37 ºC, 28 h incubation, 810 nm, 8.7 mW laser power (clockwise: intensity image, lifetime distribution map and parameters including TCSPC in a random spot in cytoplasm). Blebbing indicative of cell membrane damage was observed upon 2P-irradiation at 810 nm only. Scale bar: τ_m=1000-5000 ps.

Compound 6 2P FLIM at 910 nm

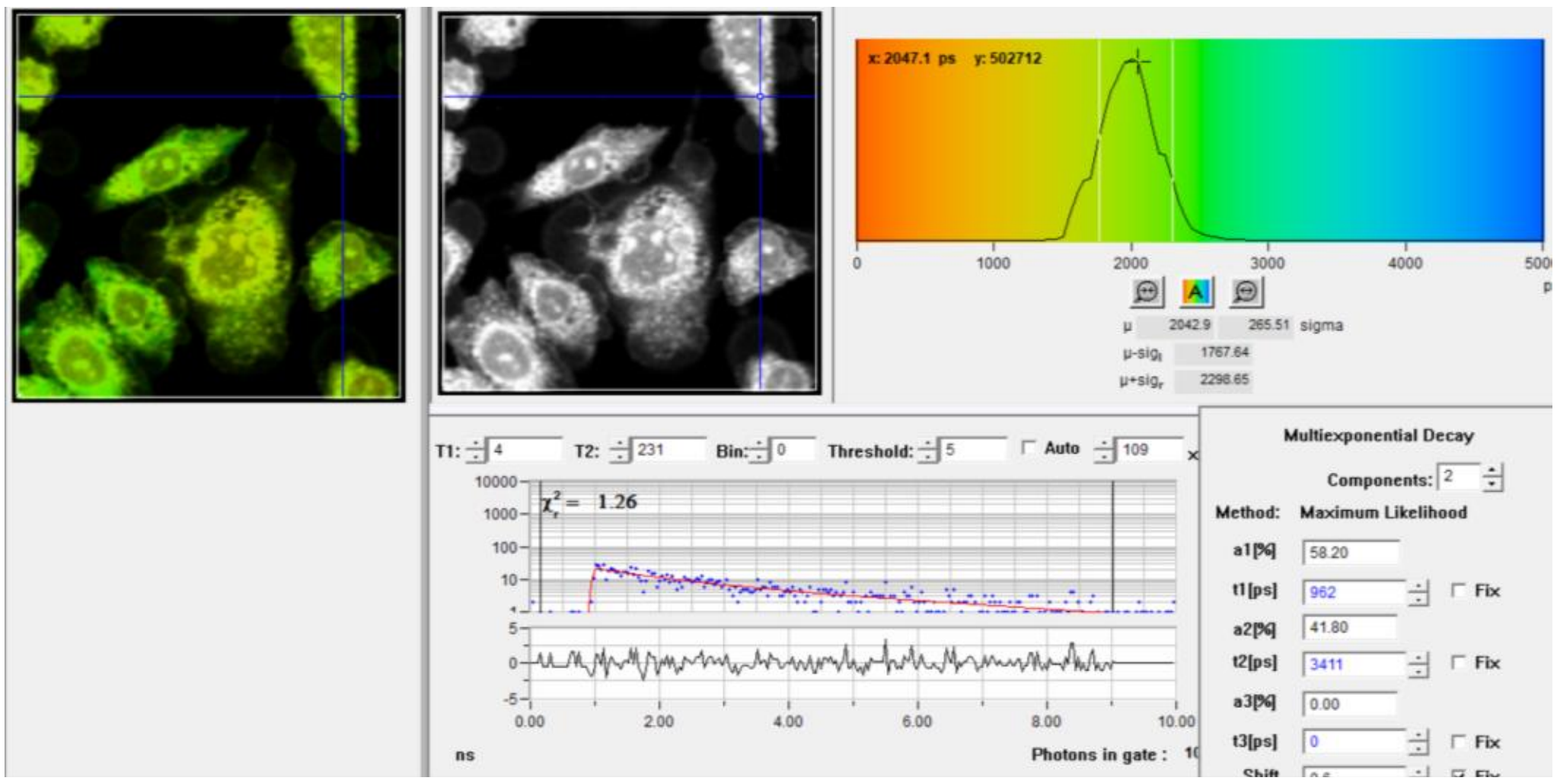

(A) Spot 1

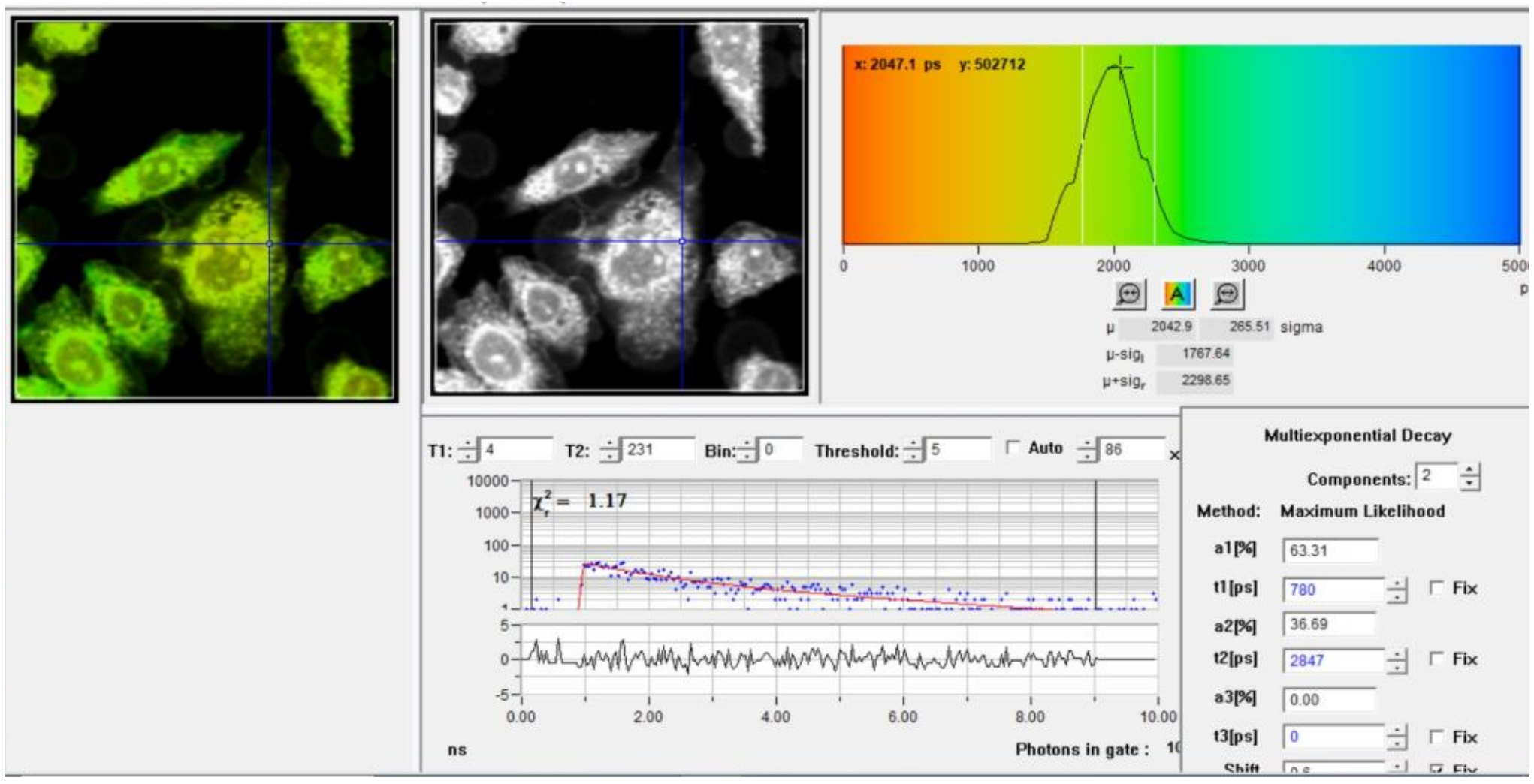

(B) Spot 2

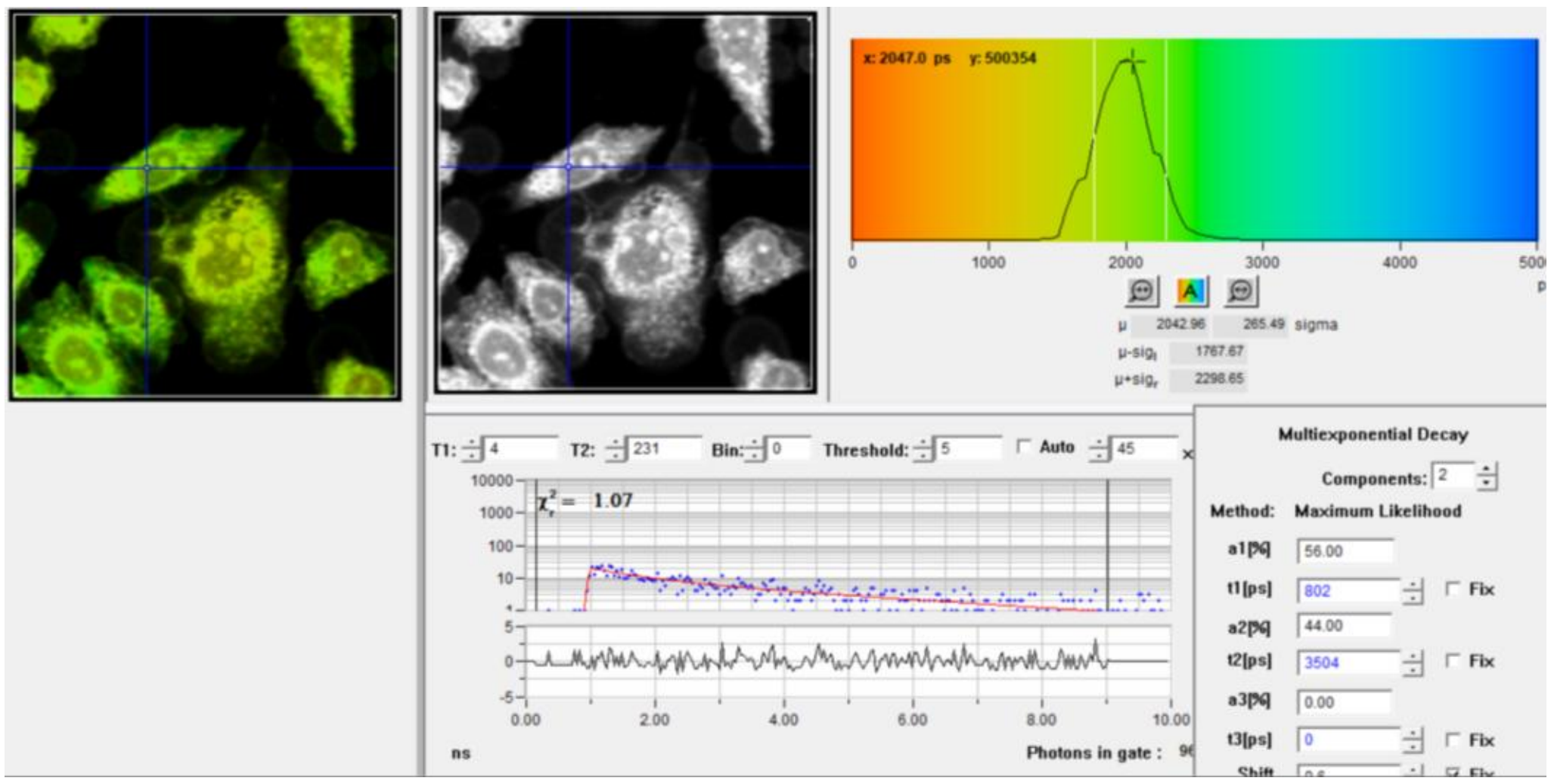

(C) Spot 3

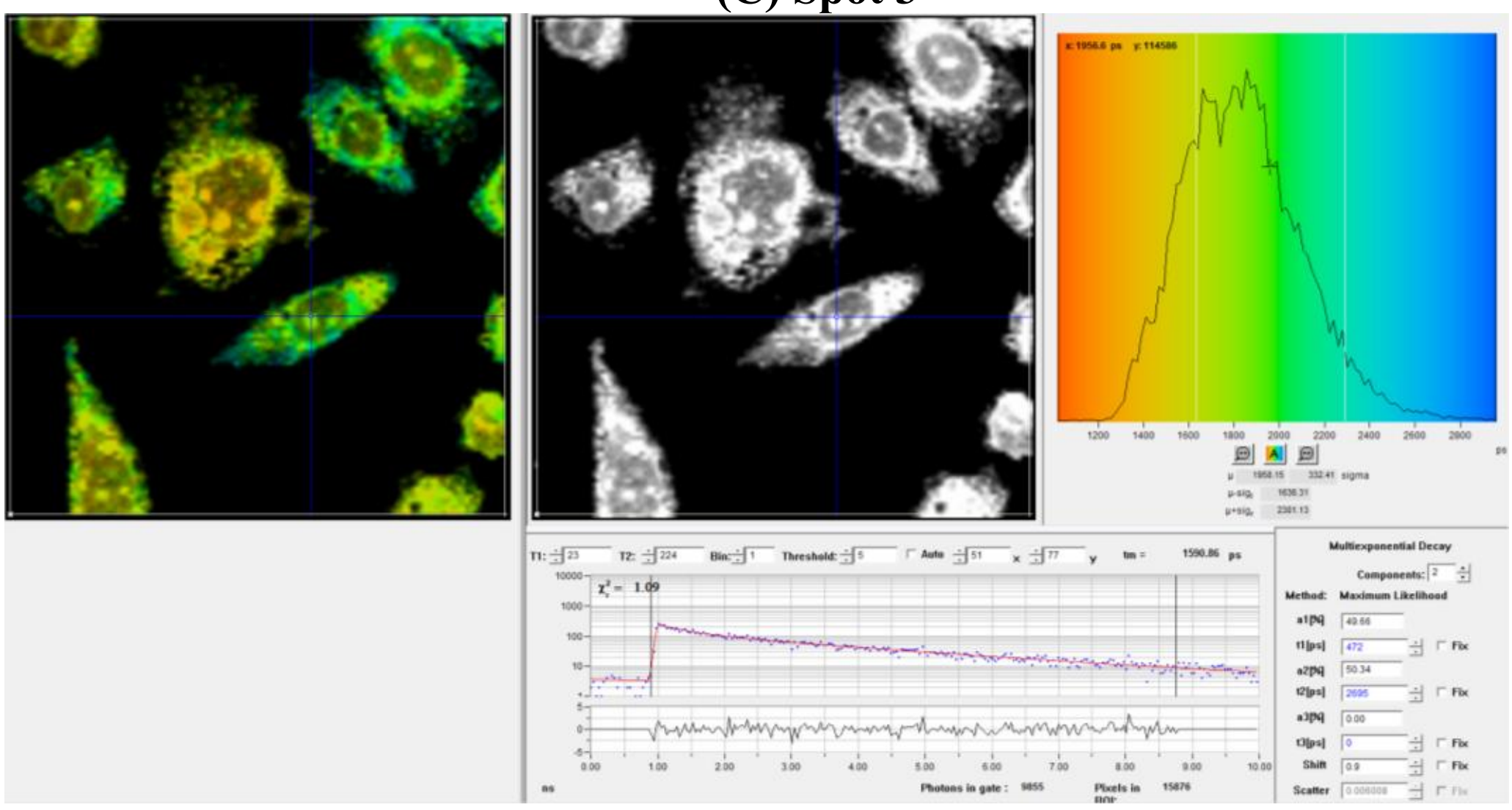

(D) Spot 4

Figure S7.43. 2P FLIM of compound **6**, 100 μm in 1% DMSO in PC-3, 15 min incubation at 37 ºC, 910 nm, (clockwise: intensity image, lifetime distribution map and

parameters including TCSPC in random spots in cellular environments). Field of view: 100 µm, Lifetime scale bars: τ_m=0-5000 ps (A-C) and 1100-2900 ps (D).

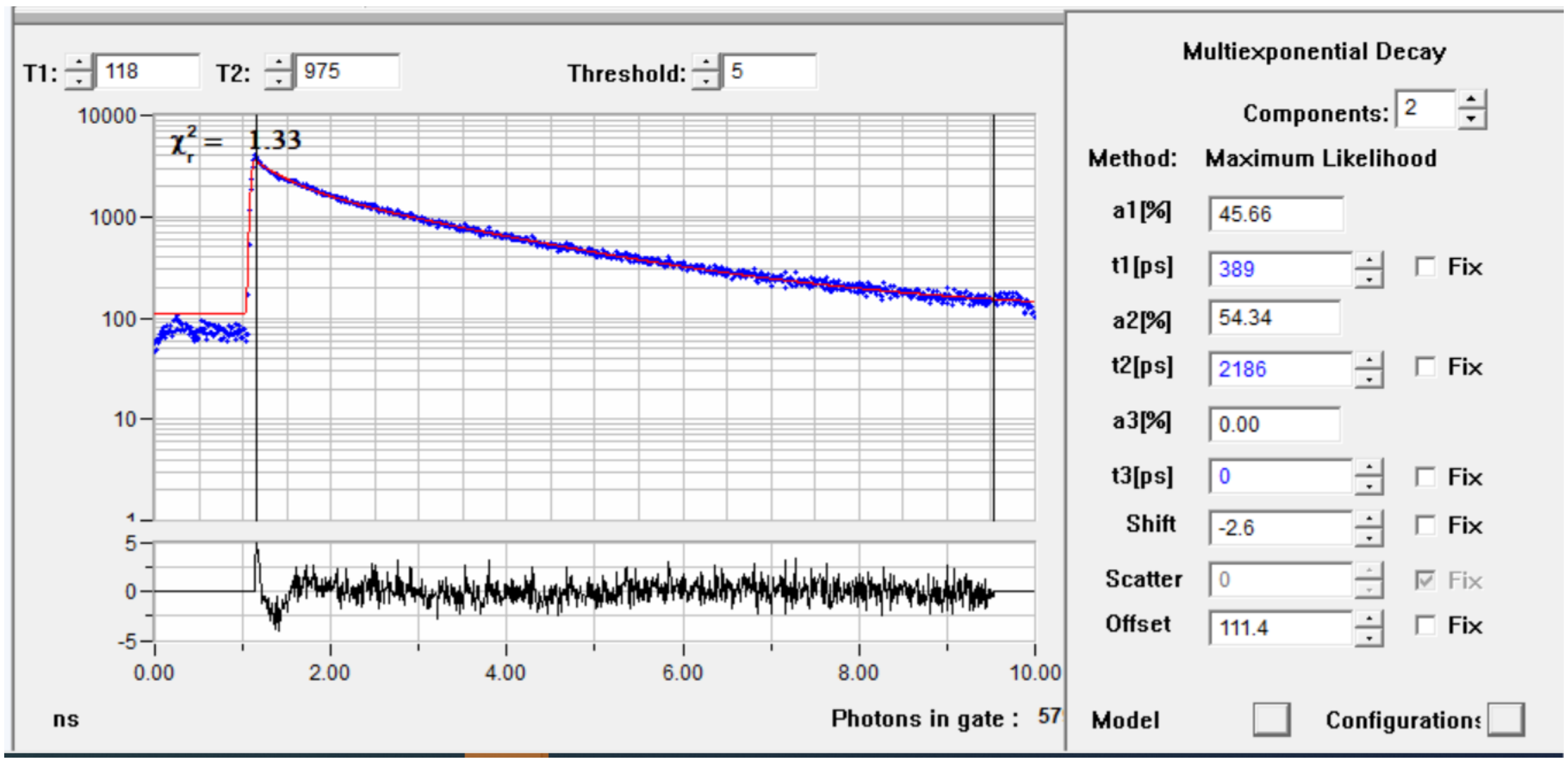

(A)

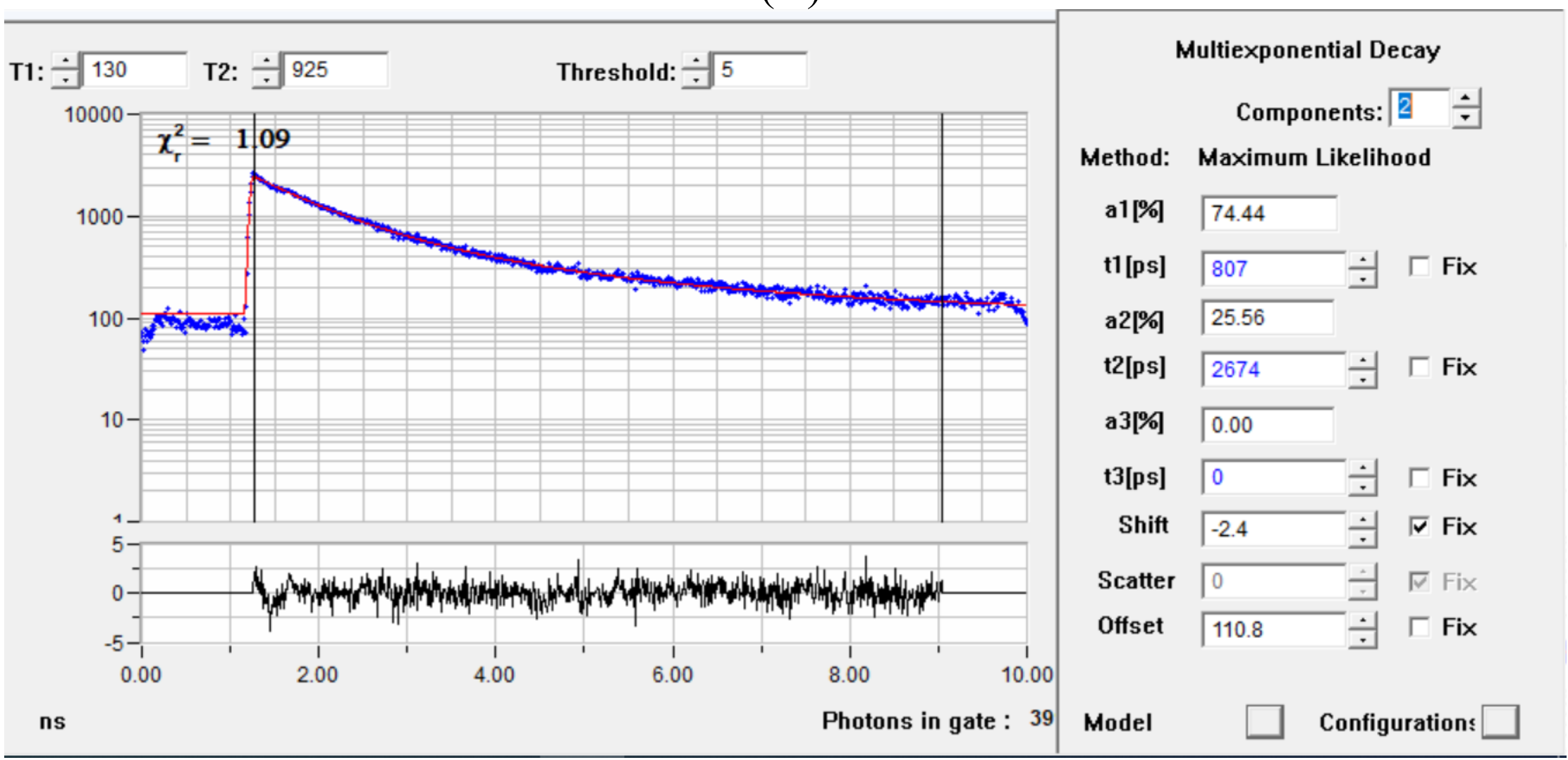

(B)

Figure S7.44. 2P TCSPC of Compound **6** in solution, 1 mM in DMSO (A) and 10 mM in DMSO (B), excitation 910 nm.

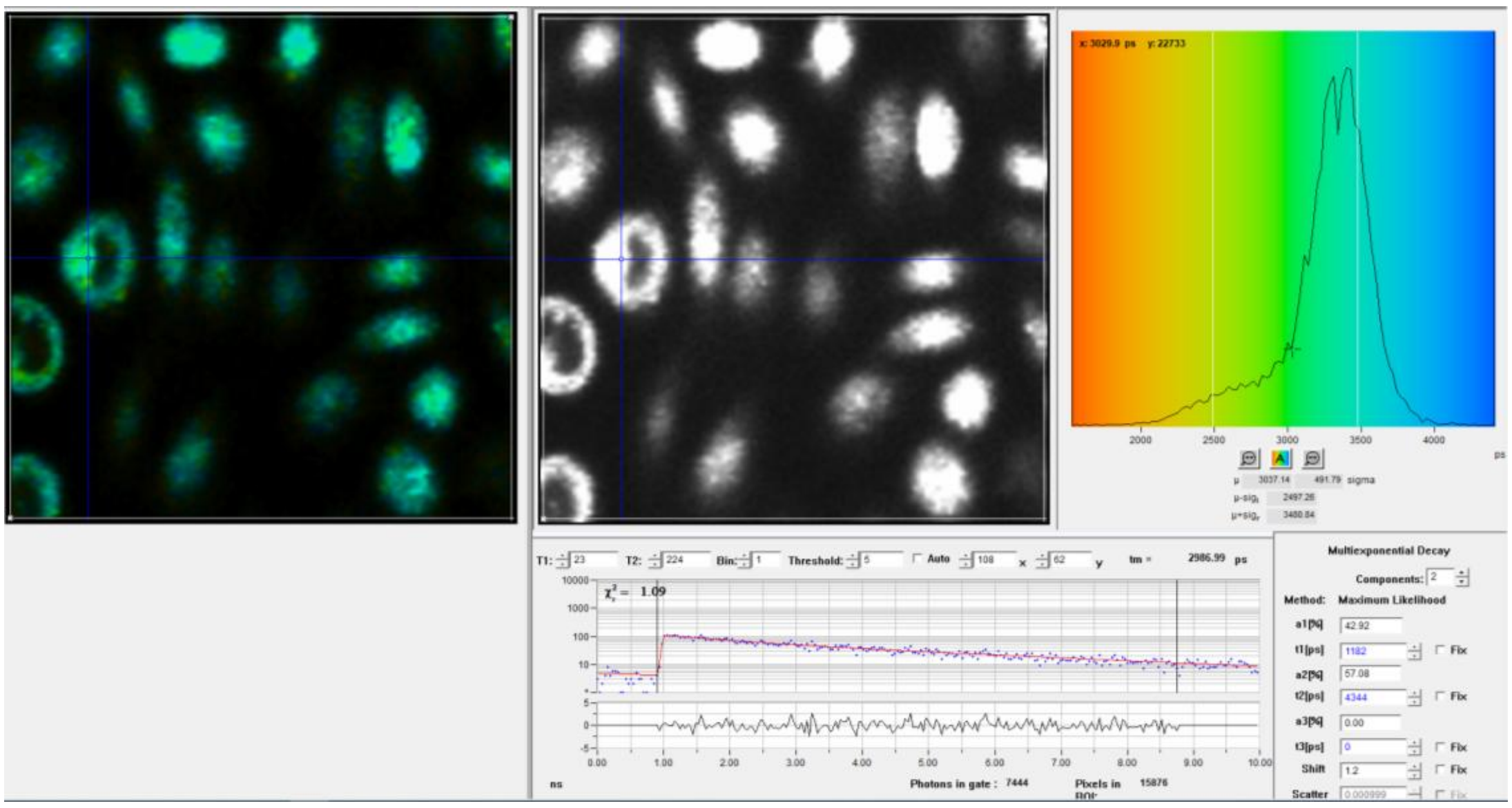

(A) Spot 1

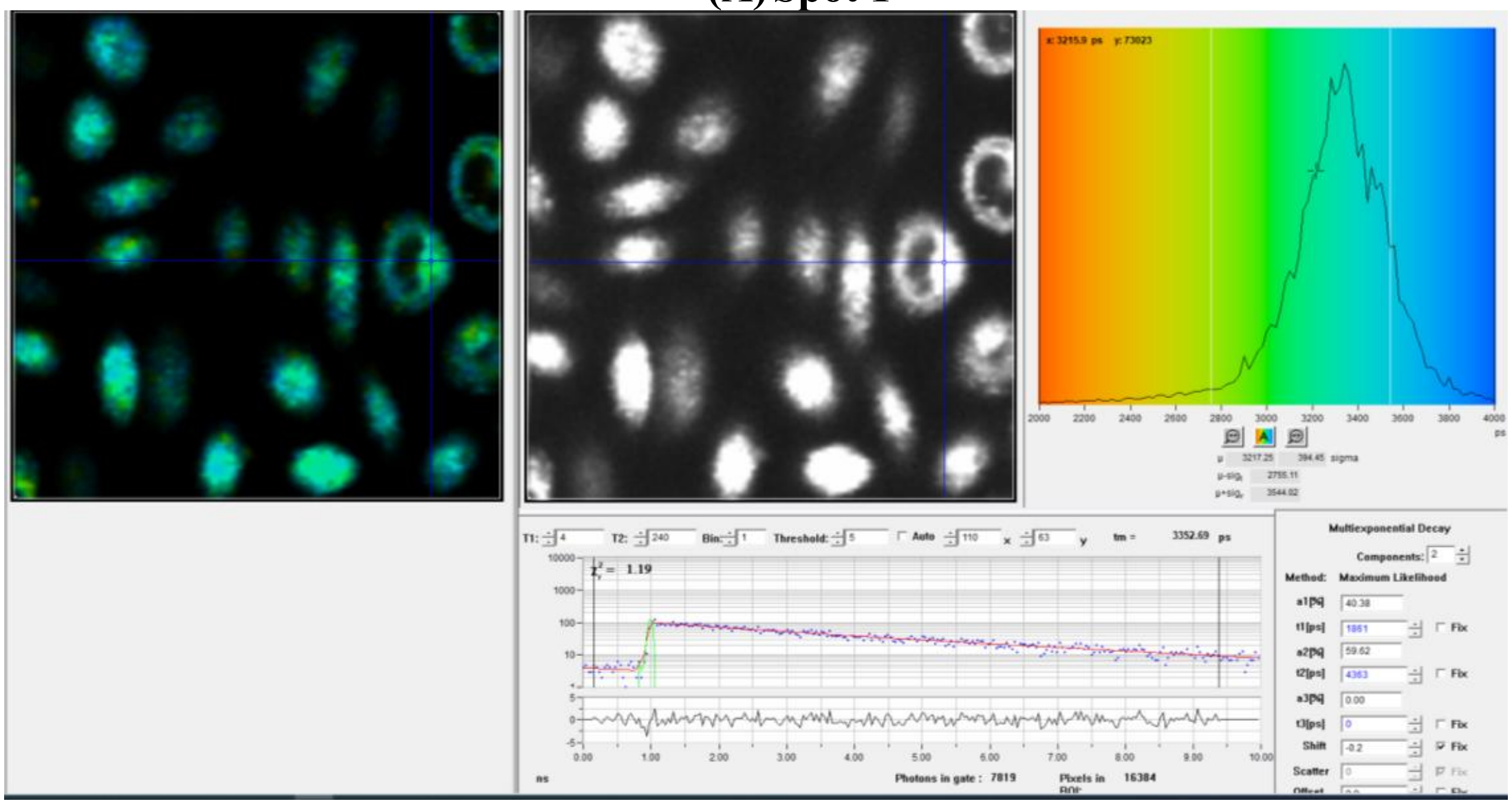

(B) Spot 2

Figure S7.45. 2P FLIM of compound **6** in 100 µm in 1% DMSO in CHO, 60 min incubation at 37 °C, 910 nm, 7 mW laser power (clockwise: lifetime distribution map, intensity image and parameters including TCSPC in random spots in cytoplasm). 100 µm field of view, Lifetime scale bar: τ_m=1500-5000 ps (A) and 2000-4000 ps (B).

Colocalisation assays by 2P FLIM for Compound 6

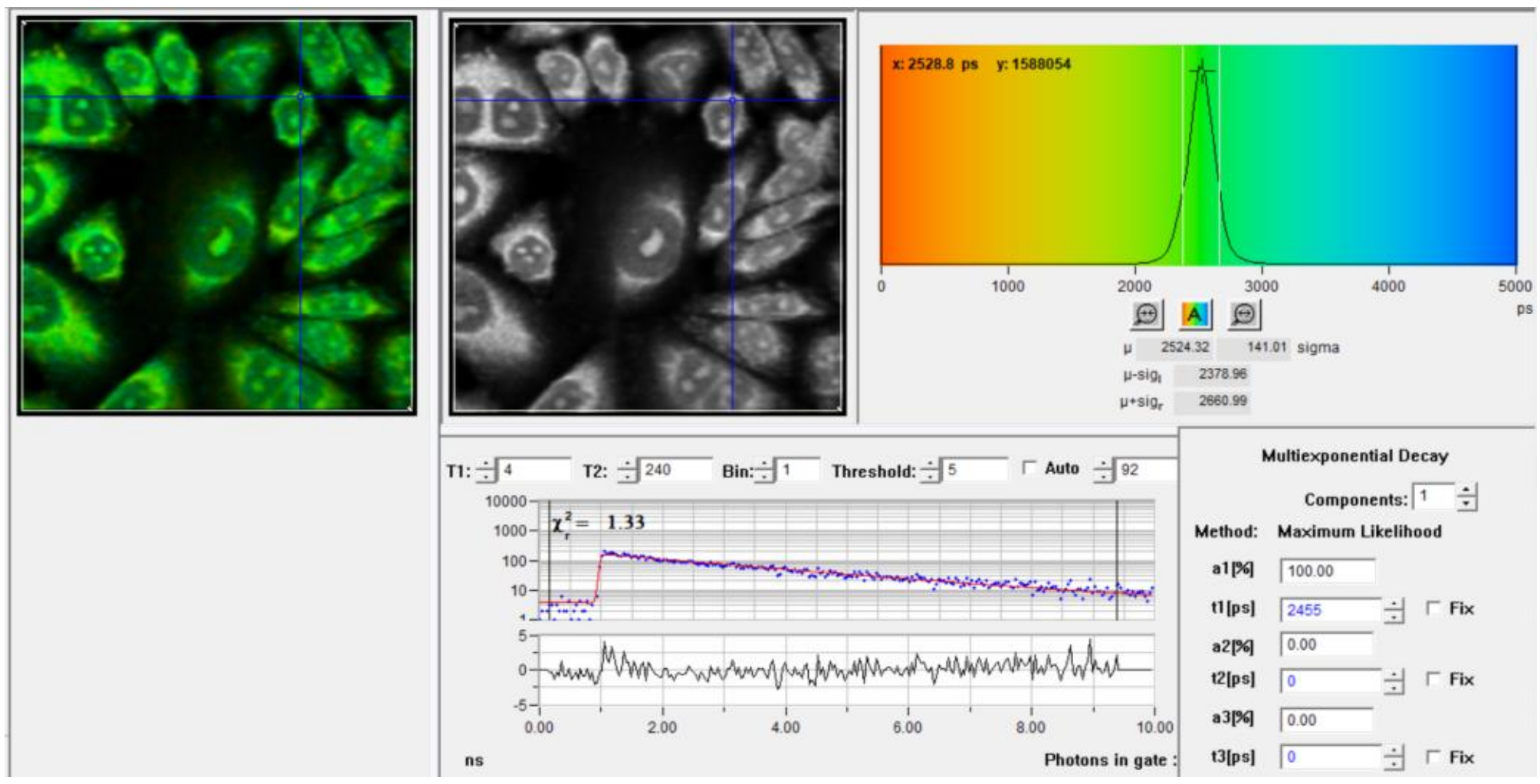

(A) Spot 1

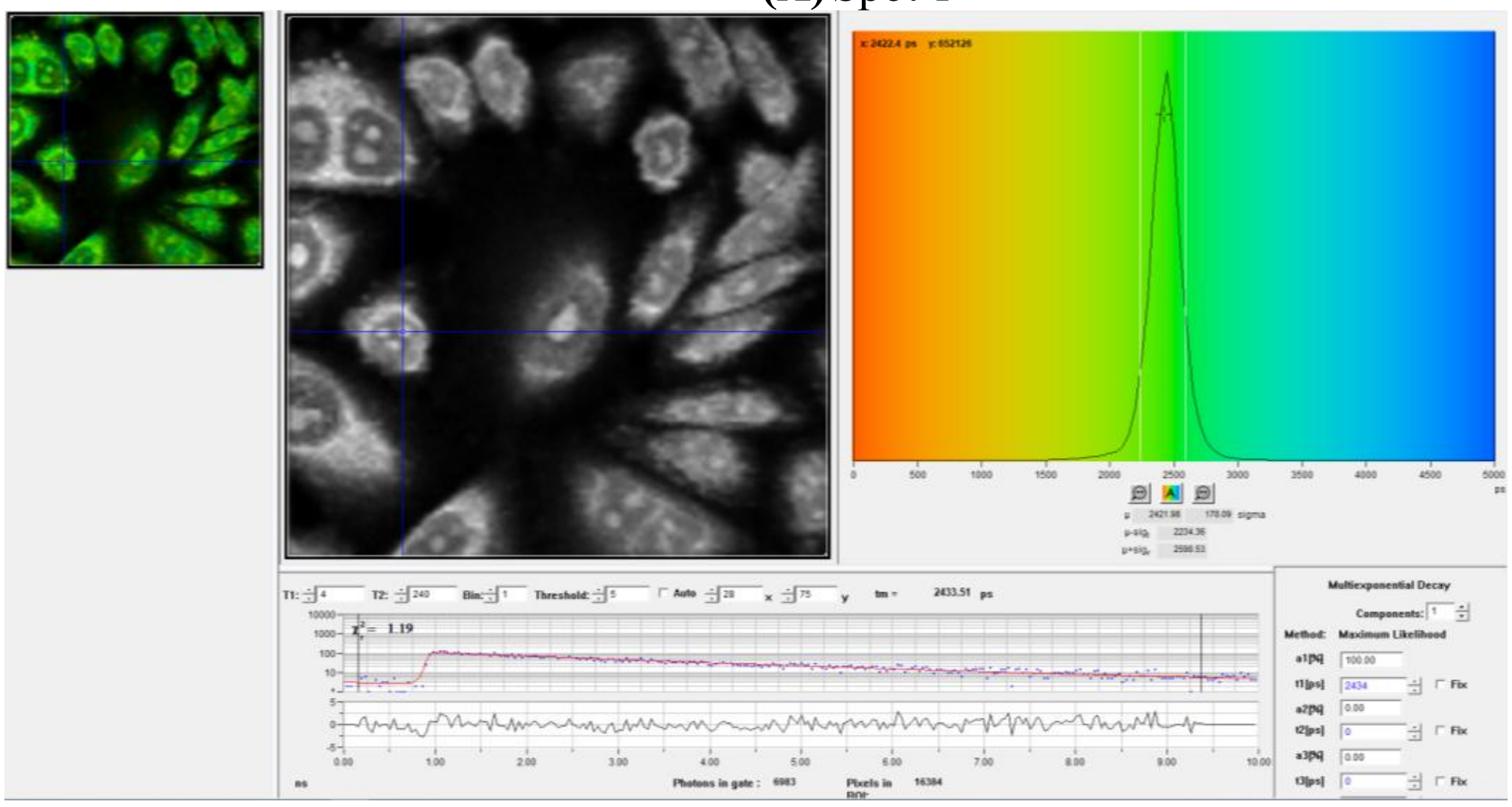

(B) Spot 2

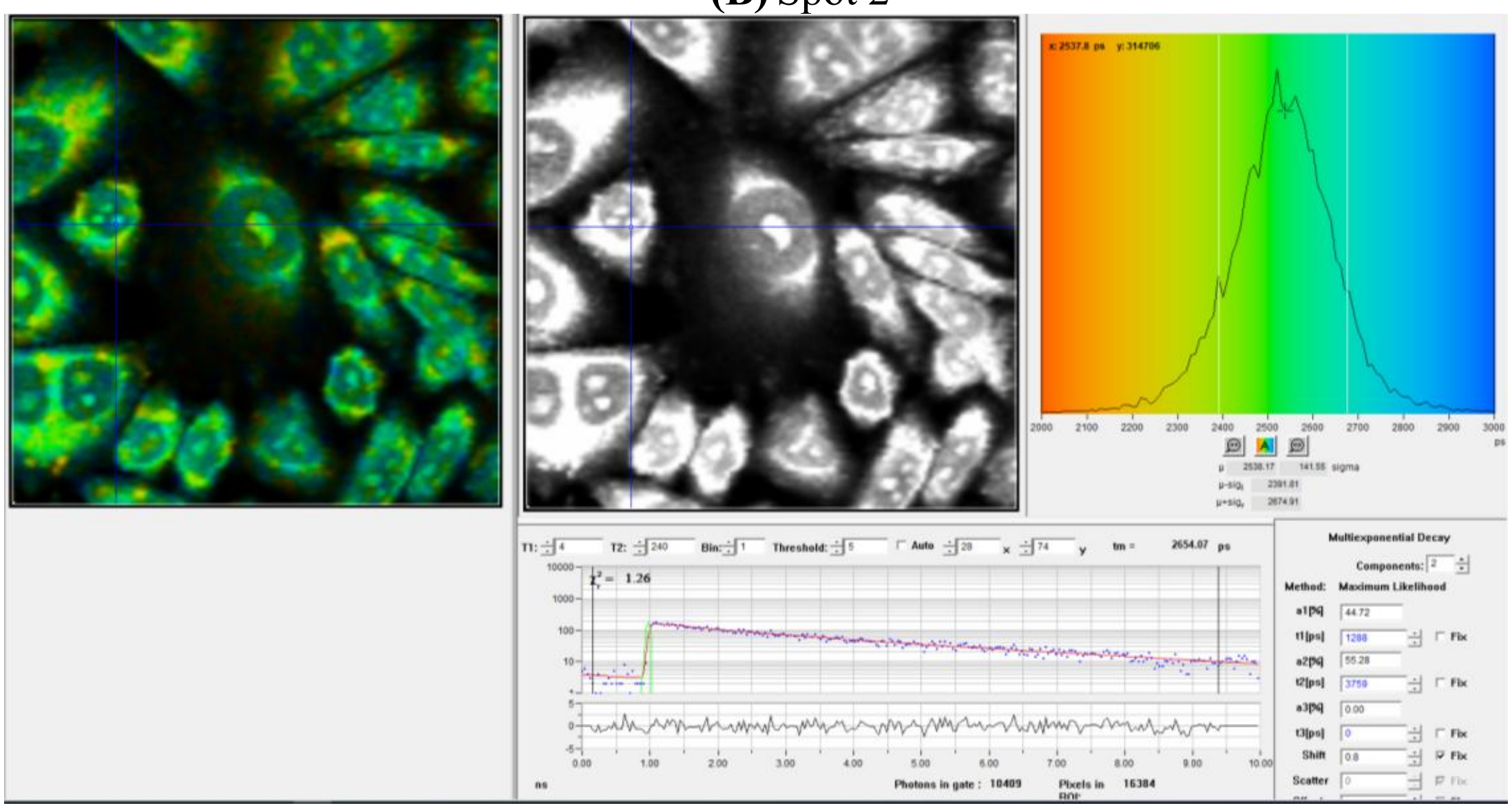

(C) Spot 3

Figure S7.46. 2P FLIM of compound **6**, 10 μM in 0.1% DMSO in CHO cells co-stained with AlexaFluor and Hoechst, 60 min incubation at 37 ºC, 870 nm, 2.0 mW power, (clockwise: lifetime distribution map, intensity image and parameters including TCSPC in random spots in the cellular environment). 100 μm field of view, Lifetime scale bar: τ_m=0-3000 ps (A-B) or 2000-3000 ps (C).

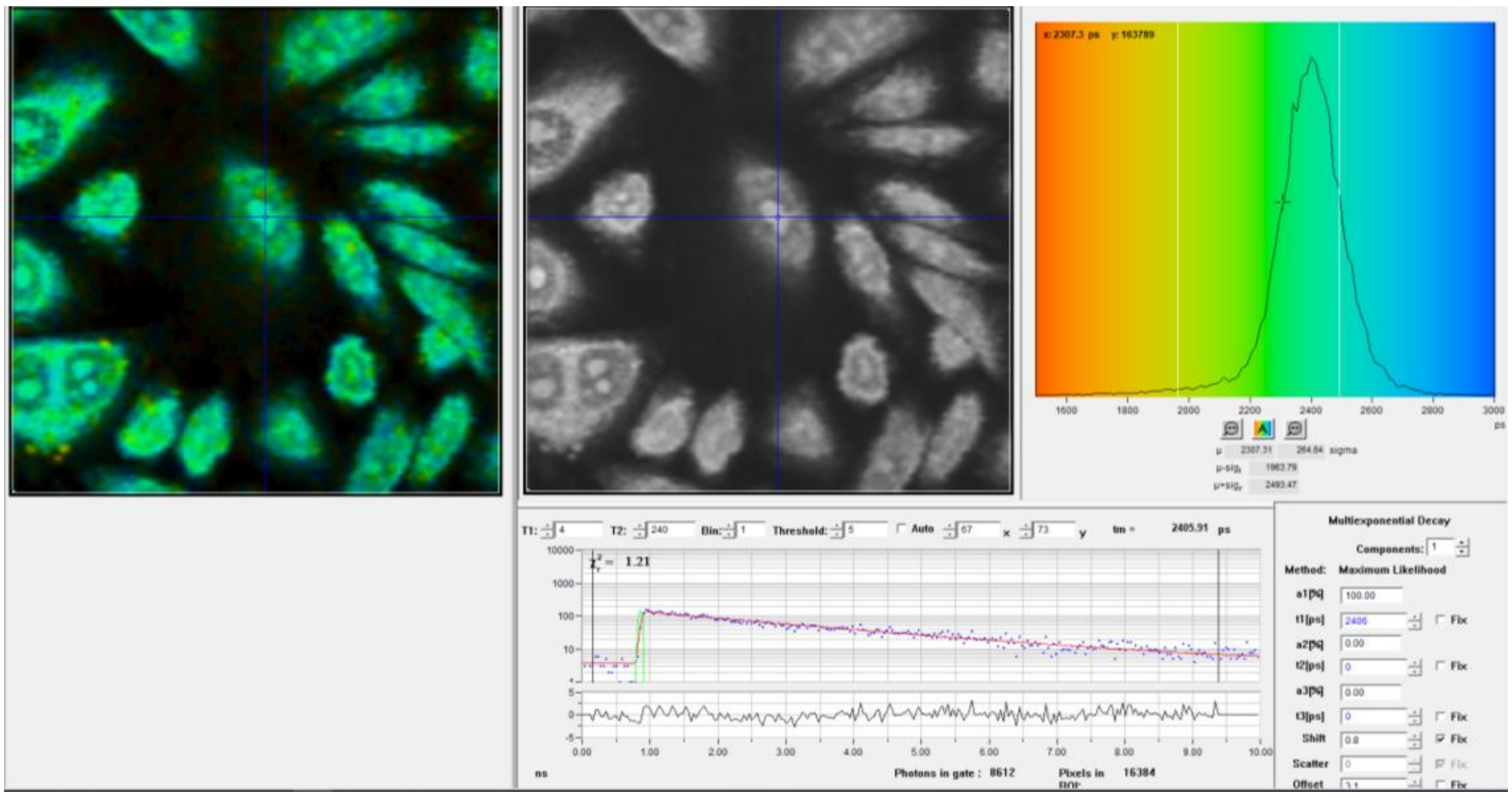

Figure S7.47. 2P FLIM of compound **6**, 10 μM in 0.1% DMSO in CHO, co-stained with AlexaFluor and Hoechst, 60 min incubation at 37 ºC, 830 nm, 7.0 mW power, (clockwise: lifetime distribution map, intensity image and parameters including TCSPC in a random spot in nucleus). 100 μm field of view, Lifetime scale bar: τ_m=1500-3000 ps.

8. Fluorescence titrations

Stock solutions of "receptor" compounds **1**, **5** and **6** of 10 mM were made by dissolving fluorophores in pH 8.21 aqueous buffer. 50 mL working solutions were prepared by diluting the stock solution with pH 8.21 aqueous methanolic buffer.
β-D-glucan was completely dissolved in pH 8.21 aqueous buffer by heating (50 °C) generated 1 mg/mL "guest" solution. Increasing volumes of the guest were transferred to a 1 mL receptor solution in a fluorescent spectroscopy cuvette and were left for 5 minutes to allow complete guest and receptor binding. Next, the same volume of the mixture was removed maintaining the total volume unchanged after each titration. Fluorescence emission spectra were recorded after each titration. The observed stability constants (K) with a coefficient of determination (r^2) were calculated by the fitting emission intensity at a single wavelength versus polysaccharide concentration using custom written non-linear curve fitting (equation 1) within the Origin 9.0 software. The binding constant (K) was determined by fitting a 1:1 binding model to I_F/I_{Fmax}.

The same method was applied at the titration of Compound 2 with D-glucose.

$$y = (1 + A_1 * A_2 * x) / (1 + A_1 * x) \quad (1)$$

y: I_F/I_{Fmax}
A_1: K (binding constant of the receptor with the guest)
A_2: I_{Fmin}/I_{Fmax} x: the concentration of β-D-glucan added to a fluorophore
I_{Fmin} is the final (minimum) fluorescence intensity;
I_{Fmax} is the initial (maximum) fluorescence intensity;
I_F is the fluorescence intensity after addition of particular β-D-glucan (guest) concentration.

Compound@glucan preparation:
Compound (10 mM, 1 mL) in DMSO was add to β-D-glucan (1 mg/mL, 1 mL) in DMSO, the mixture was stirred at room temperature overnight at pH 7.4. The vial was protected from light.

Alternative Method for the Fluorescence Titration of Boronic Acid Fluorophores with β-D-Glucan
Fluorescence titrations for Compounds 1, 5 and 6 were performed using this boronic acid-functionalised fluorophores to probe the general applicability of the boronic acid–glucan binding approach. Stock solutions of the fluorophore ("receptor") with known molarity were prepared by dissolving the fluorescent boronic acid in aqueous buffer (pH 8.21). Working solutions were then made by diluting each stock in pH 8.21 aqueous methanolic buffer to 25 µM of the corresponding fluorophore.

β-D-Glucan (denoted the "guest") was dissolved completely in pH 8.21 aqueous buffer by heating to 50 °C to obtain a 0.031 mM solution. Aliquots of the guest solution were incrementally added to 1.0 mL of each receptor solution in a fluorescence spectroscopy cuvette. After each addition, the mixture was allowed to equilibrate for 5 minutes to ensure complete receptor–guest binding. An equivalent volume was then withdrawn to maintain a constant total volume across all titration points.

Fluorescence emission spectra were recorded by exciting solution of compound 1 was $\lambda ex = 460$ nm ($\lambda em = 543$ nm) and compounds 5 or 6 at $\lambda ex = 488$ nm ($\lambda em = 514$ nm).

Binding constants (K) and coefficients of determination (r^2) were calculated by fitting fluorescence intensity data (emission at a single wavelength) as a function of polysaccharide concentration. Non-linear curve fitting was performed using a custom script in Origin 9.1 software, applying a 1:1 receptor–guest binding model using the normalised intensity ratio I_F/I_{Fmax}

$$y = (1 + A_1 * A_2 * x) / (1 + A_1 * x)$$

Definitions for Fluorescence Titration Fitting Equation:

- **y** = I_F / I_{Fmax} : Normalised fluorescence intensity
- **A_1** = K : Binding constant of the receptor with the guest
- **A_2** = I_{Fmin}/I_{Fmax} : Ratio of minimum to maximum fluorescence intensity
- **x**: Concentration of β-D-glucan added (guest)
- **I_{Fmax}**: Initial (maximum) fluorescence intensity
- **I_{Fmin}**: Final (minimum) fluorescence intensity
- **I_F**: Observed fluorescence intensity after addition of a specific concentration of glucan

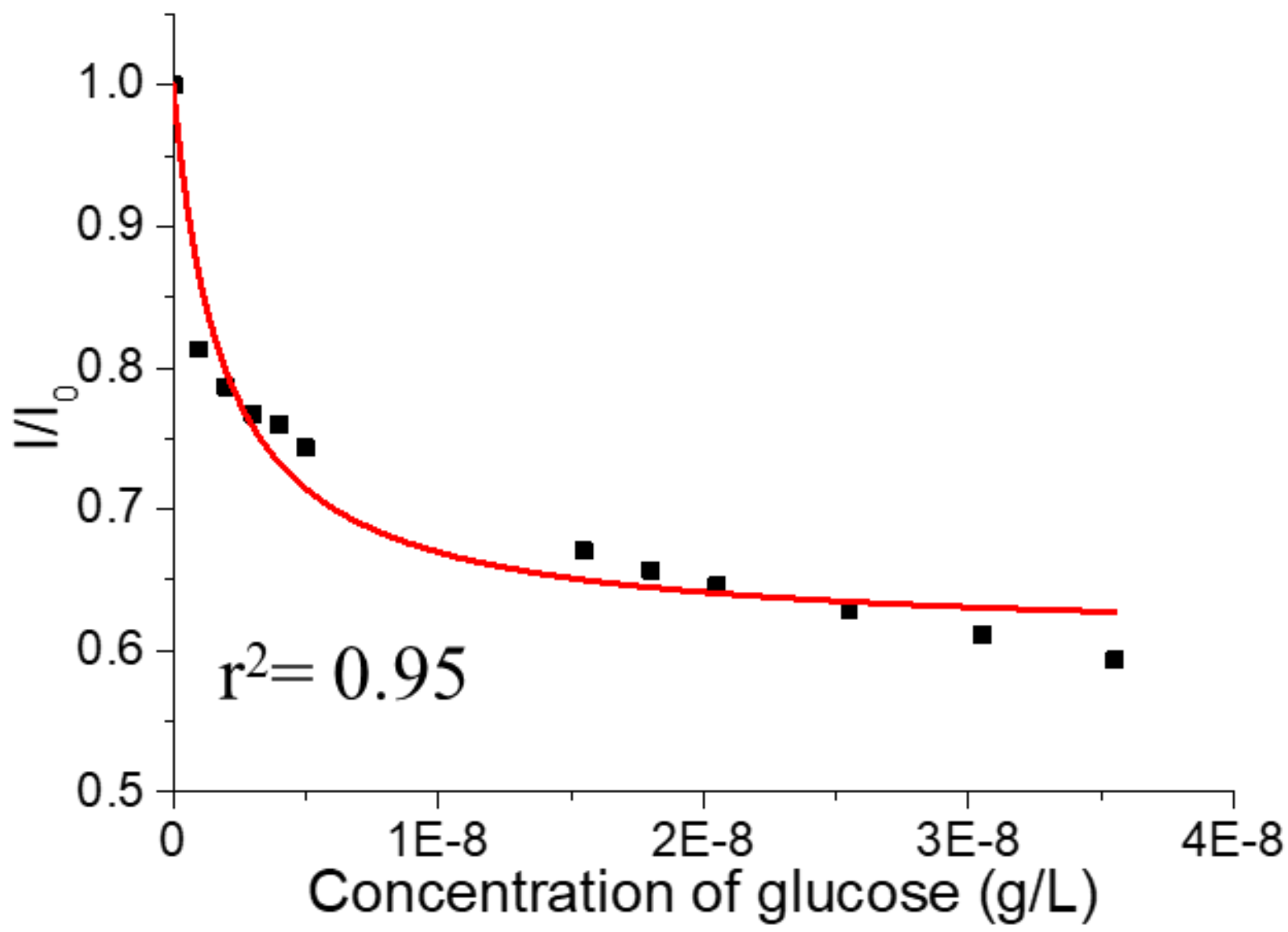

Figure S8.1. Titration of compound **2** with glucose, binding constant estimated was ca. 5.35×10^8 M^{-1}.

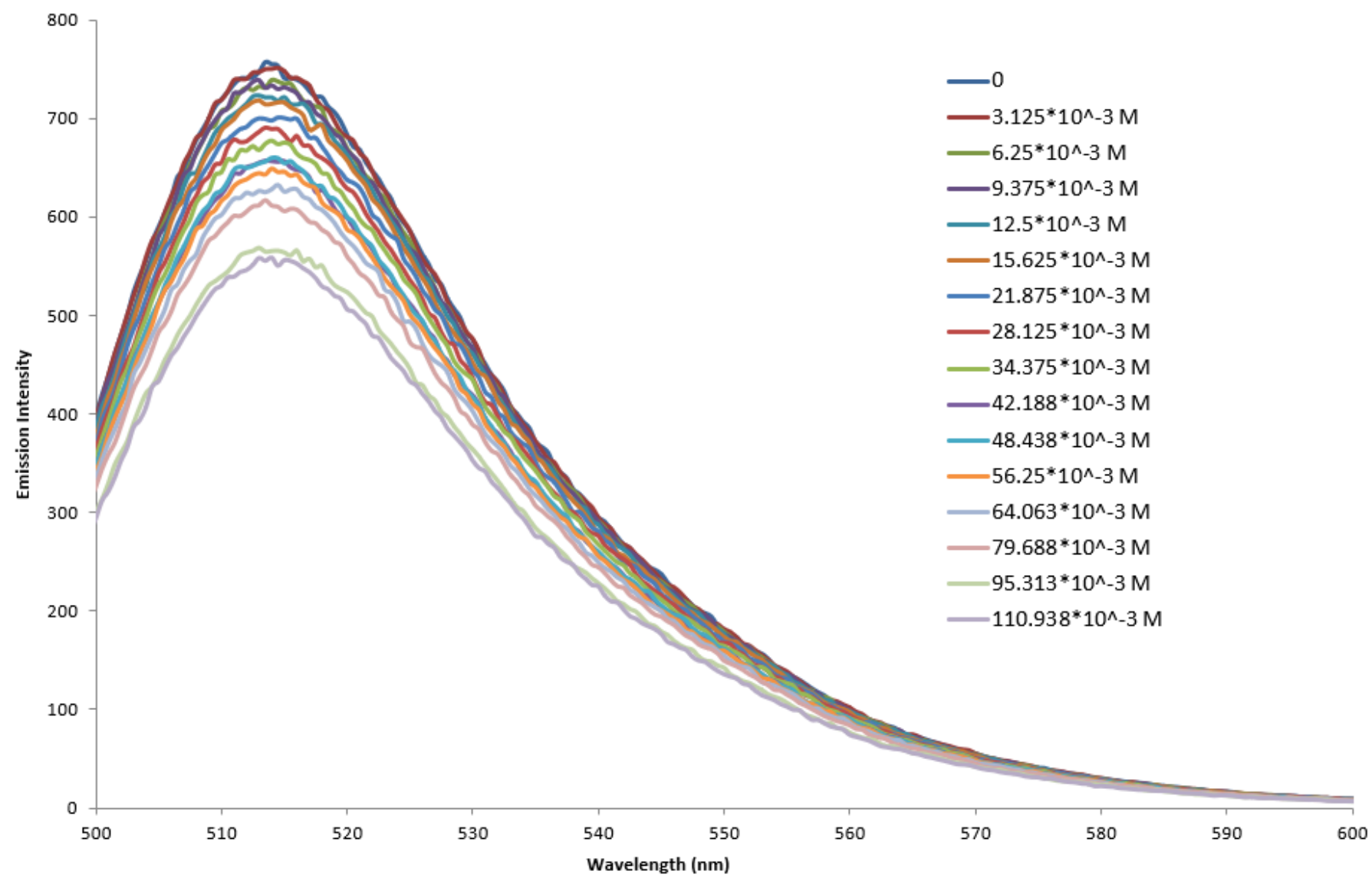

(a)

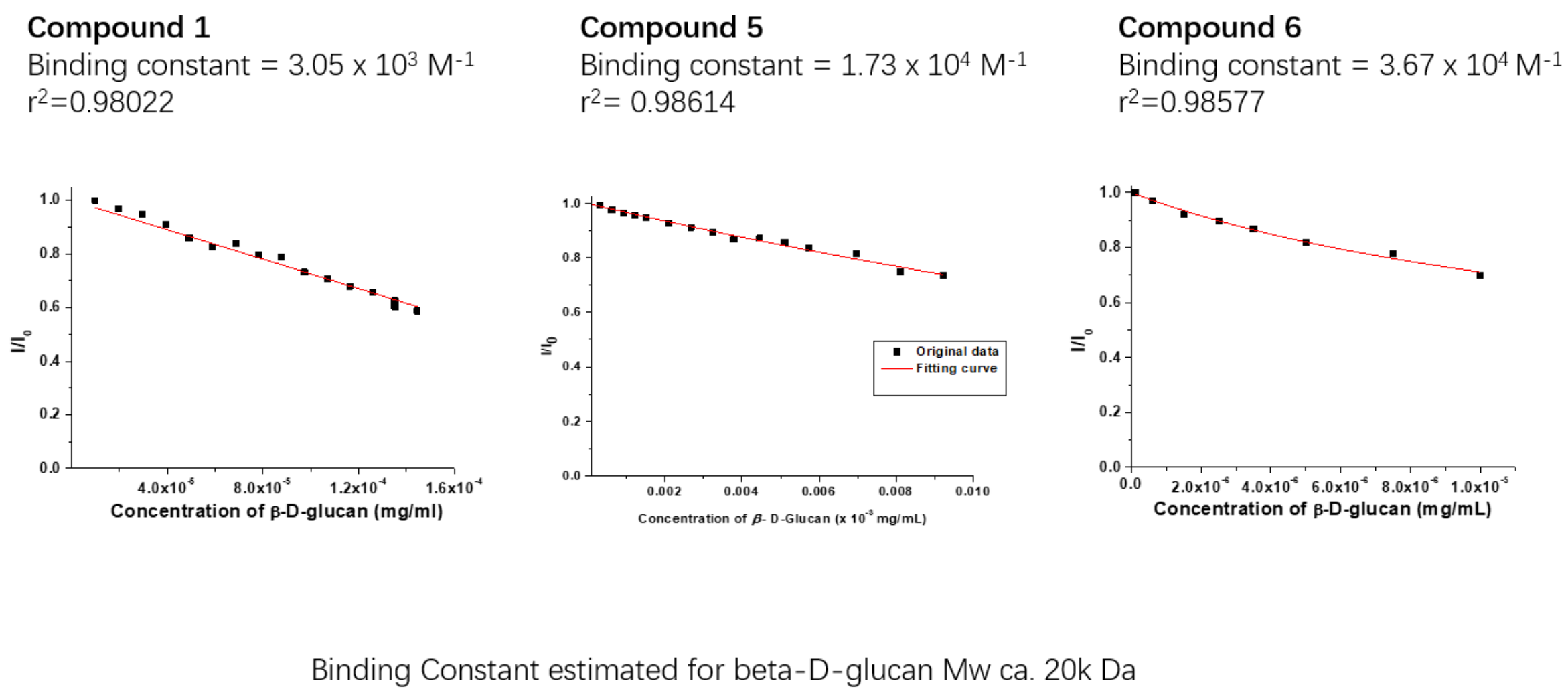

(b)

Figure S8.2. (a) Fluorescence emission decrease at the treatment of Compound 1 with beta-D-Glucan in pH 8.21 aqueous methanolic buffer. (b) Overview of titration fitted data for compounds **1, 5** and **6** with beta-D-glucan. Binding constant estimated based on an average Mw of beta-D-Glucan from Barley (Aldrich) of ca. 20 kDa.

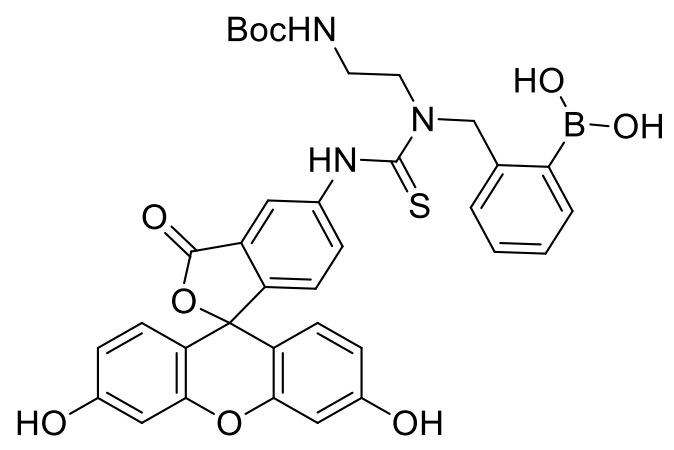

2

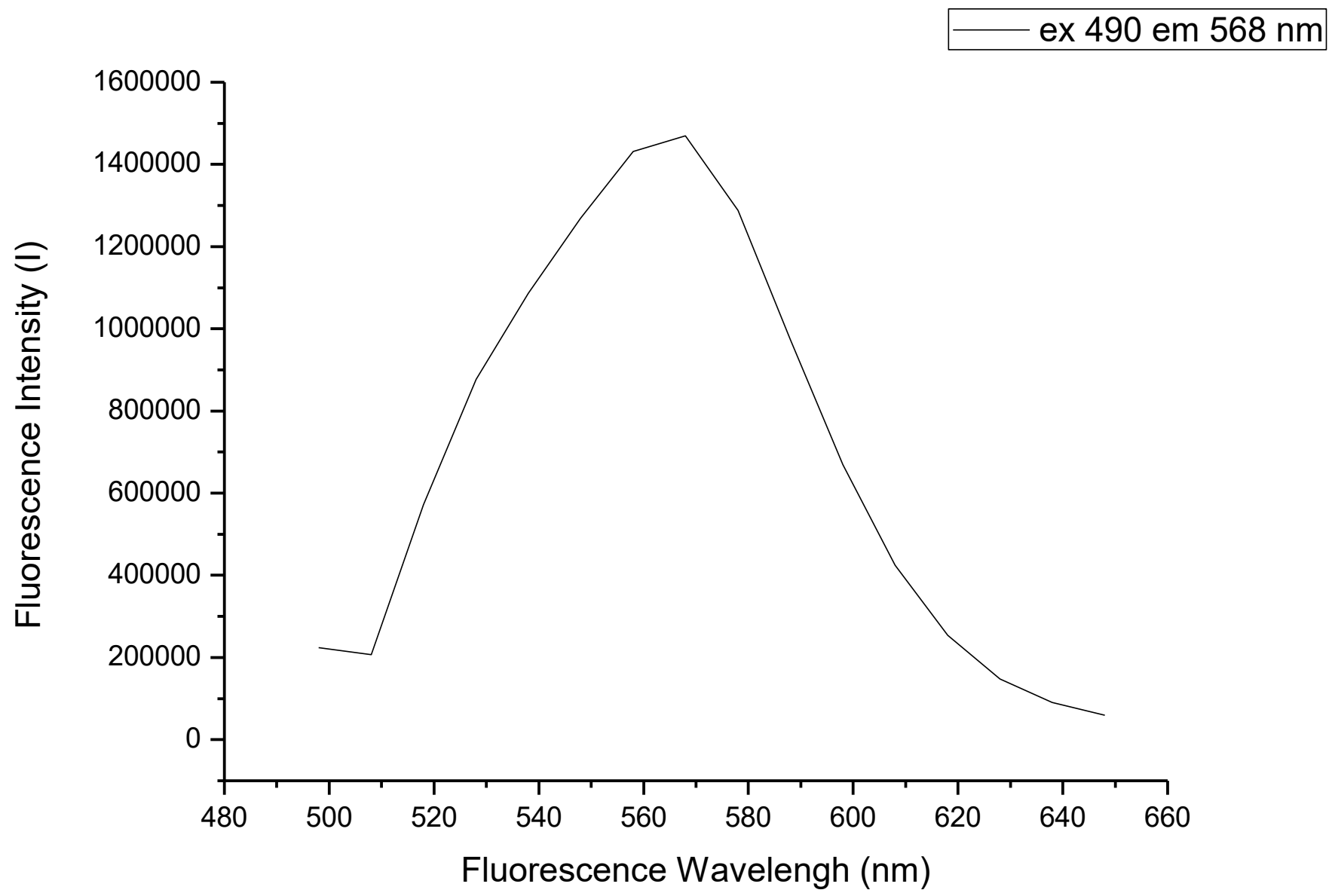

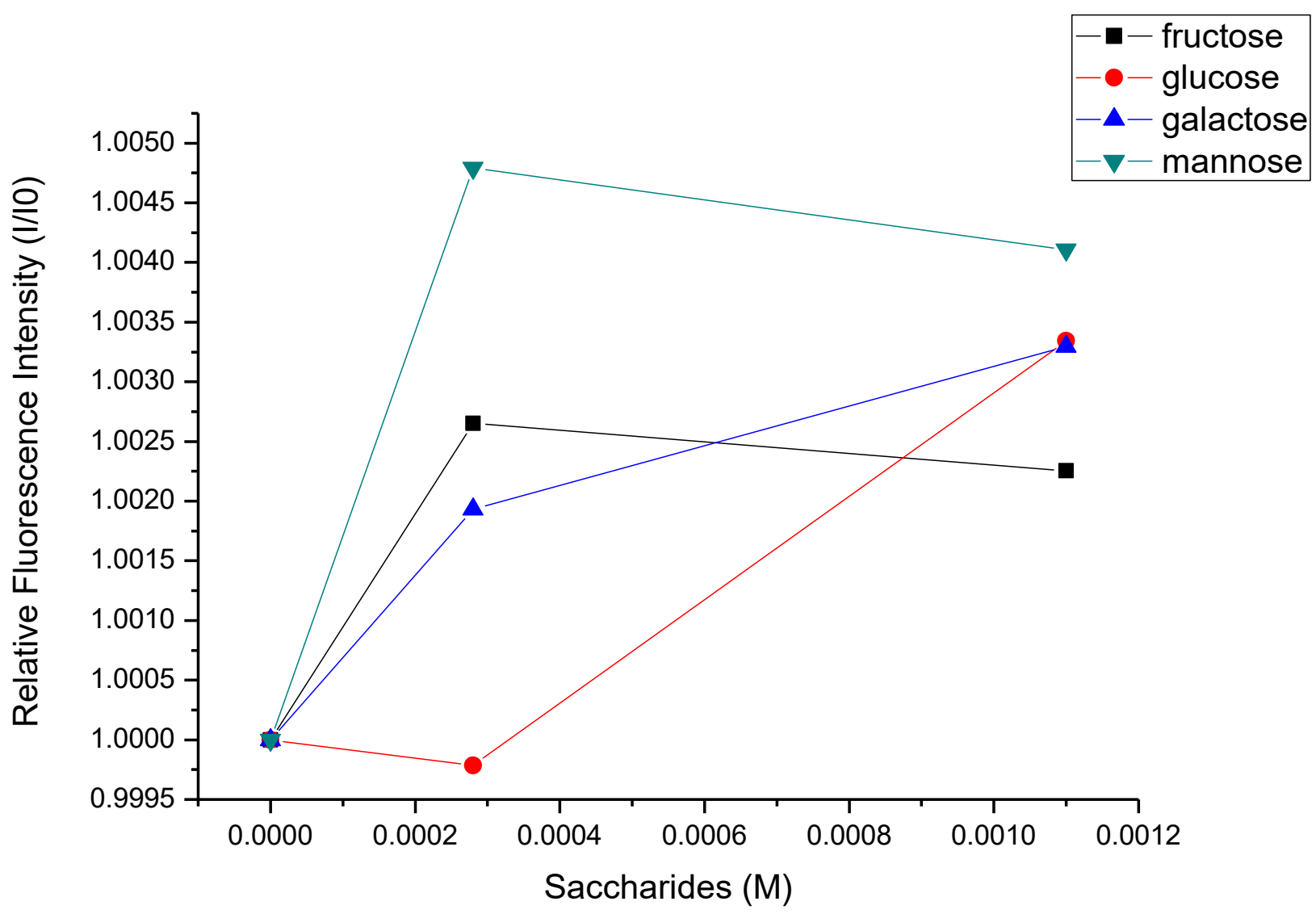

Figure S8.3. The fluorescence spectroscopy analysis of the boronic acid sensor **2** (λ_{ex} 490 nm, λ_{em} 568 nm) (top) and an overview of the fluorescence intensity of sensor **2** which increases upon the adding of saccharides (pH 8.21, aqueous methanolic buffer).

9. Avidin binding tests

<u>*Avidin binding test (fluorescence spectroscopy):*</u>

Commercially available avidin (2 mg) was dissolved into water (2 mL). Fluorescence intensity of samples 1-4 were measured.

Avidin (2 mg) was dissolved in 2 mL of water to prepare a 1 mg/mL stock solution. Compound 6 (3.87 mg, 4 mM) was dissolved in 1 mL of methanol and subsequently diluted 1:100 with water.
Compound 4 precursor (a precursor for Compound 6 without boronic acid; 3.01 mg, 4 mM) was similarly dissolved in 1 mL of methanol and diluted 1:100 with water.
Samples were prepared as follows:
Sample 1: 1 mL of 6 mixed with 1 mL of water.
Sample 2: 1 mL of 4 mixed with 1 mL of water.
Sample 3: 1 mL of 6 mixed with 1 mL of avidin solution (1 mg/mL in water).
Sample 4: 1 mL of 4 mixed with 1 mL of avidin solution (1 mg/mL in water).

Subtle colour changes were visually observed and recorded before and after the addition of avidin. UV-visible and fluorescence spectra were measured for all samples. Results showed that both 6 and its precursor 4 exhibited similar colour and absorption bands; however, **6** displayed significantly higher fluorescence intensity than the precursor lacking boronic acid. Upon addition of avidin, both colour absorption and fluorescence intensity decreased markedly for both compounds, indicating that binding to avidin causes quenching of fluorescence for these fluorophores.

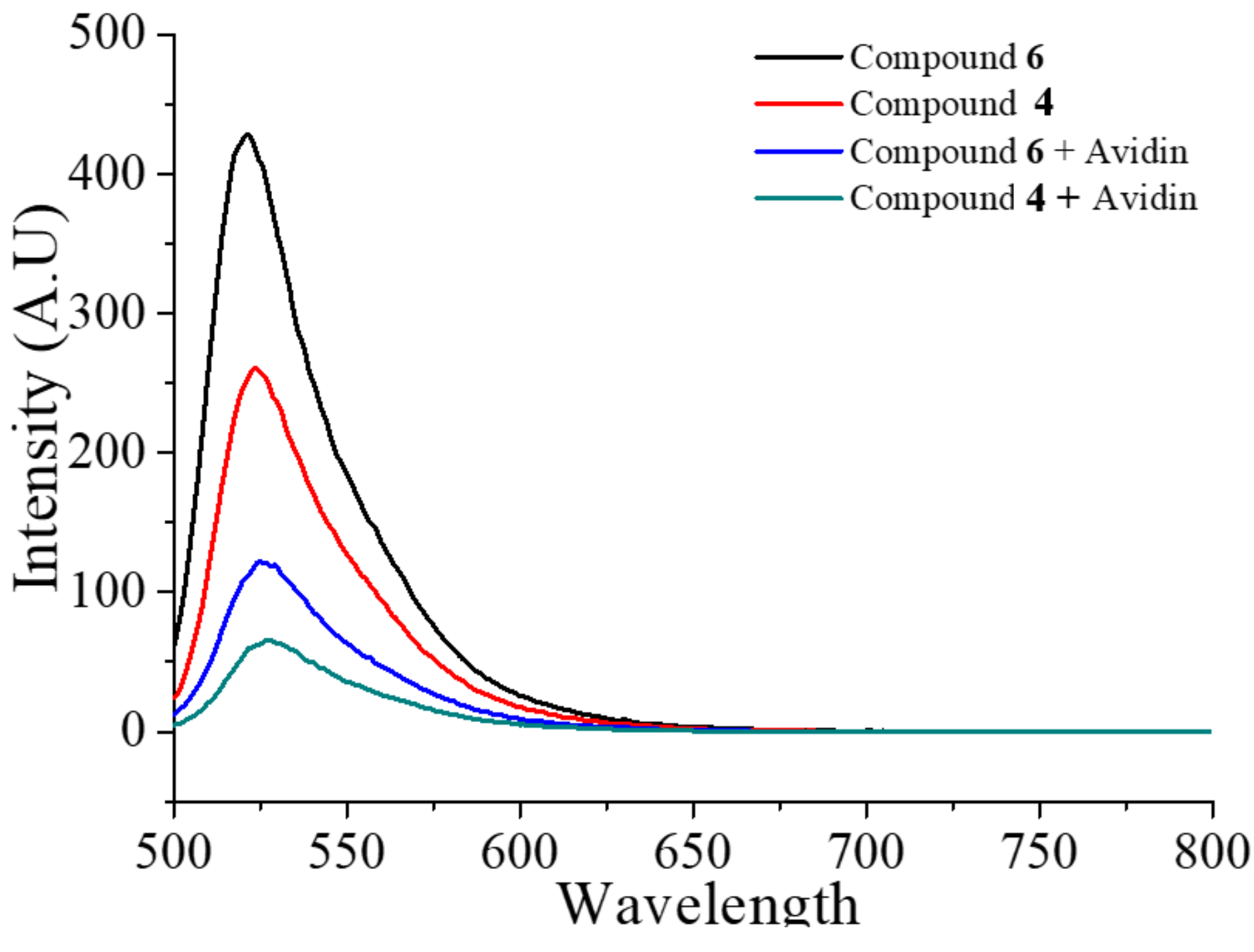

Figure S9.1. Fluorescence spectra of compounds in methanol and water mixture (1:100) before and after addition of avidin (1:1).

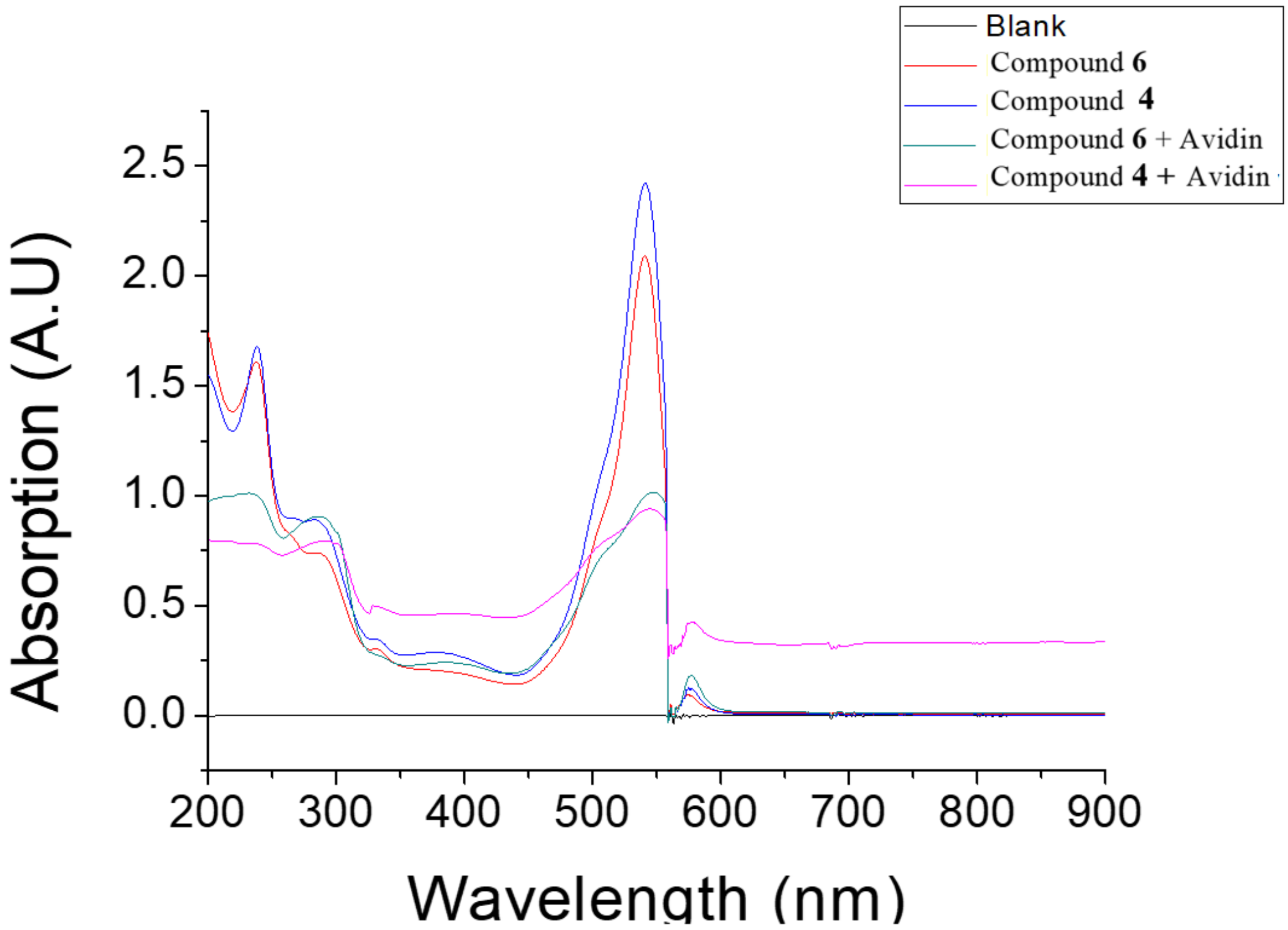

Figure S9.2. UV-Vis spectroscopy of compounds 4 and 6 in methanol and water mixture (1:100) before and after addition of avidin (1:1).

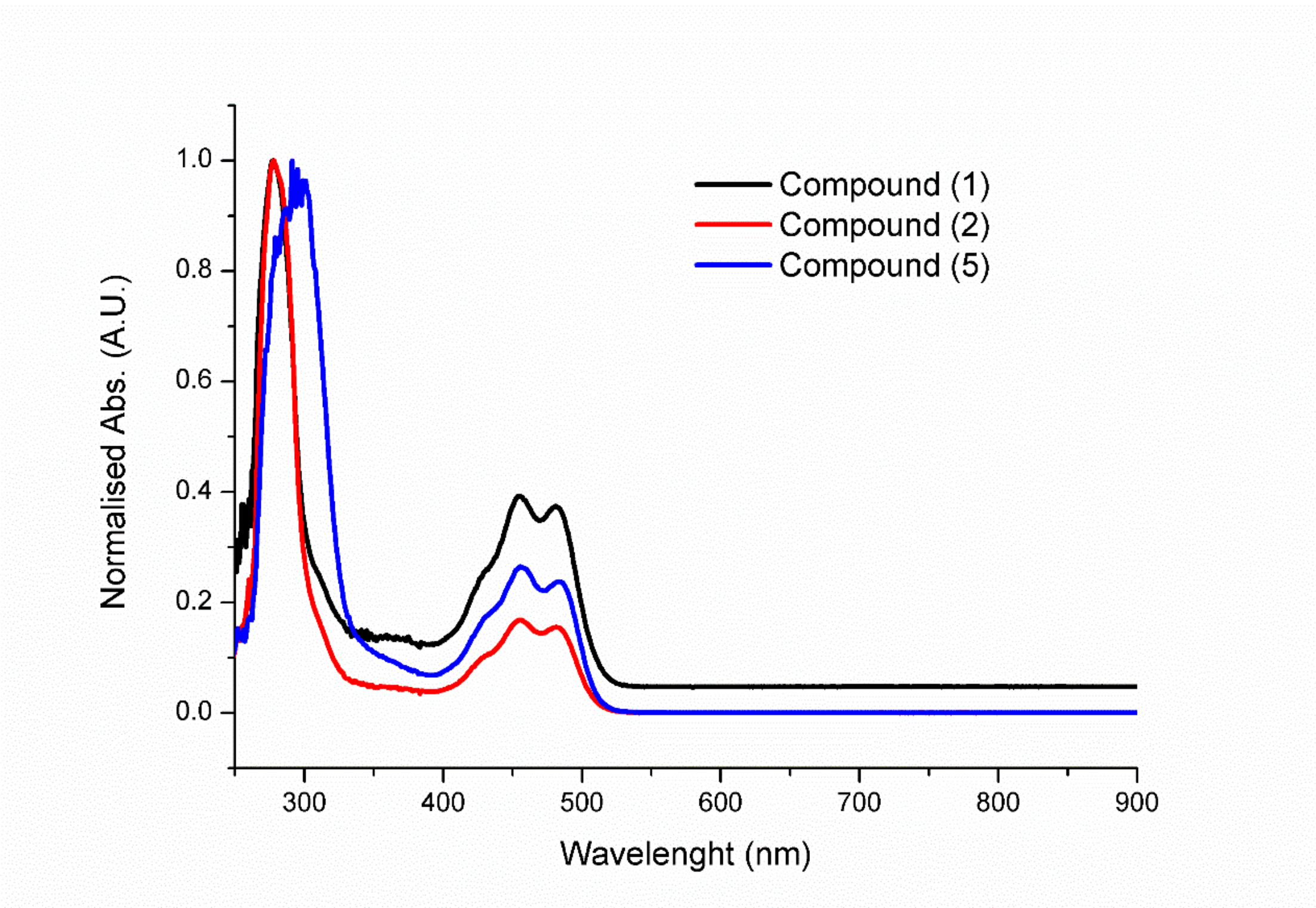

Figure S9.3. UV-Vis spectroscopy of compounds **1, 2** and **5**, 100 µM in DMSO.

General Avidin binding test using flow cytometry and confocal imaging of 5 and cryoSEM imaging of 5@glucan.

Compound **5** as obtained from the synthetic procedure above, was dissolved in methanol and diluted to 100 mL in a volumetric flask to prepare a 3.5 × 10^{-4} mM stock solution. A 1 mL aliquot of this stock solution was transferred into two microtubes, each containing 20 µL of avidin-coated polystyrene beads and 20 µL of avidin-coated agarose beads, respectively. As controls, two additional microtubes were prepared with the same types of beads but filled with 1 mL of MilliQ water instead of solutions of compound **5**. All four tubes were gently shaken to facilitate biotin–avidin binding. The tubes were then centrifuged until the beads fully sedimented. The samples treated with compound 5 were resuspended in water to remove any traces of unbound compound 5. Fluorescence imaging was performed and a comparison between flow cytometry and confocal laser scanning microscopy with excitation at 488 nm was performed

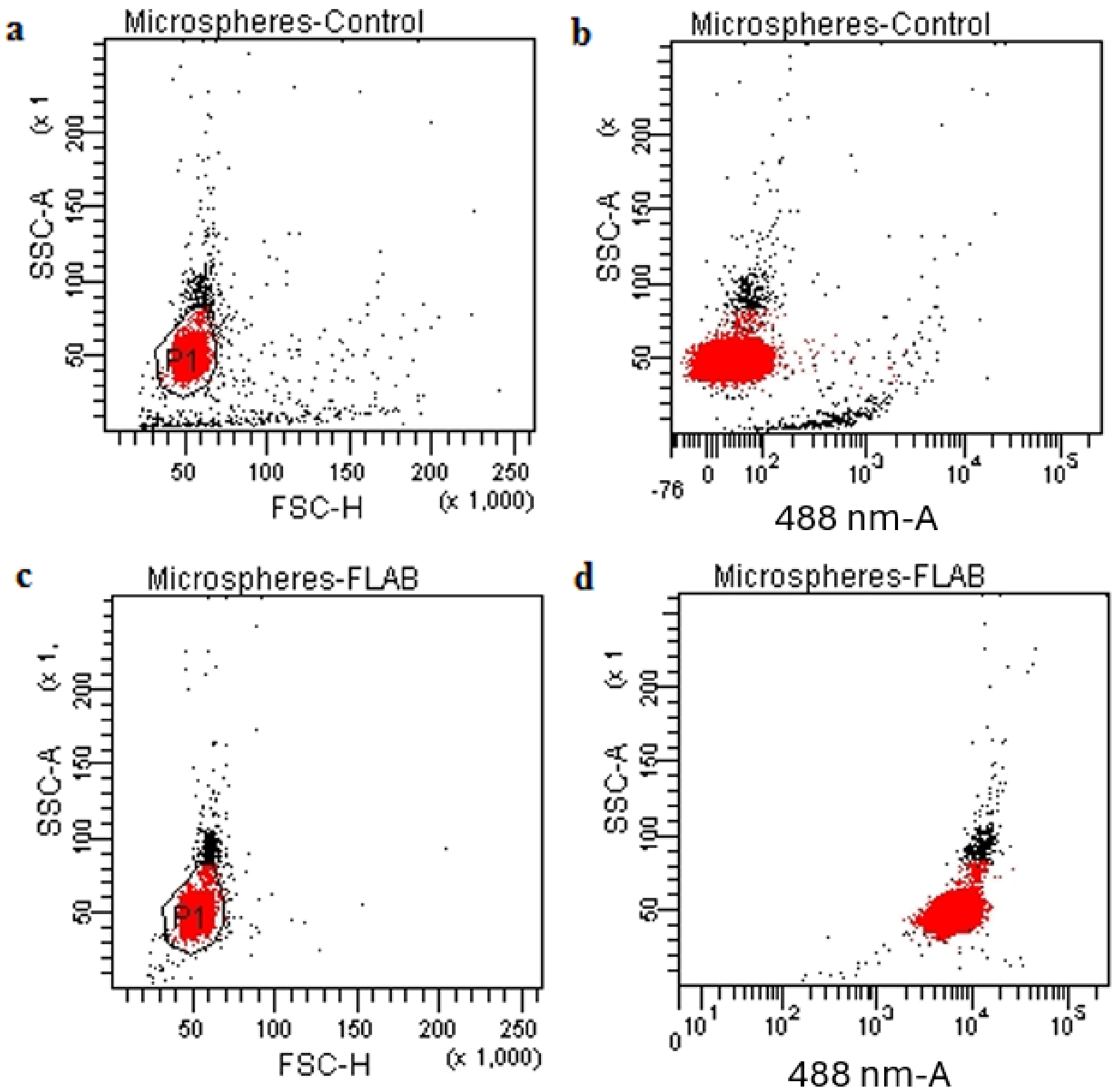

Figure S9.4. (a) polystyrene beads (10 µm) coated with avidin in 1ml water (b) polystyrene (10 µm) beads coated with avidin in 1 mL water, 488 nm green light (c) polystyrene beads (10µm) coated with avidin in 1 mL biotin control stock solution (3.5 × 10^{-4} mM) (d) polystyrene beads (10 µm) coated with avidin in 1ml compound **5** stock solution (3.5 × 10^{-4} mM), 488 nm green light.

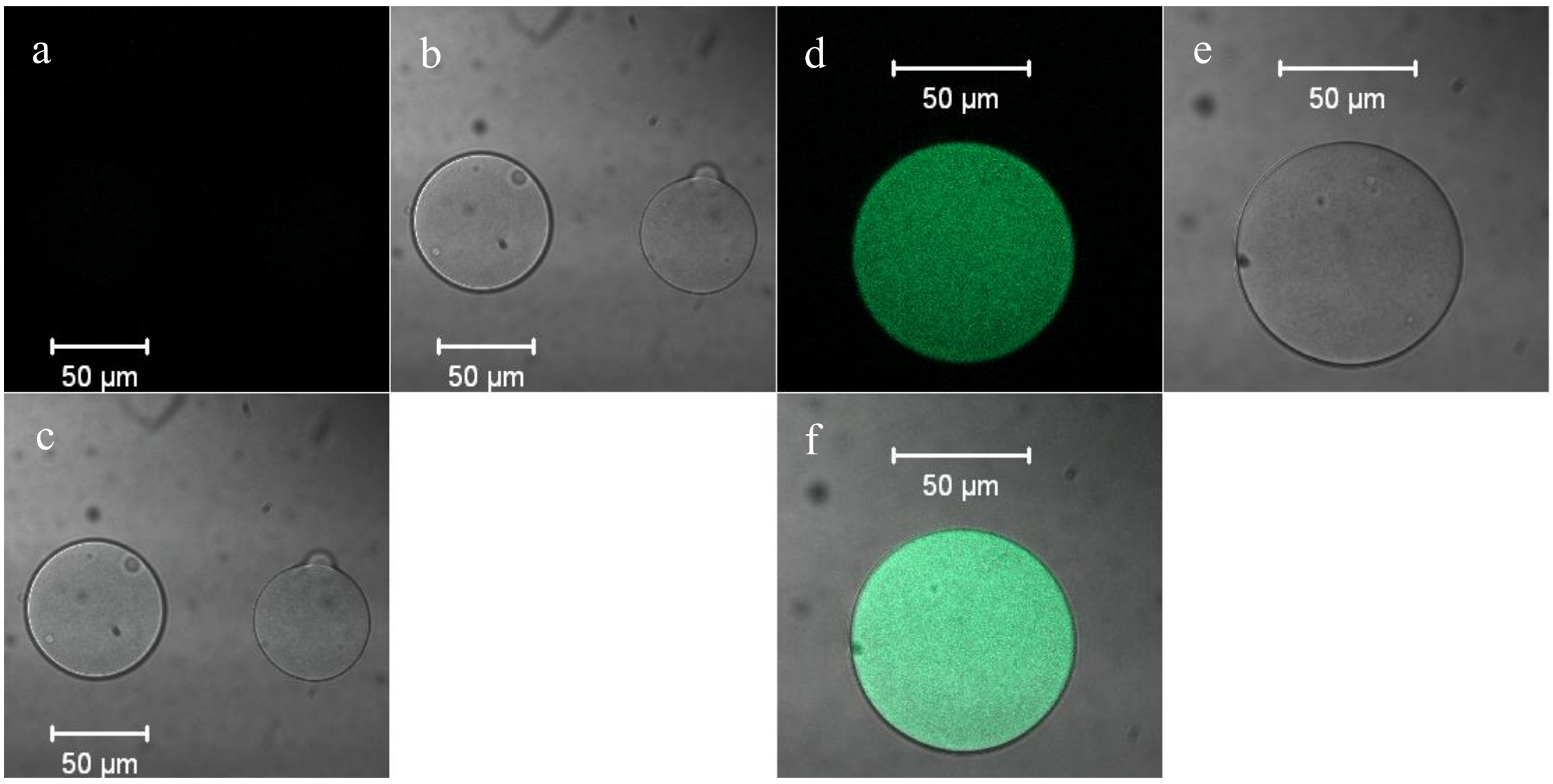

Figure S9.5. (a) Agarose beads (50 µm) coated with avidin in 1mL water, excitation 488 nm. (b) Agarose beads (50 µm) coated with avidin in 1mL water (bright field). (c) Agarose beads (50 µm) coated with avidin in 1mL water, overlay of (a) and (b). (d) Agarose beads (50 µm) coated with avidin in 1mL compound **5**, under 488 nm excitation (emission in GFP channel) (e) Agarose beads (50 µm) coated with avidin in 1mL compound **5**, bright field (f) Agarose beads (50 µm) coated with avidin in 1mL compound **5**, overlay of (d) and (e).

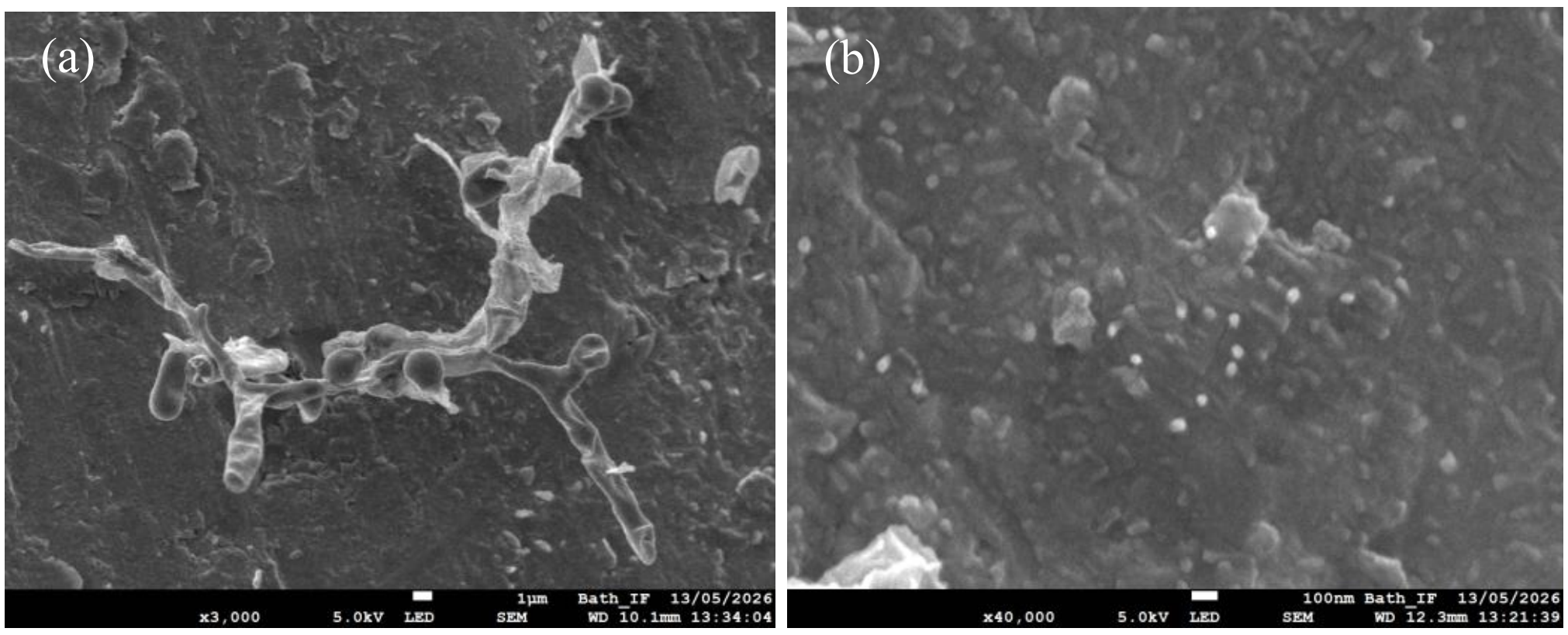

Figure S9.6. CryoSEM analysis of **5@beta-D-glucan** as formulated using 1 mg/mL DMSO:PBS at different magnifications.

10. Kinetic stability tests

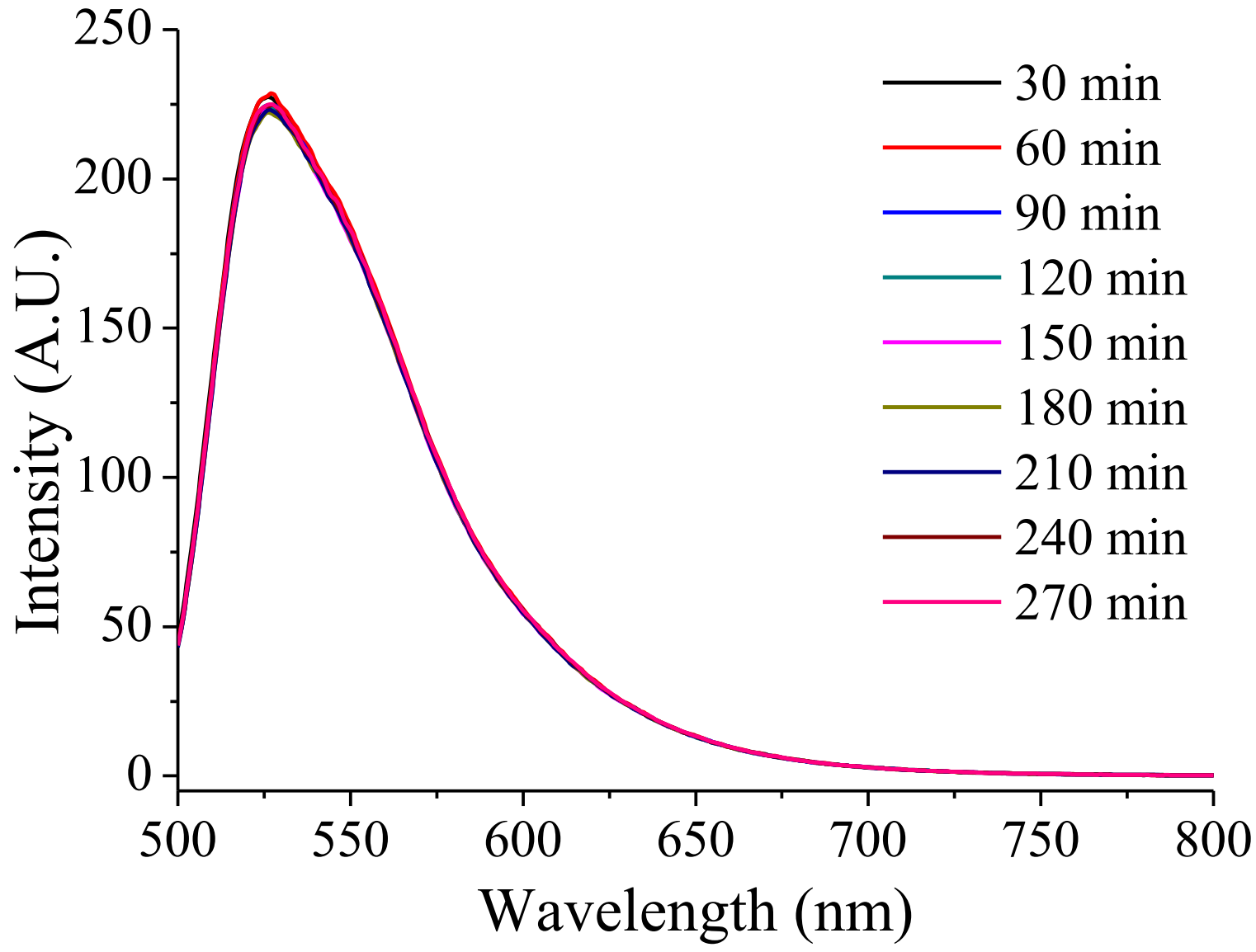

Figure S10.1. Fluorescent stability study of compound **6** over time, 100 μM in methanol: water mixture (1:40)

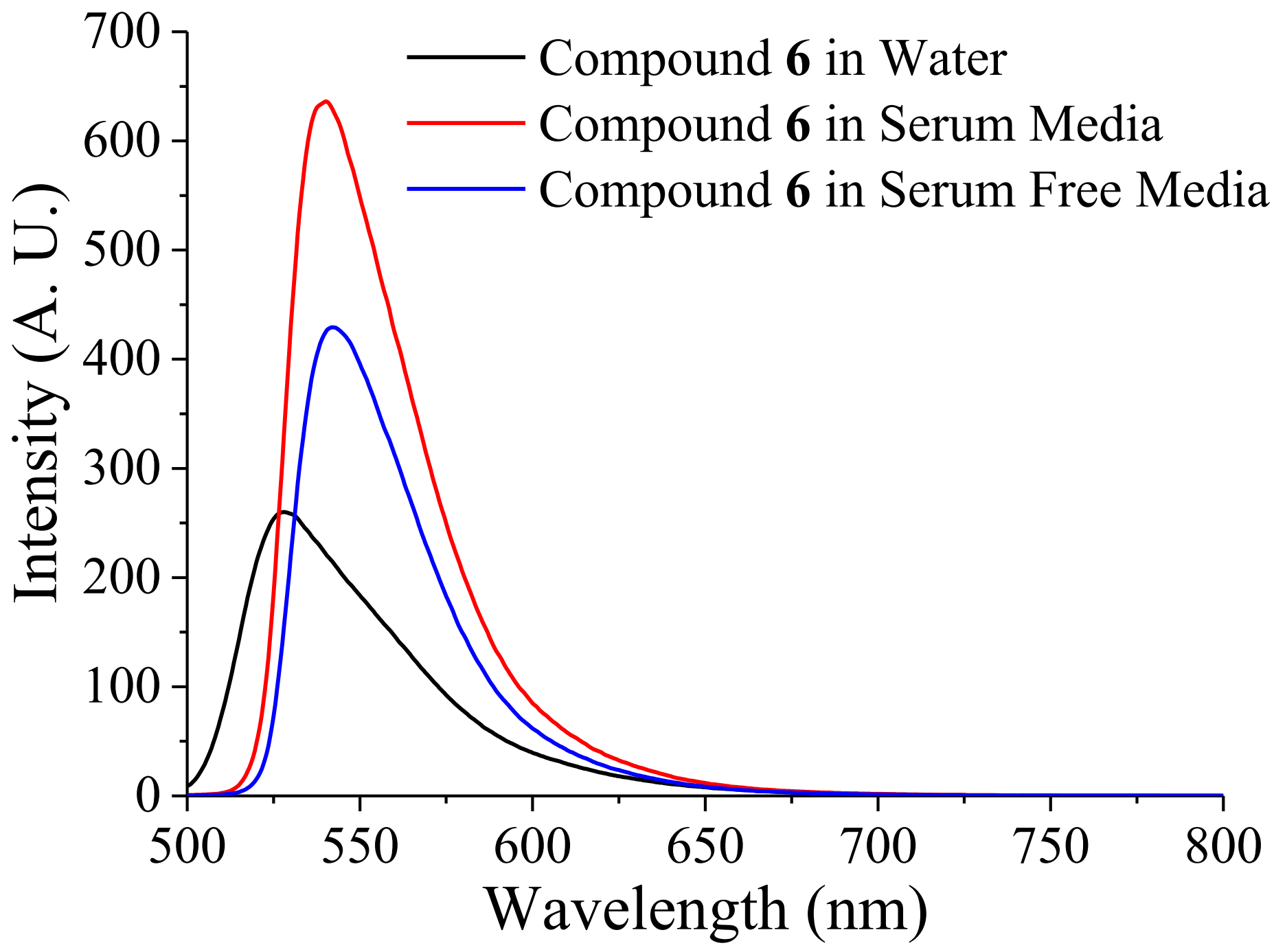

Figure S10.2. Fluorescent stability study of compound **6** in different solvent systems, 100 μM conc.

11. Imaging of core-shell nanoparticles labelled with Compound 6:

2P FLIM of $6@Fe_3O_4@SiO_2$

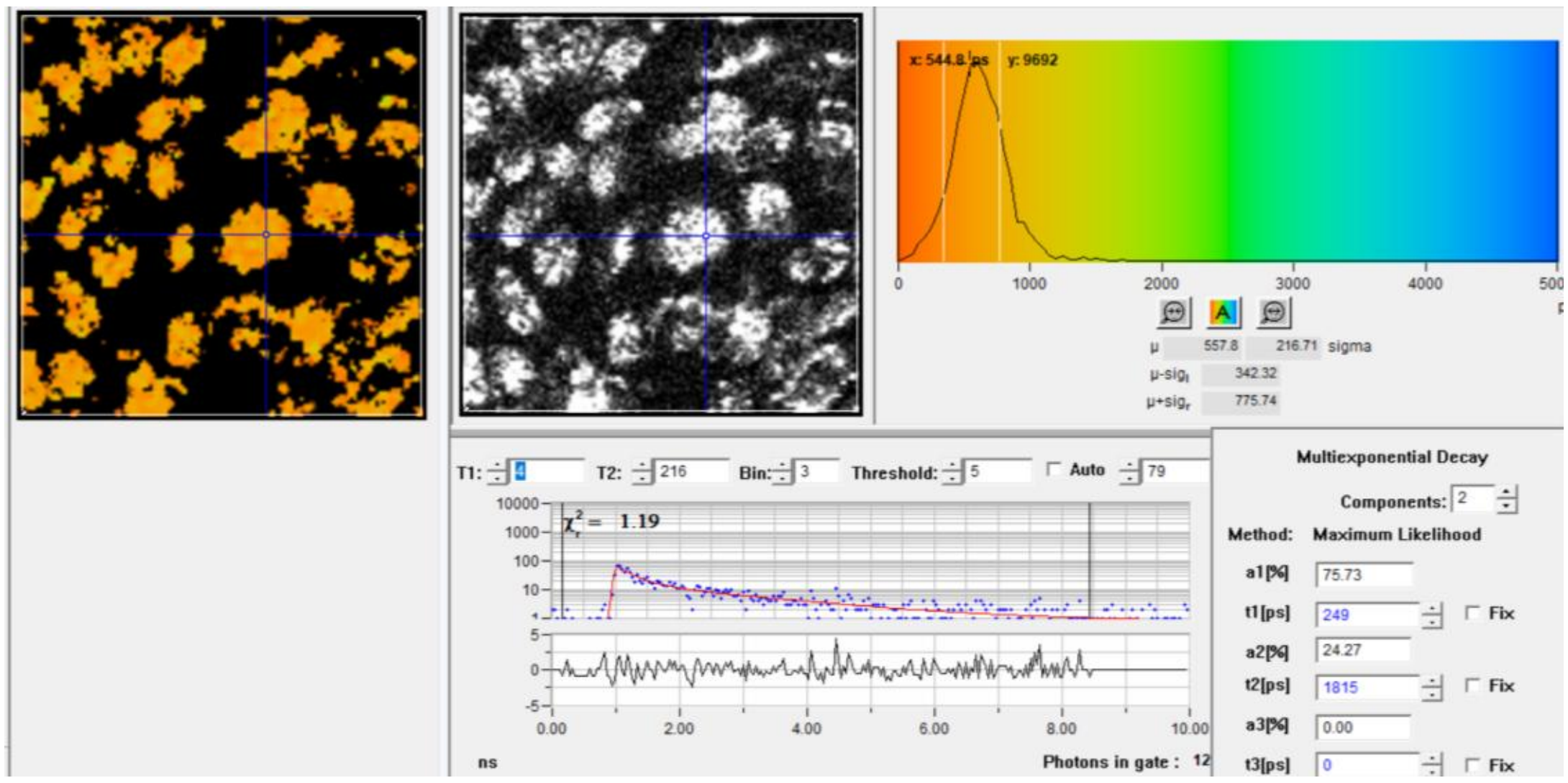

Figure S11.1. 2P FLIM of **6**@Fe_3O_4@SiO_2 10 µg/mL in 1% DMSO, hybrid analysed in healthy controls (cell line CHO), 15 min incubation at 37 ºC, 910 nm, 2 mW laser power (clockwise: lifetime distribution map, intensity image, and parameters including TCSPC in a random spot in cytoplasm). 100 µm field of view, Lifetime scale bar: τ_m=0-5000 ps.

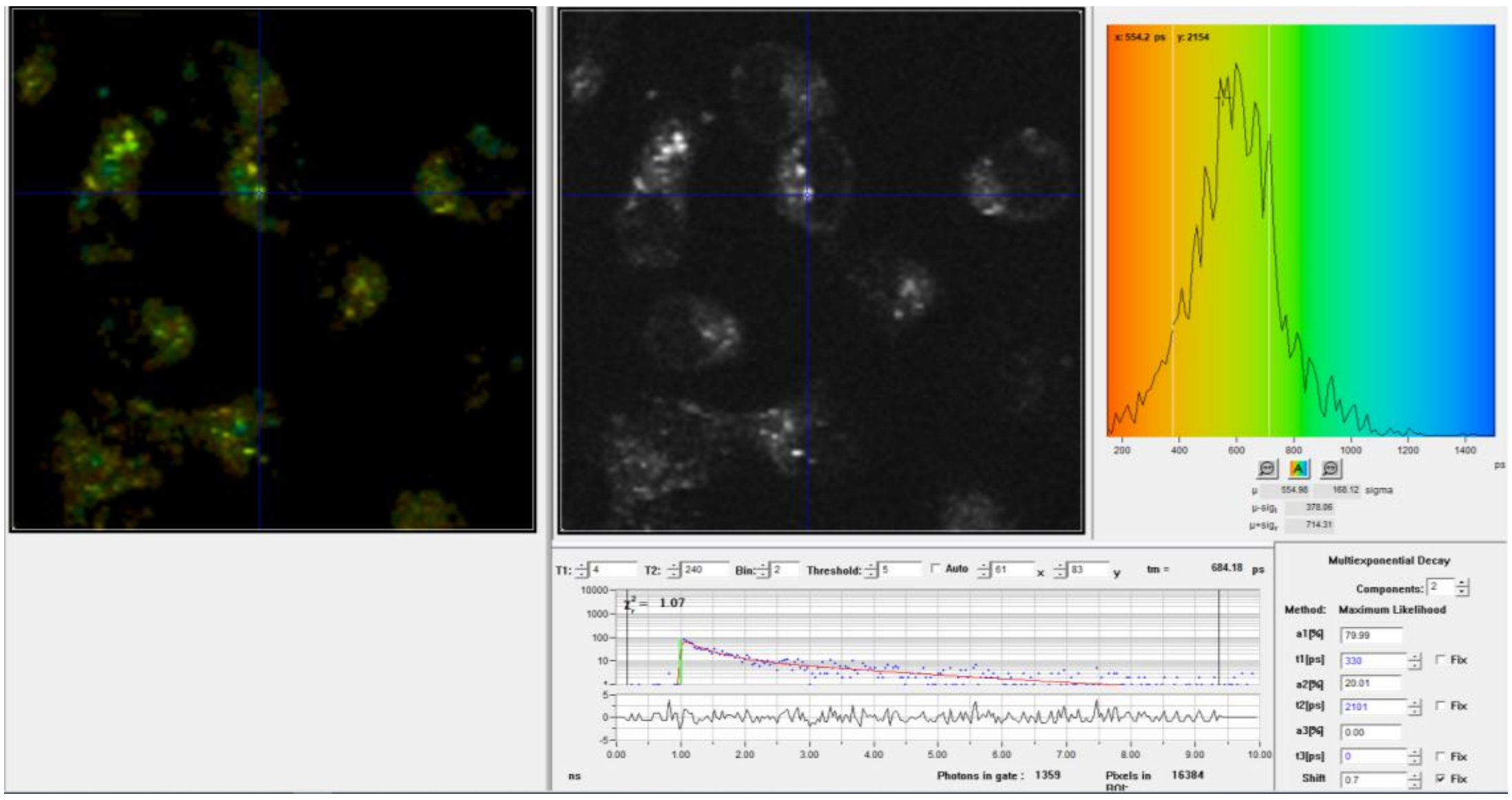

Figure S11.2. 2P FLIM at the uptake of Compound **6**@Fe_3O_4@SiO_2 hybrid in CHO, 1 mg/mL in 1% DMSO, 15 min incubation at 37 ºC, 910 nm, 8.7 mW, (clockwise: lifetime distribution map, intensity image and parameters including TCSPC in a random spot in cytoplasm). 100 um field of view, Lifetime scale bar: τ_m=200-1500 ps

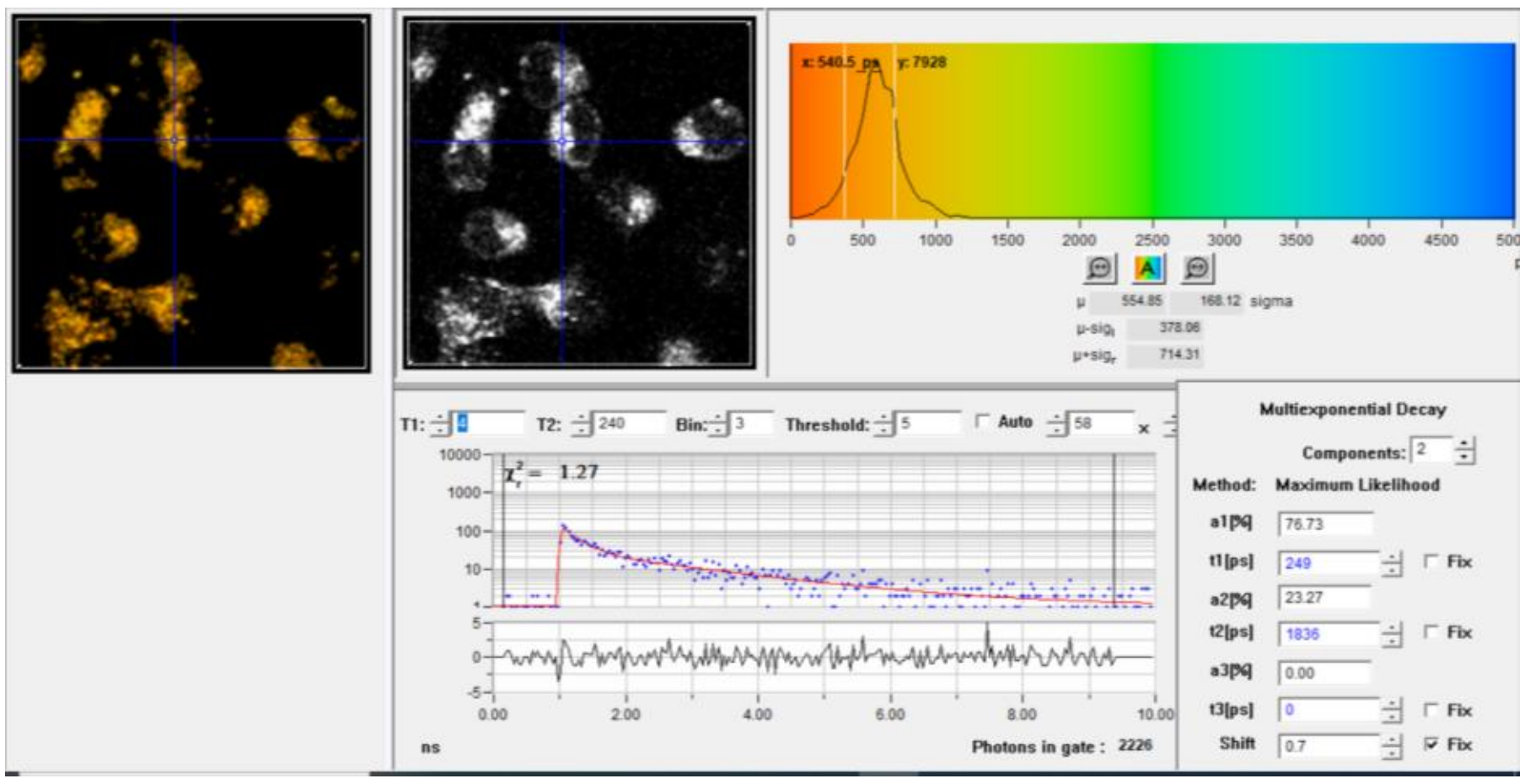

A) Spot 1

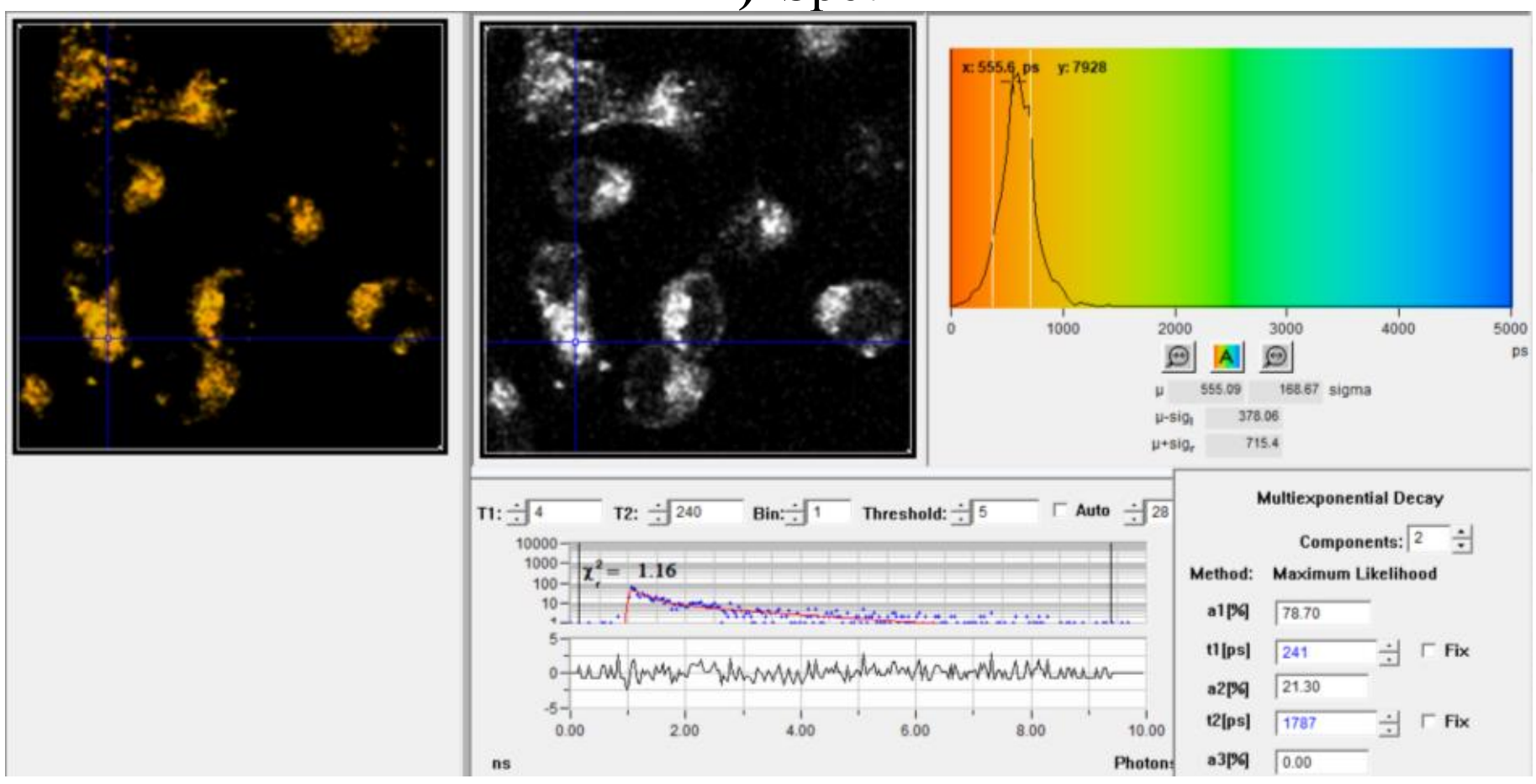

(B) Spot 2

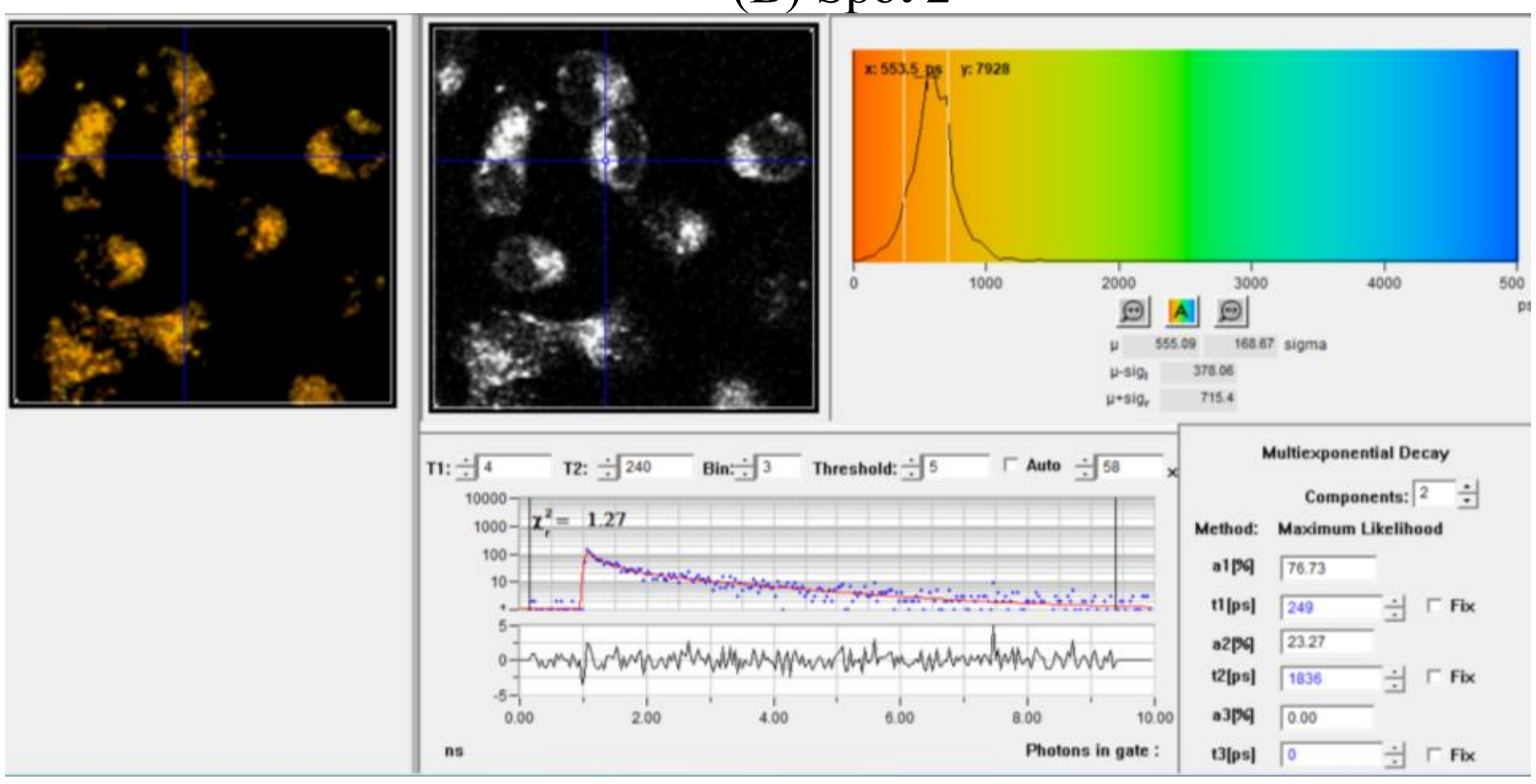

(C) Spot 3

Figure S11.3. 2P FLIM at the uptake of Compound **6**@Fe_3O_4@SiO_2 hybrid in CHO, 1 mg/mL in 1% DMSO, 15 min incubation at 37 ºC, 910 nm, 8.7 mW, (clockwise: lifetime distribution map, intensity image and parameters including TCSPC in a random spot in cytoplasm). 100 um field of view, Lifetime scale bar: τ_m =0-5000 ps

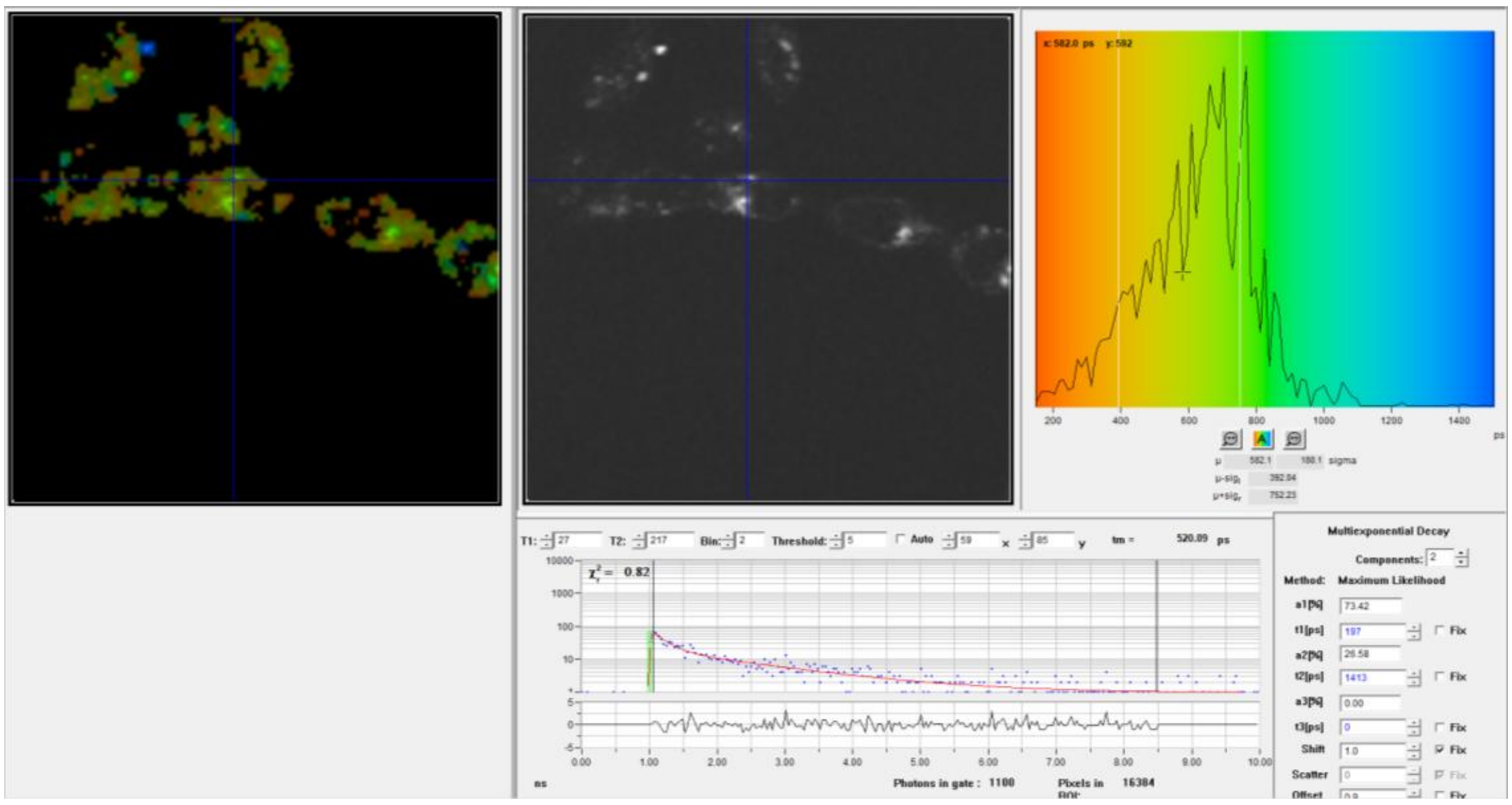

Figure S11.4. 2P FLIM at the uptake of Compound **6**@Fe_3O_4@SiO_2 hybrid in HeLa, 1 mg/mL in 1% DMSO, 15 min incubation at 37 ºC, 910 nm, 8.7 mW, (clockwise: lifetime distribution map, intensity image and parameters including TCSPC in a random spot in cytoplasm). 100 um field of view, Lifetime scale bar: τ_m =180-1500 ps.

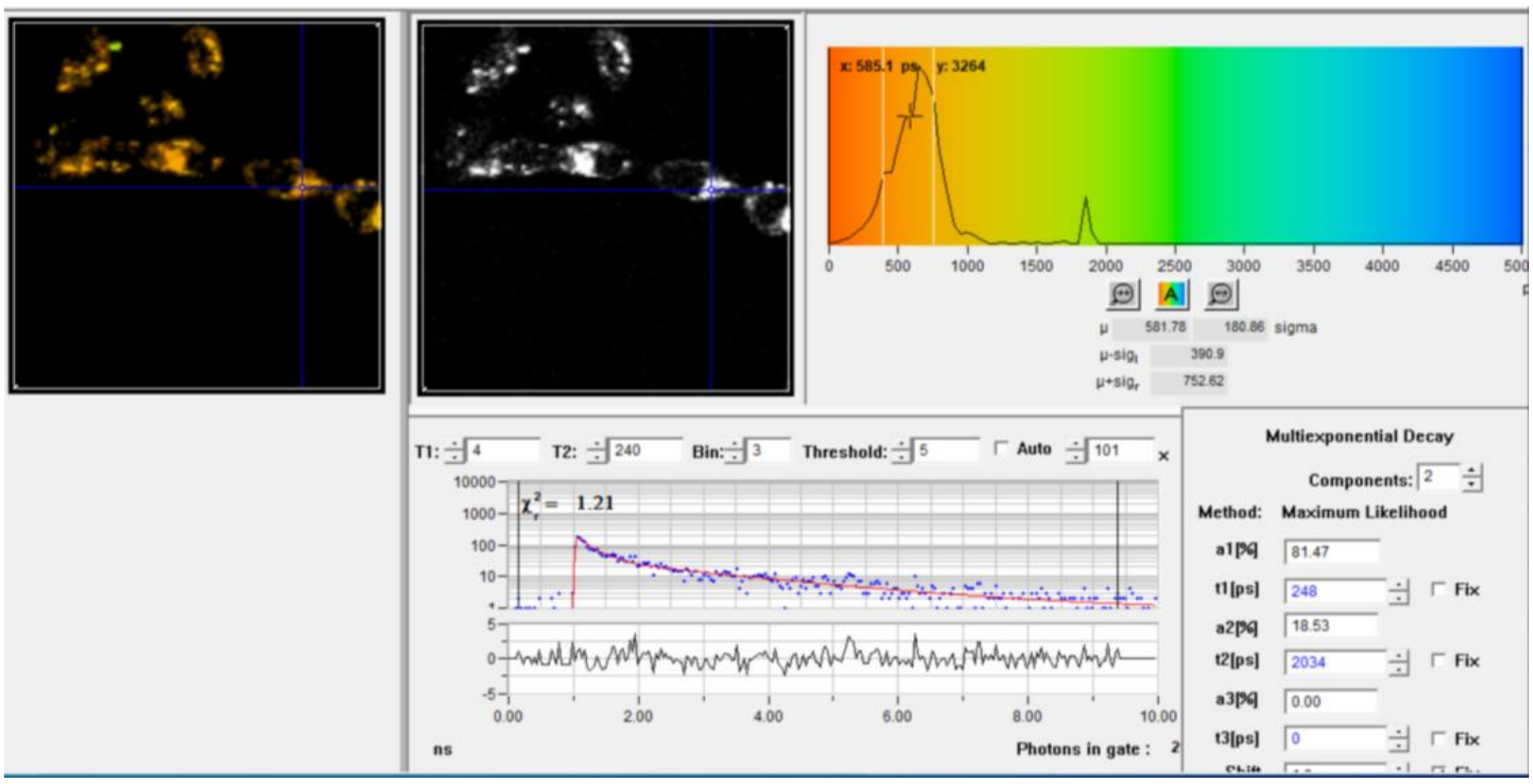

Figure S11.5. 2P FLIM at the uptake of Compound **6**@Fe_3O_4@SiO_2 hybrid in HeLa, 1 mg/mL in 1% DMSO, 15 min incubation at 37 ºC, 910 nm, 8.7 mW, (clockwise: lifetime distribution map, intensity image and parameters including TCSPC in a random spot in cytoplasm). 100 um field of view, Lifetime scale bar: τ_m =0-5000 ps.

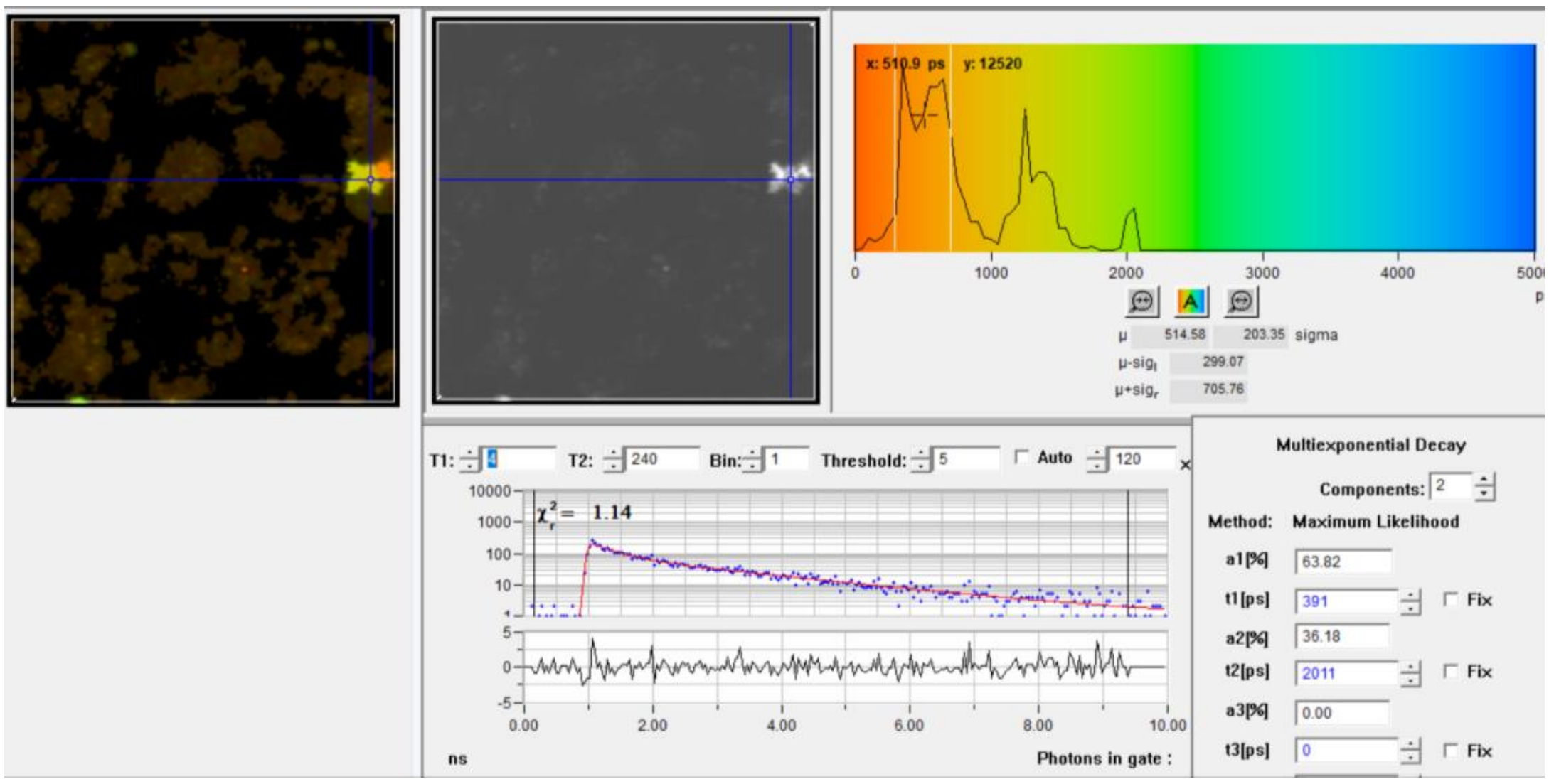

Figure S11.6. 2P FLIM at the uptake of **6**@Fe_3O_4@SiO_2 hybrid in CHO cells (alternative field of view, showing aggregated nanoparticles), 10 µg/mL in 1% DMSO, hybrid analysed in healthy controls (cell line CHO), 15 min incubation at 37 ºC, 910 nm, 2 mW laser power (clockwise: lifetime distribution map, intensity image, and parameters including TCSPC in a random spot in cytoplasm). 100 µm field of view, Lifetime scale bar: τ_m=0-5000 ps.

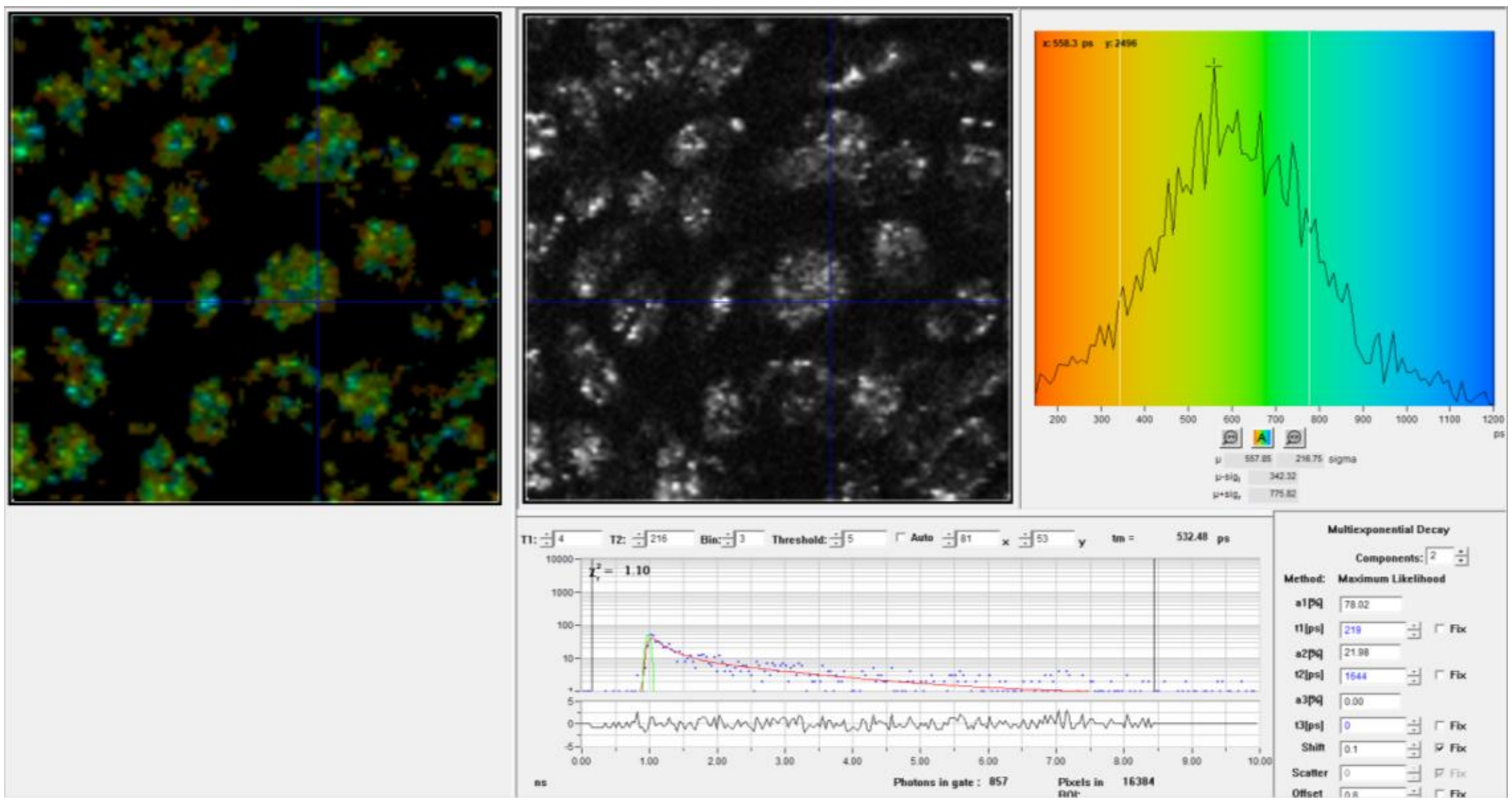

Figure S11.7. 2P FLIM at the uptake of **6**@Fe_3O_4@SiO_2 hybrid in CHO cells (alternative field of view, showing aggregated nanoparticles), 10 µg/mL in 1% DMSO, hybrid analysed in healthy controls (cell line CHO), 15 min incubation at 37 ºC, 910 nm, 2 mW laser power (clockwise: lifetime distribution map, intensity image, and parameters including TCSPC in a random spot in cytoplasm). 100 µm field of view, Lifetime scale bar: τ_m=180-1200 ps.

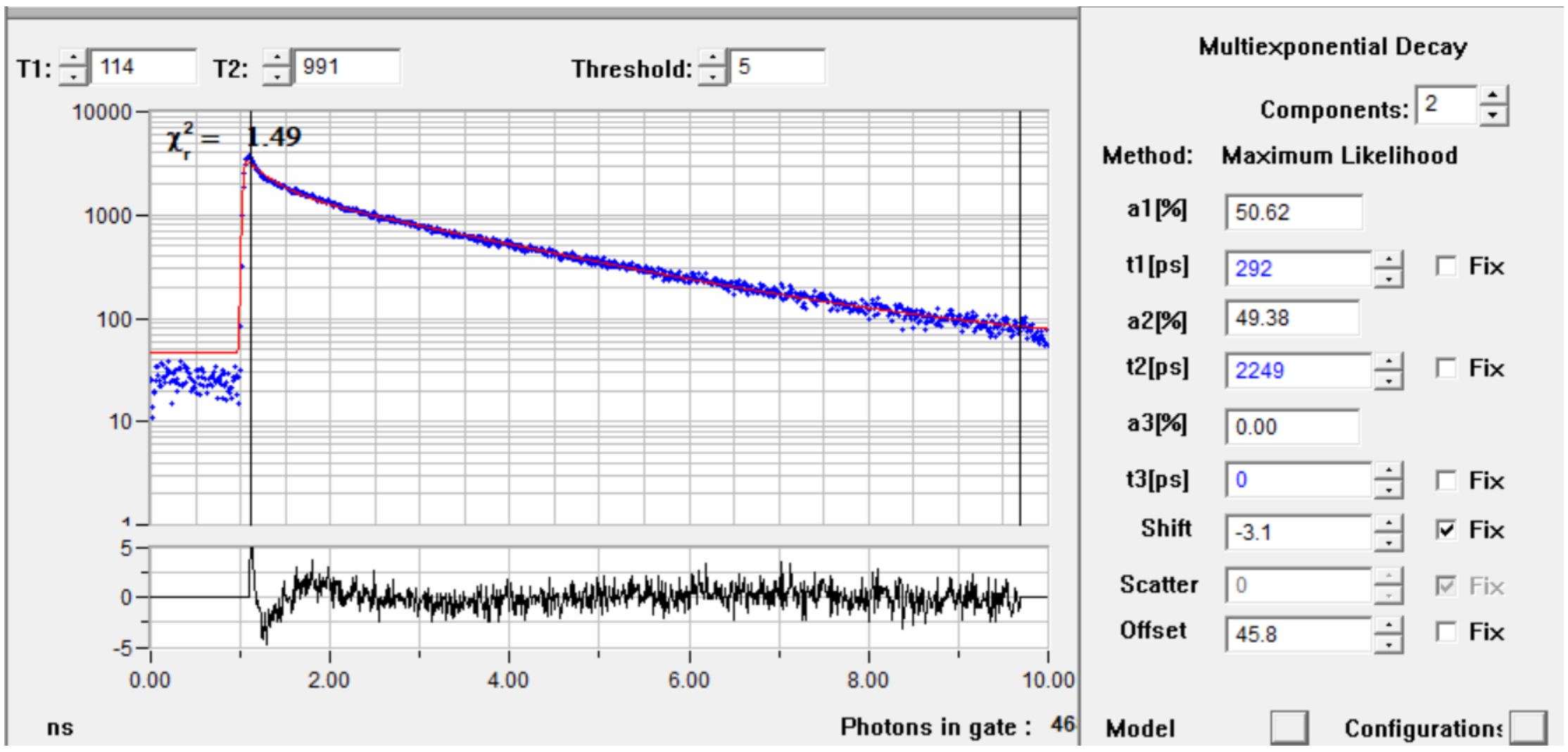

Figure S11.8. 2P TCSPC lifetime point decay of **6**@Fe_3O_4@SiO_2 hybrid in dispersed phase, 1 mg/mL in DMSO, excitation 910 nm.

12. Aggregation by DOSY and Proposed 3D structures of β-D-glucan strand labelled with Compound 1

The 1D DOSY spectra of a 5mM concentration solution of beta-D-glucan in D_2O was recorded and data was represented as a stacked plot of ^1H spectra with increasing magnetic field strength. At the highest field strength used, 33.1 G/cm, most of the beta-D-glucan signal was attenuated. From this it was possible to estimate the actual diffusion speed of the molecules of ca 8 x 10-12 m^2/s which is slow compared with that of pure H_2O is about 3 x 10-9 m^2/s. This suggests that the aggregated size of the beta-D-glucan used hereby is of the order of 300 Angstroms. A repeated experiment from a different batch gave a diffusion speed of sample 3 is similar (5 x 10-11 m2/s), which indicates a smaller size nanoparticle however the errors on this 1D DOSY was also larger suggesting that both samples are similar in nature overall and in the nanometer range in H_2O. This is consistent with the average molecular weight range expected of 20-60 kDa and with the aggregation behaviour on mica as evident from the AFM measurements recorded on the same sample. The 2D DOSY experiments performed on pinacol-protected compound 5 in presence and absence of excess beta-D-glucan in D_2O mixtures were inconclusive with respect to determining the size of the aggregates and the spectrum was dominated by the presence of unbound glucan species. High dilution titration experiments by fluorescence caried out at ca. 10 microMolar concentration ranges indicated a ca. 2 x 10^4 M^{-1} binding constant for the formation of 5@glucan in DMSO:PBS 1:1 mixtures.

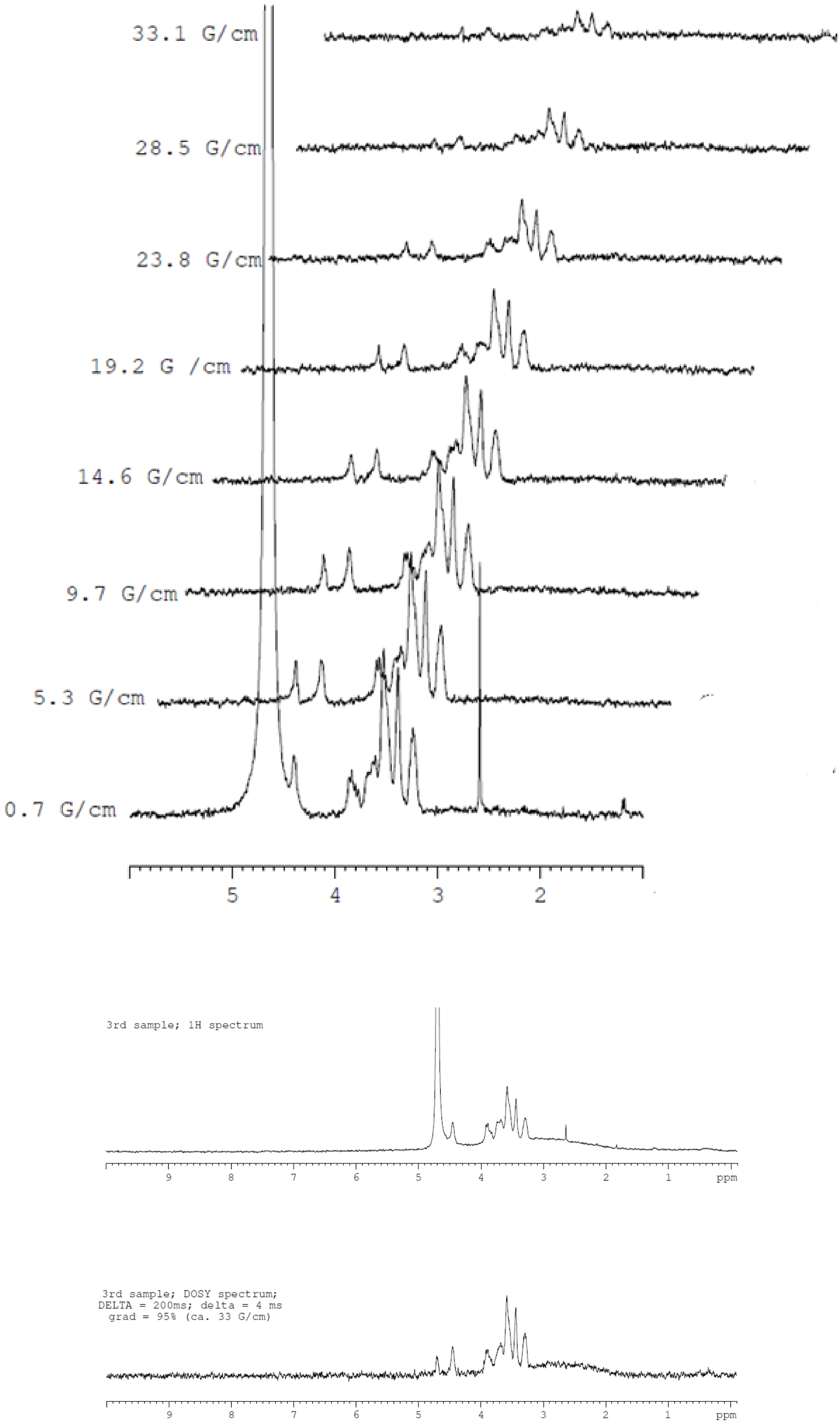

Figure S12.1. DOSY spectroscopy of a 5 mM concentration solution of beta-D-glucan in D_2O

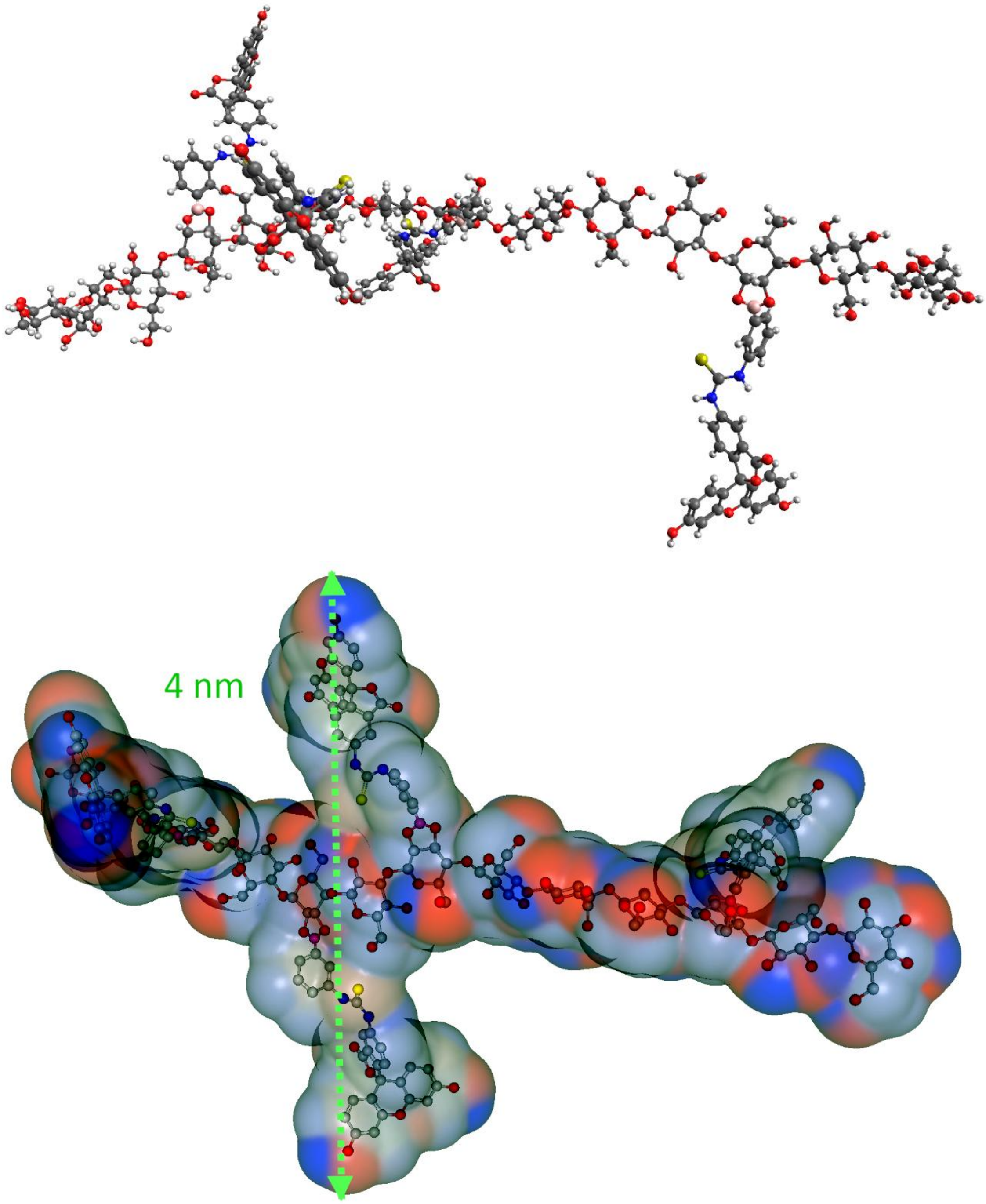

Figure S12.2. a) Proposed 3D structure of single β-D-glucan strand with four molecules of fluorophore **1** attached to the surface (GAFF, Avogadro), **b)** Solvent-accessible surface with electrostatic potential mapping (MarvinSpace).

The geometry of the **1**@glucan composite was optimised at MM^+ level aiming to visualize the possible 3D structure of them with sizes around 4 - 5 nm in the direction perpendicular to the growth direction of the strand. It is likely that upon binding the molecules prevent the formation of the triple helix, but also as show in figure S11.3., the helical aggregate can be maintained with some of the coordinated fluorophores inside of the helical structure.

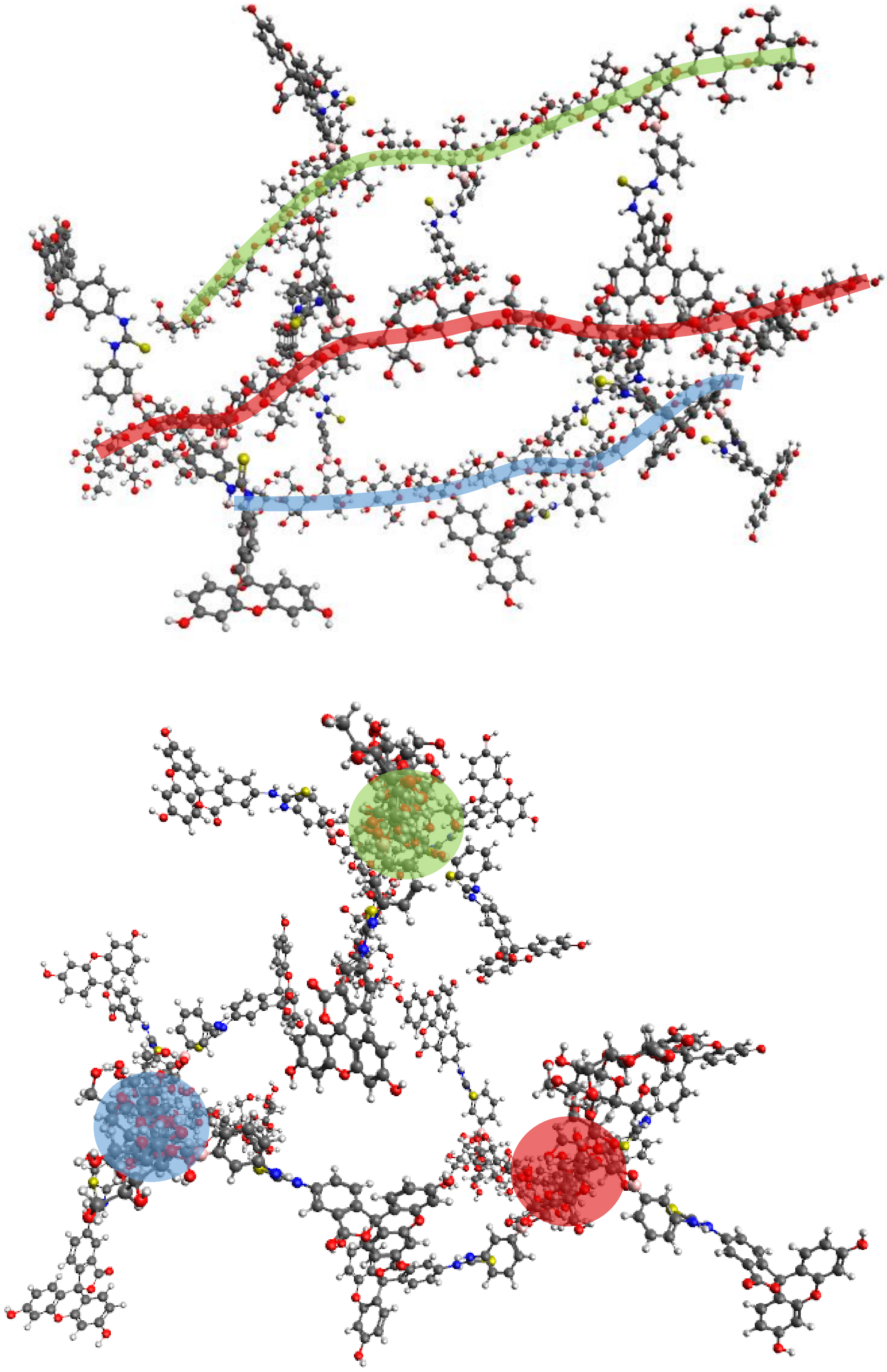

Figure S12.3. Proposed 3D structure of a β-D-glucan triple helix with fluorophore **1** attached to the surface of the glucan, some of them are inside of the helical aggregate (GAFF, Avogadro)[14-15].

13. In vivo assays

Zebrafish husbandry

All experiments were conducted with approval from the local ethical review committee at the University of Bath and in accordance with the UK Home Office regulations (Guidance on the Operation of Animals, Scientific Procedures Act, 1986). All work was performed on zebrafish (Danio *rerio*) using wildtype AB strains or the insulin mutant ins^{bns102} strain [16] (Mullapudi, 2018) maintained on an AB background which were reared at 28.5°C on a 14hr light/10hr dark cycle. ins^{bns102} mutants genotyped post-imaging, utilising a HotSHOT DNA extraction approach [17] (Meeker, 2007), using primers 5'-GTGCTCTGTTGGTCCTGTTGG-3' and 5'-CATCGACCAGATGAGATCCACAC-3', analysing the PCR products on a 3% agarose/TBE gel. Staging and husbandry were performed as previously described [18] (Westerfield, 1995). Embryos were raised in E3 media (5.0 mM NaCl, 0.17 mM KCl, 0.33 mM CaCl2, 0.33 mM MgSO4) with 0.1% methylene blue up to the point of experimentation, according to standard protocols.

Zebrafish compound treatment and imaging experiments

Compound treatment was performed at 50 μM in 1% DMSO for the indicated period of time (20, 60 or 120 minutes), using E3 media without additional methylene blue. Following treatment, larvae were washed 3 x 5 minutes in E3 media, followed by immediate imaging. Prior to imaging, zebrafish larvae (4 dpf) were anaesthetised using Tricaine (Sigma-Aldrich). Fluorescent stereomicroscope images were generated on a Leica MZFLiii system (Leica Microsystems) and captured using an Olympus EP50 camera (Evident).

Zebrafish image analysis and statistics

Fiji (ver. 2.16.0) was used for all image analysis. Fluorescent regions of interest (ROIs) were selected by utilising the "wand" tool (Legacy mode), and these were used to quantify fluorescence intensity for the digestive system. Mean fluorescence intensity values were extracted for each ROI. Mean values were calculated and plotted using GraphPad Prism (ver. 10.6.1). Statistical analysis was carried out using a one-way ANOVA test. Results are represented as mean ± SEM. The criterion for statistical significance was set at $P<0.05$.

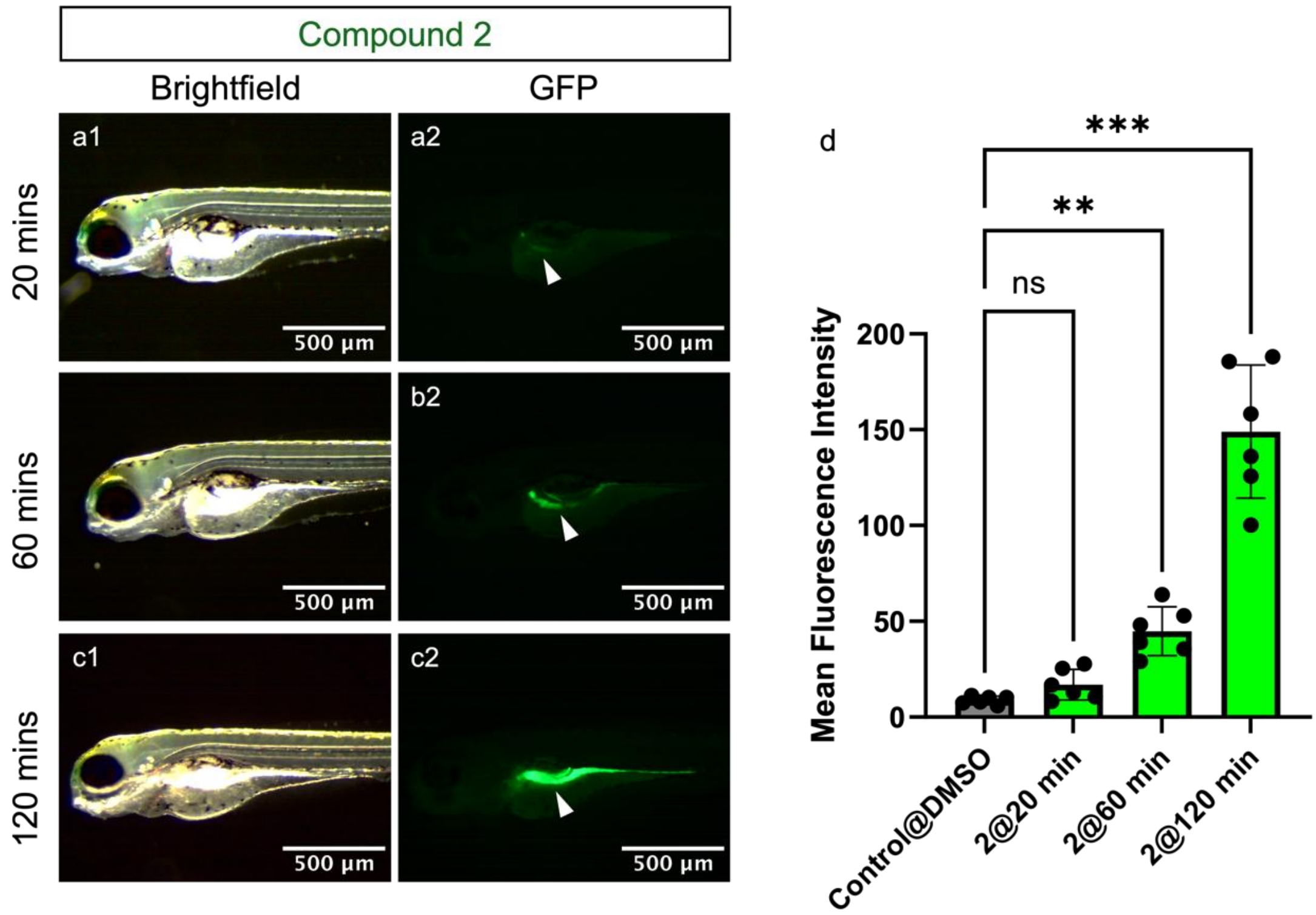

Figure S13.1. Representative brightfield and GFP stereomicroscope images showing different incubation times tested on 4dpf zebrafish at 50µM Compound 2, arrows indicate labelled digestive system, where (a) **2**, 1% DMSO, 20 minutes, (b) **2**, 1% DMSO, 60 minutes, (c) **2**, 1% DMSO, 120 minutes, (d) mean green fluorescence intensity of a_2-c_2, calculated from six treated larval fish per condition, error bar stands for the standard deviation. a_1-f_1 is the brightfield channel; a_2-f_2 is the green channel, compounds were excited at 488 nm and emitted at 510-530 nm. Significant difference determined via Brown-Forsythe and Welch ANOVA testing and confirmed through post hoc Dunnett's multiple comparison. (*) <0.05, (**) <0.010, (***) <0.001 and (****) < 0.0001.

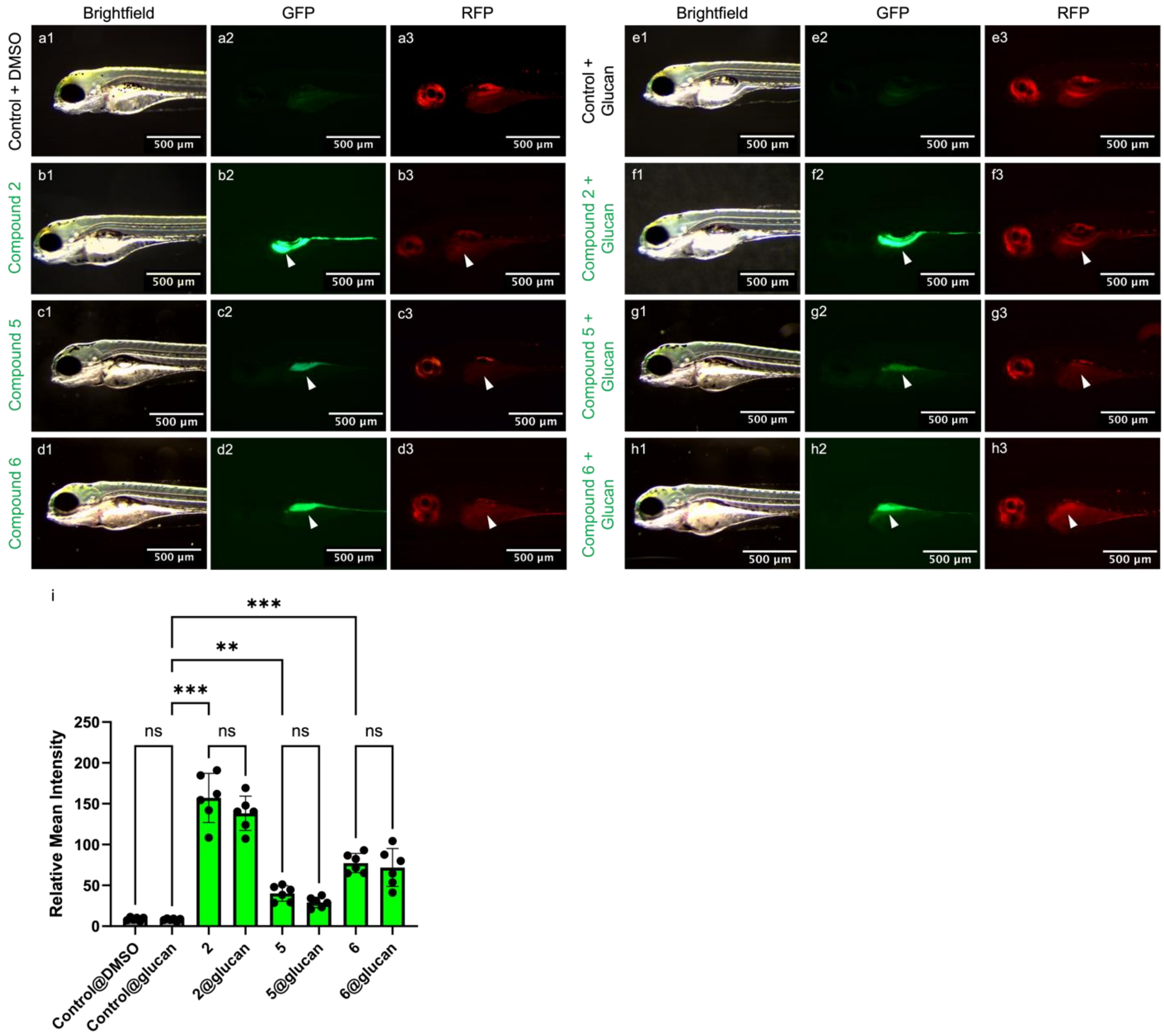

Figure 13.2. Representative brightfield, GFP and RFP stereomicroscope images of 4dpf zebrafish larvae incubated with compounds at 50 µM for 120 minutes at 28.5°C, arrows indicate labelled digestive system, where (a) 1% DMSO in embryo media, (b) **2**, 1% DMSO, (c) **5**, 1% DMSO, (d) **6**, 1% DMSO, (e) 1% DMSO @glucan, (f) **2**, 1% DMSO @glucan, (g) **5**, 1% DMSO @glucan, (h) **6**, 1% DMSO @glucan, (i) mean green fluorescence intensity of a_2-h_2, calculated from six treated larval fish per condition, error bar stands for the standard deviation. a_1-h_1 is the brightfield channel; a_2-h_2 is the green channel, compounds were excited at 488 nm and emitted at 510-530 nm; a_3-h_3 is the red channel, compounds were excited at 561 nm and emitted at 575-615 nm. Significant difference determined via Brown-Forsythe and Welch ANOVA testing and confirmed through post hoc Dunnett's multiple comparison. (*) <0.05, (**) <0.010, (***) <0.001 and (****) < 0.0001.

14. References:

1. S.A. Elfeky, S.E. Flower, N. Masumoto, F. D'Hooge, L. Labarthe, W. Chen, C. Len, T.D. James, J.S. Fossey, Chem. Asian J. 2010, 5, 581–588.

2. M.P.P. Morais, D. Marshall, S.E. Flower, C.J. Caunt, T.D. James, R.J. Williams, N.R. Waterfield, J.M. Van Den Elsen, Sci. Rep. 2013, 3, 1437.

3. M. Ratel, A. Provencher-Girard, S.S. Zhao, J. Breault-Turcot, J.R.M. Labrecque-Carbonneau, M. Branca, J.N. Pelletier, A.R. Schmitzer, J.-F. Masson, Anal. Chem. 2013, 85, 5770–5777.

4. W.K. Chow, C.M. So, C.P. Lau, F.Y. Kwong, Chem. Eur. J. 2011, 17, 6913–6917.

5. W. Stöber, A. Fink, E. Bohn, J. Colloid Interface Sci. 1968, 26, 62–69.

6. M. Lledos, V. Mirabello, S. Sarpaki, H. Ge, H.J. Smugowski, L. Carroll, E.O. Aboagye, F.I. Aigbirhio, S.W. Botchway, J.R. Dilworth, ChemNanoMat 2018, 4, 361–372.

7. A. Roychowdhury, S.P. Pati, S. Kumar, D. Das, Powder Technol. 2014, 254, 583–590.

8. S. Majumder, S. Dey, K. Bagani, S. Dey, S. Banerjee, S. Kumar, Dalton Trans. 2015, 44, 7190–7202.

9. ATCC: https://www.lgcstandards-atcc.org/search?title=All%20Products#sort=relevancy&f:contentTypeFacetATCC=[Products]

10. R.J. Geraghty, A. Capes-Davis, J.M. Davis, J. Downward, R.I. Freshney, I. Knezevic, R. Lovell-Badge, J.R.W. Masters, J. Meredith, G.N. Stacey, P. Thraves, M. Vias, Br. J. Cancer 2014, 111, 1021–1046. https://doi.org/10.1038/bjc.2014.166

11. R.M. Tyrrell, M. Pidoux, Photochem. Photobiol. 1988, 47, 405–412. https://doi.org/10.1111/j.1751-1097.1988.tb02744.x

12. V. Abbate, O. Reelfs, R.C. Hider, C. Pourzand, Biochem. J. 2015, 469, 357–366. https://doi.org/10.1042/BJ20150149

13. O. Reelfs, V. Abbate, R.C. Hider, C. Pourzand, J. Invest. Dermatol. 2016, 136, 1692–1700. https://doi.org/10.1016/j.jid.2016.03.041

14. M.D. Hanwell, D.E. Curtis, D.C. Lonie, T. Vandermeersch, E. Zurek, G.R. Hutchison, J. Cheminf. 2012, 4, 17.

15. ChemAxon, MarvinSketch 17.29.0; https://www.chemaxon.com

16. S.T. Mullapudi, C.S.M. Helker, G.L.M. Boezio, H.-M. Maischein, A.M. Sokol, S. Guenther, H. Matsuda, S. Kubicek, J. Graumann, Y.H.C. Yang, D.Y.R. Stainier, eLife 2018, 7, e42209. https://doi.org/10.7554/eLife.42209

17. N.D. Meeker, S.A. Hutchinson, L. Ho, N.S. Trede, BioTechniques 2007, 43, 610–614. https://doi.org/10.2144/000112619

18. M. Westerfield, The Zebrafish Book: A Guide for the Laboratory Use of Zebrafish (Danio rerio), 3rd ed.; University of Oregon Press: Eugene, OR, 1995, p. 385.
